# Stereospecific Nickel-Catalyzed Cross-Electrophile Coupling Reaction of Alkyl Mesylates and Allylic Difluorides to Access Enantioenriched Vinyl Fluoride-Substituted Cyclopropanes

**DOI:** 10.1021/acscatal.3c00257

**Published:** 2023-03-20

**Authors:** Patricia C. Lin, Chetan Joshi, Tristan M. McGinnis, Sharath Chandra Mallojjala, Amberly B. Sanford, Jennifer S. Hirschi, Elizabeth R. Jarvo

**Affiliations:** † Department of Chemistry, University of California, Irvine, California 92697, United States; ‡ Department of Chemistry, Binghamton University, Binghamton, New York 13902, United States

**Keywords:** cross-electrophile coupling, C−O bond activation, C−F bond activation, nickel, stereospecific, DFT calculations

## Abstract

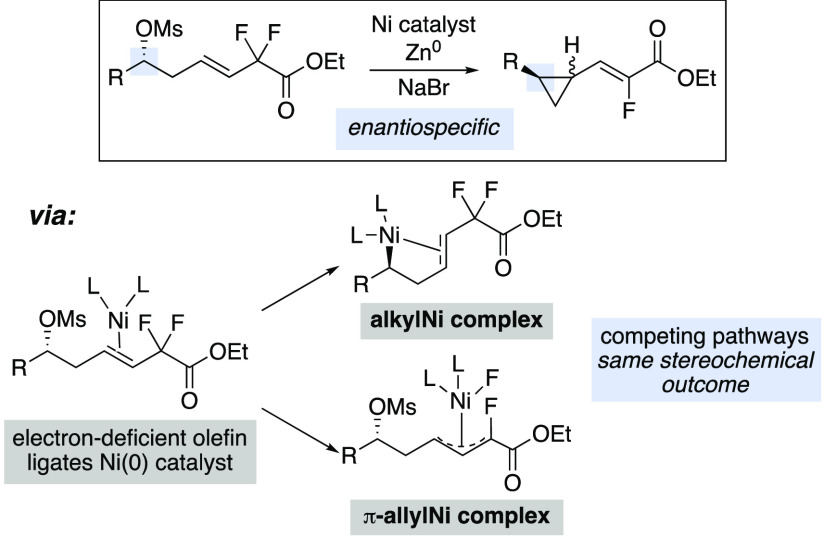

Cross-electrophile coupling reactions involving direct C–O bond activation of unactivated alkyl sulfonates or C–F bond activation of allylic *gem*-difluorides remain challenging. Herein, we report a nickel-catalyzed cross-electrophile coupling reaction between alkyl mesylates and allylic *gem*-difluorides to synthesize enantioenriched vinyl fluoride-substituted cyclopropane products. These complex products are interesting building blocks with applications in medicinal chemistry. Density functional theory (DFT) calculations demonstrate that there are two competing pathways for this reaction, both of which initiate by coordination of the electron-deficient olefin to the low-valent nickel catalyst. Subsequently, the reaction can proceed by oxidative addition of the C–F bond of the allylic *gem*-difluoride moiety or by directed polar oxidative addition of the alkyl mesylate C–O bond.

New methods for the synthesis of fluorinated compounds are important for medicinal chemistry and agrochemistry.^1^ For example, trisubstituted alkenyl fluorides serve as peptide bond mimics.^2^ One strategy for the synthesis of complex fluorinated moieties involves cross-coupling (XC) or cross-electrophile coupling (XEC) reactions of perfluorinated starting materials where one C–F bond is cleaved and others remain.^3^ For example, trifluoromethyl alkenes undergo XEC reactions, providing access to highly substituted difluorinated alkenes (Scheme 1a).^4−7^ These reactions typically employ a radical precursor and proceed via radical addition to the alkene followed by radical-polar crossover and β-fluoride elimination. Coupling reactions that use allylic geminal difluorides are less developed.^8,9^ Hayashi and co-workers have developed an enantioselective Suzuki XC reaction of allylic difluorides for the synthesis of complex α-fluoro,α,β-unsaturated carbonyls (Scheme 1b).^10^ To our knowledge, XEC reactions of allylic difluorides have not been established.^11^ We were interested in developing a new XEC reaction of allylic difluorides, building on our strategies for intramolecular XEC reactions of alkyl mesylates and alkyl fluorides,^12^ to access cyclopropanes bearing vinyl fluoride motifs. In this manuscript, we describe the development of an enantiospecific intramolecular XEC reaction of allylic *gem*-difluorides and alkyl mesylates to access enantioenriched vinyl fluoride-substituted cyclopropanes (Scheme 1c). In addition to establishing an XEC reaction involving allylic difluorides, this transformation affords synthetic access to enantioenriched cyclopropanes bearing fluorinated motifs, useful moieties for medicinal chemistry.^13^ Furthermore, we provide experimental and computational evidence that the reaction initiates through a polar mechanism, where alkyl radicals are not formed. Therefore, to the best of our knowledge, this reaction is the first XEC reaction of alkyl electrophiles with allylic perfluorides that proceeds by a polar mechanism, in contrast to radical-polar crossover. Additionally, mechanistic investigations demonstrate that the electron-deficient olefin serves as a directing group to facilitate oxidative addition (OA).

**Scheme 1:**
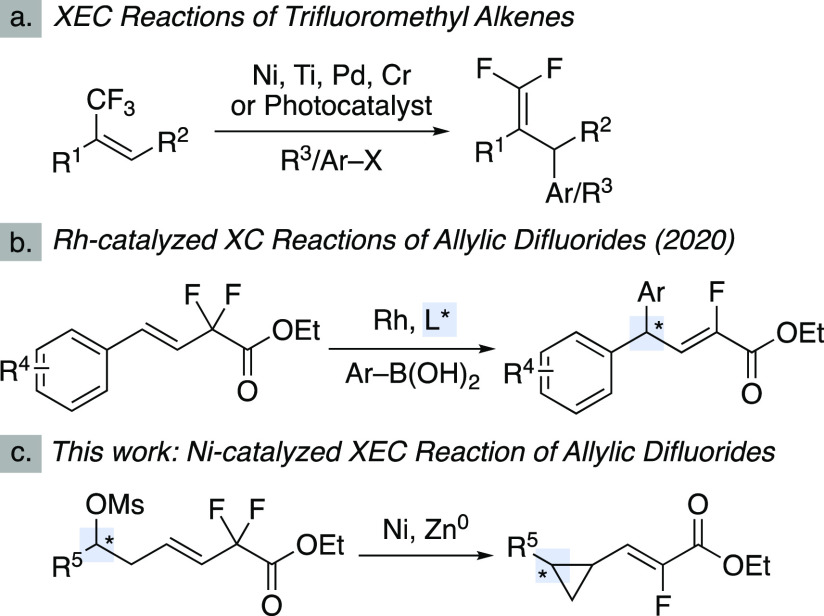
XC/XEC Reactions Involving Allylic Perfluorides

We chose mesylate **1a** as a test substrate to identify suitable reaction conditions (Table 1). Through evaluation of various nickel catalysts, additives, and solvents we discovered that Ni(PMe_3_)_2_Cl_2_, Zn^0^, and NaBr in acetonitrile afforded a 59% yield of the desired cyclopropane **2a** (entry 1). The cyclopropane moiety is formed with both *trans*- and *cis*- configuration, favoring the *trans* diastereomer, with the configuration of the alkene as exclusively the *Z*-isomer. Evaluation of several nickel precatalysts led us to identify Ni(PMe_3_)_2_Cl_2_ as the best catalyst for this transformation. Under slightly modified conditions, Ni(0) precatalysts such as Ni(cod)_2_ with *rac*-BINAP as ligand does provide the desired cyclopropane product, albeit in lower yield than when employing the Ni(II) precatalyst [(*R*)-BINAP]NiCl_2_. Once a suitable catalyst was identified, we investigated various additives. In the absence of NaBr, no product was formed (entry 2). Based on our prior work, we hypothesized that iodide salts could improve the reaction, since in the presence of iodide salts, the mesylate would be converted to an alkyl iodide in situ, and that this species would engage the nickel catalyst by XAT.^14^ However, use of NaI lowered the yield of the desired product (entry 3).^15^ Addition of MgI_2_ with NaI as the additive improved the yield relative to NaI alone but was not as high yielding as when employing NaBr (entry 5).^16^ We hypothesized that the magnesium salts assisted the transformation; however, addition of MgBr_2_ to the reaction employing NaBr did not improve the yield further (entry 6). Although zinc dust was chosen as the reductant for this transformation, we were pleased to see that employing manganese as the reductant provided a similar yield (entry 7). A control reaction showed that the nickel catalyst is necessary for this transformation (entry 8). We also investigated whether the ester moiety was necessary for reactivity and if the sterics of the ester group had any effect on the diastereoselectivity. *tert*-Butyl ester **1b** provided the desired cyclopropane in similar yield, but the diastereomeric ratio of the cyclopropane products decreased (entry 9). Replacing the ester with an amide in substrate **1c** shut down reactivity (entry 10).

**Table 1:**
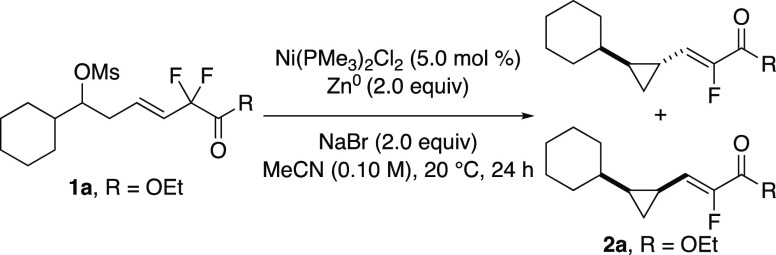
Optimization of the Reaction

entry	deviation from reaction conditions	recovered **1**^a^ (%)	yield **2**^a^ (%)	dr^a^
1	none	27	59	3.5:1
2	no NaBr	51	0	
3	NaI instead of NaBr	39	29	3.2:1
4	NaI instead of NaBr in THF^b^	14	35	3.5:1
5	NaI instead of NaBr, MgI_2_ added	0	54	3.7:1
6	MgBr_2_ (2.0 equiv) added	12	33	3.3:1
7	Mn instead of Zn	8	57	3.8:1
8	no Ni	66	0	
9	R = O*t*-Bu (**1b**)	39	50	1.8:1
10	R = NEt_2_ (**1c**)	57	0	

a Determined by ^1^H NMR based on comparison to PhTMS as internal standard.

b 0.025 M in tetrahydrofuran (THF).

With the optimized conditions in hand, we looked to establish the scope of this reaction (Scheme 2). A series of alkyl mesylates bearing a range of substituents reacted to provide the desired cyclopropane products in good yields. Pendant aryl substituents, including electron-rich and electron-poor arenes, were well tolerated (**3**–**6**). Substrates bearing heterocycles, such as substituted pyridine and benzodioxole also afforded cyclopropane products in moderate yields (**7** and **8**). The XEC reactions of unbranched alkyl substrates to give cyclopropanes **3**–**8** and **16** provided a mixture of diastereomers. We hypothesized that adding steric bulk near the mesylate center could increase diastereoselectivity. Gratifyingly, substrates with β-branching favored the formation of the *trans*-cyclopropane with up to 3.5:1 dr for cyclopropane **2a**. Various functional groups, such as Boc-protected piperidine and dioxane, were tolerated in the reaction to yield the cyclopropane products in moderate yields (**9** and **10**). Notably, sterically encumbered cyclopropanes were formed with good yields (**11**–**15**).^17^ Typically, recovered starting material is observed as the major byproduct in these reactions. For example, reactions to afford cyclopropanes **4** and **13** provided recovered starting material in 7 and 40% yields, respectively. Interestingly, in collaboration with the NIH-Developmental Therapeutics Program (DTP), we established that cyclopropane **13** exhibited antiproliferative activity against the HL-60 leukemia cell line.^18^

**Scheme 2:**
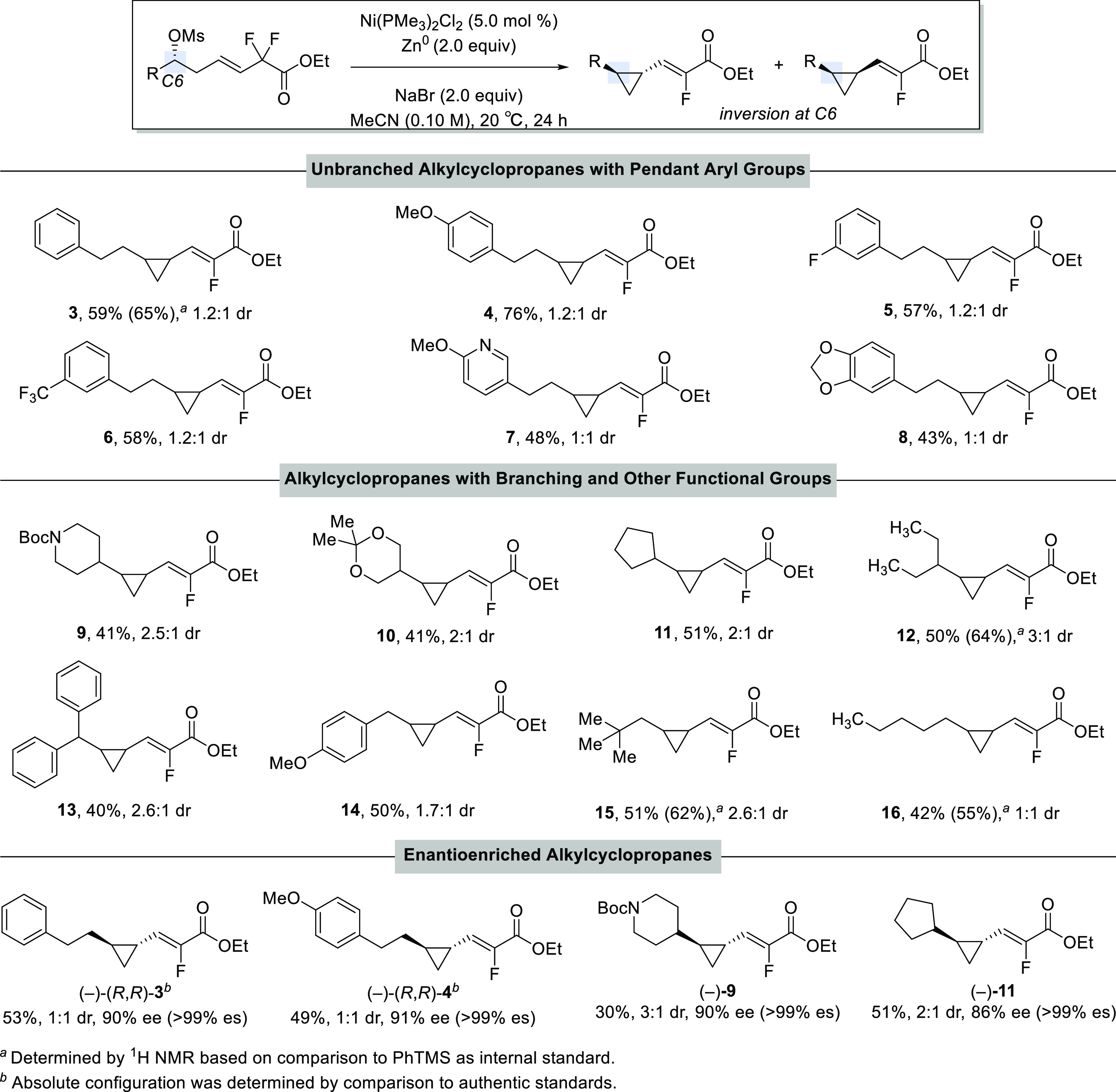
Substrate Scope

Typical nickel-catalyzed reactions of alkyl mesylates proceed through halide intermediates and stereoablative pathways.^19^ To probe the mechanism of this reaction and expand the utility of this method, we were interested in determining whether or not enantioenriched cyclopropane products could be synthesized using our reaction. We employed enantioenriched alkyl mesylates to test the reaction’s stereochemical outcome. Preparation of the requisite enantioenriched starting materials could be achieved by enantioselective allylation of aldehydes to set the carbinol stereocenters.^20^ We were surprised and pleased to see that employing enantioenriched alkyl mesylates in the reaction does afford alkylcyclopropanes with excellent stereospecificity. By comparison to authentic standards of *trans*-cyclopropanes **3** and **4**,^21^ we determined that the absolute configuration of the *trans*-cyclopropane formed from our XEC reaction is (*R*, *R*). Therefore, the reaction occurs with inversion at C-6. We were pleased to see that a range of alkyl- and aryl-substituted substrates reacted in a stereospecific manner to afford enantioenriched cyclopropane products (**3**, **4**, **9**, and **11**) in high enantiospecificity. In addition to providing access to enantioenriched products, this stereospecific reaction outcome is an unusual example of a stereospecific nickel-catalyzed reaction of an alkyl mesylate and gives us insight into the possible mechanistic pathways of this reaction (vide infra).

To show the ability to functionalize the vinyl fluoride-substituted cyclopropane products, further derivatization was evaluated (Scheme 3). A conjugate addition reaction installed a thiophenol by engaging the α-fluoro,α,β-unsaturated ester moiety, providing thioether **17** as a mixture of diastereomers.^22^ The ester moiety could also be manipulated. Ester **4** was subjected to reduction, hydrolysis, or a Grignard addition reaction to afford products **18**–**20** in good yield.

**Scheme 3:**
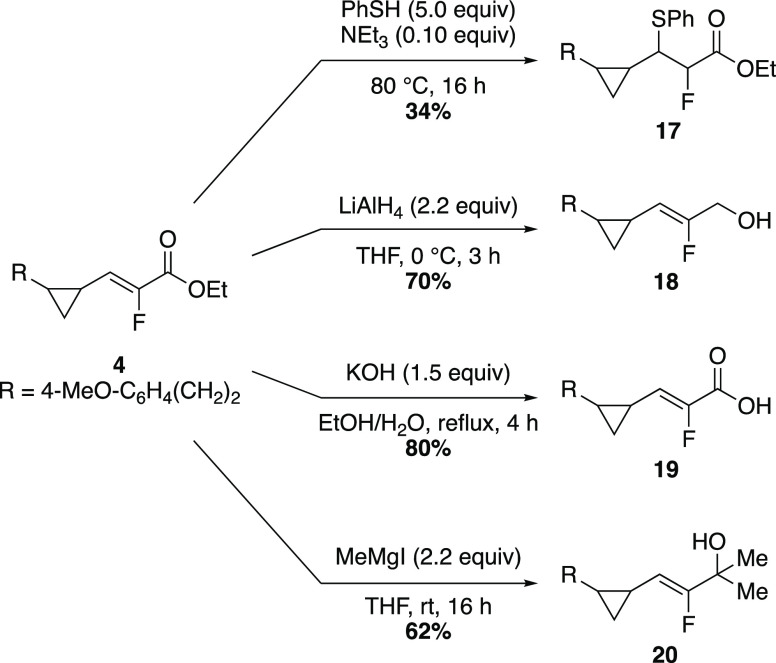
Derivatization of Reaction Product

## Possible Mechanistic Pathways

**Scheme 4:**
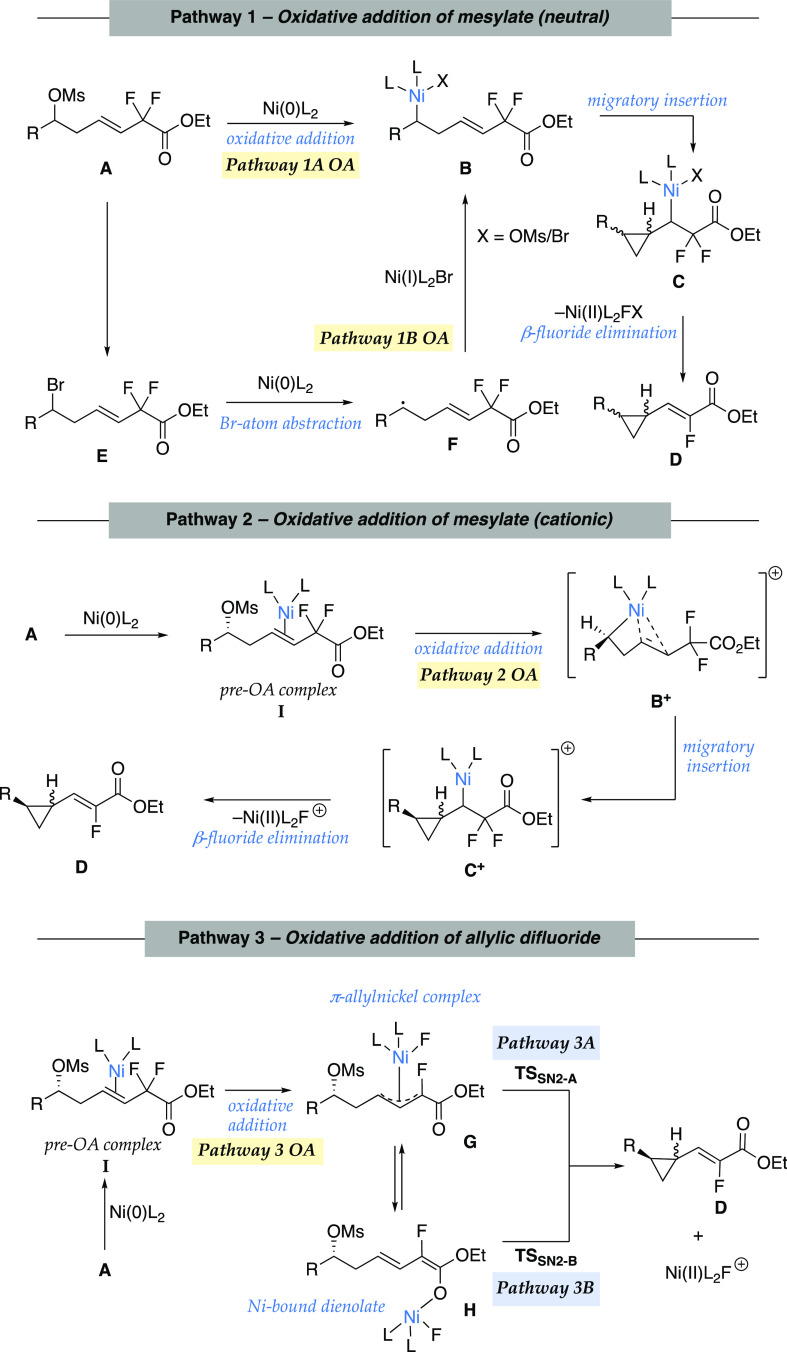
Key Mechanistic Pathways Evaluated Using Density Functional Theory (DFT) Calculations from Different Modes of Oxidative Addition

We envisioned several distinct pathways to access vinyl fluoride-substituted cyclopropanes based on which electrophilic functional group—the alkyl mesylate or the allylic *gem*-difluoride—engages the nickel catalyst to initiate the reaction (Scheme 4). Both Pathways 1A and 1B involve neutral pathways for oxidative addition of the alkyl mesylate or alternatively an alkyl bromide intermediate. Pathway 1A is initiated by stereoretentive oxidative addition of Ni(0)L_2_ into the secondary alkyl mesylate (**A**). Following oxidative addition (OA), migratory insertion (MI) of the alkyl Ni(II) complex **B** into the alkene forms α-cyclopropyl Ni(II) intermediate **C**. Subsequent β-fluoride elimination (BFE) delivers the final product, vinyl cyclopropane **D**. An alternative mechanism, Pathway 1B is initiated by S_N_2 displacement of the mesylate by bromide anion to give secondary alkyl bromide **E**. In this event, oxidative addition occurs to **E** via nickel-mediated halogen-atom abstraction.^23^ Addition of the resultant alkyl radical **F** to the Ni(I)L_2_Br species generates Ni(II) intermediate **B** (Pathway 1B). Subsequent MI and BFE (similar to Pathway 1A) deliver the vinyl cyclopropane **D**.

Next, we considered Pathway 2, where an alkene-bound Ni(0)L_2_ displaces the mesylate through a stereoinvertive oxidative addition in an S_N_2 fashion to generate the cationic Ni(II) complex **B**^**+**^.^24^ The pathway proceeds through the intramolecular MI of intermediate **B**^**+**^ to form the cationic α-cyclopropyl Ni(II) intermediate **C**^**+**^. Subsequent BFE and catalyst dissociation deliver product **D** with overall inversion at the mesylate center, which is consistent with the stereochemical outcome of this reaction.

Finally, we considered a pathway initiated by the oxidative addition of the allylic *gem*-difluoride moiety (Pathway 3). It has been shown that palladium catalysts can activate C–F bonds of allylic *gem*-difluorides to generate fluorinated π-allylpalladium complexes.^25^ The Ni analogue of this mode of allylic C–F bond activation is outlined in Pathway 3. It is important to note that Pathway 3 is fundamentally different from Pathways 1 and 2 in terms of the initial site of catalyst engagement with the substrate. The Ni(0)L_2_ catalyst undergoes OA with the allylic *gem*-difluoride (**A**) to form the fluorinated π-allylnickel complex **G**. Direct intramolecular S_N_2-attack of the π-allylnickel species on the alkyl mesylate carbon delivers the vinyl fluoride-substituted cyclopropane **D** (Pathway 3A). Alternatively, π-allylnickel complex **G** can isomerize to form a nickel dienolate intermediate **H**. With the nickel catalyst bound to the carbonyl oxygen, **H** delivers product **D** via an intramolecular S_N_2-type ring-closure step (Pathway 3B).

## DFT Evaluation of Mechanistic Pathways

We set out to establish the key mechanistic features of this XEC reaction by conducting a DFT analysis of the proposed pathways shown in Scheme 4. We chose the title reaction involving cyclohexyl-substituted secondary alkyl mesylate **1a** as the model substrate for our theoretical studies as this substrate afforded the highest diastereoselectivity of cyclopropane product (see Table 1, entry 1, *trans*/*cis* ratio of 3.5:1). DFT computations were performed using the B3LYP-D3BJ/def2TZVP PCM(acetonitrile)//B3LYP-D3/def2SVP SMD(acetonitrile) level of theory.^26^ A thorough conformational search was performed for each transition structure (TS) (for details, see Supporting Information Figures S1, S9, and S10, and for a benchmark of various computational methods, see Supporting Information Section III.E). Intrinsic reaction coordinate (IRC) calculations were performed to confirm the transition structures (TSs) connect minima along the potential energy surface. Our general approach involved the exploration of several possibilities for the initial engagement of the Ni catalyst with **A** to evaluate whether the respective pathways are energetically accessible. Computational models were further refined based on the prediction of the product stereochemistry—experimentally an overall inversion is observed at the mesylate stereocenter. A complete reaction coordinate was calculated for pathways that were deemed energetically feasible. At the outset, all reaction pathways were compared to the lowest-energy catalyst–substrate complex (pre-OA complex, **I**). In this complex, the electron-deficient olefin serves as a ligand for the nickel(0) catalyst, a strong interaction.^27^

## Pathway 1

**Figure 1:**
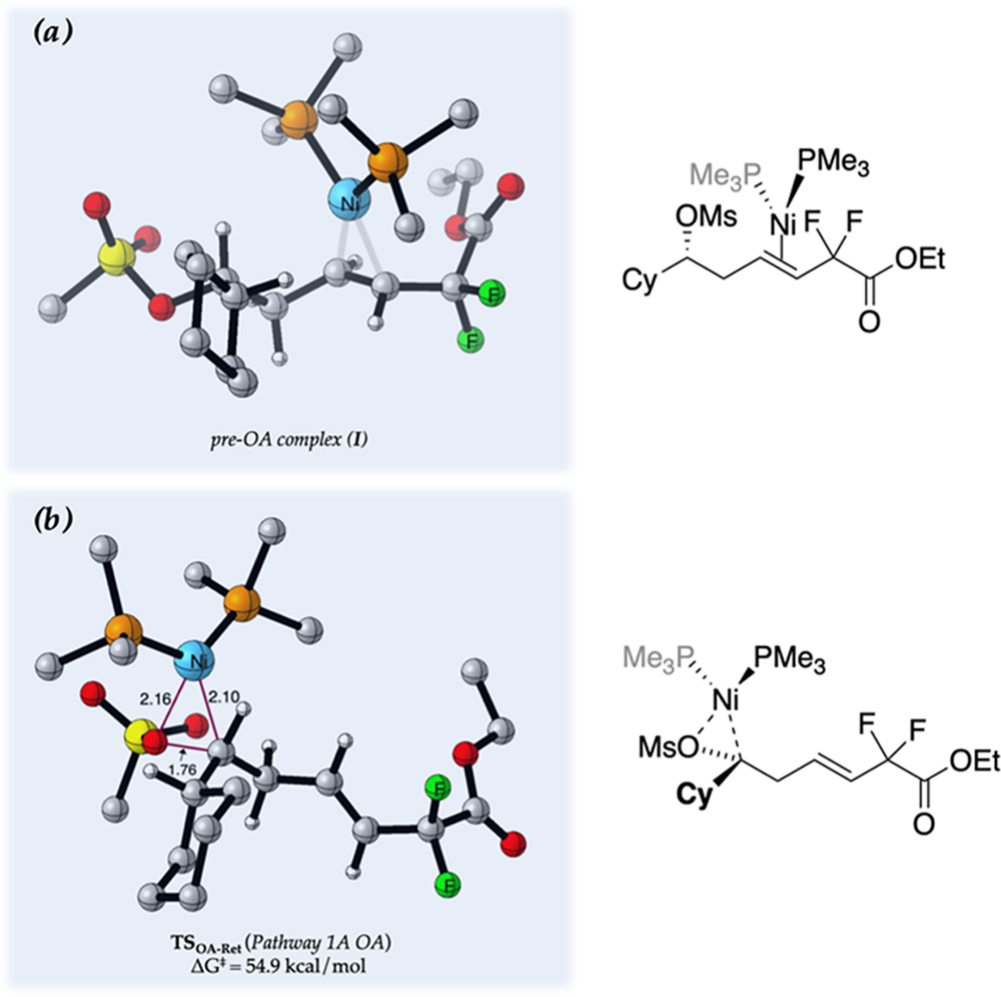
(a) Lowest-energy catalyst–substrate complex, pre-OA complex **I**. (b) Relevant oxidative addition transition structure for competing Pathway 1A.

Pathway 1A is initiated through the stereoretentive oxidative addition of the nickel catalyst with the mesylate moiety. The transition structure (**TS**_**OA-Ret**_, Figure 1) described in Pathway 1A is prohibitively high in energy: Δ*G*^‡^ = 54.9 kcal/mol with respect to the lowest-energy catalyst–substrate complex (pre-OA complex, **I**). Additionally, a reaction proceeding via **TS**_**OA-Ret**_ delivers product **D** with overall retention at the mesylate center, which is inconsistent with the experimentally observed inversion at this center. These two observations allow us to easily rule out Pathway 1A as the operational mechanism in this reaction. We also ruled out Pathway 1B for several reasons: (i) the reaction is stereospecific, proceeding with inversion at the alkyl stereogenic center, inconsistent with radical intermediates; (ii) replacing NaBr with iodide salts provided more complex and lower-yielding reaction mixtures, inconsistent with formation of key halide intermediates (vide supra, Table 1 entries 3–5); and (iii) the calculated barrier heights for the halogen-mediated pathway are relatively high.^28^

Pathway 1 was eliminated based on energetics and stereochemical outcome. However, Pathways 2 and 3 are both consistent with the observed stereochemical outcome of the reaction; therefore, we proceeded to model all steps in the catalytic cycle to identify the turnover-limiting and selectivity-determining steps in these pathways. The results from these calculations are described below.

## Pathway 2

**Figure 2:**
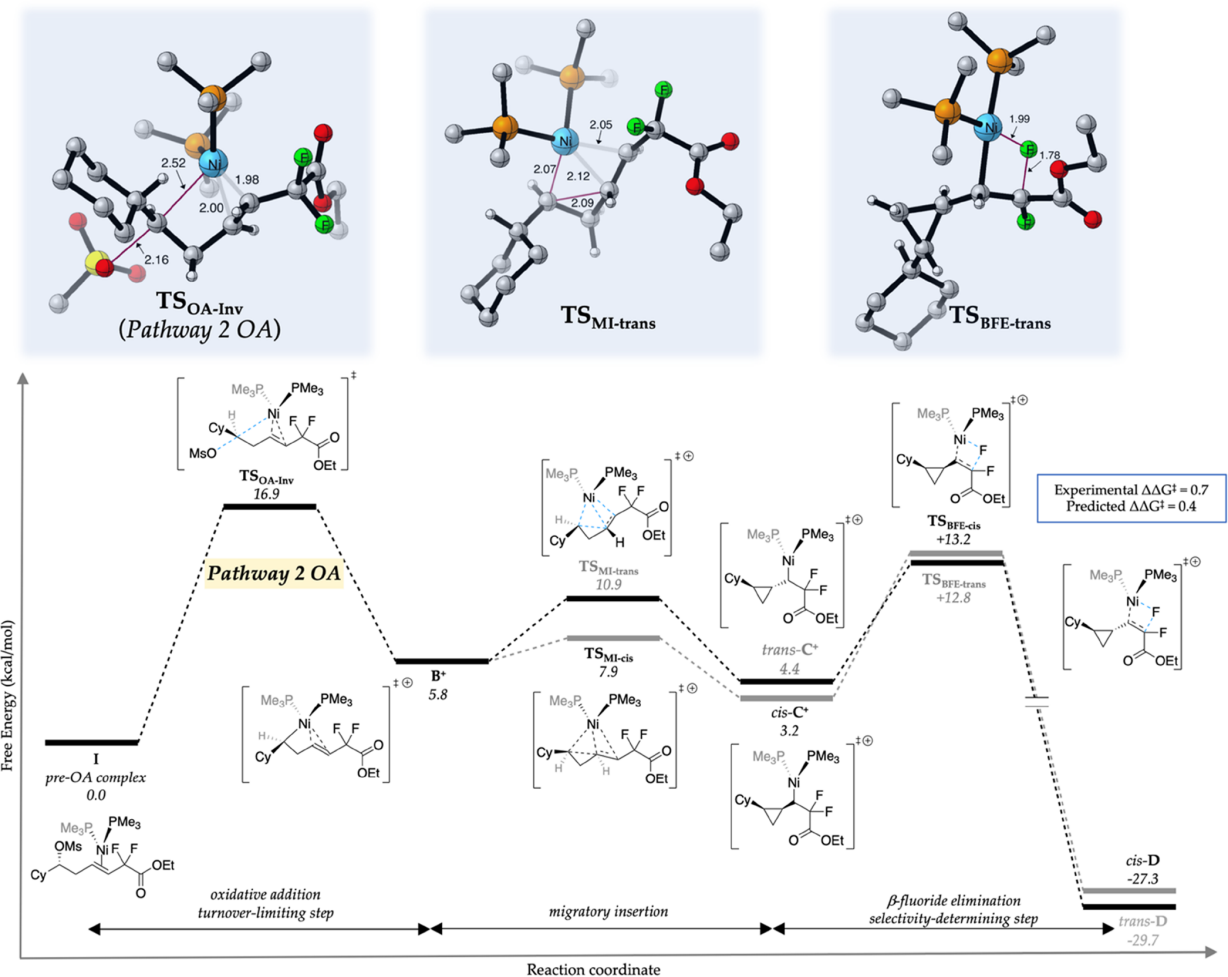
Calculated free energies for the reaction coordinate of Pathway 2. The geometric features of key transition structures leading to the major product (*trans*-cyclopropane) are shown above the respective steps. All distances are in angstroms.

The reaction coordinate for cationic Pathway 2 (Figure 2) begins with the lowest-energy catalyst–substrate complex (pre-OA complex, **I**), where the electron-deficient olefin serves as a ligand for the nickel(0) catalyst. This coordination can be considered a directing group for stereoinvertive OA at the mesylate center (**TS**_**OA-Inv**_), which occurs with a free energy barrier of only 16.9 kcal/mol versus the pre-OA complex (**I**). The **TS**_**OA-Inv**_ involves a backside displacement of the mesylate to give cationic nickel(II) intermediate **B**^**+**^ (the Ni–C bond-forming distance is 2.52 Å and the C–O bond-breaking distance is 2.16 Å). Following **TS**_**OA-Inv**_, we modeled transition structures for both the MI (**TS**_**MI-trans**_) and BFE (**TS**_**BFE-trans**_) steps leading to the formation of the major product (*trans*-**D**)—the computed barriers for these TSs are 10.9 and 12.8 kcal/mol, respectively, relative to pre-OA complex (**I**). Analysis of the full reaction coordinate for Pathway 2 leading to *trans*-**D** reveals that (a) **TS**_**OA-Inv**_ is the turnover-limiting step, (b) migratory insertion is reversible, and (c) β-fluoride elimination is the selectivity-determining step in the catalytic cycle. To obtain theoretical insight into the origin of *cis*/*trans* selectivity in this reaction, we also modeled transition structures for both the MI (**TS**_**MI-cis**_) and BFE (**TS**_**BFE-cis**_) steps leading to the formation of the minor product (*cis*-**D**). Intriguingly, while **TS**_**MI-cis**_ is 3.0 kcal/mol lower in energy than **TS**_**MI-trans**_, the selectivity-determining **TS**_**BFE-cis**_ is 0.4 kcal/mol higher in energy than **TS**_**BFE-trans**_—a ΔΔ*G*^‡^ value that is in reasonable agreement with the experimental ΔΔ*G*^‡^ of 0.7 kcal/mol (3.5:1 trans:cis). Therefore, since migratory insertion is reversible, the major pathway leading to product proceeds through the higher barrier migratory insertion (**TS**_**MI-trans**_) and the lower barrier BFE (**TS**_**BFE-trans**_), although the selectivity is modest compared to the diastereomeric pathway. Overall, we conclude that Pathway 2 is energetically accessible, consistent with the observed inversion at the mesylate center, and accurately predicts the *cis*/*trans* selectivity in this reaction.

## Pathway 3

**Figure 3:**
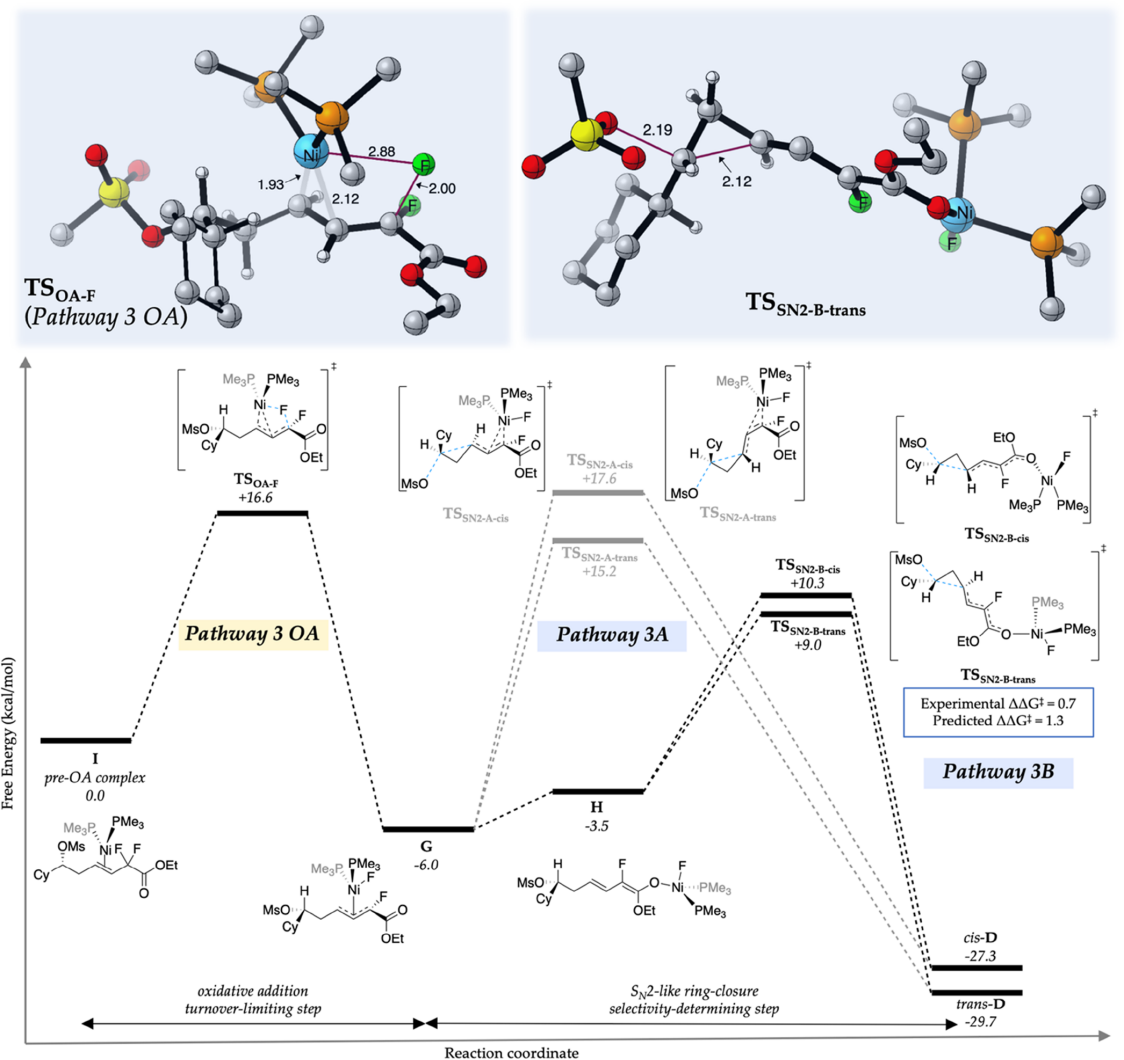
Calculated free energies for the reaction coordinate of Pathway 3. The geometric features of key transition structures leading to the major product (trans-cyclopropane) are shown above the respective steps. All distances are in angstrom.

Like Pathway 2, Pathway 3 also begins with the lowest-energy catalyst–substrate complex (pre-OA complex, **I**), where the electron-poor olefin serves as a ligand for the nickel(0) catalyst (Figure 3). We found that oxidative addition into the allylic *gem*-difluoride (**TS**_**OA-F**_) is also a viable mode of initial engagement of the Ni(0)L_2_ complex with a free energy barrier of only 16.6 kcal/mol (versus pre-OA complex, **I**) which delivers intermediate **G**—a π-allylnickel(II) fluoride complex (**TS**_**OA-F**_, the Ni–F bond-forming distance is 2.88 Å and the F–C bond-breaking distance is 2.00 Å, respectively). Along Pathway 3A, direct intramolecular S_N_2-type ring closure from the π-allylnickel(II) complex **G** can deliver either *cis*-**D** via **TS**_**SN2-A-cis**_ (Δ*G*^‡^ = 17.6 kcal/mol) or *trans*-**D** via **TS**_**SN2-A-trans**_ (Δ*G*^‡^ = 15.2 kcal/mol). However, if **G** isomerizes to the Ni-bound dienolate **H**,^29,30^ entering Pathway 3B, intramolecular S_N_2-type ring closure from **H** via **TS**_**SN2-B-cis**_ (Δ*G*^‡^ = 10.3 kcal/mol) or **TS**_**SN2-B-trans**_ (Δ*G*^‡^ = 9.0 kcal/mol) occurs more readily than Pathway 3A. Analysis of the full reaction coordinate for Pathway 3B suggests that **TS**_**OA-F**_ is the turnover-limiting step, while S_N_2-type ring closure from the nickel dienolate intermediate is the diastereoselectivity-determining step in the catalytic cycle. The ΔΔ*G*^‡^ of 1.3 kcal/mol between the **TS**_**SN2-B-cis**_ and **TS**_**SN2-B-trans**_ is within reasonable agreement of the experimental ΔΔ*G*^‡^ of 0.7 kcal/mol (3.5:1 trans:cis), and is thus consistent with the modest level of diastereoselectivity. Based on the decomposition analysis for Pathway 2, the catalyst fragments for the competing TSs show much smaller distortions for the minor product (**TS**_**BFE-cis**_) while simultaneously undergoing significantly large unfavorable distortions (∼3.0 kcal/mol) for the reactant fragment. However, for Pathway 3, the TS leading to the major product (**TS**_**SN2-B-trans**_) enjoyed fewer destabilizing distortions (1.5 kcal/mol) compared to the TS leading to the minor product (**TS**_**SN2-B-cis**_). For a detailed analysis of key transition structures of the selectivity-determining steps for Pathways 2 and 3, see Supporting Information Section III G.

From the evaluation of the free energy profiles for Pathways 2 and 3 (vide supra), it is evident that both pathways are energetically accessible and consistent with the overall stereochemical outcome of the reaction. The calculated barriers for the turnover-limiting events in each pathway are the initial oxidative addition steps (**TS**_**OA-Inv**_ and **TS**_**OA-F**_), which are within computational error of each other, suggesting that initial engagement of the nickel catalyst could simultaneously occur at either one of the electrophilic sites in **A**. This result opens exciting opportunities for ligand control in the site selectivity for oxidative addition in this reaction by selective stabilization of one of the two accessible pathways.

To gain additional insight into the origin of the free energy barriers for the respective turnover-limiting steps, we performed a detailed energy decomposition analysis for **TS**_**OA-Inv**_ (Pathway 2) and **TS**_**OA-F**_ (Pathway 3), the results of which are described in the following section.

## Transition State Analysis of Turnover-Limiting Oxidative Additions in Pathways 2 and 3

**Figure 4:**
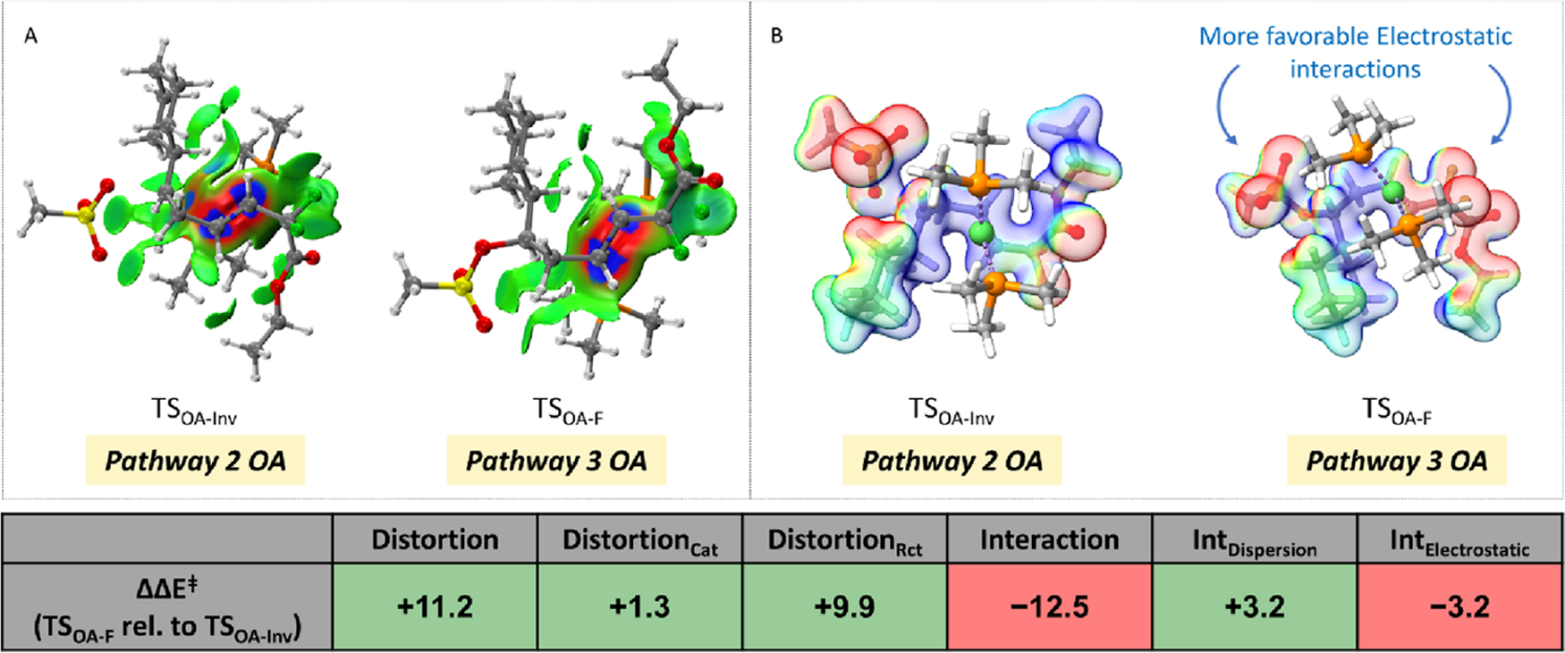
(A) Noncovalent interaction plots for the lowest-energy OA TSs. The green surfaces indicate weak van der Waals interactions (isovalue 0.008). (B) Qualitative depiction of the stabilizing electrostatic interactions operative in the oxidative addition TSs [electrostatic potential (ESP) range of −12.5 kcal/mol (red) to +12.5 kcal/mol (blue)]. Table: All energies are displayed as the relative energy of **TS**_**OA-F**_ to **TS**_**OA-Inv**._ The energies in green shaded boxes indicate the components from energy decomposition analysis favoring **TS**_**OA-Inv**_ (Pathway 2), while red shaded boxes in the table indicate the components favoring **TS**_**OA-F**_ (Pathway 3).

We performed distortion-interaction analysis^31,32^ on the oxidative addition transition states, **TS**_**OA-Inv**_ and **TS**_**OA-F**_ (Figure 4). This analysis decomposes the transition state energy into a distortion term, i.e., the energy needed for the separated reactants in their ground-state geometries to distort into the geometries resembling the transition structure, and an interaction term, i.e., the interactions between these distorted geometries as they come together to form the transition state. The **TS**_**OA-F**_ (Pathway 3) is disfavored by greater distortions for both the catalyst fragment (by 1.3 kcal/mol) and the reactant fragment (by 9.9 kcal/mol) compared to the **TS**_**OA-Inv**_ (Pathway 2) (see table in Figure 4 and a more detailed analysis in Supporting Information Section III.F). On the other hand, the interaction energy favors **TS**_**OA-F**_ by 12.5 kcal/mol over **TS**_**OA-Inv**_. This interaction energy was further decomposed into dispersion^33^ (Figure 4A) and electrostatic^34^ interaction energies (Figure 4B). Evaluating the dispersion interactions between the catalyst fragment (Ni(PMe_3_)_2_) and reactant fragment (**A**) for both oxidative addition pathways reveals 3.2 kcal/mol worth of favorable dispersion interactions in **TS**_**OA-Inv**_ compared to **TS**_**OA-F**_. Qualitative visualization of van der Waals interactions operative in these transition structures is evident by the presence of green surfaces in Figure 4A for **TS**_**OA-Inv**_ and **TS**_**OA-F**_.^35^ Finally, evaluating the electrostatic stabilization term between the catalyst and the reactant yields a 3.2 kcal/mol stabilization for **TS**_**OA-F**_ compared to **TS**_**OA-Inv**_. The leftover steric and electronic components of the interaction energy (i.e., Pauli repulsion, charge transfer, and polarization) favor **TS**_**OA-F**_. These results indicate that suitable modifications to the dispersion or electrostatic components of the reactants could allow the tuning of the oxidative addition step to favor either the stereoinvertive S_N_2-type pathway or the OA of the allylic *gem*-difluoride. A similar analysis was carried out to identify the origin of diastereoselectivity for pathways 2 and 3 as detailed in the Supporting Information (page SI 77–78). Unsurprisingly, this analysis revealed the role of steric interactions common to reactions of this class.

In summary, we report a Ni-catalyzed XEC reaction of alkyl mesylates with allylic *gem*-difluorides to access cyclopropanes bearing vinyl fluoride motifs, a potentially useful moiety in medicinal chemistry as highlighted by the anticancer activity of cyclopropane **13**. The reaction is tolerant of various functional groups, where greater diastereoselectivity of the cyclopropane diastereomers can be achieved by adding steric bulk around the mesylate center. Additionally, this reaction allows for the synthesis of enantioenriched cyclopropanes in high enantiospecificity. A mechanistic analysis utilizing DFT calculations identifies two possible competing pathways for this reaction that both proceed with inversion at the mesylate center. One possible pathway initiates with polar oxidative addition of the C–O bond of the alkyl mesylate, directed by the pendant electron-deficient olefin. Alternatively, the reaction may begin with oxidative addition of the allylic *gem*-difluoride moiety. Based on calculations, we propose that both oxidative additions proceed from the same olefin-ligated complex, and one pathway is favored by dispersion interactions while the other is favored by electrostatic interactions. Further investigation of the factors that influence the competing oxidative addition pathways, as well as the development of related reactions, is ongoing in our laboratories.

## Supporting Information Available

Full experimental procedures and characterization data as well as copies of ^1^H and ^13^C NMR spectra for all new compounds; full details of computational methods (PDF)
Coordinates for all structures (XYZ)

## Supplementary Material

cs3c00257_si_001.pdf

cs3c00257_si_002.xyz
