# Supplementary material for: Stereospecific Nickel-Catalyzed Cross-Electrophile Coupling Reaction of Alkyl Mesylates and Allylic Difluorides to Access Enantioenriched Vinyl Fluoride-Substituted Cyclopropanes
Source: ACS Catal. 2023 Mar 20;13(7):4488–99. doi: 10.1021/acscatal.3c00257 (PMC10088041; doi:10.1021/acscatal.3c00257)
Supplement: Supplementary file 1 — cs3c00257_si_001.pdf [file cs3c00257_si_001.pdf]

**Supporting Information**

# Stereospecific Nickel-Catalyzed Cross-Electrophile Coupling Reaction of Alkyl Mesylates and Allylic Difluorides to Access Enantioenriched Vinyl Fluoride-Substituted Cyclopropanes

Patricia C. Lin,[‡,†] Chetan Joshi,[‡,∥] Tristan M. McGinnis,[†] Sharath Chandra Mallojjala,[∥] Amberly B. Sanford,[†] Jennifer S. Hirschi,[*,∥] Elizabeth R. Jarvo[*,†]

[†] *Department of Chemistry, University of California, Irvine, California 92697*
[∥] *Department of Chemistry, Binghamton University, Binghamton, New York 13902*

*Corresponding authors: jhirschi@binghamton.edu; erjarvo@uci.edu

## Table of Contents

| | | |
|---|---|---|
| **I.** | **General Procedures** | SI-2 |
| **II.** | **Experimental** | SI-3 |
| | A. General Cross-Electrophile Coupling (XEC) Reaction Procedure | SI-3 |
| | Method A: XEC for Synthesis of Alkylcyclopropanes | SI-3 |
| | B. Proof of Stereochemical Outcome | SI-3 |
| | C. Characterization Data for Products in Proof of Stereochemical Outcome | SI-4 |
| | D. Characterization Data for Cyclopropanes **2–16** | SI-11 |
| | 1) Racemic Cyclopropanes | SI-11 |
| | 2) Enantioenriched Cyclopropanes | SI-22 |
| | E. General Procedures for Starting Material Synthesis | SI-23 |
| | Method B: Copper-catalyzed Alkene Functionalization | SI-23 |
| | Method C: Mesylation of Alcohol | SI-23 |
| | Method D: Triphenylsilylation of Alcohol | SI-24 |
| | F. Synthesis and Characterization Data of Intermediates and Mesylates | SI-24 |
| | 1) Intermediates and Mesylates for Racemic Cyclopropanes | SI-24 |
| | 2) Intermediates and Mesylates for Enantioenriched Cyclopropanes | SI-47 |
| | G. Characterization Data of Products from Derivatization of Cyclopropane **4** | SI-53 |
| **III.** | Computational Data for Mechanistic Studies | SI-55 |
| | A. Computational Methods | SI-55 |
| | B. Exploration of Oxidative Addition Transition Structures | |
| | 1) Stereoinvertive vs. Stereoretentive Oxidative Addition of $Ni(0)L_2$ into the Secondary Alkyl Mesylate | SI-57 |
| | 2) Oxidative Addition of $Ni(0)L_2$ into the Allylic *gem*-Difluoride And at the Secondary Alkyl Center | SI-58 |
| | 3) Pathway #1B Halogen Atom Abstraction vs. Oxidative Addition of $Ni(0)L_2$ into the Secondary Alkyl Bromide Center | SI-61 |
| | 4) $S_N2$ Displacement of Mesylate by Bromide | SI-62 |

C. Origin of Selectivity
1) Pathway #2 SI-63
2) Pathway #3 SI-65
D. Pathway 2 cationic vs neutral pathway SI-68
E. Comparison of Various Computational Methods SI-69
F. Analysis of Key Transition Structures for Oxidative Addition SI-76
G. Analysis of key transition structures for selectivity determining step for pathway 2 and 3 SI-77
H. Energetics for Different Possible Conformers SI-78
**IV. References for Supporting Information** SI-83
**V. $^1$H, $^{13}$C, $^{19}$F, COSY, and NOE NMR Spectra, HPLC, and SFC Traces** SI-85

## I. GENERAL PROCEDURES

All reactions were carried out under an atmosphere of $N_2$ when noted. All glassware was oven- or flame-dried prior to use. Tetrahydrofuran (THF), diethyl ether ($Et_2O$), dichloromethane (DCM), acetonitrile (MeCN), dimethylformamide (DMF), and toluene (PhMe) were degassed with Ar and then passed through two 4 x 36-inch columns of anhydrous neutral A-2 alumina (8 x 14 mesh; LaRoche Chemicals; activated under a flow of argon at 350 °C for 12 h) to remove $H_2O$. All other solvents utilized were purchased "anhydrous" commercially or purified as described. $^1$H NMR spectra were recorded on Bruker DRX-400 (400 MHz $^1$H, 100 MHz $^{13}$C, 376.5 MHz $^{19}$F), GN-500 (500 MHz $^1$H, 125.7 MHz $^{13}$C), CRYO-500 (500 MHz $^1$H, 125.7 MHz $^{13}$C), or AVANCE-600 (600 MHz $^1$H, 150.9 MHz $^{13}$C, 564.7 MHz $^{19}$F) spectrometers. Proton chemical shifts are reported in ppm (δ) relative to internal tetramethylsilane (TMS, δ 0.00). Data are reported as follows: chemical shift (multiplicity [singlet (s), broad singlet (br s), doublet (d), doublet of doublets (dd), doublet of doublet of doublets (ddd), triplet (t), doublet of triplets (dt), doublet of quartets (dq), triplet of doublets (td), quartet (q), quintet (quint), sextet (sext), multiplet (m), apparent singlet (ap s), apparent doublet (ad), apparent triplet (at), apparent quartet (aq), apparent quintet (aquint)], coupling constants [Hz], integration). Carbon chemical shifts are reported in ppm (δ) relative to TMS with the respective solvent resonance as the internal standard ($CDCl_3$, δ 77.16 ppm). NMR data were collected at 25 °C. Analytical thin-layer chromatography (TLC) was performed using Silica Gel 60 F254 precoated plates (0.25 mm thickness). Visualization was accomplished by irradiation with a UV lamp or stain if noted. Flash chromatography was performed using SilicaFlash F60 (40-63 µm, 60 Å) from SiliCycle. Optical rotations were measured on a Rudolph Research Analytical Autopol III Automatic Polarimeter. SFC determinations of enantiopurity were determined by chiral SFC and HPLC analysis and performed on an Agilent Technologies HPLC (1260 series) system or Agilent Technologies HPLC (1200 series) Analytical Instruments using OJ-H, OD-H, OD, and AD Chiralpak columns (100 bar, 50 ºC, 254 nm). High resolution mass spectrometry was performed by the University of California, Irvine Mass Spectrometry Center.

Bis(1,5-cyclooctadiene)nickel was purchased from Strem, stored in a glovebox freezer (–20 °C) under an atmosphere of $N_2$ and used as received. All ligands were purchased from Strem or Sigma Aldrich and were stored under $N_2$ atmosphere and used as received. All other chemicals were purchased commercially and used as received, unless otherwise noted.

# II. EXPERIMENTAL

## A. General Cross-Electrophile Coupling (XEC) Reaction Procedure:

Method A: XEC for Synthesis of Alkylcyclopropanes

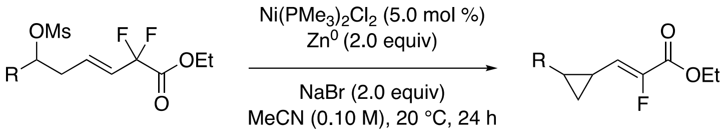


In a glovebox, an oven-dried 7-mL vial equipped with a stir bar was charged with substrate (1.0 equiv), $Ni(PMe_3)_2Cl_2$ (5.0 mol %), $Zn^0$ (2.0 equiv), NaBr (2.0 equiv), and anhydrous MeCN (0.10 M in substrate). The reaction mixture will have a murky gray-yellow color if it is proceeding without problem. After 24 h, the reaction was removed from the glovebox, filtered through a plug of silica gel (loaded with DCM, allowed to sit for 5 min, eluted with 20% EtOAc/hexanes, then eluted with $Et_2O$), and concentrated in vacuo. Phenyltrimethylsilane (PhTMS; 8.6 $\mu$L, 50. $\mu$mol) was added to determine the yield by $^1$H NMR based on comparison to PhTMS as internal standard before purification by flash column chromatography.

## B. Proof of Stereochemical Outcome:

The absolute configuration of the cyclopropane products from the XEC reaction of enantioenriched mesylate **SI-12** and **SI-13** were assigned. The experiments are summarized below. In both examples, we conclude that the XEC reaction proceeds with single inversion at the mesylate center.

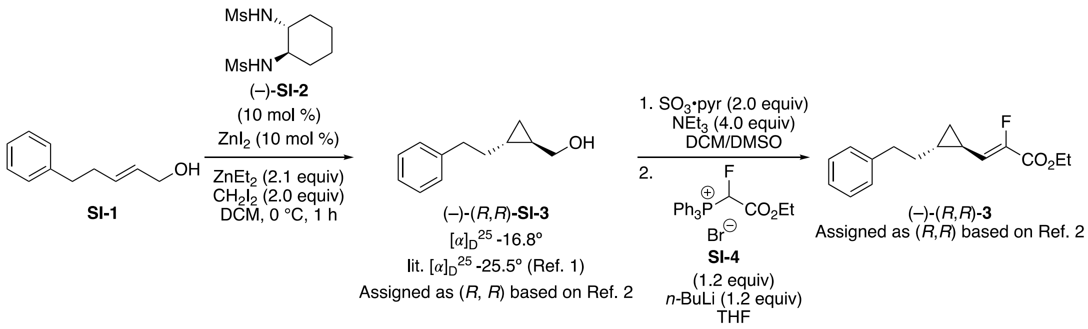


**Scheme SI-1:** Proof of Stereochemical Outcome for Cyclopropane **3**

**Allylic alcohol (SI-1)** was prepared by a Horner-Wadsworth-Emmons (HWE) reaction of hydrocinnamaldehyde, followed by reduction of the ester with diisobutylaluminum hydride. Enantioselective Simmons-Smith reaction gave cyclopropane (*R,R*)-**SI-3**, and the stereochemistry was verified by comparison of the optical rotation to the literature value.[i,ii] Conversion to the aldehyde by oxidation using $SO_3$•pyridine, followed by a Wittig olefination produced cyclopropane (–)-(*R,R*)-**3**, the stereochemistry of which is predicted to be conserved from cyclopropane (–)-(*R,R*)-**SI-3**. The stereochemistry of the product from the XEC reaction of enantioenriched mesylate **SI-12** was determined by comparison of the SFC trace of cyclopropane (–)-(*R,R*)-**3** to the SFC trace of the product from the XEC reaction of enantioenriched mesylate **SI-12**. This product corresponds to single inversion at the mesylate center in the XEC reaction.

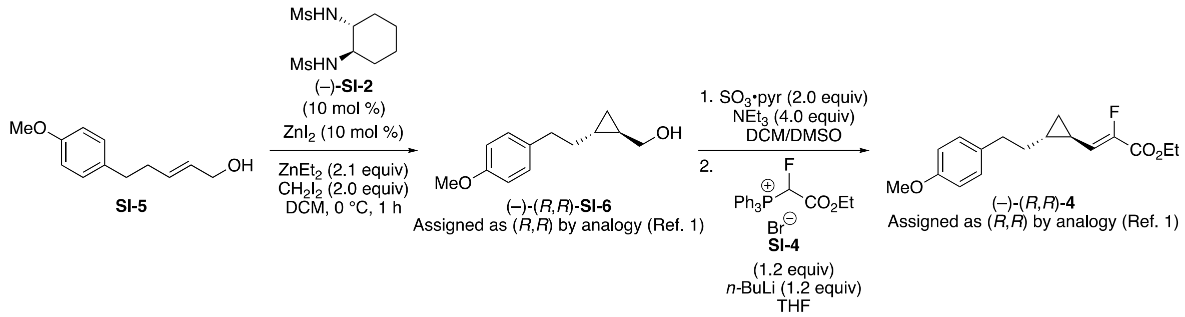


**Scheme SI-2:** Proof of Stereochemical Outcome for Cyclopropane **4**

**Allylic alcohol (SI-5)** was prepared by a Horner-Wadsworth-Emmons (HWE) reaction of 3-(4-methoxyphenyl)propional, which was prepared via a Heck reaction of 3-iodoanisole, followed by reduction of the ester with diisobutylaluminum hydride. Enantioselective Simmons-Smith reaction gave cyclopropane (–)-(*R,R*)-**SI-6**, and the stereochemistry was assigned by analogy to similar compounds synthesized by Denmark.[1] Conversion to the aldehyde by oxidation using $SO_3$•pyridine, followed by a Wittig olefination produced cyclopropane (–)-(*R,R*)-**4**, the stereochemistry of which is predicted to be conserved from (–)-(*R,R*)-**SI-6**. The stereochemistry of the product from the XEC reaction of enantioenriched mesylate **SI-13** was determined by comparison of the HPLC trace of cyclopropane (–)-(*R,R*)-**4** to the HPLC trace of the product from the XEC reaction of enantioenriched mesylate **SI-13**. This product corresponds to single inversion at the mesylate center in the XEC reaction.

## C. Characterization Data of Products in Proof of Stereochemical Outcome

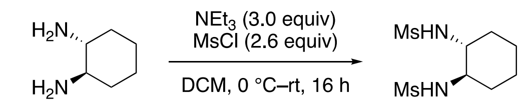


**Ligand ((–)-SI-2)** was synthesized following a procedure reported by Denmark.[1,iii] To a flame-dried round-bottom flask equipped with a stir bar was added (1*R*,2*R*)-cyclohexane-1,2-diamine (0.10 g, 0.91 mmol, 1.0 equiv), anhydrous DCM (9.1 mL, 0.10 M in substrate), then $NEt_3$ (0.38 mL, 2.7 mmol, 3.0 equiv). The reaction was cooled to 0 °C and allowed to stir for 5 min before adding MsCl (0.18 mL, 2.4 mmol, 2.6 equiv) dropwise via a syringe. The reaction was allowed to stir at 0 °C to rt for 16 h. To quench, 2 M $H_2SO_4$ (4 mL) was added at 0 °C, and the reaction mixture was extracted with DCM (3 x 20 mL). The combined organic layers were washed with brine, dried over $Na_2SO_4$, and concentrated in vacuo. The residue was then purified by flash column chromatography (25:1 DCM/MeOH) to afford the title compound as a white solid (0.17 g, 0.61 mmol, 67%). **TLC $R_f$** = 0.5 (20% EtOAc/hexanes); **$^1$H NMR** (400 MHz, $CDCl_3$) 4.72 (br s, 2H), 3.08 (br s, 2H), 3.04 (s, 6H), 2.19–2.17 (m, 2H), 1.79–1.77 (m, 2H), 1.38–1.31 (m, 4H); **$[\alpha]^{25}_D$** –7.8° (c 44.7 mg/1.5 mL MeOH). Analytical data is consistent with literature values.[1]

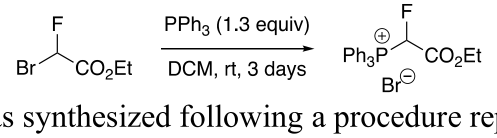


**Phosphonium salt (SI-4)** was synthesized following a procedure reported by Zeng.[iv] To a flame-dried round-bottom flask equipped with a stir bar was added triphenylphosphine (1.7 g, 6.5 mmol, 1.3 equiv), anhydrous DCM (3.8 mL, 1.3 M in substrate), then ethyl 2-bromo-2-fluoroacetate (0.59 mL, 5.0 mmol, 1.0 equiv). The reaction mixture was allowed to stir for 3 days. After 3 days, the

reaction mixture was concentrated before adding 7.5 mL $Et_2O$ and allowed to stir for 3 h, which led to white precipitate to form. The resulting mixture was filtered to afford the title compound as a white solid (1.4 g, 4.7 mmol, 93%). **$^1$H NMR** (400 MHz, $CDCl_3$) 9.71 (dd, $J$ = 41.5, 6.0 Hz, 1H), 8.01 (dd, $J$ = 13.2, 7.5 Hz, 6H), 7.83 (at, $J$ = 7.1 Hz, 3H), 7.73–7.68 (m, 6H), 4.14–4.08 (m, 2H), 1.01 (t, $J$ = 7.1 Hz, 3H). Analytical data is consistent with literature values.[4]

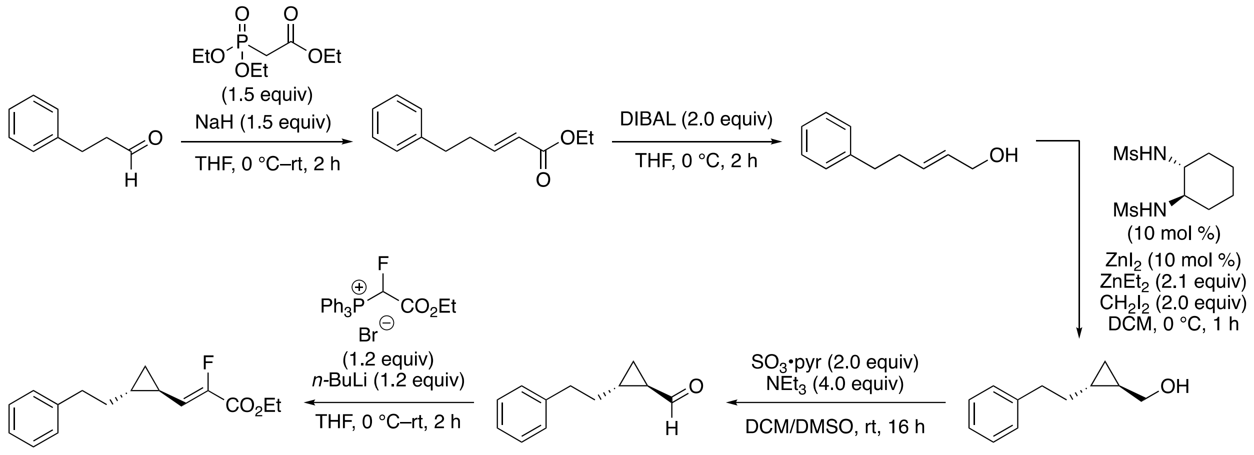


**Scheme SI-3:** Synthesis of Cyclopropane (–)-(***R***,***R***)-**3**

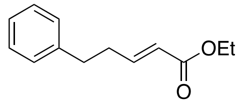


**Ester (SI-7)** was prepared according to a method reported by Deng.[v] In a glovebox, a flame-dried round-bottom flask equipped with a stir bar was charged with NaH (72 mg, 3.0 mmol, 1.5 equiv). The flask was sealed with a septum, removed from the glovebox, and placed under $N_2$. Anhydrous THF (10. mL, 0.20 M in substrate) and triethyl phosphonoacetate (0.60 mL, 3.0 mmol, 1.5 equiv) were added at 0 °C, and the reaction mixture was allowed to stir for 30 min. Hydrocinnamaldehyde (0.26 mL, 2.0 mmol, 1.0 equiv) was added at 0 °C and stirred for 15 min before warming to rt and allowed to stir for 75 min. To quench, saturated $NH_4Cl$ (aq) was added. The reaction mixture was extracted with $Et_2O$ (3 x 10 mL), and the combined organic layers were washed with brine, dried over $Na_2SO_4$, and concentrated in vacuo. The residue was purified by flash column chromatography (0–10% $Et_2O$/hexanes) to afford the title compound as a colorless oil (330 mg, 1.6 mmol, 81%). **TLC $R_f$** = 0.4 (10% EtOAc/hexanes); **$^1$H NMR** (400 MHz, $CDCl_3$) 7.31–7.25 (m, 2H), 7.22–7.17 (m, 3H), 7.00 (dt, $J$ = 15.5, 7.0 Hz, 1H), 5.84 (d, $J$ = 15.6 Hz, 1H), 4.18 (q, $J$ = 7.1 Hz, 2H), 2.78 (at, $J$ = 7.8 Hz, 2H), 2.52 (aq, $J$ = 7.4 Hz, 2H), 1.28 (t, $J$ = 7.1 Hz, 3H). Analytical data is consistent with literature values.[5]

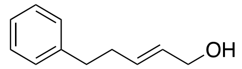


**Allylic alcohol (SI-1)** was prepared according to a method reported by Zakarian.[vi] To a flame-dried round-bottom flask equipped with a stir bar was added ester **SI-7** (0.21 g, 1.0 mmol, 1.0 equiv) in THF (2.8 mL, 0.20 M in substrate). The reaction mixture was cooled to 0 °C before adding DIBAL (1.3 mL, 1.2 mmol, 2.2 equiv, 1.0 M in hexanes) dropwise via a syringe. The reaction mixture was allowed to stir at 0 °C for 2 h. To quench, 2 N HCl (2 mL) was added. The reaction mixture was extracted with $Et_2O$ (3 x 10 mL), and the combined organic layers were

washed with brine, dried over $Na_2SO_4$, and concentrated in vacuo. The residue was purified by flash column chromatography (0–20% $Et_2O$/hexanes) to afford the title compound as a colorless oil (0.15 g, 0.94 mmol, 93%). **TLC $R_f$** = 0.2 (20% EtOAc/hexanes, $KMnO_4$ stain); **$^1$H NMR** (400 MHz, $CDCl_3$) 7.30–7.25 (m, 2H), 7.20–7.17 (m, 3H), 5.78–5.63 (m, 2H), 4.08 (ad, $J$ = 4.8 Hz, 2H), 2.71 (t, $J$ = 7.7 Hz, 2H), 2.40–2.35 (m, 2H), 1.22 (s, 1H). Analytical data is consistent with literature values.[6]

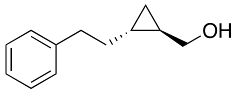

**Cyclopropane** (–)-($R$,$R$)-**SI-3** was prepared following a procedure reported by Denmark.[1] Two separate flasks, Flask A and B, were prepared according to the following procedures. **Flask A:** In a glovebox, a flame-dried round-bottom flask equipped with a stir bar was added $ZnEt_2$ (0.10 mL, 1.0 mmol, 1.0 equiv). The flask was sealed with a septum, removed from the glovebox, and placed under $N_2$. Anhydrous DCM (24 mL, 40. μM in substrate) was added before cooling the reaction mixture to 0 °C. After 5 min, diiodomethane (0.16 mL, 2.0 mmol, 2.0 equiv) was added, and the reaction mixture was allowed to stir for 5 min at 0 °C. **Flask B:** In a glovebox, a flame-dried round-bottom flask equipped with a stir bar, ligand (–)-**SI-2** (27 mg, 0.10 mmol, 0.10 equiv) and $ZnI_2$ (32 mg, 0.10 mmol, 0.10 equiv) was charged with $ZnEt_2$ (0.11 mL, 1.1 mmol, 1.1 equiv). The flask was sealed with a septum, removed from the glovebox, and placed under $N_2$. Anhydrous DCM (13 mL, 80. μM in substrate) was added before cooling the reaction mixture to 0 °C. After 5 min, alcohol **SI-1** (160 mg, 1.0 mmol, 1.0 equiv, 0.10 M in DCM) was added, and the reaction mixture was allowed to stir for 30 min at 0 °C. After 30 min, the contents were transferred via cannula to **Flask A** containing the cyclopropanating reagents prepared in advance. After cannula transfer, the reaction mixture was allowed to stir at 0 °C for an additional 30 min. To quench, 2 N NaOH (13 mL) was added. The reaction mixture was extracted with DCM (3 x 10 mL), and the combined organic layers were washed with brine, dried over $Na_2SO_4$, and concentrated in vacuo. The residue was purified by flash column chromatography (0–20% $Et_2O$/hexanes) to afford the title compound as a mixture of unreacted alcohol **SI-1** and cyclopropane product. To remove the unreacted alcohol **SI-1**, a dihydroxylation reaction was performed on the product mixture. To a round-bottom flask equipped with a stir bar was added the mixture of unreacted alcohol **SI-1** and cyclopropane product (assuming 0.10 mmol of unreacted alcohol **SI-1**, 1.0 equiv), AD-mix-$\beta$ (140 mg, 0.18 mmol, 1.8 equiv), $t$-BuOH (1.0 mL, 0.10 M in substrate), and $H_2O$ (1.0 mL, 0.10 M in substrate). The reaction was allowed to stir at 0 °C for 24 h. After 24 h, $Na_2SO_3$ (15 mg, 0.12 mmol, 1.2 equiv) was added, and the reaction mixture was allowed to stir at rt for 20 min. The reaction mixture was extracted with EtOAc (3 x 5 mL), dried over $Na_2SO_4$, and concentrated in vacuo. The compound was purified by flash column chromatography (0–20% $Et_2O$/hexanes) to afford the title compound as a clear, colorless oil (130 mg, 0.73 mmol, 73%). **TLC $R_f$** = 0.2 (20% EtOAc/hexanes, $KMnO_4$ stain); **$^1$H NMR** (400 MHz, $CDCl_3$) 7.30–7.18 (m, 5H), 3.45–3.34 (m, 2H), 2.76–2.65 (m, 2H), 1.68–1.48 (m, 2H), 1.15 (t, $J$ = 5.5 Hz, 1H), 0.87–0.79 (m, 1H), 0.65–0.58 (m, 1H), 0.40–0.31 (m, 2H); **$[\alpha]^{25}_D$** –16.8° (c 8.3 mg/1.5 mL $CHCl_3$); **SFC Analysis** (Chiralcel AD, 3% IPA/$CO_2$, 2.0 mL/min, 210 nm) indicated 65% ee: $t_R$ (major enantiomer) = 13.0 minutes, $t_R$ (minor enantiomer) = 15.1 minutes. Analytical data is consistent with literature values.[1]

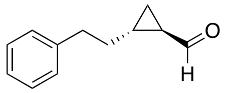


**Aldehyde** (–)-**(SI-8)** To a flame-dried round-bottom flask equipped with a stir bar was added $SO_3$•pyridine (0.23 g, 1.5 mmol, 2.0 equiv), cyclopropane (–)-(*R*,*R*)-**SI-3** (130 mg, 0.73 mmol, 1.0 equiv), $NEt_3$ (0.4 mL, 2.9 mmol, 4.0 equiv), anhydrous DCM (1.5 mL, 0.50 M in substrate), and DMSO (1.5 mL, 0.50 M in substrate). The reaction mixture was allowed to stir at rt for 16 h. To quench, saturated $NH_4Cl$ (aq) was added. The reaction mixture was extracted with DCM (3 x 10 mL), and the combined organic layers were washed with brine, dried over $Na_2SO_4$, and concentrated in vacuo. The residue was purified by flash column chromatography (0–20% $Et_2O$/hexanes) to afford the title compound as a pale-yellow oil (110 mg, 0.62 mmol, 84%). **TLC** $\mathbf{R_f}$ = 0.5 (20% EtOAc/hexanes, CAM stain); $^1$**H NMR** (400 MHz, $CDCl_3$) 8.98 (d, *J* = 5.4 Hz, 1H), 7.29–7.15 (m, 5H), 2.72 (t, *J* = 7.5 Hz, 2H), 1.75–1.58 (m, 3H), 1.49–1.43 (m, 1H), 1.30–1.26 (m, 1H), 0.93–0.88 (m, 1H); $[\alpha]^{25}_D$ –28.3° (c 8.3 mg/1.5 mL $CHCl_3$). Analytical data is consistent with literature values.[vii]

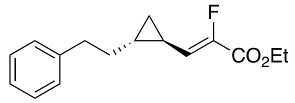


**Cyclopropane** (–)-**(*R*,*R*)-3** was prepared following a procedure reported by Wu.[viii] To a flame-dried round-bottom flask equipped with a stir bar was added phosphonium salt **SI-4** (0.19 g, 0.66 mmol, 1.2 equiv) and anhydrous THF (2.7 mL, 0.20 M in substrate). The reaction mixture was cooled to 0 °C before adding *n*-BuLi (0.26 mL, 0.66 mmol, 1.2 equiv, 2.5 M in hexanes). The reaction mixture was allowed to stir for 1 h at 0 °C before adding aldehyde (–)-**SI-8** (95 mg, 0.55 mmol, 1.0 equiv, 0.10 M in THF) dropwise via syringe. The reaction mixture was allowed to warm to rt and stir for an additional 1 h. To quench, saturated $NH_4Cl$ (aq) was added. The reaction mixture was extracted with EtOAc (3 x 10 mL) and the combined organic layers were washed with brine, dried over $Na_2SO_4$, and concentrated in vacuo. The residue was purified by flash column chromatography (0–20% EtOAc/hexanes) to afford the title compound as a pale-yellow oil (16 mg, 61 μmol, 11%). **TLC** $\mathbf{R_f}$ = 0.5 (10% EtOAc/hexanes); $^1$**H NMR** (400 MHz, $CDCl_3$) 7.29–7.25 (m, 2H), 7.20–7.16 (m, 3H), 5.59 (dd, *J* = 31.9, 10.7 Hz, 1H), 4.26 (q, *J* = 7.1 Hz, 2H), 2.71 (t, *J* = 7.6 Hz, 2H), 1.68–1.56 (m, 3H), 1.31 (t, *J* = 7.1 Hz, 3H), 1.05–0.97 (m, 1H), 0.83–0.78 (m, 2H); **HRMS** (TOF MS ES+) *m/z*: $[M + Na]^+$ calculated for $C_{16}H_{19}FO_2Na$, 285.1267; found 285.1258; $[\alpha]^{25}_D$ –38.1°; **SFC Analysis** (Chiralcel OD-H, 1% IPA/$CO_2$, 2.0 mL/min, 230 nm) indicated 65% ee: $t_R$ (major enantiomer) = 11.0 minutes, $t_R$ (minor enantiomer) = 13.3 minutes.

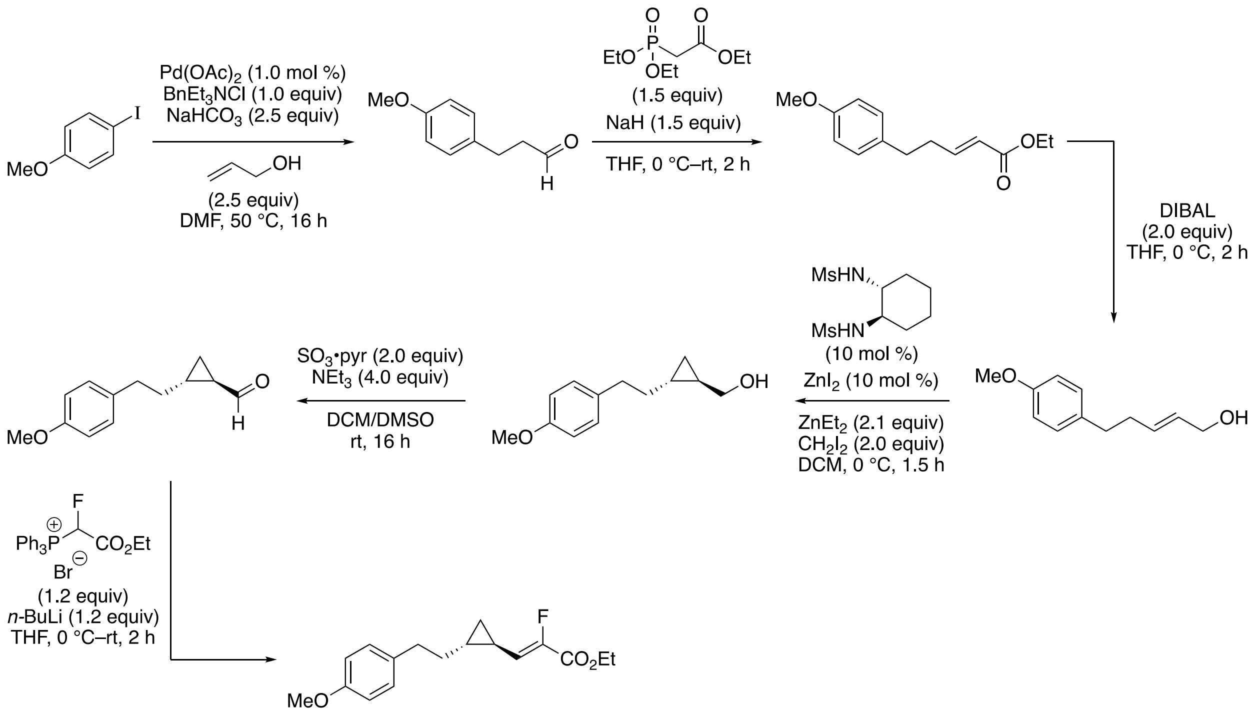

**Scheme SI-4:** Synthesis of Cyclopropane (–)-(***R***,***R***)-4

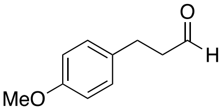

**Aldehyde (SI-9)** was prepared according to a procedure reported by Fürstner.[ix] To a flame-dried pressure tube equipped with a stir bar was added 1-iodo-4-methoxybenzene (0.47 g, 2.0 mmol, 1.0 equiv), benzyltriethylammonium chloride (0.46 g, 2.0 mmol, 1.0 equiv), and $NaHCO_3$ (0.42 g, 5.0 mmol, 2.5 equiv). The tube was sealed and pumped into the glovebox where $Pd(OAc)_2$ (4.5 mg, 20 μmol, 1.0 mol %) was added. After sealing the tube with a septum, the tube was brought out of the glovebox and added DMF (10. mL, 0.20 M in substrate) then allyl alcohol (0.34 mL, 5.0 mmol, 1.5 equiv). The tube was sealed with a teflon cap, heated to 50 °C behind a blast shield, and allowed to stir for 16 h. After 16 h, the reaction mixture was cooled to rt before filtering over celite, flushing with EtOAc. The organic layer was washed with $H_2O$ (3 x 20 mL), washed with brine, dried over $Na_2SO_4$, and concentrated in vacuo. The residue was purified by flash column chromatography (0–10% EtOAc/hexanes) to afford the title compound as a clear, colorless oil (0.25 g, 1.5 mmol, 75%). **TLC R$_f$** = 0.3 (10% EtOAc/hexanes, CAM stain); **$^1$H NMR** (400 MHz, $CDCl_3$) δ 9.81 (ap s, 1H), 7.11 (d, *J* = 8.7 Hz, 2H), 6.83 (d, *J* = 8.7 Hz, 2H), 3.78 (s, 3H), 2.91 (t, *J* = 7.5 Hz, 2H), 2.74 (t, *J* = 7.1 Hz, 2H). Analytical data is consistent with literature values.[9]

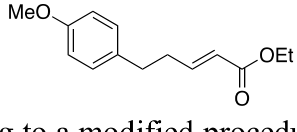

**Ester** (**SI-10)** was prepared according to a modified procedure reported by Deng.[5] In a glovebox, a flame-dried round-bottom flask equipped with a stir bar was charged with NaH (54 mg, 2.2 mmol, 1.5 equiv). The flask was sealed with a septum, removed from the glovebox, and placed under $N_2$. Anhydrous THF (7.5 mL, 0.20 M in substrate) and triethyl phosphonoacetate (0.45 mL, 2.2 mmol,

1.5 equiv) were added at 0 °C, and the reaction mixture was allowed to stir for 30 min. Aldehyde **SI-9** (0.24 g, 1.5 mmol, 1.0 equiv) was added at 0 °C and stirred for 15 min before warming to rt and stirred for 75 min. To quench, saturated $NH_4Cl$ (aq) was added. The reaction mixture was extracted with $Et_2O$ (3 x 10 mL), and the combined organic layers were washed with brine, dried over $Na_2SO_4$, and concentrated in vacuo. The residue was purified by flash column chromatography (0–10% $Et_2O$/hexanes) to afford the title compound as a colorless oil (0.32 g, 1.4 mmol, 91%). **TLC R$_f$** = 0.5 (20% EtOAc/hexanes); **$^1$H NMR** (400 MHz, $CDCl_3$) 7.08 (d, $J$ = 8.6 Hz, 2H), 6.98 (dt, $J$ = 6.9 Hz, 1H), 6.83 (d, $J$ = 8.6 Hz, 2H), 5.83 (d, $J$ = 15.7 Hz, 1H), 4.17 (q, $J$ = 7.1 Hz, 2H), 3.78 (s, 3H), 2.71 (t, $J$ = 7.7 Hz, 2H), 2.50–2.45 (m, 2H), 1.27 (t, $J$ = 7.1 Hz, 3H). Analytical data is consistent with literature values.[x]

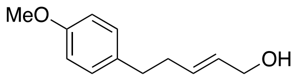


**Allylic alcohol (SI-5)** was prepared according to a modified procedure reported by Zakarian.[6] To a flame-dried round-bottom flask equipped with a stir bar was added ester **SI-10** (0.32 g, 1.4 mmol, 1.0 equiv) in THF (6.8 mL, 0.20 M in substrate). The reaction mixture was cooled to 0 °C before adding DIBAL (2.7 mL, 2.7 mmol, 2.0 equiv, 1.0 M in hexanes) dropwise via a syringe. The reaction mixture was allowed to stir at 0 °C for 2 h. To quench, 2 N HCl (4 mL) was added. The reaction mixture was extracted with $Et_2O$ (3 x 10 mL), and the combined organic layers were washed with brine, dried over $Na_2SO_4$, and concentrated in vacuo. The residue was purified by flash column chromatography (0–20% $Et_2O$/hexanes) to afford the title compound as a colorless oil (0.23 g, 1.2 mmol, 90%). **TLC R$_f$** = 0.4 (30% EtOAc/hexanes, $KMnO_4$ stain); **$^1$H NMR** (400 MHz, $CDCl_3$) 7.08 (d, $J$ = 8.5 Hz, 2H), 6.82 (d, $J$ = 8.6 Hz, 2H), 5.75–5.61 (m, 2H), 4.07 (br s, 2H), 3.78 (s, 3H), 2.64 (t, $J$ = 7.7 Hz, 2H), 2.36–2.32 (m, 2H), 1.48 (br s, 1H). Analytical data is consistent with literature values.[xi]

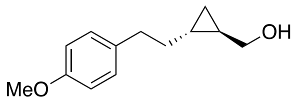


**Cyclopropane** (–)-($R$,$R$)-**SI-6** was prepared following a procedure reported by Denmark.[1] Two separate flasks, Flask A and B, were prepared according to the following procedures. **Flask A:** In a glovebox, a flame-dried round-bottom flask equipped with a stir bar was added $ZnEt_2$ (0.10 mL, 1.0 mmol, 1.0 equiv). The flask was sealed with a septum, removed from the glovebox, and placed under $N_2$. Anhydrous DCM (25 mL, 40. μM in substrate) was added before cooling the reaction mixture to 0 °C. After 5 min, diiodomethane (0.16 mL, 2.0 mmol, 2.0 equiv) was added, and the reaction mixture was allowed to stir for 5 min at 0 °C. **Flask B:** In a glovebox, a flame-dried round-bottom flask equipped with a stir bar, ligand (–)-**SI-2** (27 mg, 0.10 mmol, 0.10 equiv) and $ZnI_2$ (32 mg, 0.10 mmol, 0.10 equiv) was charged with $ZnEt_2$ (0.11 mL, 1.1 mmol, 1.1 equiv). The flask was sealed with a septum, removed from the glovebox, and placed under $N_2$. Anhydrous DCM (13 mL, 80. μM in substrate) was added before cooling the reaction mixture to 0 °C. After 5 min, allylic alcohol **SI-5** (0.20 g, 1.0 mmol, 1.0 equiv, 0.10 M in DCM) was added, and the reaction mixture was allowed to stir for 30 min at 0 °C. After 30 min, the contents were transferred via cannula to **Flask A** containing the cyclopropanating reagents prepared in advance. After cannula transfer, the reaction mixture was allowed to stir at 0 °C for an additional 30 min. To quench, 2 N NaOH (13 mL) was added. The reaction mixture was extracted with DCM (3 x 10 mL), and the combined organic layers were washed with brine, dried over $Na_2SO_4$, and concentrated in vacuo.

The residue was purified by flash column chromatography (0–20% $Et_2O$/hexanes) to afford the title compound as a mixture of unreacted alcohol **SI-5** and cyclopropane product. To remove the unreacted alcohol **SI-5**, a dihydroxylation reaction was performed on the product mixture. To a round-bottom flask equipped with a stir bar was added the mixture of unreacted alcohol **SI-5** and cyclopropane product (assuming 0.10 mmol of unreacted alcohol **SI-5**, 1.0 equiv), AD-mix-*β* (140 mg, 0.18 mmol, 1.8 equiv), *t*-BuOH (1.0 mL, 0.10 M in substrate), and $H_2O$ (1.0 mL, 0.10 M in substrate). The reaction was allowed to stir at 0 °C for 24 h. After 24 h, $Na_2SO_3$ (15 mg, 0.12 mmol, 1.2 equiv) was added, and the reaction mixture was allowed to stir at rt for 20 min. The reaction mixture was extracted with EtOAc (3 x 5 mL), dried over $Na_2SO_4$, and concentrated in vacuo. The compound was purified by flash column chromatography (0–20% $Et_2O$/hexanes) to afford the title compound as a clear, colorless oil (0.13 g, 0.61 mmol, 61%). **TLC $R_f$** = 0.3 (30% EtOAc/hexanes, $KMnO_4$ stain); **$^1$H NMR** (400 MHz, $CDCl_3$) 7.10 (d, *J* = 8.5 Hz, 2H), 6.82 (d, *J* = 8.6 Hz, 2H), 3.78 (s, 3H), 3.42–3.35 (m, 2H), 2.67–2.63 (m, 2H), 1.64–1.46 (m, 2H), 1.25 (br s, 1H), 0.88–0.79 (m, 1H), 0.65–0.57 (m, 1H), 0.39–0.30 (m, 2H); **$^{13}$C NMR** (125.7 MHz, $CDCl_3$) δ 157.9, 134.4, 129.4 (2C), 113.9 (2C), 67.2, 55.4, 35.7, 35.1, 21.5, 16.9, 10.0; **HRMS** (TOF MS ES+) *m/z*: $[M + Na]^+$ calculated for $C_{13}H_{18}O_2Na$, 229.1205; found 229.1210; **$[\alpha]^{25}_D$** –11.2° (c 8.3 mg/1.5 mL $CHCl_3$); **SFC Analysis** (Chiralcel AD, 3% IPA/$CO_2$, 2.0 mL/min, 230 nm) indicated 47% ee: $t_R$ (major enantiomer) = 27.1 minutes, $t_R$ (minor enantiomer) = 31.4 minutes.

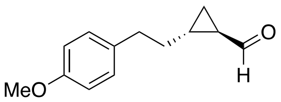


**Aldehyde ((–)-SI-11)** To a flame-dried round-bottom flask equipped with a stir bar was added $SO_3$•pyridine (0.18 g, 1.1 mmol, 2.0 equiv), cyclopropane (–)-(*R*,*R*)-**SI-6** (0.12 g, 0.56 mmol, 1.0 equiv), $NEt_3$ (0.31 mL, 2.3 mmol, 4.0 equiv), anhydrous DCM (1.1 mL, 0.50 M in substrate), and DMSO (1.1 mL, 0.50 M in substrate). The reaction mixture was allowed to stir at rt for 16 h. To quench, saturated $NH_4Cl$ was added. The reaction mixture was extracted with DCM (3 x 10 mL), and the combined organic layers were washed with brine, dried over $Na_2SO_4$, and concentrated in vacuo. The residue was purified by flash column chromatography (0–30% $Et_2O$/hexanes) to afford the title compound as a pale-yellow oil (92 mg, 0.45 mmol, 80%). **TLC $R_f$** = 0.5 (20% EtOAc/hexanes, $KMnO_4$ stain); **$^1$H NMR** (500 MHz, $CDCl_3$) 8.98 (d, *J* = 5.5 Hz, 1H), 7.07 (d, *J* = 8.5 Hz, 2H), 6.83 (d, *J* = 8.5 Hz, 2H), 3.79 (s, 3H), 2.67 (t, *J* = 7.5 Hz, 2H), 1.69–1.58 (m, 3H), 1.48–1.43 (m, 1H), 1.30–1.26 (m, 1H), 0.93–0.89 (m, 1H); **$^{13}$C NMR** (125.7 MHz, $CDCl_3$) δ 201.0, 133.5, 129.5 (2C), 123.2, 114.0 (2C), 55.4, 34.8, 34.6, 30.6, 22.4, 15.0; **HRMS** (TOF MS CI+) *m/z*: $[M]^+$ calculated for $C_{13}H_{16}O_2$, 204.1150; found 204.1150; **$[\alpha]^{25}_D$** –17.2° (c 8.3 mg/1.5 mL $CHCl_3$).

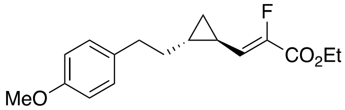


**Cyclopropane** (–)-**(*R,R*)-4** was prepared following a procedure reported by Wu.[8] To a flame-dried round-bottom flask equipped with a stir bar was added phosphonium salt **SI-4** (0.15 g, 0.53 mmol, 1.5 equiv) and anhydrous THF (1.8 mL, 0.20 M in substrate). The reaction mixture was cooled to 0 °C before adding *n*-BuLi (0.21 mL, 0.53 mmol, 1.5 equiv, 2.5 M in hexanes). The reaction mixture was allowed to stir for 1 h at 0 °C before adding aldehyde (–)-**SI-11** (72 mg, 0.35 mmol, 1.0 equiv, 0.10 M in THF) dropwise via syringe. The reaction mixture was allowed to warm to rt and stir for 16 h. To quench, saturated $NH_4Cl$ (aq) was added. The reaction mixture was extracted

with EtOAc (3 x 10 mL), and the combined organic layers were washed with brine, dried over $Na_2SO_4$, and concentrated in vacuo. The residue was purified by flash column chromatography (0–10% EtOAc/hexanes) to afford the title compound as a pale-yellow oil (26 mg, 90. $\mu$mol, 26%). **TLC R$_f$** = 0.4 (10% EtOAc/hexanes); **$^1$H NMR** (500 MHz, $CDCl_3$) 7.08 (d, *J* = 8.4 Hz, 2H), 6.82 (d, *J* = 8.3 Hz, 2H), 5.59 (dd, *J* = 32.0, 10.7 Hz, 1H), 4.26 (q, *J* = 7.1 Hz, 2H), 3.79 (s, 3H), 2.66 (t, *J* = 7.5 Hz, 2H), 1.67–1.57 (m, 3H), 1.31 (t, *J* = 7.1 Hz, 3H), 1.02–0.97 (m, 1H), 0.82–0.77 (m, 2H); **HRMS** (TOF MS ES+) *m/z*: $[M + Na]^+$ calculated for $C_{17}H_{21}FO_3Na$, 315.1372; found 315.1371; **$[\alpha]^{25}_D$** –25.0º (c 10.8 mg/1.5 mL $CHCl_3$); **SFC Analysis** (Chiralcel OD-H, 0.1% IPA/$CO_2$, 2.0 mL/min, 230 nm) indicated 47% ee: $t_R$ (major enantiomer) = 63.9 minutes, $t_R$ (minor enantiomer) = 80.7 minutes.

## D. Characterization Data for Cyclopropanes 2–16:

1) Racemic Cyclopropanes

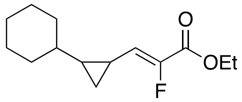


**Cyclopropane (2a)** was prepared according to Method A. The following amounts of reagents were used: mesylate **1a** (69 mg, 0.19 mmol, 1.0 equiv), $Ni(PMe_3)_2Cl_2$ (2.7 mg, 9.7 µmol, 5.0 mol %), $Zn^0$ (25 mg, 0.39 mmol, 2.0 equiv), NaBr (40. mg, 0.39 mmol, 2.0 equiv), and anhydrous MeCN (1.9 mL, 0.10 M in substrate). Before purification, a $^1$H NMR yield of 62% was obtained based on comparison to PhTMS as internal standard. The residue was then purified by flash column chromatography (0–10% EtOAc/hexanes) to afford the title compound as a mixture of cyclopropane diastereomers as a clear, colorless oil (28 mg, 0.12 mmol, 60%, 3.5:1 dr trans:cis). The dr was determined by integration of the alkene proton resonances of each cyclopropane diastereomer in the $^1$H NMR spectrum. The relative configuration of cyclopropane was assigned based on analogy to cyclopropane **14**. **TLC R$_f$** = 0.6 (10% EtOAc/hexanes); **HRMS** (TOF MS ES+) *m/z*: $[M + Na]^+$ calculated for $C_{14}H_{21}FO_2Na$, 263.1423; found 263.1425. For clarity, the $^1$H NMR, $^{13}$C NMR, and $^{19}$F NMR data of the major and minor diastereomers have been tabulated individually.

**Major diastereomer: $^1$H NMR** (500 MHz, $CDCl_3$) $\delta$ 5.62 (dd, *J* = 32.0, 10.7 Hz, 1H), 4.25 (q, *J* = 7.1 Hz, 2H), 1.90–1.56 (m, 6H), 1.31 (t, *J* = 7.5 Hz, 3H), 1.27–1.00 (m, 6H), 0.85–0.81 (m, 1H), 0.77–0.66 (m, 2H); **$^{13}$C NMR** (125.7 MHz, $CDCl_3$) δ 161.1 (d, *J* = 33.8 Hz), 147.5 (d, *J* = 251.3 Hz), 126.3 (d, *J* = 10.7 Hz), 61.4, 42.4, 32.8, 32.7, 29.3, 26.6, 26.29, 26.2, 14.5, 14.3, 14.2; **$^{19}$F NMR** (376.5 MHz, $CDCl_3$) δ -137.8 (d, *J* = 32.0 Hz, 1F).

**Minor diastereomer: $^1$H NMR** (500 MHz, $CDCl_3$) $\delta$ 5.86 (dd, *J* = 31.7, 11.0 Hz, 1H), 4.25 (q, *J* = 7.1 Hz, 2H), 1.90–1.56 (m, 7H), 1.31 (t, *J* = 7.5 Hz, 3H), 1.27–1.00 (m, 6H), 0.85–0.81 (m, 1H), 0.45 (aq, *J* = 5.3 Hz, 1H); **$^{13}$C NMR** (125.7 MHz, $CDCl_3$) δ 161.0 (d, *J* = 33.7 Hz), 148.9 (d, *J* = 253.5 Hz), 123.2 (d, *J* = 9.9 Hz), 61.4, 39.1, 33.4, 33.1, 27.7, 26.5, 26.3, 26.1, 14.6, 14.3, 14.2; **$^{19}$F NMR** (376.5 MHz, $CDCl_3$) δ -136.1 (d, *J* = 31.8 Hz, 1F).

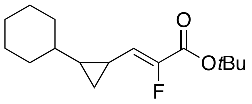


**Cyclopropane (2b)** was prepared according to Method A. The following amounts of reagents were used: mesylate **1b** (32 mg, 85 µmol, 1.0 equiv), $Ni(PMe_3)_2Cl_2$ (1.2 mg, 4.3 µmol, 5.0 mol %), $Zn^0$ (11 mg, 0.17 mmol, 2.0 equiv), NaBr (18 mg, 0.17 mmol, 2.0 equiv), and anhydrous MeCN (1.0 mL, 0.085 M in substrate). Before purification, a $^1$H NMR yield of 50% was obtained based on comparison to PhTMS as internal standard. The residue was then purified by flash column chromatography (0–10% EtOAc/hexanes) to afford the title compound as a mixture of cyclopropane diastereomers as a clear colorless oil (6.0 mg, 22 µmol, 26%, 1.8:1 dr trans:cis). The dr was determined by integration of the alkene proton resonances of each cyclopropane diastereomer in the $^1$H NMR spectrum. The relative configuration of cyclopropane was assigned based on analogy to cyclopropane **14**. **TLC R$_f$** = 0.6 (10% EtOAc/hexanes); **HRMS** (TOF MS ES+) *m/z*: $[M + H]^+$ calculated for $C_{16}H_{25}FO_2H$, 269.1917; found 269.1920. For clarity, the $^1$H NMR, $^{13}$C NMR, and $^{19}$F NMR data of the major and minor diastereomers have been tabulated individually.

**Major diastereomer: $^1$H NMR** (400 MHz, $CDCl_3$) $\delta$ 5.51 (dd, $J$ = 32.0, 10.7 Hz, 1H), 1.88–1.60 (m, 5H), 1.51 (s, 9H), 1.31–1.14 (m, 3H), 1.14–0.77 (m, 5H), 0.76–0.63 (m, 2H); **$^{13}$C NMR** (150.9 MHz, $CDCl_3$) δ 160.14 (d, $J$ = 33.7 Hz), 148.1 (d, $J$ = 252.1 Hz), 125.0 (d, $J$ = 11.6 Hz), 82.0, 42.4, 33.0, 32.7, 28.1 (3C), 26.5, 26.2 (2C), 14.2, 14.0, 12.3; **$^{19}$F NMR** (564.6 MHz, $CDCl_3$) δ -136.4 (d, $J$ = 32.0 Hz, 1F).

**Minor diastereomer: $^1$H NMR** (400 MHz, $CDCl_3$) $\delta$ 5.76 (dd, $J$ = 31.7, 11.0 Hz, 1H), 1.88–1.60 (m, 5H), 1.53 (s, 9H), 1.31–1.14 (m, 3H), 1.14–0.77 (m, 6H), 0.46–0.38 (m, 1H); **$^{13}$C NMR** (150.9 MHz, $CDCl_3$) δ 160.1 (d, $J$ = 32.6 Hz), 149.5 (d, $J$ = 255.4 Hz), 121.8 (d, $J$ = 10.5 Hz), 82.0, 42.4, 33.3, 32.6, 28.1 (3C), 26.5, 26.3, 26.1, 14.2, 14.0, 12.3; **$^{19}$F NMR** (564.6 MHz, $CDCl_3$) δ -134.7 (d, $J$ = 31.8 Hz, 1F).

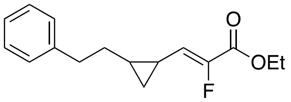


**Cyclopropane (3)** was prepared according to Method A. The following amounts of reagents were used: mesylate **SI-12** (39 mg, 0.10 mmol, 1.0 equiv), $Ni(PMe_3)_2Cl_2$ (1.4 mg, 5.0 µmol, 5.0 mol %), $Zn^0$ (13 mg, 0.21 mmol, 2.0 equiv), NaBr (21 mg, 0.21 mmol, 2.0 equiv), and anhydrous MeCN (1.0 mL, 0.10 M in substrate). Before purification, a $^1$H NMR yield of 65% was obtained based on comparison to PhTMS as internal standard. The residue was then purified by flash column chromatography (0–20% $Et_2O$/pentanes) to afford the title compound as a mixture of cyclopropane diastereomers as a pale-yellow oil (16 mg, 61 µmol, 59%, 1.2:1 dr trans:cis). The dr was determined by integration of the alkene proton resonances of each cyclopropane diastereomer in the $^1$H NMR spectrum. The relative configuration of cyclopropane was assigned based on analogy to cyclopropane **14**. **TLC R$_f$** = 0.5 (5% EtOAc/hexanes); **HRMS** (TOF MS ES+) *m/z*: $[M + Na]^+$ calculated for $C_{16}H_{19}FO_2Na$, 285.1267; found 285.1266; **SFC Analysis** (Chiralcel OD-H, 1% IPA/$CO_2$, 2.0 mL/min, 230 nm) indicated 0% ee: $t_R$ (minor diastereomer, both enantiomers) = 9.8 minutes, $t_R$ (major diastereomer, minor enantiomer) = 11.0 minutes, $t_R$ (major diastereomer, major

enantiomer) = 13.3 minutes. For clarity, the $^1$H NMR, $^{13}$C NMR, and $^{19}$F NMR data of the major and minor diastereomers have been tabulated individually.

**Major diastereomer:** **$^1$H NMR** (400 MHz, $CDCl_3$) δ 7.30–7.25 (m, 2H), 7.21–7.12 (m, 3H), 5.59 (dd, *J* = 31.9, 10.7 Hz, 1H), 4.30–4.21 (m, 2H), 2.74–2.66 (m, 2H), 1.80–1.61 (m, 2H), 1.60–1.52 (m, 1H), 1.33–1.31 (m, 3H), 1.05–0.96 (m, 1H), 0.83–0.76 (m, 2H); **$^{13}$C NMR** (125.7 MHz, $CDCl_3$) δ 160.9 (d, *J* = 33.8 Hz), 147.7 (d, *J* = 252.5 Hz), 141.8, 128.60, 128.56, 128.5 (2C), 125.9, 125.7 (d, *J* = 10.6 Hz), 61.4, 35.6, 32.0, 22.3, 15.7, 15.5, 14.4; **$^{19}$F NMR** (564.6 MHz, $CDCl_3$) δ -136.9 (d, *J* = 31.3 Hz, 1F).

**Minor diastereomer:** **$^1$H NMR** (400 MHz, $CDCl_3$) δ 7.30–7.25 (m, 2H), 7.21–7.12 (m, 3H), 5.82 (dd, *J* = 31.7, 11.0 Hz, 1H), 4.30–4.21 (m, 2H), 2.74–2.66 (m, 2H), 1.93–1.83 (m, 1H), 1.80–1.61 (m, 2H), 1.33–1.31 (m, 4H), 1.15 (td, *J* = 8.6, 4.8 Hz, 1H), 0.45 (q, *J* = 5.4 Hz, 1H); **$^{13}$C NMR** (125.7 MHz, $CDCl_3$) δ 160.7 (d, *J* = 33.8 Hz), 148.9 (d, *J* = 253.4 Hz), 141.8, 128.60, 128.56, 128.5 (2C), 125.9, 122.4 (d, *J* = 9.7 Hz), 61.5, 35.9, 35.54, 20.4, 15.6, 15.5, 12.8; **$^{19}$F NMR** (564.6 MHz, $CDCl_3$) δ -135.2 (d, *J* = 31.3 Hz, 1F).

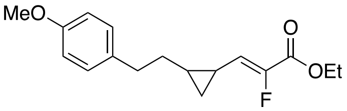


**Cyclopropane (4)** was prepared according to Method A. The following amounts of reagents were used: mesylate **SI-13** (44 mg, 0.11 mmol, 1.0 equiv), $Ni(PMe_3)_2Cl_2$ (1.5 mg, 5.4 μmol, 5.0 mol %), $Zn^0$ (14 mg, 0.22 mmol, 2.0 equiv), NaBr (22 mg, 0.22 mmol, 2.0 equiv), and anhydrous MeCN (1.1 mL, 0.10 M in substrate). Before purification, a $^1$H NMR yield of 71% was obtained based on comparison to PhTMS as internal standard. The residue was then purified by flash column chromatography (0–10% EtOAc/hexanes) to afford the title compound as a mixture of cyclopropane diastereomers as a pale-yellow oil (24 mg, 82 μmol, 76%, 1.2:1 dr). The dr was determined by integration of the alkene proton resonances of each cyclopropane diastereomer in the $^1$H NMR spectrum. The relative configuration of cyclopropane was assigned based on analogy to cyclopropane **14**. **TLC $R_f$** = 0.5 (10% EtOAc/hexanes); **HRMS** (TOF MS CI+) *m/z*: $[M + H]^+$ calculated for $C_{17}H_{21}FO_3H$, 293.1553; found 293.1568; **SFC Analysis** (Chiralcel OD-H, 0.1% IPA/$CO_2$, 2.0 mL/min, 230 nm) indicated 1% ee: $t_R$ (minor diastereomer, both enantiomers) = 44.5 minutes, $t_R$ (major diastereomer, major enantiomer) = 49.9 minutes, $t_R$ (major diastereomer, minor enantiomer) = 62.3 minutes. For clarity, the $^1$H NMR, $^{13}$C NMR, and $^{19}$F NMR data of the major and minor diastereomers have been tabulated individually.

**Major diastereomer:** **$^1$H NMR** (400 MHz, $CDCl_3$) *δ* 7.07 (d, *J* = 8.5 Hz, 2H), 6.81 (d, *J* = 8.4 Hz, 2H), 5.59 (dd, *J* = 31.9, 10.8 Hz, 1H), 4.25 (q, *J* = 7.1 Hz, 2H), 3.77 (s, 3H), 2.65 (t, *J* = 7.4 Hz, 2H), 1.76–1.52 (m, 3H), 1.30 (t, *J* = 6.4 Hz, 3H), 1.02–0.97 (m, 1H), 0.80–0.77 (m, 2H); **$^{13}$C NMR** (100 MHz, $CDCl_3$) δ 160.9 (d, *J* = 33.9 Hz), 157.9, 147.7 (d, *J* = 251.9 Hz), 133.91, 129.6 (2C), 125.7 (d, *J* = 10.6 Hz), 113.9 (2C), 61.3, 55.3, 35.7, 32.2, 22.3, 15.6, 15.5, 14.3; **$^{19}$F NMR** (376.5 MHz, $CDCl_3$) δ -137.2 (d, *J* = 31.9 Hz, 1F).

**Minor diastereomer:** **$^1$H NMR** (400 MHz, $CDCl_3$) *δ* 7.08 (d, *J* = 9.1 Hz, 2H), 6.81 (d, *J* = 8.4 Hz, 2H), 5.81 (dd, *J* = 31.6, 10.9 Hz, 1H), 4.26 (q, *J* = 6.7 Hz, 2H), 3.77 (s, 3H), 2.65 (t, *J* = 7.4 Hz, 2H), 1.90–1.82 (m, 1H), 1.76–1.52 (m, 2H), 1.32 (t, *J* = 6.3 Hz, 3H), 1.28–1.22 (m, 1H), 1.16–

1.11 (m, 1H), 0.44–0.43 (m, 1H); **$^{13}$C NMR** (100 MHz, $CDCl_3$) δ 160.7 (d, *J* = 33.9 Hz), 157.9, 148.9 (d, *J* = 253.5 Hz), 133.95, 129.6 (2C), 122.4 (d, *J* = 10.0 Hz), 113.9 (2C), 61.4, 55.3, 34.9, 34.6, 20.4, 15.4, 14.3, 12.7; **$^{19}$F NMR** (376.5 MHz, $CDCl_3$) δ -135.6 (d, *J* = 31.7 Hz, 1F).

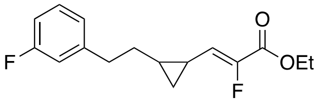


**Cyclopropane (5)** was prepared according to Method A. The following amounts of reagents were used: mesylate **SI-14** (30 mg, 76 µmol, 1.0 equiv), $Ni(PMe_3)_2Cl_2$ (1.1 mg, 3.8 µmol, 5.0 mol %), $Zn^0$ (10. mg, 0.15 mmol, 2.0 equiv), NaBr (16 mg, 0.15 mmol, 2.0 equiv), and MeCN (1.0 mL, 0.076 M in substrate). Before purification, a $^1$H NMR yield of 51% was obtained based on comparison to PhTMS as internal standard. The compound was purified by flash column chromatography (0–10% EtOAc/hexanes) to afford the title compound as a mixture of cyclopropane diastereomers as a clear, colorless oil (12 mg, 43 µmol, 57%, 1.2:1 dr). The dr was determined by integration of the alkene proton resonances of each cyclopropane diastereomer in the $^1$H NMR spectrum. The relative configuration of cyclopropane was assigned based on analogy to cyclopropane **14**. **TLC $R_f$** = 0.5 (10% EtOAc/hexanes); **HRMS** (TOF MS CI+) *m/z*: $[M+H]^+$ calculated for $C_{16}H_{18}F_2O_2H$, 281.1353; found 281.1357. For clarity, the $^1$H NMR, $^{13}$C NMR, and $^{19}$F NMR data of the major and minor diastereomers have been tabulated individually.

**Major Diastereomer: $^1$H NMR** (500 MHz, $CDCl_3$) δ 7.32–7.25 (m, 1H), 7.03–6.98 (m, 1H), 6.97–6.90 (m, 2H), 5.86 (dd, *J* = 31.5, 10.9 Hz, 1H), 4.32 (q, *J* = 7.1 Hz, 2H), 2.81–2.72 (m, 2H), 1.77–1.66 (m, 2H), 1.38 (t, *J* = 7.1 Hz, 3H), 1.25–1.19 (m, 1H), 1.10–1.02 (m, 1H), 0.86 (t, *J* = 6.8 Hz, 2H); **$^{13}$C NMR** (125.7 MHz, $CDCl_3$) δ 162.9 (d, *J* = 245.1 Hz), 160.9 (d, *J* = 33.8 Hz), 147.9 (d, *J* = 252.5 Hz), 144.4 (d, *J* = 4.6 Hz), 129.8, 125.5 (d, *J* = 10.6 Hz), 124.1 (d, *J* = 2.8 Hz), 115.3 (d, *J* = 20.8 Hz), 112.9 (d, *J* = 1.9 Hz), 61.4, 35.1, 31.6, 22.1, 15.5, 14.2, 12.7; **$^{19}$F NMR** (376.5, $CDCl_3$) δ -113.8 to -113.9 (m, 1F), -136.8 (d, *J* = 31.3 Hz, 1F).

**Minor Diastereomer: $^1$H NMR** (500 MHz, $CDCl_3$) δ 7.32–7.25 (m, 1H), 7.03–6.98 (m, 1H), 6.97–6.90 (m, 2H), 5.65 (dd, *J* = 31.8, 10.6 Hz, 1H), 4.32 (q, *J* = 7.1 Hz, 2H), 2.81–2.72 (m, 2H), 1.77–1.66 (m, 2H), 1.38 (t, *J* = 7.1 Hz, 3H), 1.99–1.90 (m, 1H), 1.87–1.78 (m, 1H), 1.64–1.58 (m, 1H), 0.51 (q, *J* = 5.5 Hz, 1H); **$^{13}$C NMR** (125.7 MHz, $CDCl_3$) δ 162.9 (d, *J* = 245.1 Hz), 160.7 (d, *J* = 33.8 Hz), 148.8 (d, , *J* = 253.9 Hz), 144.3 (d, *J* = 4.6 Hz), 129.7, 124.2 (d, *J* = 2.8 Hz), 122.1 (d, *J* = 10.2 Hz), 115.3 (d, , *J* = 20.8 Hz), 112.7 (d, *J* = 1.9 Hz), 61.4, 35.2, 31.6, 20.2, 15.3, 14.2, 12.7; **$^{19}$F NMR** (376.5, $CDCl_3$) δ -113.7 to -113.8 (m, 1F), -135.0 (d, *J* = 31.3 Hz, 1F).

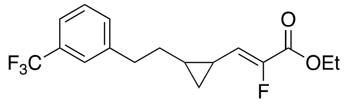


**Cyclopropane (6)** was prepared according to Method A. The following amounts of reagents were used: mesylate **SI-15** (47 mg, 0.11 mmol, 1.0 equiv), $Ni(PMe_3)_2Cl_2$ (1.5 mg, 5.3 µmol, 5.0 mol %), $Zn^0$ (14 mg, 0.21 mmol, 2.0 equiv), NaBr (22 mg, 0.21 mmol, 2.0 equiv), and anhydrous MeCN (1.1 mL, 0.10 M in substrate). Before purification, a $^1$H NMR yield of 51% was obtained based on comparison to PhTMS as internal standard. The residue was then purified by flash column chromatography (0–10% EtOAc/hexanes) to afford the title compound as a mixture of cyclopropane diastereomers as a yellow oil (21 mg, 62 µmol, 58%, 1.2:1 dr). The dr was

determined by integration of the alkene proton resonances of each cyclopropane diastereomer in the $^1$H NMR spectrum. The relative configuration of cyclopropane was assigned based on analogy to cyclopropane **14**. **TLC R$_f$** = 0.3 (10% EtOAc/hexanes); **HRMS** (TOF MS ES+) *m/z*: [M + Na]$^+$ calculated for $C_{17}H_{18}F_4O_2Na$, 353.1141; found 353.1137. For clarity, the $^1$H NMR, $^{13}$C NMR, and $^{19}$F NMR data of the major and minor diastereomers have been tabulated individually.

**Major diastereomer: $^1$H NMR** (400 MHz, $CDCl_3$) *δ* 7.46–7.34 (m, 4H), 5.58 (dd, *J* = 31.8, 10.6 Hz, 1H), 4.26 (q, *J* = 6.8 Hz, 2H), 2.77 (aq, *J* = 6.5 Hz, 2H), 1.94–1.51 (m, 3H), 1.32 (t, *J* = 7.1 Hz, 3H), 1.02–0.94 (m, 1H), 0.80 (at, *J* = 7.1 Hz, 2H); **$^{13}$C NMR** (150.9 MHz, $CDCl_3$) δ 160.9 (d, *J* = 33.8 Hz), 147.8 (d, *J* = 252.4 Hz), 142.7, 132.0, 130.7 (q, *J* = 31.9 Hz), 128.9, 125.4, 125.3 (d, *J* = 10.8 Hz), 124.4 (q, *J* = 272.5 Hz), 123.0 (q, *J* = 3.9 Hz), 61.5, 35.4, 35.3, 22.1, 15.5 (2C), 14.3; **$^{19}$F NMR** (125.7 MHz, $CDCl_3$) δ -62.8 (s, 3F), -136.8 (d, *J* = 31.7 Hz, 1F).

**Minor diastereomer: $^1$H NMR** (400 MHz, $CDCl_3$) *δ* 7.46–7.34 (m, 4H), 5.80 (dd, *J* = 31.4, 10.9 Hz, 1H), 4.26 (q, *J* = 6.8 Hz, 2H), 2.77 (aq, *J* = 6.5 Hz, 2H), 1.94–1.51 (m, 3H), 1.32 (t, *J* = 7.1 Hz, 3H), 1.28–1.14 (m, 2H), 0.45 (aq, *J* = 5.4 Hz, 1H); **$^{13}$C NMR** (150.9 MHz, $CDCl_3$) δ 160.8 (d, *J* = 33.8 Hz), 149.1 (d, *J* = 254.4 Hz), 142.8, 132.1, 130.8 (q, *J* = 31.9 Hz), 128.9, 125.4, 124.4 (q, *J* = 272.5 Hz), 123.0 (q, *J* = 3.9 Hz), 122.0 (d, *J* = 10.0 Hz), 61.5, 35.7, 31.8, 20.2, 15.4, 14.3, 12.7; **$^{19}$F NMR** (125.7 MHz, $CDCl_3$) δ -62.8 (s, 3F), -134.9 (d, *J* = 31.6 Hz, 1F).

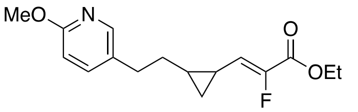


**Cyclopropane (7)** was prepared according to Method A. The following amounts of reagents were used: mesylate **SI-16** (44 mg, 0.11 mmol, 1.0 equiv), $Ni(PMe_3)_2Cl_2$ (1.6 mg, 5.5 µmol, 5.0 mol %), $Zn^0$ (14 mg, 0.22 mmol, 2.0 equiv), NaBr (22 mg, 0.22 mmol, 2.0 equiv), and MeCN (1.0 mL, 0.11 M in substrate). Before purification, a $^1$H NMR yield of 49% was obtained based on comparison to PhTMS as internal standard. The compound was purified by flash column chromatography (0–10% EtOAc/hexanes) to afford the title compound as a mixture of cyclopropane diastereomers as a clear, colorless oil (15 mg, 53 µmol, 48%, 1:1 dr). The dr was determined by integration of the alkene proton resonances of each cyclopropane diastereomer in the $^1$H NMR spectrum. The relative configuration of cyclopropane was assigned based on analogy to cyclopropane **14**. **TLC R$_f$** = 0.2 (10% EtOAc/hexanes); **HRMS** (TOF MS ES+) *m/z*: [M+Na]$^+$ $C_{16}H_{20}FNO_3Na$ calculated for 316.1325, found 316.1317. For clarity, the $^1$H NMR, $^{13}$C NMR, and $^{19}$F NMR data of the major and minor diastereomers have been tabulated individually.

**Major Diastereomer: $^1$H NMR** (500 MHz, $CDCl_3$) δ 8.05–7.99 (m, 1H), 7.45 (d, *J* = 8.6 Hz, 1H), 6.73 (dd, *J* = 8.4, 4.3 Hz, 1H), 5.65 (dd, *J* = 31.8, 10.7 Hz, 1H), 4.32 (quint, *J* = 7.0 Hz, 2H), 3.97 (s, 3H), 2.73–2.66 (m, 2H), 1.81–1.73 (m, 1H), 1.38 (q, *J* = 7.3 Hz, 3H), 1.35–1.26 (m, 2H), 1.09–1.01 (m, 1H), 0.86 (t, *J* = 6.7 Hz, 2H); **$^{13}$C NMR** (125.7 MHz, $CDCl_3$) δ 162.8, 160.8 (d, *J* = 33.7 Hz), 147.3 (d, *J* = 252.1 Hz), 146.1, 138.9, 129.5, 125.4 (d, *J* = 10.6 Hz), 110.5, 61.39, 53.4, 32.0, 31.67, 21.9, 15.45, 14.2, 12.6.; **$^{19}$F NMR** (376.5, $CDCl_3$) δ -136.7 (d, *J* = 31.3 Hz, 1F).

**Minor Diastereomer: $^1$H NMR** (500 MHz, $CDCl_3$) δ 8.05–7.99 (m, 1H), 7.45 (d, *J* = 8.6 Hz, 1H), 6.73 (dd, *J* = 8.4, 4.3 Hz, 1H), 5.65 (dd, *J* = 31.8, 10.7 Hz, 1H), 4.32 (quint, *J* = 7.0 Hz, 2H), 3.97 (s, 3H), 2.73–2.66 (m, 2H), 1.99–1.90 (m, 1H), 1.38 (q, *J* = 7.3 Hz, 3H), 1.35–1.26 (m, 2H), 1.25–

1.19 (m, 1H), 0.97–0.89 (m, 1H), 0.51 (q, $J$ = 5.4 Hz, 1H); **$^{13}$C NMR** (125.7 MHz, $CDCl_3$) δ 162.8, 160.7 (d, $J$ = 33.7 Hz), 148.9 (d, $J$ = 254.3 Hz),146.2, 139.0, 129.6, 122.0 (d, $J$ = 10.2 Hz), 110.5, 61.4, 53.4, 35.3, 31.7, 20.1, 15.42 14.3, 12.6; **$^{19}$F NMR** (376.5, $CDCl_3$) δ -134.9 (d, $J$ = 31.3 Hz, 1F).

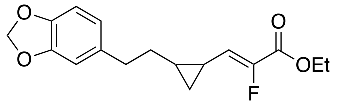


**Cyclopropane (8)** was prepared according to Method A. The following amounts of reagents were used: mesylate **SI-17** (34 mg, 82 µmol, 1.0 equiv), $Ni(PMe_3)_2Cl_2$ (1.2 mg, 4.1 µmol, 5.0 mol %), $Zn^0$ (11 mg, 0.16 mmol, 2.0 equiv), NaBr (17 mg, 0.16 mmol, 2.0 equiv), and MeCN (1.0 mL, 0.080 M in substrate). Before purification, a $^1$H NMR yield of 38% was obtained based on comparison to PhTMS as internal standard. The compound was purified by flash column chromatography (0–10% EtOAc/hexanes) to afford the title compound as a mixture of cyclopropane diastereomers as a clear, colorless oil (11 mg, 35 µmol, 43%, 1:1 dr). The dr was determined by integration of the alkene proton resonances of each cyclopropane diastereomer in the $^1$H NMR spectrum. The relative configuration of cyclopropane was assigned based on analogy to cyclopropane **14**. **TLC $R_f$** = 0.3 (10% EtOAc/hexanes); **HRMS** (TOF MS ES+) *m/z*: $[M+Na]^+$ calculated for $C_{17}H_{19}FO_4Na$, 329.1165; found 329.1152. For clarity, the $^1$H NMR, $^{13}$C NMR, and $^{19}$F NMR data of the major and minor diastereomers have been tabulated individually.

**Major Diastereomer: $^1$H NMR** (400 MHz, $CDCl_3$) δ 6.81–6.63 (m, 3H), 5.98 (s, 2H), 5.85 (dd, $J$ = 32.0, 11.0 Hz, 1H), 4.33 (quint, $J$ = 7.0 Hz, 2H), 2.75–2.63 (m, 2H), 1.74–1.65 (m, 1H), 1.65–1.56 (m, 2H), 1.38 (q, $J$ = 7.2 Hz, 3H), 1.09–1.01 (m, 1H), 0.98–0.82 (m, 2H); **$^{13}$C NMR** (125.7 MHz, $CDCl_3$) δ 160.9 (d, $J$ = 33.8 Hz), 148.8 (d, $J$ = 253.4 Hz), 145.7, 135.6, 125.6 (d, $J$ = 10.6 Hz), 121.2, 109.0, 108.1, 100.8, 61.3, 35.7, 35.2, 32.2, 22.1, 15.5, 14.3, 12.7; **$^{19}$F NMR** (376.5, $CDCl_3$) δ -137.0 (d, $J$ = 32.7 Hz, 1F).

**Minor Diastereomer: $^1$H NMR** (400 MHz, $CDCl_3$) δ 6.81–6.63 (m, 3H), 5.98 (s, 2H), 5.65 (dd, $J$ = 32.0, 10.7 Hz, 1H), 4.33 (quint, $J$ = 7.0 Hz, 2H), 2.75–2.63 (m, 2H), 1.98–1.89 (m, 1H), 1.84–1.75 (m, 1H), 1.74–1.65 (m, 1H), 1.65–1.56 (m, 2H), 1.38 (q, $J$ = 7.2 Hz, 3H), 0.53–0.48 (m, 1H); **$^{13}$C NMR** (125.7 MHz, $CDCl_3$) δ 160.7 (d, $J$ = 33.8 Hz), 147.7 (d, $J$ = 251.5 Hz), 145.7, 135.6, 122.3 (d, $J$ = 10.2 Hz), 121.2, 109.0, 108.1, 100.8, 61.3, 35.6, 35.2, 32.2, 20.2, 15.3, 14.3, 12.7; **$^{19}$F NMR** (376.5, $CDCl_3$) δ -135.3 (d, $J$ = 31.3 Hz 1F).

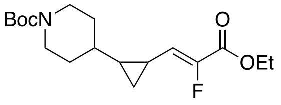


**Cyclopropane (9)** was prepared according to Method A. The following amounts of reagents were used: mesylate **SI-18** (43 mg, 95 µmol, 1.0 equiv), $Ni(PMe_3)_2Cl_2$ (1.3 mg, 4.7 µmol, 5.0 mol %), $Zn^0$ (12 mg, 0.19 mmol, 2.0 equiv), NaBr (20. mg, 0.19 mmol, 2.0 equiv), and anhydrous MeCN (0.95 mL, 0.10 M in substrate). Before purification, a $^1$H NMR yield of 44% was obtained based on comparison to PhTMS as internal standard. The residue was then purified by flash column chromatography (0–10% EtOAc/hexanes) to afford the title compound as a mixture of cyclopropane diastereomers as a pale-yellow oil (13 mg, 39 µmol, 30%, 2.5:1 dr). The dr was determined by integration of the alkene proton resonances of each cyclopropane diastereomer in

the $^{1}$H NMR spectrum. The relative configuration of cyclopropane was assigned based on analogy to cyclopropane **14**. **TLC R$_f$** = 0.5 (20% EtOAc/hexanes); **HRMS** (TOF MS ES+) *m/z*: $[M + Na]^+$ calculated for $C_{18}H_{28}FNO_4Na$, 364.1900; found 364.1897; **SFC Analysis** (Chiralcel OD-H, 2% IPA/$CO_2$, 1.5 mL/min, 230 nm) indicated 2% ee: $t_R$ (minor diastereomer, both enantiomers) = 17.9 minutes, $t_R$ (major diastereomer, major enantiomer) = 23.1 minutes, $t_R$ (major diastereomer, minor enantiomer) = 28.6 minutes. For clarity, the $^{1}$H NMR, $^{13}$C NMR, and $^{19}$F NMR data of the major and minor diastereomers have been tabulated individually.

**Major diastereomer: $^{1}$H NMR** (400 MHz, $CDCl_3$) $\delta$ 5.61 (dd, *J* = 31.8, 10.6 Hz, 1H), 4.26 (q, *J* = 7.1 Hz, 2H), 4.08 (br s, 2H), 2.65 (at, *J* = 12.6 Hz, 2H), 1.74–1.62 (m, 4H), 1.46 (s, 9H), 1.32 (t, *J* = 7.1 Hz, 3H), 1.29–1.26 (m, 2H), 0.87–0.79 (m, 3H); **$^{13}$C NMR** (125.7 MHz, $CDCl_3$) δ 160.9 (d, *J* = 33.8 Hz), 155.0, 147.8 (d, *J* = 252.3 Hz), 125.4 (d, *J* = 10.7 Hz), 79.5, 61.5, 43.9 (2C), 40.7, 37.4, 31.6 (2C), 28.6 (3C), 28.1, 14.3, 14.1; **$^{19}$F NMR** (376.5 MHz, $CDCl_3$) δ -136.9 (d, *J* = 31.7 Hz, 1F).

**Minor diastereomer: $^{1}$H NMR** (400 MHz, $CDCl_3$) $\delta$ 5.84 (dd, *J* = 31.4, 10.9 Hz, 1H), 4.26 (q, *J* = 7.1 Hz, 2H), 4.08 (br s, 2H), 2.65 (at, *J* = 12.6 Hz, 2H), 1.96–1.88 (m, 1H), 1.74–1.62 (m, 3H), 1.46 (s, 9H), 1.32 (t, *J* = 7.1 Hz, 3H), 1.29–1.26 (m, 2H), 1.19–1.01 (m, 2H), 0.52–0.48 (m, 1H); **$^{13}$C NMR** (125.7 MHz, $CDCl_3$) δ 160.8 (d, *J* = 33.8 Hz), 155.0, 149.2 (d, *J* = 257.3 Hz), 122.2 (d, *J* = 9.1 Hz), 79.5, 61.6, 43.9 (2C), 40.7, 37.0, 31.6 (2C), 28.6 (3C), 26.5, 14.2, 12.5; **$^{19}$F NMR** (376.5 MHz, $CDCl_3$) δ -134.9 (d, *J* = 32.1 Hz, 1F).

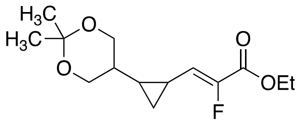


**Cyclopropane (10)** was prepared according to Method A. The following amounts of reagents were used: mesylate **SI-19** (31 mg, 80 μmol, 1.0 equiv), $Ni(PMe_3)_2Cl_2$ (1.1 mg, 4.0 μmol, 5.0 mol %), $Zn^0$ (11 mg, 0.16 mmol, 2.0 equiv), NaBr (17 mg, 0.16 mmol, 2.0 equiv), and anhydrous MeCN (0.80 mL, 0.10 M in substrate). Before purification, a $^{1}$H NMR yield of 36% was obtained based on comparison to PhTMS as internal standard. The residue was then purified by flash column chromatography (0–10% EtOAc/hexanes) to afford the title compound as a mixture of cyclopropane diastereomers as a clear, colorless oil (9.0 mg, 33 μmol, 41%, 2:1 dr). The dr was determined by integration of the alkene proton resonances of each cyclopropane diastereomer in the $^{1}$H NMR spectrum. The relative configuration of cyclopropane was assigned based on analogy to cyclopropane **14**. **TLC R$_f$** = 0.5 (30% EtOAc/hexanes); **HRMS** (TOF MS CI+) *m/z*: $[M + H]^+$ calculated for $C_{14}H_{21}FO_4H$, 273.1502; found 273.1495. For clarity, the $^{1}$H NMR, $^{13}$C NMR, and $^{19}$F NMR data of the major and minor diastereomers have been tabulated individually.

**Major diastereomer: $^{1}$H NMR** (500 MHz, $CDCl_3$) $\delta$ 5.61 (dd, *J* = 31.6, 10.5 Hz, 1H), 4.26 (q, *J* = 7.0 Hz, 2H), 3.96–3.83 (m, 2H), 3.79–3.66 (m, 2H), 1.66–1.63 (m, 1H), 1.44 (s, 3H), 1.41 (s, 3H), 1.32 (t, *J* = 7.0 Hz, 3H), 1.19–1.11 (m, 2H), 0.88–0.82 (m, 2H); **$^{13}$C NMR** (125.7 MHz, $CDCl_3$) δ 160.7, 148.1 (d, *J* = 252.1 Hz), 124.6 (d, *J* = 10.6 Hz), 98.1, 64.1 (2C), 61.6, 39.2, 25.7, 22.5, 21.5, 14.3 (2C), 13.8; **$^{19}$F NMR** (376.5 MHz, $CDCl_3$) δ -136.1 (d, *J* = 31.5 Hz, 1F).

**Minor diastereomer: $^{1}$H NMR** (500 MHz, $CDCl_3$) $\delta$ 5.80 (dd, *J* = 31.1, 10.8 Hz, 1H), 4.26 (q, *J* = 7.0 Hz, 2H), 3.96–3.83 (m, 2H), 3.79–3.66 (m, 2H), 1.97–1.90 (m, 1H), 1.66–1.63 (m, 1H), 1.46

(s, 3H), 1.41 (s, 3H), 1.33 (t, $J$ = 6.9 Hz, 3H), 1.28–1.26 (m, 1H), 1.10–1.06 (m, 1H), 0.52–0.51 (m, 1H); **$^{13}$C NMR** (125.7 MHz, $CDCl_3$) δ 161.0, 149.5 (d, $J$ = 254.4 Hz), 121.1 (d, $J$ = 9.9 Hz), 98.0, 64.6 (2C), 61.7, 35.6, 26.9, 21.2, 19.3, 13.8 (2C), 11.6; **$^{19}$F NMR** (376.5 MHz, $CDCl_3$) δ -133.3 (d, $J$ = 31.2 Hz, 1F).

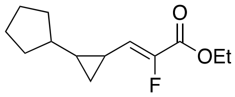


**Cyclopropane (11)** was prepared according to Method A. The following amounts of reagents were used: mesylate **SI-20** (43 mg, 0.13 mmol, 1.0 equiv), $NiCl_2(PMe_3)_2$ (1.7 mg, 6.0 µmol, 5.0 mol %), $Zn^0$ (17 mg, 0.26 mmol, 2.0 equiv), NaBr (27 mg, 0.26 mmol, 2.0 equiv), and MeCN (1.0 mL, 0.10 M in substrate). Before purification, a $^1$H NMR yield of 65% was obtained based on comparison to PhTMS as internal standard. The compound was purified by flash column chromatography (0–10% EtOAc/hexanes) to afford the title compound as a mixture of cyclopropane diastereomers as a yellow oil (13 mg, 56 µmol, 44%, 2:1 dr). The dr was determined by integration of the alkene proton resonances of each cyclopropane diastereomer in the $^1$H NMR spectrum. The relative configuration of cyclopropane was assigned based on analogy to cyclopropane **14**. **TLC $R_f$** = 0.6 (10% EtOAc/hexanes); **HRMS** (TOF MS CI+) *m/z*: $[M+H]^+$ calculated for $C_{13}H_{19}FO_2H$, 227.1447; found 227.1452; **HPLC Analysis** (Chiralcel OJ, 1.0% IPA/hexanes, 2.0 mL/min, 230 nm) indicated 1% ee: $t_R$ (minor diastereomer, both enantiomers) = 3.2 minutes, $t_R$ (major diastereomer, minor enantiomer) = 3.7 minutes, $t_R$ (major diastereomer, major enantiomer) = 4.0 minutes. For clarity, the $^1$H NMR, $^{13}$C NMR, and $^{19}$F NMR data of the major and minor diastereomers have been tabulated individually.

**Major Diastereomer: $^1$H NMR** (500 MHz, $CDCl_3$) δ 5.68 (dd, $J$ = 32.3, 10.8 Hz, 1H), 4.36–4.28 (m, 2H), 1.85–1.76 (m, 2H), 1.75–1.64 (m, 3H), 1.62–1.53 (m, 2H), 1.37 (t, $J$ = 7.0 Hz, 3H), 1.36–1.29 (m, 2H), 1.23–1.16 (m, 1H), 1.04–0.97 (m, 1H), 0.94–0.89 (m, 1H), 0.85–0.79 (m, 1H); **$^{13}$C NMR** (125.7 MHz, $CDCl_3$) δ 161.0 (d, $J$ = 33.8 Hz), 147.3 (d, $J$ = 251.1 Hz), 126.2 (d, $J$ = 10.6 Hz), 61.3, 43.9, 31.8 (2C), 27.7, 25.1, 14.8, 14.6, 14.5, 14.2; **$^{19}$F NMR** (376.5, $CDCl_3$) δ -137.6 (d, $J$ = 32.7 Hz, 1F).

**Minor Diastereomer: $^1$H NMR** (500 MHz, $CDCl_3$) δ 5.95 (dd, $J$ = 32.0, 11.0 Hz, 1H), 4.36–4.28 (m, 2H), 1.97–1.85 (m, 2H), 1.85–1.76 (m, 2H), 1.75–1.64 (m, 3H), 1.62–1.53 (m, 2H), 1.53–1.45 (m, 1H), 1.37 (t, $J$ = 7.0 Hz, 3H), 1.36–1.29 (m, 2H), 0.59–0.55 (m, 1H); **$^{13}$C NMR** (125.7 MHz, $CDCl_3$) δ 160.9 (d, $J$ = 33.8 Hz), 148.8 (d, $J$ =253.4 Hz), 123.4 (d, $J$ = 10.2 Hz), 61.3, 41.4, 33.0, 32.3, 26.7, 25.2, 15.4, 14.2, 13.0, 12.9; **$^{19}$F NMR** (376.5, $CDCl_3$) δ -136.1 (d, $J$ = 31.3 Hz, 1F).

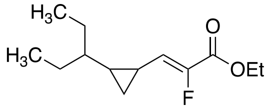


**Cyclopropane (12)** was prepared according to Method A. The following amounts of reagents were used: mesylate **SI-21** (36 mg, 0.10 mmol, 1.0 equiv), $Ni(PMe_3)_2Cl_2$ (1.5 mg, 5.2 µmol, 5.0 mol %), $Zn^0$ (14 mg, 0.21 mmol, 2.0 equiv), NaBr (21 mg, 0.21 mmol, 2.0 equiv), and anhydrous MeCN (1.0 mL, 0.10 M in substrate). Before purification, a $^1$H NMR yield of 64% was obtained based on comparison to PhTMS as internal standard. The residue was then purified by flash column chromatography (0–10% EtOAc/hexanes) to afford the title compound as a mixture of

cyclopropane diastereomers as a pale-yellow oil (12 mg, 52 μmol, 50%, 3:1 dr). The dr was determined by integration of the alkene proton resonances of each cyclopropane diastereomer in the $^{1}$H NMR spectrum. The relative configuration of cyclopropane was assigned based on analogy to cyclopropane **14**. **TLC R$_f$** = 0.5 (10% EtOAc/hexanes); **HRMS** (TOF MS ES+) *m/z*: [M + Na]$^+$ calculated for $C_{13}H_{21}FO_2Na$, 251.1423; found 251.1433. For clarity, the $^{1}$H NMR, $^{13}$C NMR, and $^{19}$F NMR data of the major and minor diastereomers have been tabulated individually.

**Major diastereomer: $^{1}$H NMR** (400 MHz, $CDCl_3$) *δ* 5.63 (dd, *J* = 32.0, 10.8 Hz, 1H), 4.26 (q, *J* = 7.1 Hz, 2H), 1.63–1.56 (m, 1H), 1.52–1.03 (m, 4H), 1.32 (t, *J* = 7.1 Hz, 3H), 0.96–0.75 (m, 3H), 0.91 (t, *J* = 7.5 Hz, 3H), 0.90 (t, *J* = 7.5 Hz, 3H), 0.66–0.60 (m, 1H); **$^{13}$C NMR** (100 MHz, $CDCl_3$) δ 161.3, 147.7 (d, *J* = 251.5 Hz), 126.2 (d, *J* = 10.7 Hz), 61.4, 45.8, 27.4, 26.9, 26.7, 15.1, 14.9, 14.3, 11.4, 10.9; **$^{19}$F NMR** (376.5 MHz, $CDCl_3$) δ -138.0 (d, *J* = 32.0 Hz, 1F).

**Minor diastereomer: $^{1}$H NMR** (400 MHz, $CDCl_3$) *δ* 5.85 (dd, *J* = 31.8, 11.1 Hz, 1H), 4.27 (q, *J* = 6.9 Hz, 2H), 1.92–1.84 (m, 1H), 1.63–1.56 (m, 1H), 1.52–1.03 (m, 4H), 1.33 (t, *J* = 7.1 Hz, 3H), 0.96–0.75 (m, 8H), 0.52–0.48 (m, 1H); **$^{13}$C NMR** (100 MHz, $CDCl_3$) δ 160.9, 147.7 (d, *J* = 251.5 Hz), 123.7 (d, *J* = 10.2 Hz), 68.7, 41.4, 27.4, 27.0, 25.9, 15.3, 15.1, 12.5, 11.5, 10.9; **$^{19}$F NMR** (376.5 MHz, $CDCl_3$) δ -136.5 (d, *J* = 31.7 Hz, 1F).

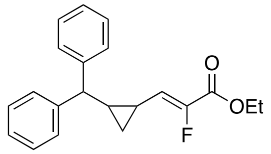


**Cyclopropane (13)** was prepared according to Method A. The following amounts of reagents were used: mesylate **SI-22** (42 mg, 96 μmol, 1.0 equiv), $Ni(PMe_3)_2Cl_2$ (1.3 mg, 4.8 μmol, 5.0 mol %), $Zn^0$ (13 mg, 0.19 mmol, 2.0 equiv), NaBr (20. mg, 0.19 mmol, 2.0 equiv), and anhydrous MeCN (0.96 mL, 0.10 M in substrate). Before purification, a $^{1}$H NMR yield of 43% was obtained based on comparison to PhTMS as internal standard. The residue was then purified by flash column chromatography (0–20% EtOAc/hexanes) to afford the title compound as a mixture of cyclopropane diastereomers as a pale-yellow oil (13 mg, 39 μmol, 40%, 2.6:1 dr). The dr was determined by integration of the alkene proton resonances of each cyclopropane diastereomer in the $^{1}$H NMR spectrum. The relative configuration of cyclopropane was assigned based on analogy to cyclopropane **14**. **TLC R$_f$** = 0.4 (10% EtOAc/hexanes); **HRMS** (TOF MS CI+) *m/z*: [M + H]$^+$ calculated for $C_{21}H_{21}FO_2H$, 325.1604; found 325.1607. For clarity, the $^{1}$H NMR, $^{13}$C NMR, and $^{19}$F NMR data of the major and minor diastereomers have been tabulated individually.

**Major diastereomer: $^{1}$H NMR** (500 MHz, $CDCl_3$) *δ* 7.31–7.18 (m, 10H), 5.72 (dd, *J* = 31.6, 10.6 Hz, 1H), 4.26 (q, *J* = 7.0 Hz, 2H), 3.50 (d, *J* = 9.1 Hz, 1H), 1.85–1.80 (m, 1H), 1.32 (t, *J* = 7.2 Hz, 3H), 1.02–0.72 (m, 3H); **$^{13}$C NMR** (125.7 MHz, $CDCl_3$) δ 161.0 (d, *J* = 34.2 Hz), 148.2, 144.2, 143.8, 128.6 (2C), 128.5 (2C), 128.4 (2C), 128.2 (2C), 128.1, 126.6, 124.6 (d, *J* = 10.7 Hz), 61.6, 54.6, 29.8, 27.6, 16.1, 14.5; **$^{19}$F NMR** (376.5 MHz, $CDCl_3$) δ -136.0 (d, *J* = 31.6 Hz, 1F).

**Minor diastereomer: $^{1}$H NMR** (500 MHz, $CDCl_3$) *δ* 7.31–7.18 (m, 10H), 5.93 (dd, *J* = 31.3, 10.7 Hz, 1H), 4.26 (q, *J* = 7.0 Hz, 2H), 3.57 (d, *J* = 11.0 Hz, 1H), 1.97–1.92 (m, 1H), 1.32 (t, *J* = 7.2 Hz, 3H), 1.02–0.72 (m, 3H); **$^{13}$C NMR** (125.7 MHz, $CDCl_3$) δ 161.0 (d, *J* = 34.2 Hz), 148.2, 144.9,

144.0, 128.6 (2C), 128.5 (2C), 128.4 (2C), 128.2 (2C), 128.1, 126.6, 121.7 (d, $J$ = 9.9 Hz), 61.5, 51.3, 30.1, 26.4, 15.9, 15.5; **$^{19}$F NMR** (376.5 MHz, $CDCl_3$) δ -134.5 (d, $J$ = 31.4 Hz, 1F).

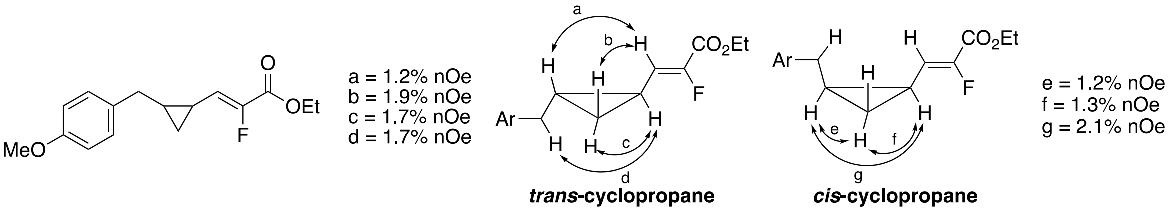


**Cyclopropane (14)** was prepared according to Method A. The following amounts of reagents were used: mesylate **SI-23** (40. mg, 0.10 mmol, 1.0 equiv), $Ni(PMe_3)_2Cl_2$ (1.4 mg, 5.1 μmol, 5.0 mol %), $Zn^0$ (13 mg, 0.21 mmol, 2.0 equiv), NaBr (21 mg, 0.21 mmol, 2.0 equiv), and anhydrous MeCN (1.0 mL, 0.10 M in substrate). Before purification, a $^1$H NMR yield of 48% was obtained based on comparison to PhTMS as internal standard. The residue was then purified by flash column chromatography (0–10% EtOAc/hexanes) to afford the title compound as a mixture of cyclopropane diastereomers as a pale-yellow oil (14 mg, 51 μmol, 50%, 1.7:1 dr). The dr was determined by integration of the alkene proton resonances of each cyclopropane diastereomer in the $^1$H NMR spectrum. The relative configuration of cyclopropane was assigned based on nOe analysis. **TLC $R_f$** = 0.4 (10% EtOAc/hexanes); **HRMS** (TOF MS Cl+) *m/z*: $[M]^+$ calculated for $C_{16}H_{19}FO_3$, 278.1318; found 278.1331. For clarity, the $^1$H NMR, $^{13}$C NMR, and $^{19}$F NMR data of the major and minor diastereomers have been tabulated individually.

**Major diastereomer: $^1$H NMR** (600 MHz, $CDCl_3$) $\delta$ 7.13 (d, $J$ = 8.4 Hz, 2H), 6.84 (d, $J$ = 8.4 Hz, 2H), 5.64 (dd, $J$ = 31.9, 10.7 Hz, 1H), 4.25 (q, $J$ = 7.3 Hz, 2H), 3.79 (s, 3H), 2.65 (dd, $J$ = 14.9, 6.9 Hz, 1H), 2.61 (dd, $J$ = 14.7, 6.7 Hz, 1H), 1.76–1.71 (m, 1H), 1.34–1.29 (m, 3H), 1.27–1.20 (m, 1H), 0.96–0.93 (m, 1H), 0.89–0.85 (m, 1H); **$^{13}$C NMR** (150.9 MHz, $CDCl_3$) δ 161.0 (d, $J$ = 33.8 Hz), 158.2, 147.9 (d, $J$ = 252.1 Hz), 132.7, 129.3 (2C), 125.4 (d, $J$ = 10.7 Hz), 113.98 (2C), 61.5, 55.4, 38.3, 23.6, 15.6, 15.3, 14.3; **$^{19}$F NMR** (564.6 MHz, $CDCl_3$) δ -136.8 (d, $J$ = 32.0 Hz, 1F).

**Minor diastereomer: $^1$H NMR** (600 MHz, $CDCl_3$) $\delta$ 7.15 (d, $J$ = 8.5 Hz, 2H), 6.84 (d, $J$ = 8.4 Hz, 2H), 5.95 (dd, $J$ = 31.6, 10.9 Hz, 1H), 4.28 (q, $J$ = 7.3 Hz, 2H), 3.79 (s, 3H), 2.73 (dd, $J$ = 15.1, 6.8 Hz, 1H), 2.67–2.59 (m, 1H), 2.02–1.97 (m, 1H), 1.55–1.49 (m, 1H), 1.34–1.29 (m, 3H), 1.27–1.20 (m, 1H), 0.65–0.62 (m, 1H); **$^{13}$C NMR** (150.9 MHz, $CDCl_3$) δ 160.9 (d, $J$ = 33.8 Hz), 158.1, 149.1 (d, $J$ = 252.4 Hz), 133.2, 129.2 (2C), 122.3 (d, $J$ = 9.9 Hz), 113.99 (2C), 61.5, 55.4, 34.7, 21.9, 15.6, 14.3, 13.2; **$^{19}$F NMR** (564.6 MHz, $CDCl_3$) δ -134.9 (d, $J$ = 31.5 Hz, 1F).

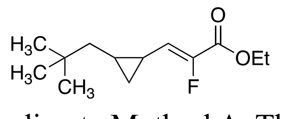

**Cyclopropane (15)** was prepared according to Method A. The following amounts of reagents were used: mesylate **SI-24** (34 mg, 0.10 mmol, 1.0 equiv), $Ni(PMe_3)_2Cl_2$ (1.4 mg, 5.4 μmol, 5.0 mol %), $Zn^0$ (13 mg, 0.20 mmol, 2.0 equiv), NaBr (21 mg, 0.20 mmol, 2.0 equiv), and anhydrous MeCN (1.0 mL, 0.10 M in substrate). Before purification, a $^1$H NMR yield of 62% was obtained based on comparison to PhTMS as internal standard. The residue was then purified by flash column chromatography (0–10% EtOAc/hexanes) to afford the title compound as a mixture of cyclopropane diastereomers as a pale-yellow oil (12 mg, 51 μmol, 51%, 2.6:1 dr). The dr was

determined by integration of the alkene proton resonances of each cyclopropane diastereomer in the $^{1}$H NMR spectrum. The relative configuration of cyclopropane was assigned based on analogy to cyclopropane **14**. **TLC R$_f$** = 0.7 (10% EtOAc/hexanes); **HRMS** (TOF MS CI+) *m/z*: [M + H]$^+$ calculated for $C_{13}H_{21}FO_2H$, 229.1604; found 229.1606. For clarity, the $^{1}$H NMR, $^{13}$C NMR, and $^{19}$F NMR data of the major and minor diastereomers have been tabulated individually.

**Major diastereomer: $^{1}$H NMR** (600 MHz, $CDCl_3$) *δ* 5.63 (dd, *J* = 32.0, 10.8 Hz, 1H), 4.26 (q, *J* = 7.2 Hz, 2H), 1.56–1.52 (m, 1H), 1.32 (t, *J* = 7.0 Hz, 3H), 1.23–1.10 (m, 2H), 0.93 (s, 9H), 0.90–0.76 (m, 3H); **$^{13}$C NMR** (150.9 MHz, $CDCl_3$) δ 161.1 (d, *J* = 33.8 Hz), 147.8 (d, *J* = 251.6 Hz), 126.1 (d, *J* = 10.8 Hz), 61.4, 48.0, 43.6, 31.7, 29.7 (3C), 19.2, 16.0, 14.3; **$^{19}$F NMR** (564.6 MHz, $CDCl_3$) δ -137.7 (d, *J* = 31.9 Hz, 1F).

**Minor diastereomer: $^{1}$H NMR** (600 MHz, $CDCl_3$) *δ* 5.85 (dd, *J* = 31.8, 11.0 Hz, 1H), 4.26 (q, *J* = 7.2 Hz, 2H), 1.89–1.83 (m, 1H), 1.33 (t, *J* = 6.7 Hz, 3H), 1.23–1.10 (m, 2H), 0.94 (s, 9H), 0.90–0.76 (m, 2H), 0.45–0.42 (m, 1H); **$^{13}$C NMR** (150.9 MHz, $CDCl_3$) δ 161.0 (d, *J* = 33.8 Hz), 148.8 (d, *J* = 253.5 Hz), 123.3 (d, *J* = 9.9 Hz), 61.5, 48.0, 43.6, 31.6, 29.6 (3C), 15.7, 14.4, 12.3; **$^{19}$F NMR** (564.6 MHz, $CDCl_3$) δ -136.2 (d, *J* = 31.8 Hz, 1F).

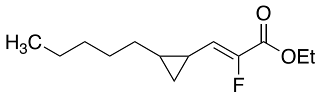


**Cyclopropane (16)** was prepared according to Method A. The following amounts of reagents were used: mesylate **SI-25** (33 mg, 96 µmol, 1.0 equiv), $Ni(PMe_3)_2Cl_2$ (1.4 mg, 4.8 µmol, 5.0 mol %), $Zn^0$ (12 mg, 0.19 mmol, 2.0 equiv), NaBr (20. mg, 0.192 mmol, 2.0 equiv), and MeCN (1.0 mL, 0.096 M in substrate). Before purification, a $^{1}$H NMR yield of 55% was obtained based on comparison to PhTMS as internal standard. The compound was purified by flash column chromatography (10% $Et_2O$/pentanes) to afford the title compound as a mixture of cyclopropane diastereomers as a clear and colorless oil (9.3 mg, 40 µmol, 42%, 1.7:1 dr). The dr was determined by integration of the alkene proton resonances of each cyclopropane diastereomer in the $^{1}$H NMR spectrum. The relative configuration of cyclopropane was assigned based on analogy to cyclopropane **14**. **TLC R$_f$** = 0.2 (10% EtOAc/hexanes); **HRMS** (TOF MS CI+) *m/z*: [M+H]$^+$ calculated for $C_{13}H_{21}FO_2H$, 229.1604; found 229.1595. For clarity, the $^{1}$H NMR, $^{13}$C NMR, and $^{19}$F NMR data of the major and minor diastereomers have been tabulated individually.

**Major Diastereomer: $^{1}$H NMR** (500 MHz, $CDCl_3$) δ 6.00–5.86 (m, 1H), 4.36–4.19 (m, 2H), 1.63–1.57 (m, 1H), 1.51–1.25 (m, 11H), 1.23–1.13 (m, 1H), 0.95 (t, *J* = 6.5 Hz, 3H), 0.88–0.78 (m, 2H); **$^{13}$C NMR** (125.7 MHz, $CDCl_3$) δ 161.0 (d, *J* = 33.8), 147.5 (d, *J* = 251.1), 126.1 (d, *J* = 10.6), 119.9, 117.5, 61.3, 33.6, 31.6, 29.7, 28.9, 22.7, 15.6, 14.2. **$^{19}$F NMR** (376.5, $CDCl_3$) δ -137.8 (d, *J* = 32.1, 1F).

**Minor Diastereomer: $^{1}$H NMR** (500 MHz, $CDCl_3$) δ 5.68 (dd, *J* = 32.0, 10.6 Hz, 1H), 4.36–4.19 (m, 2H), 1.97–1.88 (m, 1H), 1.51–1.25 (m, 11H), 1.23–1.13 (m, 1H), 1.09–1.00 (m, 1H), 0.95 (t, *J* = 6.5 Hz, 3H), 0.52–0.47 (m, 1H); **$^{13}$C NMR** (125.7 MHz, $CDCl_3$) δ 160.8 (d, *J* = 33.8), 148.7 (d, *J* = 253.4), 122.9 (d, *J* = 9.7), 119.9, 117.5, 61.3, 33.6, 31.6, 29.7, 28.9, 22.8, 15.6, 14.1; **$^{19}$F NMR** (376.5, $CDCl_3$) δ -137.8 (d, *J* = 31.0, 1F).

2) <u>Enantioenriched Cyclopropanes</u>

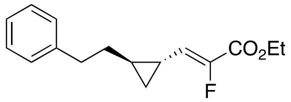

**Cyclopropane** (–)-(*R*,*R*)-**3** was prepared according to Method A. The following amounts of reagents were used: enantioenriched mesylate **SI-12** (32 mg, 84 μmol, 1.0 equiv), $Ni(PMe_3)_2Cl_2$ (1.2 mg, 4.2 μmol, 5.0 mol %), $Zn^0$ (27 mg, 0.41 mmol, 4.9 equiv), NaBr (17 mg, 0.17 mmol, 2.0 equiv), and anhydrous MeCN (1.0 mL, 0.084 M in substrate). The residue was then purified by flash column chromatography (0–10% EtOAc/hexanes) to afford the title compound as a mixture of cyclopropane diastereomers as a pale-yellow oil (12 mg, 45 μmol, 53%, 1:1 dr, 96% ee). Refer to cyclopropane **3** above for analytical data. $[\alpha]^{23}_D$ –40.0 (c 1.05 mg/mL $CHCl_3$); **SFC Analysis** (Chiralcel OD-H, 1% IPA/$CO_2$, 2.0 mL/min, 230 nm) indicated 90% ee: $t_R$ (minor diastereomer, both enantiomers) = 9.9 minutes, $t_R$ (major diastereomer, major enantiomer) = 11.0 minutes, $t_R$ (major diastereomer, minor enantiomer) = 13.3 minutes.

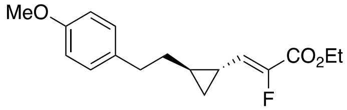

**Cyclopropane** (–)-(*R*,*R*)-**4** was prepared according to Method A. The following amounts of reagents were used: enantioenriched mesylate **SI-13** (39 mg, 97 μmol, 1.0 equiv), $Ni(PMe_3)_2Cl_2$ (1.4 mg, 4.8 μmol, 5.0 mol %), $Zn^0$ (13 mg, 0.19 mmol, 2.0 equiv), NaBr (20. mg, 0.19 mmol, 2.0 equiv), and anhydrous MeCN (0.97 mL, 0.10 M in substrate). The residue was then purified by flash column chromatography (0–10% EtOAc/hexanes) to afford the title compound as a mixture of cyclopropane diastereomers as a pale-yellow oil (14 mg, 48 μmol, 49%, 1:1 dr, 91% ee). Refer to cyclopropane **4** above for analytical data. $[\alpha]^{23}_D$ –36.5° (c 10.8 mg/1.5 mL $CHCl_3$); **SFC Analysis** (Chiralcel OD-H, 0.1% IPA, 2.0 mL/min, 230 nm) indicated 91% ee: $t_R$ (minor diastereomer, both enantiomers) = 43.8 minutes, $t_R$ (major diastereomer, major enantiomer) = 49.2 minutes, $t_R$ (major diastereomer, minor enantiomer) = 62.4 minutes.

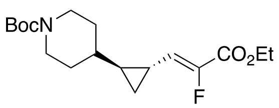

**Cyclopropane** (–)-**9** was prepared according to Method A. The following amounts of reagents were used: enantioenriched mesylate **SI-18** (49 mg, 0.11 mmol, 1.0 equiv), $Ni(PMe_3)_2Cl_2$ (1.5 mg, 5.4 μmol, 5.0 mol %), $Zn^0$ (14 mg, 0.22 mmol, 2.0 equiv), NaBr (22 mg, 0.37 mmol, 2.0 equiv), and anhydrous MeCN (1.1 mL, 0.10 M in substrate). The residue was then purified by flash column chromatography (0–10% EtOAc/hexanes) to afford the title compound as a mixture of cyclopropane diastereomers as a pale-yellow oil (11 mg, 32 μmol, 30%, 3:1 dr, 90% ee). Refer to cyclopropane **9** above for analytical data. $[\alpha]^{25}_D$ –6.4° (c 9.4 mg/1.3 mL $CHCl_3$); **SFC Analysis** (Chiralcel OD-H, 2% IPA, 1.5 mL/min, 230 nm) indicated 90% ee: $t_R$ (minor diastereomer, both enantiomers) = 18.3 minutes, $t_R$ (major diastereomer, minor enantiomer) = 22.5 minutes, $t_R$ (major diastereomer, major enantiomer) = 27.7 minutes.

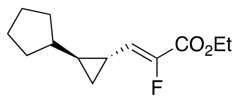

**Cyclopropane** (–)-**11** was prepared according to Method A. The following amounts of reagents were used: enantioenriched mesylate **SI-20** (30. mg, 0.089 mmol, 1.0 equiv), $Ni(PMe_3)_2Cl_2$ (1.1 mg, 4.0 µmol, 5.0 mol %), $Zn^0$ (12 mg, 0.18 mmol, 2.0 equiv), NaBr (19 mg, 0.18 mmol, 2.0 equiv), and anhydrous MeCN (1.0 mL, 0.089 M in substrate). The residue was then purified by flash column chromatography (0–10% EtOAc/hexanes) to afford the title compound as a mixture of cyclopropane diastereomers as a pale-yellow oil (10. mg, 45 µmol, 51%, 2:1 dr, 86% ee). Refer to cyclopropane **11** above for analytical data. $[\alpha]^{23}_{D}$–41.9 (*c* 0.43 mg/mL $CHCl_3$); **HPLC Analysis** (Chiralcel OJ, 1.0% IPA/hexanes, 2.0 mL/min, 230 nm) indicated 86% ee: $t_R$ (minor diastereomer, both enantiomers) = 3.1 minutes, $t_R$ (major diastereomer, minor enantiomer) = 3.7 minutes, $t_R$ (major diastereomer, major enantiomer) = 3.9 minutes.

## E. General Procedures for Starting Material Synthesis:

Method B: Copper-catalyzed Alkene Functionalization

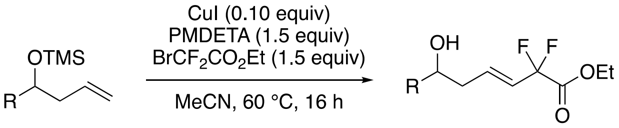


This method was adapted from a procedure reported by Wang.[xii] In a glovebox, a flame dried Schlenk tube equipped with a stir bar was charged with CuI (10. mol %). The tube was capped and removed from the glovebox and placed under $N_2$. Then, MeCN (0.20 M in substrate), silylated alcohol (1.0 equiv, 0.10 M in MeCN), PMDETA (1.5 equiv), and ethyl bromodifluoroacetate (1.5 equiv) were added. The reaction was heated to 60 °C and stirred for 16 h. The reaction mixture was allowed to cool to rt before being transferred to a round-bottom flask and concentrated in vacuo.

Method C: Mesylation of Alcohol

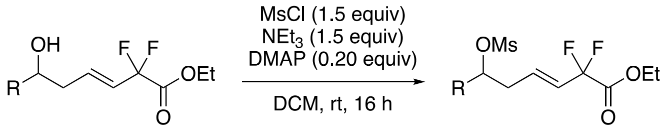


To a flame-dried round-bottom flask equipped with a stir bar was added alcohol (1.0 equiv) and DCM (0.20 M in substrate) under $N_2$. Then, $Et_3N$ (1.5 equiv), DMAP (0.20 equiv), and MsCl (1.5 equiv) were added, and the reaction mixture was allowed to stir at rt for 16 h. Saturated $NaHCO_3$ (aq) was added to quench, and the reaction mixture was extracted with DCM (3 x 20 mL). The combined organic layers were washed with brine, dried over $Na_2SO_4$, and concentrated in vacuo.

Method D: Triphenylsilylation of Alcohol

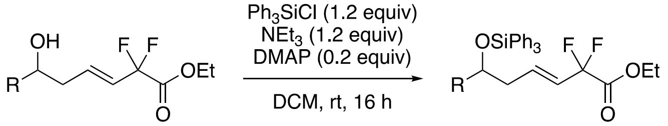


This method was adapted from a procedure reported by Zipse.[xiii] To a flame-dried round-bottom flask equipped with a stir bar was added alcohol (1.0 equiv) and DCM (0.20 M in substrate) under $N_2$. Then, $Et_3N$ (1.2 equiv), DMAP (0.20 equiv), and $Ph_3SiCl$ (1.2 equiv) were added, and the reaction mixture was allowed to stir at rt for 16 h. Saturated $NaHCO_3$ (aq) was added to quench, and the reaction mixture was extracted with DCM (3 x 20 mL). The combined organic layers were washed with brine, dried over $Na_2SO_4$, and concentrated in vacuo.

**F. Synthesis and Characterization Data of Intermediates and Mesylates:**

1) Intermediates and Mesylates for Racemic Cyclopropanes

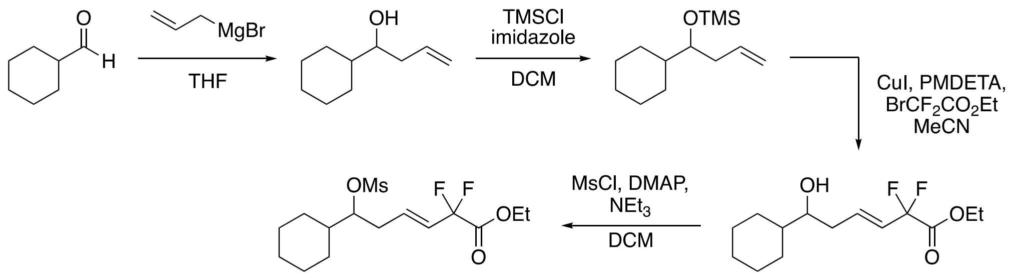


**Scheme SI-5:** Synthesis of Mesylate **1a**

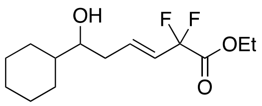

**Alcohol (SI-26)** was prepared according to Method B. The following amounts of reagents were used: silylated alcohol (0.60 g, 2.6 mmol, 1.0 equiv), CuI (50. mg, 0.26 mmol, 10. mol %), PMDETA (0.83 mL, 4.0 mmol, 1.5 equiv), ethyl bromodifluoroacetate (0.51 mL, 4.0 mmol, 1.5 equiv), and MeCN (13 mL, 0.20 M in substrate). The compound was purified by flash column chromatography (0–20% EtOAc/hexanes) to afford the title compound as a mixture of alkene diastereomers as a yellow oil (0.27 g, 0.96 mmol, 37%, 2.4:1 dr). The dr was determined by integration of one of the alkene proton resonances in the $^1$H NMR spectrum. **TLC R$_f$** = 0.3 (20% EtOAc/hexanes, CAM stain); **HRMS** (TOF MS ES+) *m/z*: $[M + Na]^+$ calculated for $C_{14}H_{22}F_2O_3Na$, 299.1435; found 299.1444. For clarity, the $^1$H NMR data of the major and minor diastereomers have been tabulated individually.

**Major diastereomer: $^1$H NMR** (400 MHz, $CDCl_3$) δ 6.39–6.30 (m, 1H), 5.83–5.66 (m, 1H), 4.32 (q, *J* = 7.1 Hz, 2H), 3.49–3.40 (m, 1H), 2.57–2.22 (m, 2H), 1.86–1.74 (m, 3H), 1.71–1.64 (m, 2H), 1.54 (d, *J* = 4.6 Hz, 1H), 1.34 (t, *J* = 8.5 Hz, 3H), 1.27–0.97 (m, 6H).

**Minor diastereomer: $^1$H NMR** (400 MHz, $CDCl_3$) δ 6.15–6.07 (m, 1H), 5.83–5.66 (m, 1H), 4.32 (q, *J* = 7.1 Hz, 2H), 3.49–3.40 (m, 1H), 2.57–2.22 (m, 2H), 1.86–1.74 (m, 3H), 1.71–1.64 (m, 2H), 1.50 (d, *J* = 5.5 Hz, 1H), 1.34 (t, *J* = 8.5 Hz, 3H), 1.27–0.97 (m, 6H).

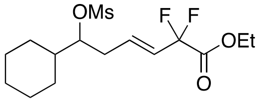

**Mesylate (1a)** was prepared according to Method C. The following amounts of reagents were used: alcohol **SI-26** (0.27 g, 0.96 mmol, 1.0 equiv), $Et_3N$ (0.20 mL, 1.5 mmol, 1.5 equiv), DMAP (12 mg, 96 µmol, 0.10 equiv), MsCl (0.11 mL, 1.5 mmol, 1.5 equiv), and DCM (4.8 mL, 0.20 M in substrate). The compound was purified by flash column chromatography (0–20% EtOAc/hexanes) to afford the title compound as a mixture of alkene diastereomers as a yellow oil (0.32 g, 0.90 mmol, 94%, 3.5:1 dr). The dr was determined by integration of one of the alkene proton resonances in the $^1$H NMR spectrum. **TLC $R_f$** = 0.3 (20% EtOAc/hexanes, CAM stain); **HRMS** (TOF MS ES+) *m/z*: $[M + Na]^+$ calculated for $C_{15}H_{24}F_2O_5SNa$, 377.1210; found 377.1220. For clarity, the $^1$H NMR, $^{13}$C NMR, and $^{19}$F NMR data of the major and minor diastereomers have been tabulated individually.

**Major diastereomer: $^1$H NMR** (400 MHz, $CDCl_3$) δ 6.32–6.23 (m, 1H), 5.88–5.71 (m, 1H), 4.59 (aquint, *J* = 5.7 Hz, 1H), 4.33 (q, *J* = 7.1 Hz, 2H), 2.99 (s, 3H), 2.81–2.73 (m, 1H), 2.65–2.51 (m, 1H), 1.83–1.59 (m, 5H), 1.35 (t, *J* = 7.2 Hz, 3H), 1.31–1.00 (m, 6H); **$^{13}$C NMR** (150.9 MHz, $CDCl_3$) δ 163.7 (t, *J* = 34.3 Hz), 134.2 (t, *J* = 9.3 Hz), 124.9 (t, *J* = 25.2 Hz), 112.0 (t, *J* = 248.1 Hz), 85.4, 63.2, 41.2, 38.70, 34.4, 28.5, 28.2, 26.1, 25.9, 25.8, 14.0; **$^{19}$F NMR** (376.5 MHz, $CDCl_3$) δ -103.6 (dd, *J* = 44.7, 10.3 Hz, 2F).

**Minor diastereomer: $^1$H NMR** (400 MHz, $CDCl_3$) δ 6.10–6.03 (m, 1H), 5.88–5.71 (m, 1H), 4.59 (aquint, *J* = 5.7 Hz, 1H), 4.33 (q, *J* = 7.1 Hz, 2H), 3.01 (s, 3H), 2.81–2.73 (m, 1H), 2.65–2.51 (m, 1H), 1.83–1.59 (m, 5H), 1.35 (t, *J* = 7.2 Hz, 3H), 1.31–1.00 (m, 6H); **$^{13}$C NMR** (150.9 MHz, $CDCl_3$) δ 163.9 (t, *J* = 34.4 Hz), 136.3 (t, *J* = 6.8 Hz), 123.7 (t, *J* = 26.2 Hz), 112.7 (t, *J* = 249.4 Hz), 86.0, 63.3, 41.6, 38.75, 34.4, 28.5, 28.2, 26.1, 25.9, 25.8, 14.0; **$^{19}$F NMR** (376.5 MHz, $CDCl_3$) δ -99.7 (d, *J* = 18.4 Hz, 2F).

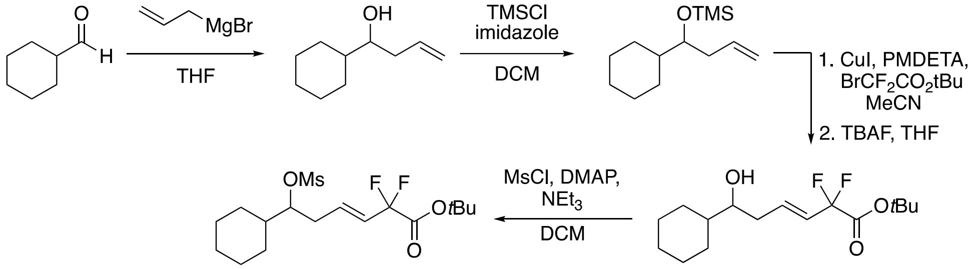


**Scheme SI-6:** Synthesis of Mesylate **1b**

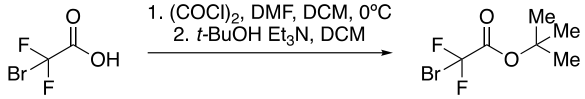


**Ester (SI-27)** was prepared with modificiations to a procedure by You.[xiv] 2-bromo-2,2-difluoroacetic acid (1.8 g, 10. mmol, 1.0 equiv) was dissolved in DCM (15 mL, 0.67 M in substrate) in a flame-dried round bottom flask with stir bar. DMF (2 drops) and oxalyl chloride (0.94 mL, 11 mmol, 1.1 equiv) were added consecutively and allowed to stir overnight. The solution was concentrated under vacuum, diluted in DCM (30. mL, 0.30 M in substrate) in a separate flame-dried flask with stir bar. The solution was cooled to 0 ºC. *t*–BuOH (2.0 mL, 20. mmol, 2.0 equiv)

and $Et_3N$ (1.5 mL, 11 mmol, 1.1 equiv) were added and allowed to stir overnight. This solution was diluted with water, extracted with DCM, washed with $NaHCO_3$, and dried with $MgSO_4$ before concentrating in vacuo. The residue was then filtered through a short plug of silica (100% pentanes) to afford the title compound as a colorless oil (1.2 g, 5.4 mmol, 54%). **$^1$H NMR** (500 MHz, $CDCl_3$) δ 1.63 (s, 9H); **$^{13}$C NMR** (125.8 MHz, $CDCl_3$) δ 158.3 (t, *J* = 30.5 Hz), 109.3 (t, *J* = 315.8 Hz), 86.8, 27.5 (3C); **$^{19}$F NMR** (376.5 MHz, $CDCl_3$) δ -60.9 (s, 2F).

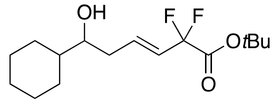


**Alcohol (SI-28)** was prepared according to Method B. The following amounts of reagents were used: silylated alcohol (0.45 g, 2.0 mmol, 1.0 equiv), CuI (38 mg, 0.20 mmol, 10. mol %), PMDETA (0.63 mL, 3.0 mmol, 1.5 equiv), ester **SI-26** (0.69 g, 3.0 mmol, 1.5 equiv), and MeCN (10. mL, 0.20 M in substrate). The compound was purified by flash column chromatography (0–40% EtOAc/hexanes) to afford the title compound as a 4:1 mixture of alkene diastereomers as a yellow oil (33 mg, 0.11 mmol, 5.4%). **TLC $R_f$** = 0.3 (20% EtOAc/hexanes); For clarity, the $^1$H NMR data of the major and minor diastereomers have been tabulated individually.

**Major diastereomer: $^1$H NMR** (500 MHz, $CDCl_3$) δ 6.42–6.32 (m, 1H), 5.89–5.69 (m, 1H), 3.52 (br s, 1H), 2.62–2.26 (m, 2H), 1.94–1.66 (m, 5H), 1.58 (s, 9H), 1.47–1.01 (m, 7H).

**Minor diastereomer: $^1$H NMR** (500 MHz, $CDCl_3$) δ 6.19–6.09 (m, 1 H), 5.89–5.69 (m, 1H), 3.52 (br s, 1H), 2.62–2.26 (m, 2H), 1.94–1.66 (m, 5H), 1.58 (s, 9H), 1.47–1.01 (m, 7H).

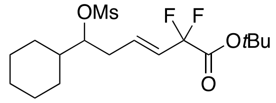


**Mesylate (1b)** was prepared according to Method C. The following amounts of reagents were used: **SI-28** (57 mg, 0.19 mmol, 1.0 equiv), $Et_3N$ (50. µL, 0.38 mmol, 2.0 equiv), DMAP (5.0 mg, 40. µmol, 0.20 equiv), MsCl (20. µL, 0.23 mmol, 1.2 equiv), and DCM (1.0 mL, 0.19 M in substrate). The compound was purified by flash column chromatography (0–20% EtOAc/hexanes) to afford the title compound as a 4:1 mixture of alkene diastereomers as a yellow oil (32 mg, 85 µmol, 44%). **TLC $R_f$** = 0.3 (20% EtOAc/hexanes); **HRMS** (TOF MS ES+) *m/z*: $[M + Na]^+$ calculated for $C_{17}H_{28}F_2O_5SNa$, 405.1523; found 405.1525. For clarity, the $^1$H NMR, $^{13}$C NMR, and $^{19}$F NMR data of the major and minor diastereomers have been tabulated individually.

**Major diastereomer: $^1$H NMR** (600 MHz, $CDCl_3$) δ 6.34–6.25 (m, 1H), 5.90–5.75 (m, 1H), 4.67–4.59 (m, 1H), 3.03 (s, 3H), 2.82–2.57 (m, 2H), 1.91–1.80 (m, 3H), 1.79–1.69 (m, 2H), 1.58 (s, 9H), 1.32–1.24 (m, 2H), 1.22–1.05 (m, 4H); **$^{13}$C NMR** (150.9 MHz, $CDCl_3$) δ 162.7 (t, *J* = 34.3 Hz), 133.5 (t, *J* = 9.4 Hz), 125.3 (t, *J* = 25.4 Hz), 111.7 (t, *J* = 248.0 Hz), 84.9, 85.4, 41.0, 38.7, 34.4, 28.5, 28.2, 27.8 (3C), 26.1, 25.9, 25.8; **$^{19}$F NMR** (376.5 MHz, $CDCl_3$) δ -103.5 (d, *J* = 10.3 Hz, 1F), -103.7 (d, *J* = 10.3 Hz, 1F).

**Minor diastereomer: $^1$H NMR** (600 MHz, $CDCl_3$) δ 6.11–6.05 (m, 1H), 5.90–5.75 (m, 1H), 4.67–4.59 (m, 1H), 3.03 (s, 3H), 2.82–2.57 (m, 2H), 1.91–1.80 (m, 3H), 1.79–1.69 (m, 2H), 1.58 (s, 9H), 1.32–1.24 (m, 2H), 1.22–1.05 (m, 4H); **$^{13}$C NMR** (150.9 MHz, $CDCl_3$) δ 162.7 (t, *J* = 34.3 Hz), 135.8 (t, *J* = 6.5 Hz), 124.0 (t, *J* = 26.0 Hz), 112.6 (t, *J* = 250.0 Hz), 86.2, 84.9, 41.5, 38.8, 34.4,

28.4, 28.2, 27.7 (3C), 26.1, 25.9 (2C); **$^{19}$F NMR** (376.5 MHz, $CDCl_3$) δ -100.0 (at, *J* = 12.6 Hz, 2F).

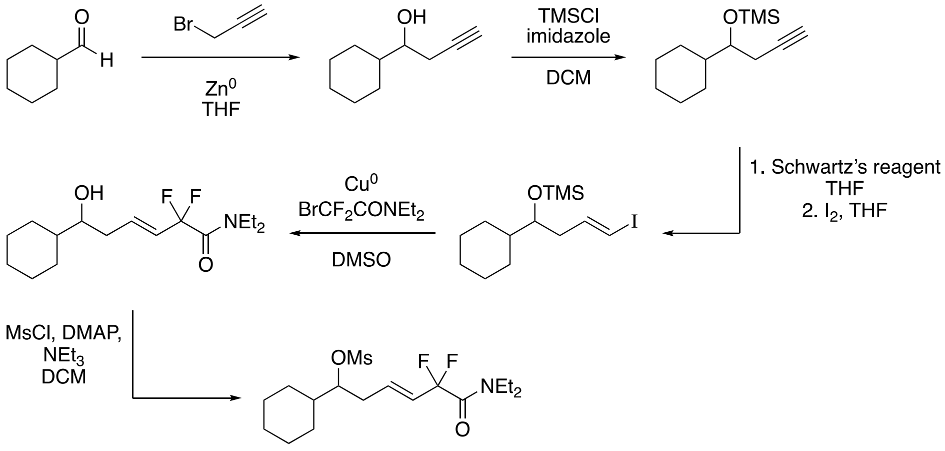


**Scheme SI-7:** Synthesis of Mesylate **1c**

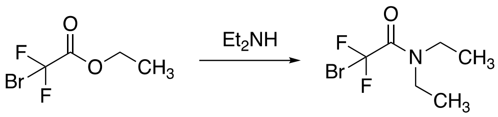


**Amide (SI-29)** was prepared following a procedure reported by Skrydstrup.[xv] To a round-bottom flask equipped with a stir bar was added ethyl bromodifluoroacetate (0.90 mL, 7.0 mmol, 1.0 equiv) followed by diethylamine (0.87 mL, 8.4 mmol, 1.2 equiv). The reaction mixture was allowed to stir at rt for 16 h. After 16 h, the reaction mixture was diluted with $Et_2O$ and washed with 1 M HCl, saturated $NaHCO_3$ (aq), and then brine. The combined organic layers were dried over $Na_2SO_4$ and concentrated in vacuo. The residue was purified by flash column chromatography (0–10% EtOAc/hexanes) to afford the title compound as a colorless oil (1.3 g, 5.7 mmol, 81%). **TLC $R_f$** = 0.3 (10% EtOAc/hexanes); **$^1$H NMR** (400 MHz, $CDCl_3$) δ 3.52 (q, *J* = 7.1 Hz, 2H), 3.43 (q, *J* = 7.1 Hz, 2H), 1.25 (t, *J* = 7.1 Hz, 3H), 1.19 (t, *J* = 7.1 Hz, 3H). Analytical data is consistent with literature values.[15]

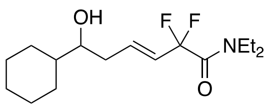

**Alcohol (SI-30)** was prepared according to a procedure reported by Hu.[xvi] To a flame-dried Schlenk flask equipped with a stir bar was added silylated alcohol (0.18 g, 0.50 mmol, 1.0 equiv), Cu$^0$ (190 mg, 3.0 mmol, 6.0 equiv), amide **SI-29** (0.17 g, 0.75 mmol, 1.5 equiv), and DMSO (2.0 mL, 0.25 M in substrate).The reaction mixture was heated to 55 °C and allowed to stir for 16 h. After 16 h, the reaction mixture was allowed to cool to rt before quenching with DI $H_2O$. The biphasic mixture was extracted with $Et_2O$, washed with $H_2O$ and dried over $Na_2SO_4$. The residue was purified by flash column chromatography (0–20% EtOAc/hexanes) to afford the title compound as a pale-yellow oil as a single alkene diastereomer (83 mg, 0.27 mmol, 55%). **TLC $R_f$** = 0.3 (20% EtOAc/hexanes, CAM stain); **$^1$H NMR** (400 MHz, $CDCl_3$) δ 6.25–6.18 (m, 1H), 5.89 (dt, *J* = 15.9, 10.5 Hz, 1H), 3.49–3.37 (m, 5H), 2.41–2.35 (m, 1H), 2.28–2.21 (m, 1H), 2.16 (br s, 1H), 1.85 (ad, *J* = 12.8 Hz, 1H), 1.78–1.75 (m, 2H), 1.67 (ad, *J* = 11.7 Hz, 2H), 1.39–1.23 (m, 3H),

1.18 (dt, $J$ = 15.8, 7.2 Hz, 6H), 1.12–0.96 (m, 3H); **HRMS** (TOF MS ES+) $m/z$: $[M + H]^+$ calculated for $C_{16}H_{27}F_2NO_2H$, 304.2088; found 304.2085.

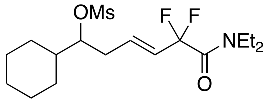


**Mesylate (1c)** was prepared according to Method C. The following amounts of reagents were used: alcohol **SI-30** (69 mg, 0.23 mmol, 1.0 equiv), $Et_3N$ (50. µL, 0.34 mmol, 1.5 equiv), DMAP (2.8 mg, 23 µmol, 0.10 equiv), MsCl (30. µL, 0.34 mmol, 1.5 equiv), and DCM (1.2 mL, 0.20 M in substrate). The compound was purified by flash column chromatography (0–30% EtOAc/hexanes) to afford the title compound as a single alkene diastereomer as a pale-yellow oil (77 mg, 0.20 mmol, 89%). **TLC $R_f$** = 0.4 (25% EtOAc/hexanes, CAM stain); **$^1$H NMR** (500 MHz, $CDCl_3$) δ 6.21–6.15 (m, 1H), 5.97 (dt, $J$ = 15.8, 10.5 Hz, 1H), 4.58 (aq, $J$ = 5.8 Hz, 1H), 3.46 (q, $J$ = 7.0 Hz, 2H), 3.39 (q, $J$ = 7.1 Hz, 2H), 3.01 (s, 3H), 2.59–2.56 (m, 2H), 1.84–1.62 (m, 6H), 1.29–1.24 (m, 2H), 1.21 (t, $J$ = 7.1 Hz, 3H), 1.16 (t, $J$ = 7.1 Hz, 3H), 1.14–1.02 (m, 3H); **$^{13}$C NMR** (125.8 MHz, $CDCl_3$) δ 162.6 (t, $J$ = 29.8 Hz), 132.1 (t, $J$ = 9.6 Hz), 126.5 (t, $J$ = 24.8 Hz), 114.6 (t, $J$ = 249.6 Hz), 85.6, 42.1 (t, J = 5.1 Hz), 41.5, 41.3, 38.8, 34.4, 28.5, 28.3, 26.1, 25.9, 25.8, 14.3, 12.4; **$^{19}$F NMR** (376.5 MHz, $CDCl_3$) δ -96.7 (dd, $J$ = 44.4, 9.9 Hz, 2F); **HRMS** (TOF MS ES+) $m/z$: $[M + Na]^+$ calculated for $C_{17}H_{29}F_2NO_4SNa$, 404.1683; found 404.1678.

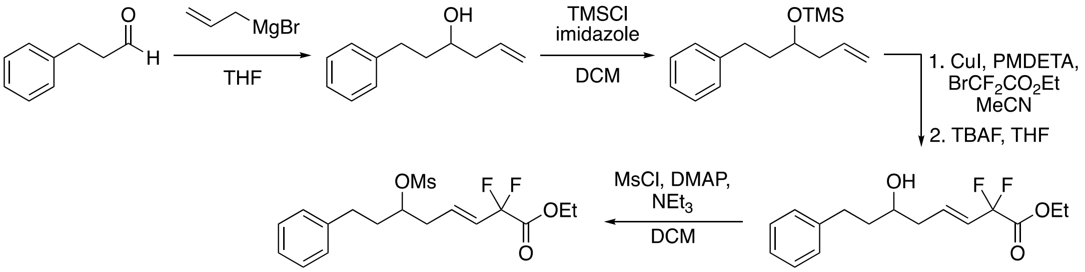


**Scheme SI-8:** Synthesis of Mesylate **SI-12**

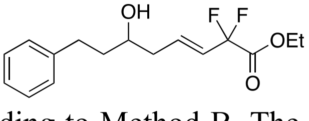


**Alcohol (SI-31)** was prepared according to Method B. The following amounts of reagents were used: silylated alcohol (1.9 g, 7.5 mmol, 1.0 equiv), CuI (0.14 g, 0.75 mmol, 0.10 equiv), PMDETA (2.3 mL, 11 mmol, 1.5 equiv), ethyl bromodifluoroacetate (1.4 mL, 11 mmol, 1.5 equiv), and MeCN (15 mL, 0.50 M in substrate). The compound was purified by flash column chromatography (0–5% EtOAc/hexanes) to afford the title compound as a mixture of alkene diastereomers as a colorless oil (1.1 g, 2.9 mmol, 39%, 1.5:1 dr). The silyl protected alcohol was then subjected to a TBAF deprotection. To a flame-dried round-bottom flask equipped with a stir bar was added the silyl-protected alcohol (1.1 g, 2.9 mmol, 1.0 equiv), TBAF (3.2 mL, 3.2 mmol, 1.1 equiv, 1.0 M in THF), and THF (5.8 mL, 0.50 M in substrate). The compound was purified by flash column chromatography (0–15% EtOAc/hexanes) to afford the title compound as a mixture of alkene diastereomers as a colorless oil (0.65 g, 2.2 mmol, 75%, 2.3:1 dr). **TLC $R_f$** = 0.5 (5% EtOAc/hexanes); **SFC Analysis** (Chiralcel OD-H, 4.0% IPA/$CO_2$, 2.0 mL/min, 210 nm) indicated 0% ee: $t_R$ (minor diastereomer, major enantiomer) = 13.5 minutes, $t_R$ (major diastereomer, major enantiomer) = 15.3 minutes, $t_R$ (minor diastereomer, minor enantiomer) = 20.6 minutes, $t_R$ (major

diastereomer, minor enantiomer) = 22.0 minutes. For clarity, the $^{1}$H NMR data of the major and minor diastereomers have been tabulated individually.

**Major Diastereomer: $^{1}$H NMR** (400 MHz) $CDCl_3$ δ 7.31–7.26 (m, 2H), 7.22–7.16 (m, 3H), 6.31 (dtt, *J* = 16.0, 7.3, 2.6 Hz, 1H), 5.84–5.66 (m, 1H), 4.31 (q, *J* = 7.1 Hz, 2H), 3.79–2.68 (m, 1H), 2.84–2.75 (m, 1H), 2.73–2.64 (m, 1H), 2.55–2.48 (m, 1H), 2.42–2.25 (m, 1H), 1.83–1.75 (m, 2H), 1.53 (d, *J* = 4.9 Hz, 1H), 1.33 (t, *J* = 7.1 Hz, 3H).

**Minor Diastereomer: $^{1}$H NMR** (400 MHz) $CDCl_3$ δ 7.31–7.26 (m, 2H), 7.22–7.16 (m, 3H), 6.07 (dtt, *J* = 11.7, 7.1, 1.9 Hz, 1H), 5.84–5.66 (m, 1H), 4.29 (q, *J* = 7.1 Hz, 2H), 3.79–2.68 (m, 1H), 2.84–2.75 (m, 1H), 2.73–2.64 (m, 1H), 2.42–2.25 (m, 2H), 1.83–1.75 (m, 2H), 1.50 (d, *J* = 5.5 Hz, 1H), 1.32 (t, *J* = 7.1 Hz, 3H).

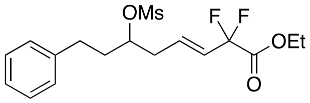


**Mesylate (SI-12)** was prepared according to Method C. The following amounts of reagents were used: alcohol **SI-31** (0.35 g, 1.2 mmol, 1.0 equiv), MsCl (0.11 mL, 1.4 mmol, 1.2 equiv), DMAP (14 mg, 0.12 mmol, 0.10 equiv), $Et_3N$ (0.24 mL, 1.8 mmol, 1.5 equiv), and DCM (5.9 mL, 0.20 M in substrate). The compound was purified by flash column chromatography (0–25% EtOAc/hex) to afford the title compound as a mixture of alkene diastereomers as a colorless oil (0.39 g, 1.0 mmol, 88%, 2.3:1 dr). **TLC $R_f$** = 0.3 (15% EtOAc/hexanes); **HRMS** (TOF MS ES+) *m/z*: $[M + Na]^+$ calculated for $C_{17}H_{22}F_2O_5SNa$, 399.1054; found 399.1044. For clarity, the $^{1}$H NMR, $^{13}$C NMR, and $^{19}$F NMR data of the major and minor diastereomers have been tabulated individually.

**Major Diastereomer: $^{1}$H NMR** (500 MHz, $CDCl_3$) δ 7.32–7.26 (m, 2H), 7.23–7.16 (m, 3H), 6.37–6.22 (m, 1H), 5.88–5.73 (m, 1H), 4.82 (quint, *J* = 5.7 Hz, 1H), 4.34–4.27 (m, 2H), 2.99 (s, 3H), 2.85–2.52 (m, 4H), 2.11–1.92 (m, 2H), 1.33 (t, *J* = 7.1 Hz, 3H); **$^{13}$C NMR** (125.7 MHz, $CDCl_3$) δ 163.7 (t, *J* = 34.5 Hz), 140.4, 133.4 (t, *J* = 9.3 Hz), 128.73 (2C), 128.4 (2C), 126.5, 125.4 (t, *J* = 25.7 Hz), 111.9 (t, *J* = 247.8 Hz), 80.4, 63.3, 38.8, 37.3, 26.1, 31.3, 14.0; **$^{19}$F NMR** (564.6 MHz, $CDCl_3$) δ -103.4 (d, *J* = 10.9 Hz, 1F), -103.5 (d, *J* = 10.9 Hz, 1F).

**Minor Diastereomer: $^{1}$H NMR** (500 MHz, $CDCl_3$) δ 7.32–7.26 (m, 2H), 7.23–7.16 (m, 3H), 6.07–5.99 (m, 1H), 5.88–5.73 (m, 1H), 4.82 (quint, *J* = 5.7 Hz, 1H), 4.34–4.27 (m, 2H), 3.00 (s, 3H), 2.85–2.52 (m, 4H), 2.11–1.92 (m, 2H), 1.32 (t, *J* = 7.0 Hz, 3H); **$^{13}$C NMR** (125.7 MHz, $CDCl_3$) δ 163.9 (t, *J* = 34.2 Hz), 140.6, 135.3 (t, *J* = 6.9 Hz), 128.69 (2C), 128.4 (2C), 126.4, 124.3 (t, *J* = 26.2 Hz), 112.7 (t, *J* = 249.7 Hz), 81.0, 63.4, 38.8, 37.3, 36.2, 33.5, 14.0; **$^{19}$F NMR** (564.6 MHz, $CDCl_3$) δ -103.4 (d, *J* = 10.9 Hz, 1F), -103.5 (d, *J* = 10.9 Hz, 1F).

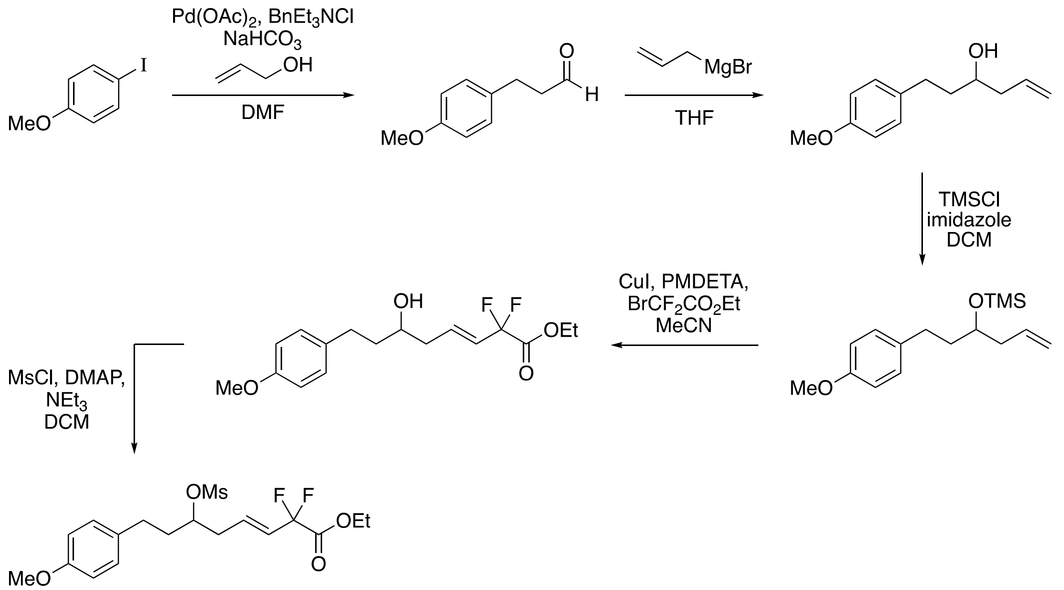

**Scheme SI-9:** Synthesis of Mesylate **SI-13**

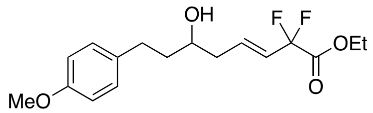

**Alcohol (SI-32)** was prepared according to Method B. The following amounts of reagents were used: silylated alcohol (1.6 g, 5.8 mmol, 1.0 equiv), CuI (110 mg, 0.58 mmol, 10. mol %), PMDETA (1.8 mL, 8.7 mmol, 1.5 equiv), ethyl bromodifluoroacetate (1.1 mL, 8.7 mmol, 1.5 equiv), and MeCN (29 mL, 0.20 M in substrate).The compound was purified by flash column chromatography (0–20% EtOAc/hexanes) to afford the title compound as a mixture of alkene diastereomers as a yellow oil with EtOAc (0.25 g, 0.76 mmol, 13%, 1.3% EtOAc by NMR, 2.3:1 dr). **TLC** $\mathbf{R_f}$ = 0.4 (25% EtOAc/hexanes, CAM stain). For clarity, the $^1$H NMR data of the major and minor diastereomers have been tabulated individually.

**Major Diastereomer: $^1$H NMR** (400 MHz, $CDCl_3$) δ 7.10 (d, *J* = 8.6 Hz, 2H), 6.83 (d, *J* = 8.6 Hz, 2H), 6.35–6.27 (m, 1H), 5.82–5.67 (m, 1H), 4.31 (q, *J* = 7.1 Hz, 2H), 3.79 (s, 3H), 3.75–3.69 (m, 1H), 2.77–2.59 (m, 2H), 2.54–2.48 (m, 1H), 2.41–2.25 (m, 1H), 1.76 (q, *J* = 7.9 Hz, 2H), 1.51 (d, *J* = 4.8 Hz, 1H), 1.33 (t, *J* = 7.1 Hz, 3H).

**Minor Diastereomer: $^1$H NMR** (400 MHz, $CDCl_3$) δ 7.11 (d, *J* = 9.1 Hz, 2H), 6.83 (d, *J* = 8.6 Hz, 2H), 6.10–6.03 (m, 1H), 5.82–5.67 (m, 1H), 4.31 (q, *J* = 7.1 Hz, 2H), 3.79 (s, 3H), 3.75–3.69 (m, 1H), 2.77–2.59 (m, 2H), 2.54–2.48 (m, 1H), 2.41–2.25 (m, 1H), 1.76 (q, *J* = 7.9 Hz, 2H), 1.48 (d, *J* = 5.4 Hz, 1H), 1.32 (t, *J* = 7.1 Hz, 3H).

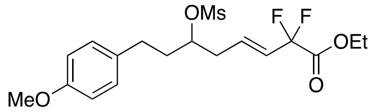

**Mesylate (SI-13)** was prepared according to Method C. The following amounts of reagents were used: alcohol **SI-32** (0.25 g, 0.76 mmol, 1.0 equiv), $Et_3N$ (0.16 mL, 1.2 mmol, 1.5 equiv), DMAP (9.3 mg, 76 μmol, 0.10 equiv), MsCl (90. μL, 1.2 mmol, 1.5 equiv), and DCM (3.8 mL, 0.20 M in

substrate). The compound was purified by flash column chromatography (0–25% EtOAc/hexanes) to afford the title compound as a mixture of alkene diastereomers as a yellow oil (0.26 g, 0.63 mmol, 83%, 2.5:1 dr). **TLC R$_f$** = 0.2 (25% EtOAc/hexanes, CAM stain); **HRMS** (TOF MS ES+) *m/z*: $[M + NH_4]^+$ calculated for $C_{18}H_{24}F_2O_6SNH_4$, 424.1606; found 424.1609. For clarity, the $^{1}$H NMR, $^{13}$C NMR, and $^{19}$F NMR data of the major and minor diastereomers have been tabulated individually.

**Major Diastereomer: $^{1}$H NMR** (600 MHz, $CDCl_3$) δ 7.09 (d, *J* = 8.5 Hz, 2H), 6.82 (d, *J* = 8.5 Hz, 2H), 6.29–6.23 (m, 1H), 5.85–5.74 (m, 1H), 4.80–4.76 (m, 1H), 4.29 (q, *J* = 7.1 Hz, 2H), 3.75 (s, 3H), 2.97 (s, 3H), 2.78–2.51 (m, 4H), 2.03–1.88 (m, 2H), 1.30 (t, *J* = 7.2 Hz, 3H); **$^{13}$C NMR** (150.9 MHz, $CDCl_3$) δ 163.5 (t, *J* = 34.3 Hz), 157.99, 133.4 (t, *J* = 9.3 Hz), 132.3, 129.2 (2C), 125.0 (t, *J* = 25.2 Hz), 113.9 (2C), 111.8 (t, *J* = 247.9 Hz), 80.3, 63.0, 55.1, 38.4, 37.0, 36.1, 30.1, 13.73; **$^{19}$F NMR** (564.6 MHz, $CDCl_3$) δ -103.3 (dd, *J* = 23.0, 10.6 Hz, 2F).

**Minor Diastereomer: $^{1}$H NMR** (600 MHz, $CDCl_3$) δ 7.09 (d, *J* = 8.5 Hz, 2H), 6.82 (d, *J* = 8.5 Hz, 2H), 6.05–6.00 (m, 1H), 5.85–5.74 (m, 1H), 4.80–4.76 (m, 1 H), 4.29 (q, *J* = 7.1 Hz, 2H), 3.75 (s, 3H), 2.98 (s, 3H), 2.78–2.51 (m, 4H), 2.03–1.88 (m, 2H), 1.30 (t, *J* = 7.2 Hz, 3H); **$^{13}$C NMR** (150.9 MHz, $CDCl_3$) δ 163.7 (t, *J* = 34.4 Hz), 157.96, 135.3 (t, *J* = 6.8 Hz), 132.4, 129.2 (2C), 123.9 (t, *J* = 26.2 Hz), 113.9 (2C), 112.6 (t, *J* = 249.3 Hz), 80.8, 63.2, 55.1, 38.4, 37.0, 36.2, 30.1, 13.71; **$^{19}$F NMR** (564.6 MHz, $CDCl_3$) δ -99.2 (d, *J* = 14.1 Hz, 2F).

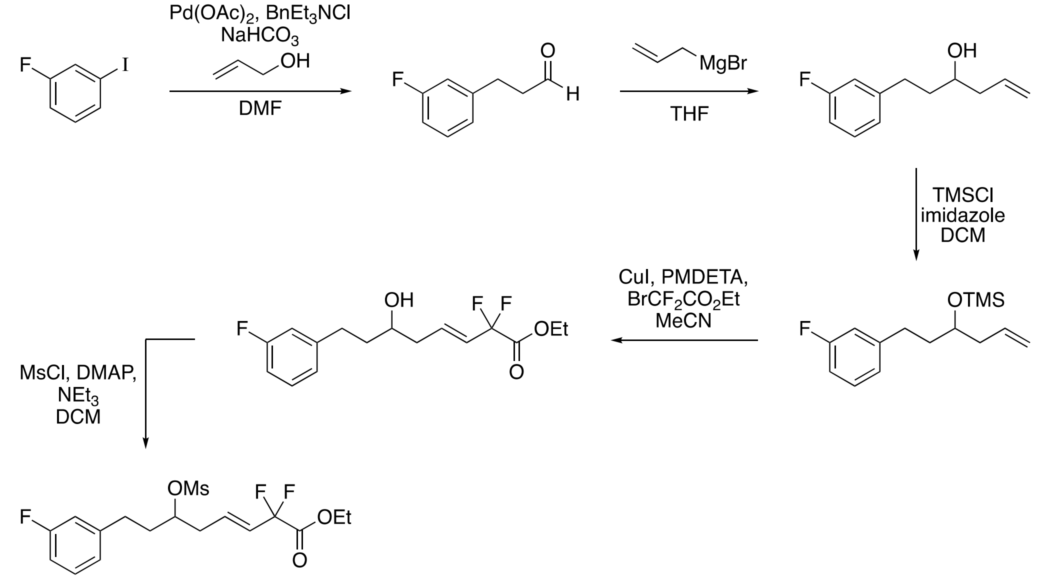


**Scheme SI-10:** Synthesis of Mesylate **SI-14**

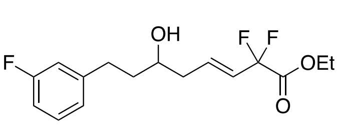

**Alcohol (SI-33)** was prepared according to Method B. The following amounts of reagents were used: silylated alcohol (0.80 g, 3.0 mmol, 1.0 equiv), CuI (57 mg, 0.30 mmol, 0.10 equiv), PMDETA (0.94 mL, 4.5 mmol, 1.5 equiv), ethyl bromodifluoroacetate (0.58 mL, 4.5 mmol, 1.5 equiv), and MeCN (6.0 mL, 0.5 M in substrate).The compound was purified by flash column chromatography (0–40% EtOAc/hexanes) to afford the title compound as a 3:1 mixture of alkene

diastereomers as a yellow oil (57 mg, 0.18 mmol, 6.0%). For clarity, the $^{1}$H NMR data of the major and minor diastereomers have been tabulated individually.

**Major Diastereomer: $^{1}$H NMR** (400 MHz, $CDCl_3$) δ 7.27–7.22 (m, 1H), 6.98 (d, $J$ = 7.9 Hz, 1H), 6.93–6.86 (m, 2H), 6.37–6.27 (m, 1H), 5.86–5.71 (m, 1H), 4.33 (q, $J$ = 7.2 Hz, 2H), 3.74 (br s, 1H), 2.86–2.77 (m, 1H), 2.74–2.65 (m, 1H), 2.57–2.25 (m, 2H), 1.84–1.74 (m, 2H), 1.56–1.48 (m, 1H), 1.35 (t, $J$ = 7.1 Hz, 3H).

**Minor Diastereomer: $^{1}$H NMR** (400 MHz, $CDCl_3$) δ 7.27–7.22 (m, 1H), 6.98 (d, $J$ = 7.9 Hz, 1H), 6.93–6.86 (m, 2H), 6.12–6.03 (m, 1H), 5.86–5.71 (m, 1H), 4.33 (q, $J$ = 7.2 Hz, 2H), 3.74 (br s, 1H), 2.86–2.77 (m, 1H), 2.74–2.65 (m, 1H), 2.57–2.25 (m, 2H), 1.84–1.74 (m, 2H), 1.56–1.48 (m, 1H), 1.35 (t, $J$ = 7.1 Hz, 3H).

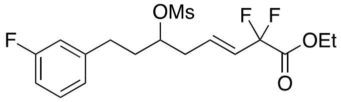


**Mesylate (SI-14)** was prepared according to Method C. The following amounts of reagents were used: alcohol **SI-33** (57 mg, 0.18 mmol, 1.0 equiv), MsCl (20. µL, 0.22 mmol, 1.2 equiv), DMAP (5.0 mg, 40 µmol, 20. mol %), $Et_3N$ (50. µL, 0.36 mmol, 2.0 equiv), DCM (2.0 mL, 0.10 M in substrate). The compound was purified by flash column chromatography (0–20% EtOAc/hexanes) to afford the title compound as a 3:1 mixture of alkene diastereomers as a yellow oil (50. mg, 0.13 mmol, 70%). **TLC $R_f$** = 0.4 (30% EtOAc/hexanes); **HRMS** (TOF MS ES+) *m/z*: $[M + Na]^+$ calculated for $C_{17}H_{21}F_3O_5SNa$, 417.0959; found, 417.0941. For clarity, the $^{1}$H NMR, $^{13}$C NMR, and $^{19}$F NMR data of the major and minor diastereomers have been tabulated individually.

**Major Diastereomer: $^{1}$H NMR** (400 MHz, $CDCl_3$) δ 7.33–7.25 (m, 1H), 7.02 (d, $J$ = 7.5 Hz, 1H), 6.94 (d, $J$ = 9.5 Hz, 2H), 6.36–6.26 (m, 1H), 5.94–5.79 (m, 1H), 4.91–4.80 (m, 1H), 4.39–4.29 (m, 2H), 3.06 (s, 3H), 2.88–2.57 (m, 4H), 2.13–1.94 (m, 2H), 1.36 (t, $J$ = 7.2 Hz, 3H); **$^{13}$C NMR** (125.7 Hz, $CDCl_3$) δ 163.7 (t, $J$ = 34.2 Hz), 162.9 (d, $J$ = 246.0 Hz), 143.0 (d, $J$ = 6.9 Hz), 133.2 (t, $J$ = 9.3 Hz), 130.13 (d, $J$ = 8.3 Hz), 125.5 (t, $J$ = 25.2 Hz), 124.1 (d, $J$ = 2.8 Hz), 115.3 (d, $J$ = 21.3 Hz), 113.3 (d $J$ = 21.3 Hz), 111.8 (t, $J$ = 248.3 Hz), 80.0, 63.2, 38.8, 37.2, 35.8, 31.0, 13.9; **$^{19}$F NMR** (376.5 MHz, $CDCl_3$) δ -103.4 (d, $J$ = 10.9 Hz, 1F), -103.5 (d, $J$ = 10.9 Hz, 1F), -113.20 (t, $J$ = 9.5 Hz, 1F).

**Minor Diastereomer: $^{1}$H NMR** (400 MHz, $CDCl_3$) δ 7.33–7.25 (m, 1H), 7.02 (d, $J$ = 7.5 Hz, 1H), 6.94 (d, $J$ = 9.5 Hz, 2H), 6.12–6.04 (m, 1H), 5.94–5.79 (m, 1H), 4.91–4.80 (m, 1H), 4.39–4.29 (m, 2H), 3.05 (s, 3H), 2.88–2.57 (m, 4H), 2.13–1.94 (m, 2H), 1.36 (t, $J$ = 7.2 Hz, 3H); **$^{13}$C NMR** (125.7 Hz, $CDCl_3$) δ 163.9 (t, $J$ = 34.2 Hz), 162.9 (d, $J$ = 246.0 Hz), 143.1 (d, $J$ = 6.9 Hz), 135.1 (t, $J$ = 9.3 Hz), 130.07 (d, $J$ = 8.3 Hz), 124.3 (t, $J$ = 25.2 Hz), 124.1 (d, $J$ = 2.8 Hz), 115.3 (d, $J$ = 21.3 Hz), 113.2 (d, $J$ = 20.8 Hz), 112.6 (t, $J$ = 249.7 Hz ), 80.6, 63.3, 38.8, 37.2, 35.8, 33.5, 13.9; **$^{19}$F NMR** (376.5 MHz, $CDCl_3$) δ -99.3 to –99.4 (m, 2F), -113.19 (t, $J$ = 9.5 Hz, 1F).

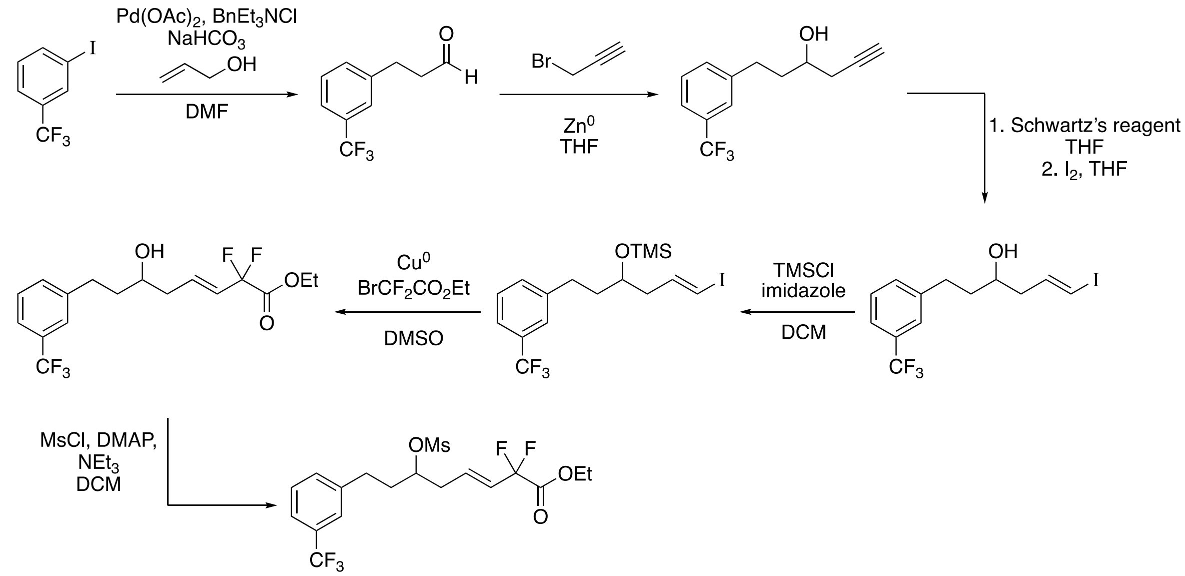


**Scheme SI-11:** Synthesis of Mesylate **SI-15**

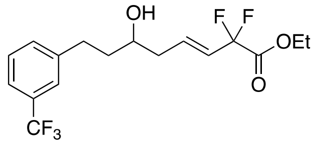


**Alcohol (SI-34)** was prepared following a procedure reported by Kumadaki.[xvii] In a flame-dried round-bottom flask equipped with a stir bar was added silylated alcohol (0.59 g, 1.3 mmol, 1.0 equiv), $Cu^0$ (0.26 g, 4.1 mmol, 3.1 equiv), ethyl bromodifluoroacetate (0.20 mL, 1.6 mmol, 1.2 equiv), and DMSO (6.7 mL, 0.20 M in substrate).The reaction flask was equipped with a reflux condenser, heated to 55 °C, and allowed to stir for 16 h. After 16 h, the reaction mixture was poured over a cold 1:1 mixture of $NH_4Cl$ and DI $H_2O$ (10 mL total). The biphasic mixture was extracted with $Et_2O$, washed with $NH_4Cl$ then brine, and dried over $Na_2SO_4$. The residue was purified by flash column chromatography (0–20% EtOAc/hexanes) to afford the title compound as a single alkene diastereomer as a pale-yellow oil (0.12 g, 0.33 mmol, 25%). **TLC R$_f$** = 0.3 (20% EtOAc/hexanes, CAM stain); **$^1$H NMR** (500 MHz, $CDCl_3$) δ 7.45–7.38 (m, 4H), 6.35–6.29 (m, 1H), 5.83–5.75 (m, 1H), 4.31 (aq, *J* = 6.9 Hz, 2H), 3.74 (ap s, 1H), 2.91–2.85 (m, 1H), 2.77–2.71 (m, 1H), 2.40–2.29 (m, 2H), 2.09 (ap s, 1H), 1.81–1.77 (m, 2H), 1.32 (t, *J* = 7.1 Hz, 3H); **$^{13}$C NMR** (125.7 Hz, $CDCl_3$) δ 164.1 (t, *J* = 34.7 Hz), 142.8, 135.8 (t, *J* = 9.0 Hz), 132.0, 130.8 (q, *J* = 31.3 Hz), 129.0, 125.2 (q, *J* = 3.9 Hz), 124.3 (q, *J* = 272.3 Hz), 124.1 (t, *J* = 25.0 Hz), 122.9 (q, *J* = 4.2 Hz), 112.1 (t, *J* = 247.8 Hz), 69.6, 63.2, 40.2, 38.4, 31.8, 13.9; **HRMS** (TOF MS ES+) *m/z*: $[M + Na]^+$ calculated for $C_{17}H_{19}F_5O_3Na$, 389.1152; found 389.1135.

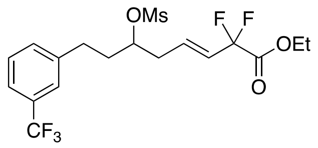


**Mesylate (SI-15)** was prepared according to Method C. The following amounts of reagents were used: alcohol **SI-34** (0.16 g, 0.42 mmol, 1.0 equiv), $Et_3N$ (90. µL, 0.64 mmol, 1.5 equiv), DMAP (5.2 mg, 42 µmol, 0.10 equiv), MsCl (40. µL, 0.51 mmol, 1.2 equiv), and DCM (2.1 mL, 0.20 M in substrate). The compound was purified by flash column chromatography (0–20%

EtOAc/hexanes) to afford the title compound as a single alkene diastereomer as a yellow oil (0.13 g, 0.29 mmol, 67%). **TLC R$_f$** = 0.3 (20% EtOAc/hexanes, CAM); **$^1$H NMR** (500 MHz, $CDCl_3$) δ 7.48–7.38 (m, 4H), 6.35–6.25 (m, 1H), 5.87–5.79 (m, 1H), 4.84 (br s, 1H), 4.31 (q, *J* = 7.0 Hz, 2H), 3.02 (s, 3H), 2.89–2.74 (m, 2H), 2.66–2.57 (m, 2H), 2.10–1.95 (m, 2H), 1.32 (t, *J* = 7.1 Hz, 3H); **$^{13}$C NMR** (125.7 Hz, $CDCl_3$) δ 163.7 (t, *J* = 34.2 Hz), 141.5, 133.2 (t, *J* = 9.2 Hz), 132.0, 131.0 (q, *J* = 32.3 Hz), 129.2, 125.6 (t, *J* = 25.2 Hz), 125.1 (q, *J* = 3.8 Hz), 124.2 (q, *J* = 272.3 Hz), 123.4 (q, *J* = 3.7 Hz), 111.9 (t, *J* = 248.5 Hz), 79.9, 63.3, 38.8, 37.3, 36.0, 31.2, 14.0; **$^{19}$F NMR** (376.5 MHz, $CDCl_3$) δ -62.9 (3F), -103.7 (dd, *J* = 36.7, 10.4 Hz, 2F); **HRMS** (TOF MS ES+) *m/z*: $[M + NH_4]^+$ calculated for $C_{18}H_{21}F_5O_5SNH_4$, 462.1374; found 462.1362.

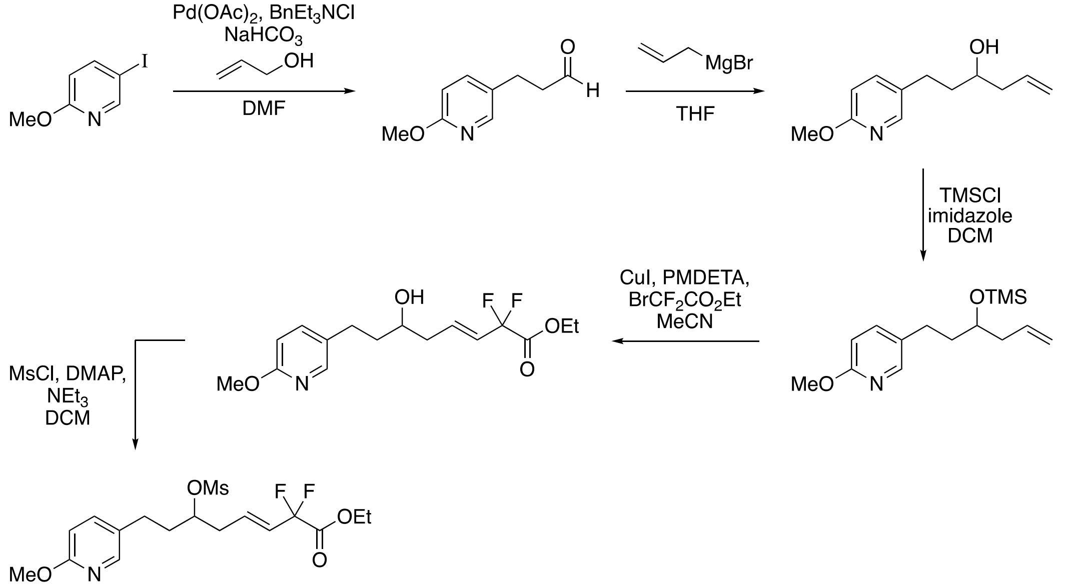


**Scheme SI-12:** Synthesis of Mesylate **SI-16**

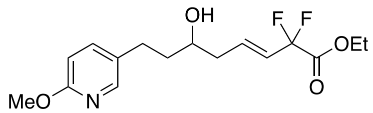

**Alcohol (SI-35)** was prepared according to Method B. The following amounts of reagents were used: silylated alcohol (0.84 g, 3.0 mmol, 1.0 equiv), CuI (57 mg, 0.30 mmol, 0.10 equiv), PMDETA (0.94 mL, 4.5 mmol, 1.5 equiv), ethyl bromodifluoroacetate (0.58 mL, 4.5 mmol, 1.5 equiv), and MeCN (15 mL, 0.20 M in substrate). The compound was purified by flash column chromatography (0–20% EtOAc/hexanes) to afford the title compound as a 6:1 mixture of alkene diastereomers as a clear, yellow oil (0.32 g, 0.97 mmol, 32%). For clarity, the $^1$H NMR data of the major and minor diastereomers have been tabulated individually.

**Major Diastereomer: $^1$H NMR** (400 MHz, $CDCl_3$) δ 7.96 (s, 1H), 7.39 (dd, *J* = 8.5, 2.5 Hz, 1H), 6.65 (d, *J* = 8.6 Hz, 1H), 6.34–6.23 (m, 1H), 5.82–5.64 (m, 1H), 4.29 (q, *J* = 7.1 Hz, 2H), 3.88 (s, 3H), 3.74–3.65 (m, 1H), 2.75–2.65 (m, 1H), 2.63–2.53 (m, 1H), 2.40–2.24 (m, 2H), 2.16 (d, *J* = 4.6 Hz, 1H), 1.75–1.66 (m, 2H), 1.31 (t, *J* = 7.1 Hz, 3H).

**Minor Diastereomer: $^1$H NMR** (400 MHz, $CDCl_3$) δ 7.96 (s, 1H), 7.39 (dd, *J* = 8.5, 2.5 Hz, 1H), 6.65 (d, *J* = 8.6 Hz, 1H), 6.09–6.00 (m, 1H), 5.82–5.64 (m, 1H), 4.29 (q, *J* = 7.1 Hz, 2H), 3.88 (s,

3H), 3.74–3.65 (m, 1H), 2.75–2.65 (m, 1H), 2.63–2.53 (m, 1H), 2.40–2.24 (m, 2H), 2.16 (d, *J* = 4.6 Hz, 1H), 1.75–1.66 (m, 2H), 1.31 (t, *J* = 7.1 Hz, 3H).

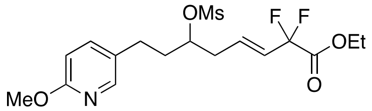


**Mesylate (SI-16)** was prepared according to Method C. The following amounts of reagents were used: alcohol **SI-35** (0.32 g, 0.96 mmol, 1.0 equiv), DMAP (23 mg, 0.19 mmol, 20. mol %), $Et_3N$ (0.26 mL, 1.9 mmol, 2.0 equiv), MsCl (90. µL, 1.2 mmol, 1.2 equiv), and DCM (5.0 mL, 0.20 M in substrate). The compound was purified by flash column chromatography (0–20% EtOAc/hexanes) to afford the title compound as a 3:1 mixture of alkene diastereomers as a yellow oil (0.30 g, 0.31 mmol, 76%). **TLC $R_f$** = 0.2 (30% EtOAc/hexanes); **HRMS** (TOF MS ES+) *m/z*: $[M + H]^+$ calculated for $C_{17}H_{23}F_2O_6SNH$, 408.1292; found 408.1291. For clarity, the $^1H$ NMR, $^{13}C$ NMR, and $^{19}F$ NMR data of the major and minor diastereomers have been tabulated individually.

**Major Diastereomer: $^1$H NMR** (500 MHz, $CDCl_3$) δ 8.03 (s, 1H), 7.47 (dd, *J* = 8.5, 2.5 Hz, 1H), 6.74 (d, *J* = 8.7 Hz, 1H), 6.36–6.26 (m, 1H), 5.95–5.78 (m, 1H), 4.91–4.78 (m, 1H), 4.37 (q, *J* = 7.1 Hz, 2H), 3.96 (s, 3H), 3.07 (s, 3H), 2.81–2.52 (m, 4H), 2.12–2.01 (m, 1H), 2.01–1.89 (m, 1H), 1.38 (t, *J* = 7.1 Hz, 3H); **$^{13}$C NMR** (125.7 MHz, $CDCl_3$) δ 163.7 (t, *J* = 34.2 Hz), 163.0, 146.1, 138.8, 133.2 (t, *J* = 9.3 Hz), 128.3, 125.5 (t, *J* = 25.2 Hz), 111.8 (t, *J* = 248.3 Hz), 110.8, 79.9, 63.2, 53.4, 38.8, 37.2, 36.1, 27.5, 14.0; **$^{19}$F NMR** (376.5, $CDCl_3$) δ -103.4 (d, *J* = 10.9 Hz, 1F), -103.5 (d, *J* = 10.9 Hz, 1F).

**Minor Diastereomer: $^1$H NMR** (500 MHz, $CDCl_3$) δ 8.03 (s, 1H), 7.47 (dd, *J* = 8.5, 2.5 Hz, 1H), 6.74 (d, *J* = 8.7 Hz, 1H), 6.11–6.03 (m, 1H), 5.95–5.78 (m, 1H), 4.91–4.78 (m, 1H), 4.37 (q, *J* = 7.1 Hz, 2H), 3.96 (s, 3H), 3.07 (s, 3H), 2.81–2.52 (m, 4H), 2.12–2.01 (m, 1H), 2.01–1.89 (m, 1H), 1.38 (t, *J* = 7.1 Hz, 3H); **$^{13}$C NMR** (125.7 MHz, $CDCl_3$) δ 163.7 (t, *J* = 34.2 Hz), 163.0, 146.1, 138.8, 133.2 (t, *J* = 9.3 Hz), 128.3, 125.5 (t, *J* = 25.2 Hz), 111.8 (t, *J* = 248.3 Hz), 110.8, 79.9, 63.2, 53.4, 38.8, 37.2, 36.1, 27.5, 14.0; **$^{19}$F NMR** (376.5, $CDCl_3$) δ -102.9 (d, *J* = 10.9 Hz, 1F), -104.0 (d, *J* = 10.9 Hz, 1F).

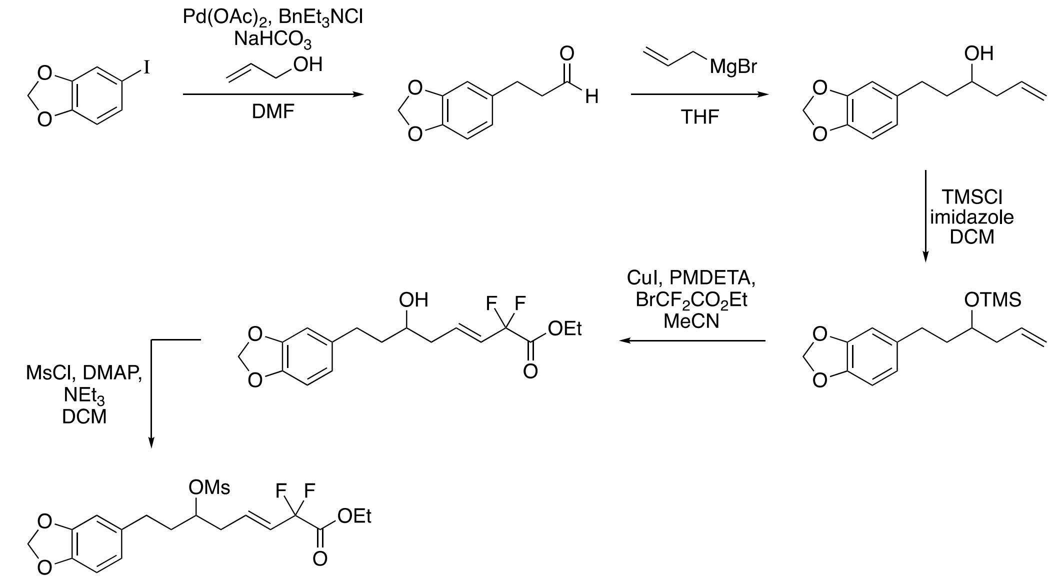

**Scheme SI-13:** Synthesis of Mesylate **SI-17**

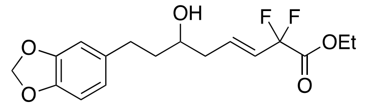

**Alcohol (SI-36)** was prepared according to Method B. In a flame-dried round-bottom flask equipped with a stir bar was added silylated alcohol (0.74 g, 2.5 mmol, 1.0 equiv), CuI (48 mg, 0.25 mmol, 0.10 equiv), ethyl bromodifluoroacetate (0.93 mL, 3.8 mmol, 1.5 equiv), PMDETA (0.78 mL, 3.8 mmol, 1.5 equiv), and MeCN (15 mL, 0.17 M in substrate). The compound was purified by flash column chromatography (0–40% EtOAc/hexanes) to afford the title compound as a clear, yellow oil (86 mg, 0.25 mmol, 10%). **$^{1}$H NMR** (400 MHz, $CDCl_3$) δ 6.74–6.59 (m, 3H), 6.35–6.25 (m, 1H), 5.90 (s, 2H), 5.82–5.66 (m, 1H), 4.34–4.26 (m, 2H), 3.75–3.66 (br s, 1H), 2.75–2.54 (m, 2H), 2.39–2.24 (m, 2H), 1.77–1.68 (m, 2H), 1.54–1.48 (m, 1H), 1.32 (t, *J* = 7.1 Hz, 3H).

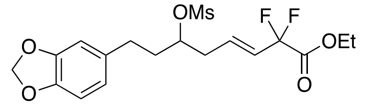

**Mesylate (SI-17)** was prepared according to Method C. The following amounts of reagents were used: alcohol **SI-36** (0.35 g, 1.0 mmol, 1.0 equiv), MsCl (90. µL, 1.2 mmol, 1.2 equiv), DMAP (26 mg, 0.21 mmol, 20. mol %), $Et_3N$ (0.29 mL, 2.1 mmol, 2.0 equiv), DCM (5.0 mL, 0.20 M in substrate). The compound was purified by flash column chromatography (0–20% EtOAc/hexanes) to afford the title compound as a yellow oil (0.31 g, 0.70 mmol, 70%). **TLC $R_f$** = 0.3 (30% EtOAc/hexanes); **$^{1}$H NMR** (400 MHz, $CDCl_3$) δ 6.77–6.60 (m, 3H), 6.33–6.20 (m, 1H), 5.93 (s, 2H), 5.89–5.75 (m, 1H), 4.81 (quint, *J* = 6.2 Hz, 1H), 4.32 (q, *J* = 7.1 Hz, 2H), 3.02 (s, 3H), 2.75–2.49 (m, 4H), 2.07–1.85 (m, 2H), 1.34 (t, *J* = 6.2 Hz, 3H); **$^{13}$C NMR** (125.7 MHz, $CDCl_3$) δ 163.7 (t, *J* = 34.2 Hz), 147.8, 146.1, 134.1, 133.3 (t, *J* = 9.3 Hz), 125.4 (t, *J* = 25.0 Hz), 121.2, 111.8 (t, *J* = 248.3 Hz), 108.8, 108.4, 100.9, 80.2, 63.2, 38.8, 37.2, 36.3, 31.0, 14.0; **$^{19}$F NMR** (376.5, $CDCl_3$) δ -103.6 (d, *J* = 10.3 Hz, 1F), -103.7 (d, *J* = 10.3 Hz, 1F); **HRMS** (TOF MS ES+) m/z: $[M + Na]^+$ calculated for $C_{18}H_{22}F_2O_7SNa$, 443.0952; found, 443.0931.

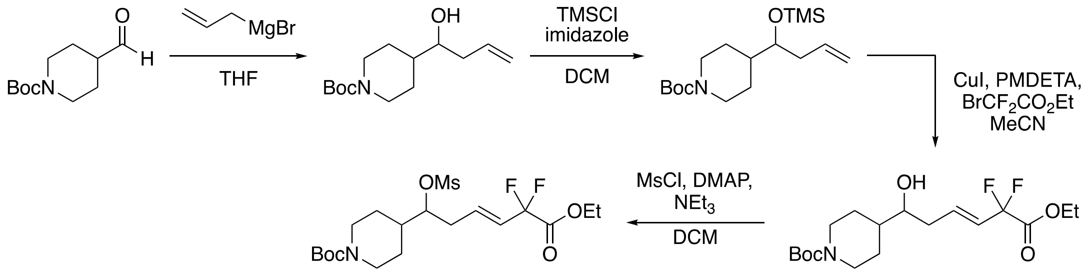

**Scheme SI-14:** Synthesis of Mesylate **SI-18**

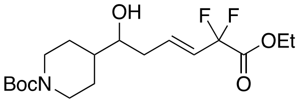

**Alcohol (SI-37)** was prepared according to Method B. The following amounts of reagents were used: silylated alcohol (0.96 g, 2.9 mmol, 1.0 equiv), CuI (56 mg, 0.29 mmol, 10. mol %), PMDETA (0.92 mL, 4.4 mmol, 1.5 equiv), ethyl bromodifluoroacetate (0.57 mL, 4.4 mmol, 1.5 equiv), and MeCN (15 mL, 0.20 M in substrate). The compound was purified by flash column chromatography (0–30% EtOAc/hexanes) to afford the title compound as a mixture of alkene diastereomers as a yellow oil (0.47 g, 1.2 mmol, 42%, 3.8:1 dr). **TLC R$_f$** = 0.3 (30% EtOAc/hexanes, $KMnO_4$ stain). For clarity, the $^1$H NMR data of the major and minor diastereomers have been tabulated individually.

**Major diastereomer: $^1$H NMR** (600 MHz, $CDCl_3$) δ 6.37–6.28 (m, 1H), 5.83–5.69 (m, 1H), 4.32 (q, *J* = 7.1 Hz, 2H), 4.16 (br s, 2H), 3.52–3.45 (m, 1H), 2.66 (br s, 2H), 2.48–2.38 (m, 1H), 2.29–2.24 (m, 1H), 1.81–1.79 (m, 1H), 1.60–1.57 (m, 1H), 1.54–1.49 (m, 2H), 1.45 (s, 9H), 1.35 (t, *J* = 7.2 Hz, 3H), 1.30–1.19 (m, 2H).

**Minor diastereomer: $^1$H NMR** (600 MHz, $CDCl_3$) δ 6.14–6.09 (m, 1H), 5.94–5.89 (m, 1H), 4.32 (q, *J* = 7.1 Hz, 2H), 4.16 (br s, 2H), 3.52–3.45 (m, 1H), 2.66 (br s, 2H), 2.48–2.38 (m, 1H), 2.29–2.24 (m, 1H), 1.81–1.79 (m, 1H), 1.60–1.57 (m, 1H), 1.54–1.49 (m, 2H), 1.46 (s, 9H), 1.35 (t, *J* = 7.2 Hz, 3H), 1.30–1.19 (m, 2H).

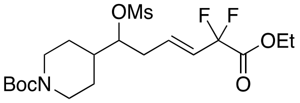

**Mesylate (SI-18)** was prepared according to Method C. The following amounts of reagents were used: alcohol **SI-37** (0.37 g, 0.98 mmol, 1.0 equiv), $Et_3N$ (0.21 mL, 1.5 mmol, 1.5 equiv), DMAP (12 mg, 98 µmol, 0.10 equiv), MsCl (0.12 mL, 1.5 mmol, 1.5 equiv), and DCM (4.9 mL, 0.20 M in substrate). The compound was purified by flash column chromatography (0–20% EtOAc/hexanes) to afford the title compound as a mixture of alkene diastereomers as a viscous yellow oil (0.37 g, 0.81 mmol, 82%, 6.4:1 dr). **TLC R$_f$** = 0.3 (25% EtOAc/hexanes, $KMnO_4$ stain); **HRMS** (TOF MS ES+) *m/z*: $[M + Na]^+$ calculated for $C_{19}H_{31}F_2NO_7SNa$, 478.1687; found 478.1682. For clarity, the $^1$H NMR, $^{13}$C NMR, and $^{19}$F NMR data of the major and minor diastereomers have been tabulated individually.

**Major diastereomer: $^1$H NMR** (400 MHz, $CDCl_3$) δ 6.32–6.25 (m, 1H), 5.90–5.81 (m, 1H), 4.63 (aq, *J* = 5.9 Hz, 1H), 4.34 (q, *J* = 7.1 Hz, 2H), 4.19 (br s, 2H), 3.01 (s, 3H), 2.70–2.51 (m, 4H),

1.85–1.76 (m, 2H), 1.67–1.62 (m, 1H), 1.46 (s, 9H), 1.36 (t, *J* = 7.1 Hz, 3H), 1.33–1.27 (m, 2H); **$^{13}$C NMR** (125.7 MHz, $CDCl_3$) δ 163.8 (t, *J* = 34.2 Hz), 154.8, 133.6 (t, *J* = 9.3 Hz), 125.5 (t, *J* = 25.2 Hz), 111.9 (t, *J* = 247.4 Hz), 83.8, 79.8, 63.3, 43.5 (2C), 40.0, 38.9, 34.4, 28.6 (3C), 27.7 (2C), 14.1; **$^{19}$F NMR** (376.5 MHz, $CDCl_3$) δ -103.7 (d, *J* = 10.4 Hz, 2F).

**Minor diastereomer: $^{1}$H NMR** (400 MHz, $CDCl_3$) δ 6.10–6.03 (m, 1H), 5.90–5.81 (m, 1H), 4.63 (aq, *J* = 5.9 Hz, 1H), 4.34 (q, *J* = 7.1 Hz, 2H), 4.19 (br s, 2H), 3.03 (s, 3H), 2.70–2.51 (m, 4H), 1.85–1.76 (m, 2H), 1.67–1.62 (m, 1H), 1.46 (s, 9H), 1.36 (t, *J* = 7.1 Hz, 3H), 1.33–1.27 (m, 2H); **$^{13}$C NMR** (125.7 MHz, $CDCl_3$) δ 163.8 (t, *J* = 34.2 Hz), 154.8, 133.6 (t, *J* = 9.3 Hz), 125.5 (t, *J* = 25.2 Hz), 111.9 (t, *J* = 247.4 Hz), 83.8, 79.8, 63.3, 43.5 (2C), 40.0, 38.9, 34.4, 28.6 (3C), 27.7 (2C), 14.1; **$^{19}$F NMR** (376.5 MHz, $CDCl_3$) δ -103.5 (d, *J* = 10.0 Hz, 2F).

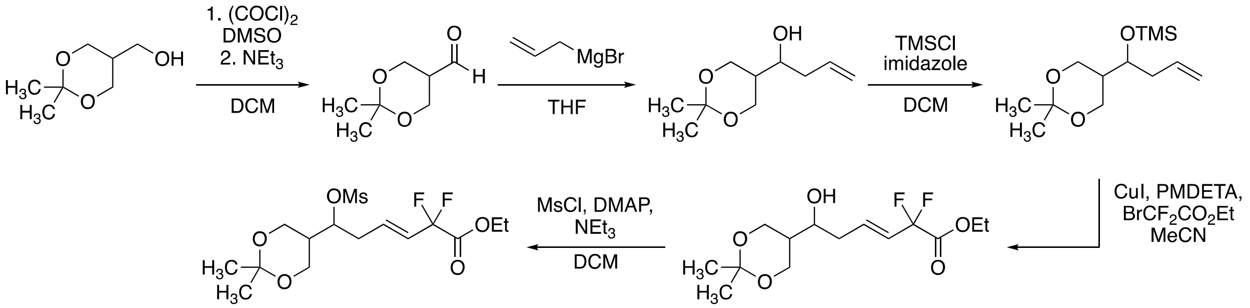


**Scheme SI-15:** Synthesis of Mesylate **SI-19**

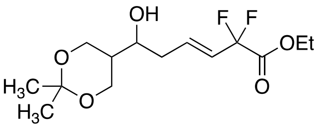

**Alcohol (SI-38)** was prepared according to Method B. The following amounts of reagents were used: silylated alcohol (0.54 g, 2.1 mmol, 1.0 equiv), CuI (40. mg, 0.21 mmol, 10. mol %), PMDETA (0.65 mL, 3.1 mmol, 1.5 equiv), ethyl bromodifluoroacetate (0.40 mL, 3.1 mmol, 1.5 equiv), and MeCN (10. mL, 0.20 M in substrate). The compound was purified by flash column chromatography (0–40% EtOAc/hexanes) to afford the title compound as a mixture of alkene diastereomers as a yellow oil (0.19 g, 0.60 mmol, 29%, 2.2:1 dr). **TLC R$_f$** = 0.4 (50% EtOAc/hexanes, CAM stain). For clarity, the $^{1}$H NMR data of the major and minor diastereomers have been tabulated individually.

**Major diastereomer: $^{1}$H NMR** (400 MHz, $CDCl_3$) δ 6.37–6.28 (m, 1H), 5.87–5.70 (m, 1H), 4.33 (q, *J* = 7.1 Hz, 2H), 4.05–3.75 (m, 5H), 2.57–2.26 (m, 2H), 2.00 (d, *J* = 4.3 Hz, 1H), 1.74–1.67 (m, 1H), 1.43 (s, 3H), 1.40 (s, 3H), 1.35 (t, *J* = 7.1 Hz, 3H).

**Minor diastereomer: $^{1}$H NMR** (400 MHz, $CDCl_3$) δ 6.13–6.06 (m, 1H), 5.87–5.70 (m, 1H), 4.33 (q, *J* = 7.1 Hz, 2H), 4.05–3.75 (m, 5H), 2.57–2.26 (m, 2H), 1.99 (d, *J* = 4.3 Hz, 1H), 1.74–1.67 (m, 1H), 1.43 (s, 3H), 1.40 (s, 3H), 1.34 (t, *J* = 7.1 Hz, 3H).

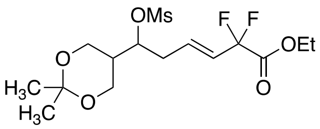

**Mesylate (SI-19)** was prepared according to Method C. The following amounts of reagents were used: alcohol **SI-38** (0.19 g, 0.62 mmol, 1.0 equiv), $Et_3N$ (0.13 mL, 0.93 mmol, 1.5 equiv), DMAP (7.6 mg, 62 μmol, 0.10 equiv), MsCl (60. μL, 0.74 mmol, 1.2 equiv), and DCM (3.1 mL, 0.20 M in substrate). The compound was purified by flash column chromatography (0–50% EtOAc/hexanes) to afford the title compound as a mixture of alkene diastereomers as a yellow oil (0.16 g, 0.42 mmol, 67%, 2.2:1 dr). **TLC** $\mathbf{R_f}$ = 0.5 (50% EtOAc/hexanes, CAM stain); **HRMS** (TOF MS ES+) *m/z*: $[M + Na]^+$ calculated for $C_{15}H_{24}F_2O_7SNa$, 409.1108; found 409.1110. For clarity, the $^1$H NMR, $^{13}$C NMR, and $^{19}$F NMR data of the major and minor diastereomers have been tabulated individually.

**Major diastereomer: $^1$H NMR** (600 MHz, $CDCl_3$) δ 6.34–6.28 (m, 1H), 5.89–5.77 (m, 1H), 5.02–4.98 (m, 1H), 4.33 (q, *J* = 7.1 Hz, 2H), 4.06–3.98 (m, 2H), 3.89–3.78 (m, 2H), 3.05 (s, 3H), 2.92–2.60 (m, 2H), 1.93–1.89 (m, 1H), 1.44 (s, 3H), 1.40 (s, 3H), 1.36 (t, *J* = 7.1 Hz, 3H); **$^{13}$C NMR** (150.9 MHz, $CDCl_3$) δ 163.7 (t, *J* = 34.3 Hz), 133.3 (t, *J* = 9.3 Hz), 125.6 (t, *J* = 25.2 Hz), 111.9 (t, *J* = 248.3 Hz), 98.61, 79.7, 63.3, 60.35, 60.2, 38.45, 37.3, 35.2, 25.6, 22.2, 14.02; **$^{19}$F NMR** (376.5 MHz, $CDCl_3$) δ -103.6 (dd, *J* = 142.1, 10.3 Hz, 2F).

**Minor diastereomer: $^1$H NMR** (600 MHz, $CDCl_3$) δ 6.13–6.09 (m, 1H), 5.89–5.77 (m, 1H), 5.02–4.98 (m, 1H), 4.33 (q, *J* = 7.1 Hz, 2H), 4.06–3.98 (m, 2H), 3.89–3.78 (m, 2H), 3.07 (s, 3H), 2.92–2.60 (m, 2H), 1.93–1.89 (m, 1H), 1.44 (s, 3H), 1.40 (s, 3H), 1.35 (t, *J* = 7.1 Hz, 3H); **$^{13}$C NMR** (150.9 MHz, $CDCl_3$) δ 163.9 (t, *J* = 34.3 Hz), 135.3 (t, *J* = 6.6 Hz), 124.3 (t, *J* = 26.2 Hz), 112.7 (t, *J* = 249.6 Hz), 98.57, 79.9, 63.4, 60.33, 60.3, 38.43, 37.5, 35.2, 25.4, 22.4, 14.01; **$^{19}$F NMR** (376.5 MHz, $CDCl_3$) δ -99.8 (at, *J* = 16.1 Hz, 2F).

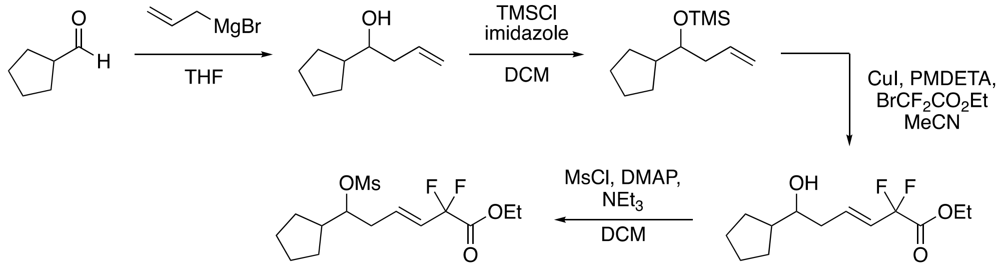


**Scheme SI-16:** Synthesis of Mesylate **SI-20**

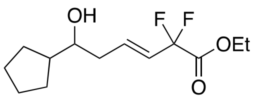

**Alcohol (SI-39)** was prepared according to Method B. The following amounts of reagents were used: silylated alcohol (0.56 g, 4.0 mmol, 1.0 equiv), CuI (76 mg, 0.40 mmol, 0.10 equiv), PMDETA (1.3 mL, 6.0 mmol, 1.5 equiv), ethyl bromodifluoroacetate (1.5 mL, 6.0 mmol, 1.5 equiv), and MeCN (20. mL, 0.20 M in substrate). The compound was purified by flash column chromatography (0–40% EtOAc/hexanes) to afford the title compound as a clear, yellow oil (0.31 g, 1.2 mmol, 29%).

**Major Diastereomer:** $^{1}$**H NMR** (500 MHz, $CDCl_3$) δ 6.44–6.30 (m, 1H), 5.87–5.66 (m, 1H), 4.33 (q, $J$ = 7.1 Hz, 2H), 3.57–3.48 (m, 1H), 2.47–2.19 (m, 2H), 1.85–1.75 (m, 1H), 1.74–1.45 (m, 6H), 1.42–1.30 (m, 3H), 1.29–1.14 (m, 2H).

**Minor Diastereomer:** $^{1}$**H NMR** (500 MHz, $CDCl_3$) δ 6.19–6.08 (m, 1H), 5.87–5.66 (m, 1H), 4.33 (q, $J$ = 7.1 Hz, 2H), 3.57–3.48 (m, 1H), 2.47–2.19 (m, 2H), 1.85–1.75 (m, 1H), 1.74–1.45 (m, 6H), 1.42–1.30 (m, 3H), 1.29–1.14 (m, 2H).

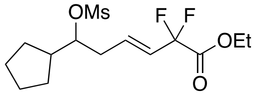


**Mesylate (SI-20)** was prepared according to Method C. The following amounts of reagents were used: alcohol **SI-39** (0.31 g, 1.2 mmol, 1.0 equiv), MsCl (0.11 mL, 1.4 mmol, 1.2 equiv), DMAP (29 mg, 0.24 mmol, 0.2 equiv), $Et_3N$ (0.33 mL, 2.4 mmol, 2.0 equiv), and DCM (5.0 mL, 0.24 M in substrate). The compound was purified by flash column chromatography (0–20% EtOAc/hexanes) to afford the title compound as a yellow oil (0.27 g, 0.79 mmol, 68%). TLC $R_f$ = 0.4 (30% EtOAc/hexanes); **HRMS** (TOF MS ES+) m/z: $[M + Na]^+$ calculated for $C_{14}H_{22}F_2O_5SNa$, 363.1054; found 363.1039.

**Major Diastereomer:** $^{1}$**H NMR** (400 MHz, $CDCl_3$) δ 6.37–6.26 (m, 1H), 5.90–5.73 (m, 1H), 4.69 (quint, $J$ = 6.2 Hz, 1H), 4.33 (q, $J$ = 7.1 Hz, 2H), 3.00 (s, 3H), 2.73–2.48 (m, 2H), 2.20–2.07 (m, 1H), 1.89–1.50 (m, 5H), 1.47–1.38 (m, 1H), 1.35 (t, J = 7.1 Hz, 3H), 1.31–1.22 (m, 1H); $^{13}$**C NMR** (125.7 MHz, $CDCl_3$) δ 163.7 (t, $J$ = 34.4 Hz), 133.9 (t, $J$ = 9.3 Hz), 125.0 (t, $J$ = 25.4 Hz), 111.9 (t, $J$ = 248.0 Hz), 84.9, 63.2, 43.5, 38.8, 36.6, 29.2, 28.8, 25.5, 25.2, 14.0; $^{19}$**F NMR** (376.5, $CDCl_3$) δ -103.5, -103.7.

**Minor Diastereomer:** $^{1}$**H NMR** (400 MHz, $CDCl_3$) δ 6.15–6.05 (m, 1H), 5.90–5.73 (m, 1H), 4.69 (quint, $J$ = 6.2 Hz, 1H), 4.33 (q, $J$ = 7.1 Hz, 2H), 3.00 (s, 3H), 2.73–2.48 (m, 2H), 2.20–2.07 (m, 1H), 1.89–1.50 (m, 5H), 1.47–1.38 (m, 1H), 1.35 (t, $J$ = 7.1 Hz, 3H), 1.31–1.22 (m, 1H); $^{13}$**C NMR** (125.7 MHz, $CDCl_3$) δ 163.7 (t, $J$ = 34.4 Hz), 136.0 (t, $J$ = 6.5 Hz), 123.7 (t, $J$ = 26.2 Hz), 111.9 (t, $J$ = 248.0 Hz), 85.6, 63.2, 43.5, 38.8, 36.6, 29.2, 28.8, 25.4, 25.2, 14.0; $^{19}$**F NMR** (376.5, $CDCl_3$) δ -103.5, -103.7.

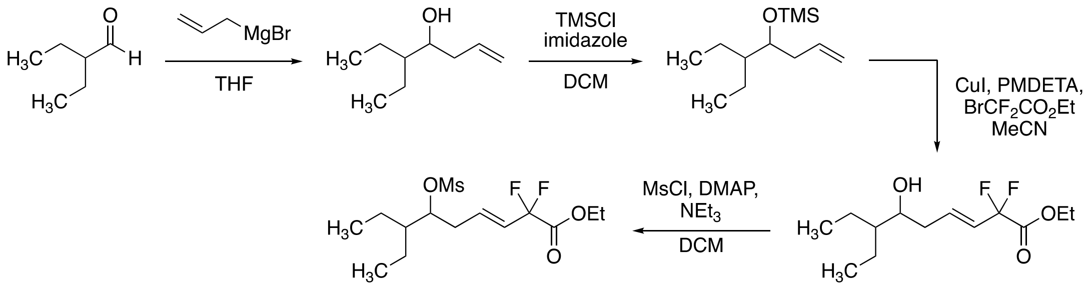

**Scheme SI-17:** Synthesis of Mesylate **SI-21**

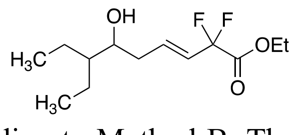

**Alcohol (SI-40)** was prepared according to Method B. The following amounts of reagents were used: silylated alcohol (1.1 g, 4.9 mmol, 1.0 equiv), CuI (94 mg, 0.49 mmol, 10. mol %), PMDETA (1.5 mL, 7.4 mmol, 1.5 equiv), ethyl bromodifluoroacetate (0.95 mL, 7.4 mmol, 1.5 equiv), and MeCN (25 mL, 0.20 M in substrate). The compound was purified by flash column chromatography (0–20% EtOAc/hexanes) to afford the title compound as a mixture of alkene diastereomers as a yellow oil (0.25 g, 0.93 mmol, 19%, 1.4:1 dr). **TLC R$_f$** = 0.4 (20% EtOAc/hexanes, CAM stain). For clarity, the $^1$H NMR of the major and minor diastereomers have been tabulated individually.

**Major diastereomer: $^1$H NMR** (400 MHz, $CDCl_3$) δ 6.39–6.30 (m, 1H), 5.84–5.67 (m, 1H), 4.32 (q, *J* = 7.1 Hz, 2H), 3.75–3.66 (m, 1H), 2.49–2.45 (m, 1H), 2.34–2.28 (m, 1H), 1.50–1.21 (m, 6H), 1.35 (t, *J* = 7.1 Hz, 3H), 0.91 (t, *J* = 7.5 Hz, 6H).

**Minor diastereomer: $^1$H NMR** (400 MHz, $CDCl_3$) δ 6.14–6.08 (m, 1H), 5.84–5.67 (m, 1H), 4.32 (q, *J* = 7.1 Hz, 2H), 3.75–3.66 (m, 1H), 2.49–2.45 (m, 1H), 2.34–2.28 (m, 1H), 1.50–1.21 (m, 6H), 1.35 (t, *J* = 7.1 Hz, 3H), 0.92 (t, *J* = 7.4 Hz, 6H).

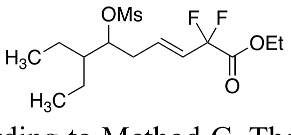

**Mesylate (SI-21)** was prepared according to Method C. The following amounts of reagents were used: alcohol **SI-40** (0.25 g, 0.93 mmol, 1.0 equiv), $Et_3N$ (0.19 mL, 1.4 mmol, 1.5 equiv), DMAP (11 mg, 93 µmol, 0.10 equiv), MsCl (90. µL, 1.1 mmol, 1.5 equiv), and DCM (4.7 mL, 0.20 M in substrate). The compound was purified by flash column chromatography (0–20% EtOAc/hexanes) to afford the title compound as a mixture of alkene diastereomers as a yellow oil (0.22 g, 0.65 mmol, 69%, 3.3:1 dr). **TLC R$_f$** = 0.3 (20% EtOAc/hexanes, CAM stain); **HRMS** (TOF MS ES+) *m/z*: $[M + Na]^+$ calculated for $C_{14}H_{24}F_2O_5SNa$, 365.1210; found 365.1210. For clarity, the $^1$H NMR, $^{13}$C NMR, and $^{19}$F NMR data of the major and minor diastereomers have been tabulated individually.

**Major diastereomer: $^1$H NMR** (400 MHz, $CDCl_3$) δ 6.32–6.24 (m, 1H), 5.89–5.72 (m, 1H), 4.85–4.80 (m, 1H), 4.32 (q, *J* = 7.1 Hz, 2H), 2.99 (s, 3H), 2.74–2.54 (m, 2H), 1.59–1.39 (m, 5H), 1.35 (t, *J* = 7.1 Hz, 3H), 0.94 (t, *J* = 7.4 Hz, 6H); **$^{13}$C NMR** (100 MHz, $CDCl_3$) δ 163.7 (t, *J* = 34.4

Hz), 134.5 (t, $J$ = 9.3 Hz), 124.8 (t, $J$ = 25.3 Hz), 111.9 (t, $J$ = 248.0 Hz), 83.1, 63.1, 44.8, 38.7, 33.9, 21.9, 21.6, 13.9, 11.6, 11.5; **$^{19}$F NMR** (376.5 MHz, $CDCl_3$) δ -103.6 (at, $J$ = 10.5 Hz, 2F).

**Minor diastereomer: $^1$H NMR** (400 MHz, $CDCl_3$) δ 6.10–6.03 (m, 1H), 5.89–5.72 (m, 1H), 4.85–4.80 (m, 1H), 4.33 (q, $J$ = 7.1 Hz, 2H), 3.00 (s, 3H), 2.74–2.54 (m, 2H), 1.59–1.39 (m, 5H), 1.35 (t, $J$ = 7.1 Hz, 3H), 0.95 (t, $J$ = 7.4 Hz, 6H); **$^{13}$C NMR** (100 MHz, $CDCl_3$) δ 163.7 (t, $J$ = 34.4 Hz), 136.6 (t, $J$ = 6.7 Hz), 123.6 (t, $J$ = 26.2 Hz), 112.7 (t, $J$ = 249.8 Hz), 83.9, 63.2, 45.3, 38.8, 33.9, 22.1, 21.6, 13.9, 11.7, 11.5; **$^{19}$F NMR** (376.5 MHz, $CDCl_3$) δ -99.7 (at, $J$ = 16.6 Hz, 2F).

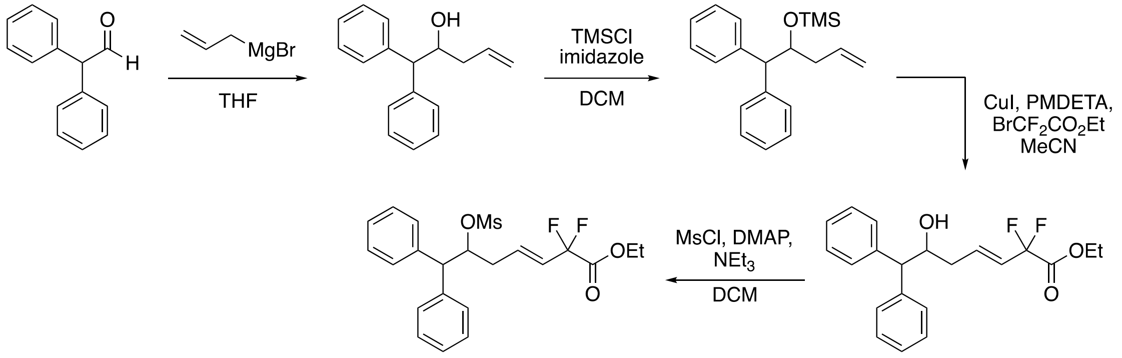


**Scheme SI-18:** Synthesis of Mesylate **SI-22**

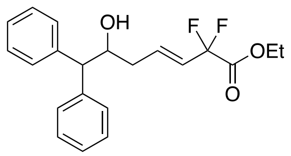

**Alcohol (SI-41)** was prepared according to Method B. The following amounts of reagents were used: silylated alcohol (0.57 g, 1.8 mmol, 1.0 equiv), CuI (35 mg, 0.18 mmol, 10. mol %), PMDETA (0.57 mL, 2.7 mmol, 1.5 equiv), ethyl bromodifluoroacetate (0.35 mL, 2.7 mmol, 1.5 equiv), and MeCN (9.1 mL, 0.20 M in substrate). The compound was purified by flash column chromatography (0–20% EtOAc/hexanes) to afford the title compound as a mixture of alkene diastereomers as a yellow oil (102 mg, 0.28 mmol, 16%, 4.9:1 dr). **TLC R$_f$** = 0.3 (20% EtOAc/hexanes); **HRMS** (TOF MS ES+) *m/z*: $[M + Na]^+$ calculated for $C_{21}H_{22}F_2O_3Na$, 383.1435; found 383.1427. For clarity, the $^1$H NMR and $^{13}$C NMR data of the major and minor diastereomers have been tabulated individually.

**Major diastereomer: $^1$H NMR** (500 MHz, $CDCl_3$) δ 7.37–7.19 (m, 10H), 6.41–6.37 (m, 1H), 5.70–5.62 (m, 1H), 4.44 (br s, 1H), 4.29 (q, $J$ = 7.2 Hz, 2H), 3.86 (d, $J$ = 8.8 Hz, 1H), 2.42–2.35 (m, 1H), 2.24–2.20 (m, 1H), 1.81 (br s, 1H), 1.32 (t, $J$ = 7.3 Hz, 3H); **$^{13}$C NMR** (125.7 MHz, $CDCl_3$) δ 164.1 (t, $J$ = 34.6 Hz), 141.7, 140.9, 136.1 (t, $J$ = 9.2 Hz), 129.0 (2C), 128.9 (2C), 128.8 (2C), 128.3 (2C), 127.2, 126.94, 123.6 (t, $J$ = 25.0 Hz), 112.2 (t, $J$ = 247.7 Hz), 72.7, 63.0, 58.4, 37.4, 14.0.

**Minor diastereomer: $^1$H NMR** (500 MHz, $CDCl_3$) δ 7.37–7.19 (m, 10H), 6.15–6.10 (m, 1H), 5.70–5.62 (m, 1H), 4.44 (br s, 1H), 4.21 (q, $J$ = 7.1 Hz, 2H), 3.89 (d, $J$ = 8.5 Hz, 1H), 2.42–2.35 (m, 1H), 2.24–2.20 (m, 1H), 1.78 (br s, 1H), 1.25 (t, $J$ = 7.2 Hz, 3H); **$^{13}$C NMR** (125.7 MHz, $CDCl_3$) δ 164.2 (t, $J$ = 33.8 Hz), 141.8, 141.1, 138.6 (t, $J$ = 6.9 Hz), 129.0 (2C), 128.9 (2C), 128.8

(2C), 128.3 (2C), 127.1, 126.86, 122.6 (t, $J$ = 26.2 Hz), 112.9 (t, $J$ = 249.4 Hz), 73.3, 63.1, 58.8, 34.1, 13.9.

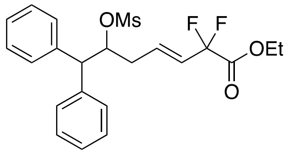


**Mesylate (SI-22)** was prepared according to Method C. The following amounts of reagents were used: alcohol **SI-41** (0.10 g, 0.28 mmol, 1.0 equiv), $Et_3N$ (60. µL, 0.43 mmol, 1.5 equiv), DMAP (3.5 mg, 28 µmol, 0.10 equiv), MsCl (30. µL, 0.43 mmol, 1.5 equiv), and DCM (1.4 mL, 0.20 M in substrate). The compound was purified by flash column chromatography (0–20% EtOAc/hexanes) to afford the title compound as a mixture of alkene diastereomers as a dark yellow oil (0.11 g, 0.25 mmol, 91%, 5.4:1 dr). **TLC R$_f$** = 0.3 (20% EtOAc/hexanes); **HRMS** (TOF MS ES+) *m/z*: $[M + NH_4]^+$ calculated for $C_{22}H_{24}F_2O_5SNH_4$, 456.1656; found 456.1660. For clarity, the $^1$H NMR, $^{13}$C NMR, and $^{19}$F NMR data of the major and minor diastereomers have been tabulated individually.

**Major diastereomer: $^1$H NMR** (400 MHz, $CDCl_3$) δ 7.42–7.21 (m, 10H), 6.38–6.31 (m, 1H), 5.61 (dt, $J$ = 15.7, 11.0 Hz, 1H), 5.48–5.43 (m, 1H), 4.32 (q, $J$ = 7.1 Hz, 2H), 4.10 (d, $J$ = 9.8 Hz, 1H), 2.79–2.46 (m, 2H), 2.19 (s, 3H), 1.34 (t, $J$ = 7.1 Hz, 3H); **$^{13}$C NMR** (100 MHz, $CDCl_3$) δ 163.8 (t, $J$ = 34.4 Hz), 140.2, 139.6, 132.7 (t, $J$ = 9.3 Hz), 129.2 (2C), 128.9 (2C), 128.8 (2C), 128.4 (2C), 127.6, 127.5, 125.8 (t, $J$ = 25.3 Hz), 112.0 (t, $J$ = 248.2 Hz), 84.0, 63.1, 55.3, 37.7, 36.5, 14.0; **$^{19}$F NMR** (376.5 MHz, $CDCl_3$) δ -103.7 (dd, $J$ = 41.2, 10.0 Hz, 2F).

**Minor diastereomer: $^1$H NMR** (400 MHz, $CDCl_3$) δ 7.42–7.21 (m, 10H), 6.17–6.10 (m, 1H), 5.61 (dt, $J$ = 15.7, 11.0 Hz, 1H), 5.48–5.43 (m, 1H), 4.24 (q, $J$ = 7.1 Hz, 2H), 4.16 (d, $J$ = 9.6 Hz, 1H), 2.79–2.46 (m, 2H), 2.13 (s, 3H), 1.27 (t, $J$ = 7.1 Hz, 3H); **$^{13}$C NMR** (100 MHz, $CDCl_3$) δ 163.8 (t, $J$ = 34.4 Hz), 140.5, 139.8, 135.4 (t, $J$ = 6.8 Hz), 129.2 (2C), 128.9 (2C), 128.8 (2C), 128.4 (2C), 127.6, 127.5, 123.9 (t, $J$ = 26.4 Hz), 112.0 (t, $J$ = 248.2 Hz), 85.2, 68.6, 56.5, 37.7, 36.5, 13.9; **$^{19}$F NMR** (376.5 MHz, $CDCl_3$) δ -100.1 (dd, $J$ = 32.5, 14.8 Hz, 2F).

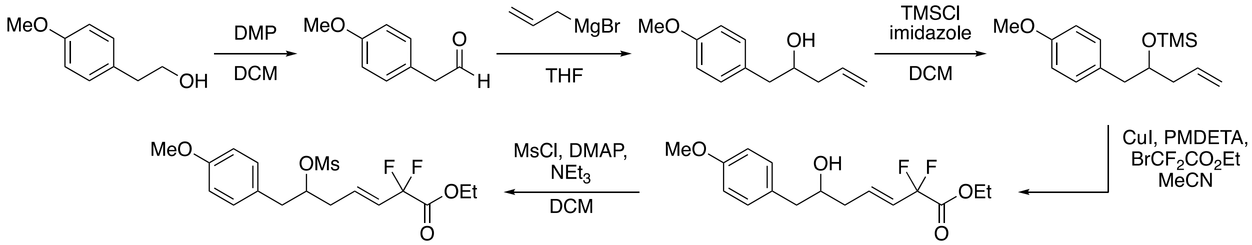


**Scheme SI-19:** Synthesis of Mesylate **SI-23**

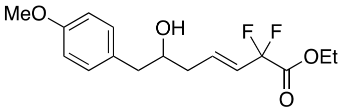


**Alcohol (SI-42)** was prepared according to Method B. The following amounts of reagents were used: silylated alcohol (1.0 g, 3.9 mmol, 1.0 equiv), CuI (75 mg, 0.39 mmol, 10. mol %), PMDETA (1.2 mL, 5.9 mmol, 1.5 equiv), ethyl bromodifluoroacetate (0.75 mL, 5.9 mmol, 1.5 equiv), and MeCN (20. mL, 0.20 M in substrate). The compound was purified by flash column

chromatography (0–30% EtOAc/hexanes) to afford the title compound as a mixture of alkene diastereomers as a yellow oil (0.22 g, 0.69 mmol, 18%, 2.6:1 dr). **TLC R$_f$** = 0.3 (25% EtOAc/hexanes, CAM stain); **HRMS** (TOF MS ES+) *m/z*: $[M + Na]^+$ calculated for $C_{16}H_{20}F_2O_4Na$, 337.1227; found 337.1233. For clarity, the $^1$H NMR, $^{13}$C NMR, and $^{19}$F NMR data of the major and minor diastereomers have been tabulated individually.

**Major diastereomer: $^1$H NMR** (600 MHz, $CDCl_3$) δ 7.09 (d, *J* = 8.6 Hz, 2H), 6.83 (d, *J* = 8.6 Hz, 2H), 6.37–6.32 (m, 1H), 5.80–5.67 (m, 1H), 4.29 (q, *J* = 7.1 Hz, 2H), 3.87–3.81 (m, 1H), 3.75 (s, 3H), 2.7 (dd, *J* = 13.5, 5.0 Hz, 1H), 2.68–2.61 (m, 1H), 2.58–2.43 (m, 1H), 2.36–2.24 (m, 2H), 1.31 (t, *J* = 7.1 Hz, 3H); **$^{13}$C NMR** (150.9 MHz, $CDCl_3$) δ 164.1 (t, *J* = 34.7 Hz), 158.3, 136.2 (t, *J* = 9.1 Hz), 130.3 (2C), 129.8, 123.4 (t, *J* = 24.9 Hz), 113.93 (2C), 112.1 (t, *J* = 247.7 Hz), 71.3, 62.9, 55.1, 42.5, 38.8, 13.79; **$^{19}$F NMR** (125.7 MHz, $CDCl_3$) δ -103.0 (at, *J* = 7.0 Hz, 2F).

**Minor diastereomer: $^1$H NMR** (600 MHz, $CDCl_3$) δ 7.09 (d, *J* = 8.6 Hz, 2H), 6.83 (d, *J* = 8.6 Hz, 2H), 6.12–6.08 (m, 1H), 5.80–5.67 (m, 1H), 4.29 (q, *J* = 7.1 Hz, 2H), 3.87–3.81 (m, 1H), 3.75 (s, 3H), 2.7 (dd, *J* = 13.5, 5.0 Hz, 1H), 2.68–2.61 (m, 1H), 2.58–2.43 (m, 1H), 2.36–2.24 (m, 2H), 1.31 (t, *J* = 7.1 Hz, 3H); **$^{13}$C NMR** (150.9 MHz, $CDCl_3$) δ 163.9 (t, *J* = 34.6 Hz), 158.3, 138.3 (t, *J* = 6.9 Hz), 130.3 (2C), 130.0, 122.5 (t, *J* = 26.2 Hz), 113.92 (2C), 112.8 (t, *J* = 249.0 Hz), 71.9, 63.0, 55.1, 42.7, 35.4, 13.77; **$^{19}$F NMR** (125.7 MHz, $CDCl_3$) δ -98.9 (d, *J* = 14.7 Hz, 2F).

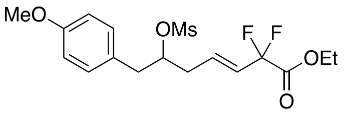


**Mesylate (SI-23)** was prepared according to Method C. The following amounts of reagents were used: alcohol **SI-42** (0.22 g, 0.69 mmol, 1.0 equiv), $Et_3N$ (0.14 mL, 1.0 mmol, 1.5 equiv), DMAP (8.4 mg, 69 μmol, 0.10 equiv), MsCl (80. μL, 1.0 mmol, 1.5 equiv), and DCM (3.5 mL, 0.20 M in substrate). The compound was purified by flash column chromatography (0–25% EtOAc/hexanes) to afford the title compound as a mixture of alkene diastereomers as a yellow oil (0.22 g, 0.57 mmol, 82%, 9% EtOAc by NMR, 2.6:1 dr). **TLC R$_f$** = 0.4 (25% EtOAc/hexanes, CAM stain); **HRMS** (TOF MS ES+) *m/z*: $[M + Na]^+$ calculated for $C_{17}H_{22}F_2O_6SNa$, 415.1003; found 415.0991. For clarity, the $^1$H NMR, $^{13}$C NMR, and $^{19}$F NMR data of the major and minor diastereomers have been tabulated individually.

**Major diastereomer: $^1$H NMR** (600 MHz, $CDCl_3$) δ 7.14 (d, *J* = 8.5 Hz, 2H), 6.85 (d, *J* = 8.6 Hz, 2H), 6.30 (dt, *J* = 15.2, 7.6 Hz, 1H), 5.86–5.76 (m, 1H), 4.83 (aquint, *J* = 6.2 Hz, 1H), 4.31 (q, *J* = 6.9 Hz, 2H), 3.77 (s, 3H), 2.95–2.88 (m, 2H), 2.78–2.49 (m, 2H), 2.58 (s, 3H), 1.33 (t, *J* = 7.3 Hz, 3H); **$^{13}$C NMR** (150.9 MHz, $CDCl_3$) δ 163.5 (t, *J* = 34.3 Hz), 158.8, 133.4 (t, *J* = 9.3 Hz), 130.6 (2C), 127.9, 125.2 (t, *J* = 25.2 Hz), 114.0 (2C), 111.8 (t, *J* = 248.0 Hz), 82.4, 63.1, 55.1, 39.7, 37.8, 36.8, 13.78; **$^{19}$F NMR** (564.6 MHz, $CDCl_3$) δ -103.4 (at, *J* = 12.2 Hz, 2F).

**Minor diastereomer: $^1$H NMR** (600 MHz, $CDCl_3$) δ 7.14 (d, *J* = 8.5 Hz, 2H), 6.85 (d, *J* = 8.6 Hz, 2H), 6.11–6.06 (m, 1H), 5.86–5.76 (m, 1H), 4.83 (aquint, *J* = 6.2 Hz, 1H), 4.31 (q, *J* = 6.9 Hz, 2H), 3.77 (s, 3H), 2.95–2.88 (m, 2H), 2.78–2.49 (m, 2H), 2.58 (s, 3H), 1.33 (t, *J* = 7.3 Hz, 3H); **$^{13}$C NMR** (150.9 MHz, $CDCl_3$) δ 163.7 (t, *J* = 34.4 Hz), 158.8, 135.4 (t, *J* = 6.7 Hz), 130.6 (2C), 128.2, 123.9 (t, *J* = 26.3 Hz), 114.0 (2C), 112.6 (t, *J* = 249.1 Hz), 83.1, 63.2, 55.1, 39.9, 37.7, 33.5, 13.76; **$^{19}$F NMR** (564.6 MHz, $CDCl_3$) δ -99.3 (at, *J* = 14.2 Hz, 2F).

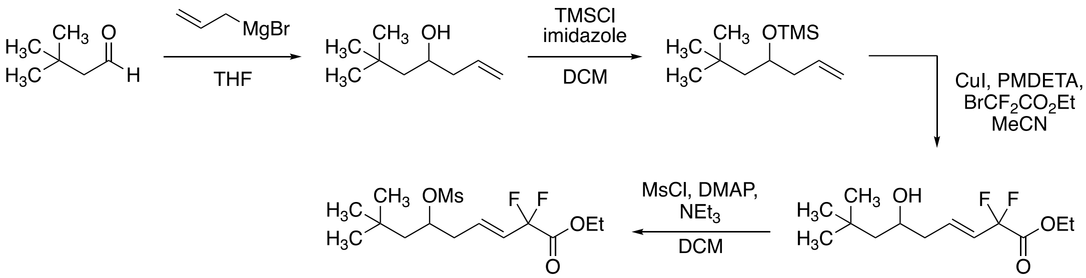


**Scheme SI-20:** Synthesis of Mesylate **SI-24**

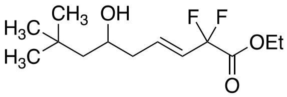

**Alcohol (SI-43)** was prepared according to Method B. The following amounts of reagents were used: silylated alcohol (0.63 g, 2.9 mmol, 1.0 equiv), CuI (57 mg, 0.30 mmol, 10. mol %), PMDETA (0.92 mL, 4.4 mmol, 1.5 equiv), ethyl bromodifluoroacetate (0.56 mL, 4.4 mmol, 1.5 equiv), and MeCN (15 mL, 0.20 M in substrate). The compound was purified by flash column chromatography (0–20% EtOAc/hexanes) to afford the title compound as a mixture of alkene diastereomers as a yellow oil (57 mg, 0.22 mmol, 7%, 8.9:1 dr). **TLC R$_f$** = 0.5 (20% EtOAc/hexanes, CAM stain); **HRMS** (TOF MS CI+) *m/z*: $[M + H]^+$ calculated for $C_{13}H_{22}F_2O_3H$, 265.1615; found 265.1618. For clarity, the $^1$H NMR data of the major and minor diastereomers have been tabulated individually.

**Major diastereomer: $^1$H NMR** (400 MHz, $CDCl_3$) δ 6.35–6.27 (m, 1H), 5.82–5.66 (m, 1H), 4.32 (q, *J* = 7.1 Hz, 2H), 3.90–3.85 (m, 1H), 2.31–2.27 (m, 2H), 1.65 (br s, 1H), 1.39–1.33 (m, 2H), 1.35 (t, *J* = 7.2 Hz, 3H), 0.96 (s, 9H).

**Minor diastereomer: $^1$H NMR** (400 MHz, $CDCl_3$) δ 6.11–6.04 (m, 1H), 5.82–5.66 (m, 1H), 4.32 (q, *J* = 7.1 Hz, 2H), 3.90–3.85 (m, 1H), 2.49–2.43 (m, 1H), 2.31–2.27 (m, 1H), 1.65 (br s, 1H), 1.39–1.33 (m, 2H), 1.35 (t, *J* = 7.2 Hz, 3H), 0.96 (s, 9H).

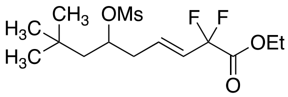

**Mesylate (SI-24)** was prepared according to Method C. The following amounts of reagents were used: alcohol **SI-43** (57 mg, 0.22 mmol, 1.0 equiv), $Et_3N$ (60. µL, 0.33 mmol, 1.5 equiv), DMAP (2.6 mg, 22 µmol, 0.10 equiv), MsCl (30. µL, 0.33 mmol, 1.5 equiv), and DCM (1.1 mL, 0.20 M in substrate). The compound was purified by flash column chromatography (0–20% EtOAc/hexanes) to afford the title compound as a mixture of alkene diastereomers as a yellow oil (71 mg, 0.21 mmol, 95%, 5.7:1 dr). **TLC R$_f$** = 0.5 (20% EtOAc/hexanes, CAM stain); **HRMS** (TOF MS CI+) *m/z*: $[M + H]^+$ calculated for $C_{14}H_{24}F_2O_5SH$, 343.1391; found 343.1391. For clarity, the $^1$H NMR, $^{13}$C NMR, and $^{19}$F NMR data of the major and minor diastereomers have been tabulated individually.

**Major diastereomer: $^1$H NMR** (600 MHz, $CDCl_3$) δ 6.30–6.25 (m, 1H), 5.86–5.75 (m, 1H), 4.96 (br s, 1H), 4.33 (q, *J* = 7.1 Hz, 2H), 3.01 (s, 3H), 2.74–2.71 (m, 1H), 2.55–2.53 (m, 1H), 1.70 (dd, *J* = 15.1, 7.6 Hz, 1H), 1.45 (dd, *J* = 15.1, 3.4 Hz, 1H), 1.35 (t, *J* = 7.1 Hz, 3H), 0.97 (s, 9H); **$^{13}$C NMR** (150.9 MHz, $CDCl_3$) δ 163.8 (t, *J* = 34.4 Hz), 133.3 (t, *J* = 9.2 Hz), 125.4 (t, *J* = 25.2 Hz),

111.9 (t, $J$ = 248.1 Hz), 78.5, 63.2, 52.6, 47.4, 39.3, 30.2, 29.7 (3C), 14.0; **$^{19}$F NMR** (125.7 MHz, $CDCl_3$) δ -103.5 (d, $J$ = 10.6 Hz, 2F).

**Minor diastereomer: $^1$H NMR** (600 MHz, $CDCl_3$) δ 6.06–6.02 (m, 1H), 5.86–5.75 (m, 1H), 4.96 (br s, 1H), 4.33 (q, $J$ = 7.1 Hz, 2H), 3.01 (s, 3H), 2.74–2.71 (m, 1H), 2.55–2.53 (m, 1H), 1.70 (dd, $J$ = 15.1, 7.6 Hz, 1H), 1.45 (dd, $J$ = 15.1, 3.4 Hz, 1H), 1.35 (t, $J$ = 7.1 Hz, 3H), 0.97 (s, 9H); **$^{13}$C NMR** (150.9 MHz, $CDCl_3$) δ 163.9 (t, $J$ = 34.5 Hz), 135.4 (t, $J$ = 6.7 Hz), 124.0 (t, $J$ = 26.2 Hz), 112.7 (t, $J$ = 249.3 Hz), 79.2, 63.3, 52.6, 47.6, 39.1, 30.2, 29.8 (3C), 14.0; **$^{19}$F NMR** (125.7 MHz, $CDCl_3$) δ -99.4 (at, $J$ = 12.7 Hz, 2F).

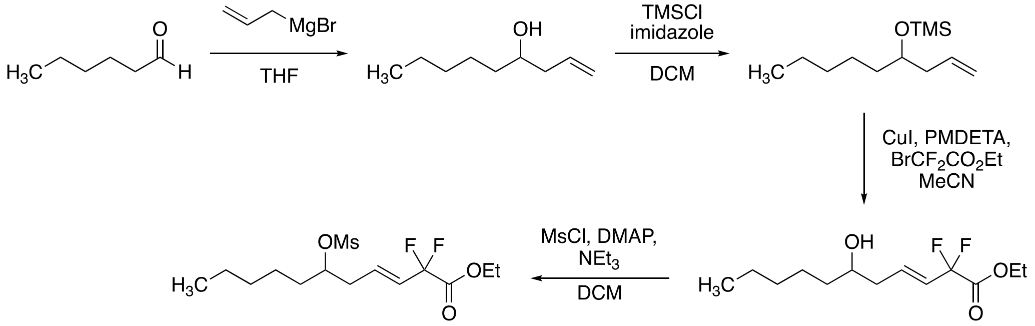


**Scheme SI-21:** Synthesis of Mesylate **SI-25**

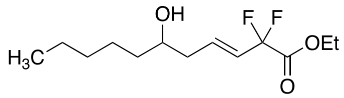

**Alcohol (SI-44)** was prepared according to Method B. The following amounts of reagents were used: silylated alcohol (0.64 g, 3.0 mmol, 1.0 equiv), CuI (57 mg, 0.30 mmol, 10. mol %), PMDETA (0.94 mL, 4.5 mmol, 1.5 equiv), ethyl bromodifluoroacetate (0.58 mL, 4.5 mmol, 1.5 equiv), and MeCN (15 mL, 0.20 M in substrate). The compound was purified by flash column chromatography (0–40% EtOAc/hexanes) to afford the title compound as a 2.5:1 mixture of alkene diastereomers as a yellow oil (0.34 g, 1.3 mmol, 43%). For clarity, the $^1$H NMR data of the major and minor diastereomers have been tabulated individually.

**Major Diastereomer: $^1$H NMR** (400 MHz, $CDCl_3$) δ 6.38–6.27 (m, 1H), 5.85–5.65 (m, 1H), 4.33 (q, $J$ = 7.1 Hz, 2H), 3.72 (br s, 1H), 2.59–2.20 (m, 2H), 1.68–1.51 (m, 1H), 1.51–1.41 (m, 3H), 1.39–1.23 (m, 8H), 0.90 (t, $J$ = 6.8 Hz, 3H).

**Minor Diastereomer: $^1$H NMR** (400 MHz, $CDCl_3$) δ 6.15–6.05 (m, 1H), 5.85–5.65 (m, 1H), 4.33 (q, $J$ = 7.1 Hz, 2H), 3.72 (br s, 1H), 2.59–2.20 (m, 2H), 1.68–1.51 (m, 1H), 1.51–1.41 (m, 3H), 1.39–1.23 (m, 8H), 0.90 (t, $J$ = 6.8 Hz, 3H).

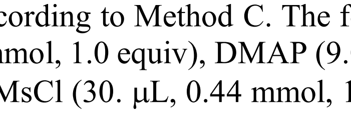

**Mesylate (SI-25)** was prepared according to Method C. The following amounts of reagents were used: alcohol **SI-44** (97 mg, 0.37 mmol, 1.0 equiv), DMAP (9.0 mg, 0.44 mmol, 20. mol %), $Et_3N$ (0.10 mL, 0.74 mmol, 2.0 equiv), MsCl (30. μL, 0.44 mmol, 1.2 equiv), and DCM (2.0 mL, 0.19 M in substrate). The compound was purified by flash column chromatography (0–20%

EtOAc/hexanes) to afford the title compound as a 2.5:1 mixture of alkene diastereomers as a yellow oil (95 mg, 0.28 mmol, 75%). **TLC $R_f$** = 0.5 (20% EtOAc/hexanes); **HRMS** (TOF MS CI+) *m/z*: $[M + NH_4]^+$ calculated for $C_{14}H_{24}F_2O_5SNH_4$, 360.1656; found 360.1646. For clarity, the $^1$H NMR, $^{13}$C NMR, and $^{19}$F NMR data of the major and minor diastereomers have been tabulated individually.

**Major Diastereomer: $^1$H NMR** (500 MHz, $CDCl_3$) δ 6.31–6.19 (m, 1H), 5.87–5.69 (m, 1H), 4.75 (quint, *J* = 6.3 Hz, 1H), 4.31 (q, *J* = 7.1 Hz, 2H), 2.97 (s, 3H), 2.77–2.46 (m, 2H), 1.77–1.57 (m, 2H), 1.46–1.21 (m, 9H), 0.88 (t, *J* = 6.6 Hz, 3H); **$^{13}$C NMR** (125.7 MHz, $CDCl_3$) δ 163.7 (t, *J* = 34.4 Hz), 133.6 (t, *J* = 9.3 Hz), 125.2 (t, *J* = 25.5 Hz), 111.9 (t, *J* = 248.3 Hz), 81.2, 63.2, 38.7, 37.1, 34.5, 34.3, 31.4, 24.6, 22.4, 13.9 **$^{19}$F NMR** (376.5, $CDCl_3$) δ -103.6 to -103.7 (m, 2F).

**Minor Diastereomer: $^1$H NMR** (500 MHz, $CDCl_3$) δ 6.08–5.97 (m, 1H), 5.87–5.69 (m, 1H), 4.75 (quint, *J* = 6.3 Hz, 1H), 4.31 (q, *J* = 7.1 Hz, 2H), 2.97 (s, 3H), 2.77–2.46 (m, 2H), 1.77–1.57 (m, 2H), 1.46–1.21 (m, 9H), 0.88 (t, *J* = 6.6 Hz, 3H); **$^{13}$C NMR** (125.7 MHz, $CDCl_3$) δ 163.7 (t, *J* = 34.4 Hz), 135.6 (t, *J* = 6.7 Hz), 124.0 (t, *J* = 26.4 Hz), 111.9 (t, *J* = 248.3 Hz), 81.9, 63.2, 38.7, 37.1, 35.2, 33.4, 31.4, 24.6, 22.4, 13.9; **$^{19}$F NMR** (376.5, $CDCl_3$) δ -99.7 (d, *J* = 13.8 Hz, 2F).

2) Intermediates and Mesylates for Enantioenriched Cyclopropanes

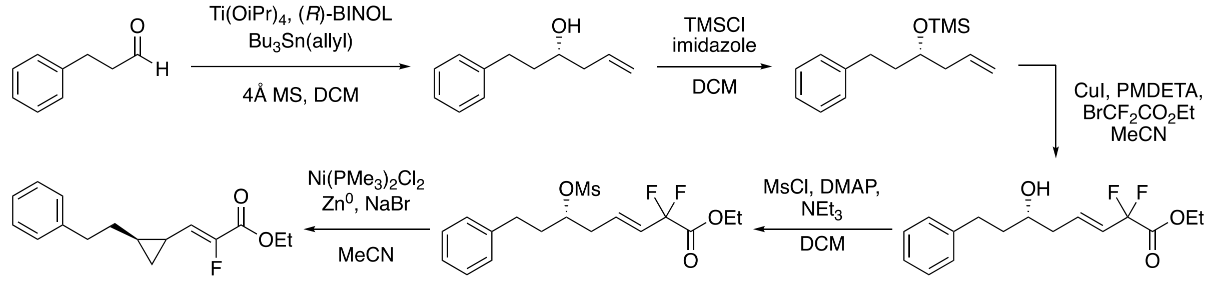


**Scheme SI-22:** Synthesis of Enantioenriched Mesylate (+)-**SI-12**

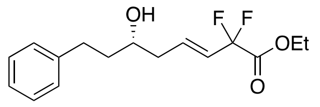

**Alcohol** (+)-**SI-31** was prepared according to Method B. The following amounts of reagents were used: enantioenriched silylated alcohol (0.74 g, 3.0 mmol, 1.0 equiv), CuI (57 mg, 0.30 mmol, 10. mol %), PMDETA (0.94 mL, 4.5 mmol, 1.5 equiv), ethyl bromodifluoroacetate (0.58 mL, 4.5 mmol, 1.5 equiv), and MeCN (15 mL, 0.20 M in substrate). The compound was purified by flash column chromatography (0–40% EtOAc/hexanes) to afford the title compound as a 4:1 mixture of alkene diastereomers as a yellow oil (0.31 g, 1.1 mmol, 35%). Refer to alcohol **SI-31** above for analytical data. **$[\alpha]^{25}_D$** +1.9 (*c* 1.05 mg/mL $CHCl_3$); **SFC Analysis** (Chiralcel OD-H, 4.0% IPA/$CO_2$, 2.0 mL/min, 210 nm) indicated 90% ee: $t_R$ (minor diastereomer, major enantiomer) = 12.9 minutes, $t_R$ (major diastereomer, major enantiomer) = 14.6 minutes, $t_R$ (minor diastereomer, minor enantiomer) = 20.6 minutes, $t_R$ (major diastereomer, minor enantiomer) = 21.3 minutes.

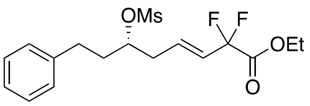

**Enantioenriched mesylate SI-12** was prepared according to Method C. The following amounts of reagents were used: enantioenriched alcohol (+)-**SI-31** (0.10 g, 0.34 mmol, 1.0 equiv), $Et_3N$ (90. µL, 0.68 mmol, 2.0 equiv), DMAP (9.0 mg, 70. µmol, 0.20 equiv), MsCl (30. µL, 0.41 mmol, 1.2 equiv), and DCM (2.0 mL, 0.17 M in substrate). The compound was purified by flash column chromatography (0–30% EtOAc/hexanes) to afford the title compound as a 4:1 mixture of alkene diastereomers as a yellow oil (55 mg, 0.15 mmol, 43%). Refer to mesylate **SI-12** above for analytical data.

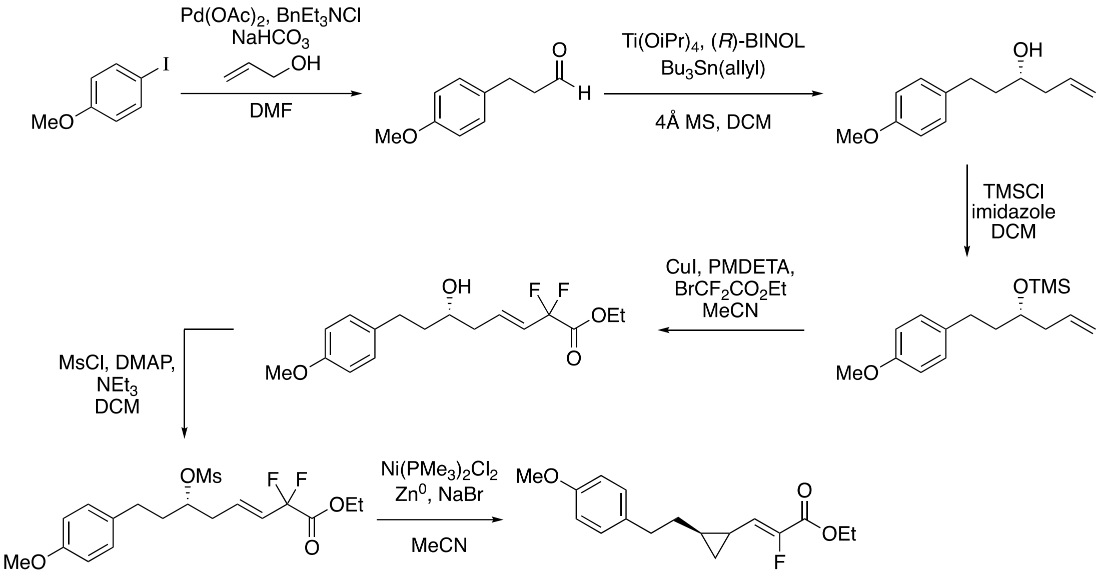


**Scheme SI-23:** Synthesis of Enantioenriched Mesylate **SI-13**

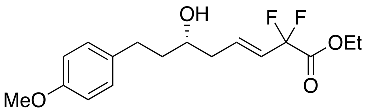

**Enantioenriched alcohol (SI-32)** was prepared according to Method B. The following amounts of reagents were used: enantioenriched silylated alcohol (1.2 g, 4.4 mmol, 1.0 equiv, 0.10 M in MeCN), CuI (84 mg, 0.44 mmol, 10. mol %), PMDETA (1.4 mL, 6.6 mmol, 1.5 equiv), ethyl bromodifluoroacetate (0.84 mL, 6.6 mmol, 1.5 equiv), and MeCN (22 mL, 0.20 M in substrate).The compound was purified by flash column chromatography (0–30% EtOAc/hexanes) to afford the title compound as a mixture of alkene diastereomers as a yellow oil (0.22 g, 0.66 mmol, 15%, 2.8:1 dr). Refer to alcohol **SI-32** above for analytical data.

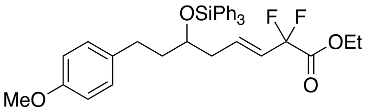

**Triphenylsilylated alcohol (SI-45)** was prepared according to Method D to determine the enantiomeric excess of alcohol **SI-32**. The following amounts of reagents were used: alcohol **SI-32** (80. mg, 0.25 mmol, 1.0 equiv), $Et_3N$ (50. µL, 0.29 mmol, 1.2 equiv), DMAP (4.9 mg, 49 µmol, 0.20 equiv), $Ph_3SiCl$ (87 mg, 0.29 mmol, 1.2 equiv), and DCM (1.2 mL, 0.20 M in substrate). The

compound was purified by flash column chromatography (0–20% EtOAc/hexanes) to afford the title compound as a mixture of alkene diastereomers as a yellow oil (38 mg, 64 μmol, 26%, 3.5:1 dr). **TLC R$_f$** = 0.5 (20% EtOAc/hexanes, CAM stain); **SFC Analysis** (Chiralcel OD-H, 1.5% IPA/$CO_2$, 2.7 mL/min, 210 nm) indicated 2% ee: $t_R$ (major diastereomer, major enantiomer) = 63.1 minutes, $t_R$ (major diastereomer, minor enantiomer) = 67.9 minutes. For clarity, the $^1$H NMR data of the major and minor diastereomers have been tabulated individually.

**Major Diastereomer: $^1$H NMR** (400 MHz, $CDCl_3$) δ 7.61 (d, *J* = 6.6 Hz, 6H), 7.45–7.35 (m, 9H), 6.86 (d, *J* = 8.3 Hz, 2H), 6.74 (d, *J* = 8.4 Hz, 2H), 6.27–6.24 (m, 1H), 5.69–5.57 (m, 1H), 4.24 (q, *J* = 7.2 Hz, 2H), 4.00–3.97 (m, 1H), 3.76 (s, 3H), 2.56–2.38 (m, 4H), 1.80–1.76 (m, 2H), 1.28–1.23 (m, 3H).

**Minor Diastereomer: $^1$H NMR** (400 MHz, $CDCl_3$) δ 7.61 (d, *J* = 6.6 Hz, 6H), 7.45–7.35 (m, 9H), 6.86 (d, *J* = 8.3 Hz, 2H), 6.74 (d, *J* = 8.4 Hz, 2H), 6.08–6.01 (m, 1H), 5.69–5.57 (m, 1H), 4.24 (q, *J* = 7.2 Hz, 2H), 4.00–3.97 (m, 1H), 3.76 (s, 3H), 2.56–2.38 (m, 4H), 1.80–1.76 (m, 2H), 1.28–1.23 (m, 3H).

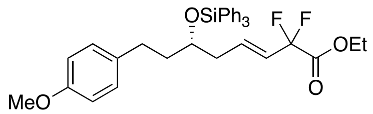


**Triphenylsilylated alcohol** (+)-**SI-45** was prepared according to Method D to determine the enantiomeric excess of enantioenriched alcohol **SI-32**. The following amounts of reagents were used: enantioenriched alcohol **SI-32** (47 mg, 0.14 mmol, 1.0 equiv), $Et_3N$ (30. μL, 0.17 mmol, 1.2 equiv), DMAP (2.9 mg, 28 μmol, 0.20 equiv), $Ph_3SiCl$ (50. mg, 0.17 mmol, 1.2 equiv), and DCM (0.71 mL, 0.20 M in substrate). The compound was purified by flash column chromatography (0–20% EtOAc/hexanes) to afford the title compound as a mixture of alkene diastereomers as a yellow oil (74 mg, 0.13 mmol, 88%, 7.8:1 dr). Refer to silyl ether **SI-45** above for analytical data. **$[\alpha]^{25}_D$** +16.2° (c 10.8 mg/1.5 mL $CHCl_3$); **SFC Analysis** (Chiralcel OD-H, 1.5% IPA/$CO_2$, 2.7 mL/min, 210 nm) indicated 91% ee: $t_R$ (major diastereomer, major enantiomer) = 62.7 minutes, $t_R$ (major diastereomer, minor enantiomer) = 68.7 minutes.

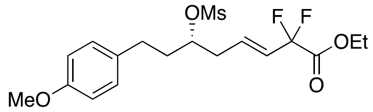


**Enantioenriched mesylate SI-13** was prepared according to Method C. The following amounts of reagents were used: enantioenriched alcohol **SI-32** (0.21 g, 0.65 mmol, 1.0 equiv), $Et_3N$ (0.14 mL, 0.97 mmol, 1.5 equiv), DMAP (7.9 mg, 65 μmol, 0.10 equiv), MsCl (80. μL, 0.97 mmol, 1.5 equiv), and DCM (3.3 mL, 0.20 M in substrate). The compound was purified by flash column chromatography (0–30% EtOAc/hexanes) to afford the title compound as a mixture of alkene diastereomers as a yellow oil (0.23 g, 0.56 mmol, 86%, 2.9:1 dr). Refer to mesylate **SI-13** above for analytical data.

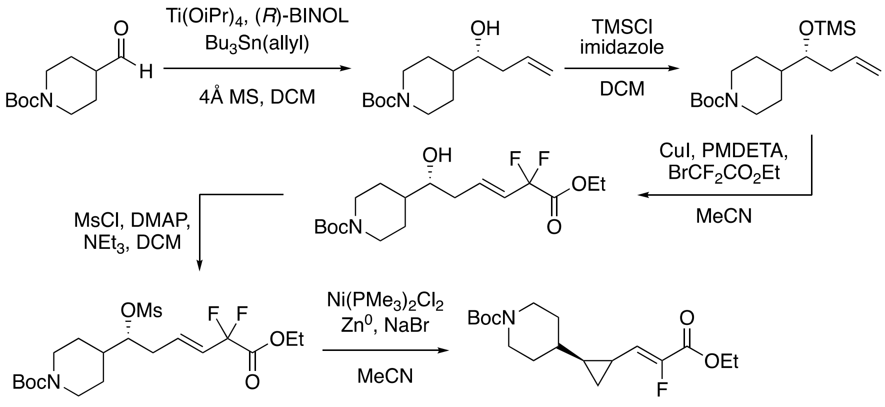


**Scheme SI-24:** Synthesis of Enantioenriched Mesylate **SI-18**

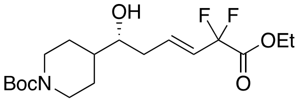

**Enantioenriched alcohol (SI-37)** was prepared according to Method B. The following amounts of reagents were used: enantioenriched silylated alcohol (1.0 g, 3.1 mmol, 1.0 equiv, 0.10 M in MeCN), CuI (59 mg, 0.31 mmol, 10. mol %), PMDETA (0.97 mL, 4.7 mmol, 1.5 equiv), ethyl bromodifluoroacetate (0.60 mL, 4.7 mmol, 1.5 equiv), and MeCN (16 mL, 0.20 M in substrate). The compound was purified by flash column chromatography (0–30% EtOAc/hexanes) to afford the title compound as a mixture of alkene diastereomers as a yellow oil (0.45 g, 1.2 mmol, 38%, 3.6:1 dr). Refer to alcohol **SI-37** above for analytical data.

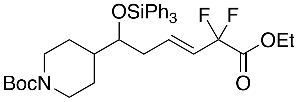

**Triphenylsilylated alcohol** (**SI-46)** was prepared according to Method D to determine the enantiomeric excess of alcohol **SI-37**. The following amounts of reagents were used: alcohol **SI-37** (42 mg, 0.11 mmol, 1.0 equiv), $Et_3N$ (20. *μ*L, 0.13 mmol, 1.2 equiv), DMAP (2.7 mg, 22 μmol, 0.20 equiv), $Ph_3SiCl$ (39 mg, 0.13 mmol, 1.2 equiv), and DCM (0.55 mL, 0.20 M in substrate). The compound was purified by flash column chromatography (0–20% EtOAc/hexanes) to afford the title compound as a mixture of alkene diastereomers as a yellow oil (46 mg, 72 μmol, 66%, 5:1 dr). **TLC $R_f$** = 0.4 (20% EtOAc/hexanes); **SFC Analysis** (Chiralcel OD-H, 1.5% IPA/$CO_2$, 2.7 mL/min, 210 nm) indicated 1% ee: $t_R$ (major diastereomer, major enantiomer) = 63.3 minutes, $t_R$ (major diastereomer, minor enantiomer) = 68.6 minutes. For clarity, the $^1$H NMR data of the major and minor diastereomers have been tabulated individually.

**Major Diastereomer: $^1$H NMR** (400 MHz, $CDCl_3$) δ 7.60 (d, *J* = 6.7 Hz, 6H), 7.45–7.36 (m, 9H), 6.25–6.18 (m, 1H), 5.66–5.50 (m, 1H), 4.24 (q, *J* = 7.1 Hz, 2H), 4.07 (br s, 2H), 3.78–3.76 (m, 1H), 2.54–2.44 (m, 2H), 2.34 (br s, 2H), 1.64–1.61 (m, 1H), 1.54–1.48 (m, 2H), 1.44 (s, 9H), 1.28 (t, *J* = 7.1 Hz, 3H), 1.24–1.14 (m, 2H).

**Minor Diastereomer: $^1$H NMR** (400 MHz, $CDCl_3$) δ 7.60 (d, *J* = 6.7 Hz, 6H), 7.45–7.36 (m, 9H), 6.03–5.98 (m, 1H), 5.66–5.50 (m, 1H), 4.24 (q, *J* = 7.1 Hz, 2H), 4.07 (br s, 2H), 3.78–3.76 (m, 1H), 2.54–2.44 (m, 2H), 2.34 (br s, 2H), 1.64–1.61 (m, 1H), 1.54–1.48 (m, 2H), 1.44 (s, 9H), 1.28 (t, *J* = 7.1 Hz, 3H), 1.24–1.14 (m, 2H).

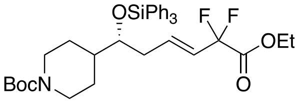

**Triphenylsilylated alcohol** (+)-**SI-46** was prepared according to Method D to determine the enantiomeric excess of enantioenriched alcohol **SI-37**. The following amounts of reagents were used: enantioenriched alcohol **SI-37** (18 mg, 48 µmol, 1.0 equiv), $Et_3N$ (8.0 µL, 57 µmol, 1.2 equiv), DMAP (1.2 mg, 9.5 µmol, 0.20 equiv), $Ph_3SiCl$ (17. mg, 57 µmol, 1.2 equiv), and DCM (0.24 mL, 0.20 M in substrate). The compound was purified by flash column chromatography (0–20% EtOAc/hexanes) to afford the title compound as a mixture of alkene diastereomers as a yellow oil (26 mg, 40. µmol, 84%, 3.1:1 dr). Refer to silyl ether **SI-46** above for analytical data. **$[\alpha]^{25}_D$** +8.1° (c 10.9 mg/1.5 mL $CHCl_3$); **SFC Analysis** (Chiralcel OD-H, 1.5% IPA/$CO_2$, 2.7 mL/min, 210 nm) indicated 91% ee: $t_R$ (major diastereomer, minor enantiomer) = 65.3 minutes, $t_R$ (major diastereomer, major enantiomer) = 69.4 minutes.

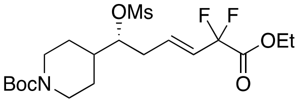

**Enantioenriched Mesylate SI-18** was prepared according to Method C. The following amounts of reagents were used: enantioenriched alcohol **SI-37** (0.41 g, 1.1 mmol, 1.0 equiv), $Et_3N$ (0.23 mL, 1.7 mmol, 1.5 equiv), DMAP (13 mg, 0.11 mmol, 0.10 equiv), MsCl (0.13mL, 1.7 mmol, 1.5 equiv), and DCM (5.5 mL, 0.20 M in substrate). The compound was purified by flash column chromatography (0–30% EtOAc/hexanes) to afford the title compound as a mixture of alkene diastereomers as a viscous yellow oil (0.39 g, 0.85 mmol, 77%, 3:1 dr). Refer to mesylate **SI-18** above for analytical data.

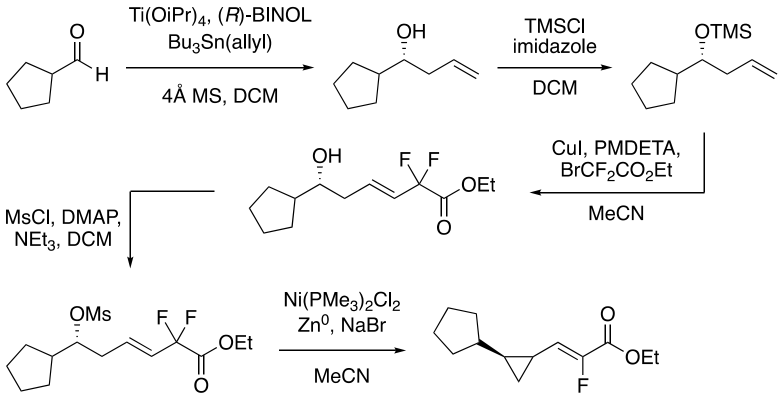


**Scheme SI-25:** Synthesis of Enantioenriched Mesylate **SI-20**

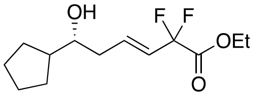

**Enantioenriched alcohol (SI-39)** was prepared according to Method B. The following amounts of reagents were used: enantioenriched silylated alcohol (0.64 g, 3.0 mmol, 1.0 equiv), CuI (57 mg, 0.30 mmol, 10. mol %), PMDETA (0.94 mL, 4.5 mmol, 1.5 equiv), ethyl bromodifluoroacetate (0.58 mL, 4.5 mmol, 1.5 equiv), and MeCN (15 mL, 0.20 M in substrate). The compound was purified by flash column chromatography (0–40% EtOAc/hexanes) to afford

the title compound as a 6:1 mixture of alkene diastereomers as a yellow oil (0.29 g, 1.1 mmol, 37%). Refer to alcohol **SI-38** above for analytical data.

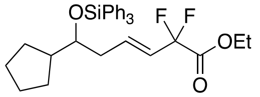

**Triphenylsilylated alcohol (SI-47)** was prepared according to Method D to determine the enantiomeric excess of alcohol **SI-39**. The following amounts of reagents were used: alcohol **SI-39** (69 mg, 0.26 mmol, 1.0 equiv), $Et_3N$ (40. μL, 0.31 mmol, 1.2 equiv), DMAP (4.8 mg, 39 μmol, 0.20 equiv), $Ph_3SiCl$ (91 mg, 0.31 mmol, 1.2 equiv), and DCM (1.0 mL, 0.26 M in substrate). The compound was purified by flash column chromatography (0–20% EtOAc/hexanes) to afford the title compound in a single diastereomers as a yellow oil (21 mg, 42 μmol, 16%). **TLC $R_f$** = 0.6 (20% EtOAc/hexanes); **$^1$H NMR** (500 MHz, $CDCl_3$) δ 7.70–7.65 (d, *J* = 6.9 Hz, 6H), 7.56–7.41 (m, 9H), 6.39–6.27 (m, 1H), 5.70–5.59 (m, 1H), 4.32 (q, *J* = 7.2 Hz, 2H), 3.86 (q, *J* = 5.7 Hz, 1H), 2.40 (br s, 2H), 2.12–2.02 (m, 1H), 1.83–1.75 (m, 2H), 1.60–1.46 (m, 4H), 1.42–1.30 (m, 4H), 1.27–1.18 (m, 1H); **HPLC Analysis** (Chiralcel OD, 0.5% IPA/hexanes, 2.0 mL/min, 230 nm) indicated 0% ee: $t_R$ (minor diastereomer, major enantiomer) = 9.8 minutes, $t_R$ (minor diastereomer, minor enantiomer) = 10.3 minutes, $t_R$ (major diastereomer, major enantiomer) = 11.3 minutes, $t_R$ (major diastereomer, minor enantiomer) = 12.8 minutes.

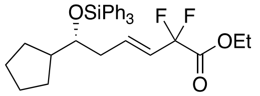

**Triphenylsilylated alcohol (–)-SI-47** was prepared according to Method D to determine the enantiomeric excess of enantioenriched alcohol **SI-39**. The following amounts of reagents were used: enantioenriched alcohol **SI-39** (20. mg, 80. μmol, 1.0 equiv), $Et_3N$ (20. μL, 0.10 mmol, 1.2 equiv), DMAP (2.0 mg, 16 μmol, 0.20 equiv), $Ph_3SiCl$ (30. mg, 0.10 mmol, 1.2 equiv), and DCM (1.0 mL, 0.08 M in substrate). The compound was purified by flash column chromatography (0–20% EtOAc/hexanes) to afford the title compound in a single diastereomer as a yellow oil (5.0 mg, 9.6 μmol, 12%). Refer to silyl ether **SI-47** above for analytical data. **$[\alpha]^{25}_D$** –7.3 (*c* 1.09 mg/mL $CHCl_3$); **HPLC Analysis** (Chiralcel OD, 0.5% IPA/hexanes, 2.0 mL/min, 230 nm) indicated 80% ee: $t_R$ (minor diastereomer, major enantiomer) = 9.3 minutes, $t_R$ (minor diastereomer, minor enantiomer) = 10.0 minutes, $t_R$ (major diastereomer, major enantiomer) = 10.6 minutes, $t_R$ (major diastereomer, minor enantiomer) = 13.2 minutes.

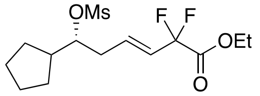

**Enantioenriched mesylate SI-20** was prepared according to Method C. The following amounts of reagents were used: enantioenriched alcohol **SI-39** (0.1 g, 0.40 mmol, 1.0 equiv), $Et_3N$ (0.11 mL, 0.80 mmol, 2.0 equiv), DMAP (10. mg, 80. μmol, 0.20 equiv), MsCl (40. μL, 0.48 mmol, 1.2 equiv), and DCM (2.0 mL, 0.20 M in substrate). The compound was purified by flash column chromatography (0–30% EtOAc/hexanes) to afford the title compound as a 6:1 mixture of alkene diastereomers as a yellow oil (90. mg, 0.26 mmol, 66%). Refer to mesylate **SI-20** above for analytical data.

## G. Characterization Data of Products from Derivatization of Cyclopropane 4:

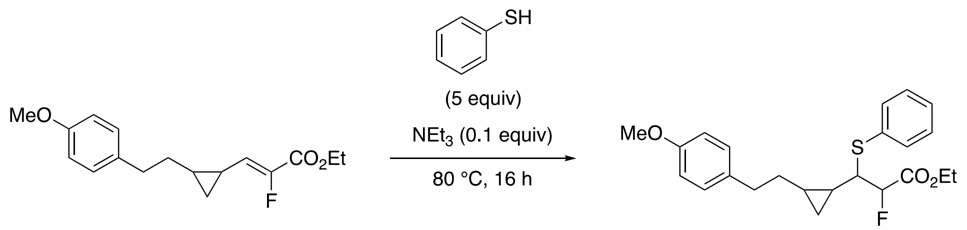


**Thioether (17)** The following procedure was adapted from Couve-Bonnaire.[xviii] To a flame-dried 7 mL vial equipped with a septa cap and a stir bar was added cyclopropane **4** (28 mg, 96 $\mu$mol, 1.0 equiv), thiophenol (50 $\mu$L, 0.48 mmol, 5.0 equiv), and $NEt_3$ (1.3 $\mu$L, 9.6 $\mu$mol, 0.10 equiv). The reaction mixture was heated to 80 °C and allowed to stir for 16 h. The reaction mixture was allowed to cool to rt. EtOAc was added to dilute the reaction mixture, and the reaction mixture was directly loaded onto a flash column for purification (0–20% EtOAc/hexanes) to yield the title compound as a mixture of diastereomers as a colorless oil (13 mg, 33 $\mu$mol, 34%). The title compound was characterized as a mixture of two cyclopropane diastereomers. **TLC $R_f$** = 0.4 (10% EtOAc/hexanes, CAM stain); **$^1$H NMR** (500 MHz, $CDCl_3$) δ 7.54–7.47 (m, 4H, both diastereomers), 7.30–7.26 (m, 6H, both diastereomers), 7.10–7.05 (m, 4H, both diastereomers), 6.81 (at, $J$ = 8.1 Hz, 4H, both diastereomers), 5.04 (dd, $J$ = 48.2, 3.3 Hz, 2H, both diastereomers), 4.25–3.98 (m, 4H, both diastereomers), 3.78 (s, 6H, both diastereomers), 3.16–3.00 (m, 2H, both diastereomers), 2.87–2.55 (m, 4H, both diastereomers), 1.93–1.86 (m, 1H, one diastereomer), 1.61–1.57 (m, 1H, one diastereomer), 1.54–1.47 (m, 1H, one diastereomer), 1.44–1.28 (m, 1H, other diastereomer), 1.26–1.21 (m, 6H, both diastereomers), 1.13–0.72 (m, 5H, both diastereomers), 0.55–0.49 (m, 1H, one diastereomer), 0.47–0.44 (m, 1H, one diastereomer), 0.09–0.04 (m, 1H, other diastereomer); **$^{13}$C NMR** (125.7 MHz, $CDCl_3$) δ 168.2 (d, $J$ = 42.5 Hz, one diastereomer), 168.0 (d, $J$ = 36.6 Hz, other diastereomer), 157.9 (one diastereomer), 157.8 (other diastereomer), 134.6 (2C, both diastereomers), 134.4 (2C, both diastereomers), 133.8 (4C, both diastereomers), 129.4 (4C, both diastereomers), 129.0 (4C, both diastereomers), 127.9 (2C, both diastereomers), 113.8 (4C, both diastereomers), 91.1 (d, $J$ = 190.1 Hz, 2C, both diastereomers), 61.8 (2C, both diastereomers), 56.5 (d, $J$ = 19.9 Hz, 2C, both diastereomers), 55.4 (2C, both diastereomers), 36.0 (one diastereomer), 35.3 (one diastereomer), 34.6 (other diastereomer), 30.7 (other diastereomer), 21.5 (one diastereomer), 20.0 (other diastereomer), 17.4 (2C, both diastereomers), 14.2 (2C, both diastereomers), 13.2 (2C, both diastereomers); **$^{19}$F NMR** (564.6 MHz, $CDCl_3$) δ -195.6 (dd, $J$ = 48.1, 29.5 Hz, 2F, both diastereomers); **HRMS** (TOF MS ES+) *m/z*: $[M + Na]^+$ calculated for $C_{23}H_{27}FO_3SNa$, 425.1563; found 425.1567.

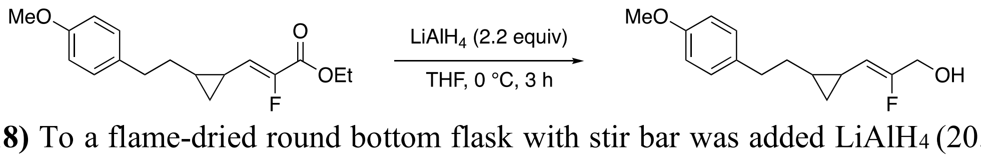


**Alcohol (18)** To a flame-dried round bottom flask with stir bar was added $LiAlH_4$ (20. mg, 0.52 mmol, 2.2 equiv) in the glovebox. The flask was sealed with a septum, then brought outside of the glovebox. THF (1.2 mL, 0.20 M in substrate) was added to the flask. The flask was cooled to 0 °C, and then cyclopropane **4** (69 mg, 0.24 mmol, 1.0 equiv, 0.10 M in THF) was added dropwise. The reaction mixture was allowed to stir for 3 h at 0 °C. To quench, a vent needle was added and 1 M HCl was added dropwise at 0 °C until the bubbling stopped. The reaction mixture was then extracted with $Et_2O$ (3 x 20 mL). The combined organic layers were washed with brine, dried over

$Na_2SO_4$, and concentrated in vacuo. The residue was loaded onto a silica plug and flushed with 100% $Et_2O$ to afford the title compound as a mixture of cyclopropane diastereomers as a colorless oil (43 mg, 0.17 mmol, 70%, 1.4:1 dr trans:cis). **TLC** $\mathbf{R_f}$ = 0.3 (20% EtOAc/hexanes, $KMnO_4$ stain); **HRMS** (TOF MS ES+) *m/z*: $[M + Na]^+$ calculated for $C_{15}H_{19}FO_2Na$, 273.1267; found 273.1269. For clarity, the $^1H$ NMR data of the major and minor diastereomers have been tabulated individually.

**Major diastereomer:** $\mathbf{^1H}$ **NMR** (400 MHz, $CDCl_3$) δ 7.08 (d, *J* = 8.5 Hz, 2H), 6.81 (d, *J* = 8.3 Hz, 2H), 4.31 (dd, *J* = 35.7, 9.7 Hz, 1H), 4.05 (d, *J* = 16.8 Hz, 2H), 3.77 (s, 3H), 2.64 (t, *J* = 7.6 Hz, 2H), 2.18 (br s, 1H), 1.60–1.53 (m, 2H), 1.04–0.88 (m, 1H), 0.79–0.71 (m, 1H), 0.60–0.50 (m, 2H).

**Minor diastereomer:** $\mathbf{^1H}$ **NMR** (400 MHz, $CDCl_3$) δ 7.09 (d, *J* = 8.5 Hz, 2H), 6.81 (d, *J* = 8.3 Hz, 2H), 4.51 (dd, *J* = 35.6, 9.7 Hz, 1H), 4.06 (d, *J* = 16.4 Hz, 2H), 3.77 (s, 3H), 2.64 (t, *J* = 7.6 Hz, 2H), 2.18 (br s, 1H), 1.76–1.64 (m, 1H), 1.60–1.53 (m, 1H), 1.44–1.38 (m, 2H), 1.04–0.88 (m, 1H), 0.17–0.13 (m, 1H).

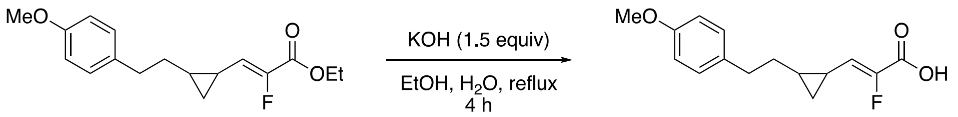


**Carboxylic acid (19)** The following procedure was adapted from Piva.[xix] To a round-bottom flask with stir bar was added cyclopropane **4** (36 mg, 0.12 mmol, 1.0 equiv), KOH (10. mg, 0.19 mmol, 1.5 equiv), and 95:5 EtOH/$H_2O$ (0.20 M in substrate). A reflux condenser was attached, and the reaction mixture was allowed to stir at reflux for 4 h. Once the reaction mixture was cooled to rt, the mixture was acidified with 1 M HCl. The mixture was extracted with hexanes, dried over $Na_2SO_4$, filtered, and concentrated in vacuo to afford the title compound as a mixture of cyclopropane diastereomers as an orange oil (26 mg, 0.10 mmol, 80%, 1:1 dr). **HRMS** (TOF MS ES+) *m/z*: $[M + Na]^+$ calculated for $C_{15}H_{17}FO_3Na$, 287.1060; found 287.1054. For clarity, the $^1H$ NMR, $^{13}C$ NMR, and $^{19}F$ NMR data of the major and minor diastereomers have been tabulated individually.

**Major diastereomer:** $\mathbf{^1H}$ **NMR** (600 MHz, $CDCl_3$) δ 7.71 (br s, 1H), 7.08 (d, *J* = 7.7 Hz, 2H), 6.81 (d, *J* = 8.6 Hz, 2H), 5.74 (dd, *J* = 31.3, 10.7 Hz, 1H), 3.78 (s, 3H), 2.68–2.60 (m, 2H), 1.80–1.72 (m, 1H), 1.68–1.55 (m, 2H), 1.23–1.15 (m, 1H), 0.88–0.82 (m, 2H); $\mathbf{^{13}C}$ **NMR** (150.9 MHz, $CDCl_3$) δ 165.5 (d, *J* = 34.6 Hz), 157.9, 146.7 (d, *J* = 249.2 Hz), 133.9, 129.5 (2C), 125.9 (d, *J* = 9.1 Hz), 114.0 (2C), 55.4, 34.7, 35.0, 21.1, 15.9, 13.2; $\mathbf{^{19}F}$ **NMR** (564.7 MHz, $CDCl_3$) δ -136.9 (d, *J* = 30.9 Hz, 1F).

**Minor diastereomer:** $\mathbf{^1H}$ **NMR** (600 MHz, $CDCl_3$) δ 7.71 (br s, 1H), 7.08 (d, *J* = 7.7 Hz, 2H), 6.82 (d, *J* = 8.6 Hz, 2H), 5.94 (dd, *J* = 31.1, 11.1 Hz, 1H), 3.77 (s, 3H), 2.68–2.60 (m, 2H), 1.94–1.85 (m, 1H), 1.68–1.55 (m, 2H), 1.36–1.28 (m, 1H), 1.09–1.01 (m, 1H), 0.51–0.47 (m, 1H); $\mathbf{^{13}C}$ **NMR** (150.9 MHz, $CDCl_3$) δ 165.8 (d, *J* = 34.5 Hz), 157.9, 147.8 (d, *J* = 249.0 Hz), 133.9, 129.4 (2C), 129.1 (d, *J* = 10.5 Hz), 113.9 (2C), 55.4, 35.7, 34.6, 22.9, 16.1, 13.2; $\mathbf{^{19}F}$ **NMR** (564.7 MHz, $CDCl_3$) δ -138.4 (d, *J* = 31.2 Hz, 1F).

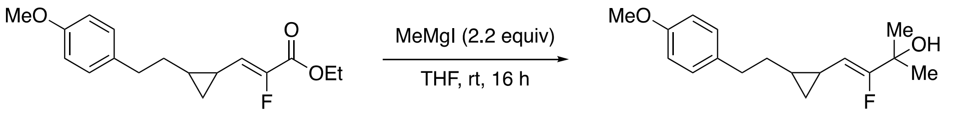


**Alcohol (20)** To a 7-mL vial equipped with a stir bar was added cyclopropane **4** (31 mg, 0.11 mmol, 1.0 equiv) and anhydrous THF (1.0 mL, 0.11 M in substrate). MeMgI (80. µL, 0.23 mmol, 2.2 equiv, 2.9 M in $Et_2O$) was added via syringe over 30 sec and allowed to stir at rt for 16 h. The reaction was quenched with MeOH and filtered through a plug of silica gel (100% $Et_2O$). The products were directly loaded onto a flash column for purification (0–20% EtOAc/hexanes) to yield the title compound in a mixture of cyclopropane diastereomers as a colorless oil (18 mg, 68 *µ*mol, 62%, 1:1 dr). **TLC $R_f$** = 0.4 (20% EtOAc/hexanes); **HRMS** (TOF MS CI+) *m/z*: $[M]^+$ calculated for $C_{17}H_{23}FO_2$, 278.1682; found 278.1680. For clarity, the $^1H$ NMR, $^{13}C$ NMR, and $^{19}F$ NMR data of the major and minor diastereomers have been tabulated individually.

**Major diastereomer: $^1$H NMR** (400 MHz, $CDCl_3$) δ 7.13–7.06 (m, 2H), 6.86–6.80 (m, 2H), 4.36 (dd, *J* = 37.0, 9.6 Hz, 1H), 3.80 (s, 3H), 2.70–2.60 (m, 2H), 1.80 (br s, 1H), 1.64–1.51 (m, 2H), 1.41–1.37 (m, 6H), 1.06–0.82 (m, 1H), 0.79–0.69 (m, 1H), 0.62–0.48 (m, 2H). **$^{13}$C NMR** (150.9 MHz, $CDCl_3$) δ 162.5 (d, *J* = 254.3 Hz), 157.72, 134.4, 129.4 (2C), 113.7 (2C), 107.2 (d, *J* = 13.8 Hz), 55.3, 36.0, 34.7, 27.3 (2C), 20.3, 17.6, 13.9, 13.4; **$^{19}$F NMR** (564.7 MHz, $CDCl_3$) δ -127.6 (d, *J* = 36.8 Hz, 1F).

**Minor diastereomer: $^1$H NMR** (400 MHz, $CDCl_3$) δ 7.13–7.06 (m, 2H), 6.86–6.80 (m, 2H), 4.58 (dd, *J* = 36.9, 9.4 Hz, 1H), 3.80 (s, 3H), 2.70–2.60 (m, 2H), 1.78 (br s, 1H), 1.75–1.69 (m, 1H), 1.64–1.51 (m, 2H), 1.33–1.29 (m, 6H), 1.06–0.82 (m, 2H), 0.15 (q, *J* = 5.2 Hz, 1H). **$^{13}$C NMR** (150.9 MHz, $CDCl_3$) δ 164.1 (d, *J* = 256.5 Hz), 157.75, 134.5, 129.4 (2C), 113.7 (2C), 103.0 (d, *J* = 12.7 Hz), 55.3, 35.0, 32.1, 27.4 (2C), 18.4, 18.3, 11.0, 11.1; **$^{19}$F NMR** (564.7 MHz, $CDCl_3$) δ -125.4 (d, *J* = 36.8 Hz, 1F).

## III. Computational Data for Mechanistic Studies

A) Computational Methods

All density functional theory (DFT) calculations presented in the manuscript were performed using B3LYP[xx]-D3BJ[xxi]def2TZVP[xxii]/PCM//B3LYP-D3[xxiii]/*def2-SVP*[xxiv]/SMD[xxv] (solvent = acetonitrile) level of theory as implemented in *Gaussian 16.*[xxvi] Ground state geometry optimization and transition structures (TSs) were located at the B3LYP-D3/def2-SVP level of theory and a SMD solvent model for acetonitrile was utilized. All starting materials and intermediates were verified as true minima whereas all the transition structures were characterized by one imaginary frequency. Single point energy calculations were performed using the B3LYP functional with Grimme's dispersion correction with Becke-Johnson damping (D3-BJ) and a triple-ζ basis set *def2*-TZVP and a PCM[xxvii] solvent model for acetonitrile. Gibbs free energies were estimated at the reaction temperature of 298 K and were corrected using Grimme's quasi-RRHO approach.[xxviii] Intermediates along the reaction coordinate were located by performing a quick reaction coordinate analysis (QRC) from the respective transition structures. IRC calculations followed by a geometry optimization were utilized to confirm TSs and local minima of all structures in the relevant pathways (Figures 2 and 3 in the manuscript). A thorough conformational search was performed for all the TSs to identify the lowest energy conformation. Cartesian coordinates of all computed starting materials, intermediates, and transition structures are included in an attached

xyz file. The higher energy conformations for each TS are also included along with the relative energies. For benchmarking purposes, all the lowest energy transition structures and intermediate geometries were calculated using a variety of computational methods for the single point energies. The single point methods include the following: B3LYP-D3(BJ)/*def2*-TZVP/SMD (acetonitrile), M06-L[xxix]/*def2*-TZVP/SMD (acetonitrile) and wB97-XD[xxx]/*def2*-TZVP/SMD (acetonitrile). For all methods the relative energies of the surface for each pathway remain consistent (i.e., the turn-over limiting and stereoselective steps are similar for each method). The oxidative addition steps for both Pathway 2 and Pathway 3 are similar in energy for all methods. The method published herein – B3LYP-D3(BJ)/*def2*-TZVP/PCM (acetonitrile) – lies within the average of the methods explored (**Section 5**).

For a systematic search on the cross-electrophile coupling reaction of allylic *gem*-difluorides we utilized mesylate **1a** (from the manuscript) as the model substrate. Various transition structures were explored for each of the different steps involved in the reaction pathway using B3LYP-D3/*def2SVP* SMD (acetonitrile) level of theory as implemented in *Gaussian 16.* The array of structures arises from the possibilities of (1) a syn or anti arrangement of the fluorine and carbonyl group, (2) a syn or anti arrangement of the cyclohexyl and methanesulfonyl group, and 3) various rotational orientations of the mesylate group. Overall, 145 unique transition structures and intermediates were identified for this reaction.

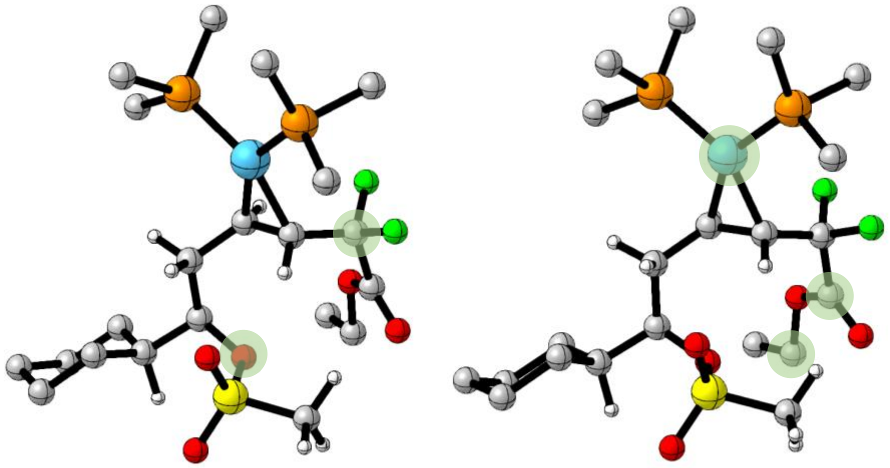

**Fig SI-1: Showing the rotatable bonds about which the conformational search was performed.**

B. Exploration of Oxidative Addition Transition Structures

1. Stereoinvertive vs. Stereoretentive Oxidative Addition of Ni(0)$L_2$ into the Secondary Alkyl Mesylate

**Pathway # 1A OA *vs* Pathway #2 OA**

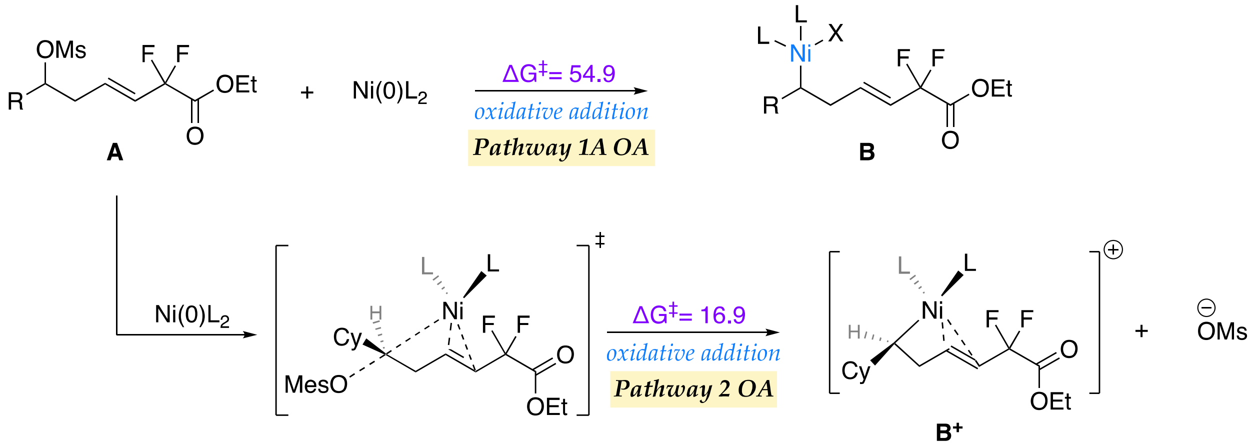


The lowest energy transition structure for stereoinvertive OA is shown in Figure 2 of the manuscript (**TS**$_{\textbf{OA-Inv}}$). An alternative stereoretentive OA into the mesylate center was calculated to be much higher in energy $\Delta G^{\ddagger} = 54.9$ kcal/mol.

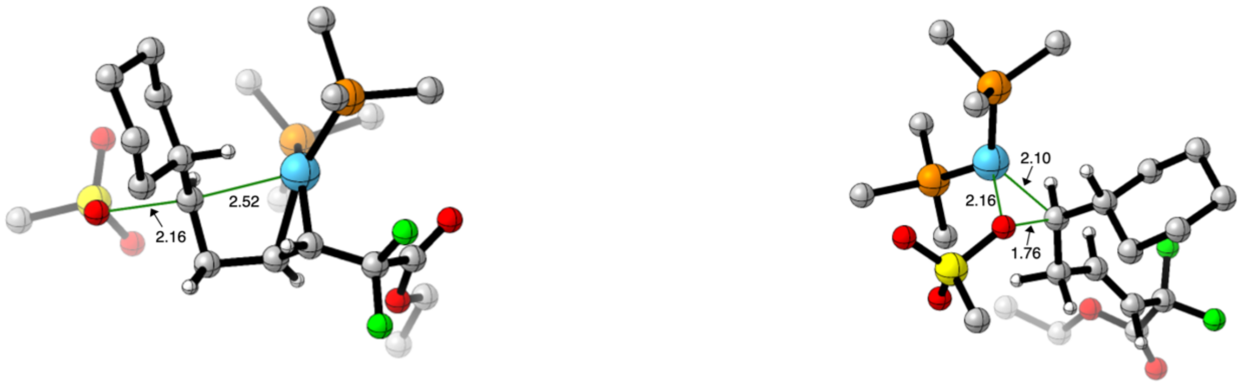


**TS**$_{\textbf{OA-Inv}}$
$\Delta G^{\ddagger} = +16.9$

**TS**$_{\textbf{OA-Ret}}$
$\Delta G^{\ddagger} = +54.9$

**Fig SI-2: Computed lowest-lying TSs for oxidative addition into the mesylate for Pathway #1A OA (stereoretentive) and Pathway #2 OA (stereoinvertive)**

2. <u>Oxidative Addition of Ni(0)L2 into the Allylic *gem*-difluoride and at the Secondary Alkyl Center</u>

**Pathway #3: Oxidative addition step via Ni(0)$L_2$ into the allylic gem-difluoride**

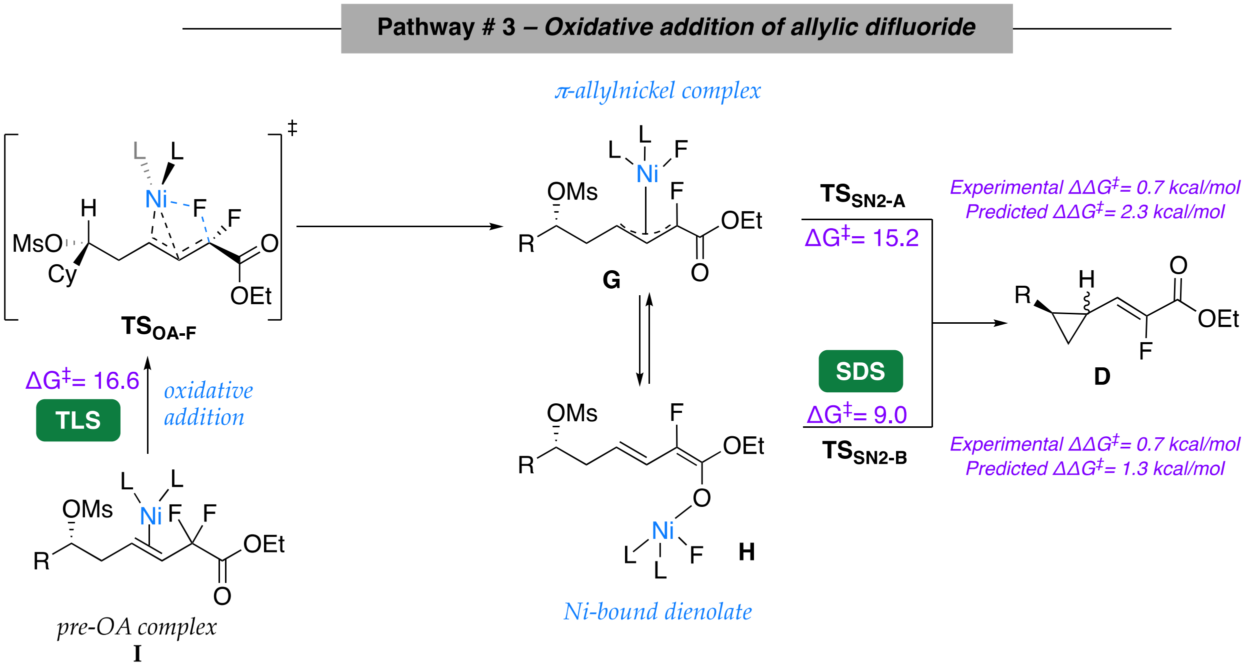


All transition structures were calculated relative to the pre-OA complex I. Generation of the pre-OA complex I from separate starting material is an exergonic process ($\Delta G^{\ddagger}$ = -30.8 kcal/mol), downhill relative to separated starting material. A total of 19 transition structures were calculated for the activation of allylic *gem*-difluorides by Ni(0)$L_2$ complex. A conformational search involved rotation along the cyclohexylmethyl methanesulfonate and ethyldifluoroacetate bonds. The lowest energy transition structure (**TS$_{OA\text{-}F}$**) for oxidative addition of Ni(0)$L_2$ into the gem-difluoride is $\Delta G^{\ddagger}$ = 16.6 kcal/mol from the pre-OA complex **I**. The lowest-energy transition structure **TS$_{OA\text{-}F}$** has a syn-arrangement of fluorine and carbonyl in the ethyl-difluoride moiety. All TSs within 3.0 kcal/mol of **TS$_{OA\text{-}F}$** were Boltzmann weighted to give the final energy used in the potential energy surface of the manuscript (Figure 3). A second conformer with a similar orientation on the cyclohexylmethyl moiety with an anti-arrangement of the fluorine and carbonyl **TS$_{OA\text{-}F17}$** was slightly higher in energy by 0.6 kcal/mol. Various conformers were located which are significantly higher in energy.

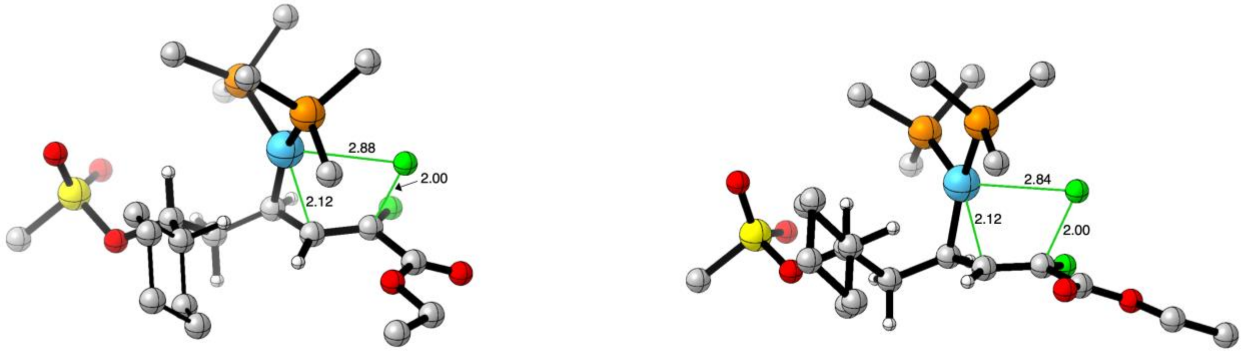


**TS**$_{OA\text{-}F}$ $\Delta G^{\ddagger}$ = *+16.6*  **TS**$_{OA\text{-}F17}$ $\Delta G^{\ddagger}$ = *+17.2*

**Fig SI-3: Computed lowest energy TSs for the oxidative addition step of Ni(0)L$_2$ into the *gem*-difluoride (Pathway #3)**

**Pathway #3: Oxidative addition step of Ni(0)L$_2$ into the *gem*-difluoride via coordination of the catalyst to the carbonyl (Pathway #3)**

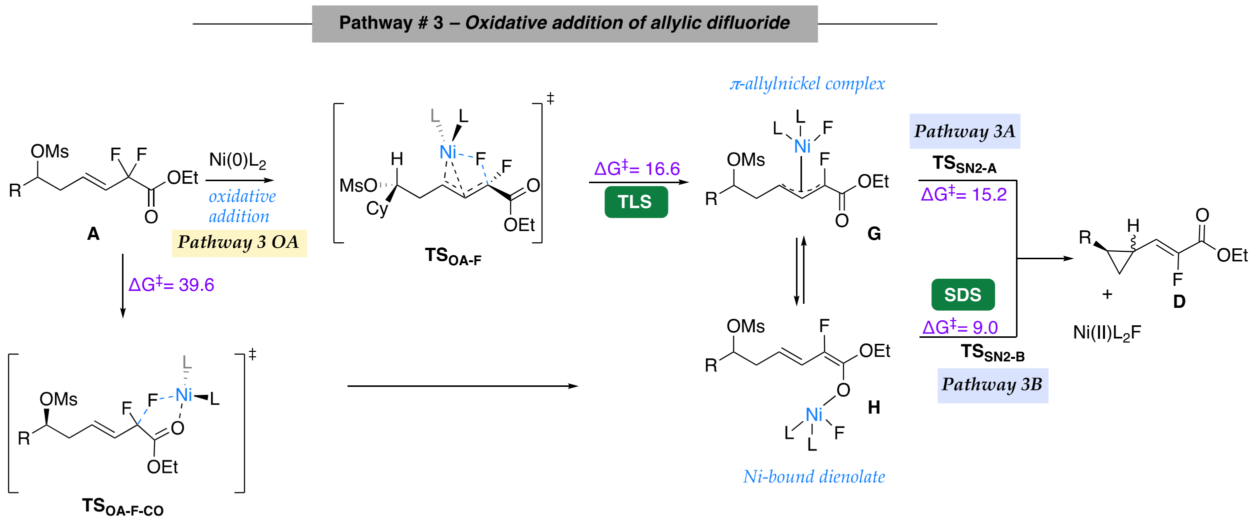


An alternate pathway for the formation of Ni-bound dienolate intermediate **G** was also investigated which can subsequently undergo S$_N$2 type ring-closure forming vinyl cyclopropane product **D**. We explored oxidative addition of Ni(0)L$_2$ catalyst into the *gem*-difluoride via nickel coordination to the carbonyl center. The lowest-lying TS is $\Delta G^{\ddagger}$ = 39.6 kcal/mol relative to **I**. The lowest lying transition structure for this pathway **TS**$_{\mathbf{OA\text{-}F\text{-}CO}}$ has a syn-arrangement of fluorine and carbonyl whereas an alternative structure with the anti-arrangement is higher in energy (**TS**$_{\mathbf{OA\text{-}F2\text{-}CO}}$ $\Delta G^{\ddagger}$ = 42.6 kcal/mol).

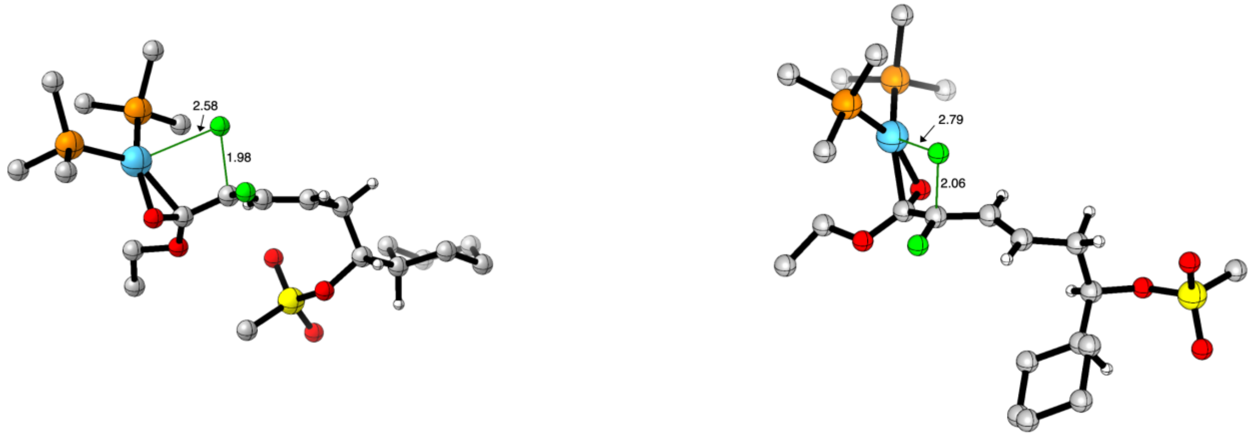


**$TS_{OA\text{-}F\text{-}CO}$**
$\Delta G^{\ddagger}$ = *+39.6*

**$TS_{OA\text{-}F2\text{-}CO}$**
$\Delta G^{\ddagger}$ = *+42.6*

**Fig SI-4: Computed lowest energy TSs for the oxidative addition of Ni(0)$L_2$ into the *gem*-difluoride via coordination of the catalyst to the carbonyl**

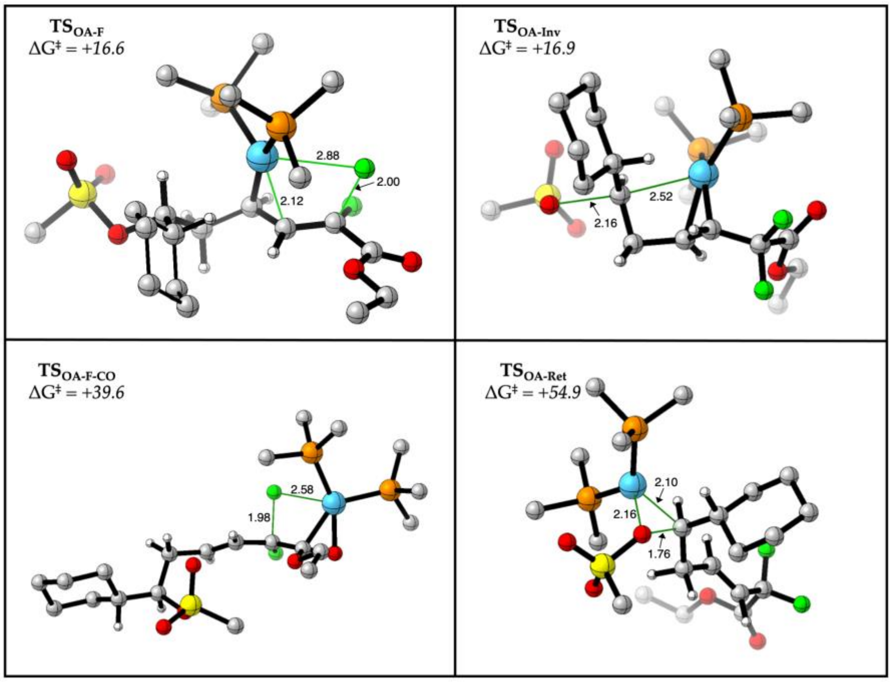


**Fig SI-5: Computed lowest energy TSs for the oxidative addition into allylic *gem*-difluoride ($TS_{OA\text{-}F}$), stereoinvertive OA ($TS_{OA\text{-}Inv}$), oxidative addition via coordination to the carbonyl ($TS_{OA\text{-}F\text{-}CO}$) and stereoretentive OA ($TS_{OA\text{-}Ret}$) at the secondary alkyl mesylate**

3. Pathway #1B halogen atom abstraction vs. oxidative addition of Ni(0)$L_2$ into the secondary alkyl bromide center

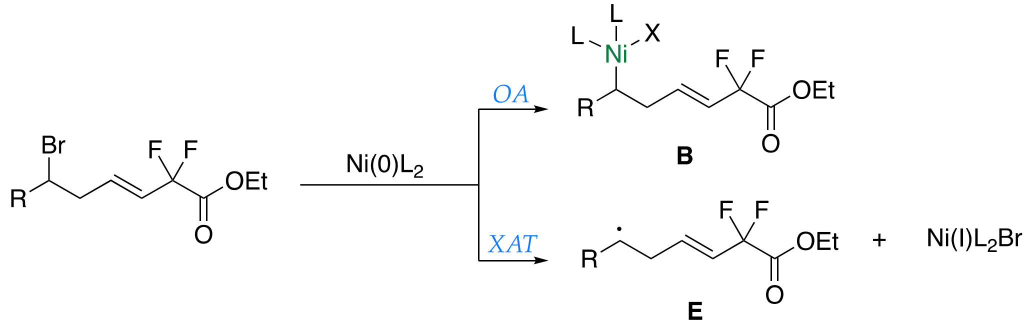


We also envisioned the halogen atom abstraction and oxidative addition of Ni(0)$L_2$ catalyst into the secondary alkyl bromide center. The lowest energy transition structure for halogen atom abstraction **$TS_{XAT}$** and stereoretentive oxidative addition **$TS_{OA\text{-}Ret\text{-}Br}$** was found to be 26.2 kcal/mol and 39.8 kcal/mol higher in energy from the secondary alkyl bromide pre-OA complex (from the reverse IRC).

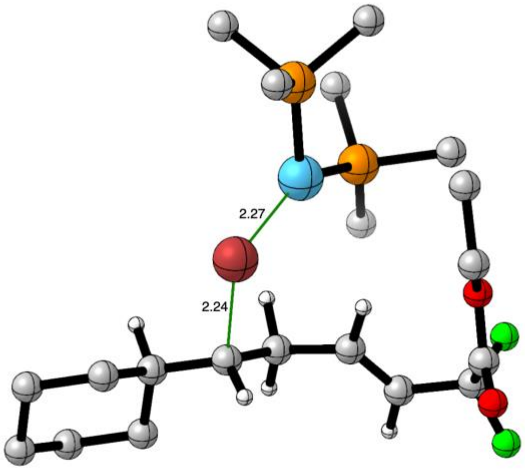


**$TS_{XAT}$**
$\Delta G^{\ddagger} = +26.2$

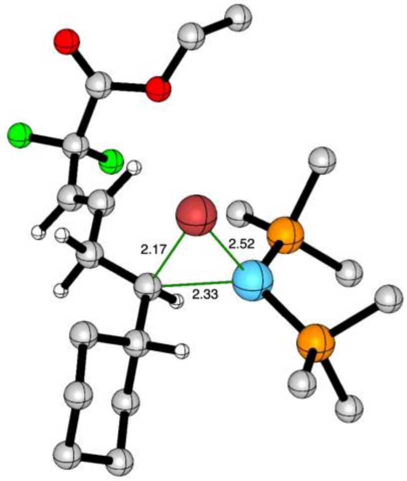


**$TS_{OA\text{-}Ret\text{-}Br}$**
$\Delta G^{\ddagger} = +39.8$

**Fig SI-6: Computed lowest energy TSs for halogen atom abstraction and oxidative addition of Ni(0)$L_2$ into the secondary alkyl bromide center for Pathway 1**

The lowest energy transition structure ($TS_{OA\text{-}Inv\text{-}Br}$) for stereoinvertive oxidative addition of Ni(0)$L_2$ into the secondary alkyl bromide center was located and found to be 19.6 kcal/mol from the secondary alkyl bromide pre-OA complex. Although the above-mentioned pathway is accessible at room temperature (however, the barrier is higher than the alternative OA TSs), stereoinvertive oxidative addition delivers product **D** with complete retention at the mesylate center which is inconsistent with the experimentally observed stereochemical outcome.

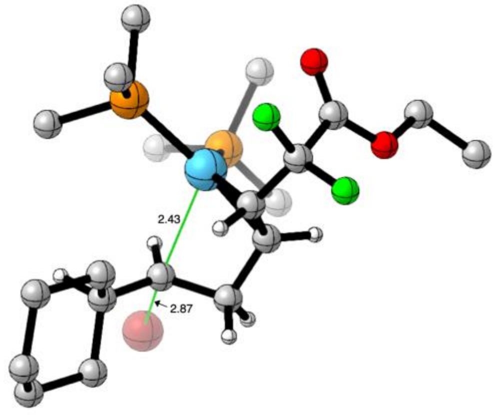


$TS_{OA\text{-}Inv\text{-}Br}$
$\Delta G^{\ddagger} = +19.6$

**Fig SI-7: Computed lowest energy TS for oxidative addition of Ni(0)$L_2$ into the secondary alkyl bromide center for Pathway 1**

4. S<u>N2 Displacement of Mesylate by Bromide</u>

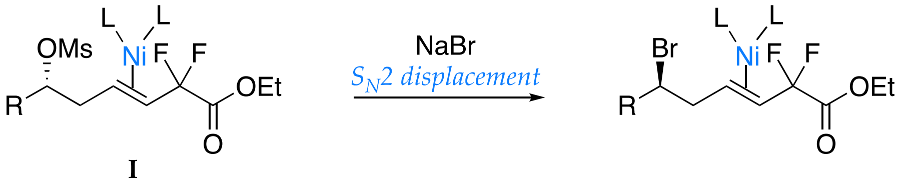


**I**

An alternate mechanism for the initiation of Pathway #1 is direct $S_N2$ displacement of mesylate by bromide at the carbon center to generate intermediate **E**. We explored the direct displacement of mesylate by bromide in the reaction. Comparing the relative free energies show **TS$_{SN2\text{-}Br}$** to **TS$_{OA\text{-}F}$** and **TS$_{OA\text{-}Inv}$** the free energy is calculated to be 8.8 kcal/mol and 7.7 kcal/mol higher in energy, respectively.

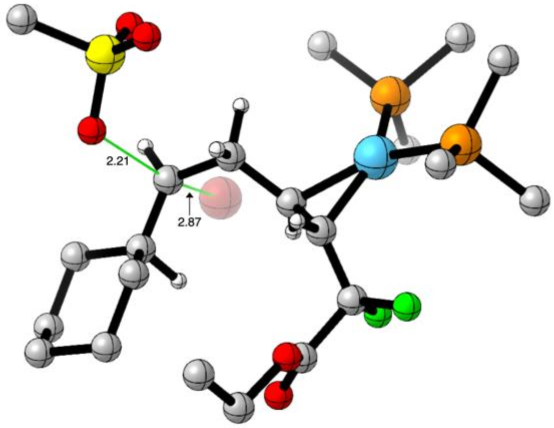


$TS_{SN2\text{-}Br}$

**Fig SI-8: Computed lowest energy TS for $S_N2$ displacement of mesylate by bromide for Pathway 1**

C. Origin of selectivity

1) Pathway #2

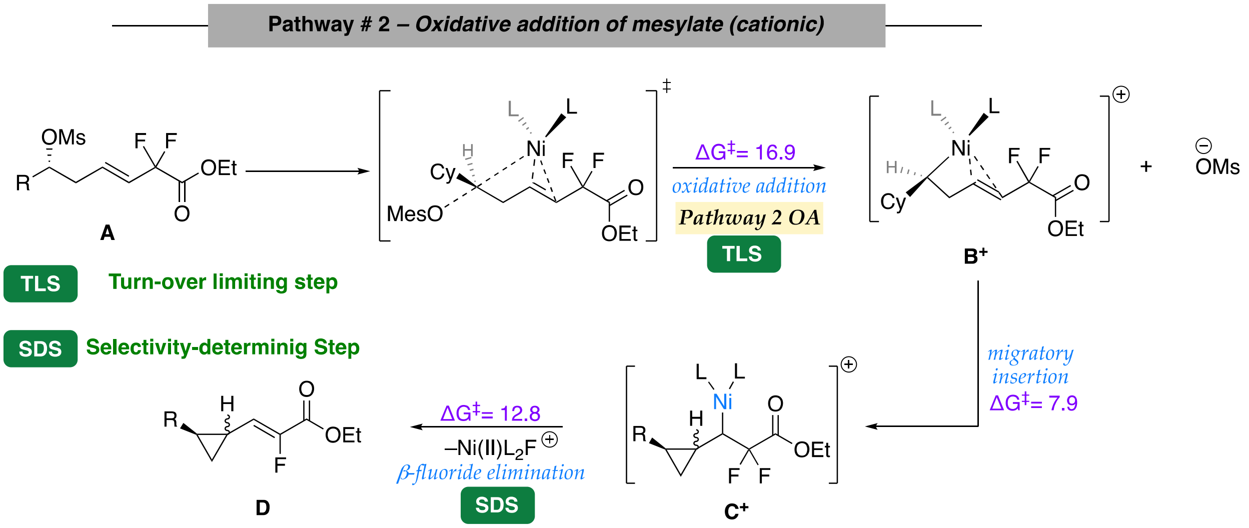


Pathway # 2 – The lowest energy transition structure for stereoinvertive OA is 16.9 kcal/mol from pre-OA complex **I**. Following the oxidative addition, complex $\mathbf{B}^+$ undergoes migratory insertion to deliver intermediate $\mathbf{C}^+$. The lowest energy transition structure for migratory insertion (**TS$_{\mathbf{MI\text{-}cis}}$**) has a barrier of 7.9 kcal/mol from the pre-OA complex **I**. A thorough conformational search was employed to locate the lowest energy stereoinvertive *cis*- and *trans*-migration TSs. The computed free energy difference for TS$_{\text{MI-cis}}$ and TS$_{\text{MI-trans}}$ at 298 K is 3.0 kcal/mol, favoring the *cis* TS. Finally, subsequent β-fluoride elimination (BFE) of $\mathbf{C}^+$ faces a barrier of 12.8 kcal/mol to deliver the final vinyl cyclopropane product and is the selectivity-determining step. The $\Delta\Delta G^{‡}$ between the lowest energy transition structures leading to the generation of vinyl cyclopropane product is 0.4 kcal/mol which is in good agreement with the experimental value (0.7 kcal/mol).

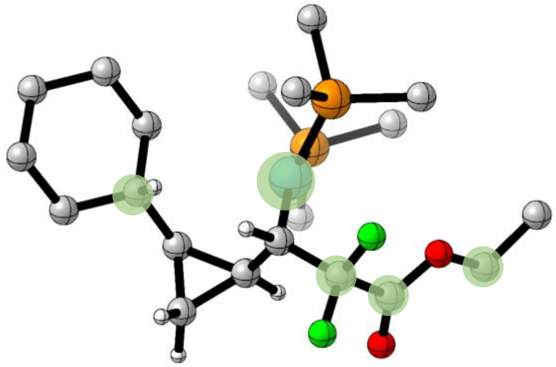

**Fig SI-9: Showing the rotatable bonds about which the conformational search was performed for migratory insertion.**

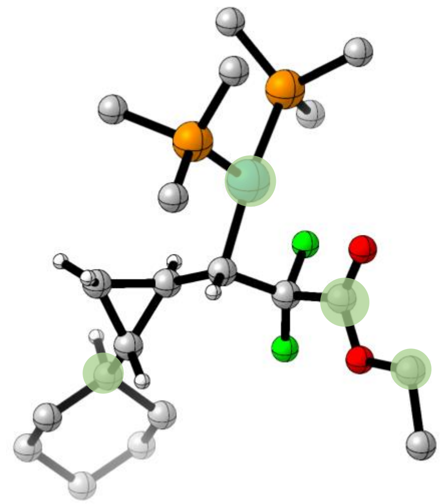

**Fig SI-10: Showing the rotatable bonds about which the conformational search was performed for β-fluoride elimination.**

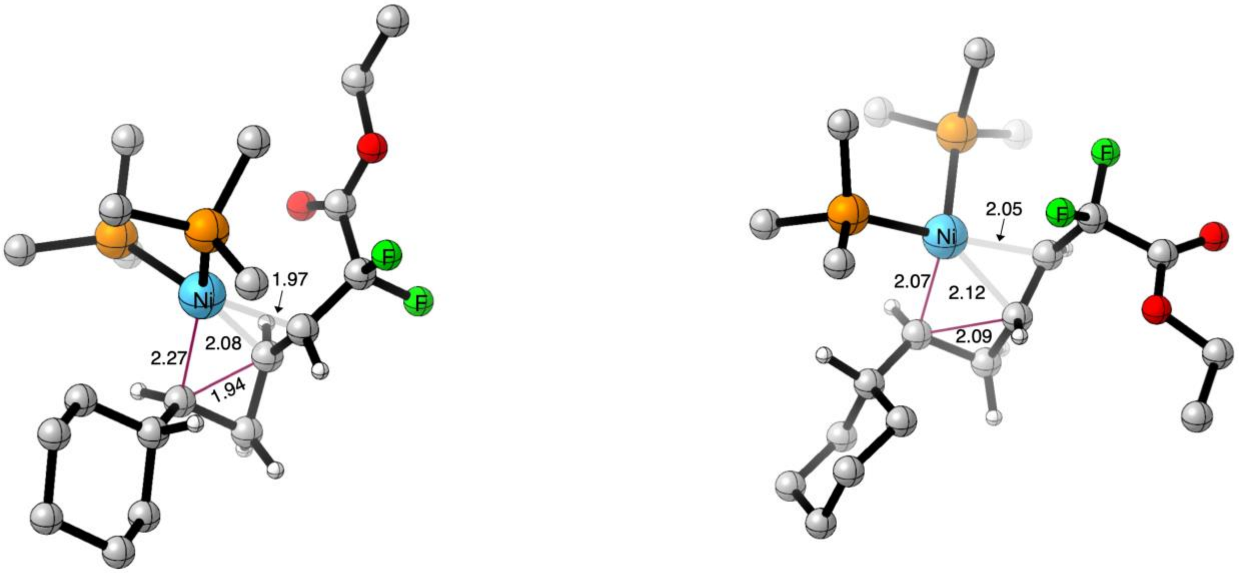


$\mathbf{TS_{MI\text{-}cis}}$
$\Delta G^{\ddagger} = +7.9$

$\mathbf{TS_{MI\text{-}trans}}$
$\Delta G^{\ddagger} = +10.9$

**Fig SI-11: Computed lowest energy TSs for migratory insertion for Pathway 2**

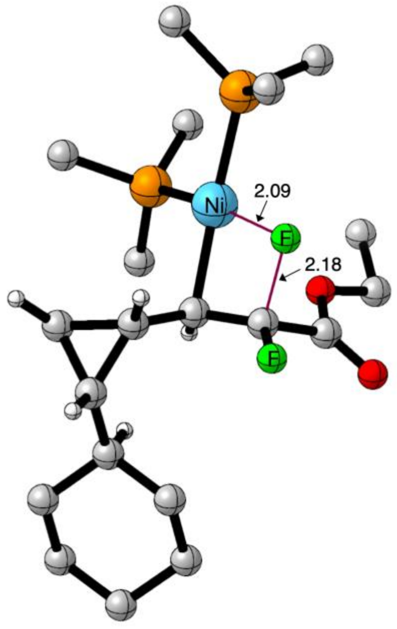


**$TS_{BFE\text{-}cis}$**
$\Delta G^{\ddagger} = +13.2$

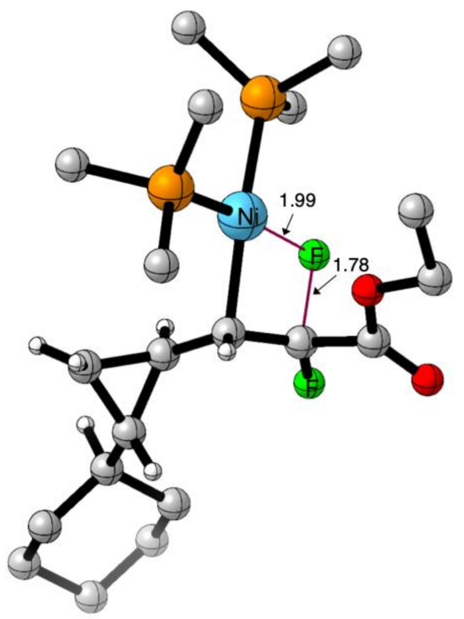


**$TS_{BFE\text{-}trans}$**
$\Delta G^{\ddagger} = +12.8$

Experimental $\Delta\Delta G^{\ddagger} = 0.7$ kcal/mol
Predicted $\Delta\Delta G^{\ddagger} = 0.4$ kcal/mol

**Fig SI-12: Computed lowest energy TSs for the β-fluoride elimination selectivity determining step for Pathway 2**

2) Pathway #3

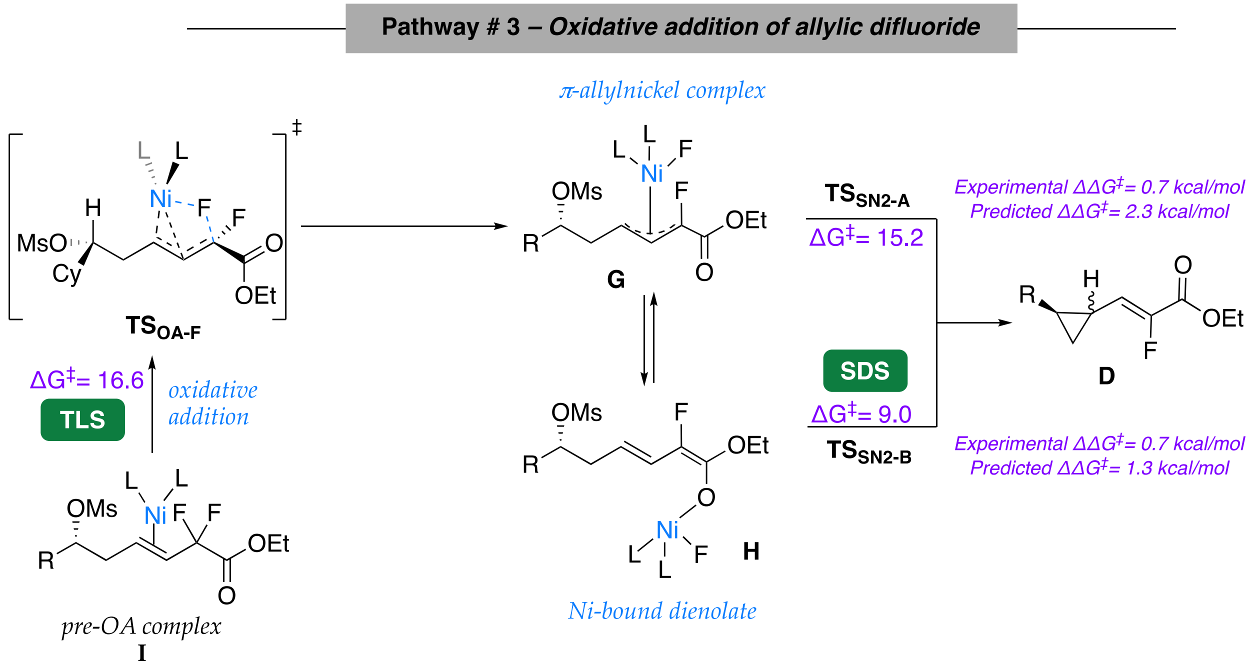

Pathway #3 – From the computed reaction coordinate diagram shown in Figure 3 of the manuscript, the ring-closure via π-allylnickel complex (**G**) or Ni-bound dienolate (**H**) intermediate is the selectivity-determining step in the reaction. A conformational search was employed for the formation of two different TSs that lead to both diastereomers of product from the π-allylnickel complex as well as Ni-bound dienolate complex. The $\Delta\Delta G^{\ddagger}$ between the lowest energy transition structures leading to the generation of vinyl cyclopropane product (**D**) from π-allylnickel complex (Pathway 3A) is 2.4 kcal/mol, whereas the $\Delta\Delta G^{\ddagger}$ between the lowest energy transition structures from Ni-dienolate complex (Pathway 3B) is 1.3 kcal/mol, which is in good agreement with the experimental value (0.7 kcal/mol). Further, a similar approach was extended to another model substrate (R= ethyl benzene, **3,** Scheme 2 in manuscript) to predict the 1.2:1 diastereoselectivity. The computed free energy difference between the two competing cis and trans transition structures was found to be 0.2 kcal/mol which was quantitatively consistent with the experimental value of ~ 0.1 kcal/mol.

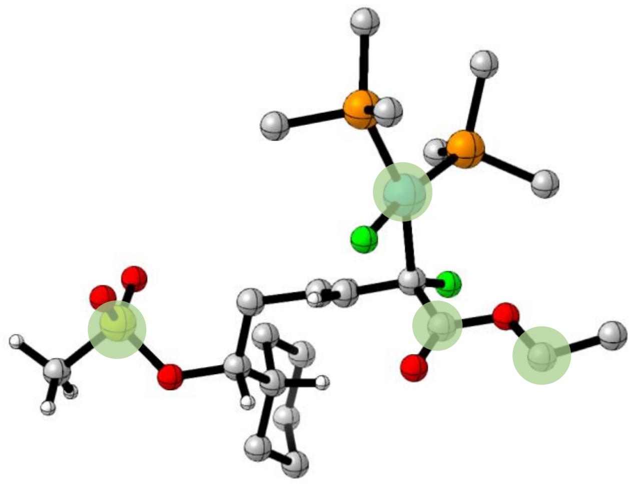

**Fig SI-13: Showing the rotatable bonds about which the conformational search was performed for π-allylnickel complex Pathway 3A.**

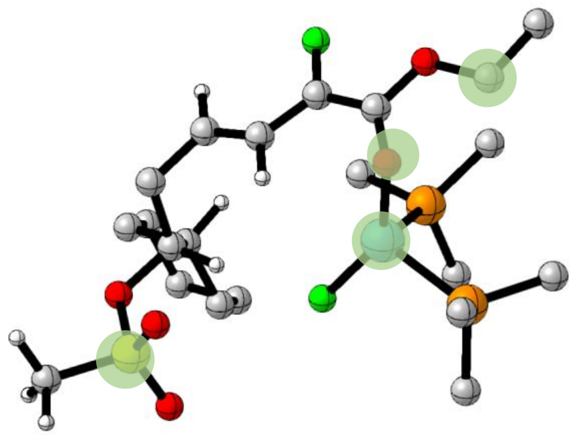

**Fig SI-14: Showing the rotatable bonds about which the conformational search was performed for Ni-bound dienolate complex Pathway 3B.**

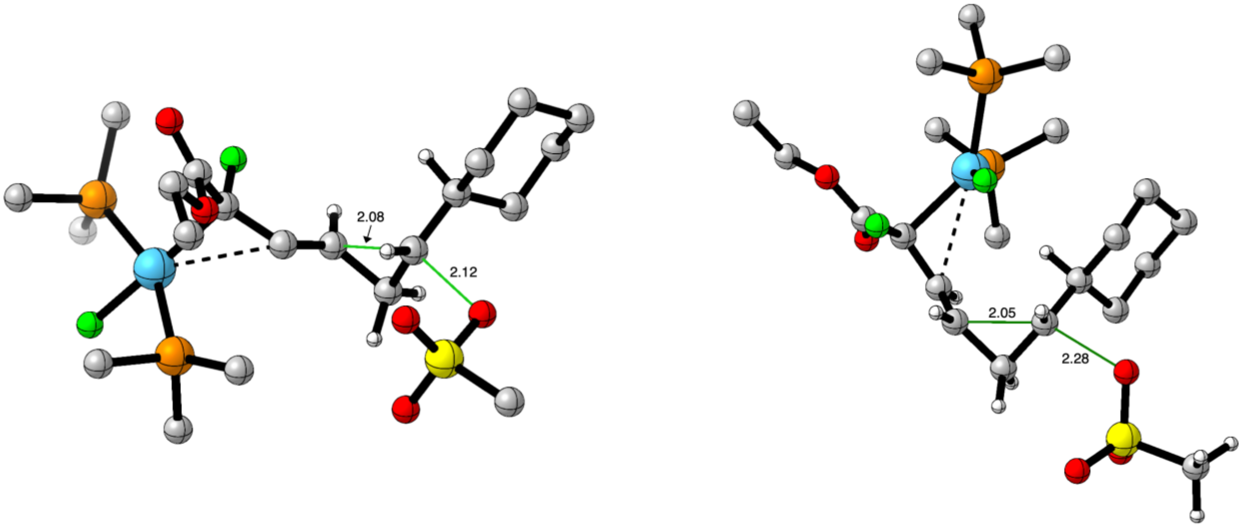


$TS_{SN2\text{-}A\text{-}cis}$
$\Delta G^{\ddagger} = +15.2$

$TS_{SN2\text{-}A\text{-}trans}$
$\Delta G^{\ddagger} = +17.6$

Experimental $\Delta\Delta G^{\ddagger}$ = 0.7 kcal/mol
Predicted $\Delta\Delta G^{\ddagger}$ = 2.4 kcal/mol

**Fig SI-15: Computed lowest energy TSs for *cis*- and *trans*-conformer from π-allyl nickel complex Pathway 3A.**

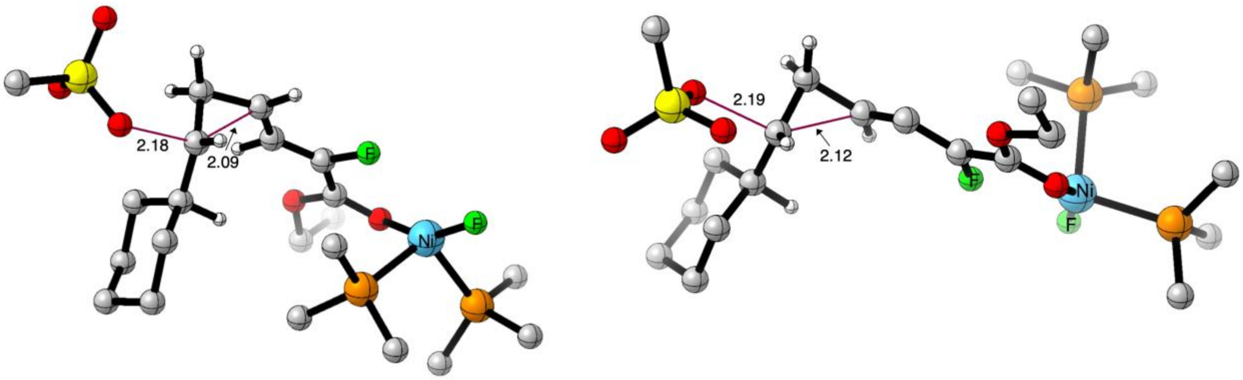


$TS_{SN2\text{-}B\text{-}trans}$
$\Delta G^{\ddagger} = +9.0$

$TS_{SN2\text{-}B\text{-}cis}$
$\Delta G^{\ddagger} = +10.3$

Experimental $\Delta\Delta G^{\ddagger}$ = 0.7 kcal/mol
Predicted $\Delta\Delta G^{\ddagger}$ = 1.3 kcal/mol

**Fig SI-16: Computed lowest energy TSs for *cis*- and *trans*-conformer from Ni-bound dienolate complex Pathway 3B.**

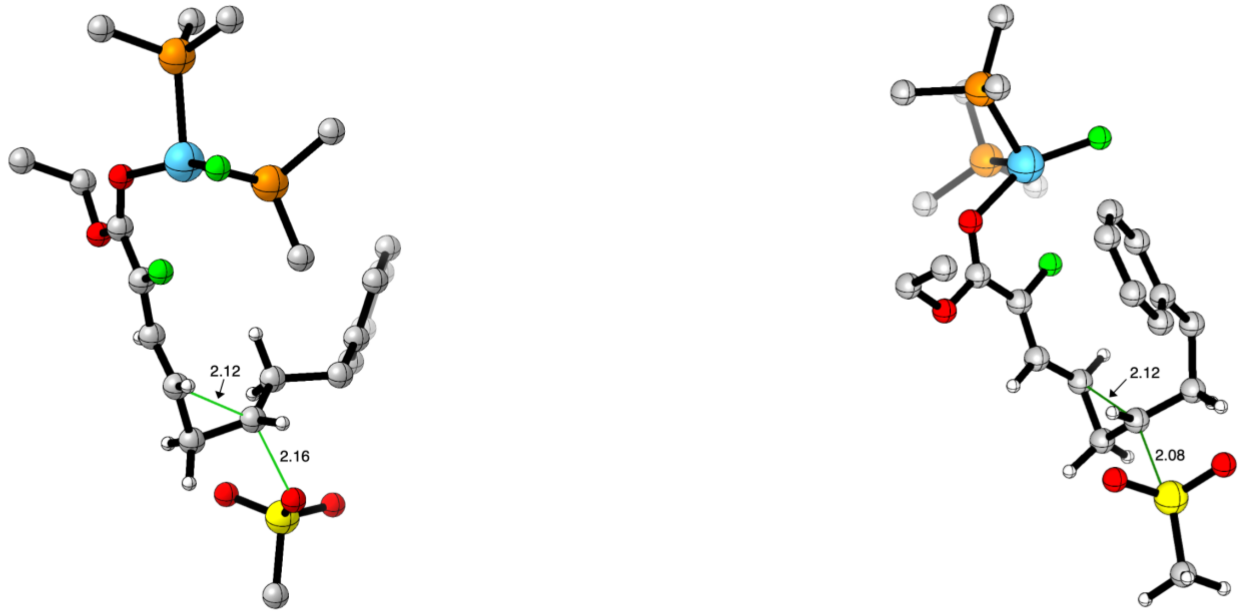


$\mathbf{TS}_{\mathbf{SN2\text{-}B\text{-}cis\text{-}phenethyl}}$
$\Delta\Delta G^{\ddagger} = 0.0$

$\mathbf{TS}_{\mathbf{SN2\text{-}trans\text{-}phenethyl}}$
$\Delta\Delta G^{\ddagger} = +0.2$

Experimental $\Delta\Delta G^{\ddagger}$ = 0.1 kcal/mol
Predicted $\Delta\Delta G^{\ddagger}$ = 0.2 kcal/mol

**Fig SI-17: Computed lowest energy TSs for *cis*- and *trans*-conformer for benzyl analog (3 in manuscript) from Ni-bound dienolate complex.**

D. Pathway 2 cationic vs. neutral pathway

We also modeled pathway 2 as a neutral species based on the forward IRC of $\mathbf{TS}_{\mathbf{OA\text{-}Inv}}$ (the forward IRC resulted in a C–O distance of 3.16 Å for the mesylate to the carbon center of the substrate indicating very little interaction between the leaving anion and the substrate). The free energy surface following intermediate B was modeled with mesylate in the system as a free ion; however, the qualitative trend for the neutral and cationic pathways are similar (Figure 2 in the manuscript for the cationic pathway and Figure SI-18 below for the neutral pathway). The rate- and selectivity-determining step remains consistent for both pathways. Beta fluoride elimination (BFE) of **C** is the selectivity-determining step and faces a barrier of 13.2 kcal/mol (compared to 12.8 kcal/mol for the cationic pathway) to deliver the final vinyl cyclopropane product. The $\Delta\Delta G^{\ddagger}$ between the lowest energy transition structures leading to the generation of *trans* and *cis* vinyl cyclopropane products is 0.2 kcal/mol (compared to $\Delta\Delta G^{\ddagger}$ = 0.4 kcal/mol for the cationic pathway). The discrepancies in energies are probably due to the lack of coordination of the free mesylate (we didn't conformationally search the position of the anion which has little affinity for the substrate) to the system rather than differences in the neutral and cationic systems.[xxxi]

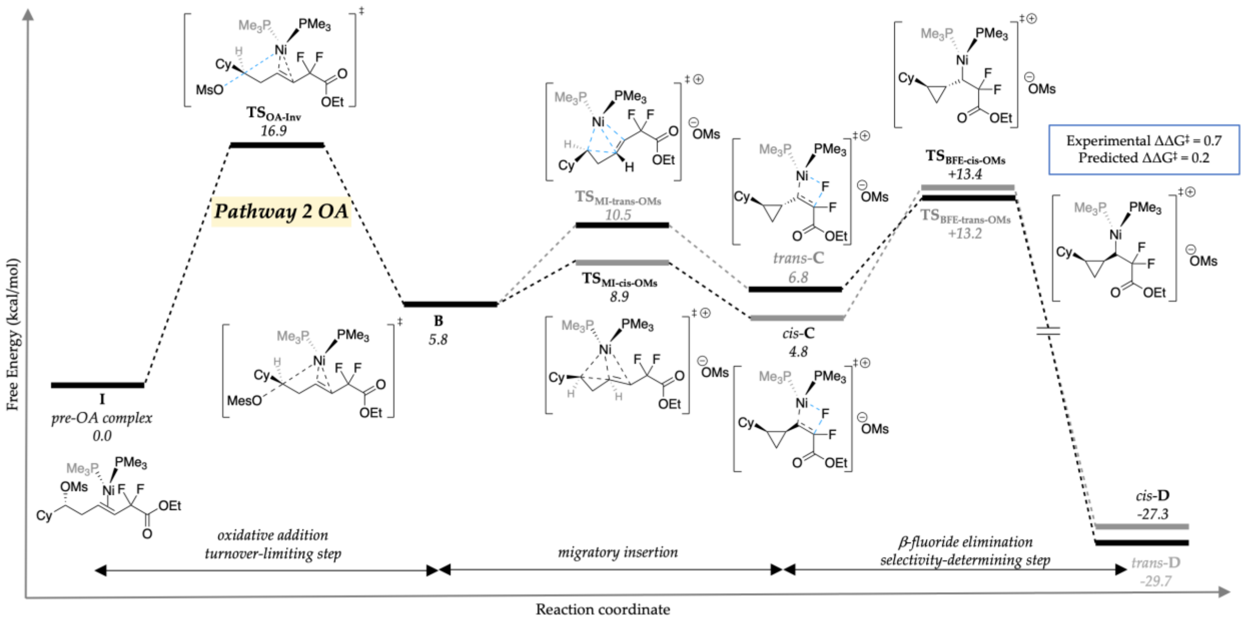


**Fig SI-18: Reaction coordinate diagram for Pathway # 2 (Neutral) calculated at the B3LYP-D3(BJ)/*def2*-TZVP PCM (acetonitrile)// B3LYP-D3/*def2*-SVP SMD (acetonitrile) level of theory.**

## E. Comparison of various computational methods

All the lowest energy transition structures and intermediate geometries were calculated using a variety of computational methods for the single point energies. The single point methods include the following: B3LYP-D3(BJ)/*def2*-TZVP/SMD (acetonitrile), M06-L/*def2*-TZVP/SMD (acetonitrile) and wB97-XD/*def2*-TZVP/SMD (acetonitrile). For all methods the relative energies of the surface for each pathway remain consistent (i.e., the turn-over limiting and stereoselective steps are similar for each method). The oxidative addition steps for both Pathway 2 and Pathway 3 are close in energy for all methods. The method published herein B3LYP-D3(BJ)/*def2*-TZVP/PCM (acetonitrile) lies within the average of the methods explored.

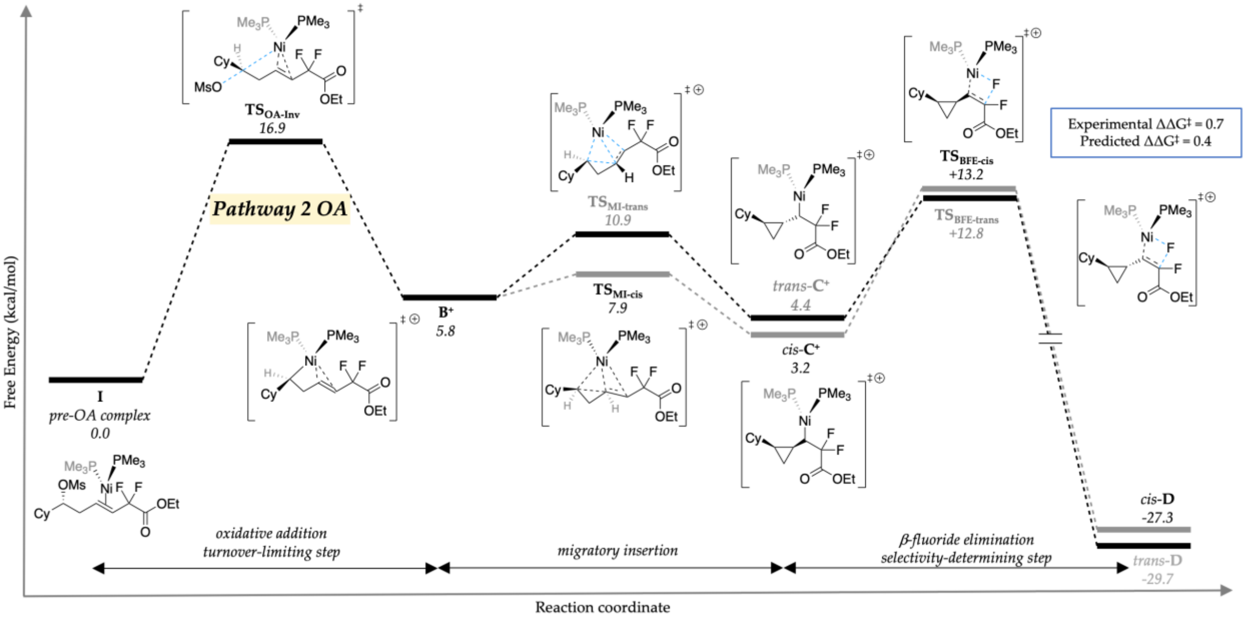


**Fig SI-19: Reaction coordinate diagram for Pathway # 2 calculated at the B3LYP-D3(BJ)/*def2*-TZVP PCM (acetonitrile)// B3LYP-D3/*def2*-SVP SMD (acetonitrile) level of theory (Figure 2 in manuscript).**

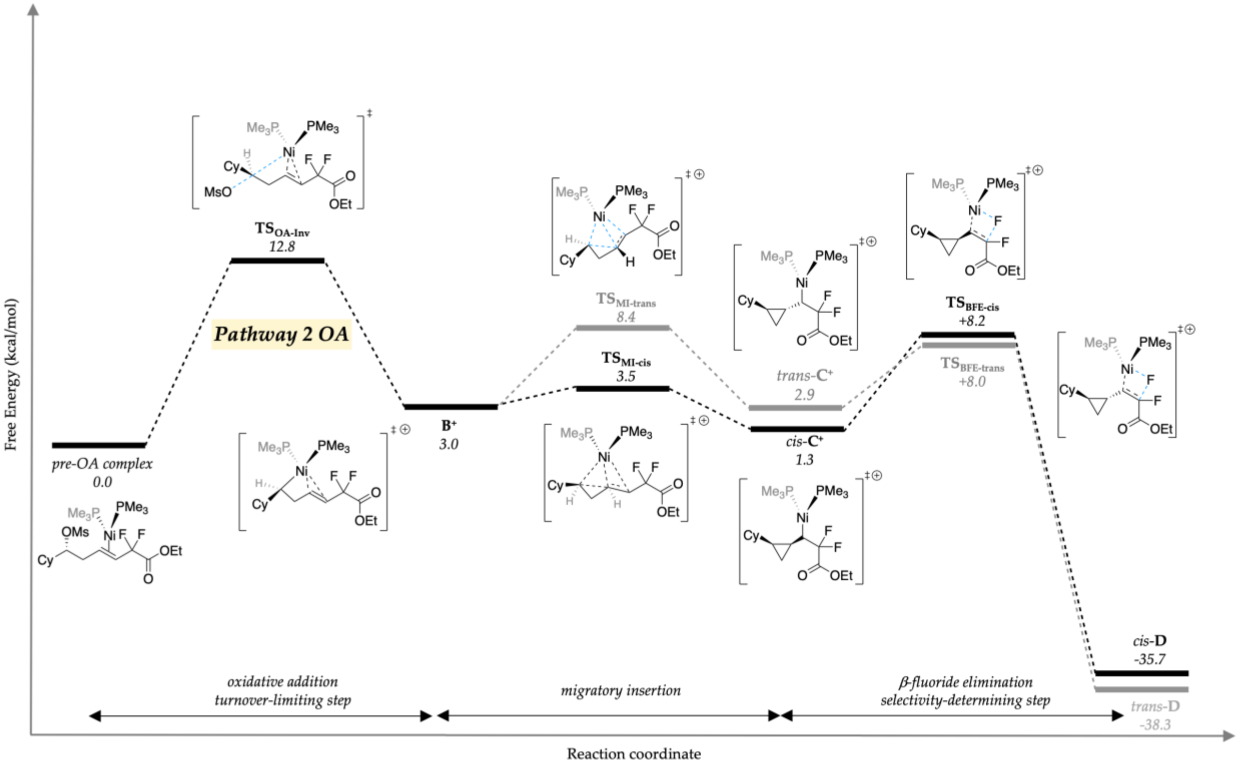


**Fig SI-20: Reaction coordinate diagram for Pathway # 2 calculated at the M06-L/*def2*-TZVP SMD (acetonitrile)// B3LYP-D3/*def2*-SVP SMD (acetonitrile) level of theory**

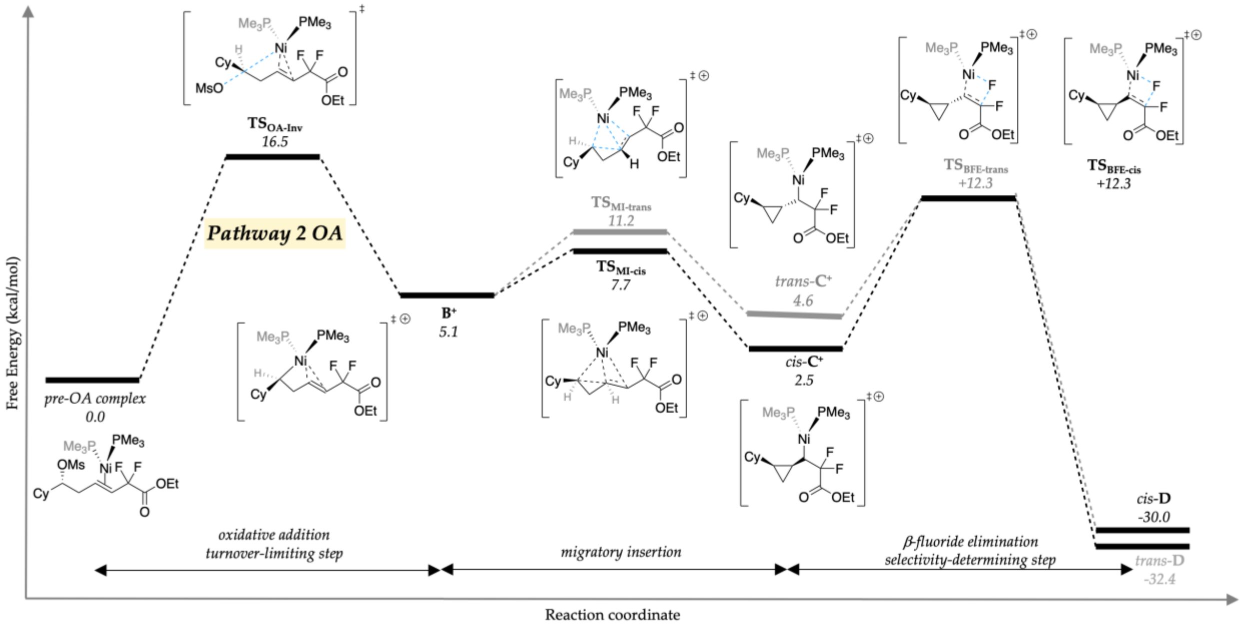


**Fig SI-21: Reaction coordinate diagram for Pathway # 2 calculated at the B3LYP-D3(BJ)/*def2*-TZVP SMD (acetonitrile)// B3LYP-D3/*def2*-SVP SM (acetonitrile) level of theory**

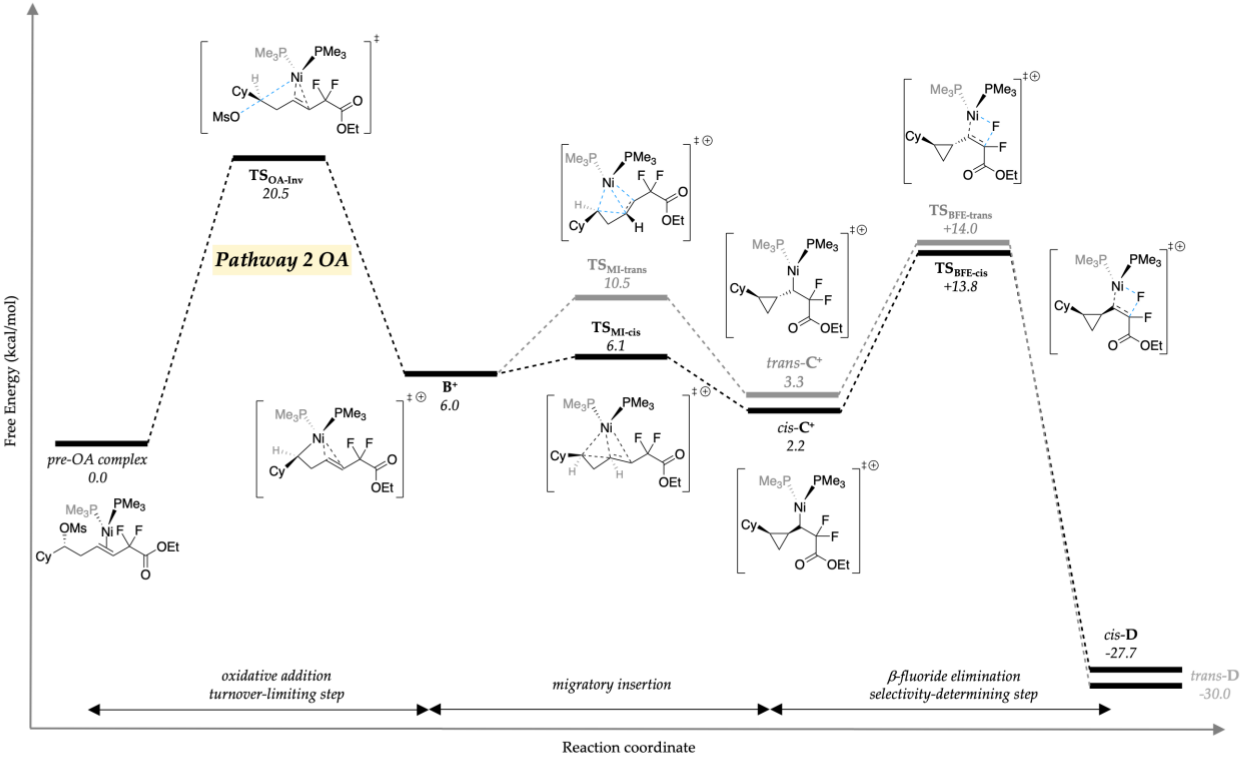


**Fig SI-22: Reaction coordinate diagram for Pathway # 2 calculated at the wB97-XD/*def2*-TZVP SMD (acetonitrile)// B3LYP-D3/*def2*-SVP SMD (acetonitrile) level of theory**

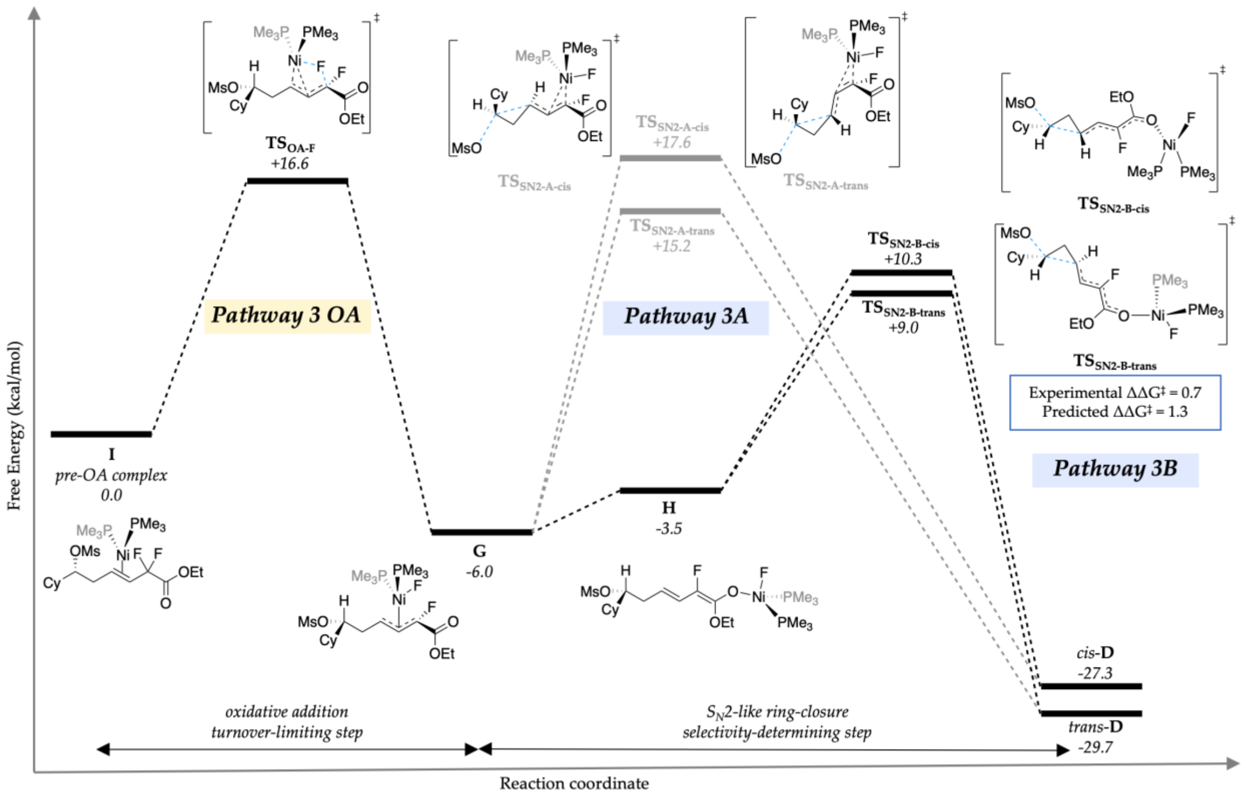


**Fig SI-23: Reaction coordinate diagram for Pathway # 3 calculated at the B3LYP-D3(BJ)/*def2*-TZVP PCM (acetonitrile)// B3LYP-D3/*def2*-SVP SMD (acetonitrile) level of theory (Figure 3 in manuscript)**

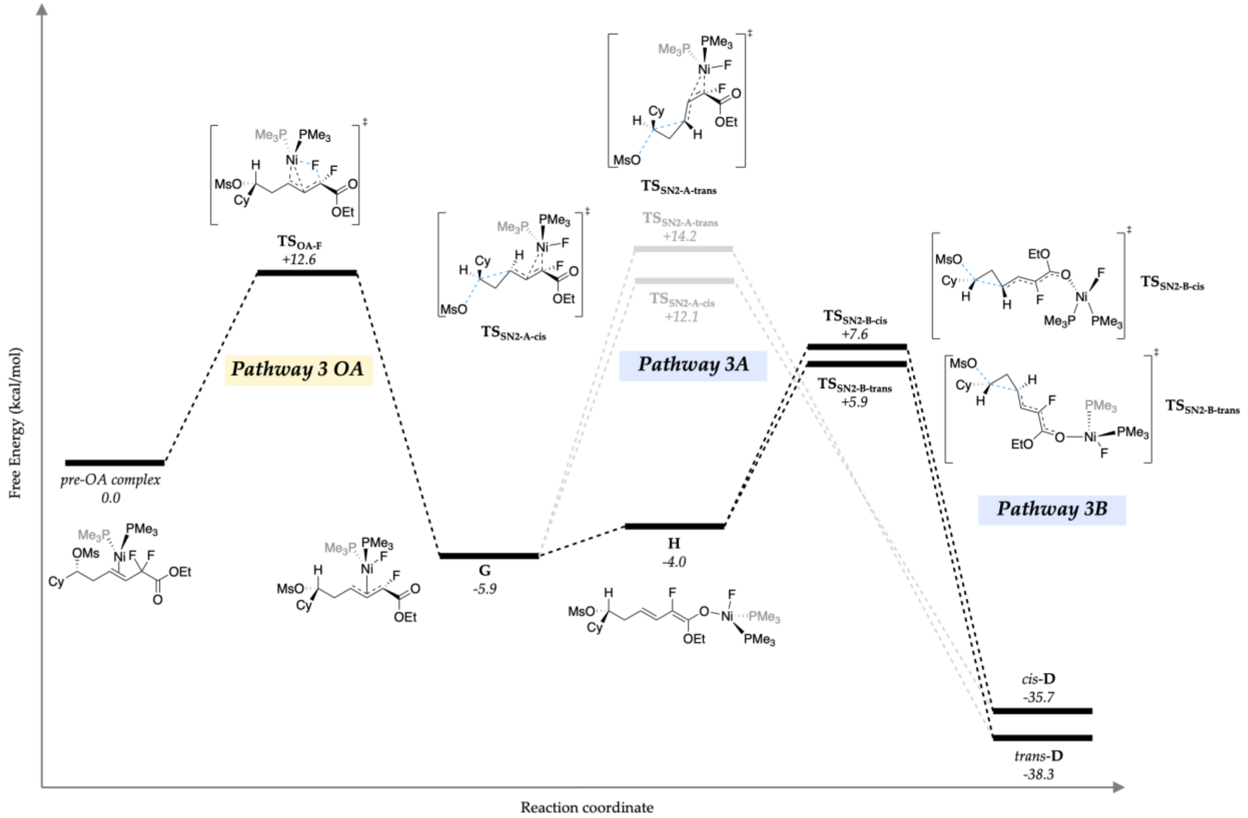


**Fig SI-24: Reaction coordinate diagram for Pathway # 3 calculated at the M06-L/*def2*-TZVP SMD (acetonitrile)// B3LYP-D3/*def2*-SVP SMD (acetonitrile) level of theory.**

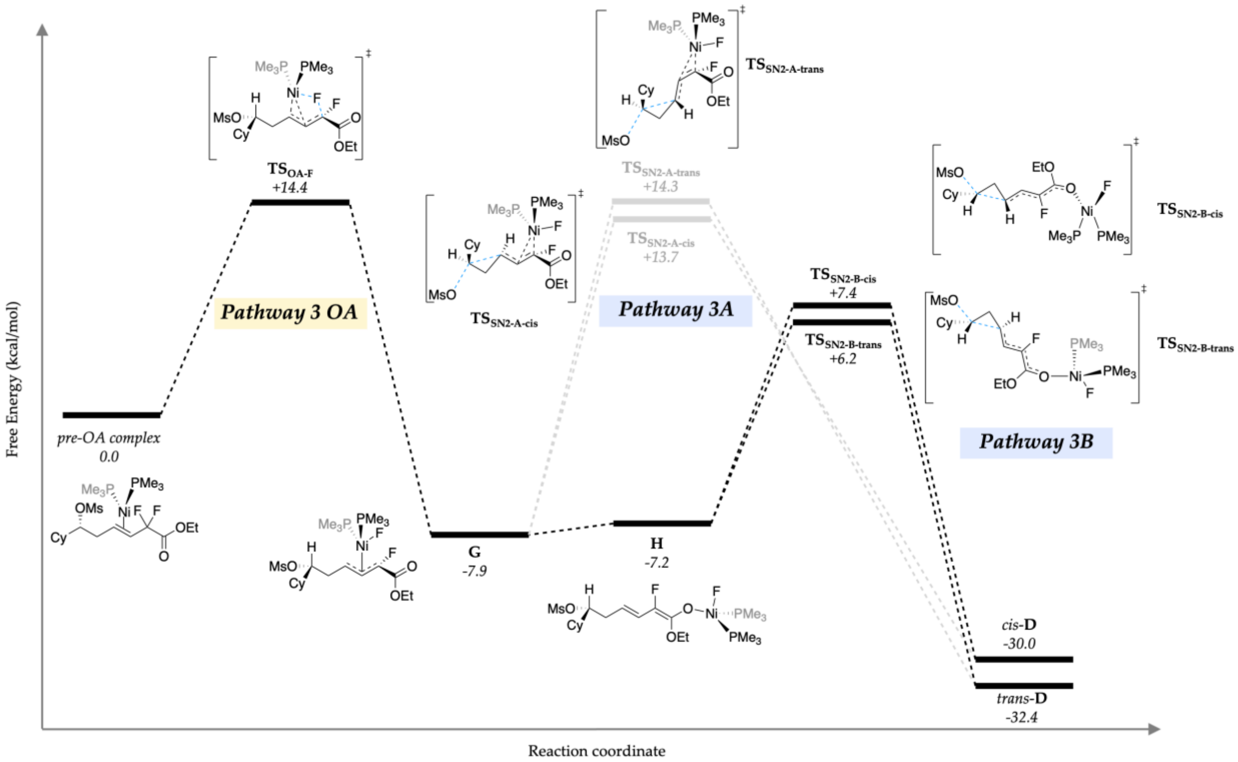


**Fig SI-25: Reaction coordinate diagram for Pathway # 3 calculated at the B3LYP-D3(BJ)/*def2*-TZVP SMD (acetonitrile)// B3LYP-D3/*def2*-SVP SMD (acetonitrile) level of theory.**

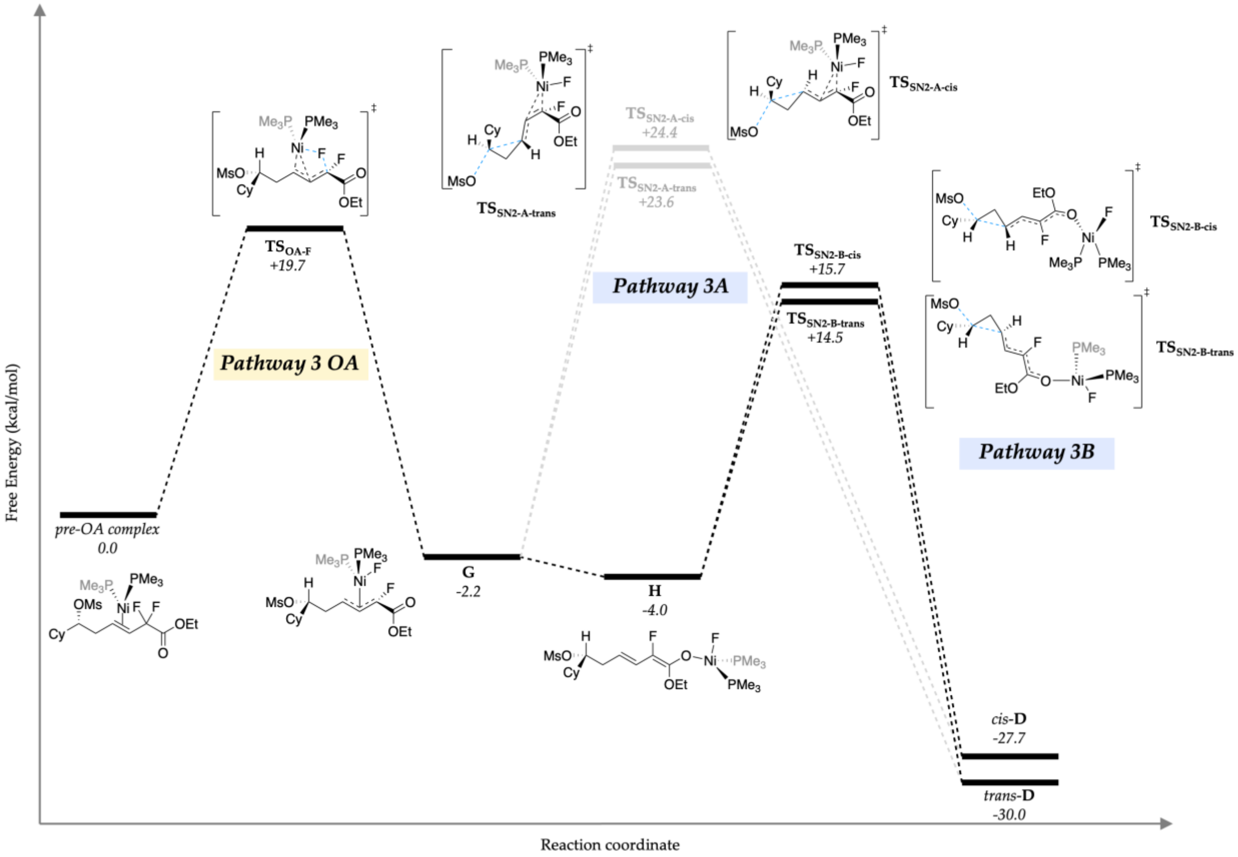


**Fig SI-26: Reaction coordinate diagram for Pathway # 3 calculated at the wB97-XD/*def2*-TZVP SMD (acetonitrile)// B3LYP-D3/*def2*-SVP SMD (acetonitrile) level of theory.**

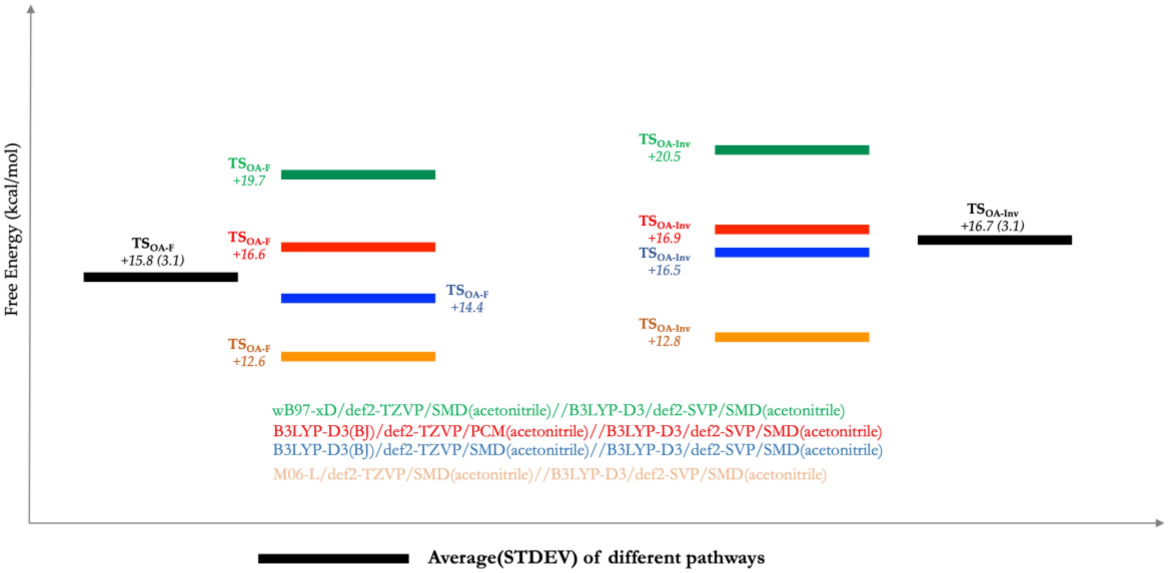


**Fig SI-27: Average (in black, the standard deviation is in parentheses) of the various methods for both oxidative addition pathways (manuscript Figures 2 and 3)**

## F. Analysis of key transition structures for oxidative addition

To better understand and the possible reaction pathways, we employed distortion/interaction and energy decomposition analysis on the key oxidative addition transition structures. Distortion energy is the energy required to deform the reactants and catalyst from their ground state minimum energy geometries. This is evident from the Figures SI-27 which show the relative distortion of the reactant and catalyst fragments of the lowest transition structures for oxidative addition for Pathway 2 (**TS$_{\text{OA-Inv}}$**) with respect to Pathway 3 (**TS$_{\text{OA-F}}$**).

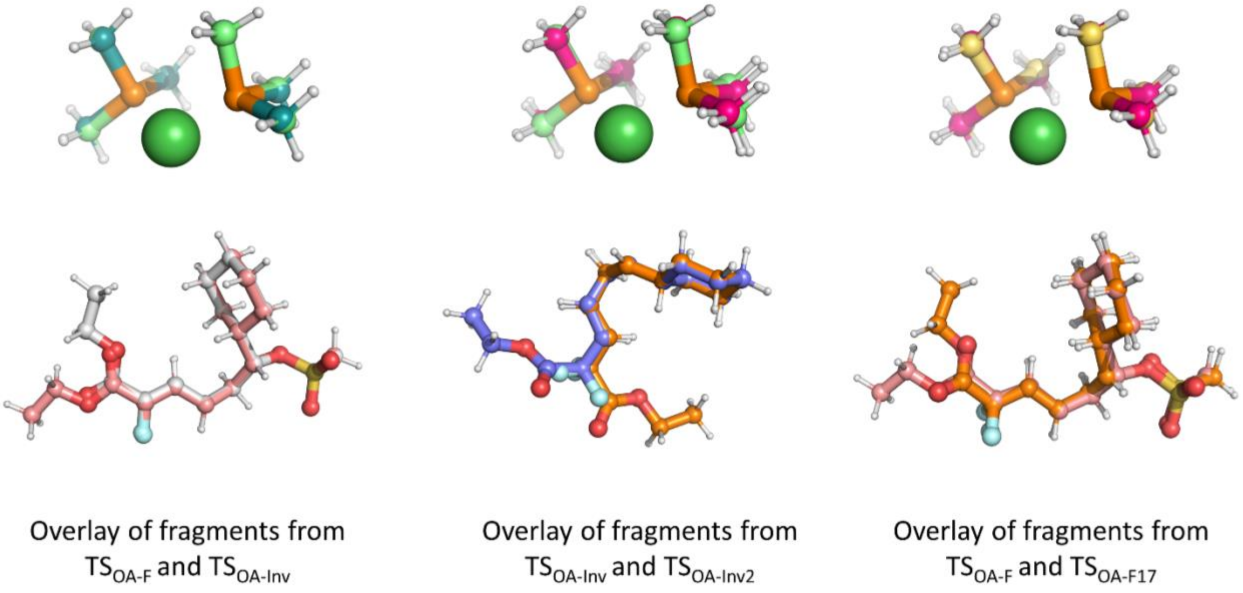


**Fig SI-28: Overlay of relative distortion of the reactant and catalyst fragments for TS$_{\text{OA-F}}$ and TS$_{\text{OA-Inv}}$**

Distortion energy for these systems is tabulated in Table SI-1 and demonstrates that both the catalyst and the reactant fragments suffer from more destabilizing distortion interactions in **TS$_{\text{OA-F}}$**. Furthermore, it is evident from the table that the two lowest-lying TSs for Pathway 3 (**TS$_{\text{O-AF}}$** and **TS$_{\text{OA-F17}}$**) have similar distortions while the oxidative addition TSs that go through the stereoinvertive pathway (**TS$_{\text{OA-F}}$** and **TS$_{\text{OA-Inv2}}$**) suffer from different amounts of distortions.

**Table SI-1:** Distortion energies of the catalyst and the reactant fragments for various oxidative addition TSs with respect to **TS$_{\text{OA-F}}$**. Energies in kcal/mol computed at B3LYP-D3BJ PCM (acetonitrile)/def2-TZVP.

| **Fragment** | **TS$_{\text{OA-F}}$** | **TS$_{\text{OA-F17}}$** | **TS$_{\text{OA-Inv}}$** | **TS$_{\text{OA-Inv2}}$** |
|---|---|---|---|---|
| **Catalyst** | 0.0 | 0.0 | −1.2 | −1.3 |
| **Reactant** | 0.0 | +0.4 | −8.3 | −9.9 |

Furthermore, interaction energies computed for these four TSs revealed a strong preference for **TS$_{\text{OA-F}}$**. The absolute interaction energy between the catalyst fragment and the reactant fragments for each of these TSs are tabulated in Table SI-2 along with the key components contributing to the interaction energy. Decomposing this energy into dispersion component based off Grimme's DFT-D3 with Becke-Johnson corrections suggested that each of these TSs enjoy different

favorable dispersion interactions and oxidative addition stereoinvertive TSs enjoy substantially more dispersion interactions compared to oxidative addition into the allyl gem-difluoride. Finally, electrostatic interaction energies computed between the catalyst and the reactant fragment reveal a strong electrostatic stabilization energy for the TSs following the OA-F pathways as shown in Table SI-2.

**Table SI-2:** Interaction energies between the catalyst and the reactant fragments for various oxidative addition TSs. Energies in kcal/mol computed at PCM-B3LYP-D3BJ/def2-TZVP.

| Component | $TS_{OA-F}$ | $TS_{OA-F17}$ | $TS_{OA-Inv}$ | $TS_{OA-Inv2}$ |
|---|---|---|---|---|
| **Absolute interactions** | −104.5 | −93.4 | −92.0 | −104.0 |
| **Dispersion interactions** | −25.9 | −26.8 | −29.1 | −25.1 |
| **Electrostatic interactions** | −4.6 | −1.4 | −0.6 | −4.3 |

G. <u>Analysis of key transition structures for selectivity determining step for pathway 2 and 3</u>

To understand the interactions that contribute to the selectivity in this reaction, we performed energy decomposition analysis as described earlier on $\mathbf{TS_{SN2\text{-}B\text{-}trans}}$ and $\mathbf{TS_{SN2\text{-}B\text{-}cis}}$. Based on this analysis, the catalyst fragments for the competing TSs show much smaller distortions for the minor product while simultaneously undergoing significantly large unfavorable distortions (~3.0 kcal/mol) for the reactant fragment. However, the TS leading to the minor product was found to enjoy significantly more favorable interactions (3.1 kcal/mol) compared to the TS leading to the major product. Furthermore, this interaction energy when decomposed into electrostatic component and dispersion components showed a *similarly* strong preference for the TS leading to the minor product. Similar analysis was carried out for $\mathbf{TS_{BFE\text{-}cis}}$ and $\mathbf{TS_{BFE\text{-}trans}}$. For this pathway, the TS leading to the major product enjoyed fewer destabilizing distortions (1.5 kcal/mol) compared to the TS leading to the minor product. Similar to the $S_N2$ pathway, the interactions between the reactant and catalyst fragments favored the TS leading to the minor product with only a fraction of stabilization coming from both dispersion and electrostatic components individually.

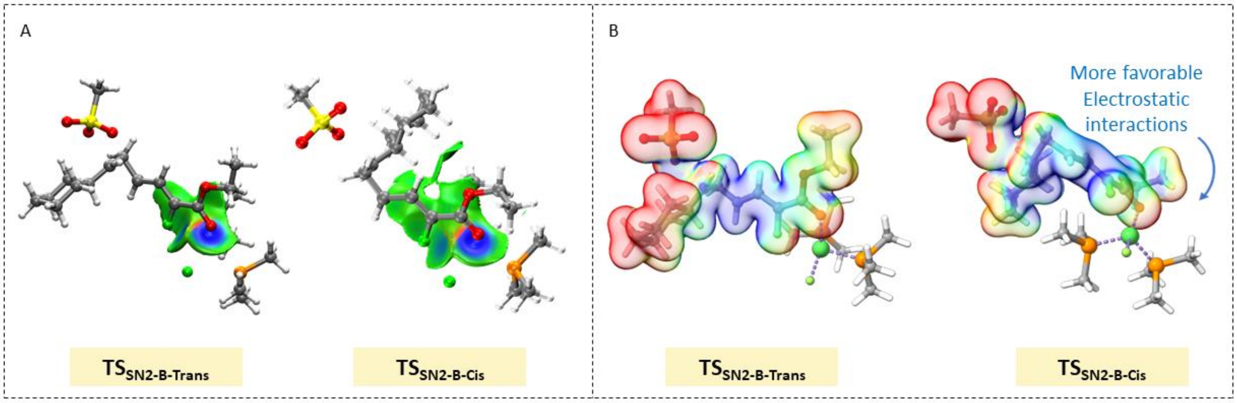


| | Distortion | $Distortion_{Cat}$ | $Distortion_{Rct}$ | Interaction | $Int_{Dispersion}$ | $Int_{Electrostatic}$ |
|---|---|---|---|---|---|---|
| $\Delta\Delta E^{\ddagger}$ ($TS_{SN2\text{-}B\text{-}Cis}$ rel. to $TS_{SN2\text{-}B\text{-}Trans}$) | +2.5 | -0.5 | +3.0 | -3.1 | -5.4 | −2.4 |

**Fig SI-29:** (A) Non-covalent interaction plots for the lowest energy OA TSs. The green surfaces indicate weak Van der Waal's interactions (isovalue 0.015). (B) Qualitative depiction of the stabilizing electrostatic interactions operative in the key selectivity determining step TSs for pathway 3. (Electrostatic potential (ESP) range of +20 kcal/mol (red) to +100 kcal/mol (blue).

Table: All energies are displayed as the relative energy of **TS$_{SN2\text{-}B\text{-}cis}$** to **TS$_{SN2\text{-}B\text{-}trans}$**. The energies in green shaded boxes indicate the components from energy decomposition analysis favoring **TS$_{SN2\text{-}B\text{-}trans}$** while red shaded boxes in the table indicate the components favoring **TS$_{SN2\text{-}B\text{-}cis}$** (Pathway 3).

**Table SI-3:** All energies are displayed as the relative energy of **TS$_{BFE\text{-}cis}$** to **TS$_{BFE\text{-}trans}$**. The energies in green shaded boxes indicate the components from energy decomposition analysis favoring **TS$_{BFE\text{-}trans}$** while red shaded boxes in the table indicate the components favoring **TS$_{BFE\text{-}cis}$** (Pathway 2).

| | Distortion | Distortion$_{Cat}$ | Distortion$_{Rct}$ | Interaction | Int$_{Dispersion}$ | Int$_{Electrostatic}$ |
|---|---|---|---|---|---|---|
| $\Delta\Delta E^{\ddagger}$ (TS$_{BFE\text{-}Cis}$ rel. to TS$_{BFE\text{-}Trans}$) | +1.5 | +0.1 | +1.4 | -1.2 | -0.3 | −0.2 |

## H. Energetics for different possible conformers

**Table SI-4:** Oxidative addition of Ni(0)L$_2$ into allyl gem-difluoride for Pathway 3. The lowest energy conformation for each TS that corresponds with the structure in the manuscript is highlighted in red.

| Transition Structures | $r_{(C\text{-}F)}$ (Å) | $r_{(Ni\text{-}F)}$ (Å) | Energy (kcal/mol) |
|---|---|---|---|
| TS-OA-F | 1.99 | 2.88 | 16.5 |
| TS-OA-F2 | 2.00 | 2.92 | 20.8 |
| TS-OA-F3 | 1.93 | 2.84 | 22.7 |
| TS-OA-F4 | 1.96 | 2.73 | 26.9 |
| TS-OA-F5 | 1.96 | 2.70 | 27.3 |
| TS-OA-F6 | 1.96 | 2.72 | 27.3 |
| TS-OA-F7 | 1.91 | 2.80 | 25.3 |
| TS-OA-F8 | 1.97 | 2.79 | 23.1 |
| TS-OA-F9 | 2.03 | 2.88 | 26.6 |
| TS-OA-F10 | 2.03 | 2.88 | 22.1 |
| TS-OA-F11 | 1.99 | 2.81 | 21.9 |
| TS-OA-F12 | 2.05 | 2.89 | 26.1 |
| TS-OA-F13 | 2.09 | 2.92 | 22.1 |
| TS-OA-F14 | 1.94 | 2.86 | 22.1 |
| TS-OA-F15 | 1.98 | 2.89 | 20.3 |
| TS-OA-F17 | 1.99 | 2.84 | 17.2 |
| TS-OA-F18 | 2.00 | 2.88 | 16.5 |
| TS-OA-F19 | 1.94 | 2.95 | 20.0 |
| TS-OA-F20 | 1.95 | 2.95 | 19.3 |

**Table SI-5:** Oxidative addition step of Ni(0)$L_2$ into the *gem*-difluoride via coordination of the catalyst to the carbonyl for Pathway #3. The lowest energy conformation for each TS that corresponds with the structure in the manuscript is highlighted in red.

| Transition Structures | $r_{(C\text{-}F)}$ (Å) | $r_{(Ni\text{-}F)}$ (Å) | Energy (kcal/mol) |
|---|---|---|---|
| TS-OA-F-CO | 2.00 | 2.63 | 39.6 |
| TS-OA-F-CO-2 | 1.98 | 2.58 | 42.6 |
| TS-OA-F-CO-3 | 2.08 | 2.83 | 44.8 |
| TS-OA-F-CO-4 | 2.02 | 2.60 | 43.8 |
| TS-OA-F-CO-5 | 1.97 | 2.60 | 42.8 |
| TS-OA-F-CO-6 | 2.00 | 3.06 | 41.1 |

**Table SI-6:** Intramolecular direct ring closure from π-allylnickel complex–for Pathway 3A- cis and trans conformations. The lowest energy conformation for each TS that corresponds with the structure in the manuscript is highlighted in red.

| Transition Structures | $r_{(C\text{-}C)}$ (Å) | $r_{(C\text{-}O)}$ (Å) | Energy (kcal/mol) |
|---|---|---|---|
| TS-SN2-A-cis | 2.05 | 2.27 | 17.6 |
| TS-SN2-A-cis-2 | 2.03 | 2.41 | 23.0 |
| TS-SN2-A-cis-3 | 2.02 | 2.41 | 22.2 |
| TS-SN2-A-cis-4 | 2.10 | 2.47 | 18.7 |
| TS-SN2-A-cis-5 | 2.05 | 2.27 | 17.6 |
| | | | |
| TS-SN2-A-trans | 2.03 | 2.36 | 15.2 |
| TS-SN2-A-trans-2 | 2.06 | 2.25 | 19.9 |
| TS-SN2-A-trans-3 | 2.10 | 2.29 | 19.7 |
| TS-SN2-A-trans-4 | 2.05 | 2.28 | 20.3 |
| TS-SN2-A-trans-5 | 2.01 | 2.34 | 16.6 |
| TS-SN2-A-trans-6 | 2.02 | 2.33 | 20.9 |
| TS-SN2-A-trans-7 | 2.04 | 2.31 | 17.0 |
| TS-SN2-A-trans-8 | 2.01 | 2.34 | 20.9 |
| TS-SN2-A-trans-9 | 2.04 | 2.31 | 16.6 |

**Table SI-7:** Intramolecular ring closure from nickel dienolate intermediate for Pathway 3B- cis and trans conformations. The lowest energy conformation for each TS that corresponds with the structure in the manuscript is highlighted in red.

| Transition Structures | $r_{(C\text{-}C)}$ (Å) | $r_{(C\text{-}O)}$ (Å) | Energy (kcal/mol) |
|---|---|---|---|
| TS-SN2-B-cis | 2.12 | 2.19 | 10.3 |
| TS-SN2-B-cis-2 | 2.12 | 2.20 | 12.5 |
| TS-SN2-B-cis-3 | 2.16 | 2.13 | 13.7 |
| TS-SN2-B-cis-4 | 2.10 | 2.19 | 15.9 |
| TS-SN2-B-cis-5 | 2.12 | 2.20 | 11.6 |
| TS-SN2-B-cis-6 | 2.11 | 2.18 | 14.1 |
| TS-SN2-B-cis-7 | 2.13 | 2.19 | 12.5 |

| | | | |
|---|---|---|---|
| TS-SN2-B-cis-8 | 2.09 | 2.20 | 11.5 |
| | | | |
| TS-SN2-B-trans | 2.09 | 2.18 | 9.0 |
| TS-SN2-B-trans-2 | 2.10 | 2.19 | 10.8 |
| TS-SN2-B-trans-3 | 2.09 | 2.18 | 9.4 |
| TS-SN2-B-trans-4 | 2.09 | 2.18 | 9.5 |
| TS-SN2-B-trans-5 | 2.09 | 2.19 | 9.4 |
| TS-SN2-B-trans-6 | 2.11 | 2.17 | 11.0 |
| TS-SN2-B-trans-7 | 2.08 | 2.22 | 13.2 |
| TS-SN2-B-trans-8 | 2.11 | 2.17 | 12.0 |
| TS-SN2-B-trans-9 | 2.08 | 2.22 | 9.7 |
| TS-SN2-B-trans-10 | 2.11 | 2.17 | 11.0 |
| TS-SN2-B-trans-11 | 2.11 | 2.18 | 12.7 |

**Table SI-8:** Intramolecular ring closure from the nickel dienolate intermediate for Pathway 3B-cis and trans conformations for **substrate 3**. The lowest energy conformation for each TS that corresponds with the structure in the manuscript is highlighted in red.

| Transition Structures | $r_{(C-C)}$ (Å) | $r_{(C-O)}$ (Å) | Energy (kcal/mol) |
|---|---|---|---|
| TS-SN2-B-cis-phenethyl | 2.11 | 2.16 | 0.0 |
| TS-SN2-B-cis-phenethyl-2 | 2.13 | 2.12 | 2.3 |
| TS-SN2-B-cis-phenethyl-3 | 2.12 | 2.14 | 4.4 |
| TS-SN2-B-cis-phenethyl-4 | 2.14 | 2.15 | 1.0 |
| TS-SN2-B-cis-phenethyl-5 | 2.14 | 2.17 | 1.7 |
| TS-SN2-B-cis-phenethyl-6 | 2.14 | 2.11 | 3.3 |
| TS-SN2-B-cis-phenethyl-7 | 2.12 | 2.12 | 1.1 |
| TS-SN2-B-cis-phenethyl-8 | 2.15 | 2.18 | 2.3 |
| TS-SN2-B-cis-phenethyl-9 | 2.13 | 2.08 | 0.4 |
| TS-SN2-B-cis-phenethyl-10 | 2.12 | 2.12 | 1.0 |
| TS-SN2-B-cis-phenethyl-11 | 2.12 | 2.17 | 0.3 |
| TS-SN2-B-cis-phenethyl-12 | 2.14 | 2.17 | 0.9 |
| | | | |
| TS-SN2-B-trans-phenethyl | 2.12 | 2.08 | 0.2 |
| TS-SN2-B-trans-phenethyl-2 | 2.11 | 2.17 | 2.8 |
| TS-SN2-B-trans-phenethyl-3 | 2.13 | 2.08 | 1.5 |
| TS-SN2-B-trans-phenethyl-4 | 2.10 | 2.06 | 4.8 |
| TS-SN2-B-trans-phenethyl-5 | 2.13 | 2.08 | 0.7 |
| TS-SN2-B-trans-phenethyl-6 | 2.12 | 2.14 | 1.1 |
| TS-SN2-B-trans-phenethyl-7 | 2.11 | 2.14 | 1.1 |
| TS-SN2-B-trans-phenethyl-8 | 2.13 | 2.08 | 1.5 |
| TS-SN2-B-trans-phenethyl-9 | 2.12 | 2.09 | 6.2 |
| TS-SN2-B-trans-phenethyl-10 | 2.11 | 2.08 | 3.9 |
| TS-SN2-B-trans-phenethyl-11 | 2.11 | 2.10 | 4.0 |

**Table SI-9:** Stereoinvertive and stereoretentive oxidative addition for Pathway 2. The lowest energy conformation for each TS that corresponds with the structure in the manuscript is highlighted in red.

| Transition Structures | $r_{(C\text{-}O)}$ (Å) | $r_{(Ni\text{-}C)}$ (Å) | Energy (kcal/mol) |
|---|---|---|---|
| TS-OA-Inv | 2.16 | 2.52 | 16.9 |
| TS-OA-Inv-2 | 2.15 | 2.49 | 16.9 |
| TS-OA-Inv-3 | 2.14 | 2.49 | 17.8 |
| TS-OA-Inv-4 | 2.19 | 2.46 | 21.4 |
| TS-OA-Inv-5 | 2.21 | 2.76 | 18.9 |
| TS-OA-Inv-6 | 2.21 | 2.75 | 19.3 |
| TS-OA-Inv-7 | 2.09 | 2.40 | 21.9 |
| TS-OA-Ret | 1.76 | 2.09 | 54.9 |

**Table SI-10:** Migratory Insertion – cis and trans conformers for Pathway 2. The lowest energy conformation for each TS that corresponds with the structure in the manuscript is highlighted in red.

| Transition Structures | $r_{(C\text{-}C)}$ (Å) | $r_{(Ni\text{-}C)}$ (Å) | Energy (kcal/mol) |
|---|---|---|---|
| TS-MI-cis | 1.88 | 2.20 | 7.9 |
| TS-MI-cis-2 | 1.89 | 2.18 | 8.7 |
| | | | |
| TS-MI-trans | 2.09 | 2.07 | 10.9 |
| TS-MI-trans-2 | 2.07 | 2.08 | 11.3 |
| TS-MI-trans-3 | 1.94 | 2.15 | 17.5 |
| TS-MI-trans-4 | 1.92 | 2.20 | 27.1 |
| TS-MI-trans-5 | 1.91 | 2.20 | 25.4 |
| TS-MI-trans-6 | 2.06 | 2.07 | 20.0 |
| TS-MI-trans-7 | 1.96 | 2.11 | 20.4 |

**Table SI-11:** β-Fluoride elimination for Pathway 2. The lowest energy conformation for each TS that corresponds with the structure in the manuscript is highlighted in red.

| Transition Structures | $r_{(C\text{-}F)}$ (Å) | $r_{(Ni\text{-}F)}$ (Å) | Energy (kcal/mol) |
|---|---|---|---|
| TS-BFE-cis | 1.78 | 1.98 | 13.2 |
| TS-BFE-cis-2 | 1.77 | 1.97 | 14.4 |
| TS-BFE-cis-3 | 1.78 | 1.98 | 14.1 |
| TS-BFE-cis-4 | 1.76 | 1.99 | 21.3 |
| TS-BFE-cis-5 | 1.78 | 1.97 | 18.4 |
| TS-BFE-cis-6 | 1.77 | 1.98 | 16.5 |
| TS-BFE-cis-7 | 1.77 | 1.98 | 16.5 |
| | | | |
| TS-BFE-trans | 1.78 | 1.98 | 12.8 |
| TS-BFE-trans-2 | 1.77 | 1.98 | 13.8 |
| TS-BFE-trans-3 | 1.75 | 1.99 | 14.0 |

| | | | |
|---|---|---|---|
| TS-BFE-trans-4 | 1.77 | 1.98 | 14.4 |
| TS-BFE-trans-5 | 1.75 | 1.99 | 15.4 |
| TS-BFE-trans-6 | 1.79 | 1.98 | 15.6 |
| TS-BFE-trans-7 | 1.75 | 2.02 | 16.1 |
| TS-BFE-trans-8 | 1.77 | 1.98 | 14.2 |
| TS-BFE-trans-9 | 1.75 | 2.01 | 15.4 |
| TS-BFE-trans-10 | 1.75 | 2.02 | 16.8 |
| TS-BFE-trans-11 | 1.76 | 1.95 | 17.9 |
| TS-BFE-trans-12 | 1.73 | 2.01 | 17.1 |
| TS-BFE-trans-13 | 1.76 | 2.01 | 19.3 |
| TS-BFE-trans-14 | 1.75 | 1.99 | 14.7 |
| TS-BFE-trans-15 | 1.76 | 2.00 | 14.5 |

# IV. REFERENCES FOR SUPPORTING INFORMATION


[i] Denmark, S. E.; O'Connor, S. P. Catalytic, Enantioselective Cyclopropanation of Allylic Alcohols. Substrate Generality. *J. Org. Chem*. **1997**, *62*, 584–594.

[ii] Takahashi, H.; Yoshioka, M.; Shibasaki, M.; Ohno, M.; Imai, N.; Kobayashi, S. A catalytic enantioselective reaction using a $C_2$-symmetric disulfonamide as a chiral ligand: Simmons-Smith cyclopropanation of allylic alcohols by the $Et_2Zn$-$CH_2I_2$-disulfonamide system. *Tetrahedron* **1995**, *51*, 12013–12026.

[iii] Denmark, S. E.; Christenson, B. L.; O'Connor, S. P. Catalytic enantioselective cyclopropanation with bis(halomethyl)zinc reagents. II. The effect of promoter structure on selectivity. *Tetrahedron Lett*. **1995**, *36*, 2219–2222.

[iv] Zeng, X.-L.; Deng, Z.-Y.; Liu, C.; Zhao, G.; Lin, J.-H.; Zheng, X.; Xiao, J.-C. Nucleophilic monofluoroalkylation with fluorinated phosphonium salt toward carbonyl and imine compounds. *J. Fluor. Chem*. **2017**, *193*, 17–23.

[v] Song, Y. Y.; He, H. G.; Li, Y.; Deng, Y. A facile total synthesis of amorfrutin A. *Tetrahedron Lett*. **2013**, *54*, 2658–2660.

[vi] Podunavac, M.; Lacharity, J. J.; Jones, K. E.; Zakarian, A. Stereodivergence in the Ireland–Claisen Rearrangement of α-Alkoxy Esters. *Org. Lett*. **2018**, *20*, 4867–4870.

[vii] Risatti, C. A.; Taylor, R. E. Enantioselective synthesis of cyclopropanes by aldehyde homologation. *Angew. Chem. Int. Ed*. **2004**, *43*, 6671–6672.

[viii] An, X.; Zha, Q.; Wu, Y. Perhydrolysis in Ethereal $H_2O_2$ Mediated by $MoO_2(acac)_2$: Distinct Chemoselectivity between Ketones, Ketals, and Epoxides. *Org. Lett*. **2019**, *21*, 1542–1546.

[ix] Schaubach, S.; Gebauer, K.; Ungeheuer, F.; Hoffmeister, L.; Ilg, M. K.; Wirtz, C.; Fürstner, A. A Two-Component Alkyne Metathesis Catalyst System with an Improved Substrate Scope and Functional Group Tolerance: Development and Applications to Natural Product Synthesis. *Chem. Eur. J*. **2016**, *22*, 8494–8507.

[x] Pearson, A. J.; Ghidu, V. P. Iron Tricarbonyl Stabilized Pentadienyl Cation as Initiator for Cascade Polycyclizations: A Diastereoselective Entry into Octahydrophenanthrenes. *J. Org. Chem*. **2004**, *69*, 8975–8978.

[xi] Kim, H.; Jang, J.; Shin, S. Gold-Catalyzed Asymmetric Thioallylation of Propiolates via Charge-Induced Thio-Claisen Rearrangement. *J. Am. Chem. Soc*. **2020**, *142*, 20788–20795.

[xii] Wang, X.; Zhao, S.; Liu, J.; Zhu, D.; Guo, M.; Tang, X.; Wang, G. Copper-Catalyzed C–H Difluoroalkylations and Perfluoroalkylations of Alkenes and (Hetero) arenes. *Org. Lett*. **2017**, *19*, 4187–4190.

[xiii] Marin-Luna, M.; Pölloth, B.; Zott, F.; Zipse, H. Size-dependent rate acceleration in the silylation of secondary alcohols: the bigger the faster. *Chem. Sci*. **2018**, *9*, 6509–6515.

[xiv] Li, M.; Yang, Y.; Zhou, D.; Wan, D.; You, J. Nickel-catalyzed addition-type alkenylation of unactivated, aliphatic C–H bonds with alkynes: a concise route to polysubstituted γ-butyrolactones. *Org. Lett*. **2015**, *17*, 2546–2549.

[xv] Andersen, T. L.; Frederiksen, M. W.; Domino, K.; Skrydstrup, T. Direct access to α, α-difluoroacylated arenes by palladium-catalyzed carbonylation of (hetero) aryl boronic acid derivatives. *Angew. Chem. Int. Ed*. **2016**, *55*, 10396–10400.

[xvi] Zhu, J.; Zhang, W.; Zhang, L.; Liu, J.; Zheng, J.; Hu, J. Copper-Mediated Fluoroalkylation Reactions with Iododifluoroacetamides: Controlling the Selectivity among Cross-Coupling, Intramolecular Cyclization, and Homocoupling Reactions. *J. Org. Chem*. **2010**, *75*, 5505–5512.

[xvii] Sato, K.; Omote, M.; Ando, A.; Kumadaki, I. Reactions of ethyl bromodifluoroacetate in the presence of copper powder. *J. Fluor. Chem*. **2004**, *125*, 509–515.

[xviii] Huang, X.; David, E.; Jubault, P.; Besset, T.; Couve-Bonnaire, S. Organocatalyzed sulfa-Michael addition of thiophenols on trisubstituted α-fluoroacrylates, a straightforward access to chiral fluorinated compounds. *J. Org. Chem*. **2020**, *85*, 14055–14067.

[xix] Bargiggia, F.; Dos Santos, S.; Piva, O. Asymmetric photodeconjugation: Highly stereoselective synthesis of α-fluorocarboxylic derivatives. *Synthesis* **2002**, *3*, 427–437.

[xx] Becke, A. D. *J. Chem. Phys*. **1993**, *98*, 5648–5652.

[xxi] Grimme, S.; Ehrlich, S.; Goerigk, L. Effect of the damping function in dispersion corrected density functional theory. *J. Comput. Chem*. **2011**, *32*, 1456–1465.

[xxii] Weigend, F.; Ahlrichs, R. Balanced basis sets of split valence, triple zeta valence and quadruple zeta valence quality for H to Rn: Design and assessment of accuracy. *Phys. Chem. Chem. Phys*. **2005**, *7*, 3297–3305.

[xxiii] Grimme, S.; Antony, J.; Ehrlich, S.; Krieg, H. A consistent and accurate ab initio parametrization of density functional dispersion correction (DFT-D) for the 94 elements H-Pu. *J. Chem. Phys*. **2010**, *132*, 154104.

[xxiv] Cross reference endnote xxii

[xxv] Marenich, A. V.; Cramer, C. J.; Truhlar, D. G. Universal Solvation Model Based on Solute Electron Density and on a Continuum Model of the Solvent Defined by the Bulk Dielectric Constant and Atomic Surface Tensions. *J. Phys. Chem. B* **2009**, *113*, 6378–6396.
[xxvi] Frisch, M. J.; Trucks, G. W.; Schlegel, H. B.; Scuseria, G. E.; Robb, M. A.; Cheeseman, J. R.; Scalmani, G.; Barone, V.; Petersson, G. A.; Nakatsuji, H.; Li, X.; Caricato, M.; Marenich, A. V.; Bloino, J.; Janesko, B. G.; Gomperts, R.; Mennucci, B.; Hratchian, H. P.; Ortiz, J. V.; Izmaylov, A. F.; Sonnenberg, J. L.; Williams; Ding, F.; Lipparini, F.; Egidi, F.; Goings, J.; Peng, B.; Petrone, A.; Henderson, T.; Ranasinghe, D.; Zakrzewski, V. G.; Gao, J.; Rega, N.; Zheng, G.; Liang, W.; Hada, M.; Ehara, M.; Toyota, K.; Fukuda, R.; Hasegawa, J.; Ishida, M.; Nakajima, T.; Honda, Y.; Kitao, O.; Nakai, H.; Vreven, T.; Throssell, K.; Montgomery Jr., J. A.; Peralta, J. E.; Ogliaro, F.; Bearpark, M. J.; Heyd, J. J.; Brothers, E. N.; Kudin, K. N.; Staroverov, V. N.; Keith, T. A.; Kobayashi, R.; Normand, J.; Raghavachari, K.; Rendell, A. P.; Burant, J. C.; Iyengar, S. S.; Tomasi, J.; Cossi, M.; Millam, J. M.; Klene, M.; Adamo, C.; Cammi, R.; Ochterski, J. W.; Martin, R. L.; Morokuma, K.; Farkas, O.; Foresman, J. B.; Fox, D. J. Gaussian 16 Rev. C.01. **2016**.
[xxvii] (a) Miertus, S.; Scrocco, E.; Tomasi, J. Electrostatic interaction of a solute with a continuum. A direct utilizaion of AB initio molecular potentials for the prevision of solvent effects. *Chem. Phys.* **1981**, *55*, 117–129. (b) Tomasi, J.; Mennucci, B.; Cammi, R. Quantum mechanical continuum solvation models. *Chem. Rev.* **2005**, *105*, 2999–3093.
[xxviii] Grimme, S. Supramolecular binding thermodynamics by dispersion-corrected density functional theory. *Chem. Eur. J.* **2012**, *18*, 9955–9964.
[xxix] Zhao, Y.; Truhlar, D. G. The M06 suite of density functionals for main group thermochemistry, thermochemical kinetics, noncovalent interactions, excited states, and transition elements: two new functionals and systematic testing of four M06-class functionals and 12 other functionals. *Theor. Chem. Acc.* **2008**, *120*, 215–241.
[xxx] Chai, J. D.; Head-Gordon, M. Long-range corrected hybrid density functionals with damped atom–atom dispersion corrections. *Phys. Chem. Chem. Phys.* **2008**, *10*, 6615–6620.
[xxxi] (a) Bento, A.P.; Solà, M.; Bickelhaupt, F. M. E2 and SN2 Reactions of $X^-$ + $CH_3CH_2X$ (X = F, Cl); an ab Initio and DFT Benchmark Study. *J. Chem. Theory Comput.* **2008,** *4,* 929–940. (b) cross reference endnote xxv. (c) Mardirossian, N.; Head-Gordon, M. Thirty years of density functional theory in computational chemistry: an overview and extensive assessment of 200 density functionals. *Mol. Phys.* **2017**, *115*, 2315-2372. (d) Bäcktorp, C.; Noorby, P-O. A DFT comparison of the neutral and cationic Heck pathways. *Dalton Trans.*, **2011**, *40*, 11308–11314.

1H spectrum

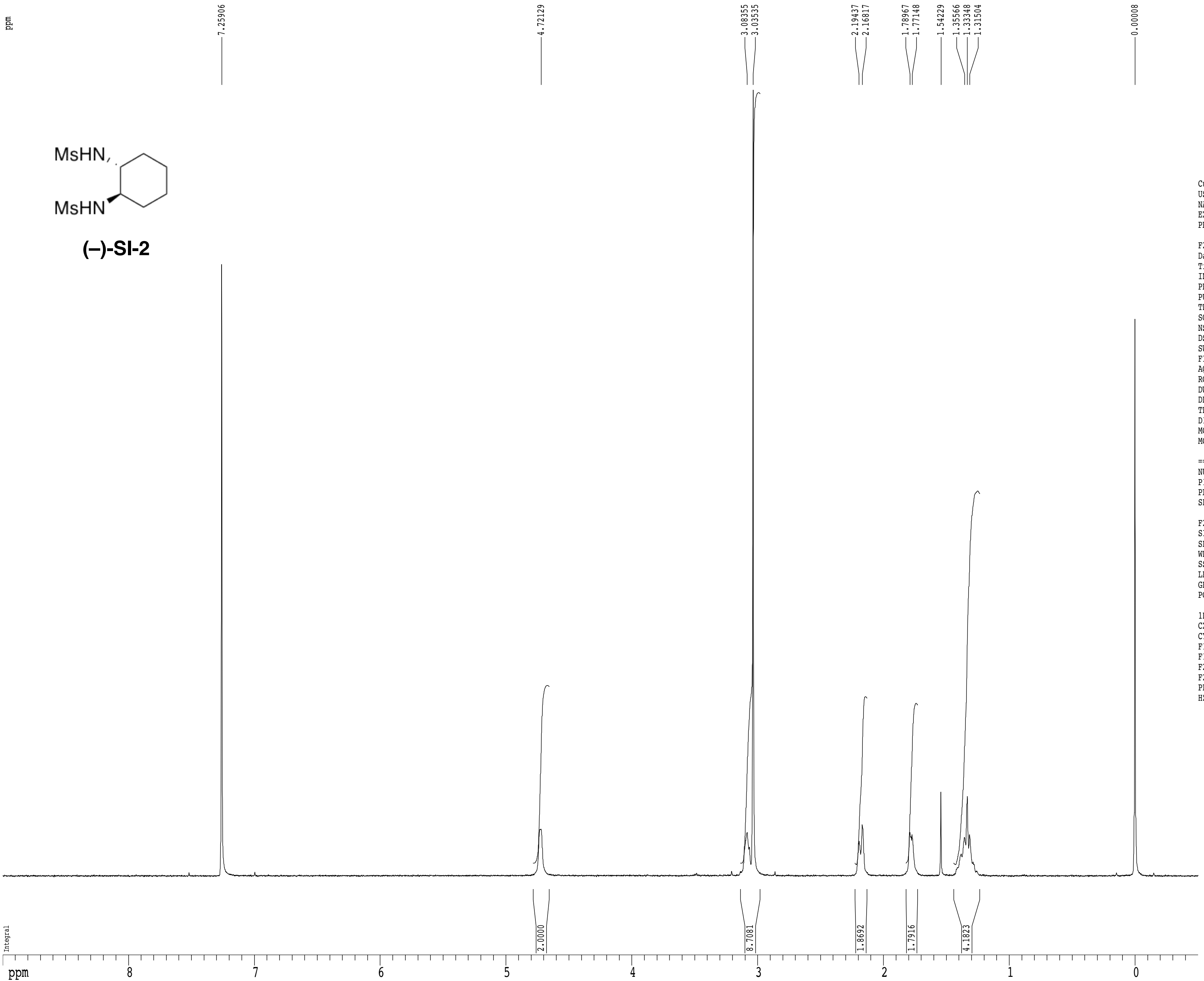


**(–)-SI-2**

```
Current Data Parameters
USER            linpc2
NAME        pcl-3-074
EXPNO                2
PROCNO               1

F2 - Acquisition Parameters
Date_         20220323
Time              8.22
INSTRUM         drx400
PROBHD   5 mm QNP H/F/P
PULPROG           zg30
TD               38460
SOLVENT          CDCl3
NS                   8
DS                   2
SWH          6410.256 Hz
FIDRES       0.166673 Hz
AQ          2.9999299 sec
RG                1024
DW              78.000 usec
DE                4.50 usec
TE               298.0 K
D1          0.10000000 sec
MCREST      0.00000000 sec
MCWRK       0.01500000 sec

======== CHANNEL f1 ========
NUC1                1H
P1               12.00 usec
PL1              -0.90 dB
SFO1       400.1328009 MHz

F2 - Processing parameters
SI               65536
SF         400.1300215 MHz
WDW                 EM
SSB                  0
LB                0.30 Hz
GB                   0
PC                2.00

1D NMR plot parameters
CX               22.80 cm
CY               15.00 cm
F1P              9.000 ppm
F1             3601.17 Hz
F2P             -0.500 ppm
F2             -200.06 Hz
PPMCM          0.41667 ppm/cm
HZCM         166.72086 Hz/cm
```

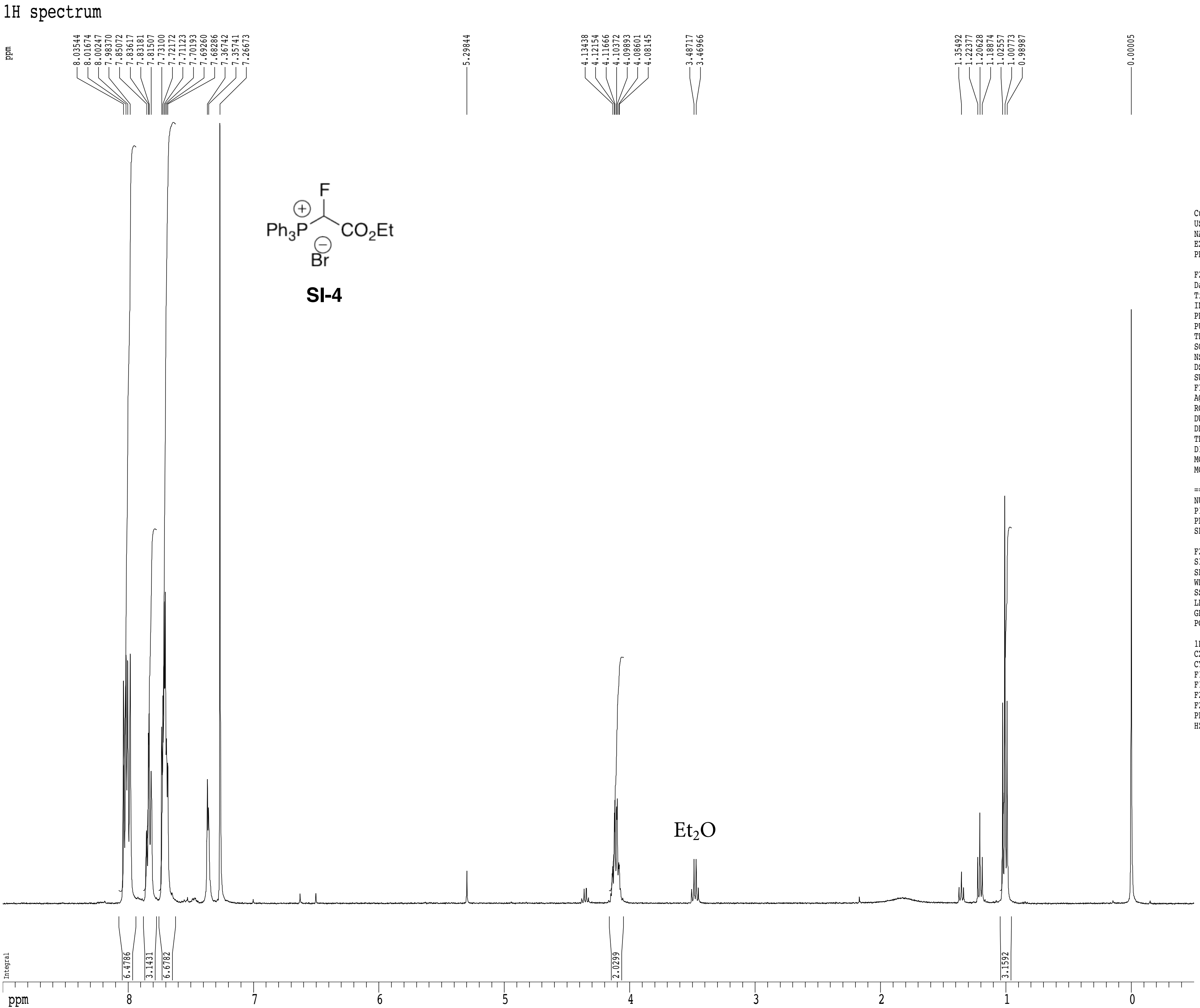


Current Data Parameters
USER linpc2
NAME pcl-3-092
EXPNO 1
PROCNO 1

F2 - Acquisition Parameters
Date_ 20220328
Time 14.42
INSTRUM drx400
PROBHD 5 mm QNP H/F/P
PULPROG zg30
TD 38460
SOLVENT CDCl3
NS 8
DS 2
SWH 6410.256 Hz
FIDRES 0.166673 Hz
AQ 2.9999299 sec
RG 645.1
DW 78.000 usec
DE 4.50 usec
TE 298.0 K
D1 0.10000000 sec
MCREST 0.00000000 sec
MCWRK 0.01500000 sec

======== CHANNEL f1 ========
NUC1 1H
P1 12.00 usec
PL1 -0.90 dB
SFO1 400.1328009 MHz

F2 - Processing parameters
SI 65536
SF 400.1300189 MHz
WDW EM
SSB 0
LB 0.30 Hz
GB 0
PC 2.00

1D NMR plot parameters
CX 22.80 cm
CY 15.00 cm
F1P 9.000 ppm
F1 3601.17 Hz
F2P -0.500 ppm
F2 -200.06 Hz
PPMCM 0.41667 ppm/cm
HZCM 166.72084 Hz/cm

1H spectrum

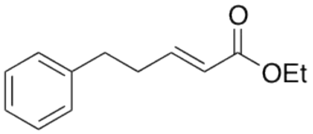

**SI-7**

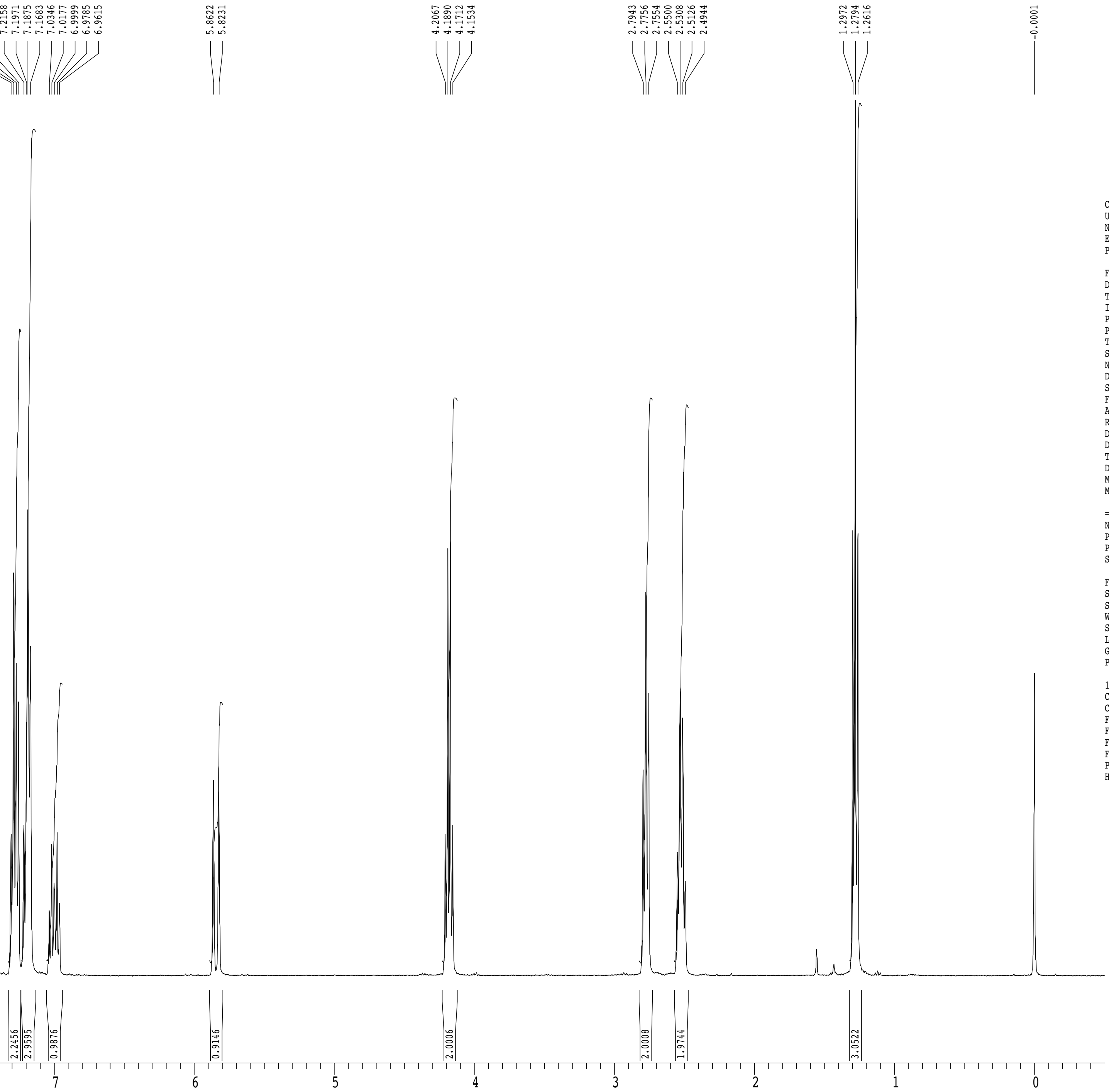


Current Data Parameters
USER linpc2
NAME pcl-3-072
EXPNO 1
PROCNO 1

F2 - Acquisition Parameters
Date_ 20220311
Time 12.51
INSTRUM drx400
PROBHD 5 mm QNP H/F/P
PULPROG zg30
TD 65536
SOLVENT CDCl3
NS 8
DS 2
SWH 6410.256 Hz
FIDRES 0.097813 Hz
AQ 5.1118579 sec
RG 287.4
DW 78.000 usec
DE 4.50 usec
TE 298.0 K
D1 0.10000000 sec
MCREST 0.00000000 sec
MCWRK 0.01500000 sec

======== CHANNEL f1 ========
NUC1 1H
P1 12.00 usec
PL1 -0.90 dB
SFO1 400.1328009 MHz

F2 - Processing parameters
SI 65536
SF 400.1300237 MHz
WDW EM
SSB 0
LB 0.30 Hz
GB 0
PC 2.00

1D NMR plot parameters
CX 22.80 cm
CY 15.00 cm
F1P 9.000 ppm
F1 3601.17 Hz
F2P -0.500 ppm
F2 -200.06 Hz
PPMCM 0.41667 ppm/cm
HZCM 166.72086 Hz/cm

1H spectrum

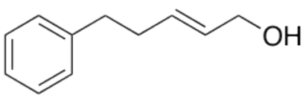


**SI-1**

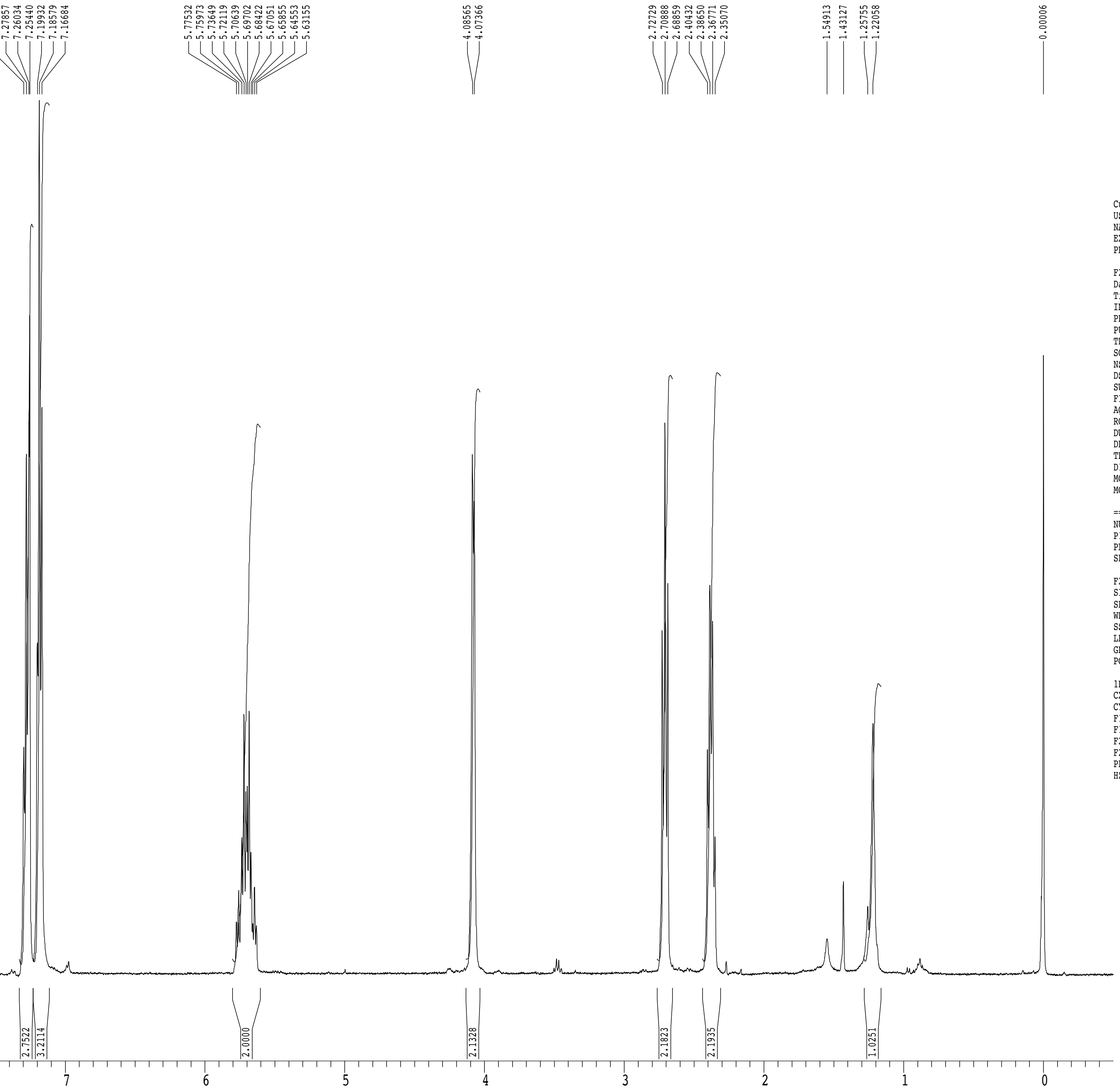


Current Data Parameters
USER linpc2
NAME pcl-3-075
EXPNO 1
PROCNO 1

F2 - Acquisition Parameters
Date_ 20220314
Time 11.51
INSTRUM drx400
PROBHD 5 mm QNP H/F/P
PULPROG zg30
TD 38460
SOLVENT CDCl3
NS 8
DS 2
SWH 6410.256 Hz
FIDRES 0.166673 Hz
AQ 2.9999299 sec
RG 406.4
DW 78.000 usec
DE 4.50 usec
TE 298.0 K
D1 0.10000000 sec
MCREST 0.00000000 sec
MCWRK 0.01500000 sec

======== CHANNEL f1 ========
NUC1 1H
P1 12.00 usec
PL1 -0.90 dB
SFO1 400.1328009 MHz

F2 - Processing parameters
SI 65536
SF 400.1300235 MHz
WDW EM
SSB 0
LB 0.30 Hz
GB 0
PC 2.00

1D NMR plot parameters
CX 22.80 cm
CY 15.00 cm
F1P 9.000 ppm
F1 3601.17 Hz
F2P -0.500 ppm
F2 -200.06 Hz
PPMCM 0.41667 ppm/cm
HZCM 166.72086 Hz/cm

1H spectrum

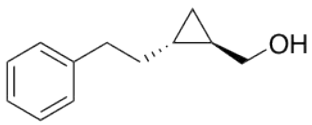

(–)-*(R,R)*-SI-3

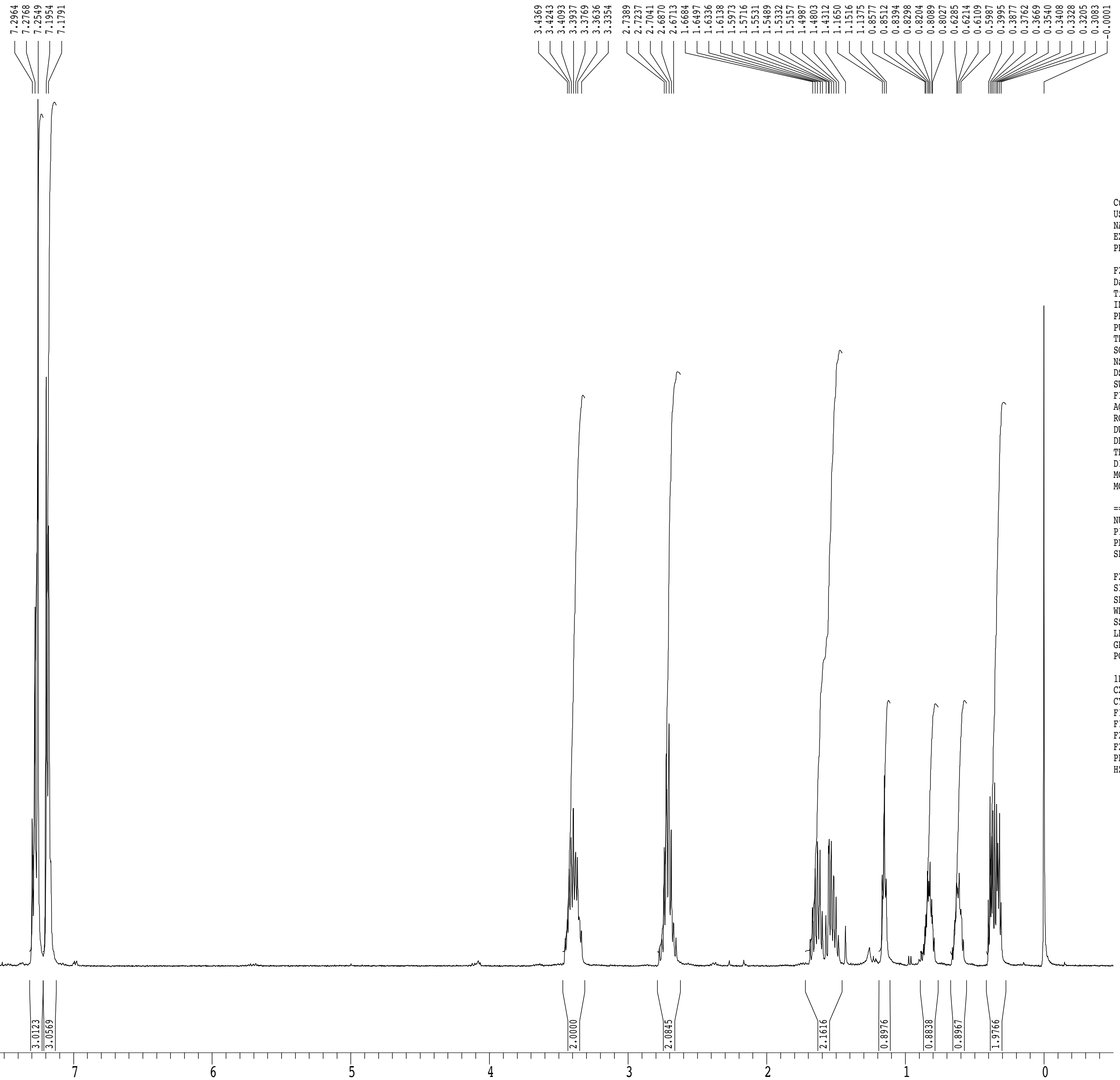


Current Data Parameters
USER linpc2
NAME pcl-3-087
EXPNO 1
PROCNO 1

F2 - Acquisition Parameters
Date_ 20220323
Time 13.32
INSTRUM drx400
PROBHD 5 mm QNP H/F/P
PULPROG zg30
TD 65536
SOLVENT CDCl3
NS 8
DS 2
SWH 6410.256 Hz
FIDRES 0.097813 Hz
AQ 5.1118579 sec
RG 456.1
DW 78.000 usec
DE 4.50 usec
TE 298.0 K
D1 0.10000000 sec
MCREST 0.00000000 sec
MCWRK 0.01500000 sec

======== CHANNEL f1 ========
NUC1 1H
P1 12.00 usec
PL1 -0.90 dB
SFO1 400.1328009 MHz

F2 - Processing parameters
SI 65536
SF 400.1300234 MHz
WDW EM
SSB 0
LB 0.30 Hz
GB 0
PC 2.00

1D NMR plot parameters
CX 22.80 cm
CY 15.00 cm
F1P 9.000 ppm
F1 3601.17 Hz
F2P -0.500 ppm
F2 -200.06 Hz
PPMCM 0.41667 ppm/cm
HZCM 166.72086 Hz/cm

**SFC Chiracel AD, 3% IPA/$CO_2$, 2.0 mL/min**

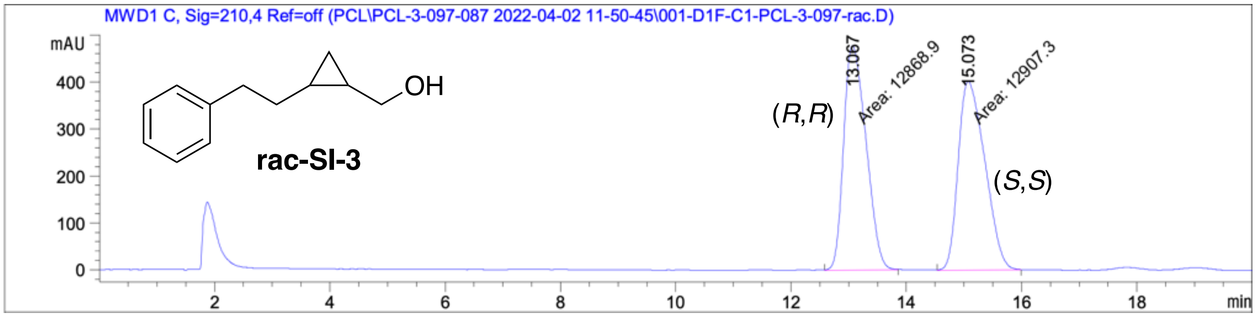


Signal 3: MWD1 C, Sig=210,4 Ref=off

| Peak # | RetTime [min] | Type | Width [min] | Area [mAU*s] | Height [mAU] | Area % |
|---|---|---|---|---|---|---|
| 1 | 13.067 | MM | 0.4489 | 1.28689e4 | 477.83917 | 49.9256 |
| 2 | 15.073 | MM | 0.5352 | 1.29073e4 | 401.93872 | 50.0744 |

Totals : 2.57762e4 879.77789

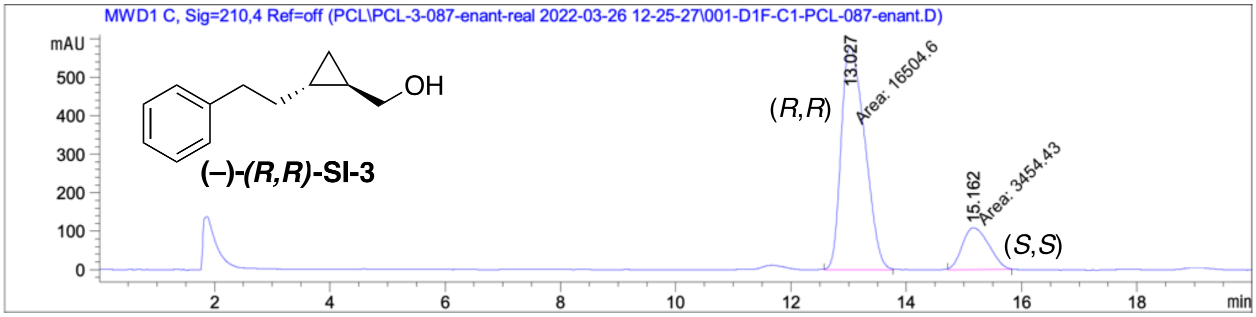


Signal 3: MWD1 C, Sig=210,4 Ref=off

| Peak # | RetTime [min] | Type | Width [min] | Area [mAU*s] | Height [mAU] | Area % |
|---|---|---|---|---|---|---|
| 1 | 13.027 | MM | 0.4725 | 1.65046e4 | 582.18030 | 82.6924 |
| 2 | 15.162 | MM | 0.5305 | 3454.42798 | 108.52956 | 17.3076 |

Totals : 1.99590e4 690.70985

1H spectrum

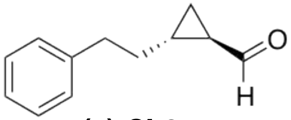

(–)-SI-8

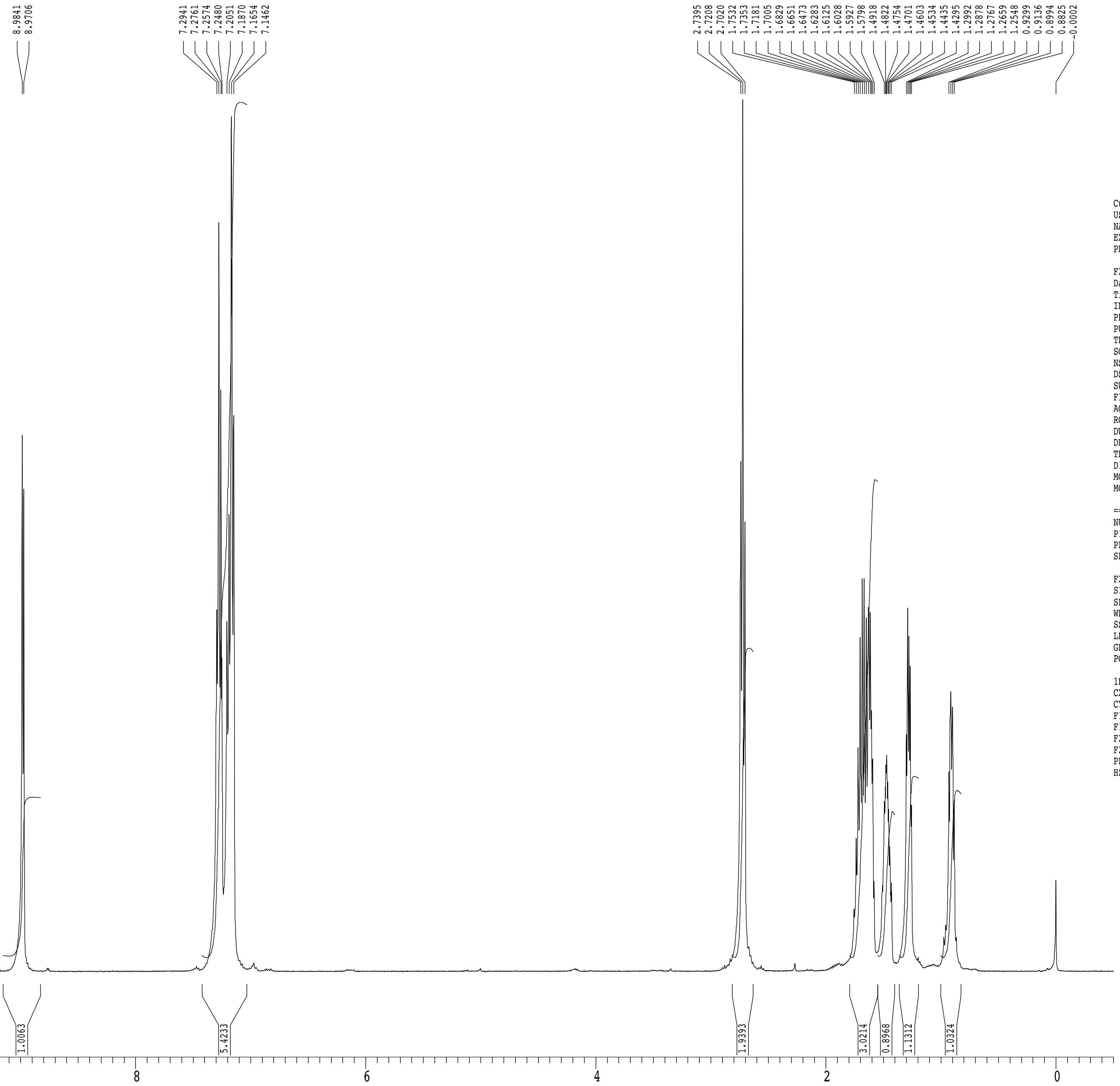


```
Current Data Parameters
USER            linpc2
NAME        pcl-3-094
EXPNO               1
PROCNO              1

F2 - Acquisition Parameters
Date_        20220329
Time             9.42
INSTRUM        drx400
PROBHD   5 mm QNP H/F/P
PULPROG          zg30
TD              38460
SOLVENT         CDCl3
NS                  8
DS                  2
SWH          6410.256 Hz
FIDRES       0.166673 Hz
AQ          2.9999299 sec
RG                128
DW             78.000 usec
DE               4.50 usec
TE              298.0 K
D1         0.10000000 sec
MCREST     0.00000000 sec
MCWRK      0.01500000 sec

======== CHANNEL f1 ========
NUC1               1H
P1              12.00 usec
PL1             -0.90 dB
SFO1      400.1328009 MHz

F2 - Processing parameters
SI              65536
SF        400.1300257 MHz
WDW                EM
SSB                 0
LB               0.30 Hz
GB                  0
PC               2.00

1D NMR plot parameters
CX              22.80 cm
CY              15.00 cm
F1P            11.000 ppm
F1            4401.43 Hz
F2P            -0.500 ppm
F2            -200.07 Hz
PPMCM         0.50439 ppm/cm
HZCM        201.81998 Hz/cm
```

1H spectrum

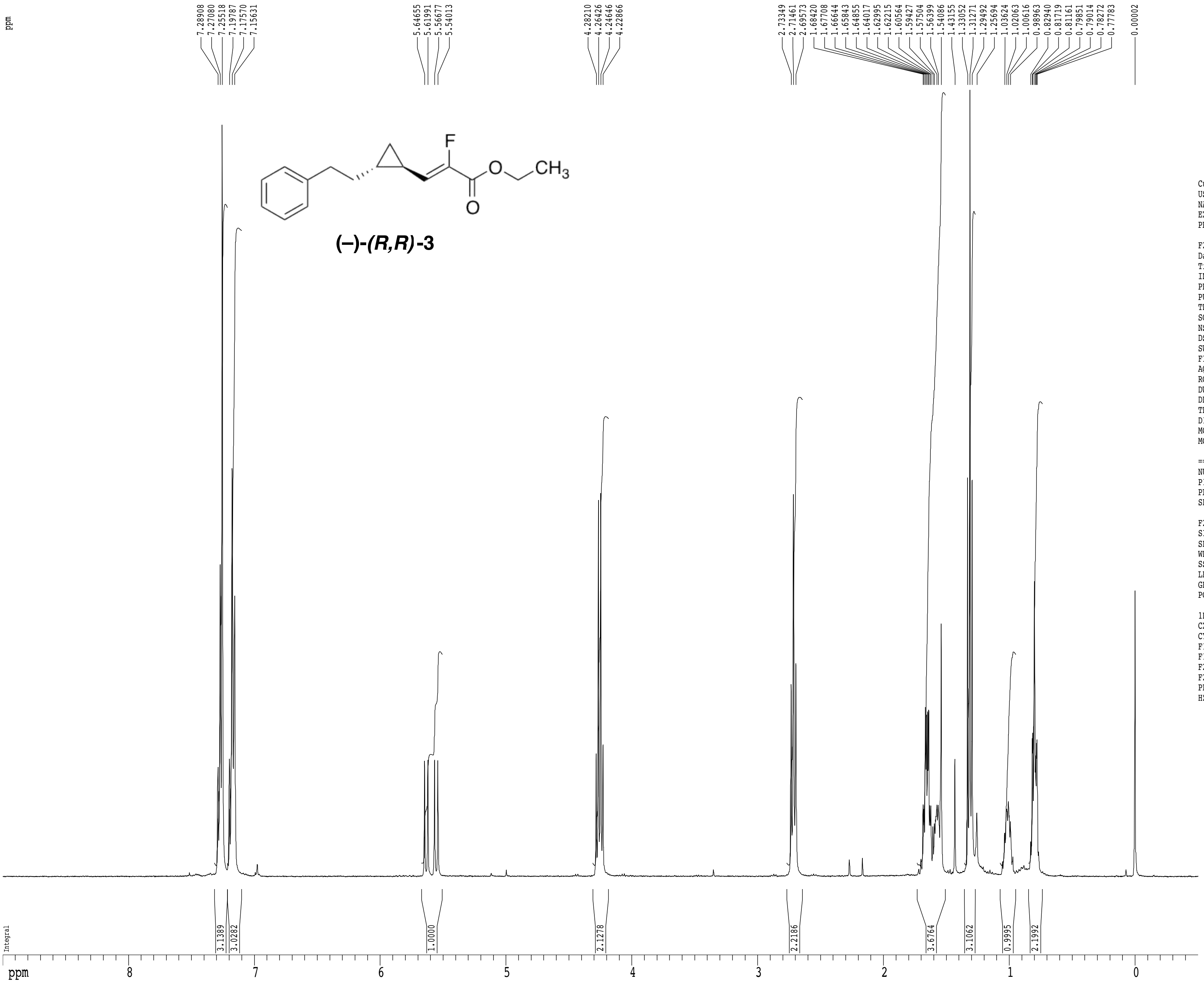


```
Current Data Parameters
USER              linpc2
NAME           pcl-3-095
EXPNO                  4
PROCNO                 1

F2 - Acquisition Parameters
Date_           20220330
Time               13.13
INSTRUM           drx400
PROBHD   5 mm QNP H/F/P
PULPROG             zg30
TD                 38460
SOLVENT            CDCl3
NS                     8
DS                     2
SWH             6410.256 Hz
FIDRES          0.166673 Hz
AQ             2.9999299 sec
RG                   362
DW                78.000 usec
DE                  4.50 usec
TE                 298.0 K
D1            0.10000000 sec
MCREST        0.00000000 sec
MCWRK         0.01500000 sec

======== CHANNEL f1 ========
NUC1                  1H
P1                 12.00 usec
PL1                -0.90 dB
SFO1         400.1328009 MHz

F2 - Processing parameters
SI                 65536
SF           400.1300229 MHz
WDW                   EM
SSB                    0
LB                  0.30 Hz
GB                     0
PC                  2.00

1D NMR plot parameters
CX                 22.80 cm
CY                 15.00 cm
F1P                9.000 ppm
F1               3601.17 Hz
F2P               -0.500 ppm
F2               -200.06 Hz
PPMCM            0.41667 ppm/cm
HZCM            166.72086 Hz/cm
```

**SFC Chiracel OD-H, 1% IPA/$CO_2$, 2.0 mL/min**

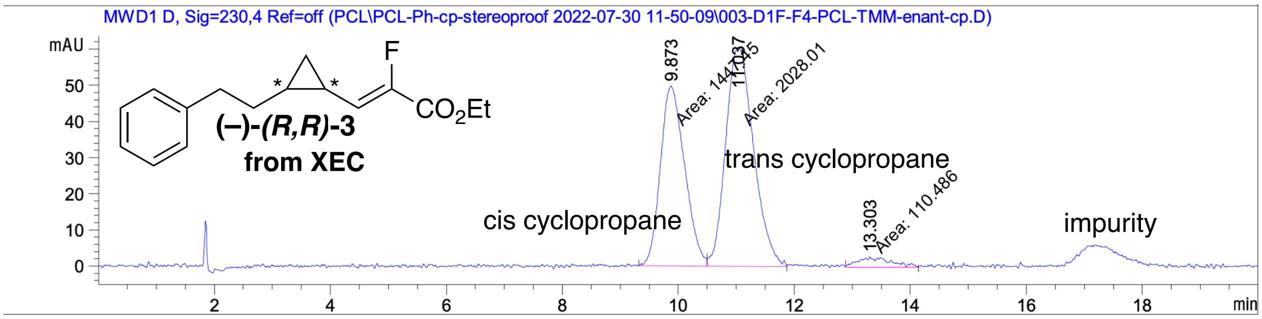


Signal 4: MWD1 D, Sig=230,4 Ref=off

| Peak # | RetTime [min] | Type | Width [min] | Area [mAU*s] | Height [mAU] | Area % |
|---|---|---|---|---|---|---|
| 1 | 9.873 | MF | 0.4859 | 1447.44983 | 49.65176 | 40.3645 |
| 2 | 11.037 | FM | 0.5572 | 2028.01355 | 60.65751 | 56.5544 |
| 3 | 13.303 | MM | 0.6396 | 110.48612 | 2.87905 | 3.0811 |

Totals : 3585.94950 113.18832

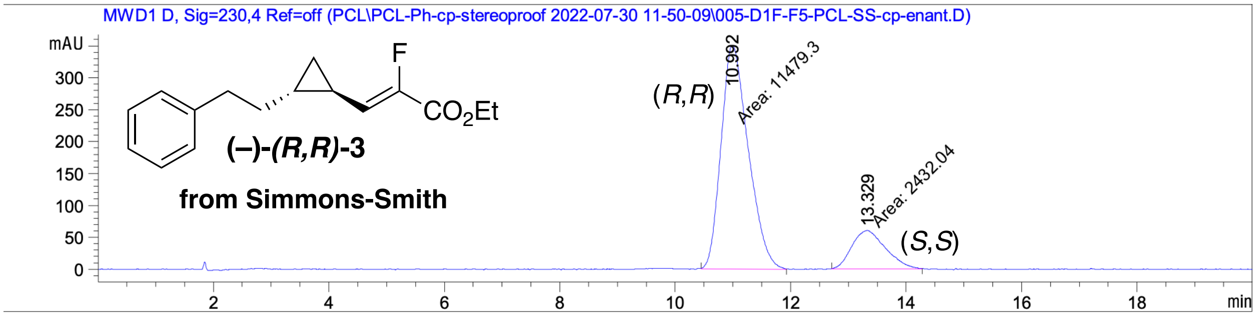


Signal 4: MWD1 D, Sig=230,4 Ref=off

| Peak # | RetTime [min] | Type | Width [min] | Area [mAU*s] | Height [mAU] | Area % |
|---|---|---|---|---|---|---|
| 1 | 10.992 | MM | 0.5460 | 1.14793e4 | 350.38983 | 82.5175 |
| 2 | 13.329 | MM | 0.6726 | 2432.04419 | 60.26916 | 17.4825 |

Totals : 1.39113e4 410.65899

1H spectrum

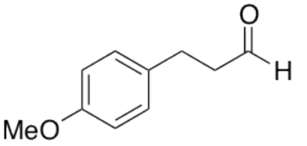

SI-9

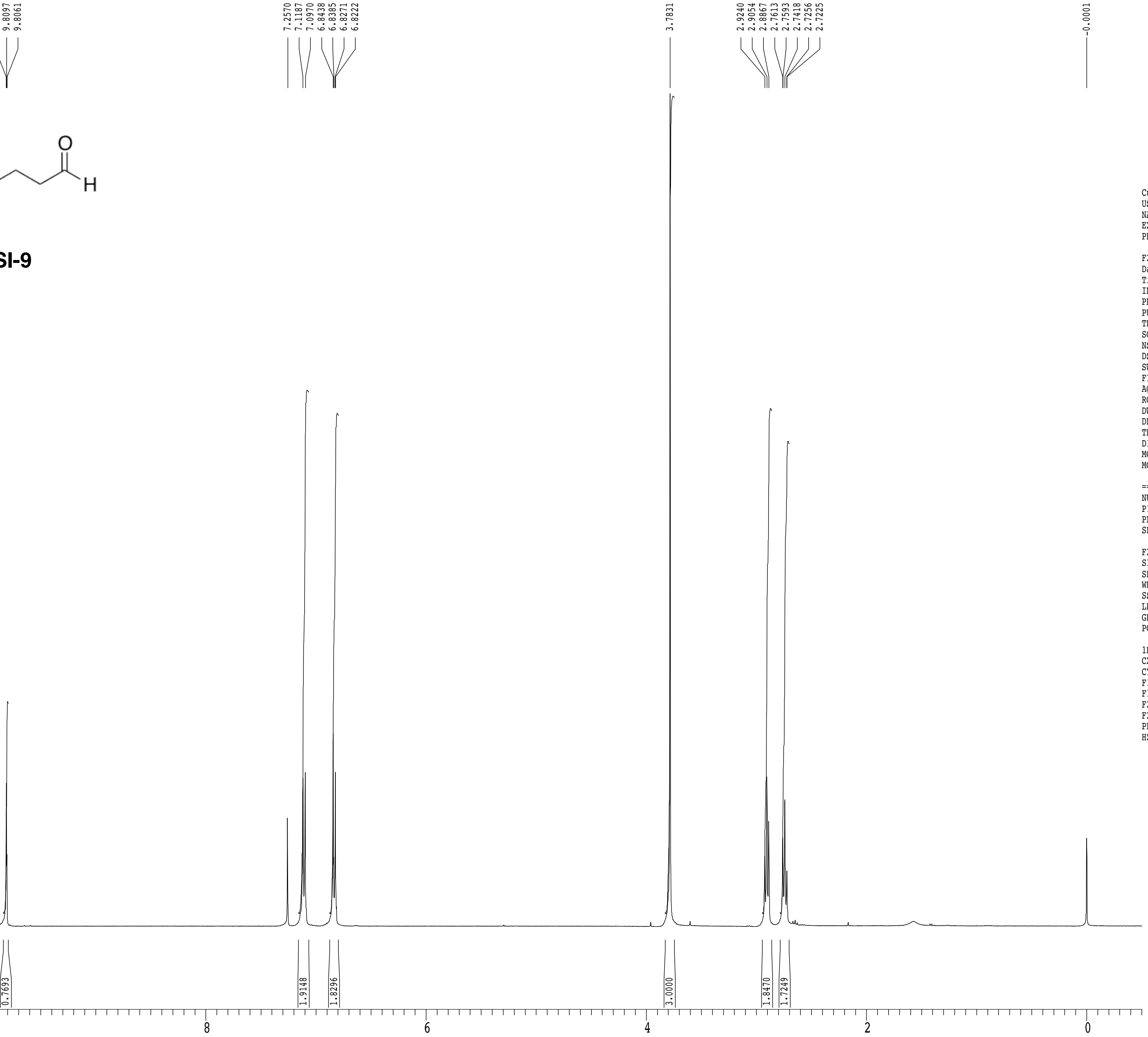


Current Data Parameters
USER linpc2
NAME pcl-2-039
EXPNO 2
PROCNO 1

F2 - Acquisition Parameters
Date_ 20210506
Time 15.07
INSTRUM drx400
PROBHD 5 mm QNP H/F/P
PULPROG zg30
TD 65536
SOLVENT CDCl3
NS 8
DS 2
SWH 6410.256 Hz
FIDRES 0.097813 Hz
AQ 5.1118579 sec
RG 406.4
DW 78.000 usec
DE 4.50 usec
TE 298.0 K
D1 0.10000000 sec
MCREST 0.00000000 sec
MCWRK 0.01500000 sec

======== CHANNEL f1 ========
NUC1 1H
P1 12.00 usec
PL1 -1.60 dB
SFO1 400.1328009 MHz

F2 - Processing parameters
SI 65536
SF 400.1300226 MHz
WDW EM
SSB 0
LB 0.30 Hz
GB 0
PC 2.00

1D NMR plot parameters
CX 22.80 cm
CY 15.00 cm
F1P 11.000 ppm
F1 4401.43 Hz
F2P -0.500 ppm
F2 -200.07 Hz
PPMCM 0.50439 ppm/cm
HZCM 201.81998 Hz/cm

1H spectrum

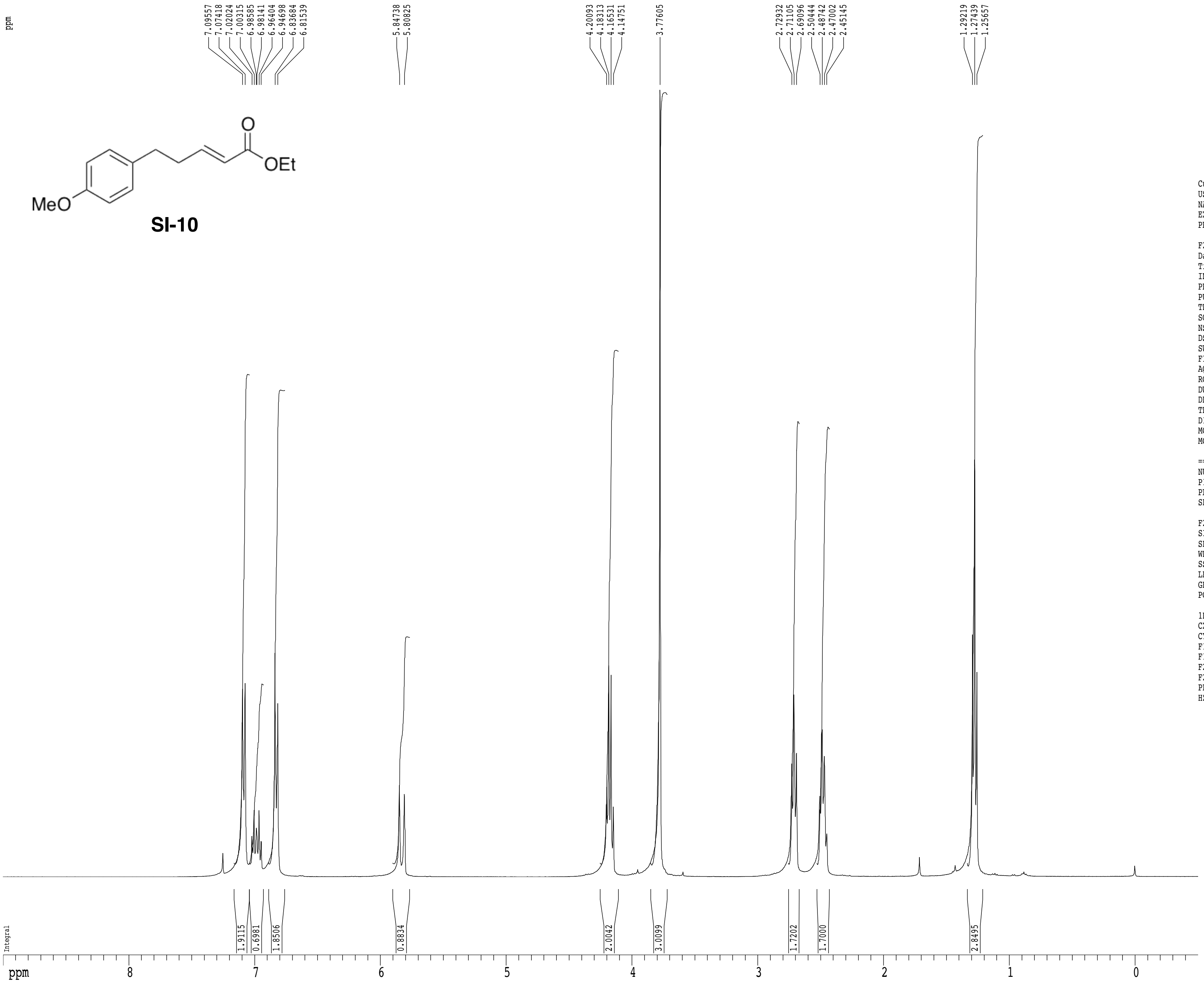


```
Current Data Parameters
USER              linpc2
NAME           pcl-3-099
EXPNO                  1
PROCNO                 1

F2 - Acquisition Parameters
Date_           20220405
Time               14.23
INSTRUM           drx400
PROBHD   5 mm QNP H/F/P
PULPROG             zg30
TD                 38460
SOLVENT            CDCl3
NS                     8
DS                     2
SWH            6410.256 Hz
FIDRES         0.166673 Hz
AQ            2.9999299 sec
RG                  71.8
DW                78.000 usec
DE                  4.50 usec
TE                 298.0 K
D1            0.10000000 sec
MCREST        0.00000000 sec
MCWRK         0.01500000 sec

======== CHANNEL f1 ========
NUC1                  1H
P1                 12.00 usec
PL1                -0.90 dB
SFO1         400.1328009 MHz

F2 - Processing parameters
SI                 65536
SF           400.1300239 MHz
WDW                   EM
SSB                    0
LB                  0.30 Hz
GB                     0
PC                  2.00

1D NMR plot parameters
CX                 22.80 cm
CY                 15.00 cm
F1P                9.000 ppm
F1               3601.17 Hz
F2P               -0.500 ppm
F2               -200.06 Hz
PPMCM            0.41667 ppm/cm
HZCM           166.72086 Hz/cm
```

1H spectrum

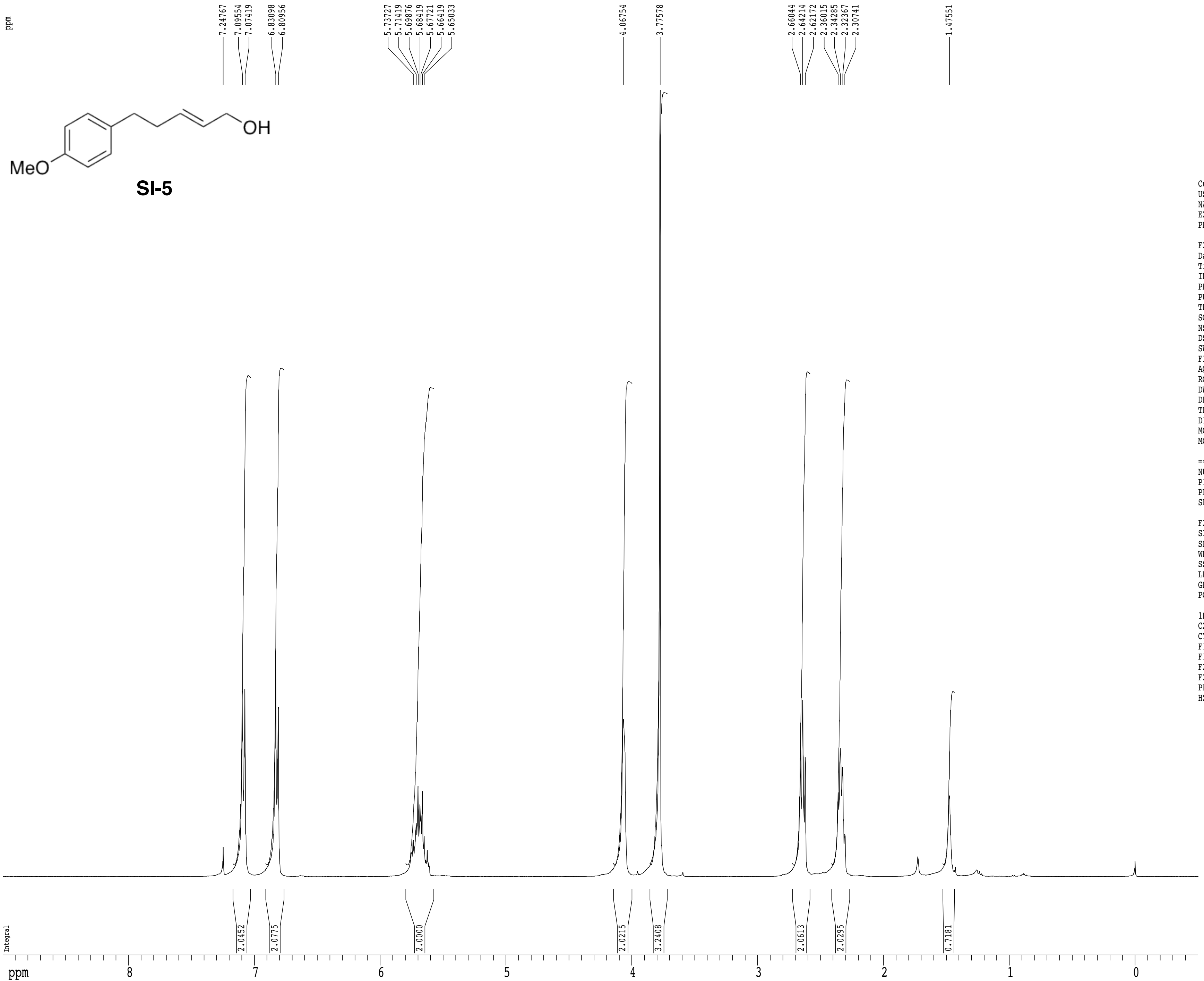


```
Current Data Parameters
USER              linpc2
NAME           pcl-3-100
EXPNO                  1
PROCNO                 1

F2 - Acquisition Parameters
Date_           20220406
Time               10.03
INSTRUM           drx400
PROBHD   5 mm QNP H/F/P
PULPROG             zg30
TD                 38460
SOLVENT            CDCl3
NS                     8
DS                     2
SWH             6410.256 Hz
FIDRES          0.166673 Hz
AQ             2.9999299 sec
RG                 101.6
DW                78.000 usec
DE                  4.50 usec
TE                 298.0 K
D1            0.10000000 sec
MCREST        0.00000000 sec
MCWRK         0.01500000 sec

======== CHANNEL f1 ========
NUC1                  1H
P1                 12.00 usec
PL1                -0.90 dB
SFO1         400.1328009 MHz

F2 - Processing parameters
SI                 65536
SF           400.1300257 MHz
WDW                   EM
SSB                    0
LB                  0.30 Hz
GB                     0
PC                  2.00

1D NMR plot parameters
CX                 22.80 cm
CY                 15.00 cm
F1P                9.000 ppm
F1               3601.17 Hz
F2P               -0.500 ppm
F2               -200.06 Hz
PPMCM            0.41667 ppm/cm
HZCM           166.72086 Hz/cm
```

1H spectrum

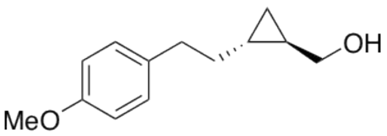

(–)-*(R,R)*-SI-6

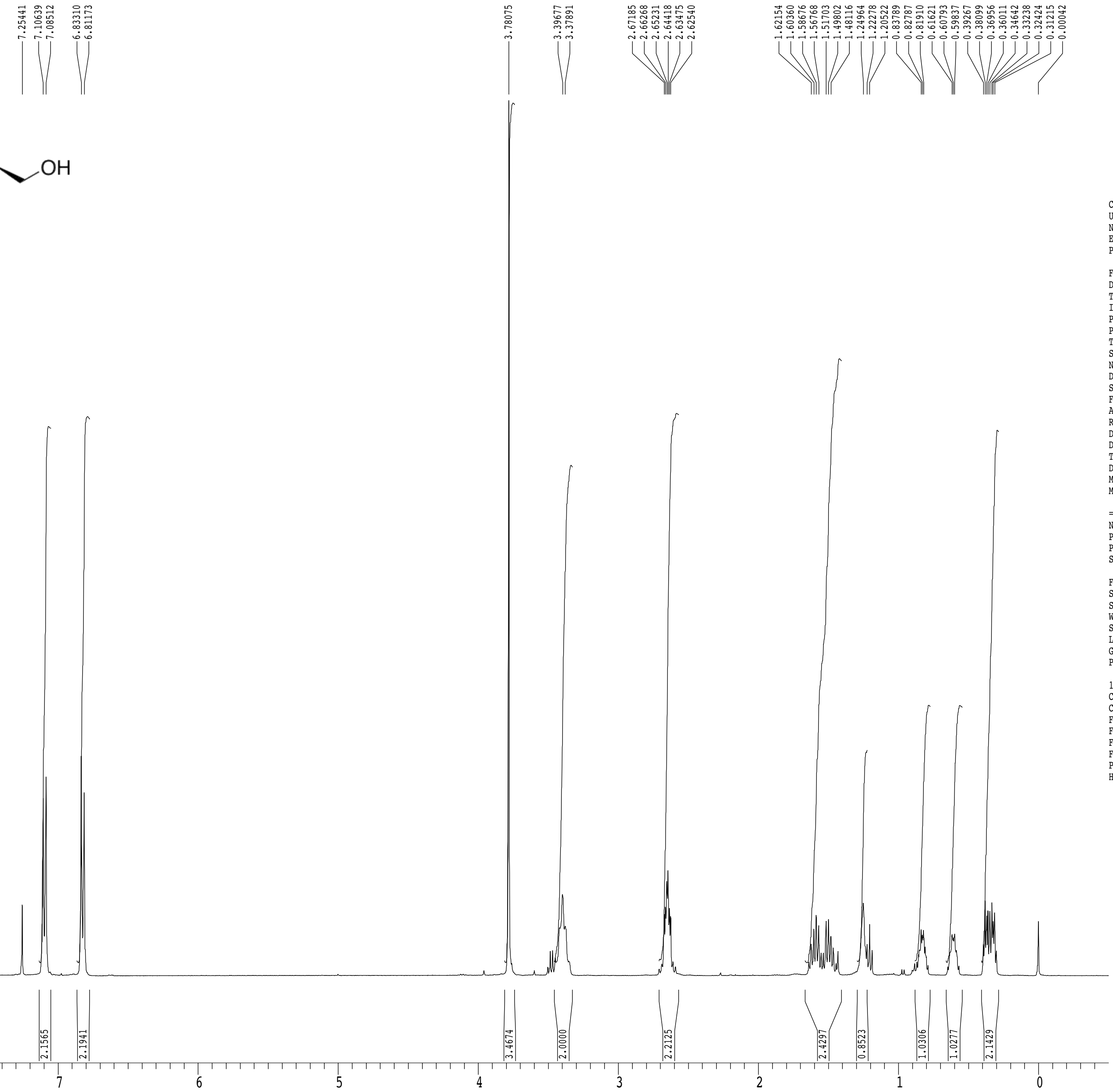


Current Data Parameters
USER linpc2
NAME pcl-3-104
EXPNO 1
PROCNO 1

F2 - Acquisition Parameters
Date_ 20220407
Time 13.54
INSTRUM drx400
PROBHD 5 mm QNP H/F/P
PULPROG zg30
TD 38460
SOLVENT CDCl3
NS 8
DS 2
SWH 6410.256 Hz
FIDRES 0.166673 Hz
AQ 2.9999299 sec
RG 161.3
DW 78.000 usec
DE 4.50 usec
TE 298.0 K
D1 0.10000000 sec
MCREST 0.00000000 sec
MCWRK 0.01500000 sec

======== CHANNEL f1 ========
NUC1 1H
P1 12.00 usec
PL1 -0.90 dB
SFO1 400.1328009 MHz

F2 - Processing parameters
SI 65536
SF 400.1300233 MHz
WDW EM
SSB 0
LB 0.30 Hz
GB 0
PC 2.00

1D NMR plot parameters
CX 22.80 cm
CY 15.00 cm
F1P 9.000 ppm
F1 3601.17 Hz
F2P -0.500 ppm
F2 -200.06 Hz
PPMCM 0.41667 ppm/cm
HZCM 166.72086 Hz/cm

13C spectrum with 1H decoupling

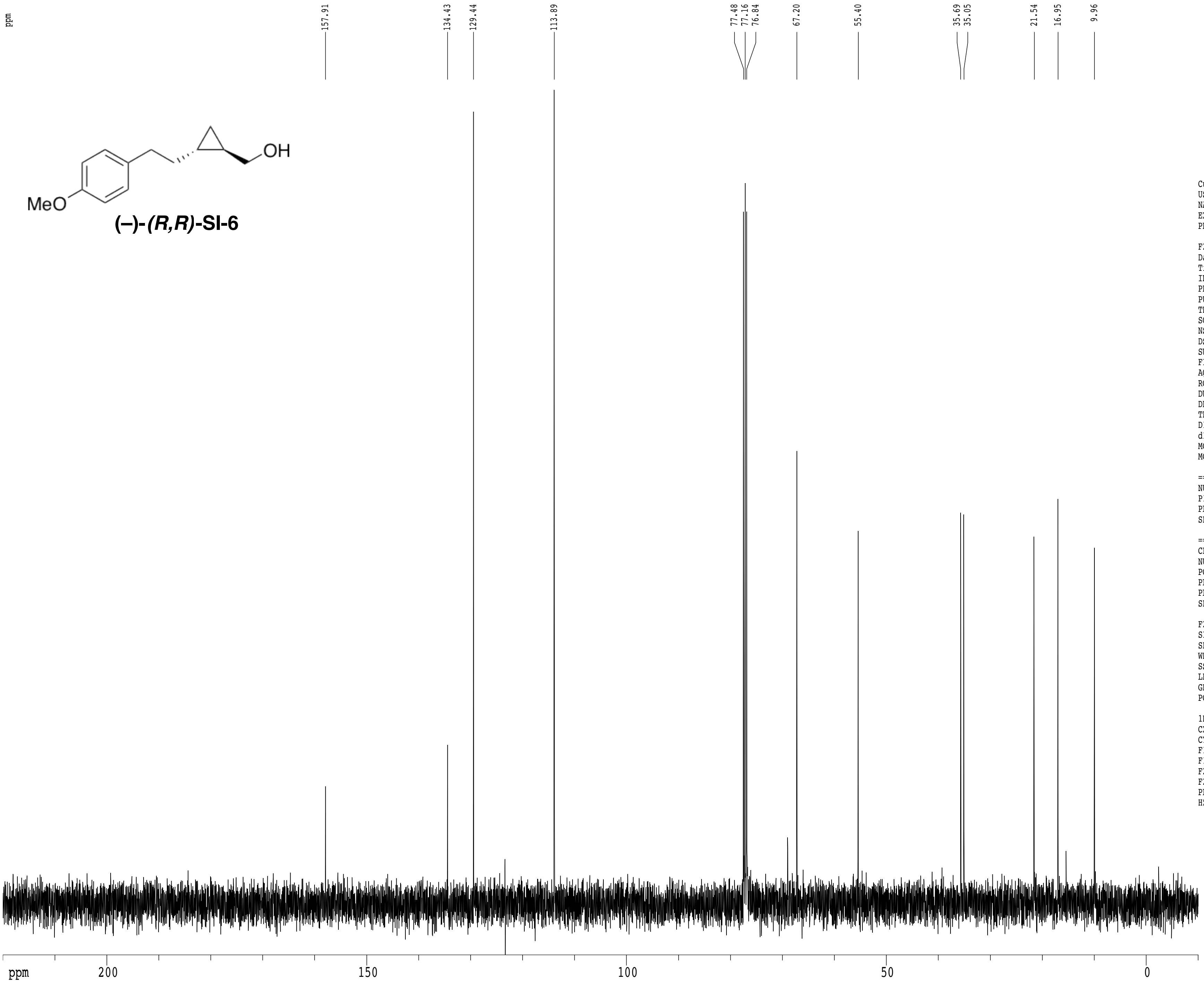


```
Current Data Parameters
USER            linpc2
NAME            pcl-3-104
EXPNO           2
PROCNO          1

F2 - Acquisition Parameters
Date_           20220407
Time            13.56
INSTRUM         drx400
PROBHD   5 mm QNP H/F/P
PULPROG         zgdc30
TD              65536
SOLVENT         CDCl3
NS              88
DS              4
SWH             24154.590 Hz
FIDRES          0.368570 Hz
AQ              1.3566452 sec
RG              14596.5
DW              20.700 usec
DE              20.39 usec
TE              298.0 K
D1              0.10000000 sec
d11             0.03000000 sec
MCREST          0.00000000 sec
MCWRK           0.01500000 sec

======== CHANNEL f1 ========
NUC1            13C
P1              7.90 usec
PL1             -3.00 dB
SFO1            100.6237964 MHz

======== CHANNEL f2 ========
CPDPRG2         waltz16
NUC2            1H
PCPD2           90.00 usec
PL2             -0.90 dB
PL12            17.00 dB
SFO2            400.1328009 MHz

F2 - Processing parameters
SI              65536
SF              100.6127584 MHz
WDW             EM
SSB             0
LB              1.00 Hz
GB              0
PC              1.00

1D NMR plot parameters
CX              22.80 cm
CY              15.50 cm
F1P             220.000 ppm
F1              22134.81 Hz
F2P             -10.000 ppm
F2              -1006.13 Hz
PPMCM           10.08772 ppm/cm
HZCM            1014.95325 Hz/cm
```

**SFC Chiracel AD, 3% IPA/$CO_2$, 2.0 mL/min**

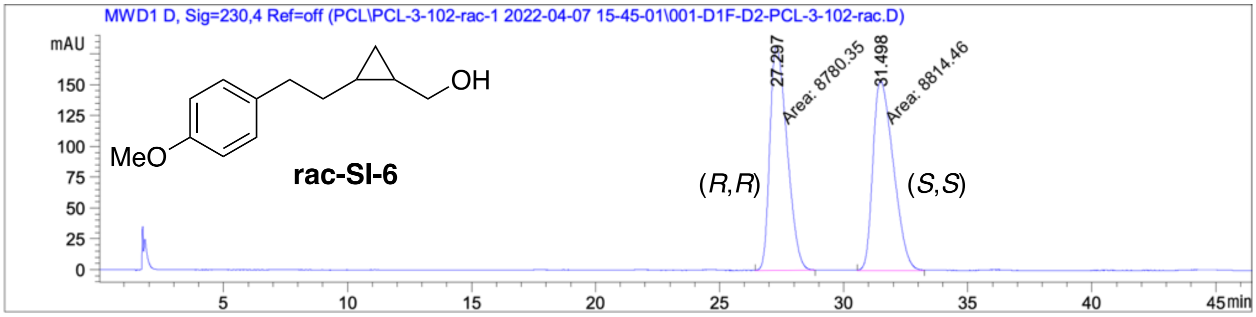


Signal 4: MWD1 D, Sig=230,4 Ref=off

| Peak # | RetTime [min] | Type | Width [min] | Area [mAU*s] | Height [mAU] | Area % |
|---|---|---|---|---|---|---|
| 1 | 27.297 | MM | 0.8035 | 8780.34766 | 182.12155 | 49.9031 |
| 2 | 31.498 | MM | 0.9561 | 8814.45703 | 153.64972 | 50.0969 |

Totals : 1.75948e4 335.77127

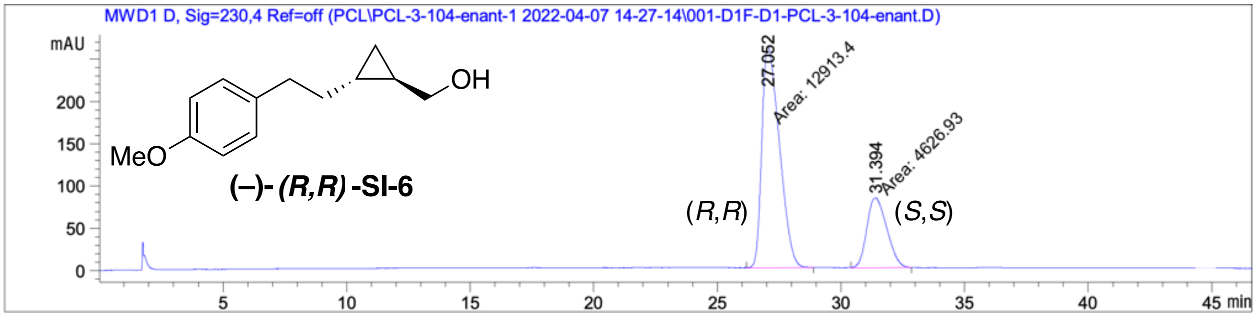


Signal 4: MWD1 D, Sig=230,4 Ref=off

| Peak # | RetTime [min] | Type | Width [min] | Area [mAU*s] | Height [mAU] | Area % |
|---|---|---|---|---|---|---|
| 1 | 27.052 | MM | 0.8207 | 1.29134e4 | 262.22864 | 73.6212 |
| 2 | 31.394 | MM | 0.9333 | 4626.92822 | 82.62690 | 26.3788 |

Totals : 1.75403e4 344.85554

1H spectrum

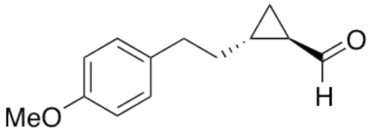


**(–)-SI-11**

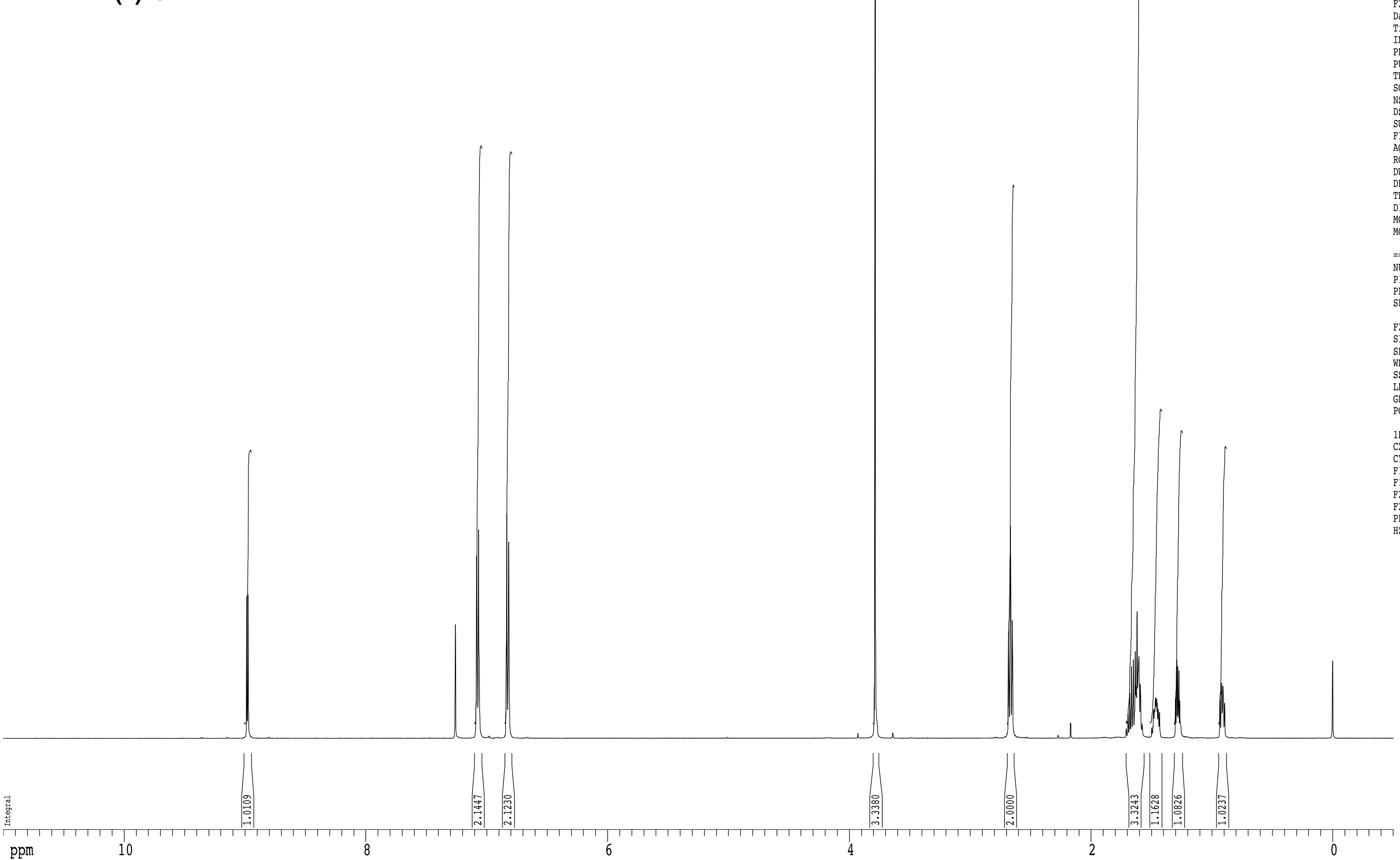


Current Data Parameters
USER linpc2
NAME pcl-3-103
EXPNO 3
PROCNO 1

F2 - Acquisition Parameters
Date_ 20220409
Time 12.36
INSTRUM gn500
PROBHD 5 mm broadband
PULPROG zg30
TD 48074
SOLVENT CDCl3
NS 8
DS 2
SWH 8012.820 Hz
FIDRES 0.166677 Hz
AQ 2.9998677 sec
RG 724.1
DW 62.400 usec
DE 6.00 usec
TE 298.0 K
D1 0.10000000 sec
MCREST 0.00000000 sec
MCWRK 0.01500000 sec

======== CHANNEL f1 ========
NUC1 1H
P1 12.00 usec
PL1 -6.00 dB
SFO1 498.6534906 MHz

F2 - Processing parameters
SI 65536
SF 498.6500291 MHz
WDW EM
SSB 0
LB 0.30 Hz
GB 0
PC 1.00

1D NMR plot parameters
CX 22.80 cm
CY 15.00 cm
F1P 11.000 ppm
F1 5485.15 Hz
F2P -0.500 ppm
F2 -249.33 Hz
PPMCM 0.50439 ppm/cm
HZCM 251.51208 Hz/cm

13C spectrum with 1H decoupling

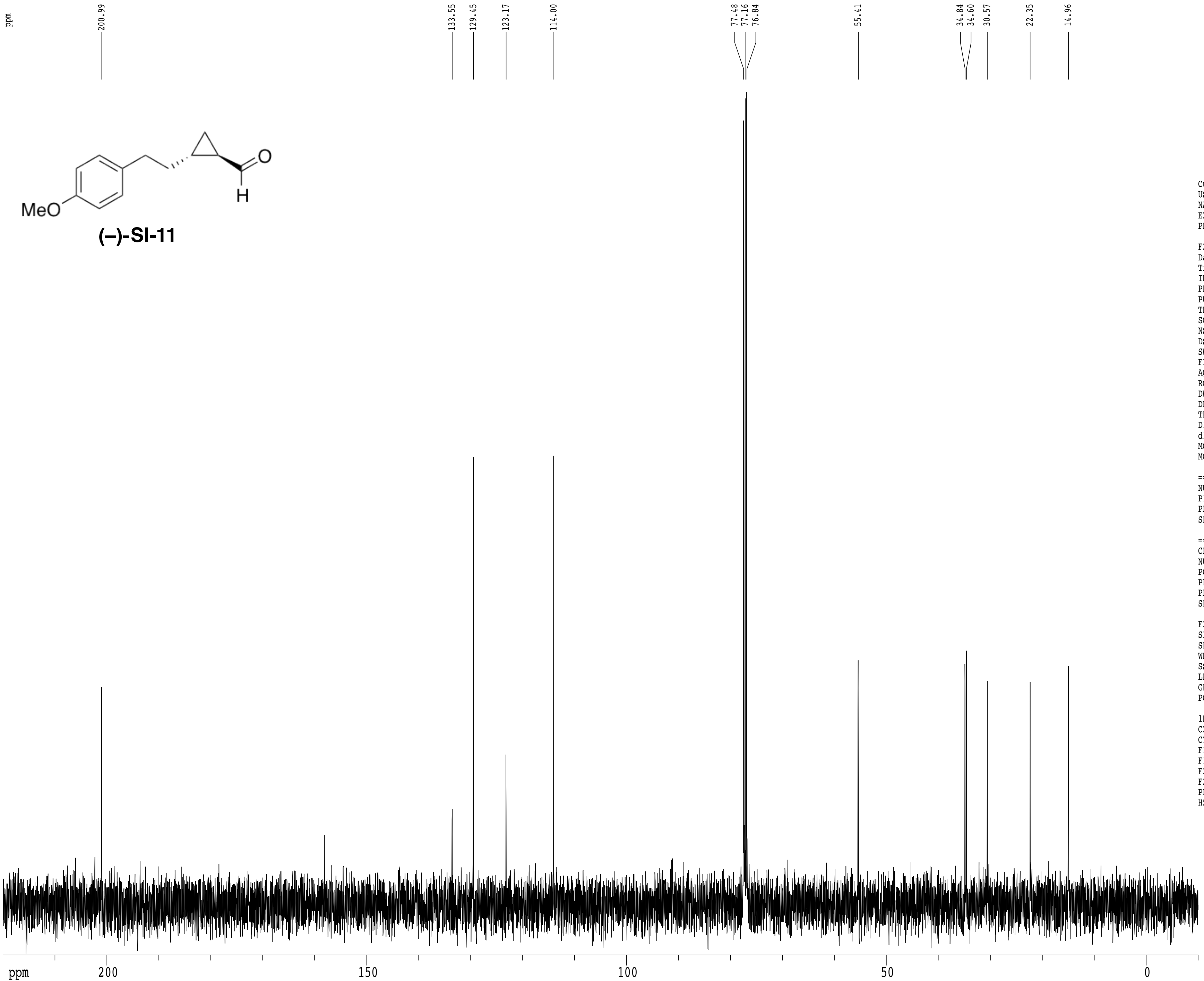


**(–)-SI-11**

```
Current Data Parameters
USER              linpc2
NAME           pcl-3-103
EXPNO                  2
PROCNO                 1

F2 - Acquisition Parameters
Date_           20220408
Time               14.42
INSTRUM           drx400
PROBHD   5 mm QNP H/F/P
PULPROG           zgdc30
TD                 65536
SOLVENT            CDCl3
NS                    88
DS                     4
SWH           24154.590 Hz
FIDRES         0.368570 Hz
AQ            1.3566452 sec
RG                9195.2
DW                20.700 usec
DE                 20.39 usec
TE                 298.0 K
D1            0.10000000 sec
d11           0.03000000 sec
MCREST        0.00000000 sec
MCWRK         0.01500000 sec

======== CHANNEL f1 ========
NUC1                 13C
P1                  7.90 usec
PL1                -3.00 dB
SFO1         100.6237964 MHz

======== CHANNEL f2 ========
CPDPRG2          waltz16
NUC2                  1H
PCPD2              90.00 usec
PL2                -0.90 dB
PL12               17.00 dB
SFO2         400.1328009 MHz

F2 - Processing parameters
SI                 65536
SF           100.6127576 MHz
WDW                   EM
SSB                    0
LB                  1.00 Hz
GB                     0
PC                  1.00

1D NMR plot parameters
CX                 22.80 cm
CY                 15.50 cm
F1P              220.000 ppm
F1              22134.81 Hz
F2P              -10.000 ppm
F2              -1006.13 Hz
PPMCM           10.08772 ppm/cm
HZCM          1014.95325 Hz/cm
```

1H spectrum

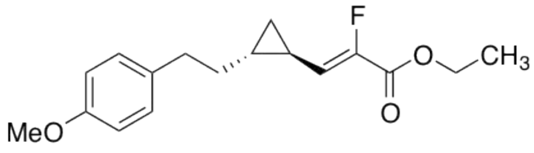


**(–)-*(R,R)*-4**

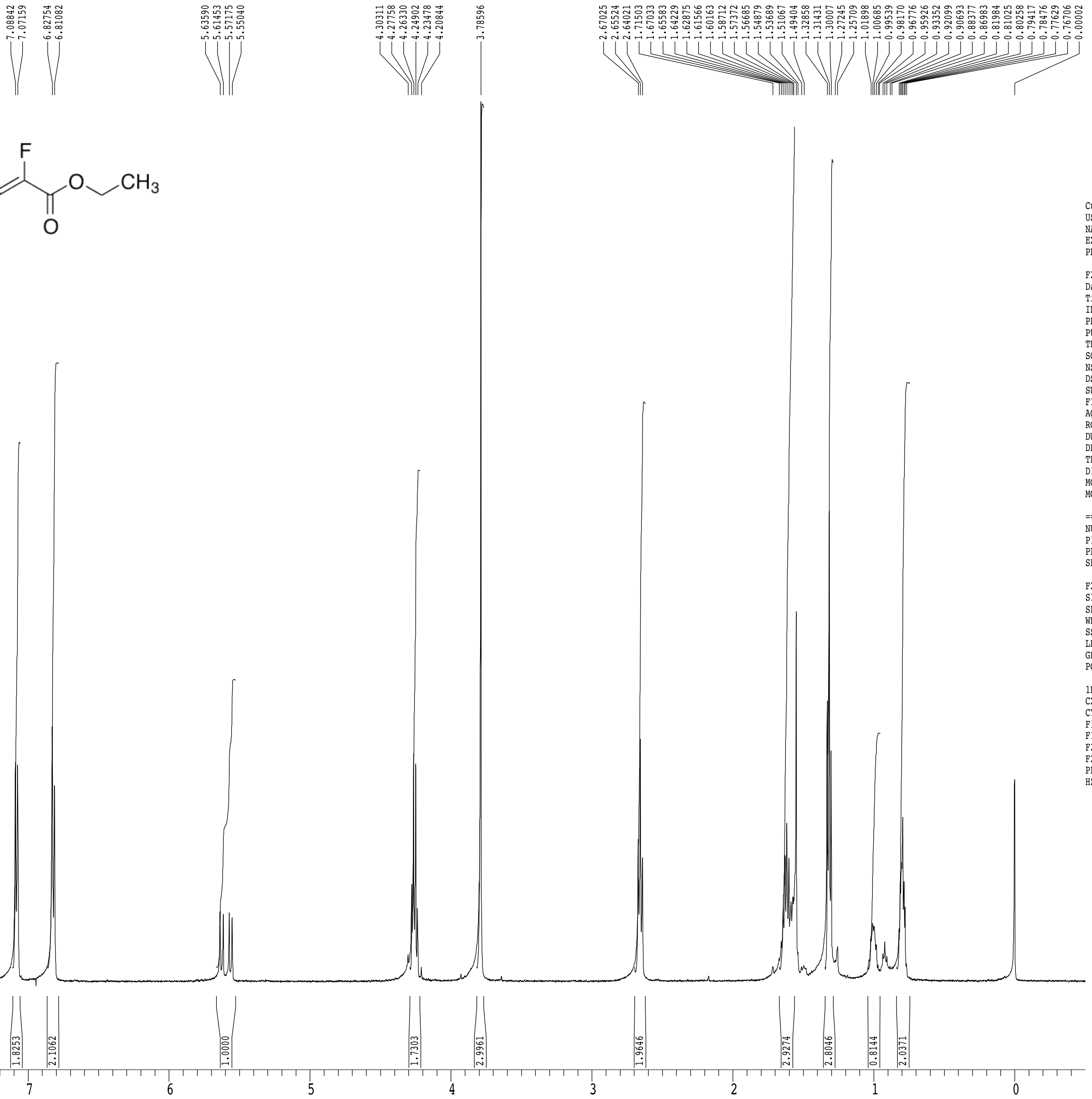


Current Data Parameters
USER linpc2
NAME pcl-3-105
EXPNO 2
PROCNO 1

F2 - Acquisition Parameters
Date_ 20220409
Time 12.34
INSTRUM gn500
PROBHD 5 mm broadband
PULPROG zg30
TD 48074
SOLVENT CDCl3
NS 8
DS 2
SWH 8012.820 Hz
FIDRES 0.166677 Hz
AQ 2.9998677 sec
RG 1625.5
DW 62.400 usec
DE 6.00 usec
TE 298.0 K
D1 0.10000000 sec
MCREST 0.00000000 sec
MCWRK 0.01500000 sec

======== CHANNEL f1 ========
NUC1 1H
P1 12.00 usec
PL1 -6.00 dB
SFO1 498.6534906 MHz

F2 - Processing parameters
SI 65536
SF 498.6500281 MHz
WDW EM
SSB 0
LB 0.30 Hz
GB 0
PC 1.00

1D NMR plot parameters
CX 22.80 cm
CY 15.00 cm
F1P 9.000 ppm
F1 4487.85 Hz
F2P -0.500 ppm
F2 -249.32 Hz
PPMCM 0.41667 ppm/cm
HZCM 207.77084 Hz/cm

**SFC Chiracel OD-H, 0.1% IPA/$CO_2$, 2.0 mL/min**

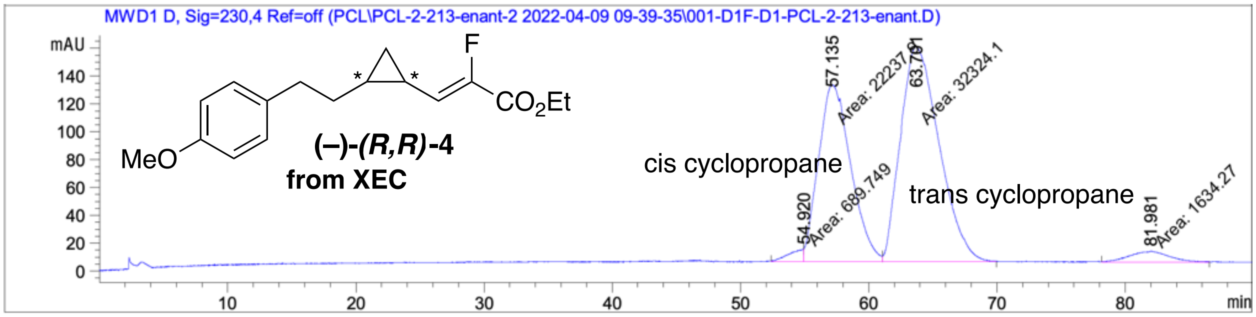


```
Signal 4: MWD1 D, Sig=230,4 Ref=off

Peak RetTime Type  Width     Area      Height     Area
  #   [min]        [min]   [mAU*s]     [mAU]        %
----|-------|----|-------|----------|----------|--------|
   1  54.920 MF    1.2951  689.74902    8.87626   1.2125
   2  57.135 MF    2.9245 2.22379e4   126.73267  39.0920
   3  63.701 FM    3.4880 3.23241e4   154.45380  56.8226
   4  81.981 MM    3.4452 1634.26892    7.90596   2.8729

Totals :                  5.68860e4   297.96868
```

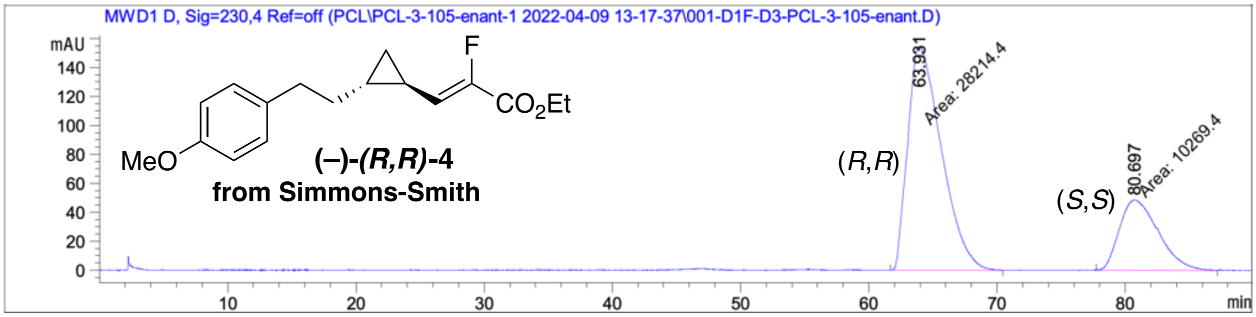


```
Signal 4: MWD1 D, Sig=230,4 Ref=off

Peak RetTime Type  Width     Area      Height     Area
  #   [min]        [min]   [mAU*s]     [mAU]        %
----|-------|----|-------|----------|----------|--------|
   1  63.931 MM    3.0407 2.82144e4   154.65009  73.3150
   2  80.697 MM    3.5293 1.02694e4    48.49546  26.6850

Totals :                  3.84838e4   203.14555
```

1H spectrum

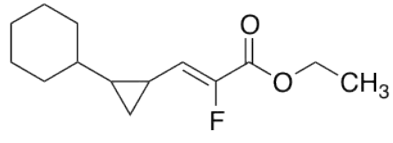


**2a**

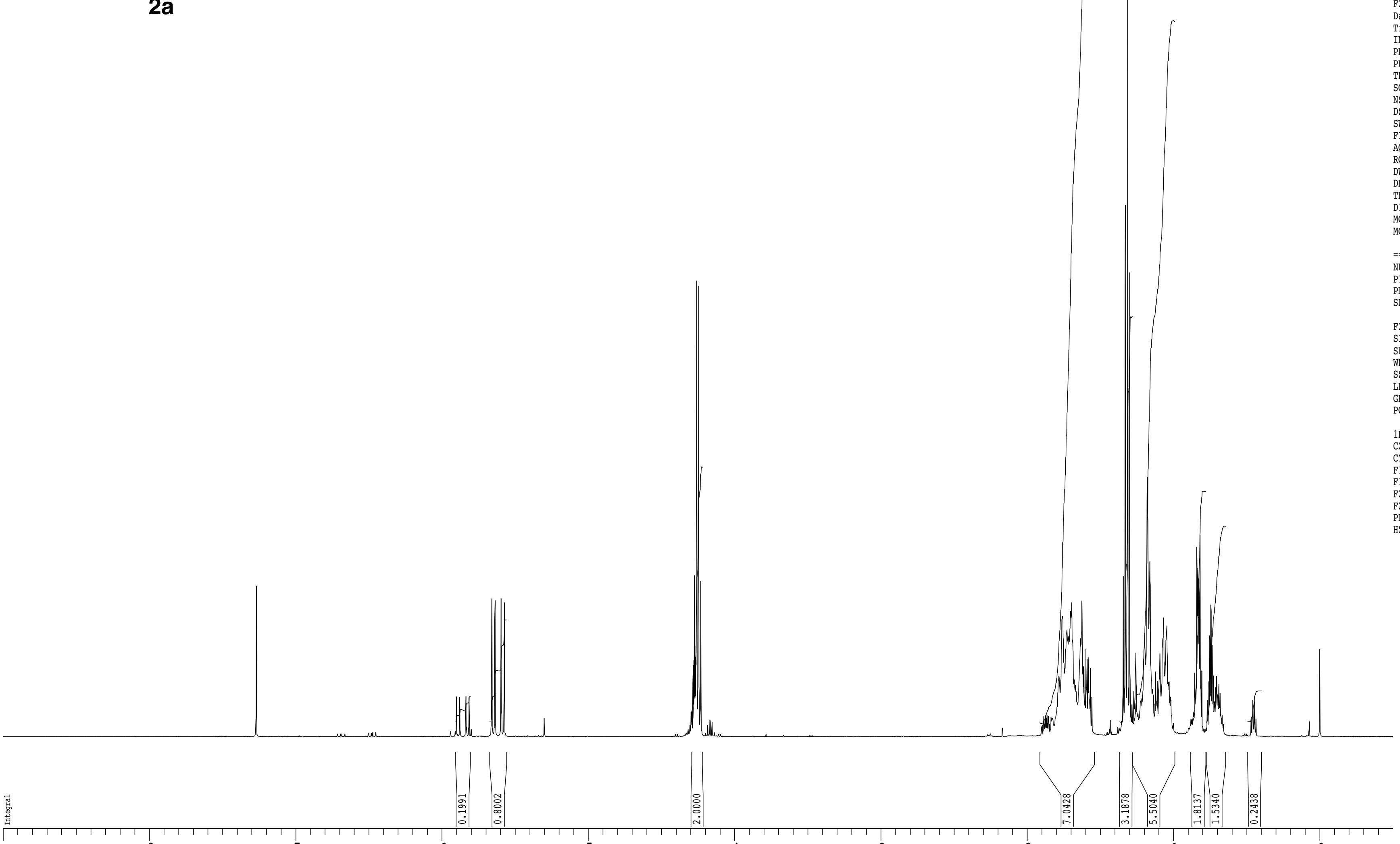


Current Data Parameters
USER linpc2
NAME pcl-2-033
EXPNO 2
PROCNO 1

F2 - Acquisition Parameters
Date_ 20210607
Time 12.21
INSTRUM cryo500
PROBHD 5 mm CPTCI 1H-
PULPROG zg30
TD 81728
SOLVENT CDCl3
NS 8
DS 2
SWH 8012.820 Hz
FIDRES 0.098043 Hz
AQ 5.0998774 sec
RG 4.5
DW 62.400 usec
DE 6.00 usec
TE 298.0 K
D1 0.10000000 sec
MCREST 0.00000000 sec
MCWRK 0.01500000 sec

======== CHANNEL f1 ========
NUC1 1H
P1 9.75 usec
PL1 1.60 dB
SFO1 500.2235015 MHz

F2 - Processing parameters
SI 65536
SF 500.2200266 MHz
WDW EM
SSB 0
LB 0.30 Hz
GB 0
PC 1.00

1D NMR plot parameters
CX 22.80 cm
CY 15.00 cm
F1P 9.000 ppm
F1 4501.98 Hz
F2P -0.500 ppm
F2 -250.11 Hz
PPMCM 0.41667 ppm/cm
HZCM 208.42502 Hz/cm

Z-restored spin-echo 13C spectrum with 1H decoupling

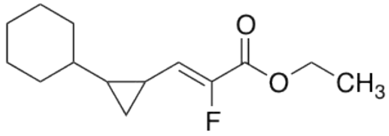

**2a**

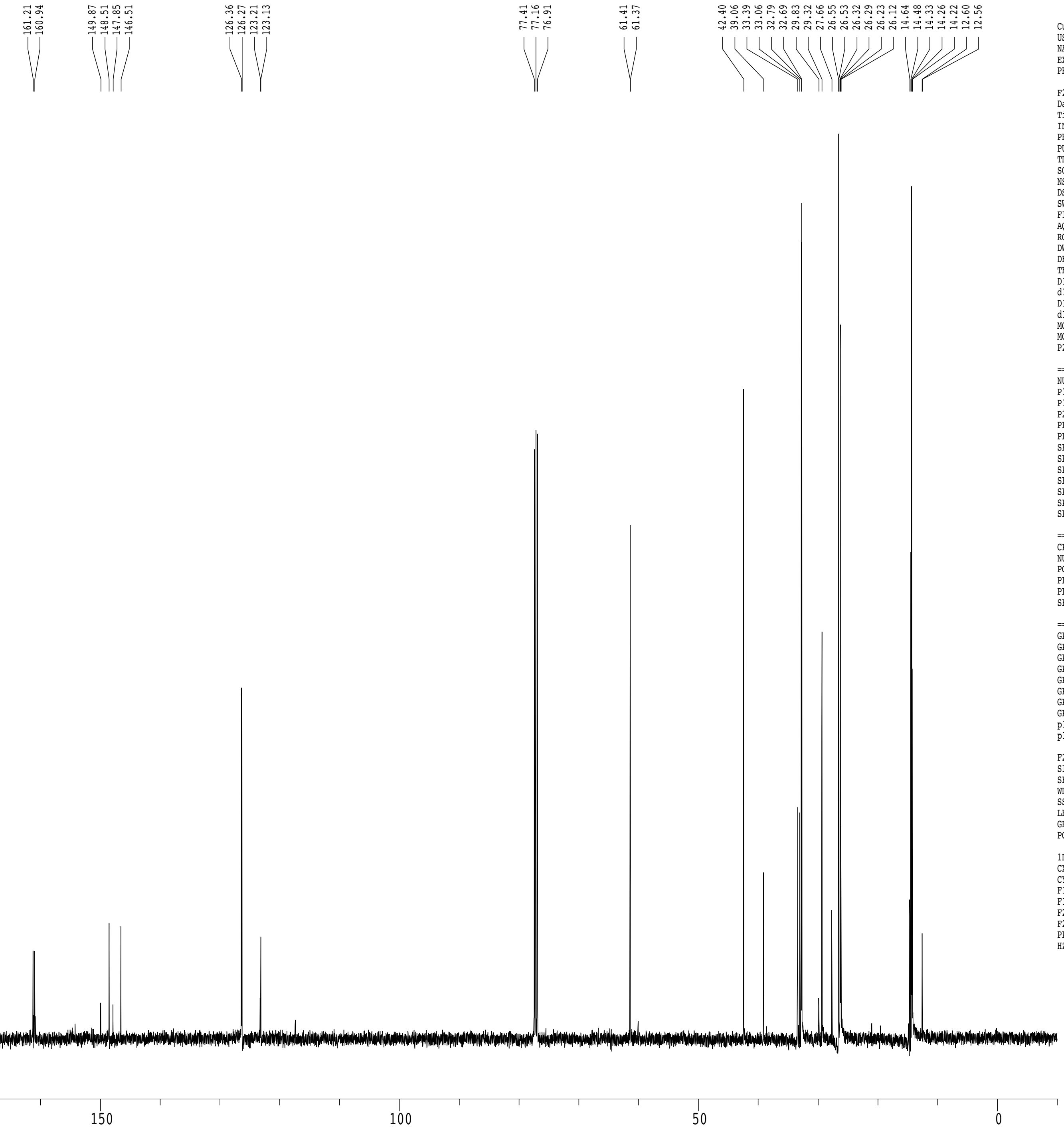


Current Data Parameters
USER linpc2
NAME pcl-2-033
EXPNO 3
PROCNO 1

F2 - Acquisition Parameters
Date_ 20210607
Time 12.22
INSTRUM cryo500
PROBHD 5 mm CPTCI 1H-
PULPROG SpinEchopg30gp2.prd
TD 65536
SOLVENT CDCl3
NS 144
DS 16
SWH 30303.031 Hz
FIDRES 0.462388 Hz
AQ 1.0813940 sec
RG 1824.6
DW 16.500 usec
DE 6.00 usec
TE 298.0 K
D1 0.25000000 sec
d11 0.03000000 sec
D16 0.00020000 sec
d17 0.00019600 sec
MCREST 0.00000000 sec
MCWRK 0.01500000 sec
P2 37.70 usec

======== CHANNEL f1 ========
NUC1 13C
P1 18.85 usec
P12 2000.00 usec
P20 500.00 usec
PL0 120.00 dB
PL1 -1.00 dB
SFO1 125.7942548 MHz
SP2 1.55 dB
SP4 1.55 dB
SPNAM2 Crp60comp.4
SPNAM4 Crp60,0.5,20.1
SPOFF2 0.00 Hz
SPOFF4 0.00 Hz

======== CHANNEL f2 ========
CPDPRG2 waltz16
NUC2 1H
PCPD2 100.00 usec
PL2 1.60 dB
PL12 22.00 dB
SFO2 500.2225011 MHz

====== GRADIENT CHANNEL =====
GPNAM1 SINE.100
GPNAM2 SINE.100
GPX1 0.00 %
GPX2 0.00 %
GPY1 0.00 %
GPY2 0.00 %
GPZ1 30.00 %
GPZ2 50.00 %
p15 500.00 usec
p16 1000.00 usec

F2 - Processing parameters
SI 65536
SF 125.7804076 MHz
WDW EM
SSB 0
LB 1.00 Hz
GB 0
PC 2.00

1D NMR plot parameters
CX 22.80 cm
CY 15.65 cm
F1P 220.000 ppm
F1 27671.69 Hz
F2P -10.000 ppm
F2 -1257.80 Hz
PPMCM 10.08772 ppm/cm
HZCM 1268.83752 Hz/cm

19F spectrum

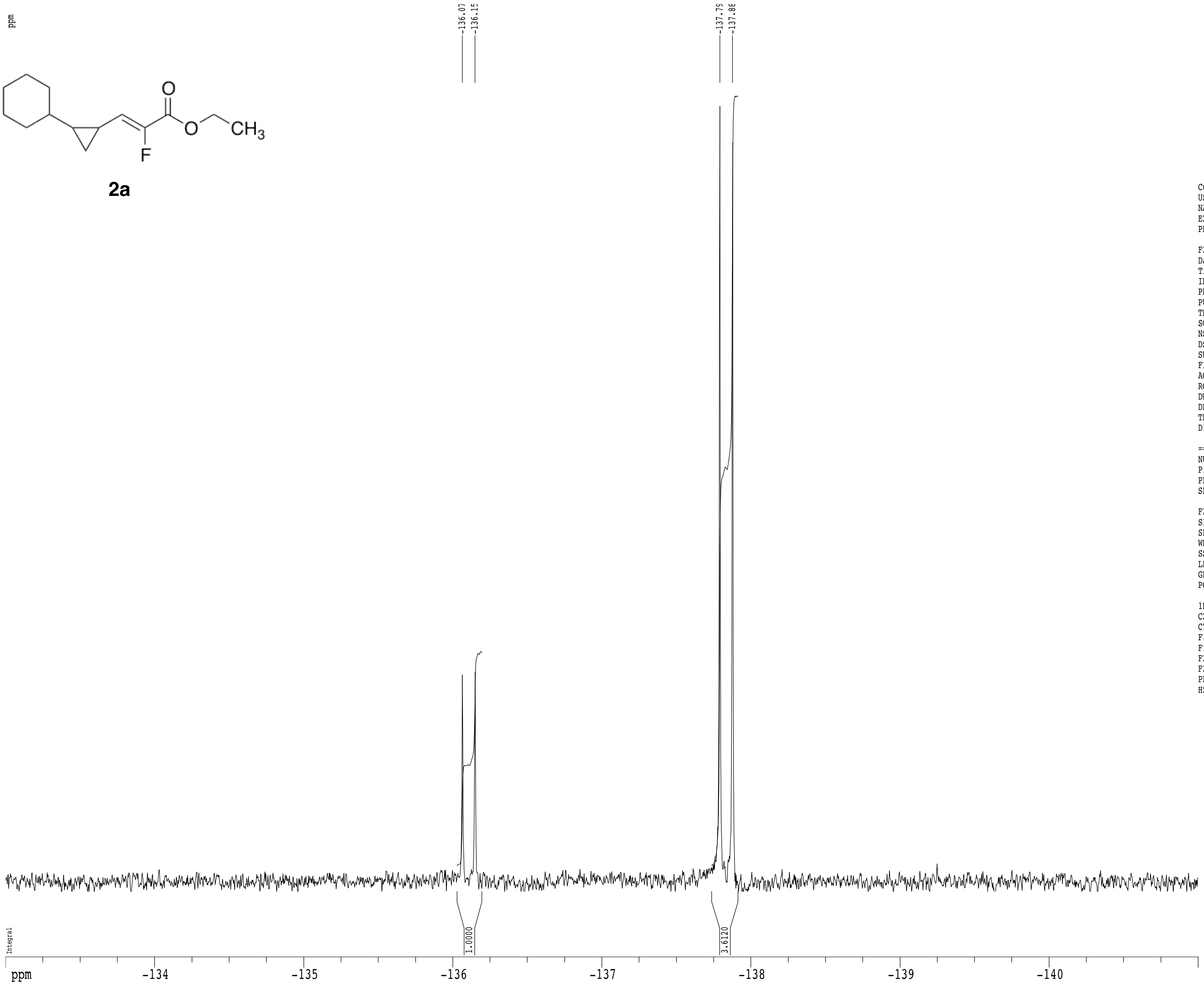


Current Data Parameters
USER linpc2
NAME pcl-2-020A
EXPNO 5
PROCNO 1

F2 - Acquisition Parameters
Date_ 20210426
Time 8.57
INSTRUM drx400
PROBHD 5 mm QNP H/F/P
PULPROG zgflqn30
TD 65536
SOLVENT CDCl3
NS 32
DS 2
SWH 75187.969 Hz
FIDRES 1.147277 Hz
AQ 0.4358644 sec
RG 6502
DW 6.650 usec
DE 9.46 usec
TE 297.9 K
D1 2.00000000 sec

======== CHANNEL f1 ========
NUC1 19F
P1 11.75 usec
PL1 -6.00 dB
SFO1 376.4646491 MHz

F2 - Processing parameters
SI 65536
SF 376.4984640 MHz
WDW EM
SSB 0
LB 1.00 Hz
GB 0
PC 1.00

1D NMR plot parameters
CX 22.80 cm
CY 15.00 cm
F1P -133.000 ppm
F1 -50074.30 Hz
F2P -141.000 ppm
F2 -53086.29 Hz
PPMCM 0.35088 ppm/cm
HZCM 132.10474 Hz/cm

1H spectrum

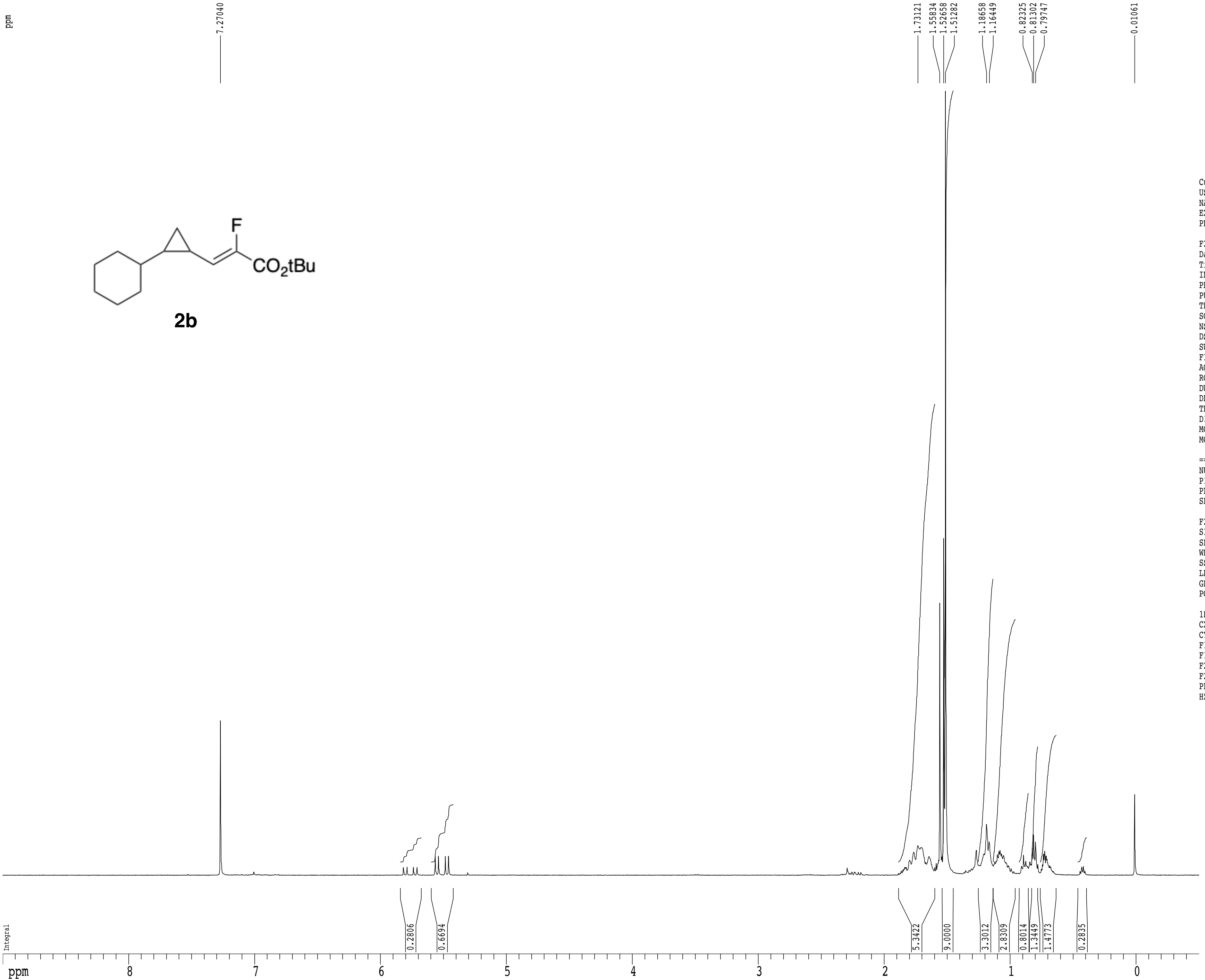


```
Current Data Parameters
USER          mcginnit
NAME          tmm-3-178
EXPNO                2
PROCNO               1

F2 - Acquisition Parameters
Date_         20210714
Time             18.11
INSTRUM         drx400
PROBHD   5 mm QNP H/F/P
PULPROG           zg30
TD               65536
SOLVENT          CDCl3
NS                   8
DS                   2
SWH           6410.256 Hz
FIDRES        0.097813 Hz
AQ           5.1118579 sec
RG               456.1
DW              78.000 usec
DE                4.50 usec
TE               298.0 K
D1          0.10000000 sec
MCREST      0.00000000 sec
MCWRK       0.01500000 sec

======== CHANNEL f1 ========
NUC1                1H
P1               12.00 usec
PL1              -1.60 dB
SFO1       400.1328009 MHz

F2 - Processing parameters
SI               65536
SF         400.1300175 MHz
WDW                 EM
SSB                  0
LB                0.30 Hz
GB                   0
PC                2.00

1D NMR plot parameters
CX               22.80 cm
CY               15.00 cm
F1P              9.000 ppm
F1             3601.17 Hz
F2P             -0.500 ppm
F2             -200.06 Hz
PPMCM          0.41667 ppm/cm
HZCM         166.72084 Hz/cm
```

13C spectrum with 1H decoupling

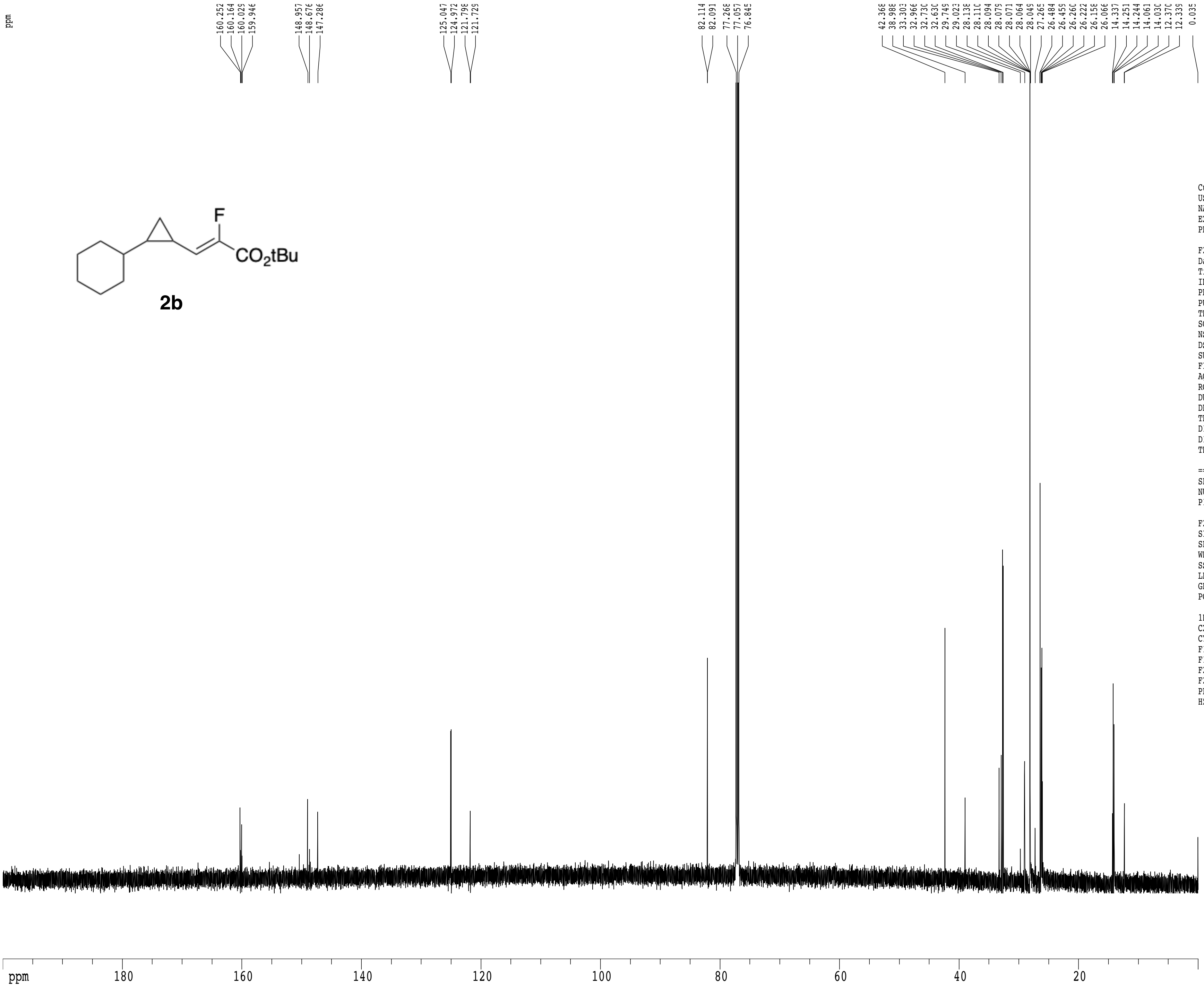


**2b**

Current Data Parameters
USER mcginnit
NAME tmm-3-178
EXPNO 6
PROCNO 1

F2 - Acquisition Parameters
Date_ 20210716
Time 14.40
INSTRUM av600
PROBHD 5 mm CPBBO BB-
PULPROG zgdc30
TD 65536
SOLVENT CDCl3
NS 344
DS 4
SWH 36231.883 Hz
FIDRES 0.552855 Hz
AQ 0.9044468 sec
RG 2050
DW 13.800 usec
DE 19.63 usec
TE 298.0 K
D1 0.40000001 sec
D11 0.03000000 sec
TD0 1

======== CHANNEL f1 ========
SFO1 150.9194080 MHz
NUC1 13C
P1 10.10 usec

F2 - Processing parameters
SI 65536
SF 150.9028085 MHz
WDW no
SSB 0
LB 0.00 Hz
GB 0
PC 1.00

1D NMR plot parameters
CX 22.80 cm
CY 75.00 cm
F1P 200.000 ppm
F1 30180.56 Hz
F2P 0.000 ppm
F2 0.00 Hz
PPMCM 8.77193 ppm/cm
HZCM 1323.70886 Hz/cm

19F spectrum

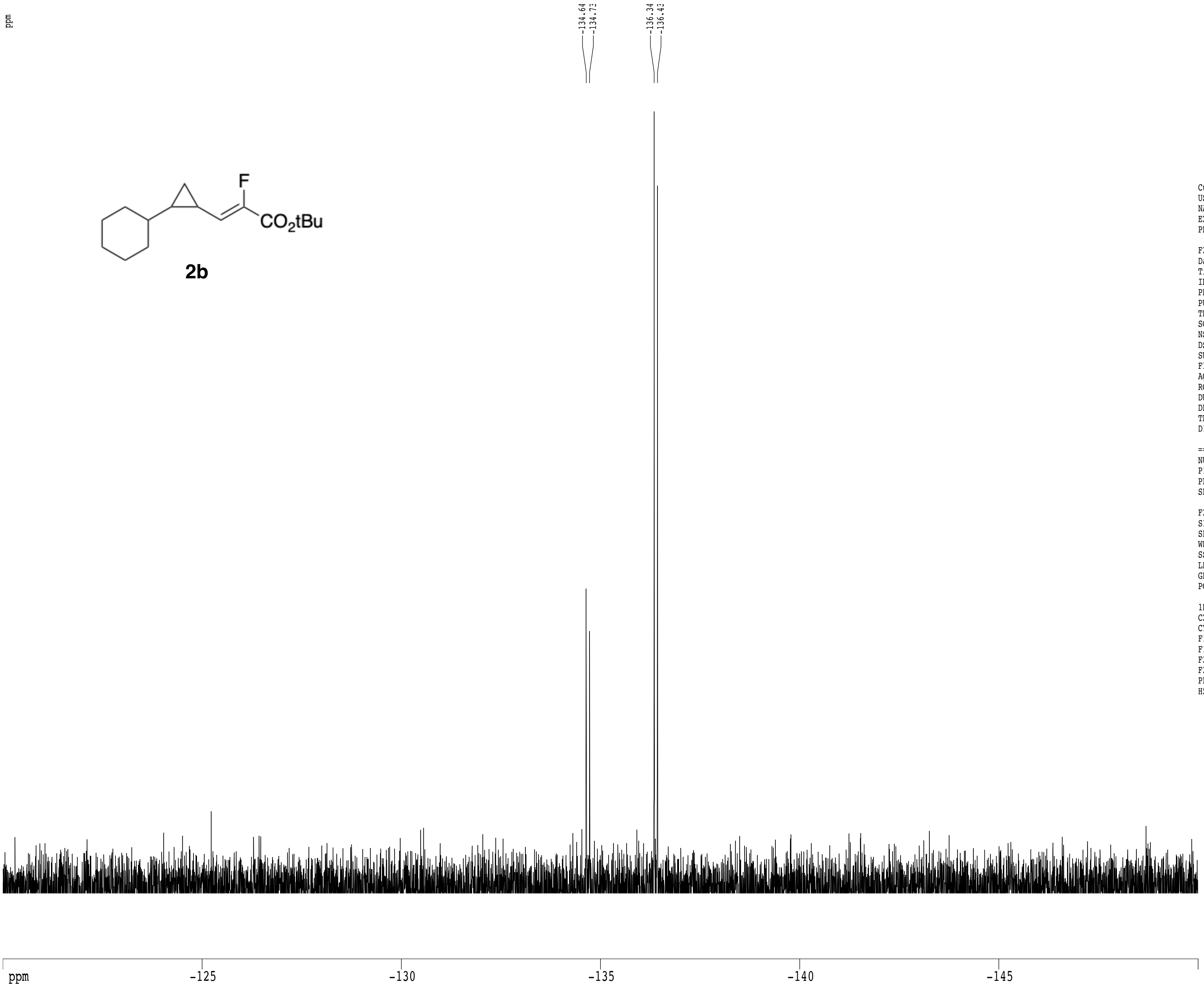


Current Data Parameters
USER mcginnit
NAME tmm-3-178
EXPNO 8
PROCNO 1

F2 - Acquisition Parameters
Date_ 20210716
Time 15.43
INSTRUM drx400
PROBHD 5 mm QNP H/F/P
PULPROG zgflqn30
TD 65536
SOLVENT CDCl3
NS 78
DS 2
SWH 75187.969 Hz
FIDRES 1.147277 Hz
AQ 0.4358644 sec
RG 6502
DW 6.650 usec
DE 9.46 usec
TE 298.0 K
D1 2.00000000 sec

======== CHANNEL f1 ========
NUC1 19F
P1 11.75 usec
PL1 -6.00 dB
SFO1 376.4646491 MHz

F2 - Processing parameters
SI 65536
SF 376.4984640 MHz
WDW no
SSB 0
LB 0.00 Hz
GB 0
PC 1.00

1D NMR plot parameters
CX 22.80 cm
CY 15.00 cm
F1P -120.000 ppm
F1 -45179.82 Hz
F2P -150.000 ppm
F2 -56474.77 Hz
PPMCM 1.31579 ppm/cm
HZCM 495.39273 Hz/cm

1H spectrum

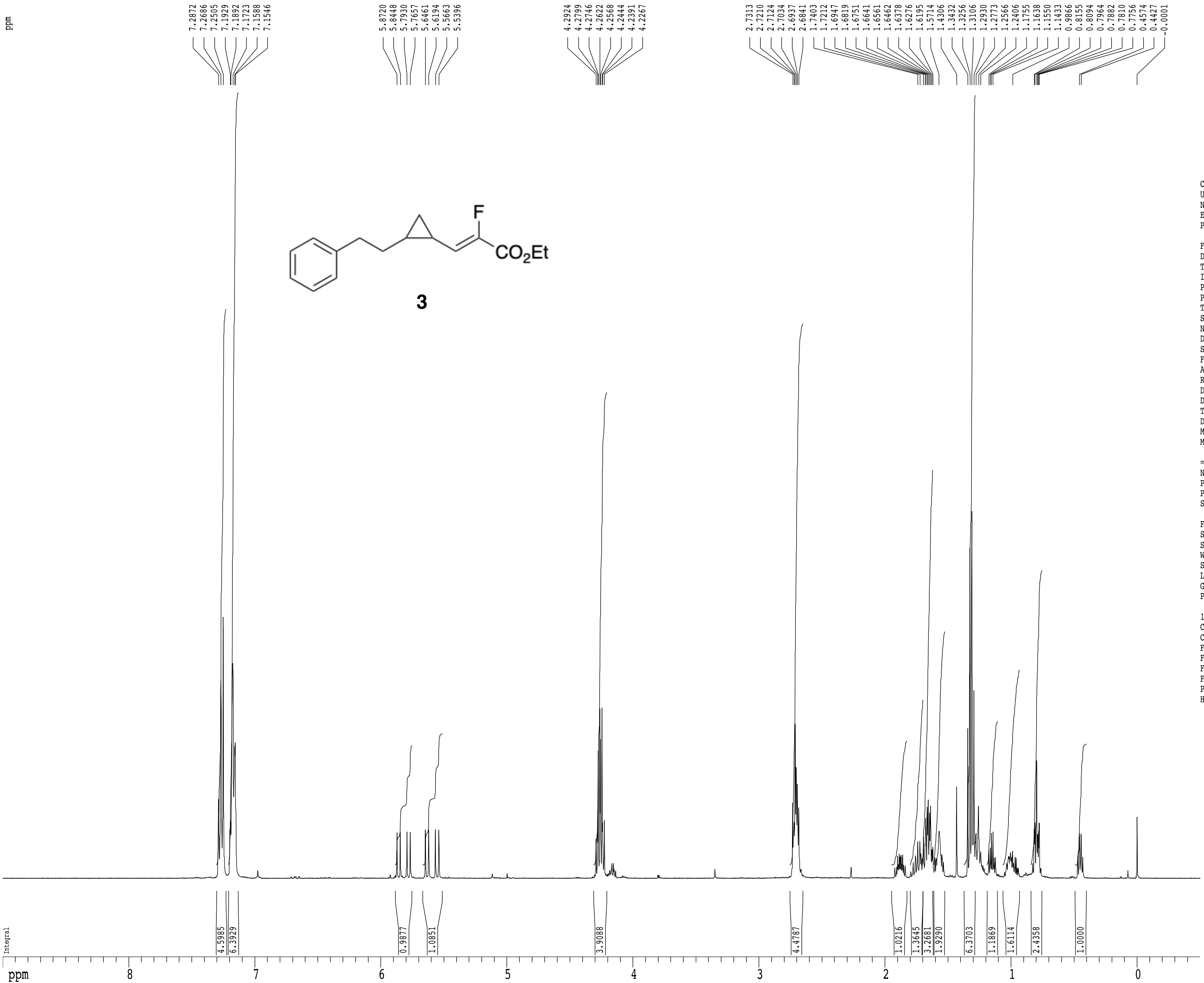


**3**

Current Data Parameters
USER sanforda
NAME ABS-3-280-nih
EXPNO 1
PROCNO 1

F2 - Acquisition Parameters
Date_ 20201130
Time 15.45
INSTRUM drx400
PROBHD 5 mm QNP H/F/P
PULPROG zg30
TD 38460
SOLVENT CDCl3
NS 8
DS 2
SWH 6410.256 Hz
FIDRES 0.166673 Hz
AQ 2.9999299 sec
RG 161.3
DW 78.000 usec
DE 4.50 usec
TE 298.0 K
D1 0.10000000 sec
MCREST 0.00000000 sec
MCWRK 0.01500000 sec

======== CHANNEL f1 ========
NUC1 1H
P1 12.00 usec
PL1 -1.60 dB
SFO1 400.1328009 MHz

F2 - Processing parameters
SI 65536
SF 400.1300251 MHz
WDW no
SSB 0
LB 0.00 Hz
GB 0
PC 2.00

1D NMR plot parameters
CX 22.80 cm
CY 7.00 cm
F1P 9.000 ppm
F1 3601.17 Hz
F2P -0.500 ppm
F2 -200.06 Hz
PPMCM 0.41667 ppm/cm
HZCM 166.72086 Hz/cm

Z-restored spin-echo 13C spectrum with 1H decoupling

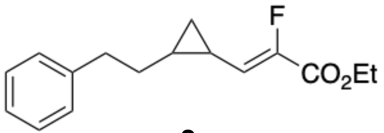


**3**

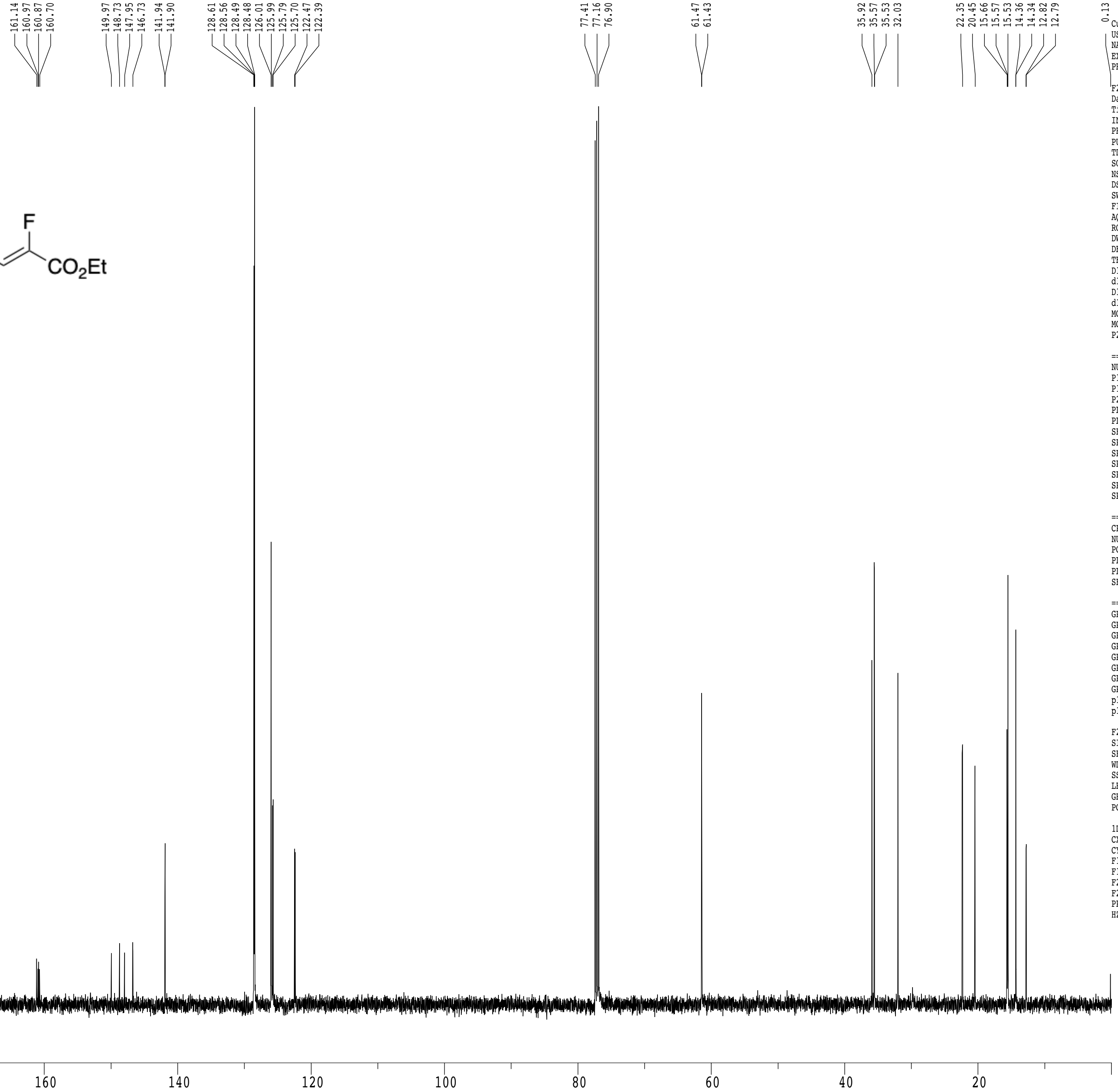


Current Data Parameters
USER sanforda
NAME ABS-4-026-carbon
EXPNO 1
PROCNO 1

F2 - Acquisition Parameters
Date_ 20201222
Time 15.06
INSTRUM cryo500
PROBHD 5 mm CPTCI 1H-
PULPROG SpinEchopg30gp2.prd
TD 65536
SOLVENT CDCl3
NS 296
DS 16
SWH 30303.031 Hz
FIDRES 0.462388 Hz
AQ 1.0813940 sec
RG 7298.2
DW 16.500 usec
DE 6.00 usec
TE 298.0 K
D1 0.25000000 sec
d11 0.03000000 sec
D16 0.00020000 sec
d17 0.00019600 sec
MCREST 0.00000000 sec
MCWRK 0.01500000 sec
P2 37.70 usec

======== CHANNEL f1 ========
NUC1 13C
P1 18.85 usec
P12 2000.00 usec
P20 500.00 usec
PL0 120.00 dB
PL1 -1.00 dB
SFO1 125.7942548 MHz
SP2 1.55 dB
SP4 1.55 dB
SPNAM2 Crp60comp.4
SPNAM4 Crp60,0.5,20.1
SPOFF2 0.00 Hz
SPOFF4 0.00 Hz

======== CHANNEL f2 ========
CPDPRG2 waltz16
NUC2 1H
PCPD2 100.00 usec
PL2 1.60 dB
PL12 22.00 dB
SFO2 500.2225011 MHz

====== GRADIENT CHANNEL =====
GPNAM1 SINE.100
GPNAM2 SINE.100
GPX1 0.00 %
GPX2 0.00 %
GPY1 0.00 %
GPY2 0.00 %
GPZ1 30.00 %
GPZ2 50.00 %
p15 500.00 usec
p16 1000.00 usec

F2 - Processing parameters
SI 65536
SF 125.7804080 MHz
WDW EM
SSB 0
LB 1.00 Hz
GB 0
PC 2.00

1D NMR plot parameters
CX 22.80 cm
CY 15.65 cm
F1P 200.000 ppm
F1 25156.08 Hz
F2P 0.000 ppm
F2 0.00 Hz
PPMCM 8.77193 ppm/cm
HZCM 1103.33691 Hz/cm

19F spectrum

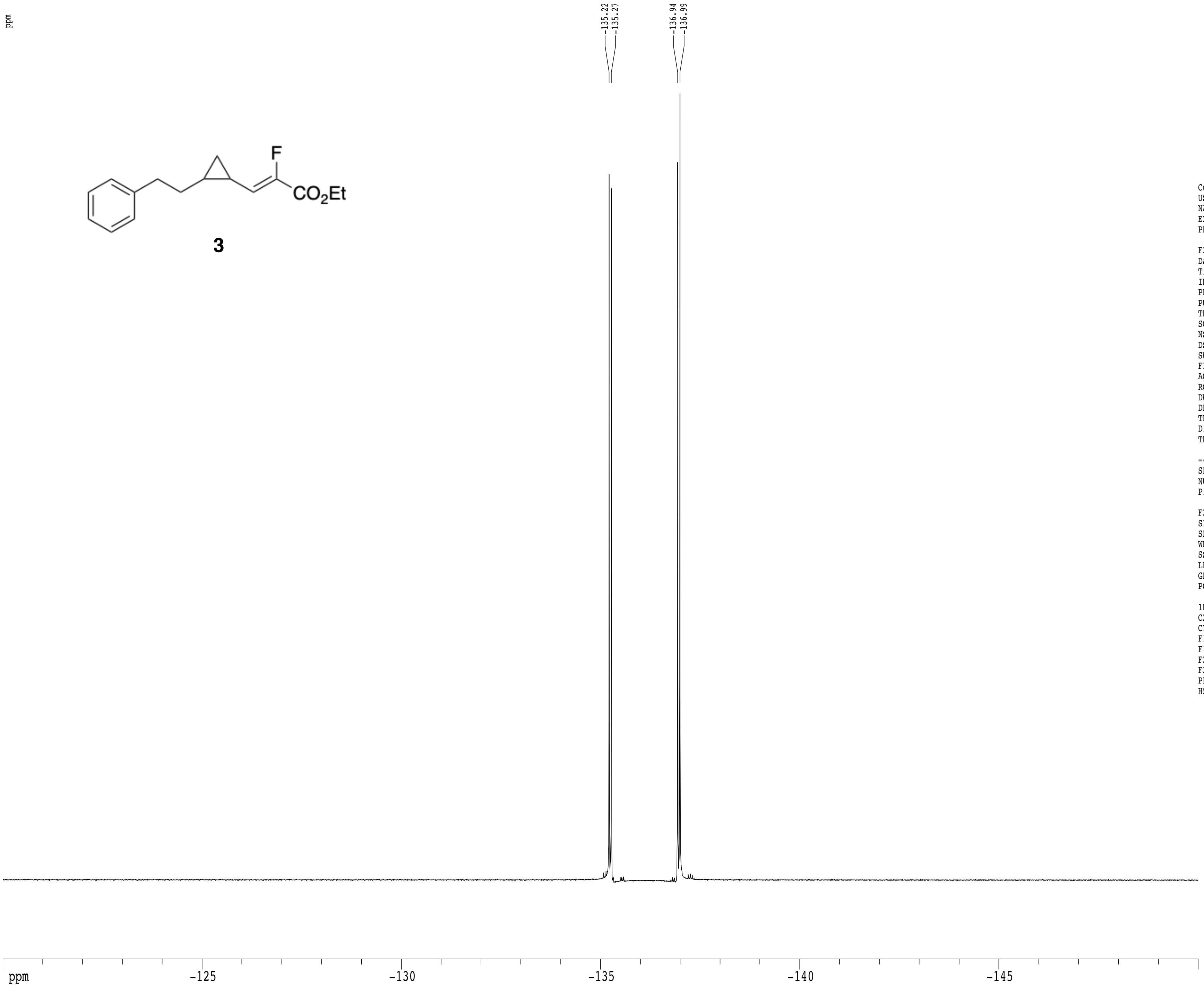


Current Data Parameters
USER sanforda
NAME ABS-4-056-f
EXPNO 1
PROCNO 1

F2 - Acquisition Parameters
Date_ 20210212
Time 12.47
INSTRUM av600
PROBHD 5 mm CPBBO BB-
PULPROG zgflqn30
TD 131072
SOLVENT CDCl3
NS 16
DS 2
SWH 178571.422 Hz
FIDRES 1.362392 Hz
AQ 0.3670516 sec
RG 575
DW 2.800 usec
DE 18.00 usec
TE 298.1 K
D1 3.00000000 sec
TD0 1

======== CHANNEL f1 ========
SFO1 564.6299196 MHz
NUC1 19F
P1 18.25 usec

F2 - Processing parameters
SI 131072
SF 564.6863858 MHz
WDW no
SSB 0
LB 0.00 Hz
GB 0
PC 1.00

1D NMR plot parameters
CX 22.80 cm
CY 15.00 cm
F1P -120.000 ppm
F1 -67762.37 Hz
F2P -150.000 ppm
F2 -84702.96 Hz
PPMCM 1.31579 ppm/cm
HZCM 743.00842 Hz/cm

**SFC Chiracel OD-H, 1% IPA/$CO_2$, 2.0 mL/min**

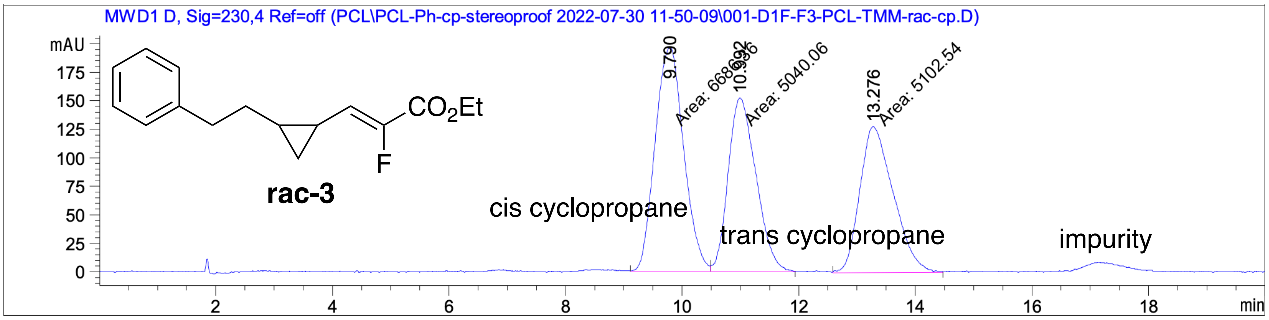


```
Signal 4: MWD1 D, Sig=230,4 Ref=off

Peak RetTime Type  Width     Area      Height     Area
  #   [min]        [min]   [mAU*s]     [mAU]        %
----|-------|----|-------|----------|----------|--------|
   1   9.790 MF    0.5661 6686.36084  196.84850  39.7313
   2  10.992 FM    0.5520 5040.06494  152.16756  29.9488
   3  13.276 MM    0.6641 5102.54004  128.05997  30.3200

Totals :                  1.68290e4   477.07602
```

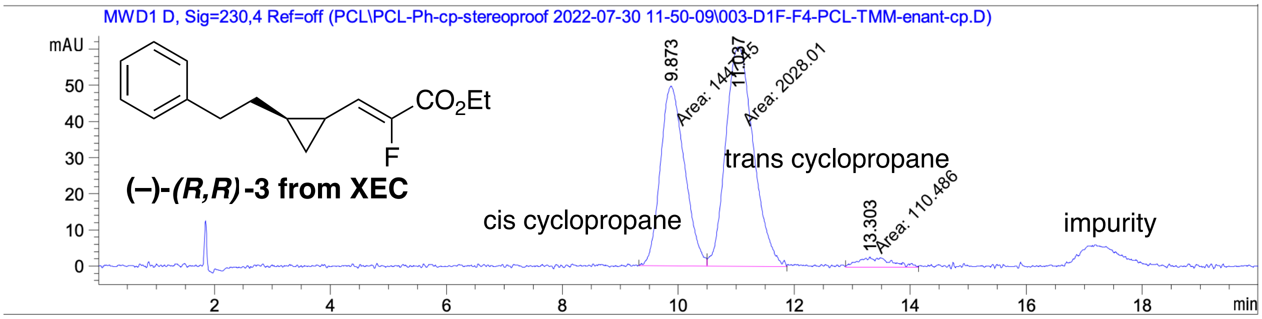


```
Signal 4: MWD1 D, Sig=230,4 Ref=off

Peak RetTime Type  Width     Area      Height     Area
  #   [min]        [min]   [mAU*s]     [mAU]        %
----|-------|----|-------|----------|----------|--------|
   1   9.873 MF    0.4859 1447.44983   49.65176  40.3645
   2  11.037 FM    0.5572 2028.01355   60.65751  56.5544
   3  13.303 MM    0.6396  110.48612    2.87905   3.0811

Totals :                  3585.94950  113.18832
```

1H spectrum

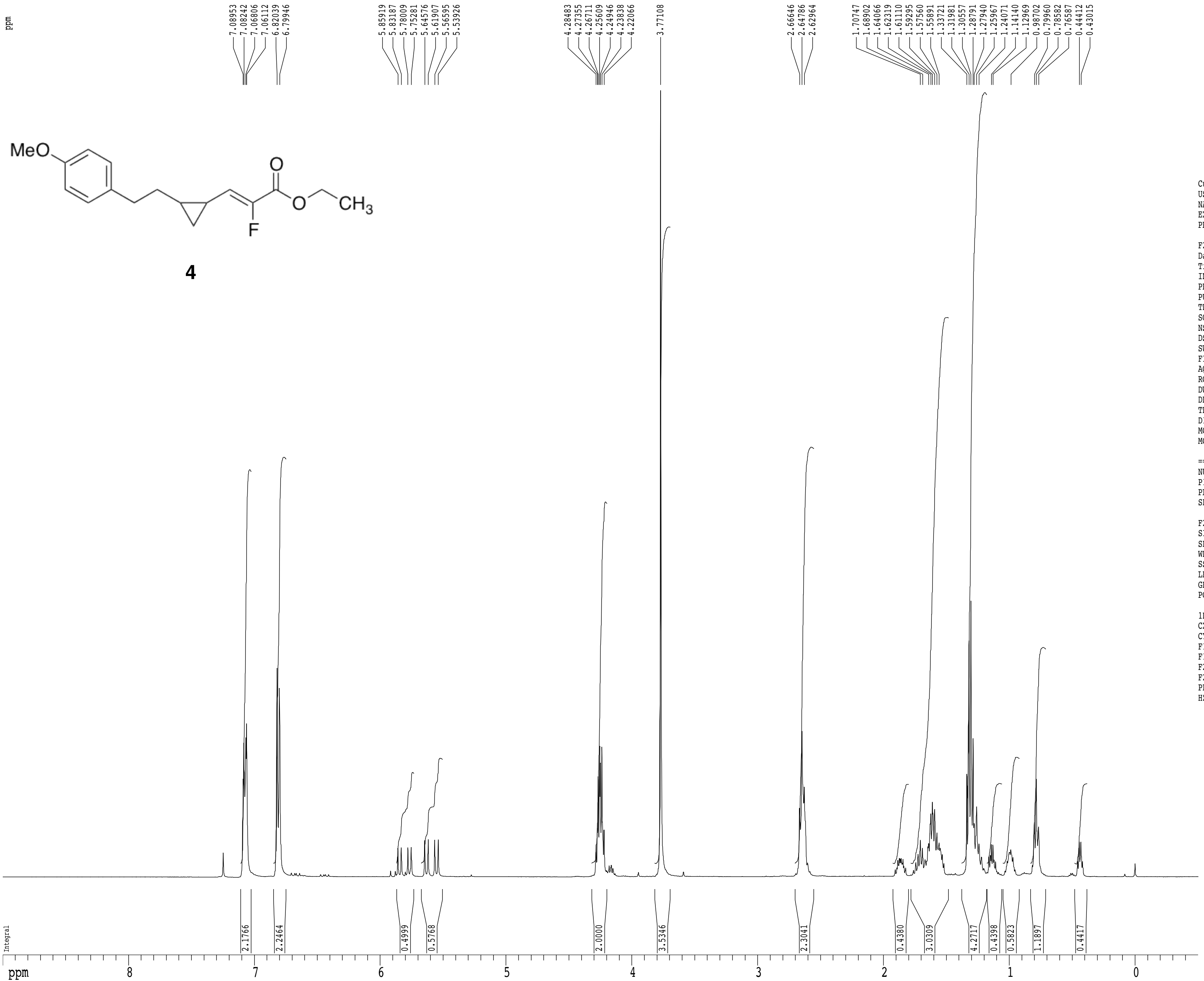


4

```
Current Data Parameters
USER              linpc2
NAME           pcl-2-066
EXPNO                  3
PROCNO                 1

F2 - Acquisition Parameters
Date_           20210610
Time               14.06
INSTRUM           drx400
PROBHD   5 mm QNP H/F/P
PULPROG             zg30
TD                 65536
SOLVENT            CDCl3
NS                     8
DS                     2
SWH           6410.256 Hz
FIDRES        0.097813 Hz
AQ           5.1118579 sec
RG                  50.8
DW              78.000 usec
DE                4.50 usec
TE               298.0 K
D1          0.10000000 sec
MCREST      0.00000000 sec
MCWRK       0.01500000 sec

======== CHANNEL f1 ========
NUC1                  1H
P1               12.00 usec
PL1              -1.60 dB
SFO1        400.1328009 MHz

F2 - Processing parameters
SI                 65536
SF          400.1300264 MHz
WDW                   EM
SSB                    0
LB                  0.30 Hz
GB                     0
PC                  2.00

1D NMR plot parameters
CX                 22.80 cm
CY                 15.00 cm
F1P                9.000 ppm
F1               3601.17 Hz
F2P               -0.500 ppm
F2               -200.06 Hz
PPMCM            0.41667 ppm/cm
HZCM           166.72086 Hz/cm
```

13C spectrum with 1H decoupling

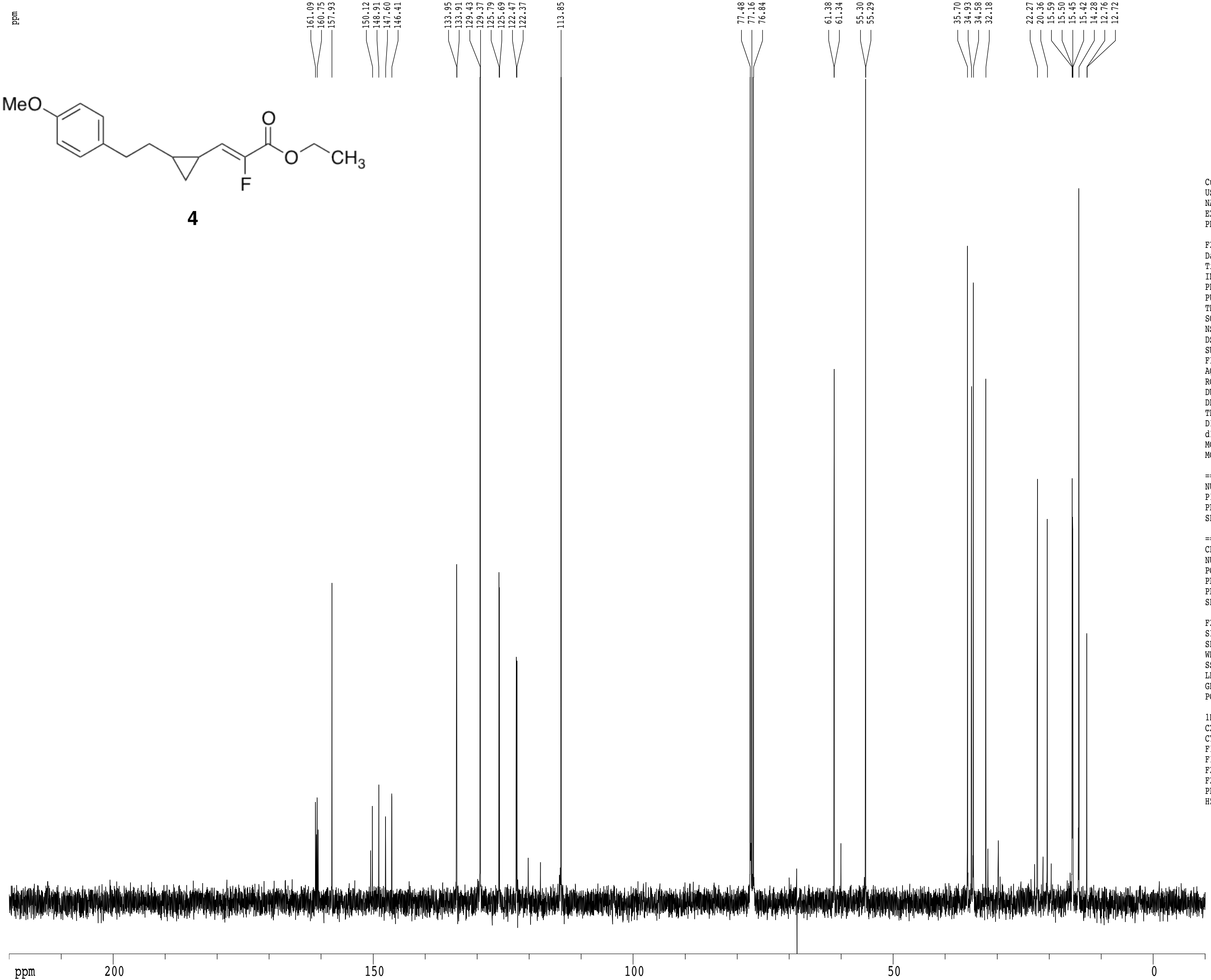

4


Current Data Parameters
USER linpc2
NAME pcl-2-066
EXPNO 4
PROCNO 1

F2 - Acquisition Parameters
Date_ 20210610
Time 14.08
INSTRUM drx400
PROBHD 5 mm QNP H/F/P
PULPROG zgdc30
TD 65536
SOLVENT CDCl3
NS 224
DS 4
SWH 24154.590 Hz
FIDRES 0.368570 Hz
AQ 1.3566452 sec
RG 14596.5
DW 20.700 usec
DE 20.39 usec
TE 298.0 K
D1 0.10000000 sec
d11 0.03000000 sec
MCREST 0.00000000 sec
MCWRK 0.01500000 sec

======== CHANNEL f1 ========
NUC1 13C
P1 8.30 usec
PL1 -3.00 dB
SFO1 100.6237964 MHz

======== CHANNEL f2 ========
CPDPRG2 waltz16
NUC2 1H
PCPD2 90.00 usec
PL2 -1.60 dB
PL12 16.50 dB
SFO2 400.1328009 MHz

F2 - Processing parameters
SI 65536
SF 100.6127650 MHz
WDW EM
SSB 0
LB 1.00 Hz
GB 0
PC 1.00

1D NMR plot parameters
CX 22.80 cm
CY 45.00 cm
F1P 220.000 ppm
F1 22134.81 Hz
F2P -10.000 ppm
F2 -1006.13 Hz
PPMCM 10.08772 ppm/cm
HZCM 1014.95337 Hz/cm

19F spectrum

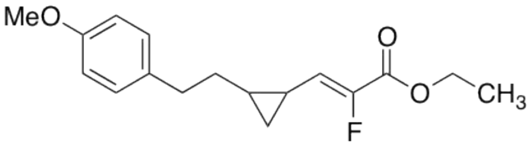

4

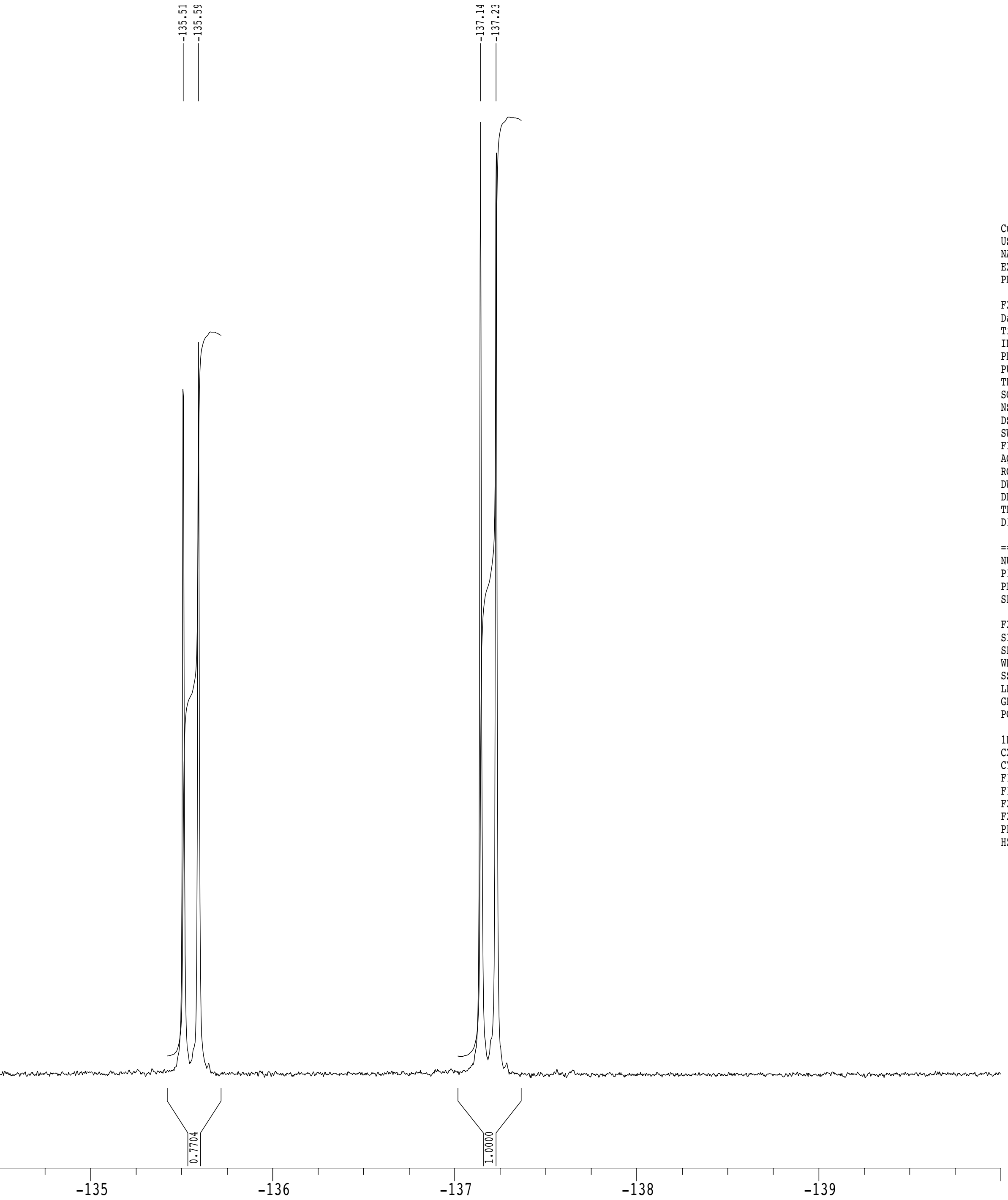


Current Data Parameters
USER linpc2
NAME pcl-2-066
EXPNO 5
PROCNO 1

F2 - Acquisition Parameters
Date_ 20210610
Time 14.16
INSTRUM drx400
PROBHD 5 mm QNP H/F/P
PULPROG zgflqn30
TD 65536
SOLVENT CDCl3
NS 64
DS 2
SWH 75187.969 Hz
FIDRES 1.147277 Hz
AQ 0.4358644 sec
RG 8192
DW 6.650 usec
DE 9.46 usec
TE 298.0 K
D1 2.00000000 sec

======== CHANNEL f1 ========
NUC1 19F
P1 11.75 usec
PL1 -6.00 dB
SFO1 376.4646491 MHz

F2 - Processing parameters
SI 65536
SF 376.4984640 MHz
WDW EM
SSB 0
LB 1.00 Hz
GB 0
PC 1.00

1D NMR plot parameters
CX 22.80 cm
CY 15.00 cm
F1P -132.000 ppm
F1 -49697.80 Hz
F2P -140.000 ppm
F2 -52709.79 Hz
PPMCM 0.35088 ppm/cm
HZCM 132.10474 Hz/cm

**SFC Chiracel OD-H, 0.1% IPA/$CO_2$, 2.0 mL/min**

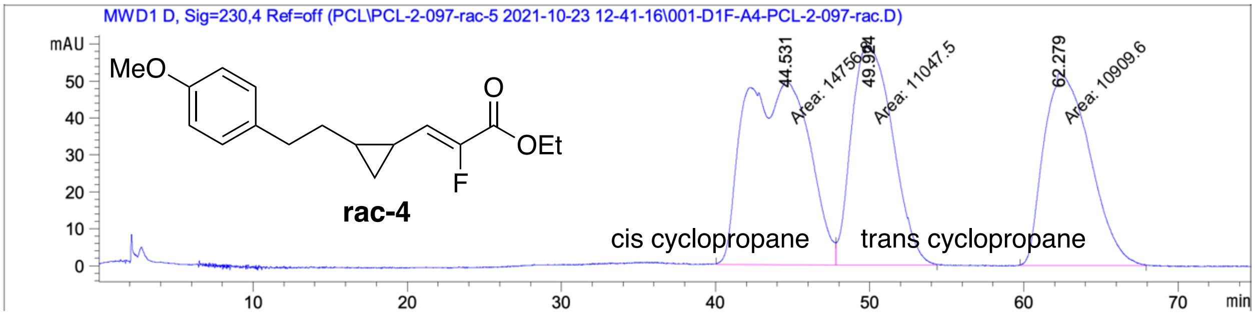


Signal 4: MWD1 D, Sig=230,4 Ref=off

| Peak # | RetTime [min] | Type | Width [min] | Area [mAU*s] | Height [mAU] | Area % |
|---|---|---|---|---|---|---|
| 1 | 44.531 | MF | 4.9450 | 1.47568e4 | 49.73637 | 40.1940 |
| 2 | 49.924 | FM | 3.1080 | 1.10475e4 | 59.24161 | 30.0907 |
| 3 | 62.279 | MM | 3.4941 | 1.09096e4 | 52.03885 | 29.7153 |

Totals : 3.67138e4 161.01682

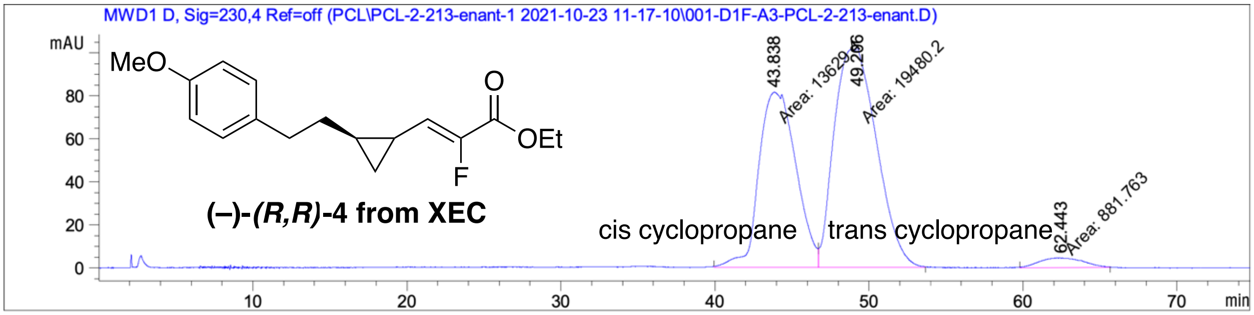


Signal 4: MWD1 D, Sig=230,4 Ref=off

| Peak # | RetTime [min] | Type | Width [min] | Area [mAU*s] | Height [mAU] | Area % |
|---|---|---|---|---|---|---|
| 1 | 43.838 | MF | 2.7921 | 1.36294e4 | 81.35760 | 40.0966 |
| 2 | 49.206 | FM | 3.1460 | 1.94802e4 | 103.20026 | 57.3093 |
| 3 | 62.443 | MM | 3.3020 | 881.76331 | 4.45065 | 2.5941 |

Totals : 3.39913e4 189.00851

1H spectrum

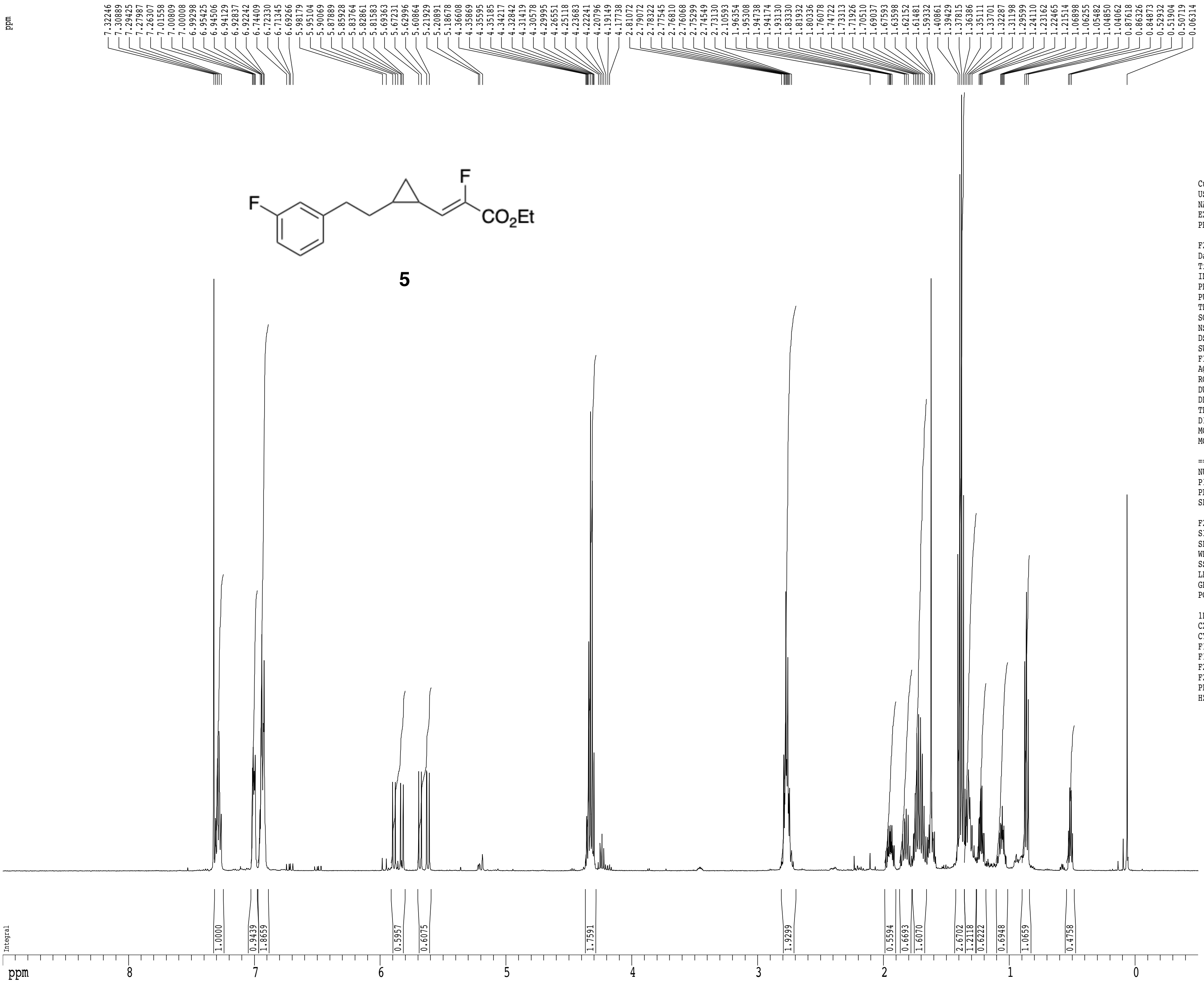


```
Current Data Parameters
USER            mcginnit
NAME     tmm-3-105-char
EXPNO                  1
PROCNO                 1

F2 - Acquisition Parameters
Date_           20210514
Time               13.24
INSTRUM          cryo500
PROBHD   5 mm CPTCI 1H-
PULPROG             zg30
TD                 81728
SOLVENT            CDCl3
NS                     8
DS                     2
SWH             8012.820 Hz
FIDRES          0.098043 Hz
AQ             5.0998774 sec
RG                   4.5
DW                62.400 usec
DE                  6.00 usec
TE                 298.0 K
D1            0.10000000 sec
MCREST        0.00000000 sec
MCWRK         0.01500000 sec

======== CHANNEL f1 ========
NUC1                  1H
P1                  9.75 usec
PL1                 1.60 dB
SFO1         500.2235015 MHz

F2 - Processing parameters
SI                 65536
SF           500.2200000 MHz
WDW                   EM
SSB                    0
LB                  0.30 Hz
GB                     0
PC                  1.00

1D NMR plot parameters
CX                 22.80 cm
CY                 15.00 cm
F1P                9.000 ppm
F1               4501.98 Hz
F2P               -0.500 ppm
F2               -250.11 Hz
PPMCM            0.41667 ppm/cm
HZCM           208.42500 Hz/cm
```

Z-restored spin-echo 13C spectrum with 1H decoupling

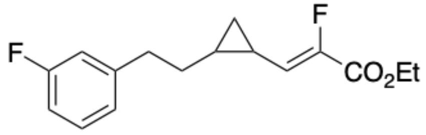

5

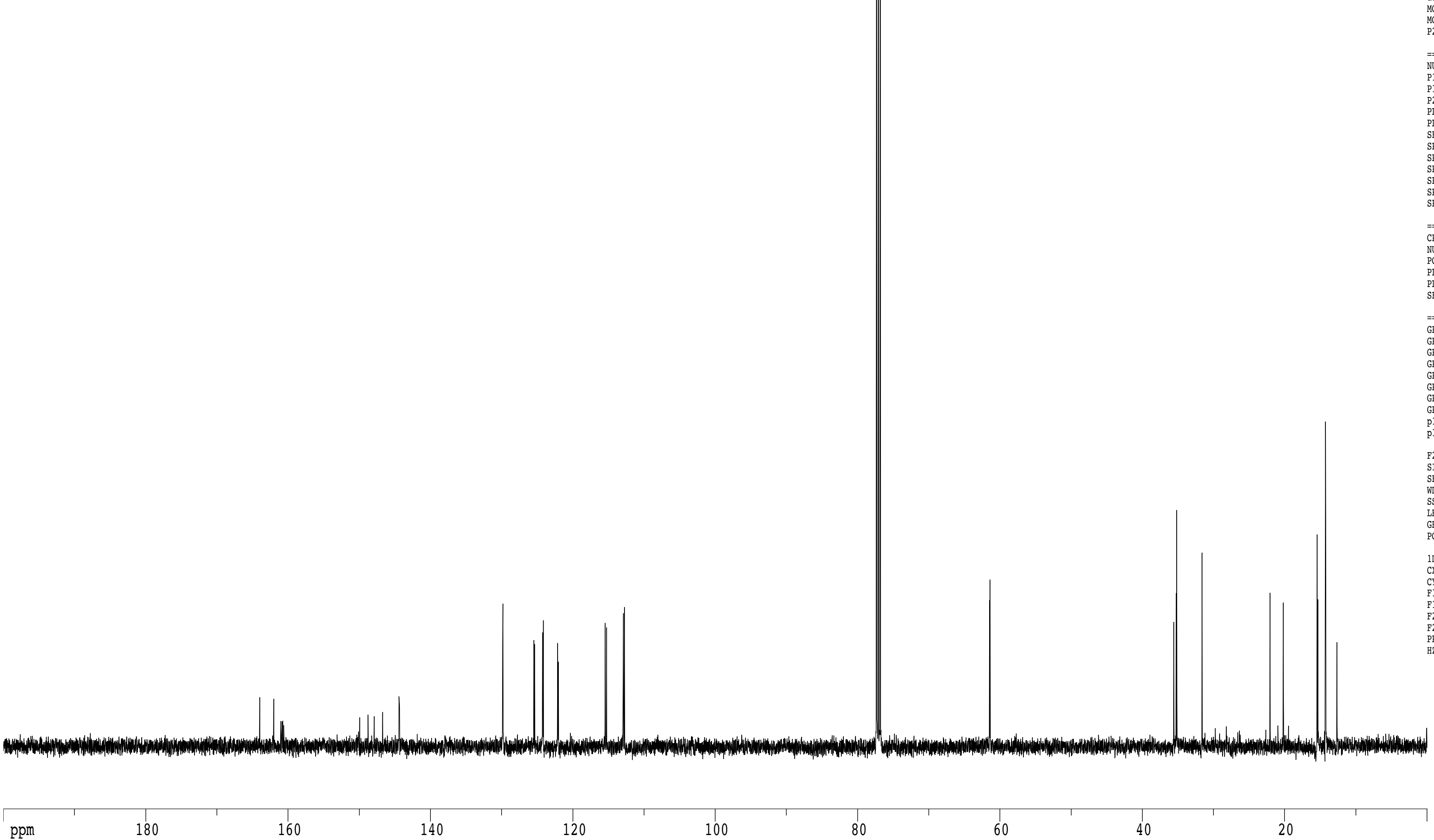


```
Current Data Parameters
USER          mcginnit
NAME     tmm-3-105-char
EXPNO                2
PROCNO               1

F2 - Acquisition Parameters
Date_         20210514
Time             13.27
INSTRUM        cryo500
PROBHD   5 mm CPTCI 1H-
PULPROG  SpinEchopg30gp2.prd
TD               65536
SOLVENT          CDCl3
NS                 331
DS                  16
SWH          30303.031 Hz
FIDRES        0.462388 Hz
AQ           1.0813940 sec
RG                6502
DW              16.500 usec
DE                6.00 usec
TE               298.0 K
D1          0.25000000 sec
d11         0.03000000 sec
D16         0.00020000 sec
d17         0.00019600 sec
MCREST      0.00000000 sec
MCWRK       0.01500000 sec
P2               37.70 usec

======== CHANNEL f1 ========
NUC1               13C
P1               18.85 usec
P12            2000.00 usec
P20             500.00 usec
PL0             120.00 dB
PL1              -1.00 dB
SFO1       125.7942548 MHz
SP2               1.55 dB
SP4               1.55 dB
SPNAM2     Crp60comp.4
SPNAM4   Crp60,0.5,20.1
SPOFF2            0.00 Hz
SPOFF4            0.00 Hz

======== CHANNEL f2 ========
CPDPRG2        waltz16
NUC2                1H
PCPD2           100.00 usec
PL2               1.60 dB
PL12             22.00 dB
SFO2       500.2225011 MHz

====== GRADIENT CHANNEL =====
GPNAM1        SINE.100
GPNAM2        SINE.100
GPX1              0.00 %
GPX2              0.00 %
GPY1              0.00 %
GPY2              0.00 %
GPZ1             30.00 %
GPZ2             50.00 %
p15             500.00 usec
p16            1000.00 usec

F2 - Processing parameters
SI               65536
SF         125.7804190 MHz
WDW                 EM
SSB                  0
LB                1.00 Hz
GB                   0
PC                2.00

1D NMR plot parameters
CX               22.80 cm
CY               15.65 cm
F1P            200.000 ppm
F1            25156.08 Hz
F2P              0.000 ppm
F2                0.00 Hz
PPMCM          8.77193 ppm/cm
HZCM        1103.33704 Hz/cm
```

19F spectrum

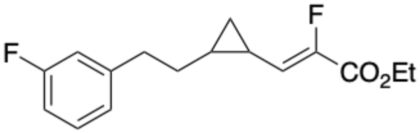

**5**

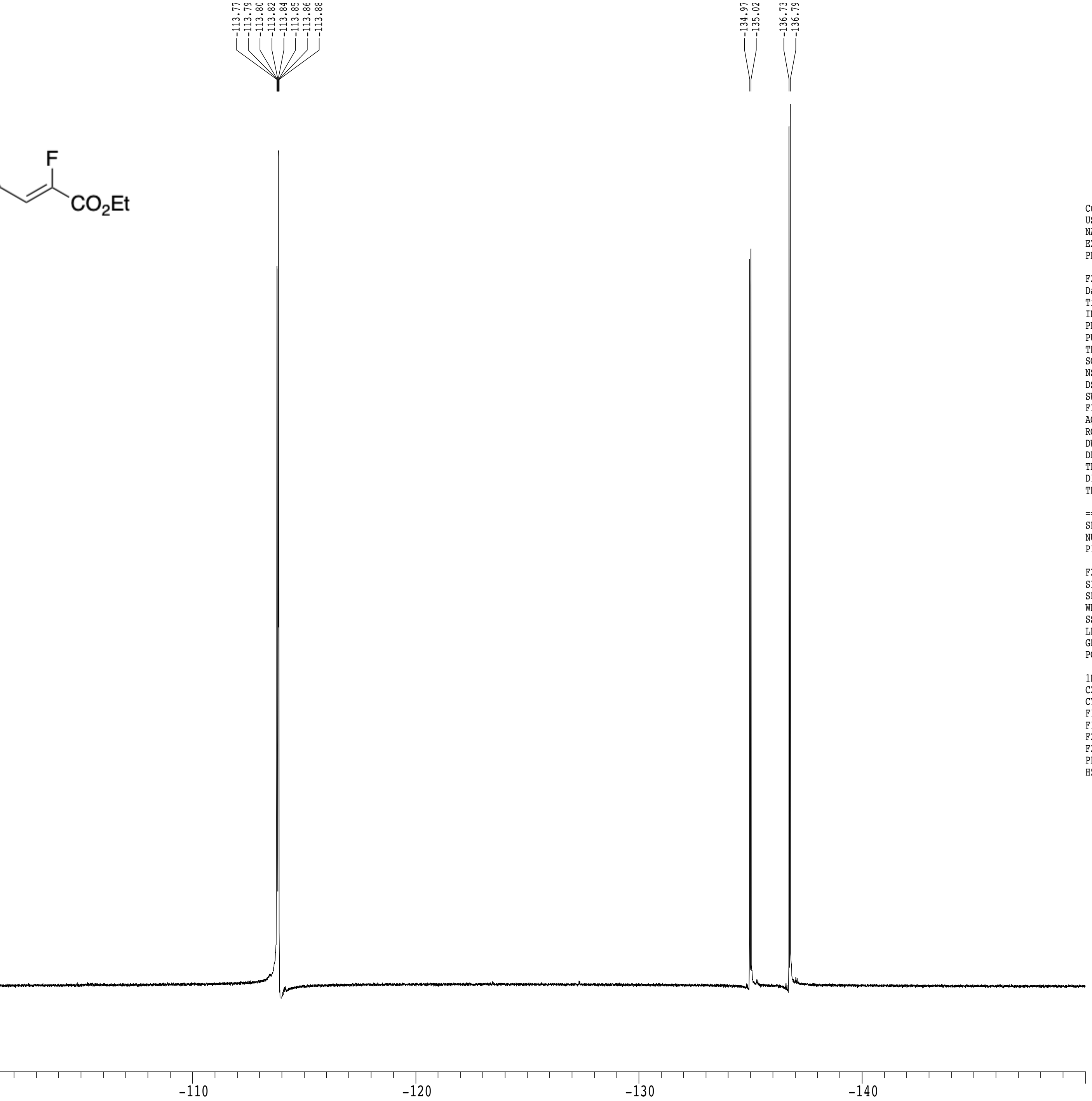


```
Current Data Parameters
USER            mcginnit
NAME     tmm-3-105-char
EXPNO                  3
PROCNO                 1

F2 - Acquisition Parameters
Date_           20210514
Time               14.08
INSTRUM            av600
PROBHD   5 mm CPBBO BB-
PULPROG         zgflqn30
TD                131072
SOLVENT            CDCl3
NS                    16
DS                     2
SWH           178571.422 Hz
FIDRES          1.362392 Hz
AQ             0.3670516 sec
RG                   575
DW                 2.800 usec
DE                 18.00 usec
TE                 298.0 K
D1            3.00000000 sec
TD0                    1

======== CHANNEL f1 ========
SFO1        564.6299196 MHz
NUC1                 19F
P1                 18.25 usec

F2 - Processing parameters
SI                131072
SF           564.6863858 MHz
WDW                   no
SSB                    0
LB                  0.00 Hz
GB                     0
PC                  1.00

1D NMR plot parameters
CX                 22.80 cm
CY                 15.00 cm
F1P              -90.000 ppm
F1             -50821.78 Hz
F2P             -150.000 ppm
F2             -84702.96 Hz
PPMCM            2.63158 ppm/cm
HZCM          1486.01685 Hz/cm
```

1H spectrum

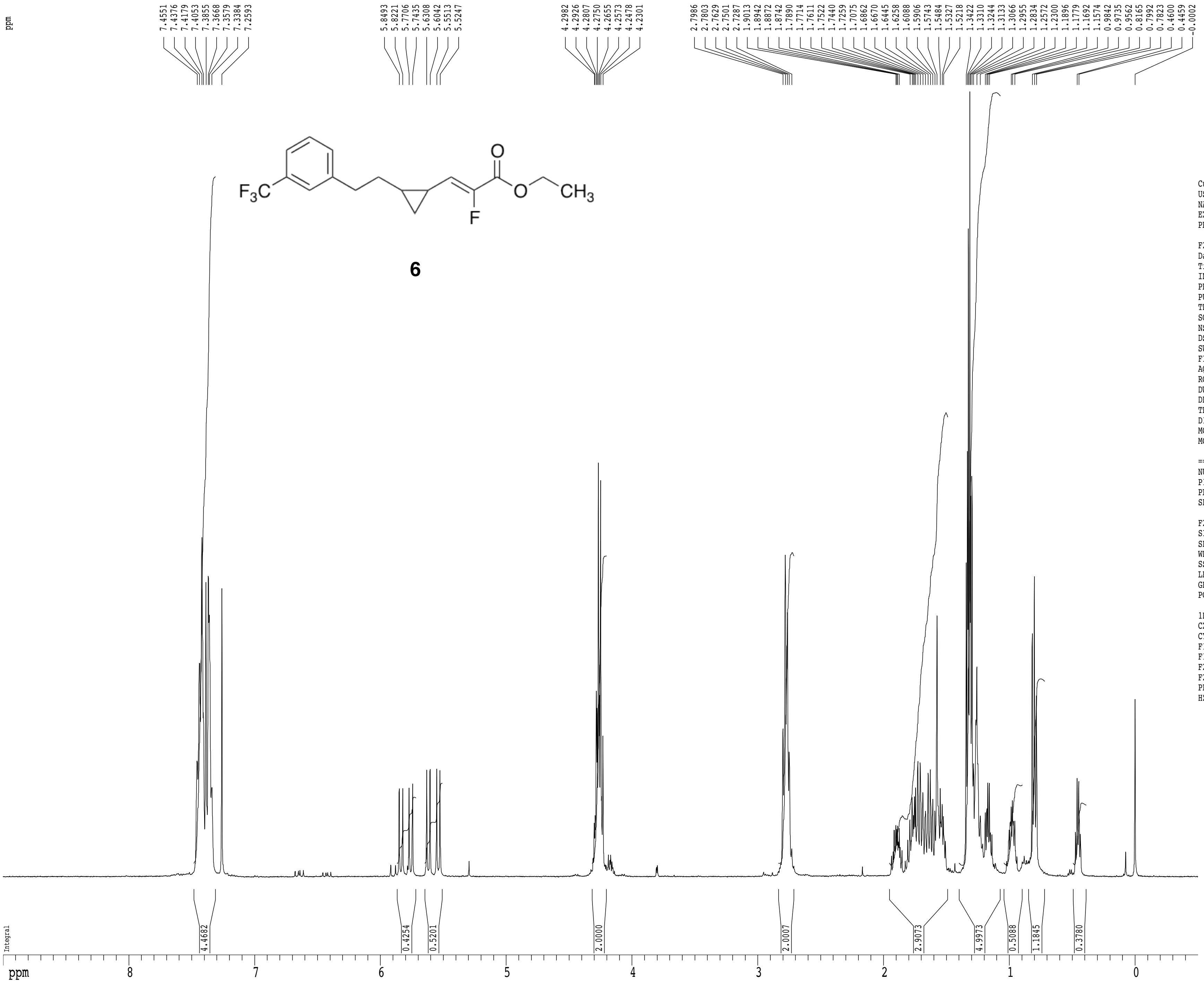


Current Data Parameters
USER linpc2
NAME pcl-2-029
EXPNO 2
PROCNO 1

F2 - Acquisition Parameters
Date_ 20210517
Time 10.00
INSTRUM drx400
PROBHD 5 mm QNP H/F/P
PULPROG zg30
TD 65536
SOLVENT CDCl3
NS 8
DS 2
SWH 6410.256 Hz
FIDRES 0.097813 Hz
AQ 5.1118579 sec
RG 256
DW 78.000 usec
DE 4.50 usec
TE 298.0 K
D1 0.10000000 sec
MCREST 0.00000000 sec
MCWRK 0.01500000 sec

======== CHANNEL f1 ========
NUC1 1H
P1 12.00 usec
PL1 -1.60 dB
SFO1 400.1328009 MHz

F2 - Processing parameters
SI 65536
SF 400.1300219 MHz
WDW EM
SSB 0
LB 0.30 Hz
GB 0
PC 2.00

1D NMR plot parameters
CX 22.80 cm
CY 15.00 cm
F1P 9.000 ppm
F1 3601.17 Hz
F2P -0.500 ppm
F2 -200.06 Hz
PPMCM 0.41667 ppm/cm
HZCM 166.72086 Hz/cm

13C spectrum

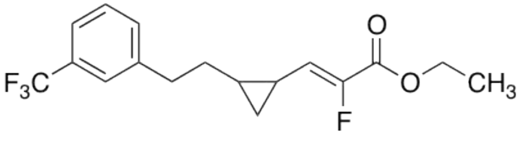


**6**

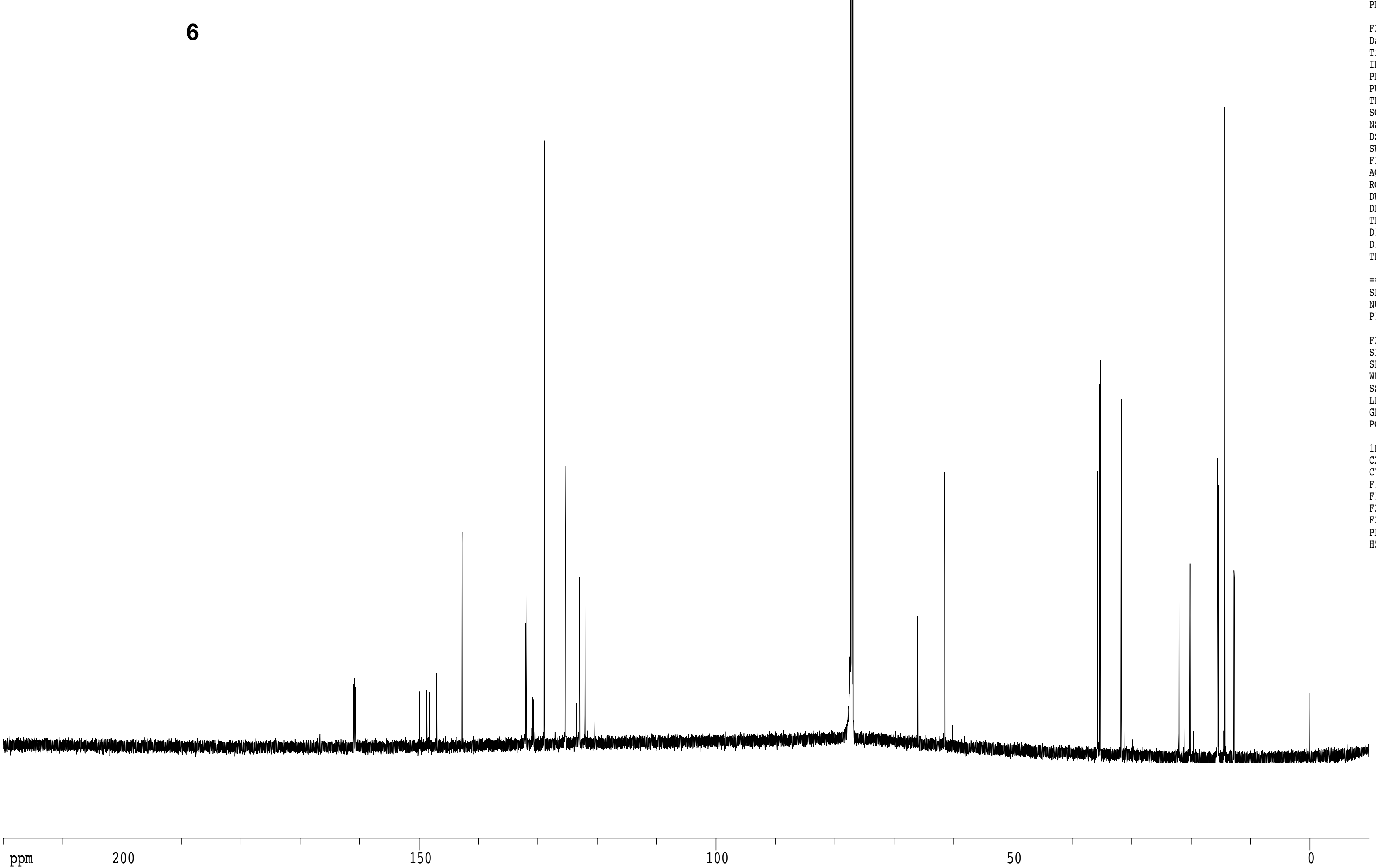


Current Data Parameters
USER linpc2
NAME pcl-1-231
EXPNO 7
PROCNO 1

F2 - Acquisition Parameters
Date_ 20210819
Time 14.38
INSTRUM av600
PROBHD 5 mm CPBBO BB-
PULPROG zgdc30
TD 65536
SOLVENT CDCl3T
NS 1024
DS 4
SWH 36231.883 Hz
FIDRES 0.552855 Hz
AQ 0.9044468 sec
RG 2050
DW 13.800 usec
DE 19.63 usec
TE 298.0 K
D1 0.40000001 sec
D11 0.03000000 sec
TD0 1

======== CHANNEL f1 ========
SFO1 150.9194080 MHz
NUC1 13C
P1 10.10 usec

F2 - Processing parameters
SI 65536
SF 150.9027936 MHz
WDW no
SSB 0
LB 0.00 Hz
GB 0
PC 1.00

1D NMR plot parameters
CX 22.80 cm
CY 45.00 cm
F1P 220.000 ppm
F1 33198.61 Hz
F2P -10.000 ppm
F2 -1509.03 Hz
PPMCM 10.08772 ppm/cm
HZCM 1522.26501 Hz/cm

19F spectrum

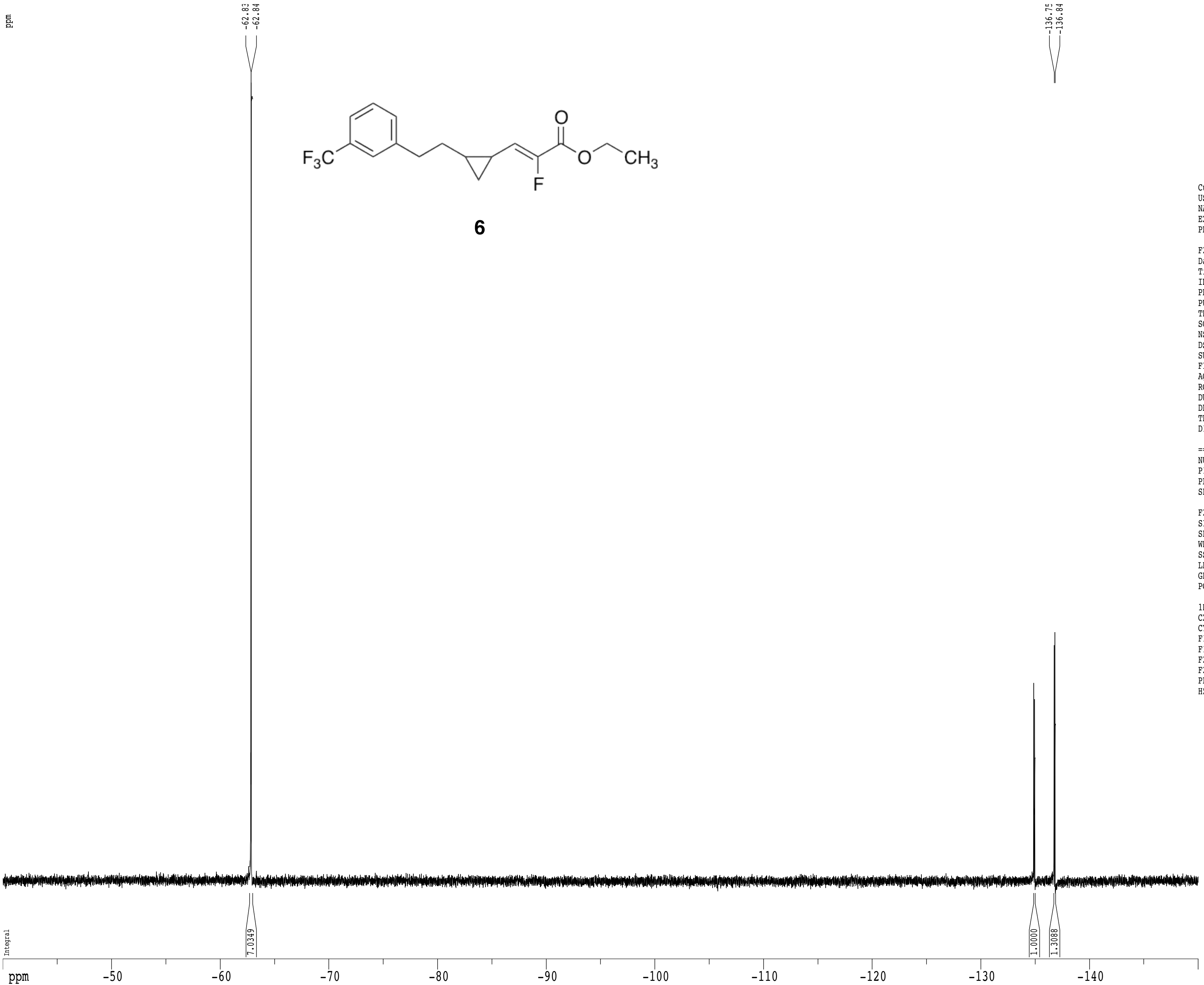


Current Data Parameters
USER linpc2
NAME pcl-2-029
EXPNO 4
PROCNO 1

F2 - Acquisition Parameters
Date_ 20210517
Time 10.10
INSTRUM drx400
PROBHD 5 mm QNP H/F/P
PULPROG zgflqn30
TD 65536
SOLVENT CDCl3
NS 16
DS 2
SWH 75187.969 Hz
FIDRES 1.147277 Hz
AQ 0.4358644 sec
RG 5792.6
DW 6.650 usec
DE 9.46 usec
TE 298.0 K
D1 2.00000000 sec

======== CHANNEL f1 ========
NUC1 19F
P1 11.75 usec
PL1 -6.00 dB
SFO1 376.4646491 MHz

F2 - Processing parameters
SI 65536
SF 376.4984640 MHz
WDW EM
SSB 0
LB 1.00 Hz
GB 0
PC 1.00

1D NMR plot parameters
CX 22.80 cm
CY 30.00 cm
F1P -40.000 ppm
F1 -15059.94 Hz
F2P -150.000 ppm
F2 -56474.77 Hz
PPMCM 4.82456 ppm/cm
HZCM 1816.44006 Hz/cm

1H spectrum

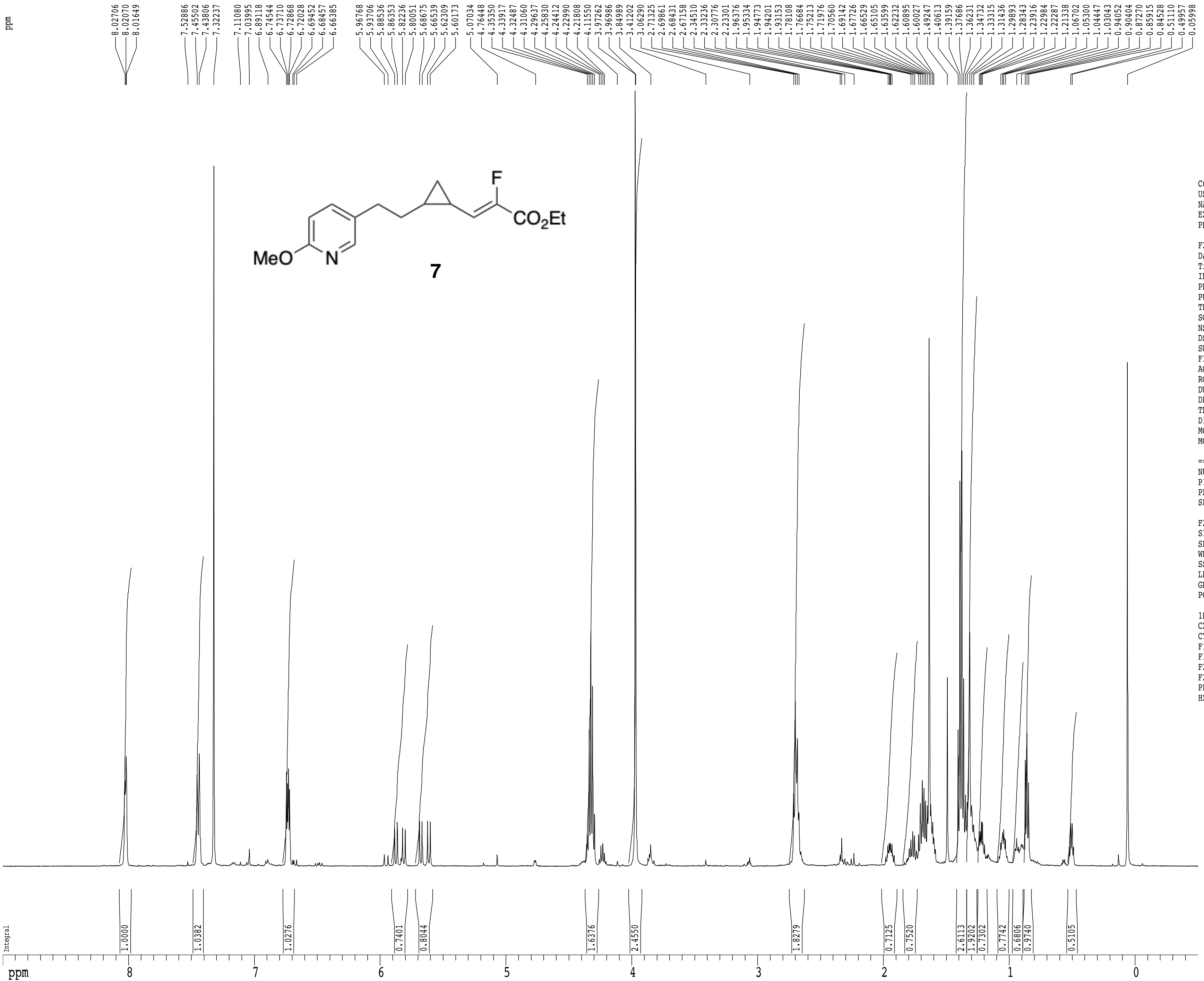


Current Data Parameters
USER mcginnit
NAME tmm-3-228a-char
EXPNO 1
PROCNO 1

F2 - Acquisition Parameters
Date_ 20211223
Time 11.42
INSTRUM cryo500
PROBHD 5 mm CPTCI 1H-
PULPROG zg30
TD 81728
SOLVENT CDCl3
NS 8
DS 2
SWH 8012.820 Hz
FIDRES 0.098043 Hz
AQ 5.0998774 sec
RG 9
DW 62.400 usec
DE 6.00 usec
TE 298.0 K
D1 0.10000000 sec
MCREST 0.00000000 sec
MCWRK 0.01500000 sec

======== CHANNEL f1 ========
NUC1 1H
P1 9.75 usec
PL1 1.60 dB
SFO1 500.2235015 MHz

F2 - Processing parameters
SI 65536
SF 500.2200000 MHz
WDW EM
SSB 0
LB 0.30 Hz
GB 0
PC 1.00

1D NMR plot parameters
CX 22.80 cm
CY 15.00 cm
F1P 9.000 ppm
F1 4501.98 Hz
F2P -0.500 ppm
F2 -250.11 Hz
PPMCM 0.41667 ppm/cm
HZCM 208.42500 Hz/cm

13C spectrum

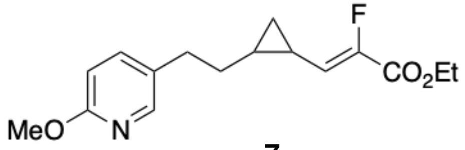


**7**

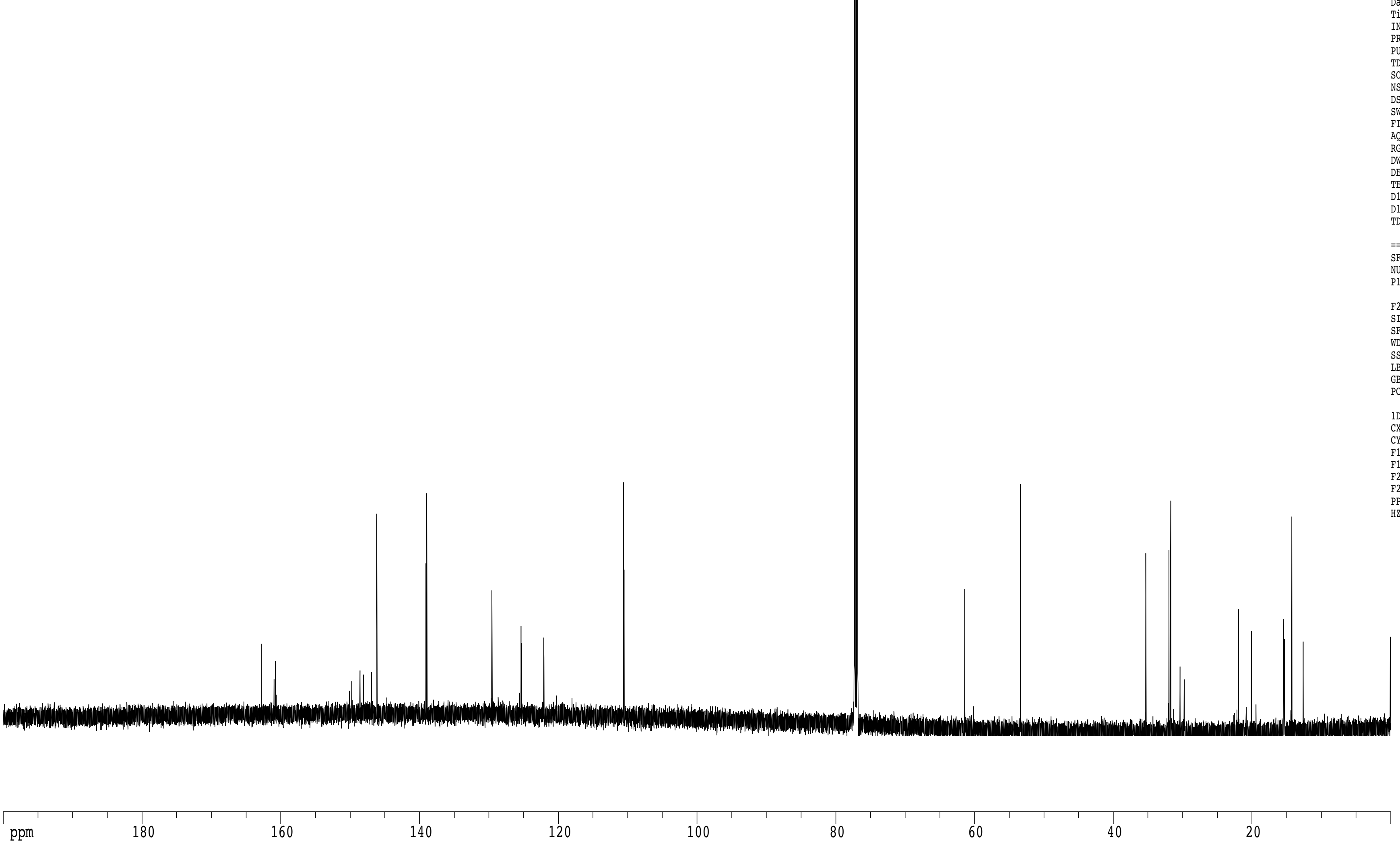


```
Current Data Parameters
USER          mcginnit
NAME     tmm-3-228a-char
EXPNO               12
PROCNO               1

F2 - Acquisition Parameters
Date_         20211223
Time             13.04
INSTRUM          av600
PROBHD   5 mm CPBBO BB-
PULPROG         zgdc30
TD               65536
SOLVENT          CDCl3
NS                 514
DS                   4
SWH          36231.883 Hz
FIDRES        0.552855 Hz
AQ           0.9044468 sec
RG                2050
DW              13.800 usec
DE               19.63 usec
TE               298.0 K
D1          0.40000001 sec
D11         0.03000000 sec
TD0                  1

======== CHANNEL f1 ========
SFO1       150.9194080 MHz
NUC1               13C
P1               10.10 usec

F2 - Processing parameters
SI               65536
SF         150.9028085 MHz
WDW                 no
SSB                  0
LB                0.00 Hz
GB                   0
PC                1.00

1D NMR plot parameters
CX               22.80 cm
CY               75.00 cm
F1P            200.000 ppm
F1            30180.56 Hz
F2P              0.000 ppm
F2                0.00 Hz
PPMCM          8.77193 ppm/cm
HZCM        1323.70886 Hz/cm
```

19F spectrum

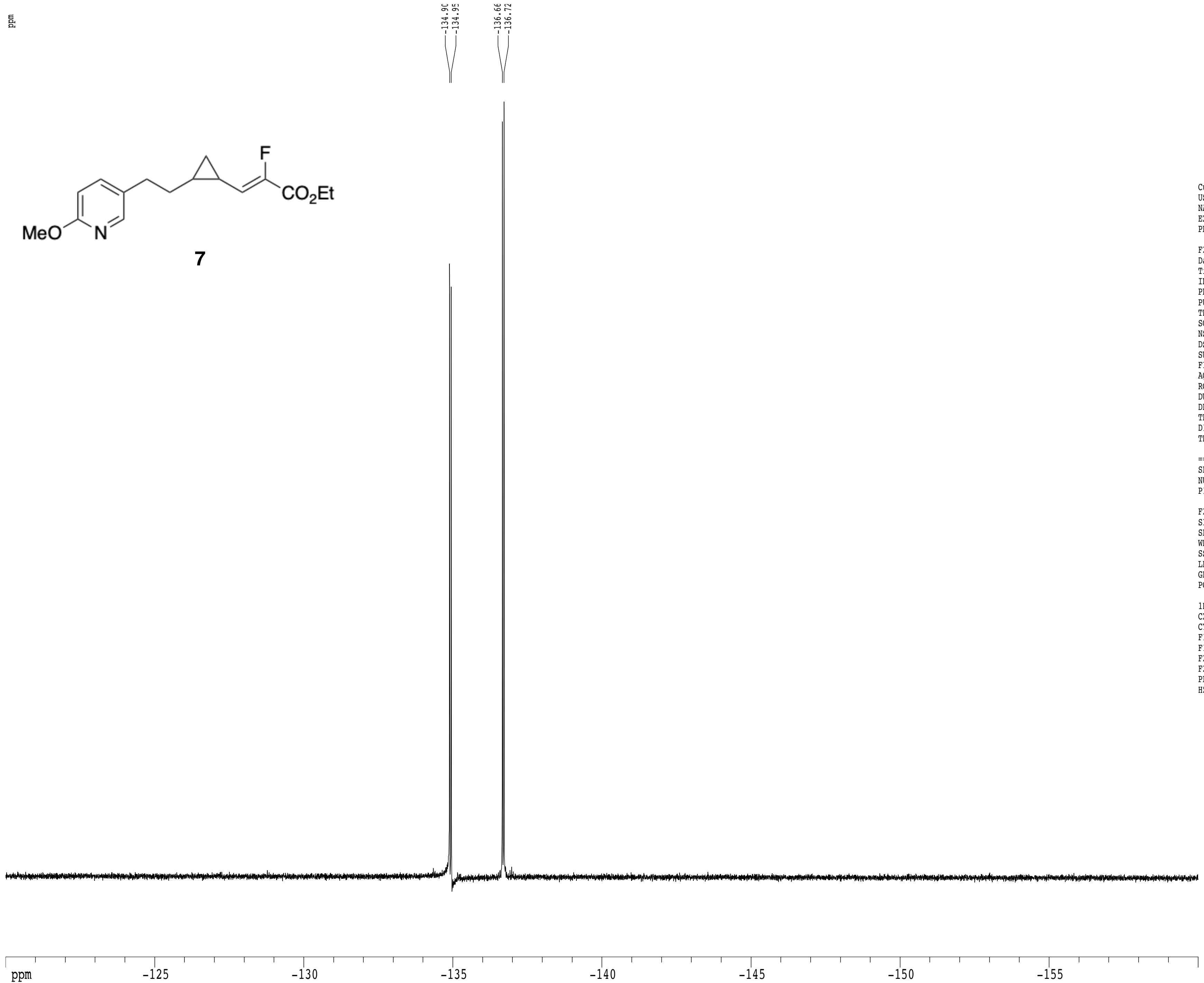


Current Data Parameters
USER mcginnit
NAME tmm-3-228a-char
EXPNO 3
PROCNO 1

F2 - Acquisition Parameters
Date_ 20211223
Time 12.59
INSTRUM av600
PROBHD 5 mm CPBBO BB-
PULPROG zgflqn30
TD 131072
SOLVENT CDCl3
NS 16
DS 2
SWH 178571.422 Hz
FIDRES 1.362392 Hz
AQ 0.3670516 sec
RG 575
DW 2.800 usec
DE 18.00 usec
TE 298.0 K
D1 3.00000000 sec
TD0 1

======== CHANNEL f1 ========
SFO1 564.6299196 MHz
NUC1 19F
P1 18.25 usec

F2 - Processing parameters
SI 131072
SF 564.6863858 MHz
WDW no
SSB 0
LB 0.00 Hz
GB 0
PC 1.00

1D NMR plot parameters
CX 22.80 cm
CY 15.00 cm
F1P -120.000 ppm
F1 -67762.38 Hz
F2P -160.000 ppm
F2 -90349.83 Hz
PPMCM 1.75439 ppm/cm
HZCM 990.67773 Hz/cm

1H spectrum

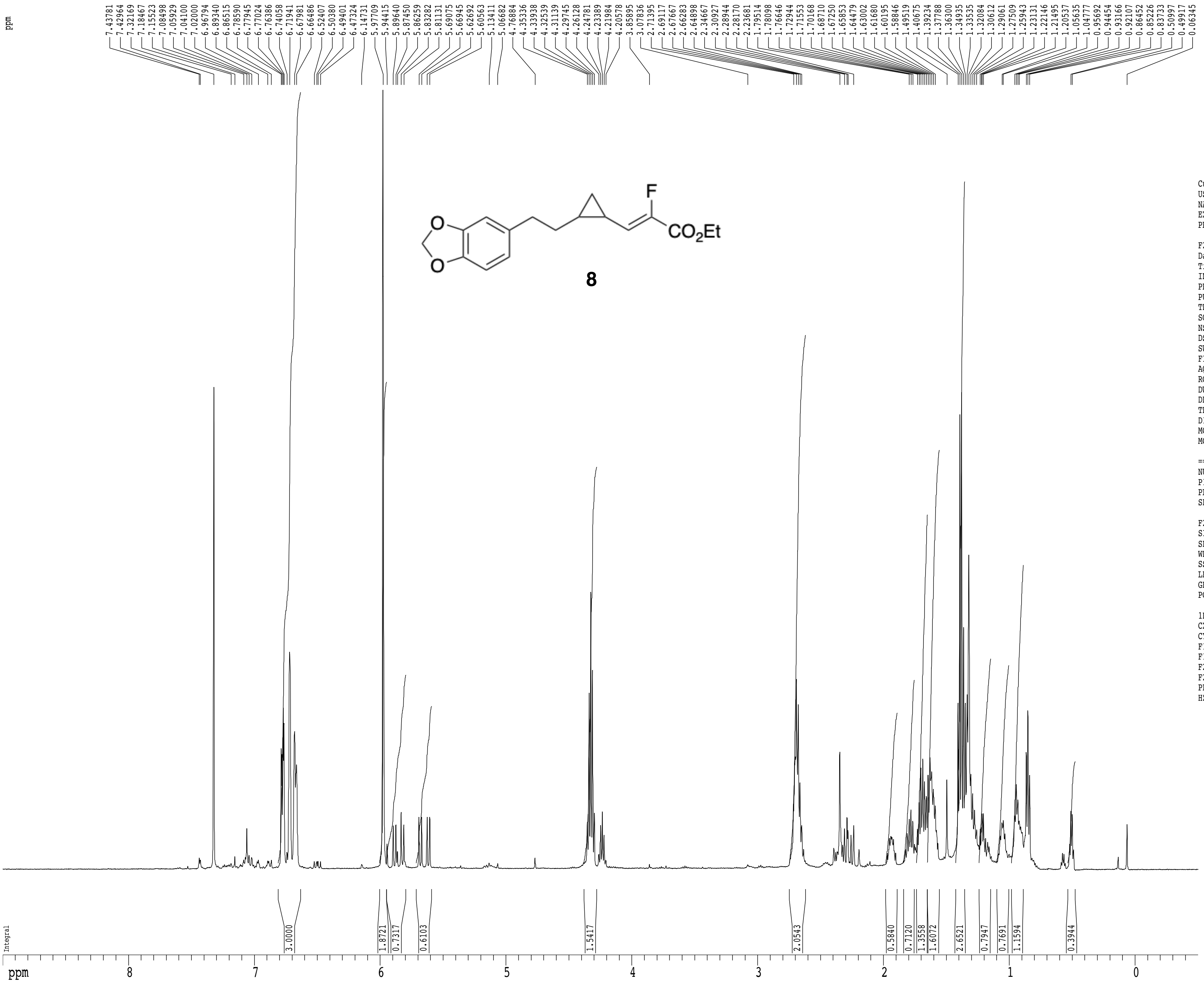


```
Current Data Parameters
USER             mcginnit
NAME           tmm-3-146
EXPNO                   6
PROCNO                  1

F2 - Acquisition Parameters
Date_            20210607
Time                15.33
INSTRUM          cryo500
PROBHD   5 mm CPTCI 1H-
PULPROG              zg30
TD                  81728
SOLVENT             CDCl3
NS                      8
DS                      2
SWH             8012.820 Hz
FIDRES          0.098043 Hz
AQ             5.0998774 sec
RG                    5.7
DW                 62.400 usec
DE                   6.00 usec
TE                  298.0 K
D1             0.10000000 sec
MCREST         0.00000000 sec
MCWRK          0.01500000 sec

======== CHANNEL f1 ========
NUC1                   1H
P1                   9.75 usec
PL1                  1.60 dB
SFO1         500.2235015 MHz

F2 - Processing parameters
SI                  65536
SF           500.2200000 MHz
WDW                    EM
SSB                     0
LB                   0.30 Hz
GB                      0
PC                   1.00

1D NMR plot parameters
CX                  22.80 cm
CY                  15.00 cm
F1P                 9.000 ppm
F1                4501.98 Hz
F2P                -0.500 ppm
F2                -250.11 Hz
PPMCM             0.41667 ppm/cm
HZCM            208.42500 Hz/cm
```

Z-restored spin-echo 13C spectrum with 1H decoupling

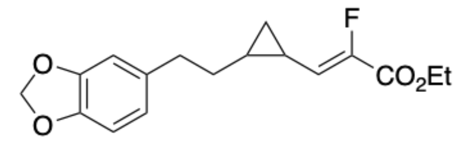

8

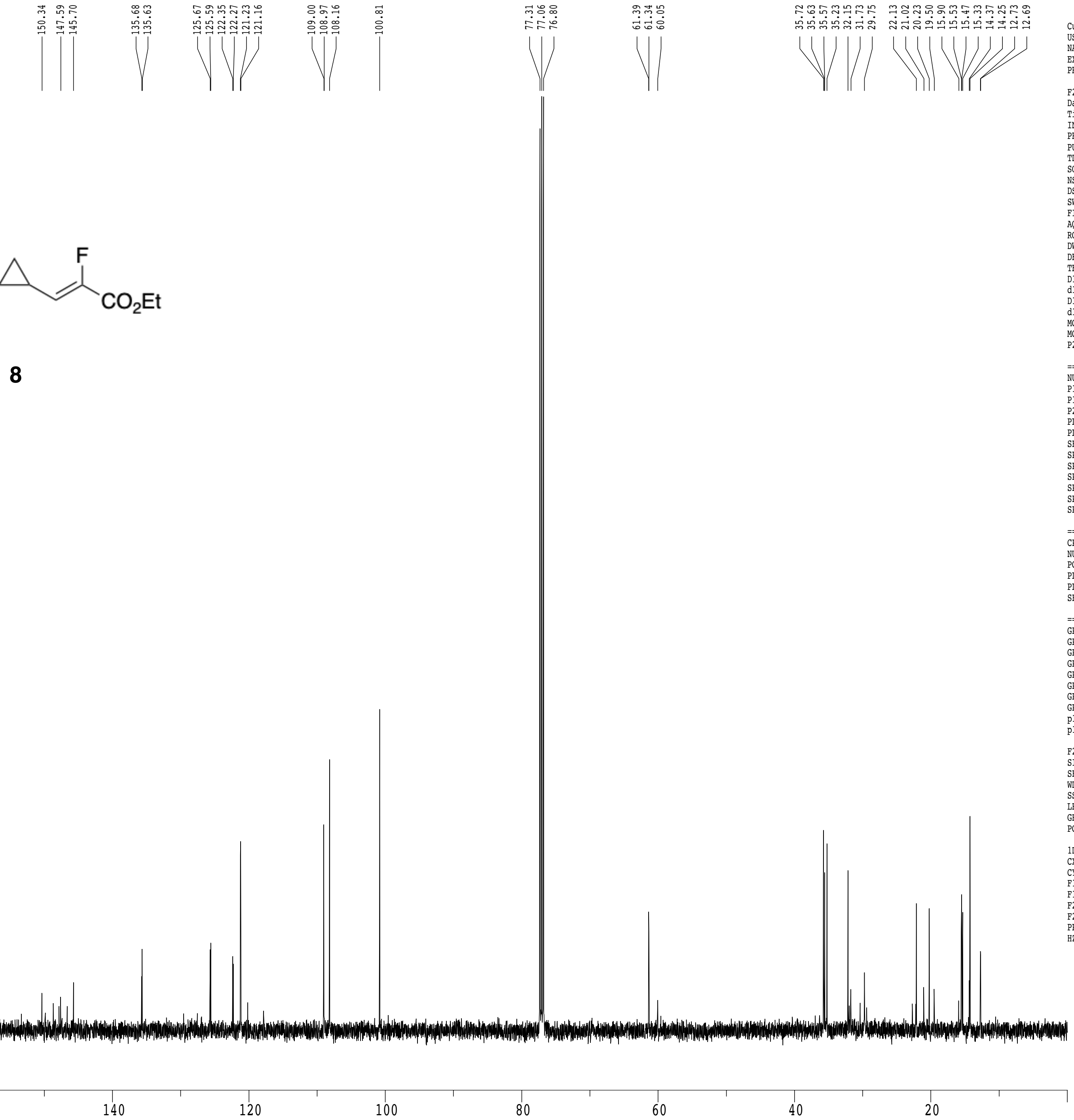


Current Data Parameters
USER mcginnit
NAME tmm-3-146
EXPNO 7
PROCNO 1

F2 - Acquisition Parameters
Date_ 20210607
Time 15.37
INSTRUM cryo500
PROBHD 5 mm CPTCI 1H-
PULPROG SpinEchopg30gp2.prd
TD 65536
SOLVENT CDCl3
NS 600
DS 16
SWH 30303.031 Hz
FIDRES 0.462388 Hz
AQ 1.0813940 sec
RG 2896.3
DW 16.500 usec
DE 6.00 usec
TE 298.0 K
D1 0.25000000 sec
d11 0.03000000 sec
D16 0.00020000 sec
d17 0.00019600 sec
MCREST 0.00000000 sec
MCWRK 0.01500000 sec
P2 37.70 usec

======== CHANNEL f1 ========
NUC1 13C
P1 18.85 usec
P12 2000.00 usec
P20 500.00 usec
PL0 120.00 dB
PL1 -1.00 dB
SFO1 125.7942548 MHz
SP2 1.55 dB
SP4 1.55 dB
SPNAM2 Crp60comp.4
SPNAM4 Crp60,0.5,20.1
SPOFF2 0.00 Hz
SPOFF4 0.00 Hz

======== CHANNEL f2 ========
CPDPRG2 waltz16
NUC2 1H
PCPD2 100.00 usec
PL2 1.60 dB
PL12 22.00 dB
SFO2 500.2225011 MHz

====== GRADIENT CHANNEL =====
GPNAM1 SINE.100
GPNAM2 SINE.100
GPX1 0.00 %
GPX2 0.00 %
GPY1 0.00 %
GPY2 0.00 %
GPZ1 30.00 %
GPZ2 50.00 %
p15 500.00 usec
p16 1000.00 usec

F2 - Processing parameters
SI 65536
SF 125.7804190 MHz
WDW EM
SSB 0
LB 1.00 Hz
GB 0
PC 2.00

1D NMR plot parameters
CX 22.80 cm
CY 15.65 cm
F1P 200.000 ppm
F1 25156.08 Hz
F2P 0.000 ppm
F2 0.00 Hz
PPMCM 8.77193 ppm/cm
HZCM 1103.33704 Hz/cm

19F spectrum

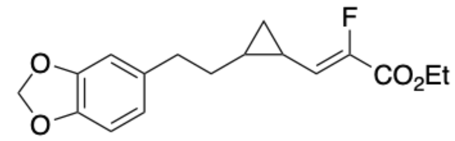

8

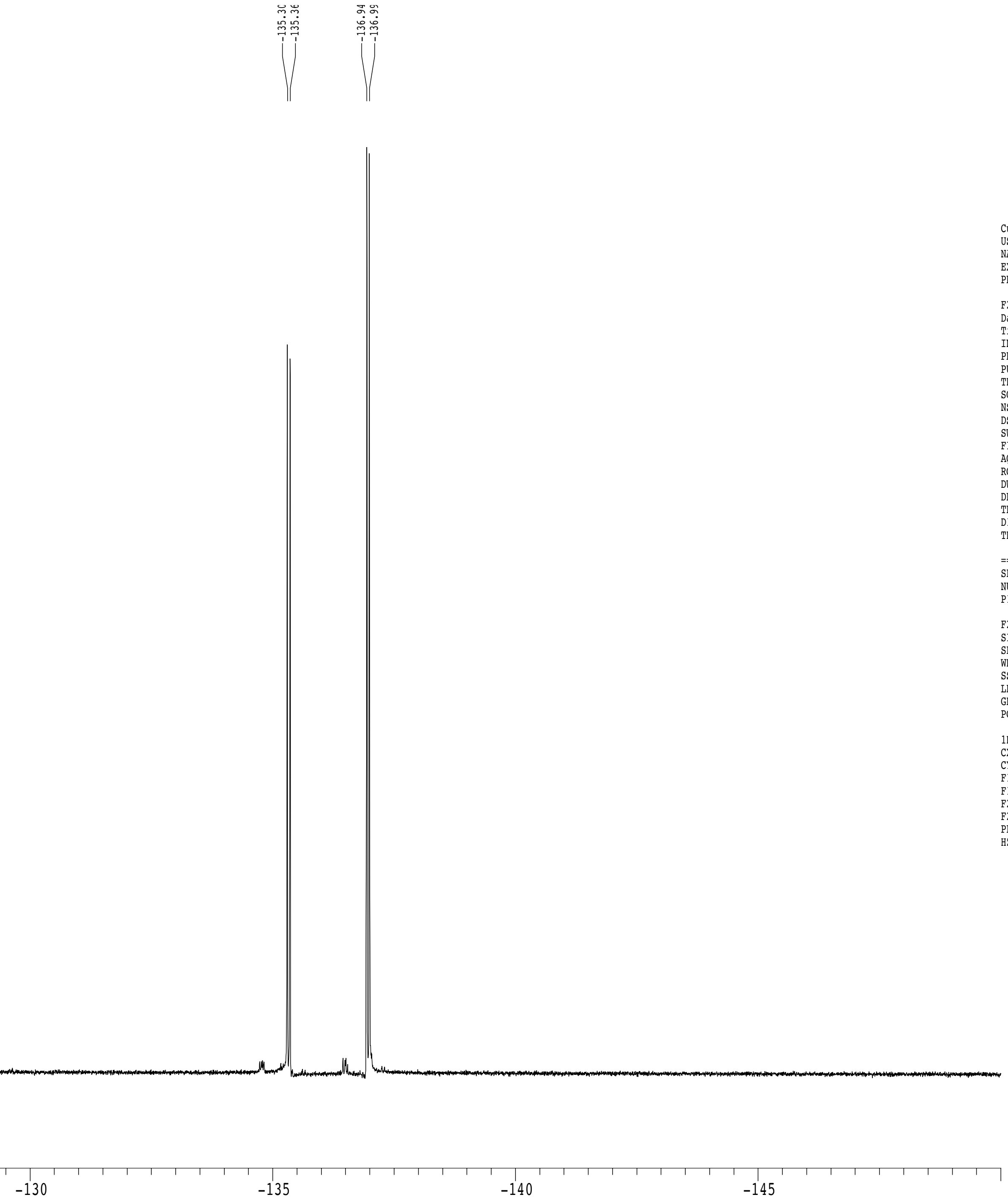


Current Data Parameters
USER mcginnit
NAME tmm-3-146
EXPNO 8
PROCNO 1

F2 - Acquisition Parameters
Date_ 20210607
Time 15.58
INSTRUM av600
PROBHD 5 mm CPBBO BB-
PULPROG zgflqn30
TD 131072
SOLVENT CDCl3
NS 16
DS 2
SWH 178571.422 Hz
FIDRES 1.362392 Hz
AQ 0.3670516 sec
RG 575
DW 2.800 usec
DE 18.00 usec
TE 298.0 K
D1 3.00000000 sec
TD0 1

======== CHANNEL f1 ========
SFO1 564.6299196 MHz
NUC1 19F
P1 18.25 usec

F2 - Processing parameters
SI 131072
SF 564.6863858 MHz
WDW no
SSB 0
LB 0.00 Hz
GB 0
PC 1.00

1D NMR plot parameters
CX 22.80 cm
CY 15.00 cm
F1P -120.000 ppm
F1 -67762.37 Hz
F2P -150.000 ppm
F2 -84702.96 Hz
PPMCM 1.31579 ppm/cm
HZCM 743.00842 Hz/cm

1H spectrum

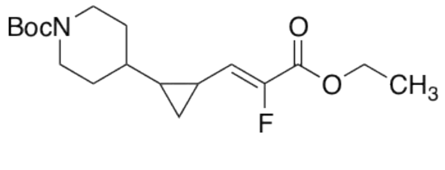


**9**

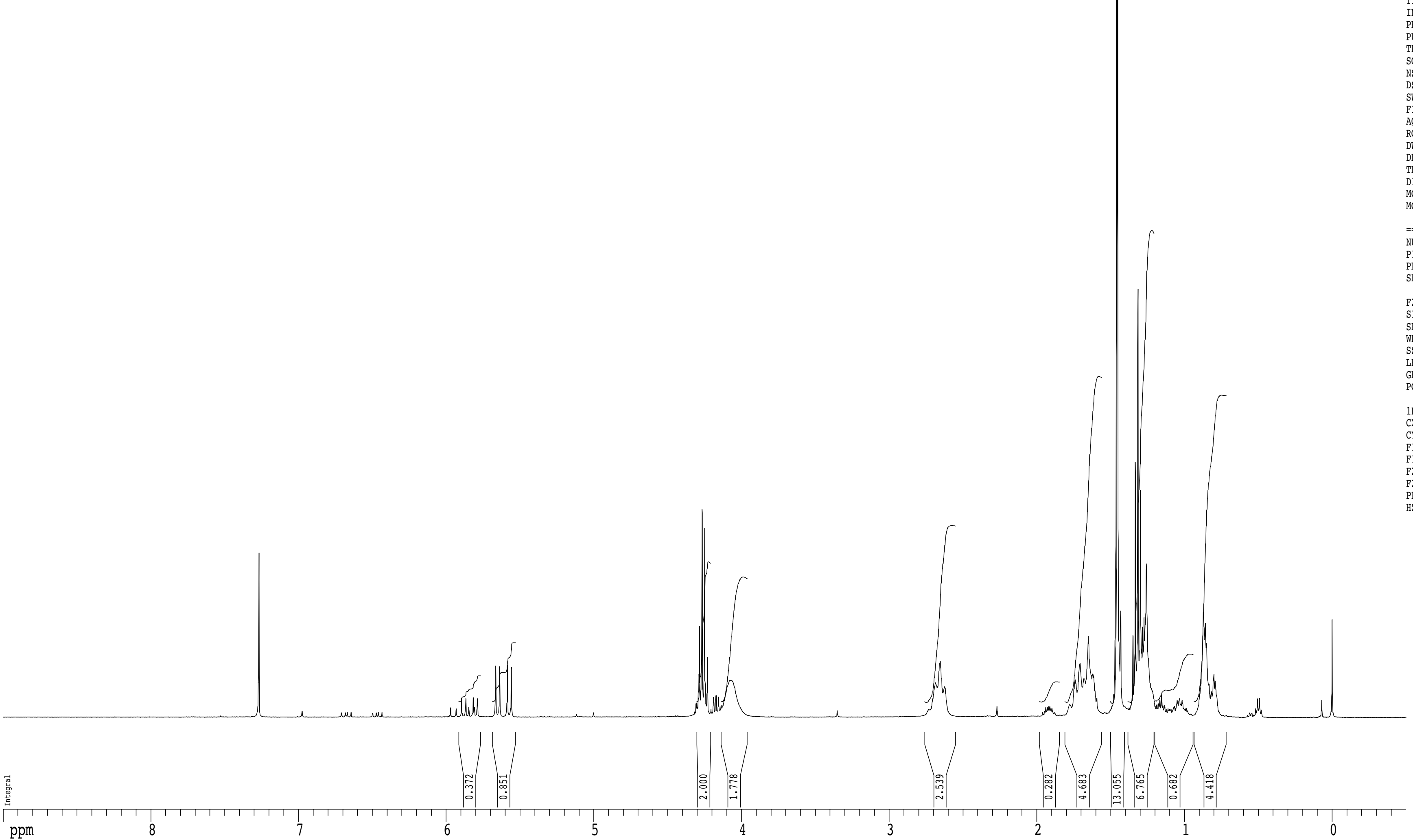


Current Data Parameters
USER linpc2
NAME pcl-2-115
EXPNO 2
PROCNO 1

F2 - Acquisition Parameters
Date_ 20210720
Time 8.22
INSTRUM drx400
PROBHD 5 mm QNP H/F/P
PULPROG zg30
TD 65536
SOLVENT CDCl3
NS 8
DS 2
SWH 6410.256 Hz
FIDRES 0.097813 Hz
AQ 5.1118579 sec
RG 203.2
DW 78.000 usec
DE 4.50 usec
TE 298.0 K
D1 0.10000000 sec
MCREST 0.00000000 sec
MCWRK 0.01500000 sec

======== CHANNEL f1 ========
NUC1 1H
P1 12.00 usec
PL1 -1.60 dB
SFO1 400.1328009 MHz

F2 - Processing parameters
SI 65536
SF 400.1300178 MHz
WDW EM
SSB 0
LB 0.30 Hz
GB 0
PC 2.00

1D NMR plot parameters
CX 22.80 cm
CY 45.00 cm
F1P 9.000 ppm
F1 3601.17 Hz
F2P -0.500 ppm
F2 -200.06 Hz
PPMCM 0.41667 ppm/cm
HZCM 166.72084 Hz/cm

Z-restored spin-echo 13C spectrum with 1H decoupling

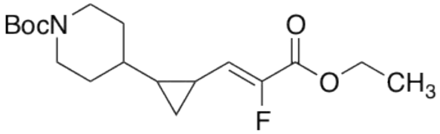

**9**

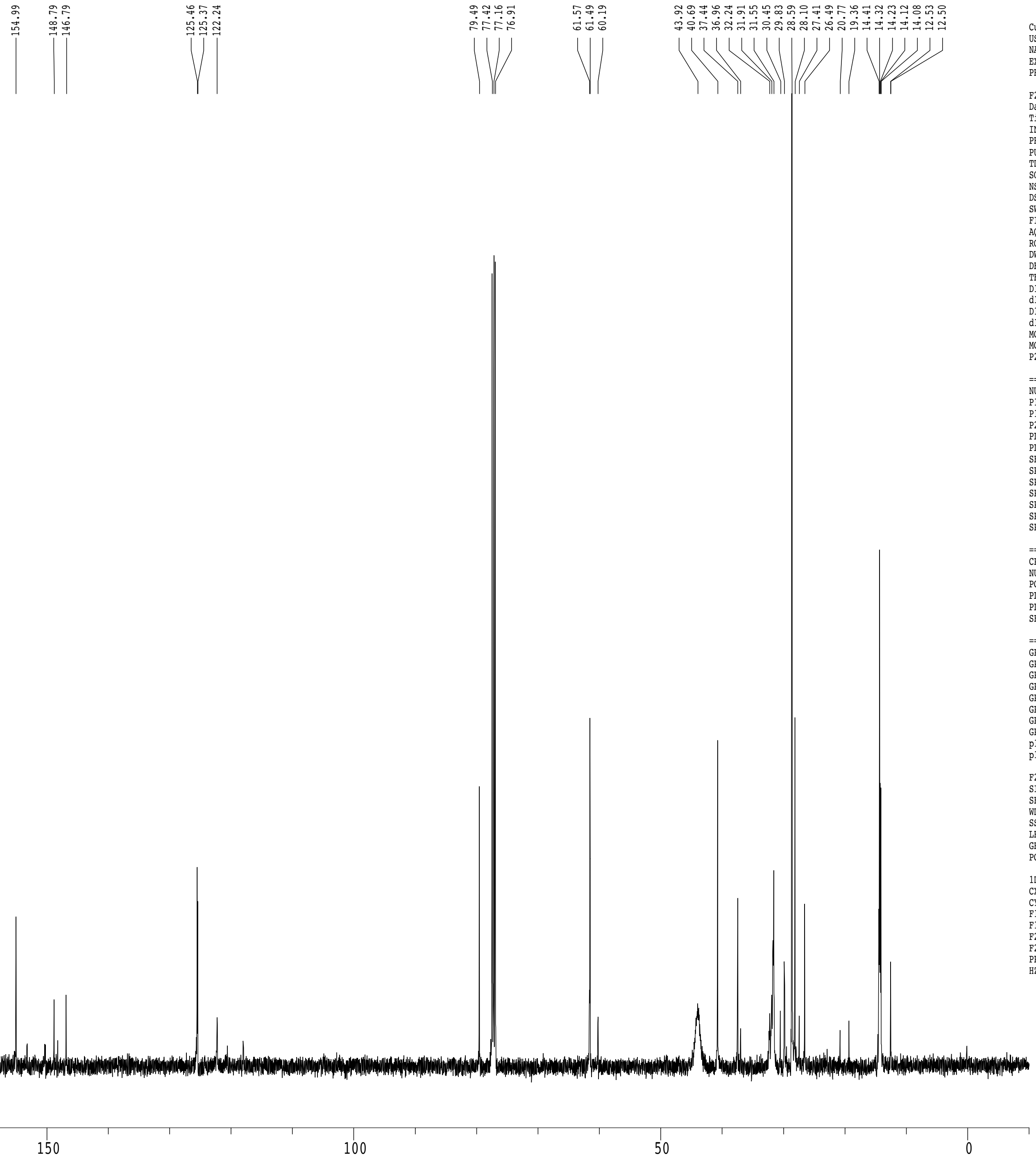


```
Current Data Parameters
USER              linpc2
NAME           pcl-2-115
EXPNO                  6
PROCNO                 1

F2 - Acquisition Parameters
Date_           20210721
Time                8.17
INSTRUM          cryo500
PROBHD   5 mm CPTCI 1H-
PULPROG  SpinEchopg30gp2.prd
TD                 65536
SOLVENT            CDCl3
NS                  1024
DS                    16
SWH            30303.031 Hz
FIDRES          0.462388 Hz
AQ             1.0813940 sec
RG                  6502
DW                16.500 usec
DE                  6.00 usec
TE                 298.0 K
D1            0.25000000 sec
d11           0.03000000 sec
D16           0.00020000 sec
d17           0.00019600 sec
MCREST        0.00000000 sec
MCWRK         0.01500000 sec
P2                 37.70 usec

======== CHANNEL f1 ========
NUC1                 13C
P1                 18.85 usec
P12              2000.00 usec
P20               500.00 usec
PL0               120.00 dB
PL1                -1.00 dB
SFO1         125.7942548 MHz
SP2                 1.55 dB
SP4                 1.55 dB
SPNAM2       Crp60comp.4
SPNAM4    Crp60,0.5,20.1
SPOFF2              0.00 Hz
SPOFF4              0.00 Hz

======== CHANNEL f2 ========
CPDPRG2          waltz16
NUC2                  1H
PCPD2             100.00 usec
PL2                 1.60 dB
PL12               22.00 dB
SFO2         500.2225011 MHz

====== GRADIENT CHANNEL =====
GPNAM1          SINE.100
GPNAM2          SINE.100
GPX1                0.00 %
GPX2                0.00 %
GPY1                0.00 %
GPY2                0.00 %
GPZ1               30.00 %
GPZ2               50.00 %
p15               500.00 usec
p16              1000.00 usec

F2 - Processing parameters
SI                 65536
SF           125.7804071 MHz
WDW                   EM
SSB                    0
LB                  1.00 Hz
GB                     0
PC                  2.00

1D NMR plot parameters
CX                 22.80 cm
CY                 40.00 cm
F1P              220.000 ppm
F1              27671.69 Hz
F2P              -10.000 ppm
F2              -1257.80 Hz
PPMCM           10.08772 ppm/cm
HZCM          1268.83752 Hz/cm
```

19F spectrum

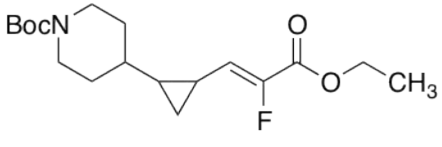


**9**

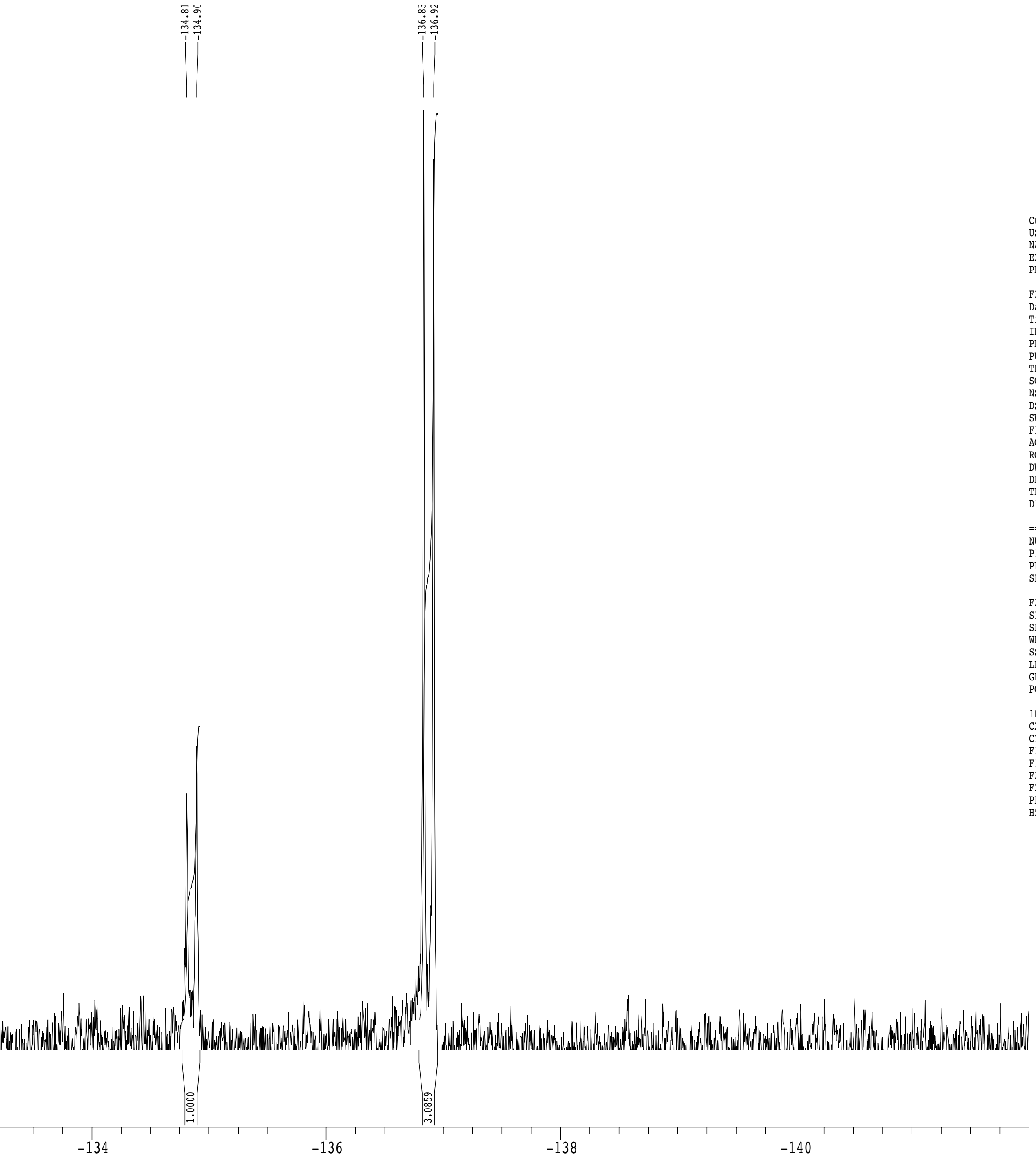


```
Current Data Parameters
USER          linpc2
NAME          pcl-2-115
EXPNO         4
PROCNO        1

F2 - Acquisition Parameters
Date_         20210720
Time          8.35
INSTRUM       drx400
PROBHD   5 mm QNP H/F/P
PULPROG       zgflqn30
TD            65536
SOLVENT       CDCl3
NS            40
DS            2
SWH           75187.969 Hz
FIDRES        1.147277 Hz
AQ            0.4358644 sec
RG            11585.2
DW            6.650 usec
DE            9.46 usec
TE            298.0 K
D1            2.00000000 sec

======== CHANNEL f1 ========
NUC1          19F
P1            11.75 usec
PL1           -6.00 dB
SFO1          376.4646491 MHz

F2 - Processing parameters
SI            65536
SF            376.4984640 MHz
WDW           EM
SSB           0
LB            1.00 Hz
GB            0
PC            1.00

1D NMR plot parameters
CX            22.80 cm
CY            15.00 cm
F1P           -130.000 ppm
F1            -48944.80 Hz
F2P           -142.000 ppm
F2            -53462.79 Hz
PPMCM         0.52632 ppm/cm
HZCM          198.15710 Hz/cm
```

**SFC Chiracel OD-H, 2% IPA/$CO_2$, 1.5 mL/min**

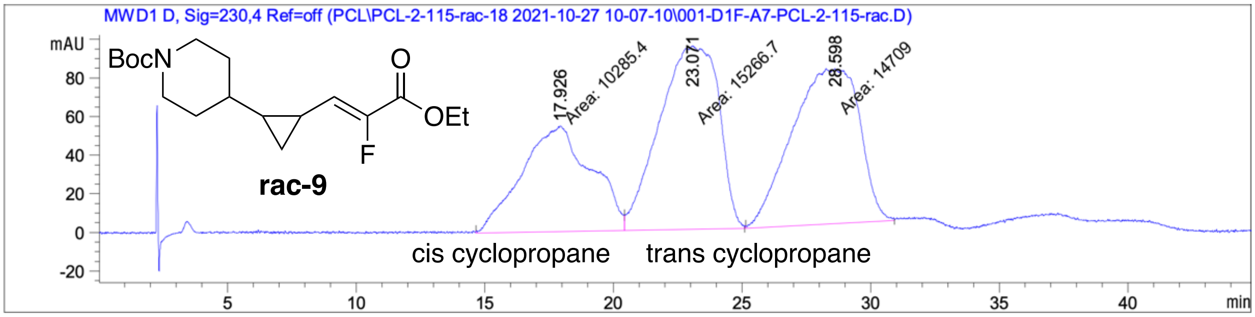


Signal 4: MWD1 D, Sig=230,4 Ref=off

| Peak # | RetTime [min] | Type | Width [min] | Area [mAU*s] | Height [mAU] | Area % |
|---|---|---|---|---|---|---|
| 1 | 17.926 | MF | 3.1328 | 1.02854e4 | 54.71899 | 25.5468 |
| 2 | 23.071 | FM | 2.6810 | 1.52667e4 | 94.90704 | 37.9192 |
| 3 | 28.598 | MM | 3.0613 | 1.47090e4 | 80.08051 | 36.5341 |

Totals : 4.02611e4 229.70653

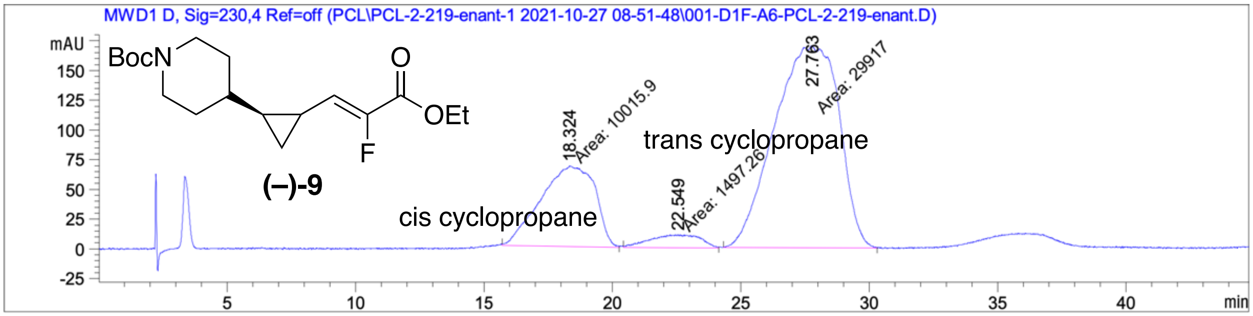


Signal 4: MWD1 D, Sig=230,4 Ref=off

| Peak # | RetTime [min] | Type | Width [min] | Area [mAU*s] | Height [mAU] | Area % |
|---|---|---|---|---|---|---|
| 1 | 18.324 | MM | 2.4653 | 1.00159e4 | 67.71262 | 24.1754 |
| 2 | 22.549 | MM | 2.2634 | 1497.26184 | 11.02496 | 3.6139 |
| 3 | 27.763 | MM | 2.9345 | 2.99170e4 | 169.91434 | 72.2106 |

Totals : 4.14302e4 248.65191

1H spectrum

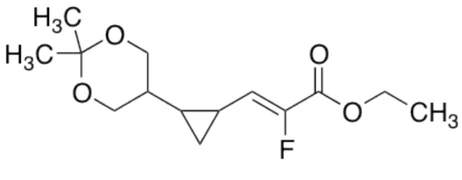


**10**

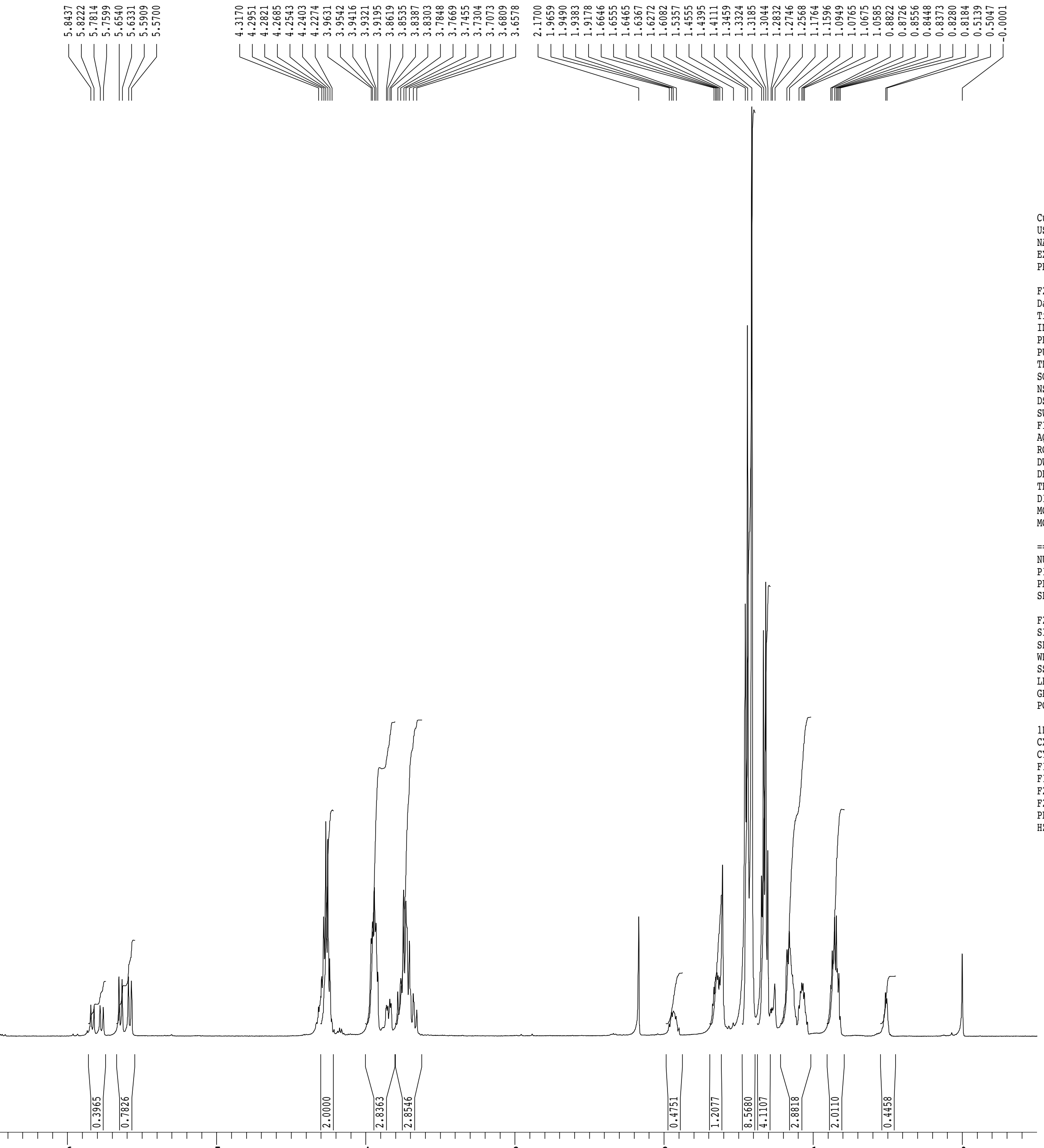


Current Data Parameters
USER linpc2
NAME pcl-2-031
EXPNO 2
PROCNO 1

F2 - Acquisition Parameters
Date_ 20210609
Time 15.53
INSTRUM cryo500
PROBHD 5 mm CPTCI 1H-
PULPROG zg30
TD 81728
SOLVENT CDCl3
NS 8
DS 2
SWH 8012.820 Hz
FIDRES 0.098043 Hz
AQ 5.0998774 sec
RG 7.1
DW 62.400 usec
DE 6.00 usec
TE 298.0 K
D1 0.10000000 sec
MCREST 0.00000000 sec
MCWRK 0.01500000 sec

======== CHANNEL f1 ========
NUC1 1H
P1 9.75 usec
PL1 1.60 dB
SFO1 500.2235015 MHz

F2 - Processing parameters
SI 65536
SF 500.2200274 MHz
WDW EM
SSB 0
LB 0.30 Hz
GB 0
PC 1.00

1D NMR plot parameters
CX 22.80 cm
CY 15.00 cm
F1P 9.000 ppm
F1 4501.98 Hz
F2P -0.500 ppm
F2 -250.11 Hz
PPMCM 0.41667 ppm/cm
HZCM 208.42502 Hz/cm

Z-restored spin-echo 13C spectrum with 1H decoupling

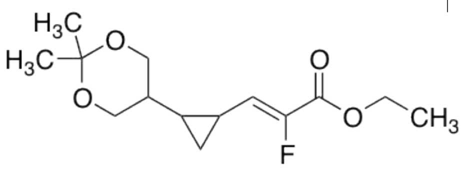


**10**

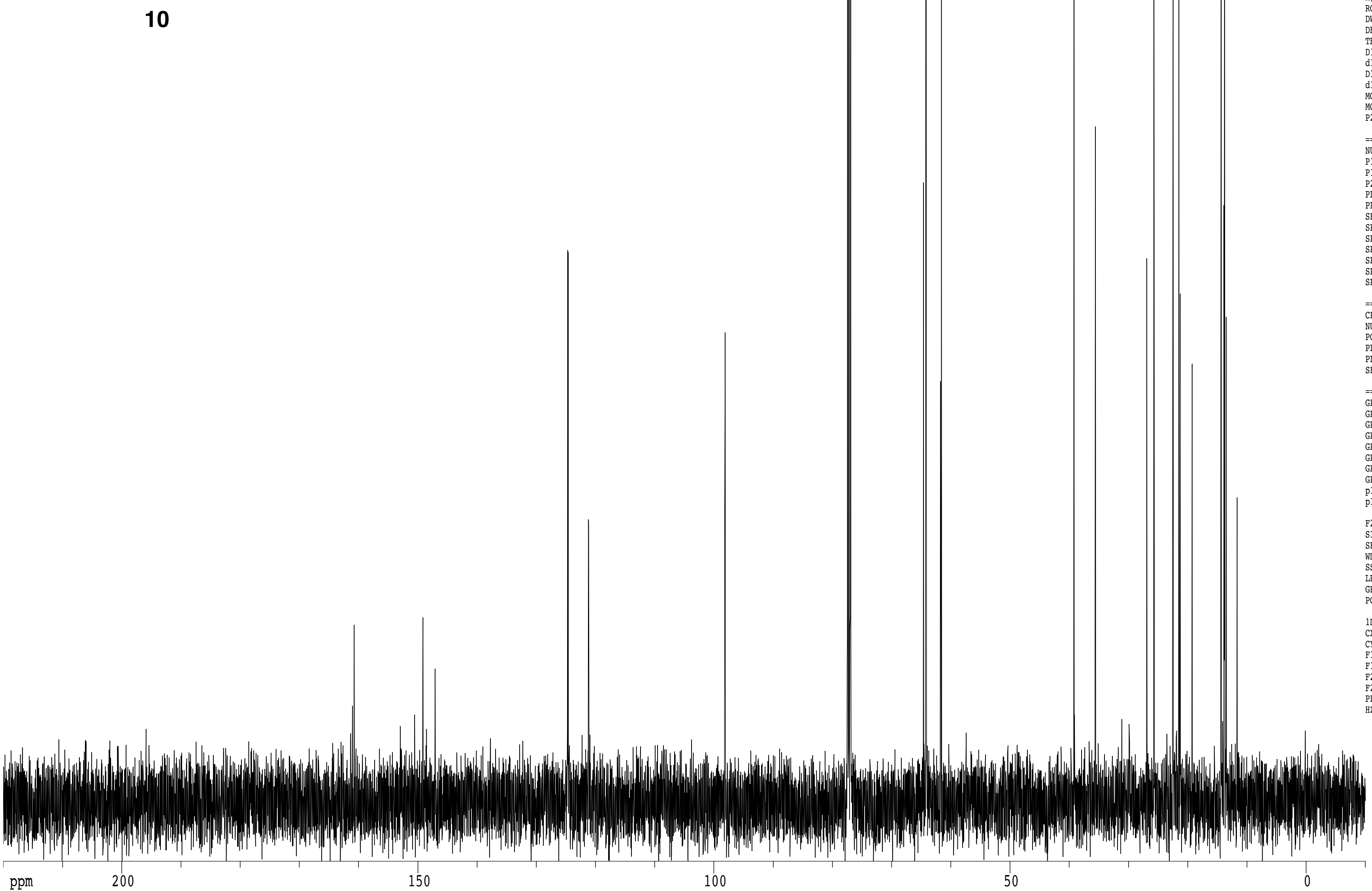


```
Current Data Parameters
USER            linpc2
NAME          pcl-2-031
EXPNO                3
PROCNO               1

F2 - Acquisition Parameters
Date_         20210609
Time             15.56
INSTRUM        cryo500
PROBHD   5 mm CPTCI 1H-
PULPROG  SpinEchopg30gp2.prd
TD               65536
SOLVENT          CDCl3
NS                 208
DS                  16
SWH          30303.031 Hz
FIDRES        0.462388 Hz
AQ           1.0813940 sec
RG              7298.2
DW              16.500 usec
DE                6.00 usec
TE               298.0 K
D1          0.25000000 sec
d11         0.03000000 sec
D16         0.00020000 sec
d17         0.00019600 sec
MCREST      0.00000000 sec
MCWRK       0.01500000 sec
P2               37.70 usec

======== CHANNEL f1 ========
NUC1               13C
P1               18.85 usec
P12            2000.00 usec
P20             500.00 usec
PL0             120.00 dB
PL1              -1.00 dB
SFO1       125.7942548 MHz
SP2               1.55 dB
SP4               1.55 dB
SPNAM2     Crp60comp.4
SPNAM4   Crp60,0.5,20.1
SPOFF2            0.00 Hz
SPOFF4            0.00 Hz

======== CHANNEL f2 ========
CPDPRG2        waltz16
NUC2                1H
PCPD2           100.00 usec
PL2               1.60 dB
PL12             22.00 dB
SFO2       500.2225011 MHz

====== GRADIENT CHANNEL =====
GPNAM1        SINE.100
GPNAM2        SINE.100
GPX1              0.00 %
GPX2              0.00 %
GPY1              0.00 %
GPY2              0.00 %
GPZ1             30.00 %
GPZ2             50.00 %
p15             500.00 usec
p16            1000.00 usec

F2 - Processing parameters
SI               65536
SF         125.7804066 MHz
WDW                 EM
SSB                  0
LB                1.00 Hz
GB                   0
PC                2.00

1D NMR plot parameters
CX               22.80 cm
CY               45.00 cm
F1P            220.000 ppm
F1            27671.69 Hz
F2P            -10.000 ppm
F2            -1257.80 Hz
PPMCM         10.08772 ppm/cm
HZCM        1268.83740 Hz/cm
```

19F spectrum

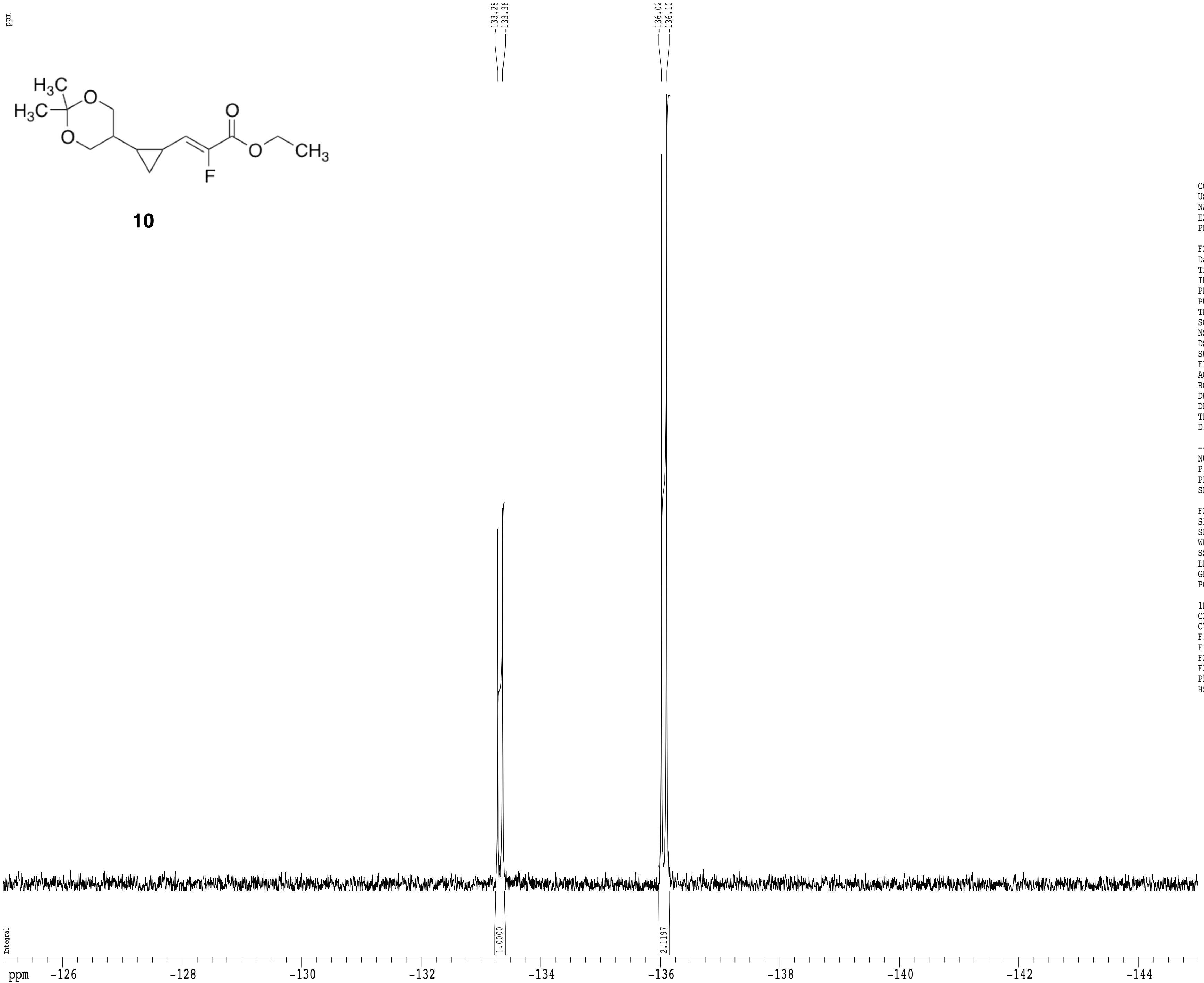

**10**


```
Current Data Parameters
USER            linpc2
NAME          pcl-2-031
EXPNO                 4
PROCNO                1

F2 - Acquisition Parameters
Date_          20210609
Time              16.13
INSTRUM          drx400
PROBHD   5 mm QNP H/F/P
PULPROG        zgflqn30
TD                65536
SOLVENT           CDCl3
NS                   64
DS                    2
SWH           75187.969 Hz
FIDRES         1.147277 Hz
AQ            0.4358644 sec
RG               7298.2
DW                6.650 usec
DE                 9.46 usec
TE                298.0 K
D1           2.00000000 sec

======== CHANNEL f1 ========
NUC1                19F
P1                11.75 usec
PL1               -6.00 dB
SFO1        376.4646491 MHz

F2 - Processing parameters
SI                65536
SF          376.4984640 MHz
WDW                  EM
SSB                   0
LB                 1.00 Hz
GB                    0
PC                 1.00

1D NMR plot parameters
CX                22.80 cm
CY                15.00 cm
F1P            -125.000 ppm
F1            -47062.31 Hz
F2P            -145.000 ppm
F2            -54592.28 Hz
PPMCM           0.87719 ppm/cm
HZCM          330.26184 Hz/cm
```

1H spectrum

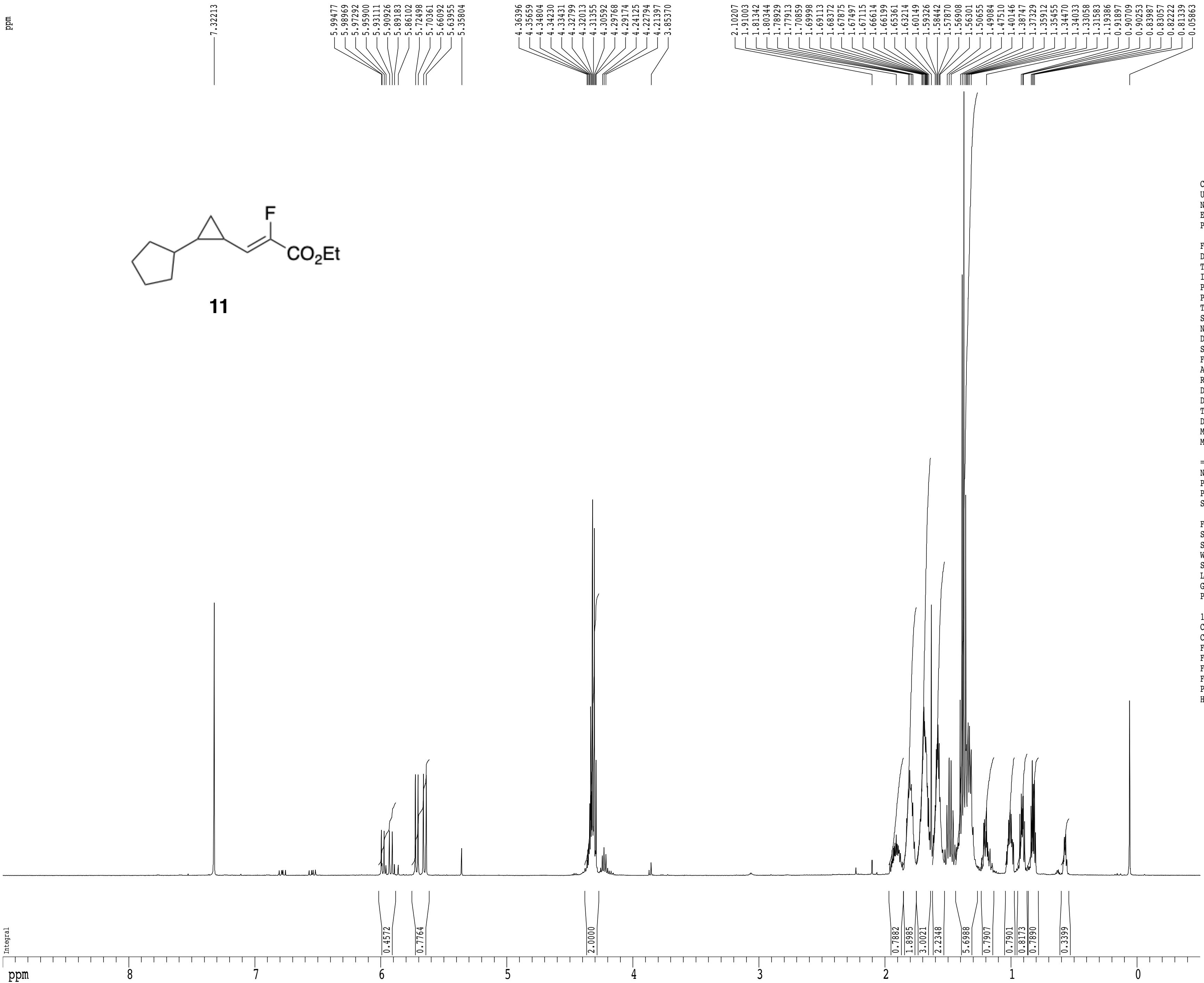


**11**

Current Data Parameters
USER mcginnit
NAME tmm-3-125-char
EXPNO 1
PROCNO 1

F2 - Acquisition Parameters
Date_ 20210514
Time 13.50
INSTRUM cryo500
PROBHD 5 mm CPTCI 1H-
PULPROG zg30
TD 81728
SOLVENT CDCl3
NS 8
DS 2
SWH 8012.820 Hz
FIDRES 0.098043 Hz
AQ 5.0998774 sec
RG 5
DW 62.400 usec
DE 6.00 usec
TE 298.0 K
D1 0.10000000 sec
MCREST 0.00000000 sec
MCWRK 0.01500000 sec

======== CHANNEL f1 ========
NUC1 1H
P1 9.75 usec
PL1 1.60 dB
SFO1 500.2235015 MHz

F2 - Processing parameters
SI 65536
SF 500.2200000 MHz
WDW EM
SSB 0
LB 0.30 Hz
GB 0
PC 1.00

1D NMR plot parameters
CX 22.80 cm
CY 15.00 cm
F1P 9.000 ppm
F1 4501.98 Hz
F2P -0.500 ppm
F2 -250.11 Hz
PPMCM 0.41667 ppm/cm
HZCM 208.42500 Hz/cm

Z-restored spin-echo 13C spectrum with 1H decoupling

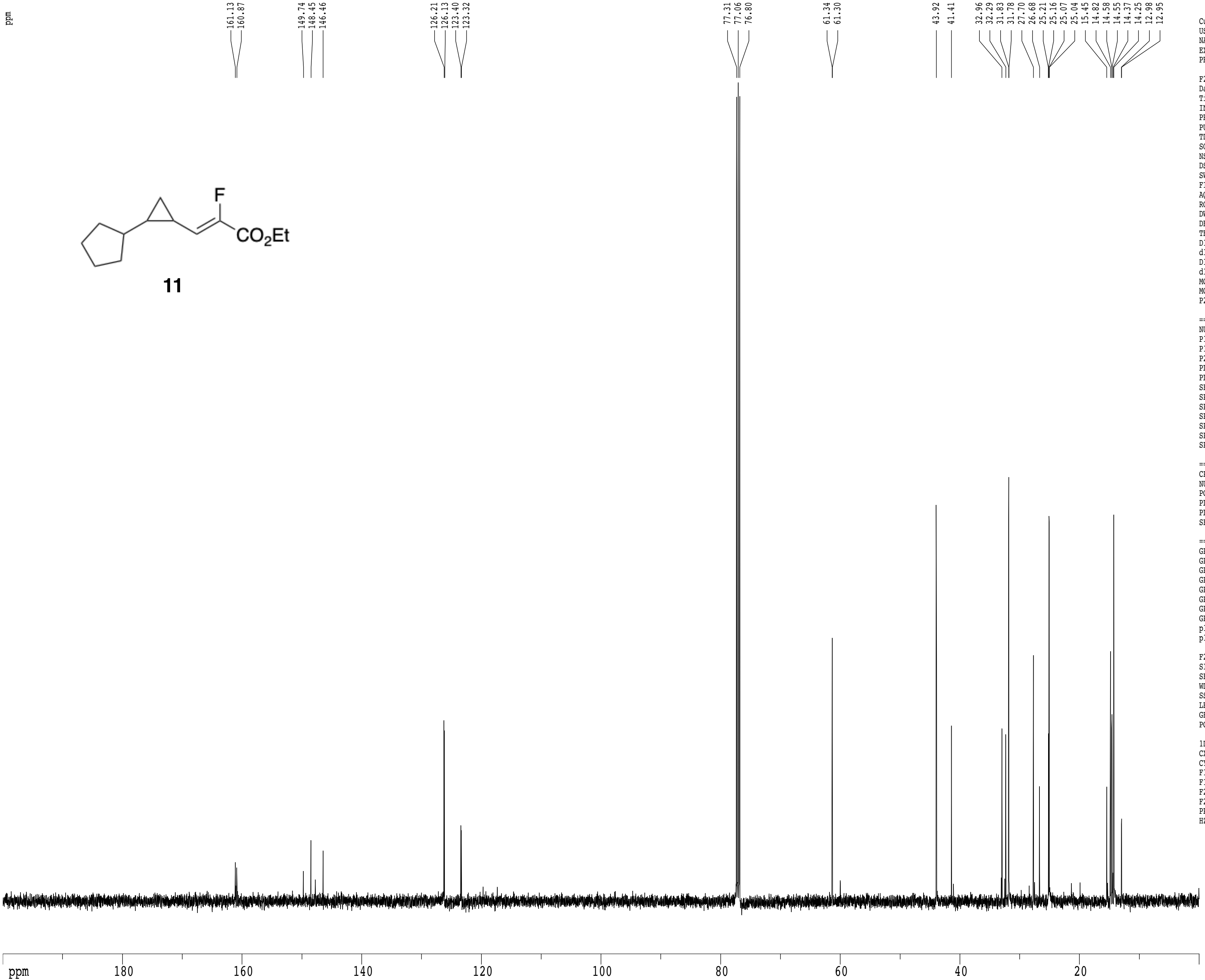


**11**

Current Data Parameters
USER mcginnit
NAME tmm-3-125-char
EXPNO 2
PROCNO 1

F2 - Acquisition Parameters
Date_ 20210514
Time 13.58
INSTRUM cryo500
PROBHD 5 mm CPTCI 1H-
PULPROG SpinEchopg30gp2.prd
TD 65536
SOLVENT CDCl3
NS 327
DS 16
SWH 30303.031 Hz
FIDRES 0.462388 Hz
AQ 1.0813940 sec
RG 7298.2
DW 16.500 usec
DE 6.00 usec
TE 298.0 K
D1 0.25000000 sec
d11 0.03000000 sec
D16 0.00020000 sec
d17 0.00019600 sec
MCREST 0.00000000 sec
MCWRK 0.01500000 sec
P2 37.70 usec

======== CHANNEL f1 ========
NUC1 13C
P1 18.85 usec
P12 2000.00 usec
P20 500.00 usec
PL0 120.00 dB
PL1 -1.00 dB
SFO1 125.7942548 MHz
SP2 1.55 dB
SP4 1.55 dB
SPNAM2 Crp60comp.4
SPNAM4 Crp60,0.5,20.1
SPOFF2 0.00 Hz
SPOFF4 0.00 Hz

======== CHANNEL f2 ========
CPDPRG2 waltz16
NUC2 1H
PCPD2 100.00 usec
PL2 1.60 dB
PL12 22.00 dB
SFO2 500.2225011 MHz

====== GRADIENT CHANNEL =====
GPNAM1 SINE.100
GPNAM2 SINE.100
GPX1 0.00 %
GPX2 0.00 %
GPY1 0.00 %
GPY2 0.00 %
GPZ1 30.00 %
GPZ2 50.00 %
p15 500.00 usec
p16 1000.00 usec

F2 - Processing parameters
SI 65536
SF 125.7804190 MHz
WDW EM
SSB 0
LB 1.00 Hz
GB 0
PC 2.00

1D NMR plot parameters
CX 22.80 cm
CY 15.65 cm
F1P 200.000 ppm
F1 25156.08 Hz
F2P 0.000 ppm
F2 0.00 Hz
PPMCM 8.77193 ppm/cm
HZCM 1103.33704 Hz/cm

19F spectrum

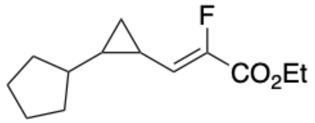

11

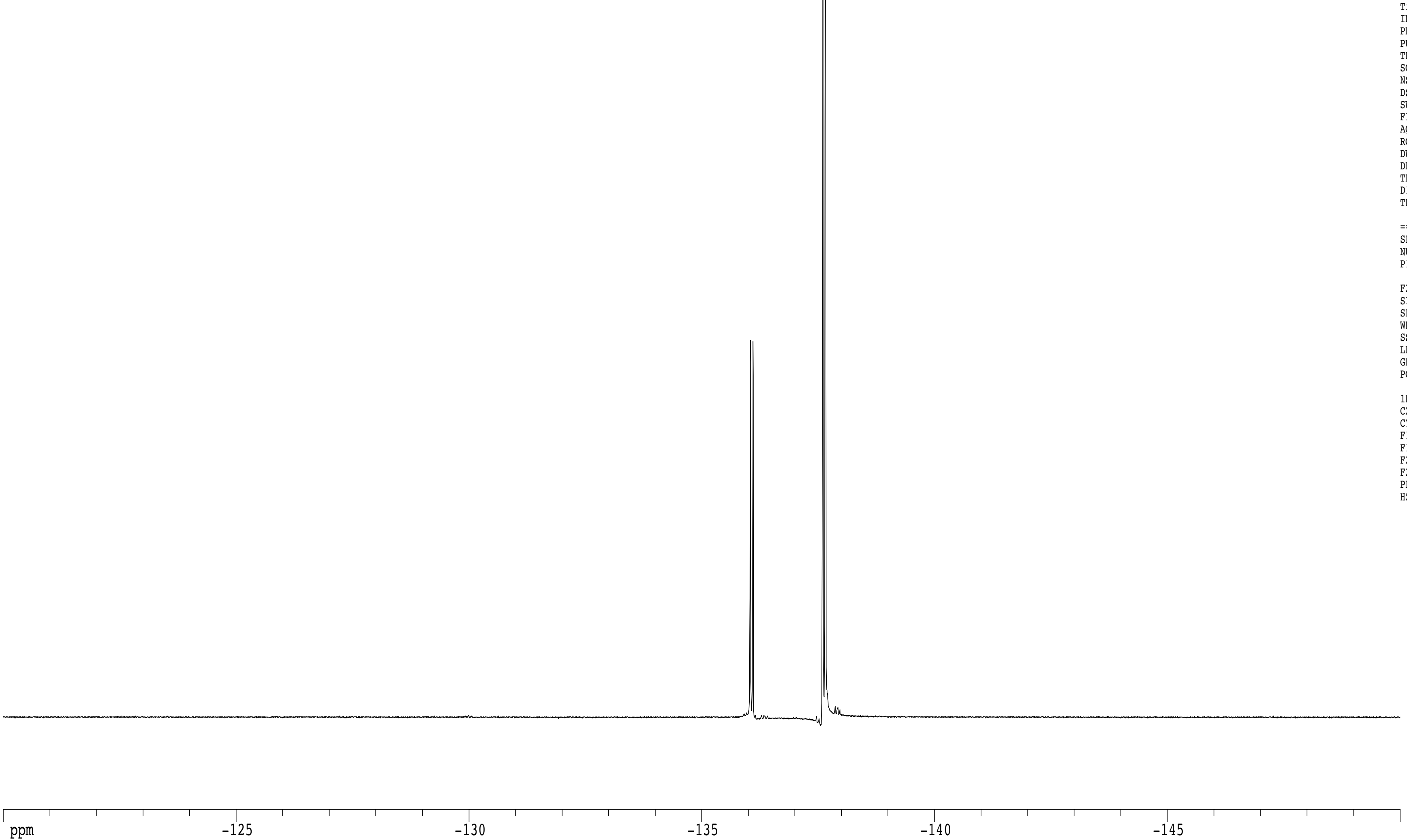


Current Data Parameters
USER mcginnit
NAME tmm-3-125-char
EXPNO 3
PROCNO 1

F2 - Acquisition Parameters
Date_ 20210514
Time 14.25
INSTRUM av600
PROBHD 5 mm CPBBO BB-
PULPROG zgflqn30
TD 131072
SOLVENT CDCl3
NS 16
DS 2
SWH 178571.422 Hz
FIDRES 1.362392 Hz
AQ 0.3670516 sec
RG 575
DW 2.800 usec
DE 18.00 usec
TE 298.0 K
D1 3.00000000 sec
TD0 1

======== CHANNEL f1 ========
SFO1 564.6299196 MHz
NUC1 19F
P1 18.25 usec

F2 - Processing parameters
SI 131072
SF 564.6863858 MHz
WDW no
SSB 0
LB 0.00 Hz
GB 0
PC 1.00

1D NMR plot parameters
CX 22.80 cm
CY 15.00 cm
F1P -120.000 ppm
F1 -67762.37 Hz
F2P -150.000 ppm
F2 -84702.96 Hz
PPMCM 1.31579 ppm/cm
HZCM 743.00842 Hz/cm

**HPLC Chiracel OJ, 1% IPA/hexanes, 2.0 mL/min**

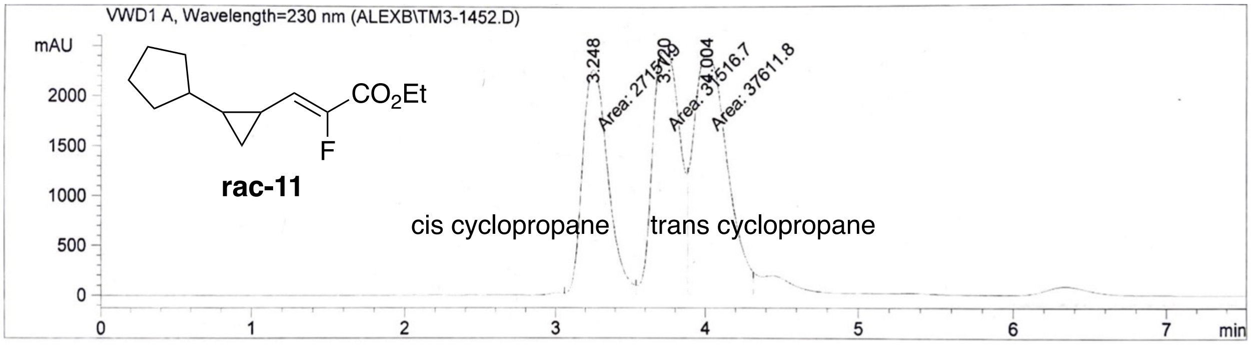


Signal 2: VWD1 A, Wavelength=230 nm

| Peak # | RetTime [min] | Type | Width [min] | Area mAU *s | Height [mAU] | Area % |
|---|---|---|---|---|---|---|
| 1 | 3.248 | MF | 0.1994 | 2.71519e4 | 2269.00171 | 28.2009 |
| 2 | 3.720 | MF | 0.2131 | 3.15167e4 | 2464.63843 | 32.7343 |
| 3 | 4.004 | MF | 0.2592 | 3.76118e4 | 2418.35547 | 39.0648 |
| Totals : | | | | 9.62803e4 | 7151.99561 | |

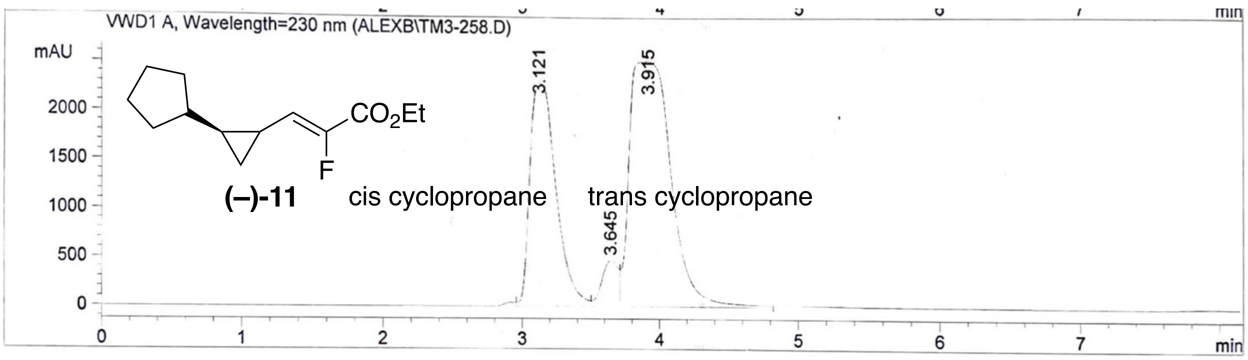


Signal 2: VWD1 A, Wavelength=230 nm

| Peak # | RetTime [min] | Type | Width [min] | Area mAU *s | Height [mAU] | Area % |
|---|---|---|---|---|---|---|
| 1 | 3.121 | VV | 0.2008 | 3.06097e4 | 2316.11401 | 34.7471 |
| 2 | 3.645 | VV | 0.1295 | 3982.29883 | 476.46460 | 4.5206 |
| 3 | 3.915 | VV | 0.2921 | 5.35008e4 | 2542.67187 | 60.7323 |
| Totals : | | | | 8.80928e4 | 5335.25049 | |

1H spectrum

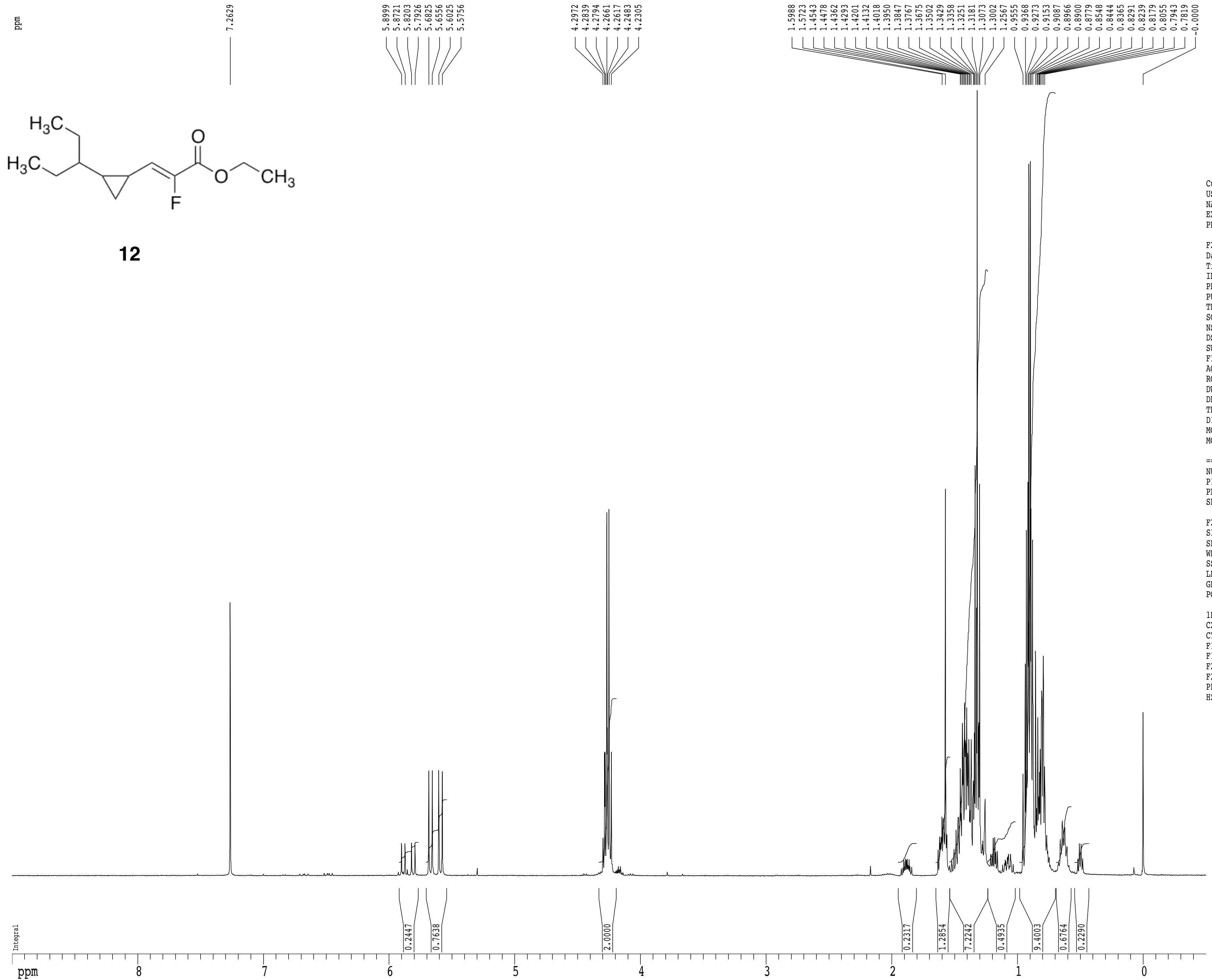


Current Data Parameters
USER linpc2
NAME pcl-2-030
EXPNO 3
PROCNO 1

F2 - Acquisition Parameters
Date_ 20210517
Time 10.13
INSTRUM drx400
PROBHD 5 mm QNP H/F/P
PULPROG zg30
TD 65536
SOLVENT CDCl3
NS 8
DS 2
SWH 6410.256 Hz
FIDRES 0.097813 Hz
AQ 5.1118579 sec
RG 256
DW 78.000 usec
DE 4.50 usec
TE 298.0 K
D1 0.10000000 sec
MCREST 0.00000000 sec
MCWRK 0.01500000 sec

======== CHANNEL f1 ========
NUC1 1H
P1 12.00 usec
PL1 -1.60 dB
SFO1 400.1328009 MHz

F2 - Processing parameters
SI 65536
SF 400.1300205 MHz
WDW EM
SSB 0
LB 0.30 Hz
GB 0
PC 2.00

1D NMR plot parameters
CX 22.80 cm
CY 15.00 cm
F1P 9.000 ppm
F1 3601.17 Hz
F2P -0.500 ppm
F2 -200.06 Hz
PPMCM 0.41667 ppm/cm
HZCM 166.72086 Hz/cm

13C spectrum with 1H decoupling

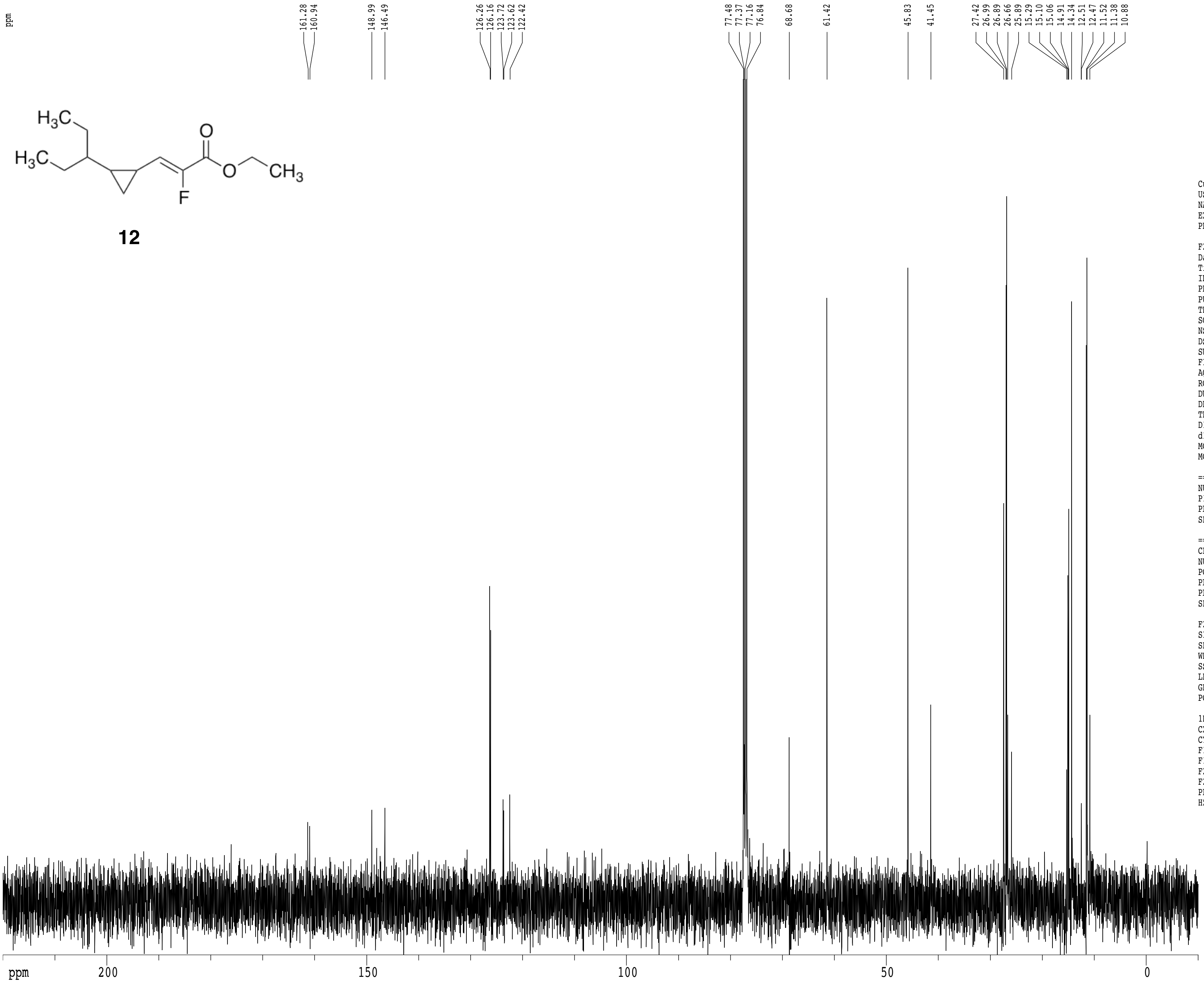

**12**



Current Data Parameters
USER linpc2
NAME pcl-2-030
EXPNO 4
PROCNO 1

F2 - Acquisition Parameters
Date_ 20210517
Time 10.15
INSTRUM drx400
PROBHD 5 mm QNP H/F/P
PULPROG zgdc30
TD 65536
SOLVENT CDCl3
NS 376
DS 4
SWH 24154.590 Hz
FIDRES 0.368570 Hz
AQ 1.3566452 sec
RG 9195.2
DW 20.700 usec
DE 20.39 usec
TE 298.0 K
D1 0.10000000 sec
d11 0.03000000 sec
MCREST 0.00000000 sec
MCWRK 0.01500000 sec

======== CHANNEL f1 ========
NUC1 13C
P1 8.30 usec
PL1 -3.00 dB
SFO1 100.6237964 MHz

======== CHANNEL f2 ========
CPDPRG2 waltz16
NUC2 1H
PCPD2 90.00 usec
PL2 -1.60 dB
PL12 16.50 dB
SFO2 400.1328009 MHz

F2 - Processing parameters
SI 65536
SF 100.6127565 MHz
WDW EM
SSB 0
LB 1.00 Hz
GB 0
PC 1.00

1D NMR plot parameters
CX 22.80 cm
CY 50.00 cm
F1P 220.000 ppm
F1 22134.81 Hz
F2P -10.000 ppm
F2 -1006.13 Hz
PPMCM 10.08772 ppm/cm
HZCM 1014.95325 Hz/cm

19F spectrum

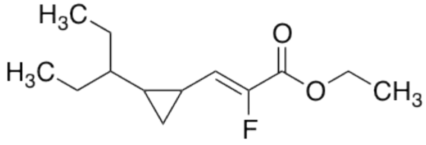


**12**

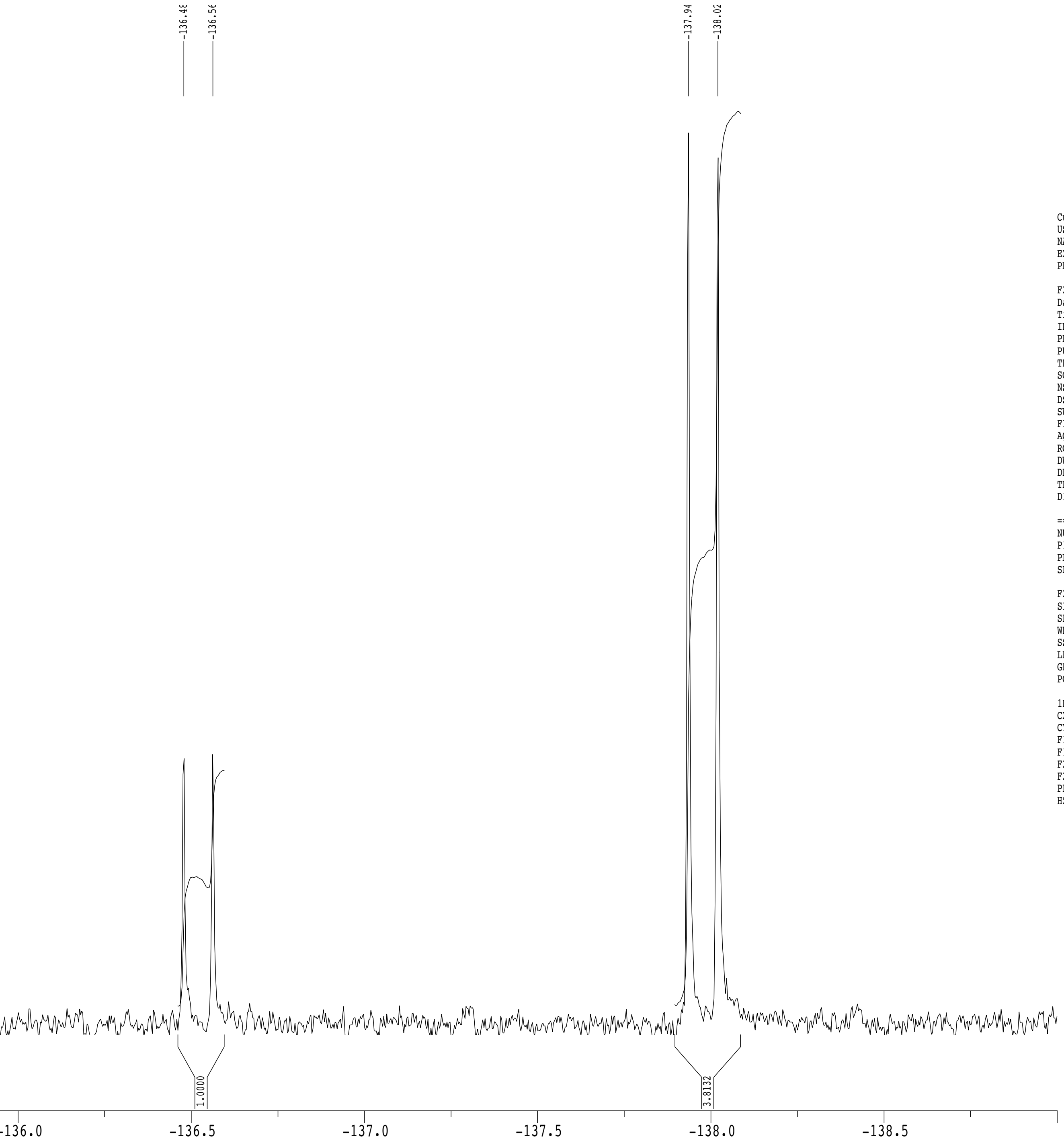


```
Current Data Parameters
USER              linpc2
NAME          pcl-2-030
EXPNO                 5
PROCNO                1

F2 - Acquisition Parameters
Date_          20210517
Time              10.25
INSTRUM          drx400
PROBHD   5 mm QNP H/F/P
PULPROG        zgflqn30
TD                65536
SOLVENT           CDCl3
NS                   16
DS                    2
SWH           75187.969 Hz
FIDRES         1.147277 Hz
AQ            0.4358644 sec
RG                 6502
DW                6.650 usec
DE                 9.46 usec
TE                298.0 K
D1           2.00000000 sec

======== CHANNEL f1 ========
NUC1                19F
P1                11.75 usec
PL1               -6.00 dB
SFO1        376.4646491 MHz

F2 - Processing parameters
SI                65536
SF          376.4984640 MHz
WDW                  EM
SSB                   0
LB                 1.00 Hz
GB                    0
PC                 1.00

1D NMR plot parameters
CX                22.80 cm
CY                15.00 cm
F1P            -135.000 ppm
F1            -50827.29 Hz
F2P            -139.000 ppm
F2            -52333.29 Hz
PPMCM           0.17544 ppm/cm
HZCM           66.05237 Hz/cm
```

1H spectrum

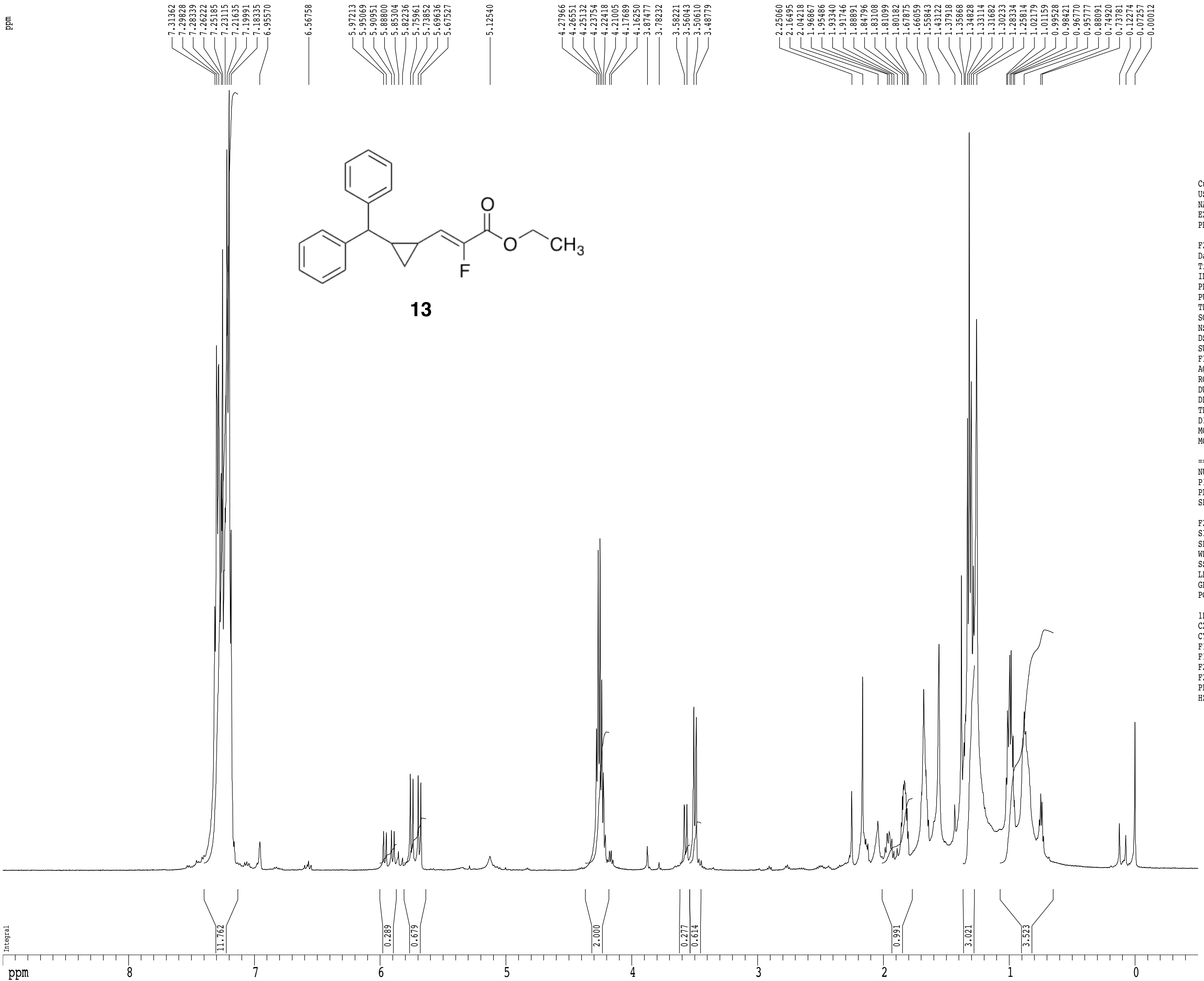


| | |
|---|---|
| Current Data Parameters | |
| USER | linpc2 |
| NAME | pcl-2-063 |
| EXPNO | 5 |
| PROCNO | 1 |
| F2 - Acquisition Parameters | |
| Date_ | 20210609 |
| Time | 15.43 |
| INSTRUM | cryo500 |
| PROBHD | 5 mm CPTCI 1H- |
| PULPROG | zg30 |
| TD | 81728 |
| SOLVENT | CDCl3 |
| NS | 8 |
| DS | 2 |
| SWH | 8012.820 Hz |
| FIDRES | 0.098043 Hz |
| AQ | 5.0998774 sec |
| RG | 5.7 |
| DW | 62.400 usec |
| DE | 6.00 usec |
| TE | 298.0 K |
| D1 | 0.10000000 sec |
| MCREST | 0.00000000 sec |
| MCWRK | 0.01500000 sec |
| ======== CHANNEL f1 ======== | |
| NUC1 | 1H |
| P1 | 9.75 usec |
| PL1 | 1.60 dB |
| SFO1 | 500.2235015 MHz |
| F2 - Processing parameters | |
| SI | 65536 |
| SF | 500.2200347 MHz |
| WDW | EM |
| SSB | 0 |
| LB | 0.30 Hz |
| GB | 0 |
| PC | 1.00 |
| 1D NMR plot parameters | |
| CX | 22.80 cm |
| CY | 15.00 cm |
| F1P | 9.000 ppm |
| F1 | 4501.98 Hz |
| F2P | -0.500 ppm |
| F2 | -250.11 Hz |
| PPMCM | 0.41667 ppm/cm |
| HZCM | 208.42502 Hz/cm |

Z-restored spin-echo 13C spectrum with 1H decoupling

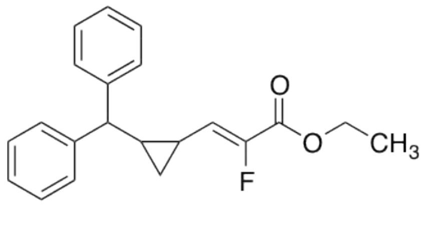

**13**

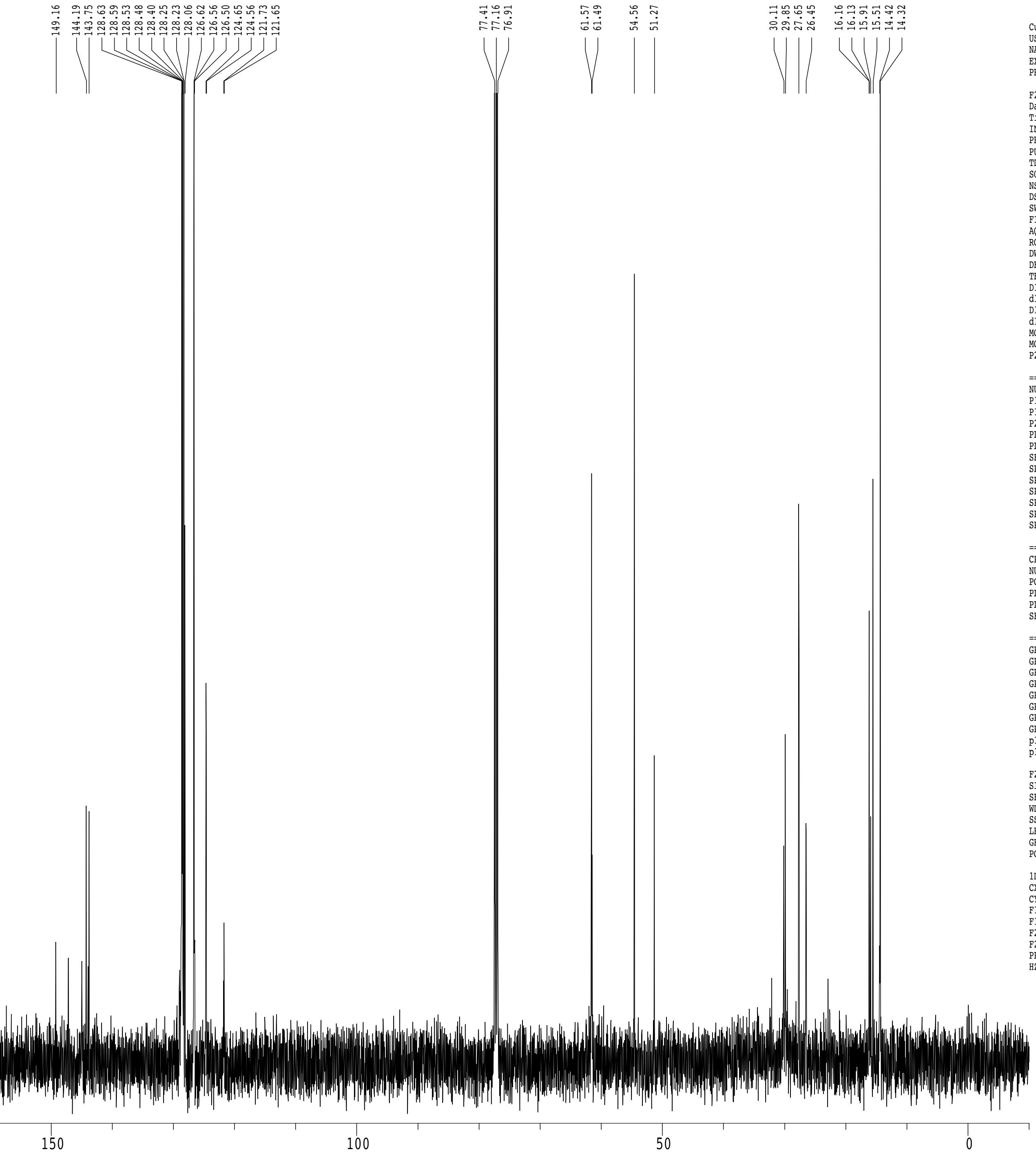


Current Data Parameters
USER linpc2
NAME pcl-2-063
EXPNO 6
PROCNO 1

F2 - Acquisition Parameters
Date_ 20210609
Time 15.47
INSTRUM cryo500
PROBHD 5 mm CPTCI 1H-
PULPROG SpinEchopg30gp2.prd
TD 65536
SOLVENT CDCl3
NS 184
DS 16
SWH 30303.031 Hz
FIDRES 0.462388 Hz
AQ 1.0813940 sec
RG 7298.2
DW 16.500 usec
DE 6.00 usec
TE 298.0 K
D1 0.25000000 sec
d11 0.03000000 sec
D16 0.00020000 sec
d17 0.00019600 sec
MCREST 0.00000000 sec
MCWRK 0.01500000 sec
P2 37.70 usec

======== CHANNEL f1 ========
NUC1 13C
P1 18.85 usec
P12 2000.00 usec
P20 500.00 usec
PL0 120.00 dB
PL1 -1.00 dB
SFO1 125.7942548 MHz
SP2 1.55 dB
SP4 1.55 dB
SPNAM2 Crp60comp.4
SPNAM4 Crp60,0.5,20.1
SPOFF2 0.00 Hz
SPOFF4 0.00 Hz

======== CHANNEL f2 ========
CPDPRG2 waltz16
NUC2 1H
PCPD2 100.00 usec
PL2 1.60 dB
PL12 22.00 dB
SFO2 500.2225011 MHz

====== GRADIENT CHANNEL =====
GPNAM1 SINE.100
GPNAM2 SINE.100
GPX1 0.00 %
GPX2 0.00 %
GPY1 0.00 %
GPY2 0.00 %
GPZ1 30.00 %
GPZ2 50.00 %
p15 500.00 usec
p16 1000.00 usec

F2 - Processing parameters
SI 65536
SF 125.7804076 MHz
WDW EM
SSB 0
LB 1.00 Hz
GB 0
PC 2.00

1D NMR plot parameters
CX 22.80 cm
CY 30.00 cm
F1P 220.000 ppm
F1 27671.69 Hz
F2P -10.000 ppm
F2 -1257.80 Hz
PPMCM 10.08772 ppm/cm
HZCM 1268.83752 Hz/cm

19F spectrum

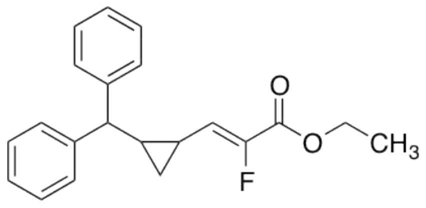

**13**

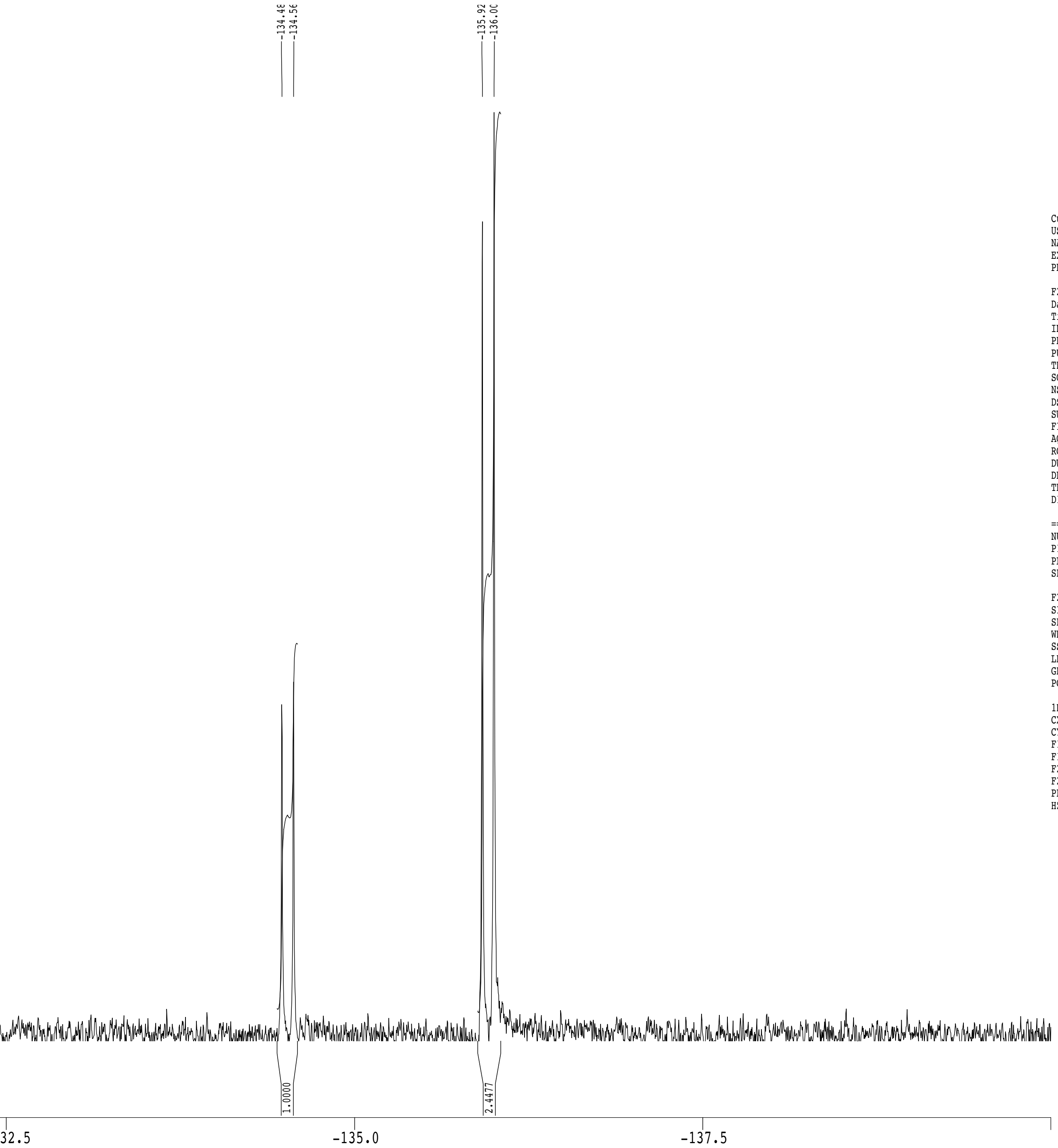


Current Data Parameters
USER linpc2
NAME pcl-2-063
EXPNO 4
PROCNO 1

F2 - Acquisition Parameters
Date_ 20210608
Time 13.47
INSTRUM drx400
PROBHD 5 mm QNP H/F/P
PULPROG zgflqn30
TD 65536
SOLVENT CDCl3
NS 32
DS 2
SWH 75187.969 Hz
FIDRES 1.147277 Hz
AQ 0.4358644 sec
RG 4597.6
DW 6.650 usec
DE 9.46 usec
TE 298.0 K
D1 2.00000000 sec

======== CHANNEL f1 ========
NUC1 19F
P1 11.75 usec
PL1 -6.00 dB
SFO1 376.4646491 MHz

F2 - Processing parameters
SI 65536
SF 376.4984640 MHz
WDW EM
SSB 0
LB 1.00 Hz
GB 0
PC 1.00

1D NMR plot parameters
CX 22.80 cm
CY 15.00 cm
F1P -130.000 ppm
F1 -48944.80 Hz
F2P -140.000 ppm
F2 -52709.79 Hz
PPMCM 0.43860 ppm/cm
HZCM 165.13092 Hz/cm

| Developmental Therapeutics Program | NSC: D-833092 / 1 | Conc: 1.00E-5 Molar | Test Date: Jan 24, 2022 |
|---|---|---|---|
| One Dose Mean Graph | Experiment ID: 2201OS51 | | Report Date: Jul 20, 2022 |

| Panel/Cell Line | Growth Percent For cyclopropane 13 |
|---|---|
| Leukemia | |
| CCRF-CEM | 85.38 |
| HL-60(TB) | -7.32 |
| K-562 | 70.43 |
| MOLT-4 | 76.75 |
| RPMI-8226 | 69.53 |
| SR | 63.32 |
| Non-Small Cell Lung Cancer | |
| A549/ATCC | 91.67 |
| EKVX | 86.48 |
| HOP-62 | 124.58 |
| HOP-92 | 84.13 |
| NCI-H226 | 91.77 |
| NCI-H23 | 73.76 |
| NCI-H322M | 80.60 |
| NCI-H460 | 38.65 |
| NCI-H522 | 94.82 |
| Colon Cancer | |
| COLO 205 | 116.89 |
| HCC-2998 | 102.46 |
| HCT-116 | 61.98 |
| HCT-15 | 66.98 |
| HT29 | 80.59 |
| KM12 | 83.46 |
| SW-620 | 75.09 |
| CNS Cancer | |
| SF-268 | 99.06 |
| SF-295 | 88.70 |
| SF-539 | 96.54 |
| SNB-19 | 95.86 |
| SNB-75 | 90.42 |
| U251 | 108.69 |
| Melanoma | |
| LOX IMVI | 84.93 |
| MALME-3M | 81.22 |
| M14 | 91.74 |
| MDA-MB-435 | 84.77 |
| SK-MEL-2 | 86.31 |
| SK-MEL-28 | 102.95 |
| SK-MEL-5 | 88.88 |
| UACC-257 | 104.60 |
| UACC-62 | 65.11 |
| Ovarian Cancer | |
| IGROV1 | 90.83 |
| OVCAR-3 | 103.00 |
| OVCAR-4 | 87.31 |
| OVCAR-5 | 102.42 |
| OVCAR-8 | 105.27 |
| NCI/ADR-RES | 89.38 |
| SK-OV-3 | 148.40 |
| Renal Cancer | |
| 786-0 | 93.01 |
| A498 | 87.93 |
| ACHN | 98.88 |
| CAKI-1 | 65.58 |
| RXF 393 | 77.23 |
| SN12C | 98.78 |
| TK-10 | 101.50 |
| UO-31 | 70.24 |
| Prostate Cancer | |
| PC-3 | 78.82 |
| DU-145 | 105.58 |
| Breast Cancer | |
| MCF7 | 80.68 |
| MDA-MB-231/ATCC | 88.40 |
| HS 578T | 81.27 |
| BT-549 | 116.61 |
| T-47D | 70.68 |
| MDA-MB-468 | 36.18 |
| Mean | 86.00 |
| Delta | 93.32 |
| Range | 155.72 |

Mean Growth Percent - Growth Percent

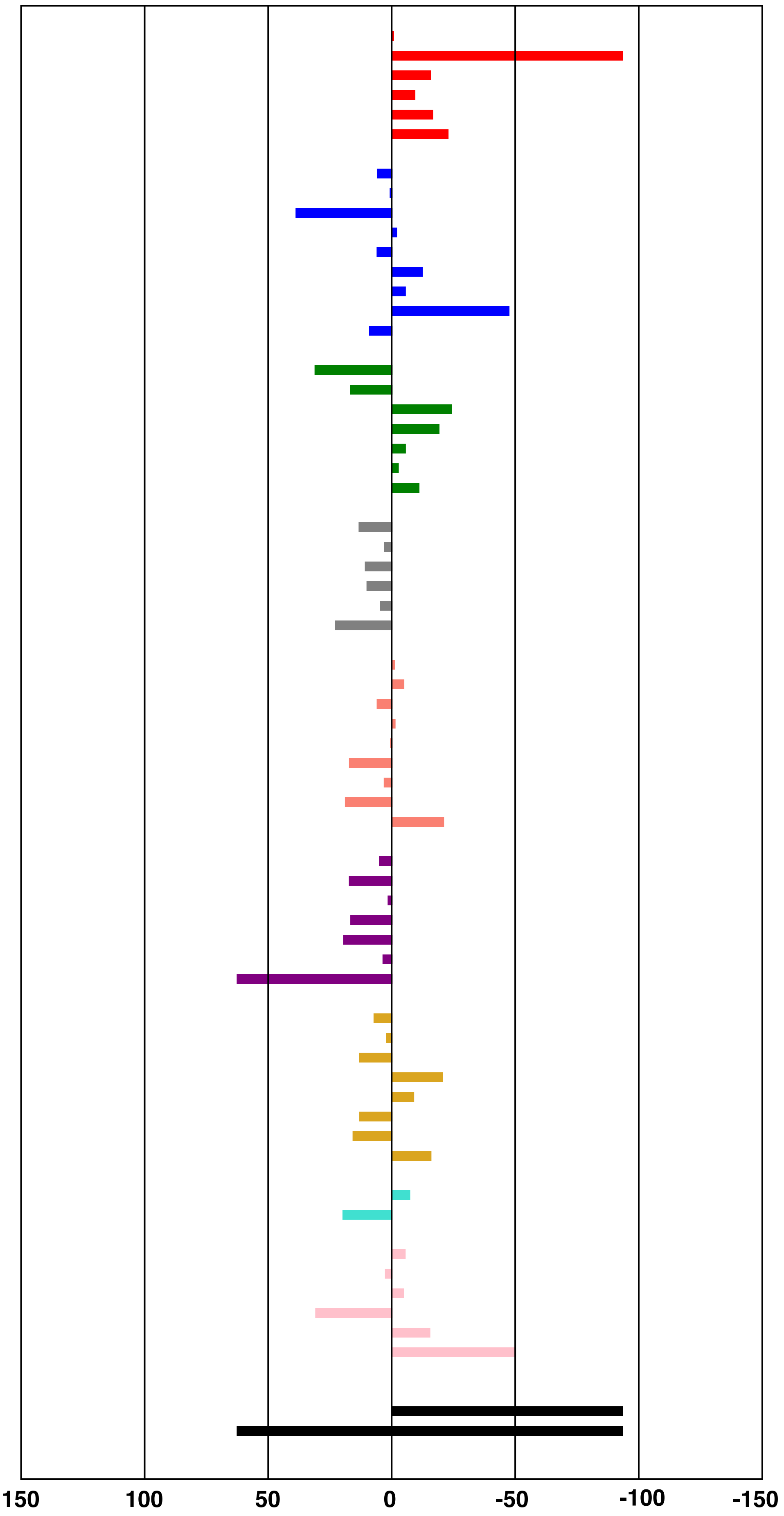

1H spectrum

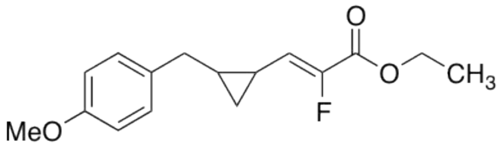


**14**

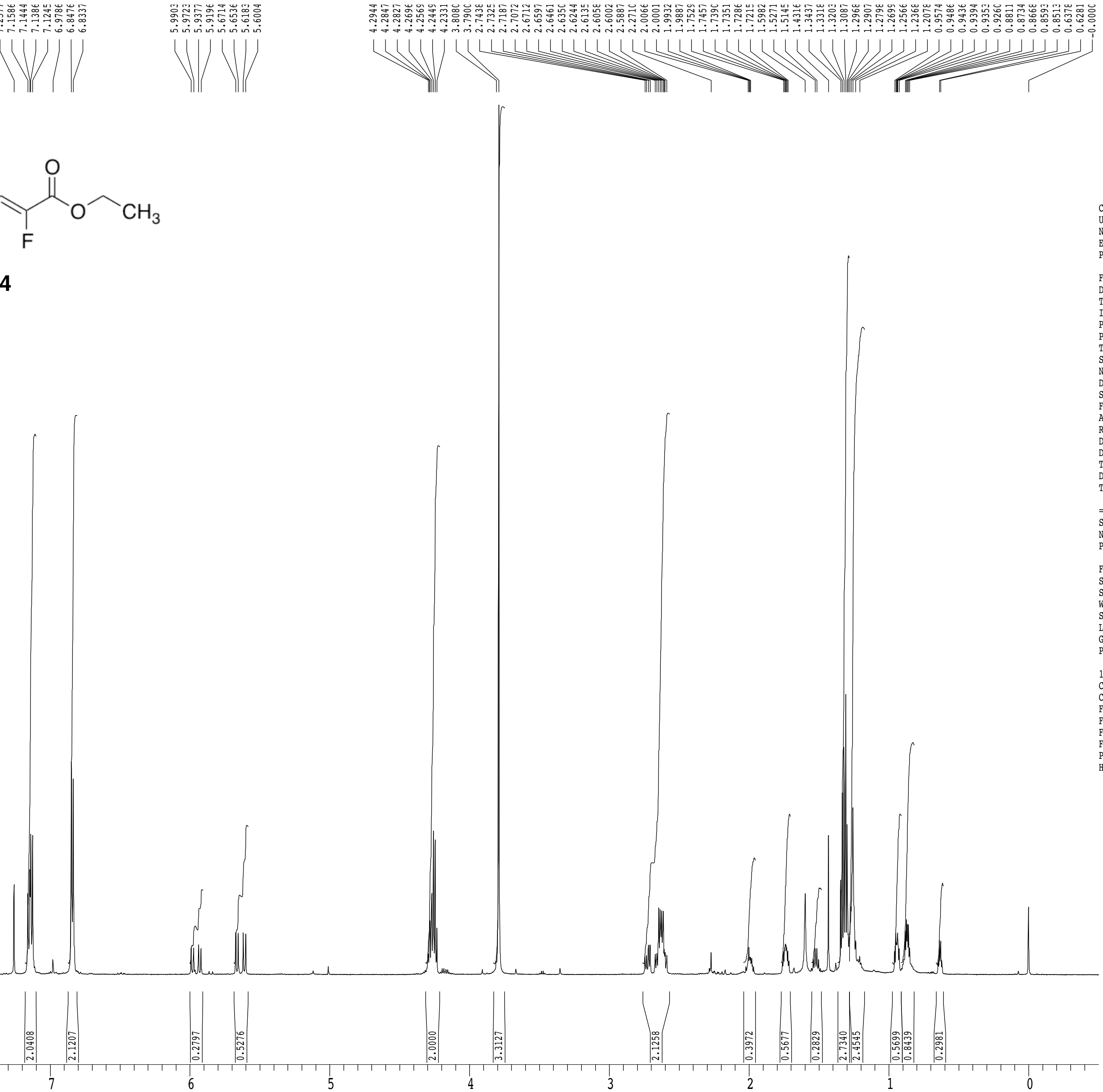


Current Data Parameters
USER linpc2
NAME pcl-2-165
EXPNO 2
PROCNO 1

F2 - Acquisition Parameters
Date_ 20210906
Time 11.27
INSTRUM av600
PROBHD 5 mm CPBBO BB-
PULPROG zg30
TD 98074
SOLVENT CDCl3T
NS 8
DS 2
SWH 9615.385 Hz
FIDRES 0.098042 Hz
AQ 5.0998979 sec
RG 10
DW 52.000 usec
DE 14.23 usec
TE 298.0 K
D1 0.10000000 sec
TD0 1

======== CHANNEL f1 ========
SFO1 600.1342009 MHz
NUC1 1H
P1 9.50 usec

F2 - Processing parameters
SI 65536
SF 600.1300367 MHz
WDW no
SSB 0
LB 0.00 Hz
GB 0
PC 1.00

1D NMR plot parameters
CX 22.80 cm
CY 15.00 cm
F1P 9.000 ppm
F1 5401.17 Hz
F2P -0.500 ppm
F2 -300.06 Hz
PPMCM 0.41667 ppm/cm
HZCM 250.05420 Hz/cm

13C spectrum

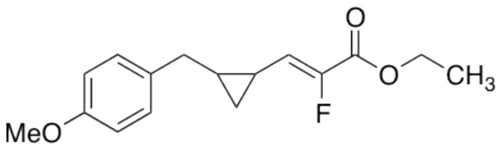

**14**

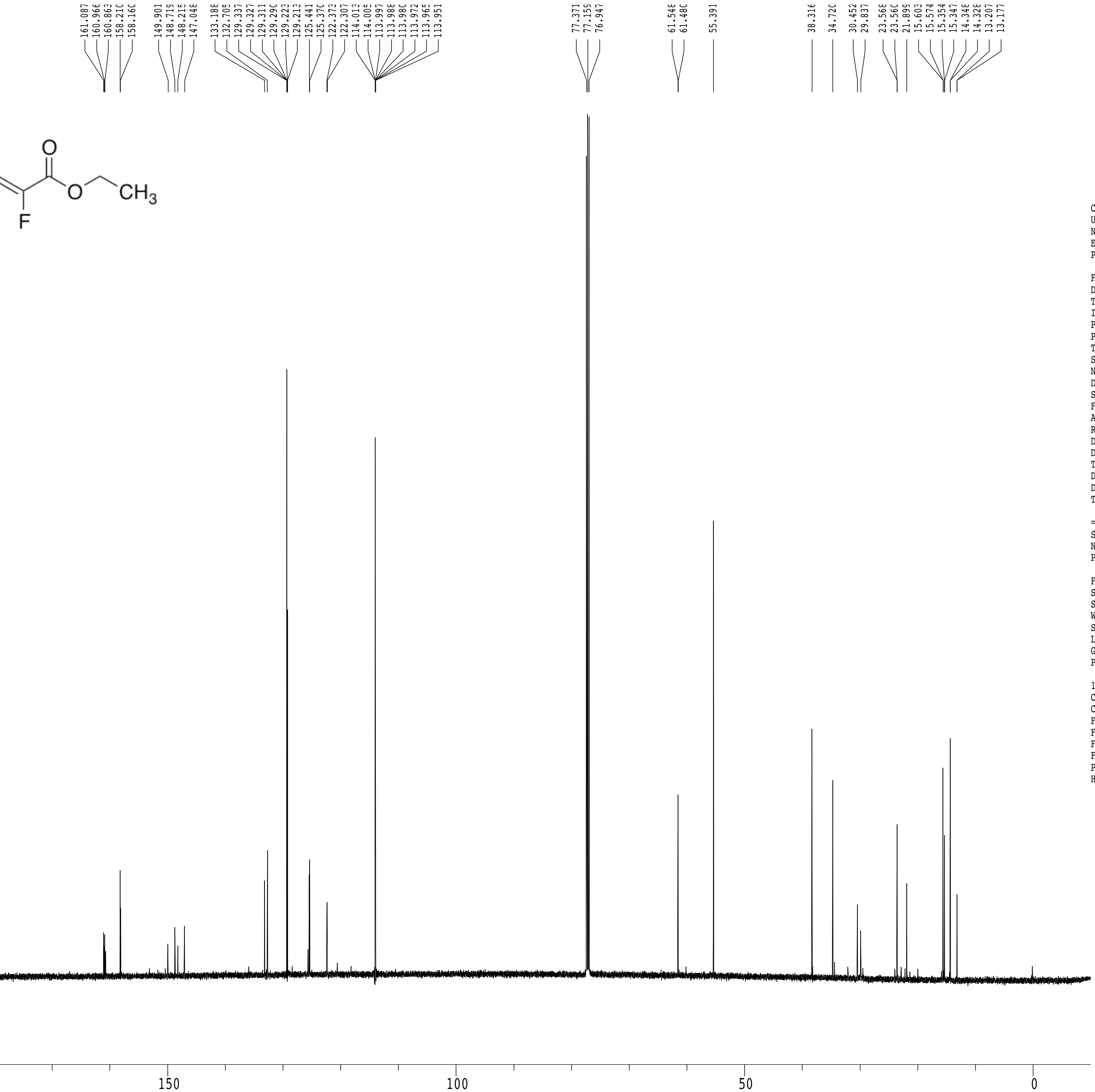


Current Data Parameters
USER linpc2
NAME pcl-2-165
EXPNO 4
PROCNO 1

F2 - Acquisition Parameters
Date_ 20210906
Time 11.41
INSTRUM av600
PROBHD 5 mm CPBBO BB-
PULPROG zgdc30
TD 65536
SOLVENT CDCl3T
NS 351
DS 4
SWH 36231.883 Hz
FIDRES 0.552855 Hz
AQ 0.9044468 sec
RG 2050
DW 13.800 usec
DE 19.63 usec
TE 298.0 K
D1 0.40000001 sec
D11 0.03000000 sec
TD0 1

======== CHANNEL f1 ========
SFO1 150.9194080 MHz
NUC1 13C
P1 10.10 usec

F2 - Processing parameters
SI 65536
SF 150.9027958 MHz
WDW no
SSB 0
LB 0.00 Hz
GB 0
PC 1.00

1D NMR plot parameters
CX 22.80 cm
CY 15.00 cm
F1P 220.000 ppm
F1 33198.62 Hz
F2P -10.000 ppm
F2 -1509.03 Hz
PPMCM 10.08772 ppm/cm
HZCM 1522.26514 Hz/cm

19F spectrum

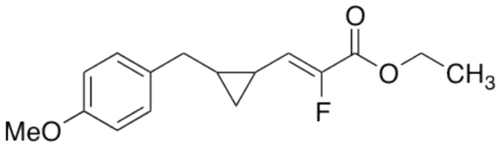


**14**

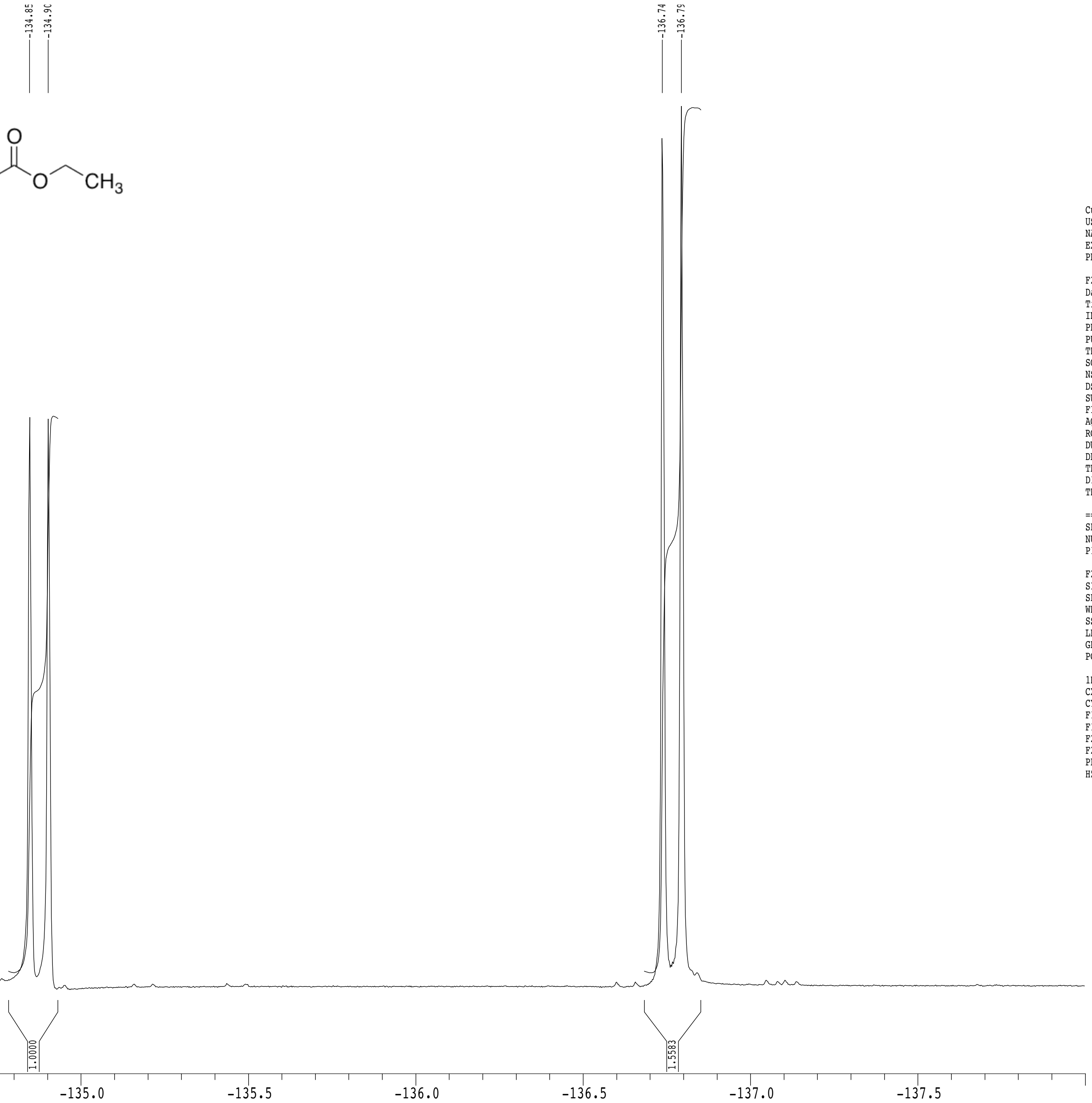


```
Current Data Parameters
USER              linpc2
NAME           pcl-2-165
EXPNO                  3
PROCNO                 1

F2 - Acquisition Parameters
Date_           20210906
Time               11.33
INSTRUM            av600
PROBHD   5 mm CPBBO BB-
PULPROG        zgflqn30
TD                131072
SOLVENT           CDCl3T
NS                    16
DS                     2
SWH          178571.422 Hz
FIDRES         1.362392 Hz
AQ            0.3670516 sec
RG                   575
DW                 2.800 usec
DE                 18.00 usec
TE                 298.0 K
D1            3.00000000 sec
TD0                    1

======== CHANNEL f1 ========
SFO1        564.6299196 MHz
NUC1                 19F
P1                 18.25 usec

F2 - Processing parameters
SI                131072
SF           564.6864227 MHz
WDW                   no
SSB                    0
LB                  0.00 Hz
GB                     0
PC                  1.00

1D NMR plot parameters
CX                 22.80 cm
CY                 15.00 cm
F1P             -134.000 ppm
F1             -75667.98 Hz
F2P             -138.000 ppm
F2             -77926.73 Hz
PPMCM            0.17544 ppm/cm
HZCM            99.06779 Hz/cm
```

gcosy60

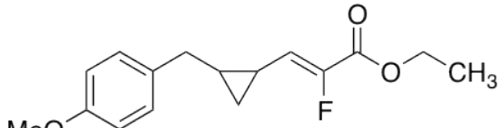


**14**

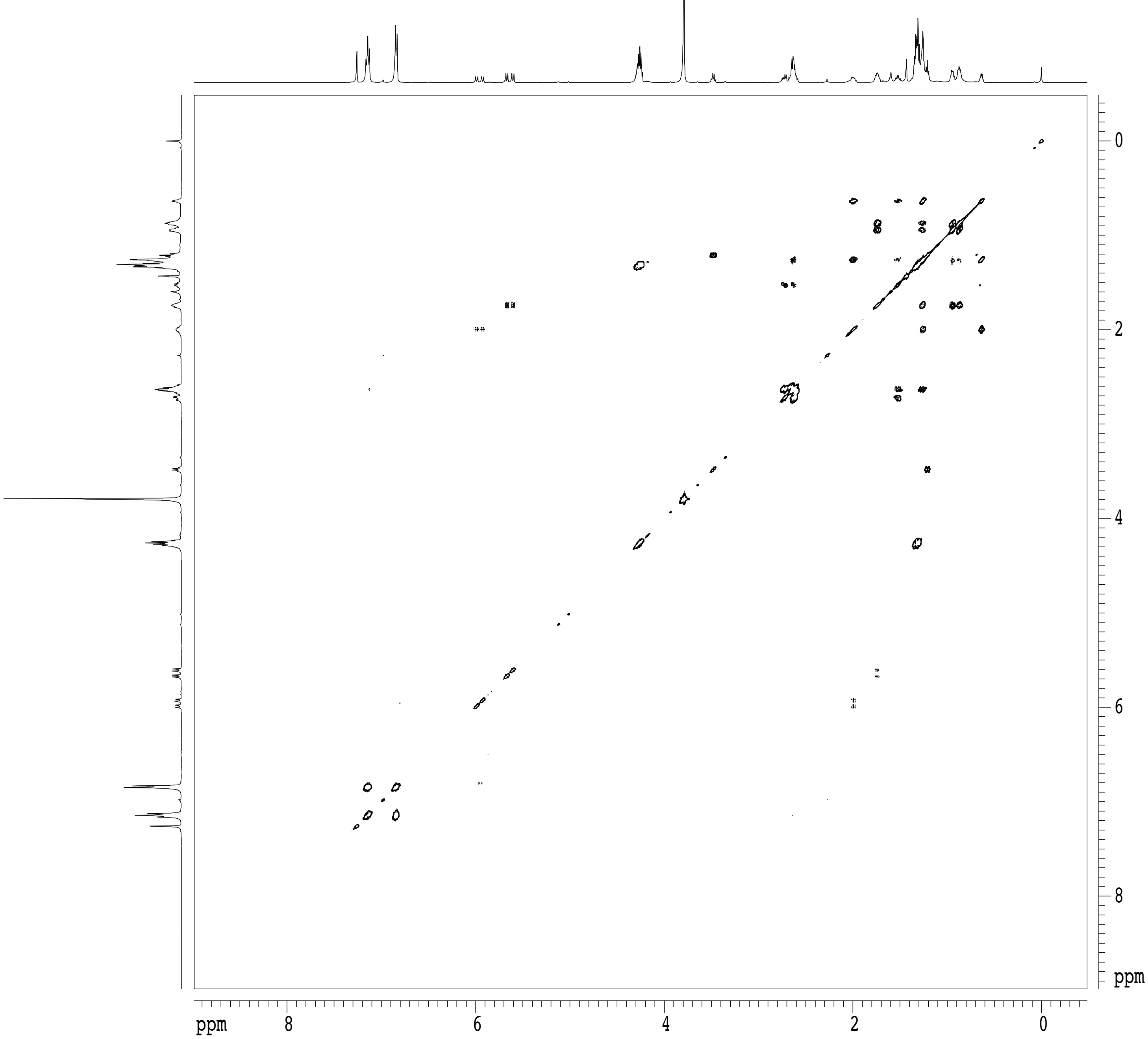


Current Data Parameters
USER linpc2
NAME pcl-2-165-cosy
EXPNO 2
PROCNO 1

F2 - Acquisition Parameters
Date_ 20211029
Time 13.54
INSTRUM cryo500
PROBHD 5 mm CPTCI 1H-
PULPROG cosygp60.prd
TD 2048
SOLVENT CDCl3
NS 1
DS 16
SWH 4734.849 Hz
FIDRES 2.311938 Hz
AQ 0.2163188 sec
RG 143.7
DW 105.600 usec
DE 6.00 usec
TE 298.0 K
d0 0.00000300 sec
D1 1.00000000 sec
d13 0.00000300 sec
D16 0.00020000 sec
IN0 0.00021120 sec

======== CHANNEL f1 ========
NUC1 1H
P1 9.75 usec
PL1 1.60 dB
SFO1 500.2221573 MHz

====== GRADIENT CHANNEL =====
GPNAM1 SMSQ10.100
GPNAM2 SMSQ10.100
GPX1 0.00 %
GPX2 0.00 %
GPY1 0.00 %
GPY2 0.00 %
GPZ1 17.00 %
GPZ2 17.00 %
P16 1000.00 usec

F1 - Acquisition parameters
ND0 1
TD 512
SFO1 500.2222 MHz
FIDRES 9.247751 Hz
SW 9.465 ppm
FnMODE QF

F2 - Processing parameters
SI 1024
SF 500.2200314 MHz
WDW SINE
SSB 0
LB 0.00 Hz
GB 0
PC 1.00

F1 - Processing parameters
SI 1024
MC2 QF
SF 500.2200314 MHz
WDW SINE
SSB 0
LB 0.00 Hz
GB 0

2D NMR plot parameters
CX2 15.00 cm
CX1 15.00 cm
F2PLO 8.983 ppm
F2LO 4493.36 Hz
F2PHI -0.483 ppm
F2HI -241.49 Hz
F1PLO 8.983 ppm
F1LO 4493.36 Hz
F1PHI -0.483 ppm
F1HI [illegible]
F2PPMCM 0.63104 ppm/cm
F2HZCM 315.65659 Hz/cm
F1PPMCM 0.63104 ppm/cm
F1HZCM 315.65659 Hz/cm

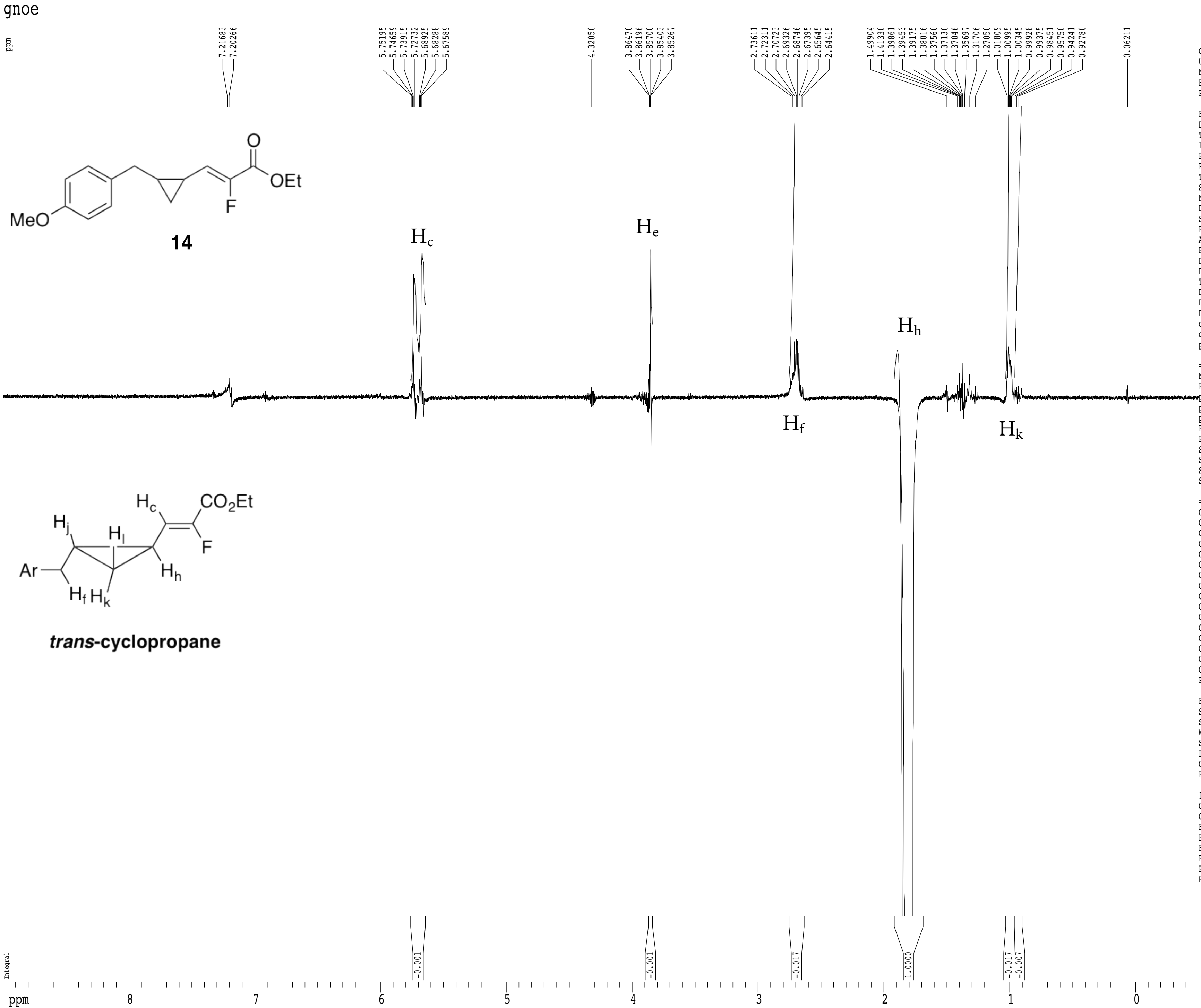


Current Data Parameters
USER linpc2
NAME pcl-2-165-noe
EXPNO 3
PROCNO 1

F2 - Acquisition Parameters
Date_ 20211030
Time 11.16
INSTRUM cryo500
PROBHD 5 mm CPTCI 1H-
PULPROG gnoe1cc22.prd
TD 65536
SOLVENT CDCl3
NS 128
DS 8
SWH 8012.820 Hz
FIDRES 0.122266 Hz
AQ 4.0894966 sec
RG 161.3
DW 62.400 usec
DE 6.00 usec
TE 298.0 K
D1 1.00000000 sec
D8 0.50000000 sec
D16 0.00020000 sec
d21 0.33375451 sec
d22 0.16399699 sec
p2 19.50 usec

======== CHANNEL f1 ========
NUC1 1H
P1 9.75 usec
p3 29.25 usec
p4 39.00 usec
p5 26.00 usec
P29 40000.00 usec
PL1 1.60 dB
SFO1 500.2209016 MHz
SP9 60.00 dB
SPNAM9 gauss1.512
SPOFF9 0.00 Hz

====== GRADIENT CHANNEL =====
GPNAM1 SMSQ10.100
GPNAM2 SMSQ10.100
GPNAM3 SMSQ10.100
GPNAM4 SMSQ10.100
GPX1 0.00 %
GPX2 0.00 %
GPX3 0.00 %
GPX4 0.00 %
GPY1 0.00 %
GPY2 0.00 %
GPY3 0.00 %
GPY4 0.00 %
GPZ1 7.00 %
GPZ2 3.00 %
GPZ3 2.30 %
GPZ4 -2.30 %
P16 1000.00 usec

F2 - Processing parameters
SI 65536
SF 500.2200000 MHz
WDW no
SSB 0
LB 0.00 Hz
GB 0
PC 1.00

1D NMR plot parameters
CX 22.80 cm
CY 50.00 cm
F1P 9.000 ppm
F1 4501.98 Hz
F2P -0.500 ppm
F2 -250.11 Hz
PPMCM 0.41667 ppm/cm
HZCM 208.42500 Hz/cm

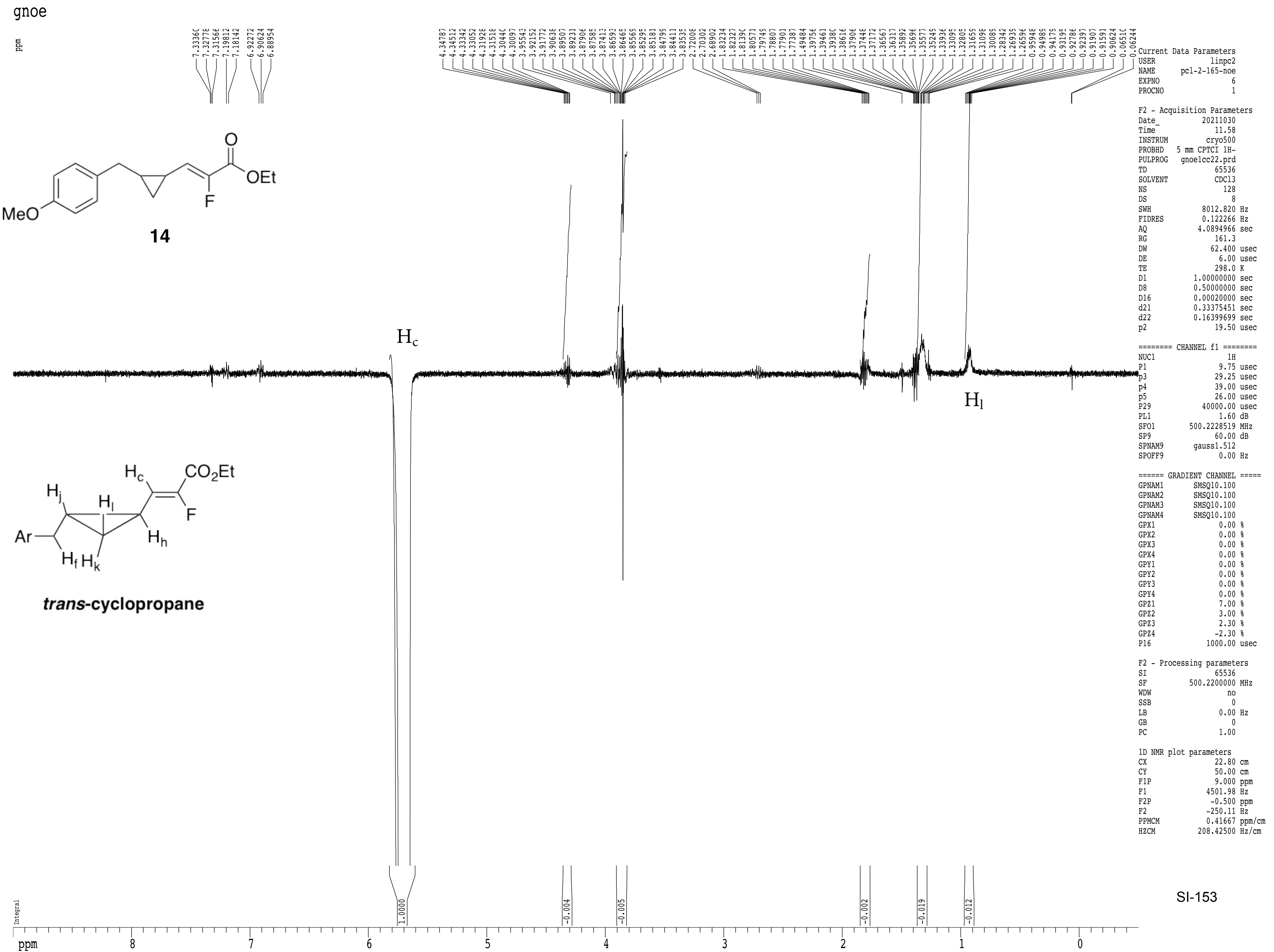
gnoe
ppm
7.3336C 7.32778 7.31566 7.19812 7.18142 6.92272 6.90624 6.88954
4.34787 4.34512 4.33342 4.33052 4.31928 4.31518 4.30440 4.30097 3.95543 3.92152 3.91772 3.90638 3.89507 3.89231 3.87906 3.87585 3.87413 3.86593 3.86465 3.85569 3.85295 3.85181 3.84799 3.84411 3.83535 2.72008 2.70302 2.68902 1.83234 1.82327 1.81390 1.80571 1.79749 1.78807 1.77901 1.77387 1.49484 1.39756 1.39461 1.39380 1.38616 1.37906 1.37448 1.37172 1.36567 1.36317 1.35892 1.35696 1.35577 1.35249 1.33936 1.33095 1.32805 1.31659 1.31098 1.30089 1.28342 1.26939 1.26596 0.95948 0.94989 0.94179 0.93195 0.92786 0.92397 0.91907 0.91591 0.90624 0.06510 0.06244
O
OEt
F
MeO
14
Hc
CO2Et
Hj
Hl
F
Ar
Hh
Hf Hk
trans-cyclopropane
Hc
Hl
Current Data Parameters
USER linpc2
NAME pcl-2-165-noe
EXPNO 6
PROCNO 1
F2 - Acquisition Parameters
Date_ 20211030
Time 11.58
INSTRUM cryo500
PROBHD 5 mm CPTCI 1H-
PULPROG gnoe1cc22.prd
TD 65536
SOLVENT CDCl3
NS 128
DS 8
SWH 8012.820 Hz
FIDRES 0.122266 Hz
AQ 4.0894966 sec
RG 161.3
DW 62.400 usec
DE 6.00 usec
TE 298.0 K
D1 1.00000000 sec
D8 0.50000000 sec
D16 0.00020000 sec
d21 0.33375451 sec
d22 0.16399699 sec
p2 19.50 usec
======== CHANNEL f1 ========
NUC1 1H
P1 9.75 usec
p3 29.25 usec
p4 39.00 usec
p5 26.00 usec
P29 40000.00 usec
PL1 1.60 dB
SFO1 500.2228519 MHz
SP9 60.00 dB
SPNAM9 gauss1.512
SPOFF9 0.00 Hz
====== GRADIENT CHANNEL =====
GPNAM1 SMSQ10.100
GPNAM2 SMSQ10.100
GPNAM3 SMSQ10.100
GPNAM4 SMSQ10.100
GPX1 0.00 %
GPX2 0.00 %
GPX3 0.00 %
GPX4 0.00 %
GPY1 0.00 %
GPY2 0.00 %
GPY3 0.00 %
GPY4 0.00 %
GPZ1 7.00 %
GPZ2 3.00 %
GPZ3 2.30 %
GPZ4 -2.30 %
P16 1000.00 usec
F2 - Processing parameters
SI 65536
SF 500.2200000 MHz
WDW no
SSB 0
LB 0.00 Hz
GB 0
PC 1.00
1D NMR plot parameters
CX 22.80 cm
CY 50.00 cm
F1P 9.000 ppm
F1 4501.98 Hz
F2P -0.500 ppm
F2 -250.11 Hz
PPMCM 0.41667 ppm/cm
HZCM 208.42500 Hz/cm
Integral
1.0000
-0.004
-0.005
-0.002
-0.019
-0.012
ppm 8 7 6 5 4 3 2 1 0

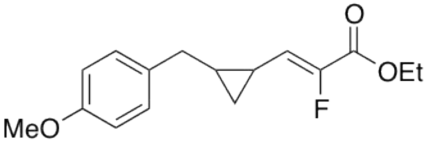

**14**

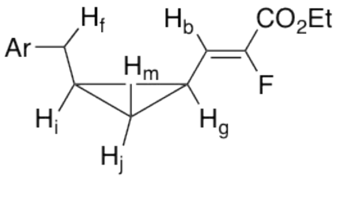


***cis*-cyclopropane**

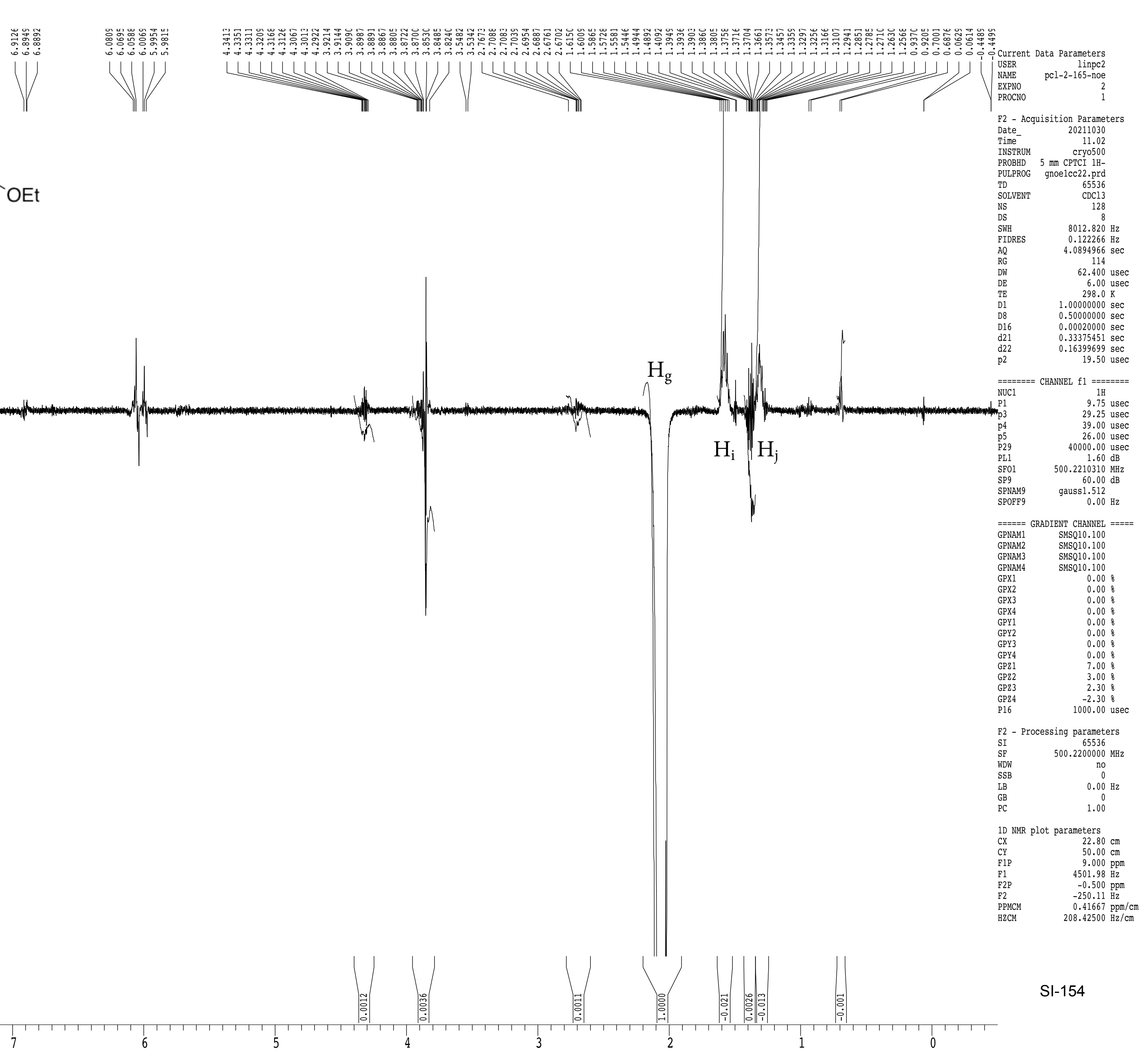

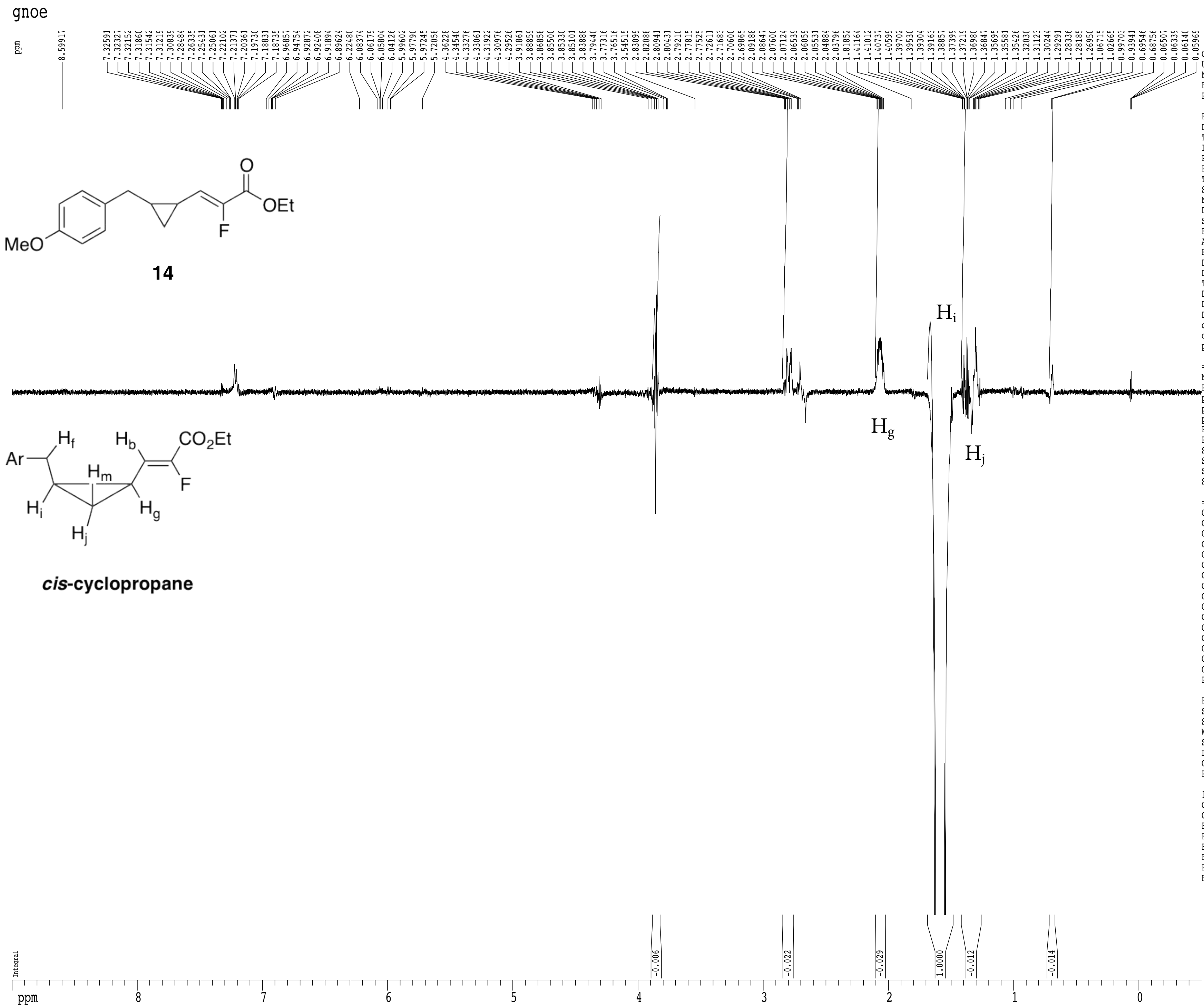


Current Data Parameters
USER linpc2
NAME pcl-2-165-noe
EXPNO 4
PROCNO 1

F2 - Acquisition Parameters
Date_ 20211030
Time 11.30
INSTRUM cryo500
PROBHD 5 mm CPTCI 1H-
PULPROG gnoe1cc22.prd
TD 65536
SOLVENT CDCl3
NS 128
DS 8
SWH 8012.820 Hz
FIDRES 0.122266 Hz
AQ 4.0894966 sec
RG 161.3
DW 62.400 usec
DE 6.00 usec
TE 298.0 K
D1 1.00000000 sec
D8 0.50000000 sec
D16 0.00020000 sec
d21 0.33375451 sec
d22 0.16399699 sec
p2 19.50 usec

======== CHANNEL f1 ========
NUC1 1H
P1 9.75 usec
p3 29.25 usec
p4 39.00 usec
p5 26.00 usec
P29 40000.00 usec
PL1 1.60 dB
SFO1 500.2207923 MHz
SP9 60.00 dB
SPNAM9 gauss1.512
SPOFF9 0.00 Hz

====== GRADIENT CHANNEL =====
GPNAM1 SMSQ10.100
GPNAM2 SMSQ10.100
GPNAM3 SMSQ10.100
GPNAM4 SMSQ10.100
GPX1 0.00 %
GPX2 0.00 %
GPX3 0.00 %
GPX4 0.00 %
GPY1 0.00 %
GPY2 0.00 %
GPY3 0.00 %
GPY4 0.00 %
GPZ1 7.00 %
GPZ2 3.00 %
GPZ3 2.30 %
GPZ4 -2.30 %
P16 1000.00 usec

F2 - Processing parameters
SI 65536
SF 500.2200000 MHz
WDW no
SSB 0
LB 0.00 Hz
GB 0
PC 1.00

1D NMR plot parameters
CX 22.80 cm
CY 50.00 cm
F1P 9.000 ppm
F1 4501.98 Hz
F2P -0.500 ppm
F2 -250.11 Hz
PPMCM 0.41667 ppm/cm
HZCM 208.42500 Hz/cm

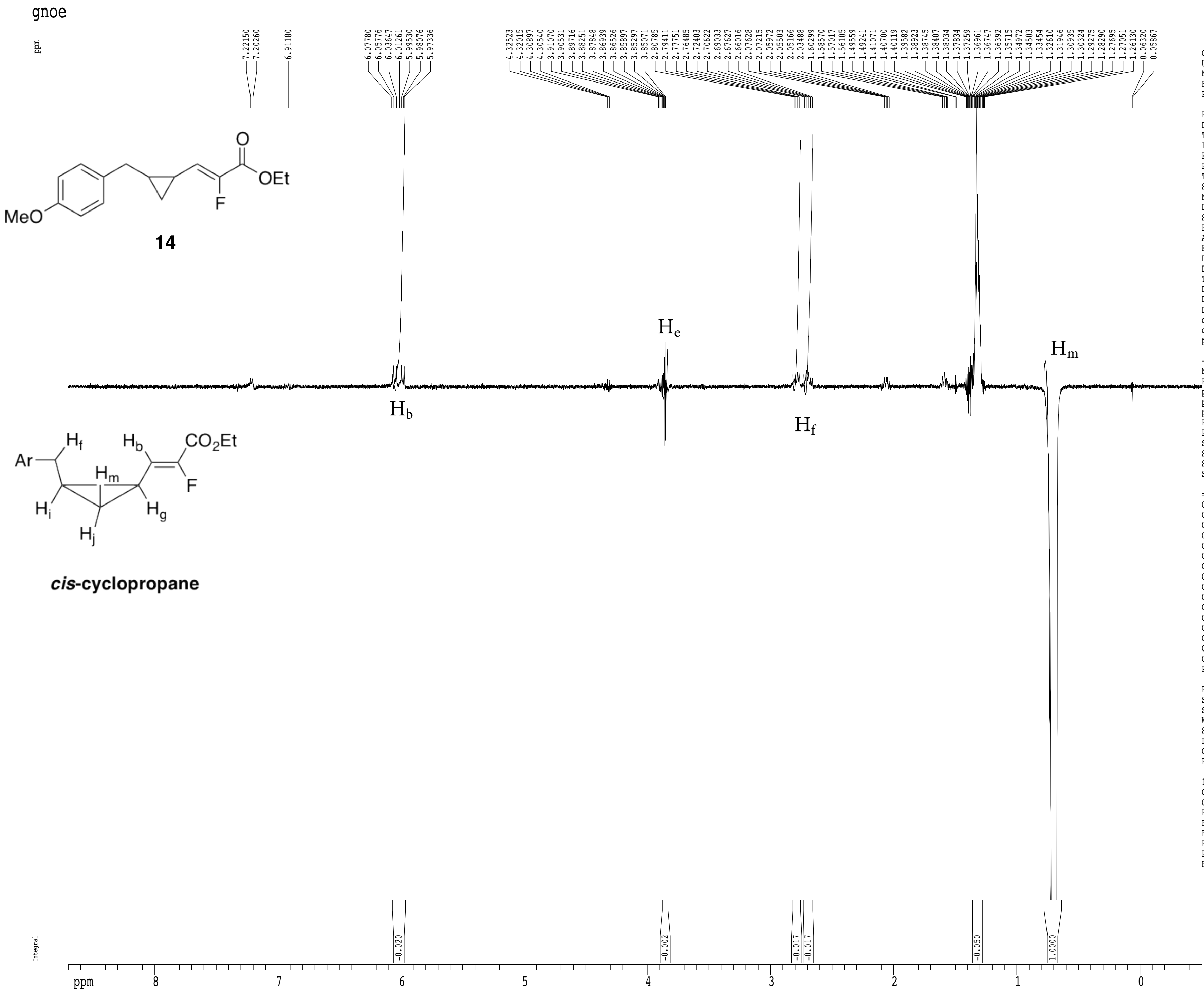


```
Current Data Parameters
USER            linpc2
NAME      pcl-2-165-noe
EXPNO                5
PROCNO               1

F2 - Acquisition Parameters
Date_         20211030
Time             11.44
INSTRUM        cryo500
PROBHD   5 mm CPTCI 1H-
PULPROG   gnoe1cc22.prd
TD               65536
SOLVENT          CDCl3
NS                 128
DS                   8
SWH           8012.820 Hz
FIDRES        0.122266 Hz
AQ           4.0894966 sec
RG               161.3
DW              62.400 usec
DE                6.00 usec
TE               298.0 K
D1          1.00000000 sec
D8          0.50000000 sec
D16         0.00020000 sec
d21         0.33375451 sec
d22         0.16399699 sec
p2               19.50 usec

======== CHANNEL f1 ========
NUC1                1H
P1                9.75 usec
p3               29.25 usec
p4               39.00 usec
p5               26.00 usec
P29           40000.00 usec
PL1               1.60 dB
SFO1       500.2203478 MHz
SP9              60.00 dB
SPNAM9       gauss1.512
SPOFF9            0.00 Hz

====== GRADIENT CHANNEL =====
GPNAM1       SMSQ10.100
GPNAM2       SMSQ10.100
GPNAM3       SMSQ10.100
GPNAM4       SMSQ10.100
GPX1              0.00 %
GPX2              0.00 %
GPX3              0.00 %
GPX4              0.00 %
GPY1              0.00 %
GPY2              0.00 %
GPY3              0.00 %
GPY4              0.00 %
GPZ1              7.00 %
GPZ2              3.00 %
GPZ3              2.30 %
GPZ4             -2.30 %
P16            1000.00 usec

F2 - Processing parameters
SI               65536
SF         500.2200000 MHz
WDW                 no
SSB                  0
LB                0.00 Hz
GB                   0
PC                1.00

1D NMR plot parameters
CX               22.80 cm
CY               50.00 cm
F1P              9.000 ppm
F1             4501.98 Hz
F2P             -0.500 ppm
F2             -250.11 Hz
PPMCM          0.41667 ppm/cm
HZCM         208.42500 Hz/cm
```

1H spectrum

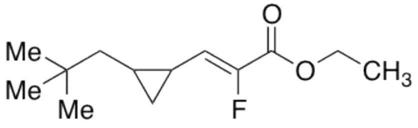


**15**

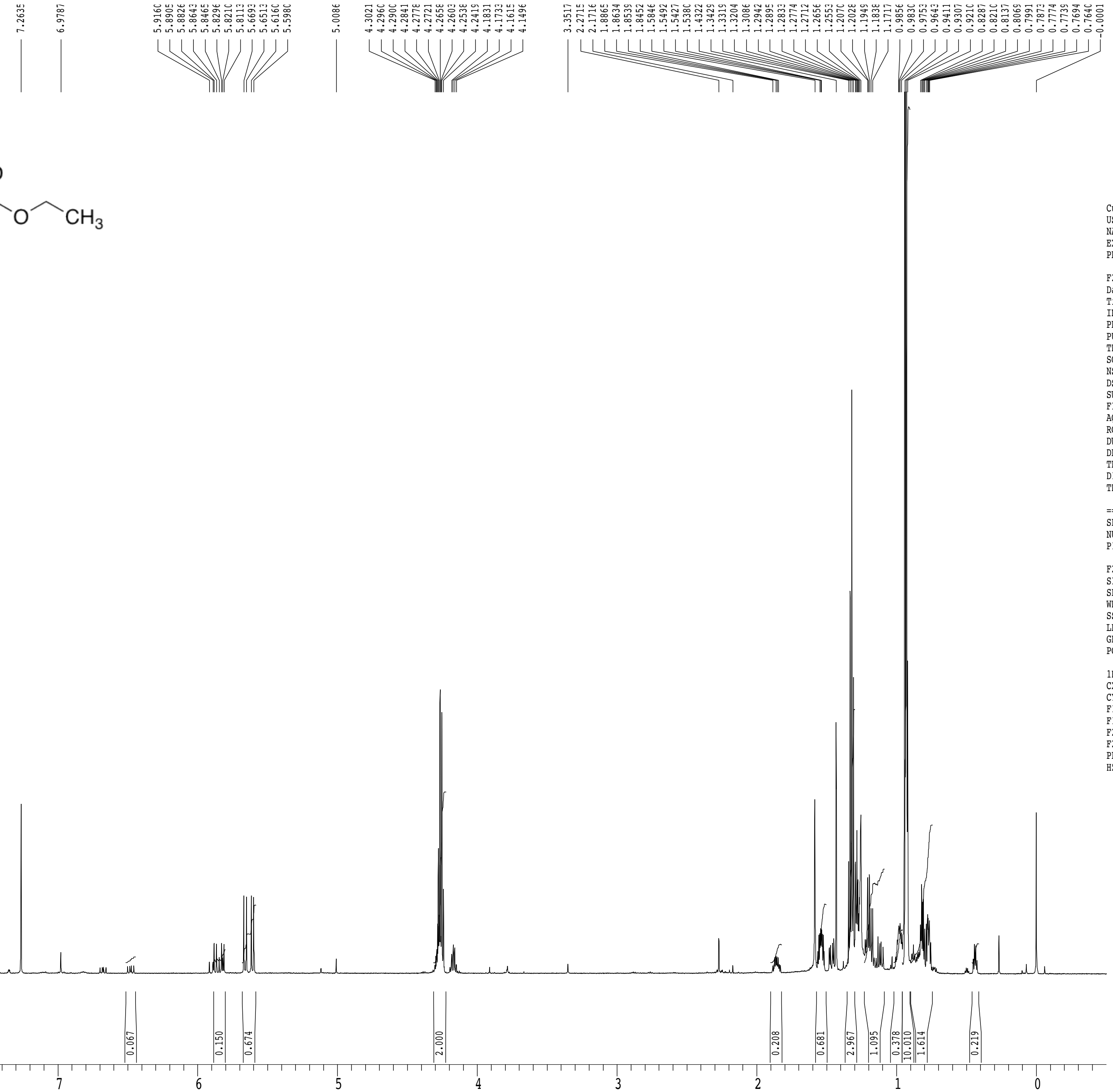


```
Current Data Parameters
USER            linpc2
NAME         pcl-2-153
EXPNO                2
PROCNO               1

F2 - Acquisition Parameters
Date_         20210818
Time             13.07
INSTRUM          av600
PROBHD   5 mm CPBBO BB-
PULPROG           zg30
TD               98074
SOLVENT          CDCl3T
NS                   8
DS                   2
SWH          9615.385 Hz
FIDRES       0.098042 Hz
AQ          5.0998979 sec
RG                  10
DW              52.000 usec
DE               14.23 usec
TE               298.0 K
D1          0.10000000 sec
TD0                  1

======== CHANNEL f1 ========
SFO1       600.1342009 MHz
NUC1                1H
P1                9.50 usec

F2 - Processing parameters
SI               65536
SF         600.1300329 MHz
WDW                 no
SSB                  0
LB                0.00 Hz
GB                   0
PC                1.00

1D NMR plot parameters
CX               22.80 cm
CY               45.00 cm
F1P              9.000 ppm
F1             5401.17 Hz
F2P             -0.500 ppm
F2             -300.06 Hz
PPMCM          0.41667 ppm/cm
HZCM         250.05418 Hz/cm
```

13C spectrum

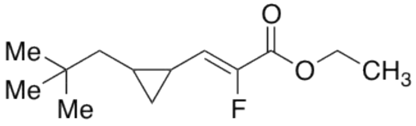


**15**

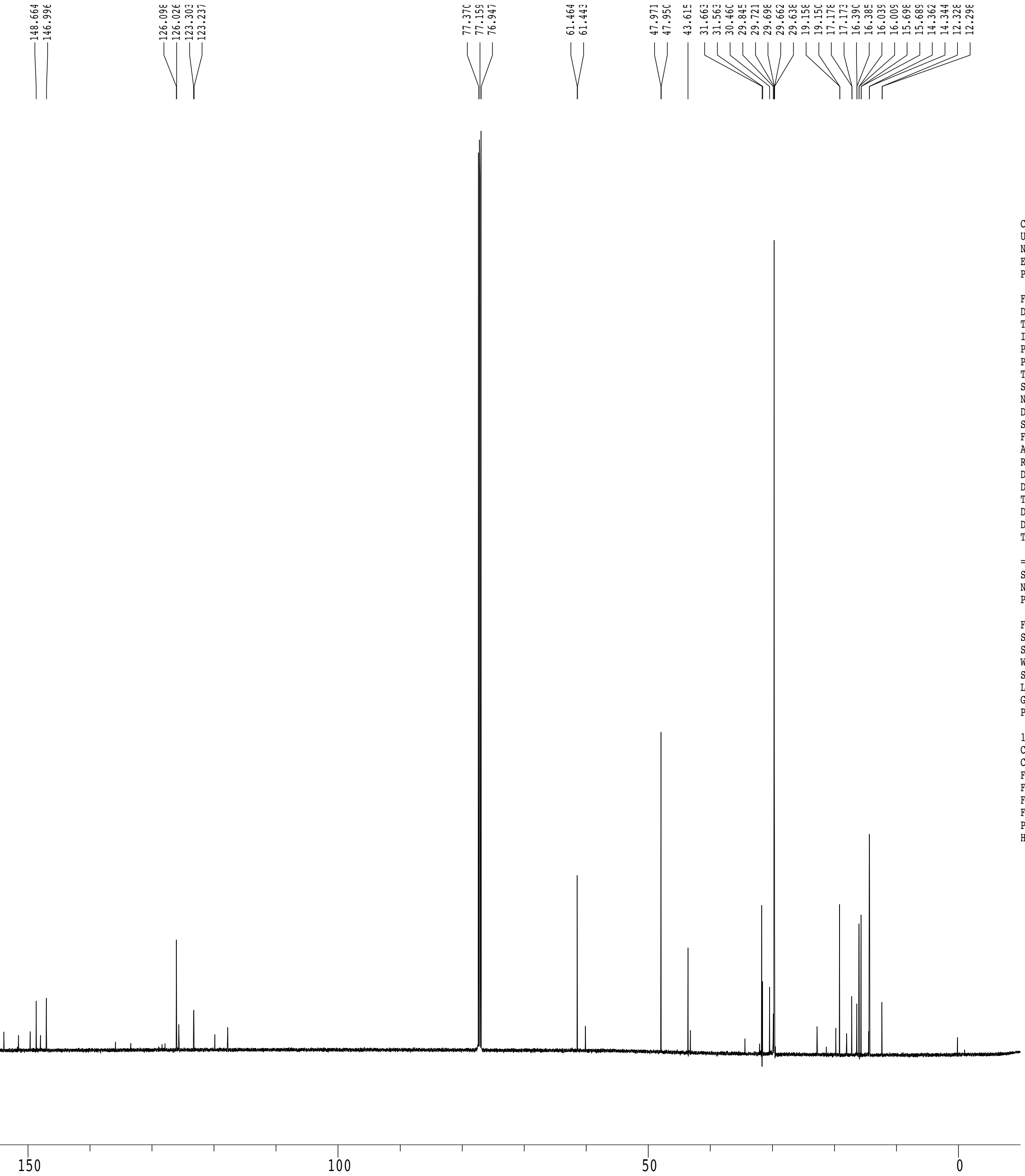


Current Data Parameters
USER linpc2
NAME pcl-2-153
EXPNO 4
PROCNO 1

F2 - Acquisition Parameters
Date_ 20210818
Time 13.20
INSTRUM av600
PROBHD 5 mm CPBBO BB-
PULPROG zgdc30
TD 65536
SOLVENT CDCl3T
NS 865
DS 4
SWH 36231.883 Hz
FIDRES 0.552855 Hz
AQ 0.9044468 sec
RG 2050
DW 13.800 usec
DE 19.63 usec
TE 298.0 K
D1 0.40000001 sec
D11 0.03000000 sec
TD0 1

======== CHANNEL f1 ========
SFO1 150.9194080 MHz
NUC1 13C
P1 10.10 usec

F2 - Processing parameters
SI 65536
SF 150.9027936 MHz
WDW no
SSB 0
LB 0.00 Hz
GB 0
PC 1.00

1D NMR plot parameters
CX 22.80 cm
CY 15.00 cm
F1P 220.000 ppm
F1 33198.61 Hz
F2P -10.000 ppm
F2 -1509.03 Hz
PPMCM 10.08772 ppm/cm
HZCM 1522.26501 Hz/cm

19F spectrum

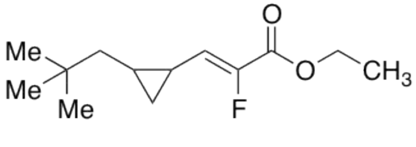


**15**

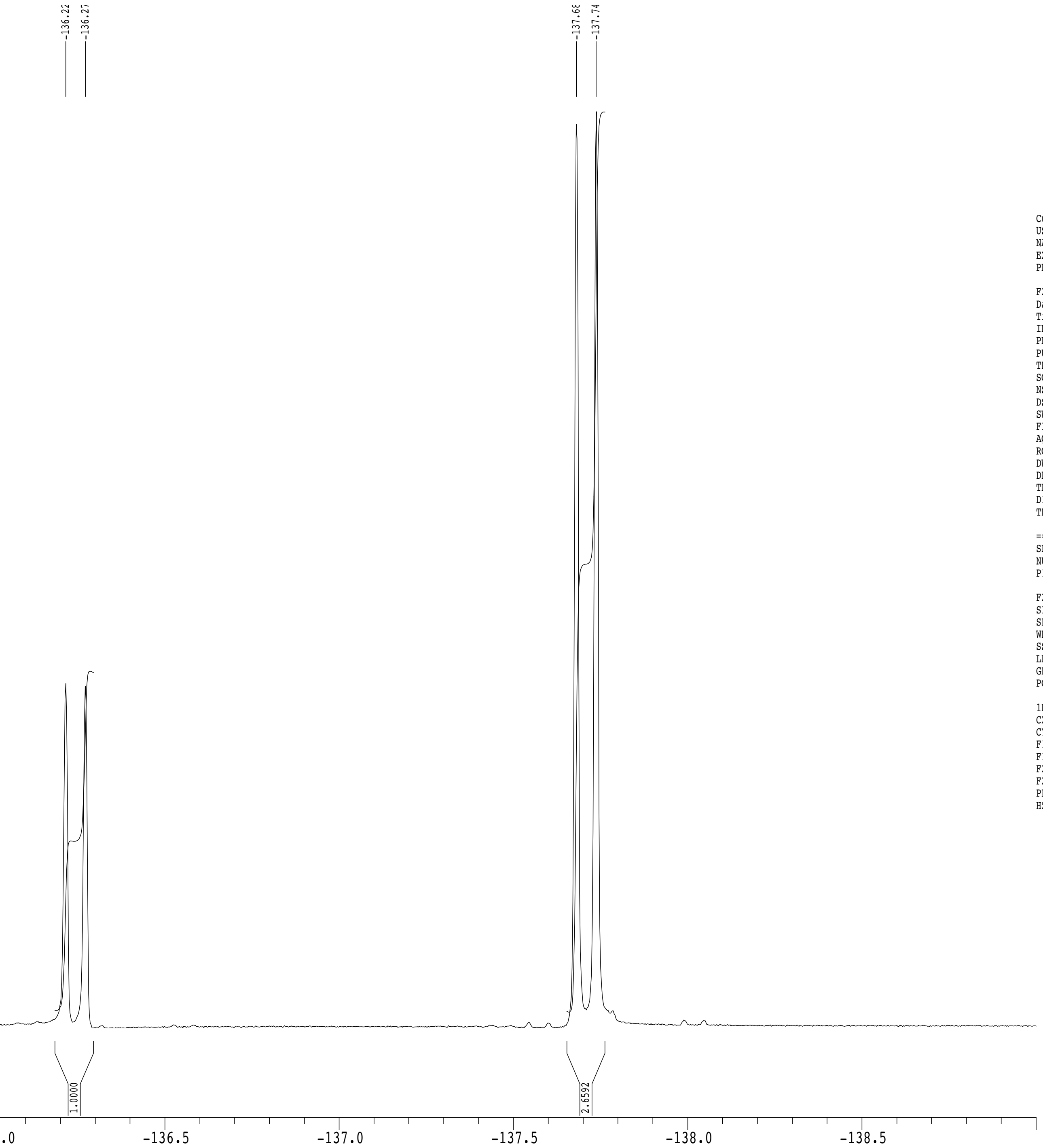


```
Current Data Parameters
USER              linpc2
NAME           pcl-2-153
EXPNO                  3
PROCNO                 1

F2 - Acquisition Parameters
Date_           20210818
Time               13.11
INSTRUM            av600
PROBHD   5 mm CPBBO BB-
PULPROG        zgflqn30
TD                131072
SOLVENT           CDCl3T
NS                    16
DS                     2
SWH           178571.422 Hz
FIDRES          1.362392 Hz
AQ             0.3670516 sec
RG                   575
DW                 2.800 usec
DE                 18.00 usec
TE                 298.0 K
D1            3.00000000 sec
TD0                    1

======== CHANNEL f1 ========
SFO1        564.6299196 MHz
NUC1                 19F
P1                 18.25 usec

F2 - Processing parameters
SI                131072
SF           564.6864191 MHz
WDW                   no
SSB                    0
LB                  0.00 Hz
GB                     0
PC                  1.00

1D NMR plot parameters
CX                 22.80 cm
CY                 15.00 cm
F1P             -135.000 ppm
F1             -76232.66 Hz
F2P             -139.000 ppm
F2             -78491.41 Hz
PPMCM            0.17544 ppm/cm
HZCM            99.06779 Hz/cm
```

1H spectrum

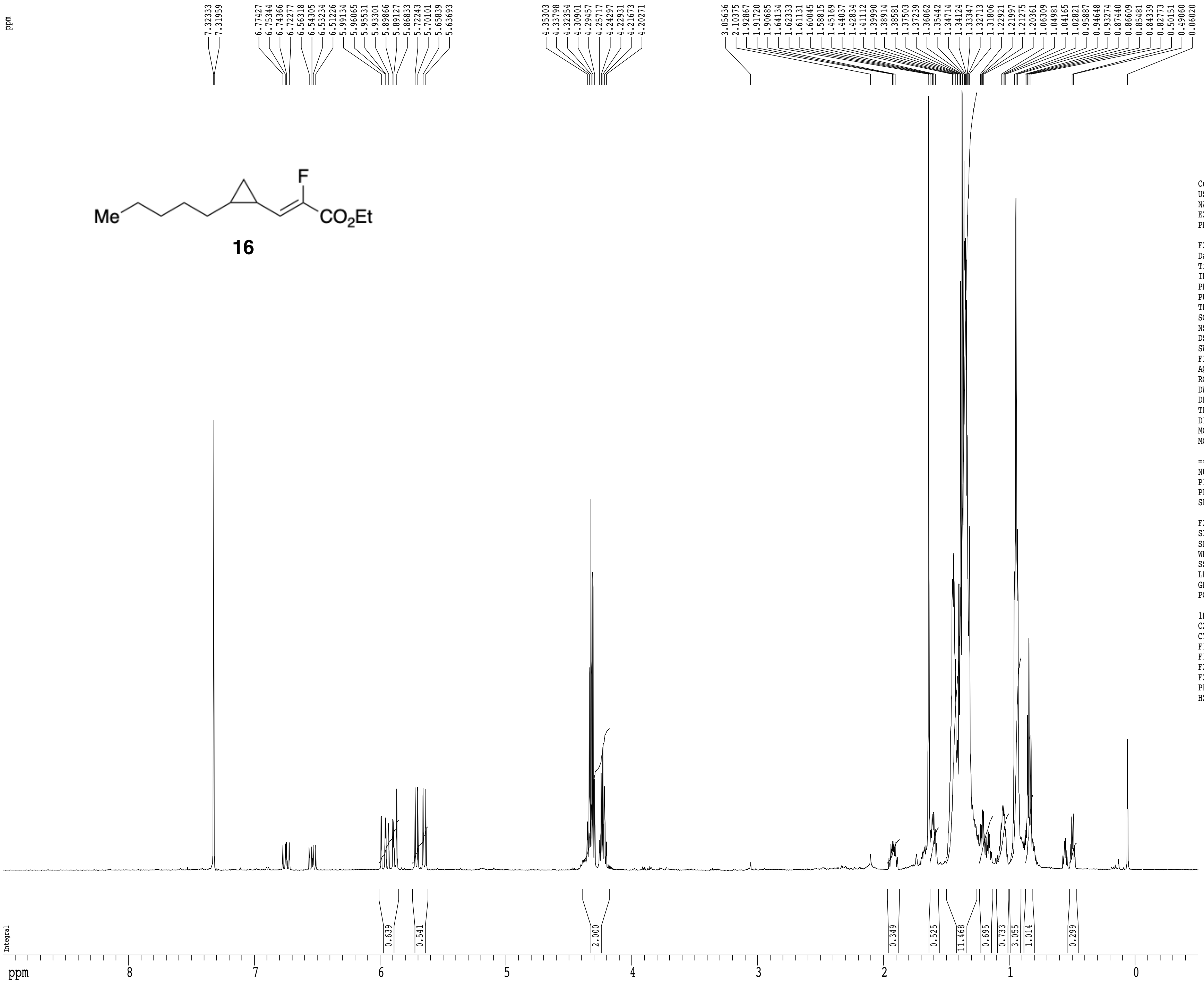


**16**



Current Data Parameters
USER mcginnit
NAME tmm-3-202
EXPNO 4
PROCNO 1

F2 - Acquisition Parameters
Date_ 20210806
Time 14.10
INSTRUM cryo500
PROBHD 5 mm CPTCI 1H-
PULPROG zg30
TD 81728
SOLVENT CDCl3
NS 8
DS 2
SWH 8012.820 Hz
FIDRES 0.098043 Hz
AQ 5.0998774 sec
RG 4.5
DW 62.400 usec
DE 6.00 usec
TE 298.0 K
D1 0.10000000 sec
MCREST 0.00000000 sec
MCWRK 0.01500000 sec

======== CHANNEL f1 ========
NUC1 1H
P1 9.75 usec
PL1 1.60 dB
SFO1 500.2235015 MHz

F2 - Processing parameters
SI 65536
SF 500.2200000 MHz
WDW EM
SSB 0
LB 0.30 Hz
GB 0
PC 1.00

1D NMR plot parameters
CX 22.80 cm
CY 15.00 cm
F1P 9.000 ppm
F1 4501.98 Hz
F2P -0.500 ppm
F2 -250.11 Hz
PPMCM 0.41667 ppm/cm
HZCM 208.42500 Hz/cm

Z-restored spin-echo 13C spectrum with 1H decoupling

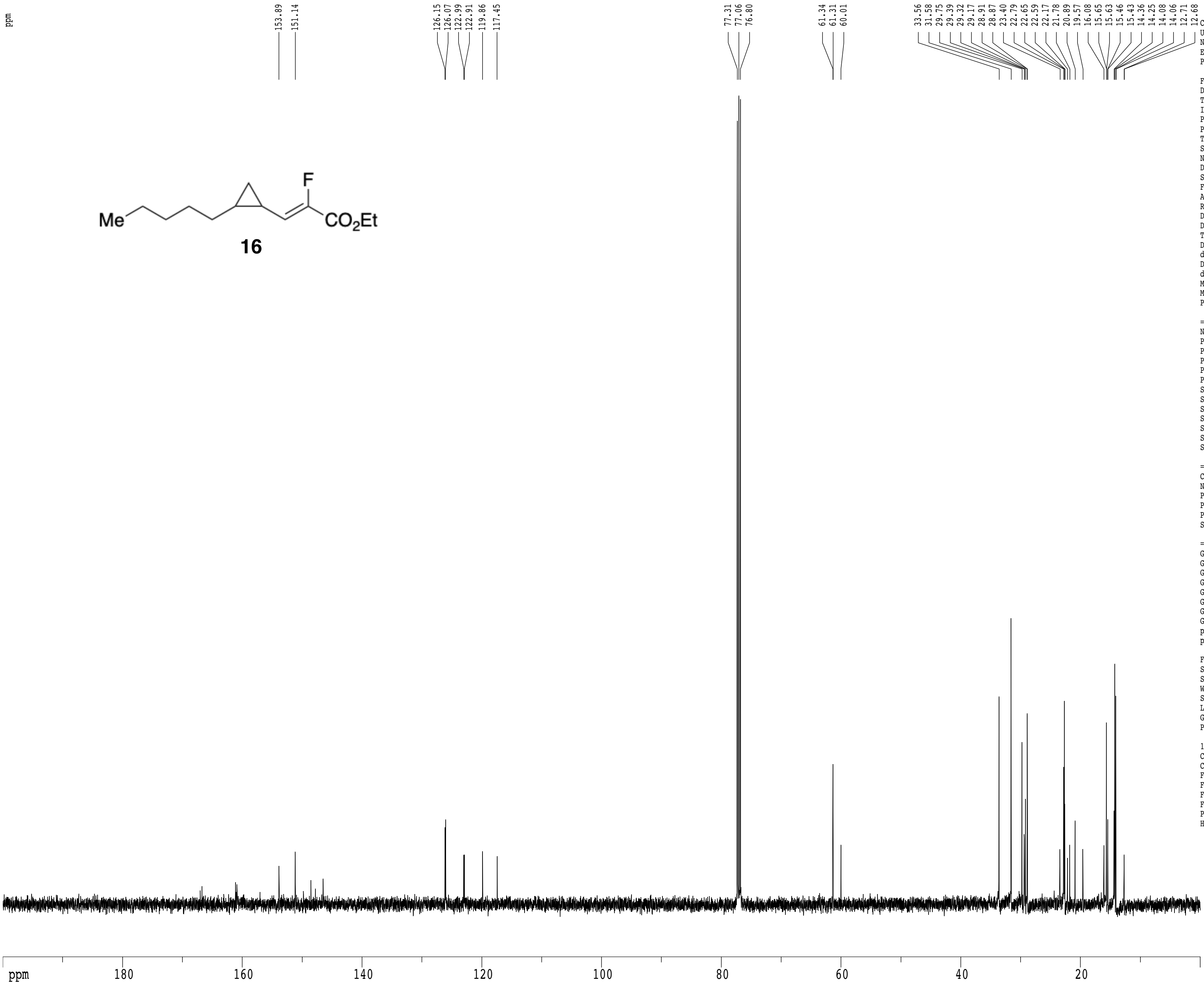


```
Current Data Parameters
USER          mcginnit
NAME          tmm-3-202
EXPNO                 5
PROCNO                1

F2 - Acquisition Parameters
Date_          20210806
Time              14.12
INSTRUM         cryo500
PROBHD   5 mm CPTCI 1H-
PULPROG  SpinEchopg30gp2.prd
TD                65536
SOLVENT           CDCl3
NS                  301
DS                   16
SWH           30303.031 Hz
FIDRES         0.462388 Hz
AQ            1.0813940 sec
RG               4597.6
DW               16.500 usec
DE                 6.00 usec
TE                298.0 K
D1           0.25000000 sec
d11          0.03000000 sec
D16          0.00020000 sec
d17          0.00019600 sec
MCREST       0.00000000 sec
MCWRK        0.01500000 sec
P2                37.70 usec

======== CHANNEL f1 ========
NUC1                13C
P1                18.85 usec
P12             2000.00 usec
P20              500.00 usec
PL0              120.00 dB
PL1               -1.00 dB
SFO1        125.7942548 MHz
SP2                1.55 dB
SP4                1.55 dB
SPNAM2      Crp60comp.4
SPNAM4   Crp60,0.5,20.1
SPOFF2             0.00 Hz
SPOFF4             0.00 Hz

======== CHANNEL f2 ========
CPDPRG2         waltz16
NUC2                 1H
PCPD2            100.00 usec
PL2                1.60 dB
PL12              22.00 dB
SFO2        500.2225011 MHz

====== GRADIENT CHANNEL =====
GPNAM1         SINE.100
GPNAM2         SINE.100
GPX1               0.00 %
GPX2               0.00 %
GPY1               0.00 %
GPY2               0.00 %
GPZ1              30.00 %
GPZ2              50.00 %
p15              500.00 usec
p16             1000.00 usec

F2 - Processing parameters
SI                65536
SF          125.7804190 MHz
WDW                  EM
SSB                   0
LB                 1.00 Hz
GB                    0
PC                 2.00

1D NMR plot parameters
CX                22.80 cm
CY                15.65 cm
F1P             200.000 ppm
F1             25156.08 Hz
F2P               0.000 ppm
F2                 0.00 Hz
PPMCM           8.77193 ppm/cm
HZCM         1103.33704 Hz/cm
```

19F spectrum

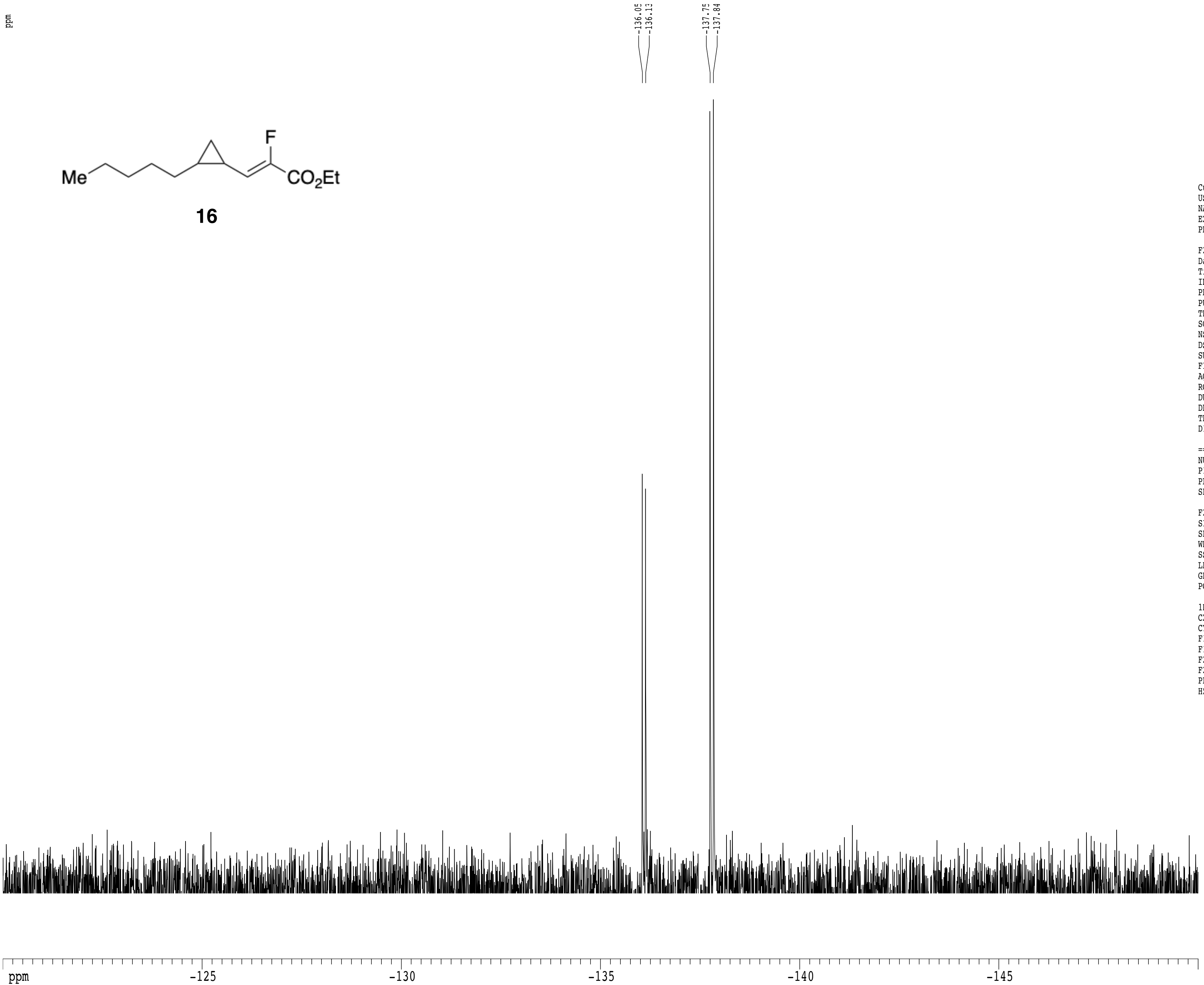


```
Current Data Parameters
USER          mcginnit
NAME          tmm-3-202
EXPNO                6
PROCNO               1

F2 - Acquisition Parameters
Date_         20210806
Time             15.53
INSTRUM         drx400
PROBHD   5 mm QNP H/F/P
PULPROG       zgflqn30
TD               65536
SOLVENT          CDCl3
NS                 106
DS                   2
SWH          75187.969 Hz
FIDRES        1.147277 Hz
AQ           0.4358644 sec
RG                3251
DW               6.650 usec
DE                9.46 usec
TE               298.0 K
D1          2.00000000 sec

======== CHANNEL f1 ========
NUC1               19F
P1               11.75 usec
PL1              -6.00 dB
SFO1       376.4646491 MHz

F2 - Processing parameters
SI               65536
SF         376.4984640 MHz
WDW                 EM
SSB                  0
LB                1.00 Hz
GB                   0
PC                1.00

1D NMR plot parameters
CX               22.80 cm
CY               15.00 cm
F1P           -120.000 ppm
F1           -45179.82 Hz
F2P           -150.000 ppm
F2           -56474.77 Hz
PPMCM          1.31579 ppm/cm
HZCM         495.39273 Hz/cm
```

# 1H spectrum

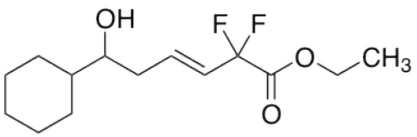


**SI-26**

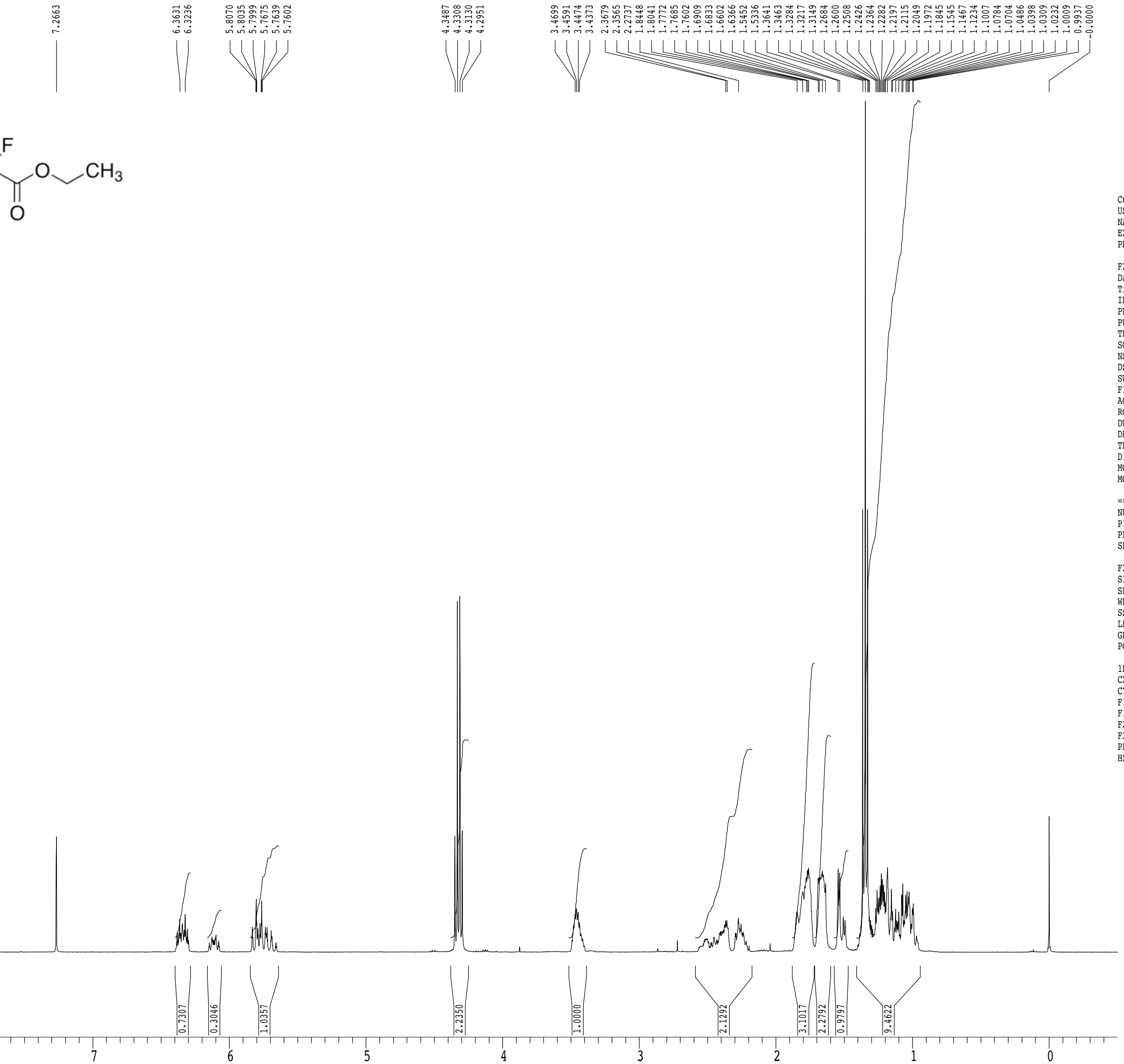


Current Data Parameters
USER linpc2
NAME pcl-1-247
EXPNO 4
PROCNO 1

F2 - Acquisition Parameters
Date_ 20210219
Time 15.42
INSTRUM drx400
PROBHD 5 mm QNP H/F/P
PULPROG zg30
TD 65536
SOLVENT CDCl3
NS 8
DS 2
SWH 6410.256 Hz
FIDRES 0.097813 Hz
AQ 5.1118579 sec
RG 128
DW 78.000 usec
DE 4.50 usec
TE 298.0 K
D1 0.10000000 sec
MCREST 0.00000000 sec
MCWRK 0.01500000 sec

======== CHANNEL f1 ========
NUC1 1H
P1 12.00 usec
PL1 -1.60 dB
SFO1 400.1328009 MHz

F2 - Processing parameters
SI 65536
SF 400.1300187 MHz
WDW EM
SSB 0
LB 0.30 Hz
GB 0
PC 2.00

1D NMR plot parameters
CX 22.80 cm
CY 15.00 cm
F1P 9.000 ppm
F1 3601.17 Hz
F2P -0.500 ppm
F2 -200.06 Hz
PPMCM 0.41667 ppm/cm
HZCM 166.72084 Hz/cm

13C spectrum with 1H decoupling

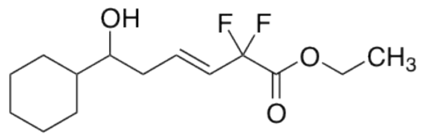

**SI-26**

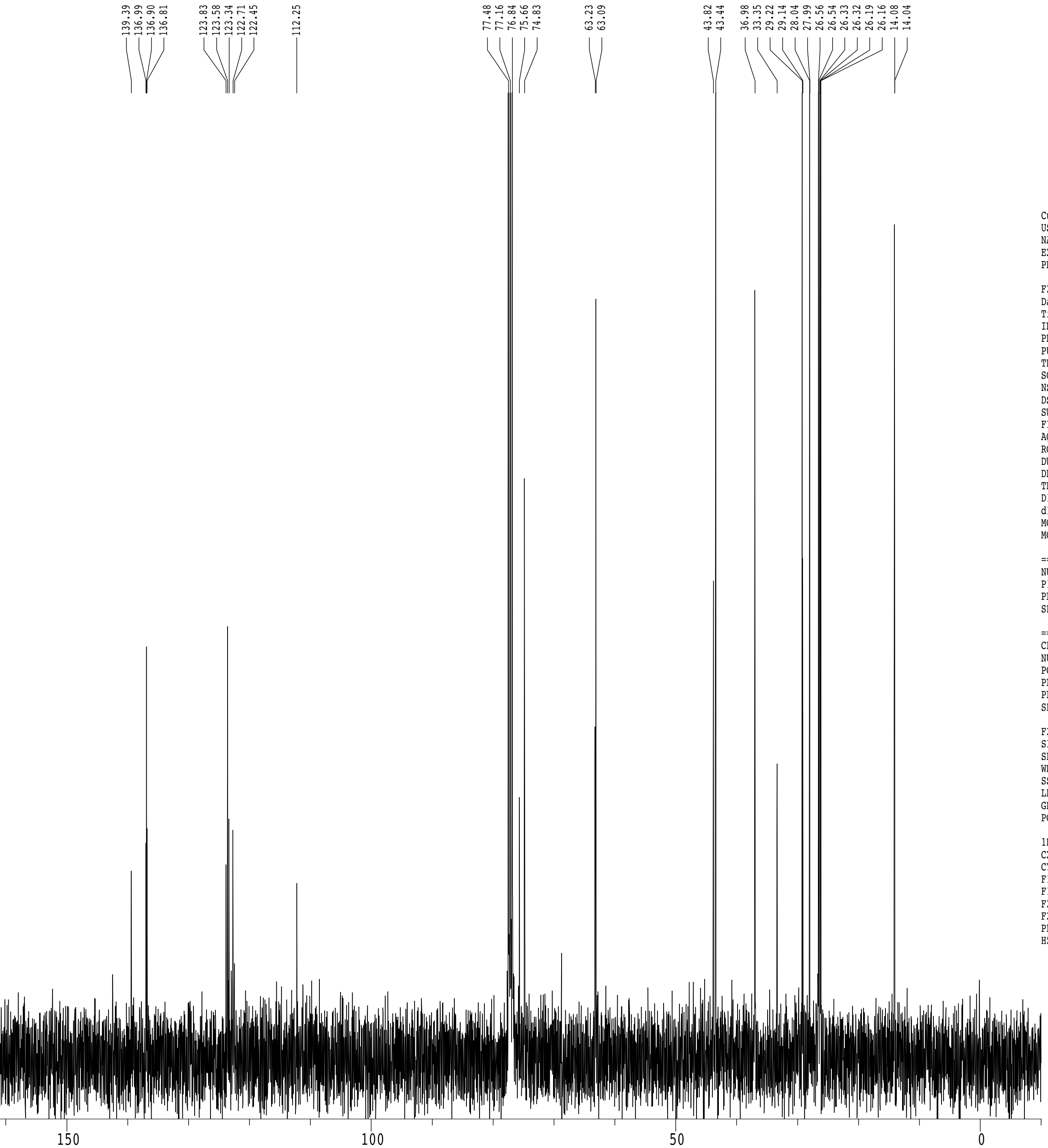


Current Data Parameters
USER linpc2
NAME pcl-1-247
EXPNO 5
PROCNO 1

F2 - Acquisition Parameters
Date_ 20210219
Time 15.46
INSTRUM drx400
PROBHD 5 mm QNP H/F/P
PULPROG zgdc30
TD 65536
SOLVENT CDCl3
NS 160
DS 4
SWH 24154.590 Hz
FIDRES 0.368570 Hz
AQ 1.3566452 sec
RG 9195.2
DW 20.700 usec
DE 20.39 usec
TE 298.0 K
D1 0.10000000 sec
d11 0.03000000 sec
MCREST 0.00000000 sec
MCWRK 0.01500000 sec

======== CHANNEL f1 ========
NUC1 13C
P1 8.30 usec
PL1 -3.00 dB
SFO1 100.6237964 MHz

======== CHANNEL f2 ========
CPDPRG2 waltz16
NUC2 1H
PCPD2 90.00 usec
PL2 -1.60 dB
PL12 16.50 dB
SFO2 400.1328009 MHz

F2 - Processing parameters
SI 65536
SF 100.6127573 MHz
WDW EM
SSB 0
LB 1.00 Hz
GB 0
PC 1.00

1D NMR plot parameters
CX 22.80 cm
CY 45.00 cm
F1P 220.000 ppm
F1 22134.81 Hz
F2P -10.000 ppm
F2 -1006.13 Hz
PPMCM 10.08772 ppm/cm
HZCM 1014.95325 Hz/cm

1H spectrum

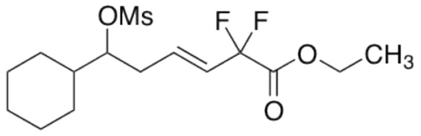
1a

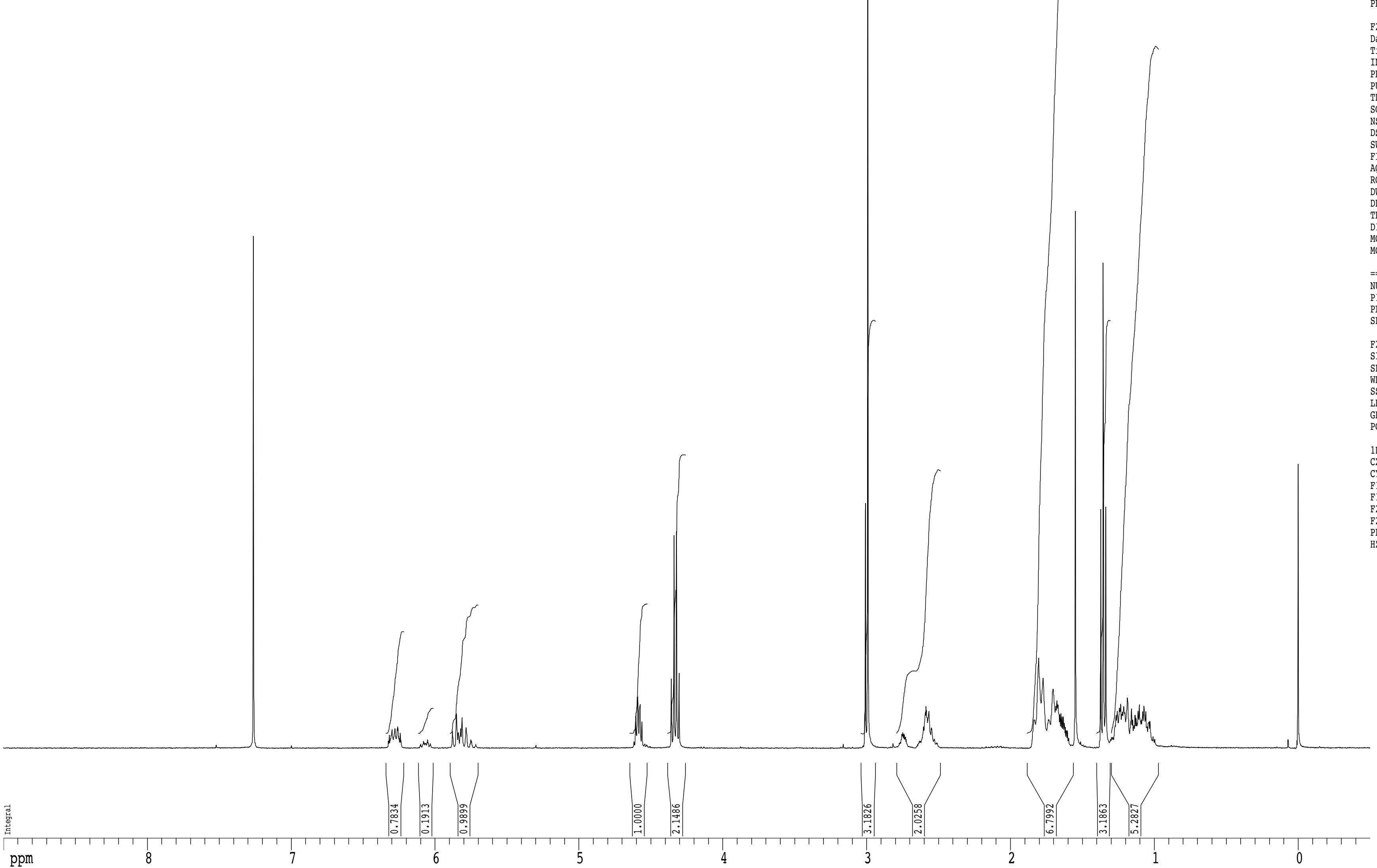


Current Data Parameters
USER linpc2
NAME pcl-1-301
EXPNO 1
PROCNO 1

F2 - Acquisition Parameters
Date_ 20210412
Time 14.43
INSTRUM drx400
PROBHD 5 mm QNP H/F/P
PULPROG zg30
TD 65536
SOLVENT CDCl3
NS 8
DS 2
SWH 6410.256 Hz
FIDRES 0.097813 Hz
AQ 5.1118579 sec
RG 574.7
DW 78.000 usec
DE 4.50 usec
TE 298.0 K
D1 0.10000000 sec
MCREST 0.00000000 sec
MCWRK 0.01500000 sec

======== CHANNEL f1 ========
NUC1 1H
P1 12.00 usec
PL1 -1.60 dB
SFO1 400.1328009 MHz

F2 - Processing parameters
SI 65536
SF 400.1300212 MHz
WDW EM
SSB 0
LB 0.30 Hz
GB 0
PC 2.00

1D NMR plot parameters
CX 22.80 cm
CY 15.00 cm
F1P 9.000 ppm
F1 3601.17 Hz
F2P -0.500 ppm
F2 -200.06 Hz
PPMCM 0.41667 ppm/cm
HZCM 166.72086 Hz/cm

13C spectrum

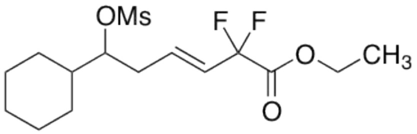


**1a**

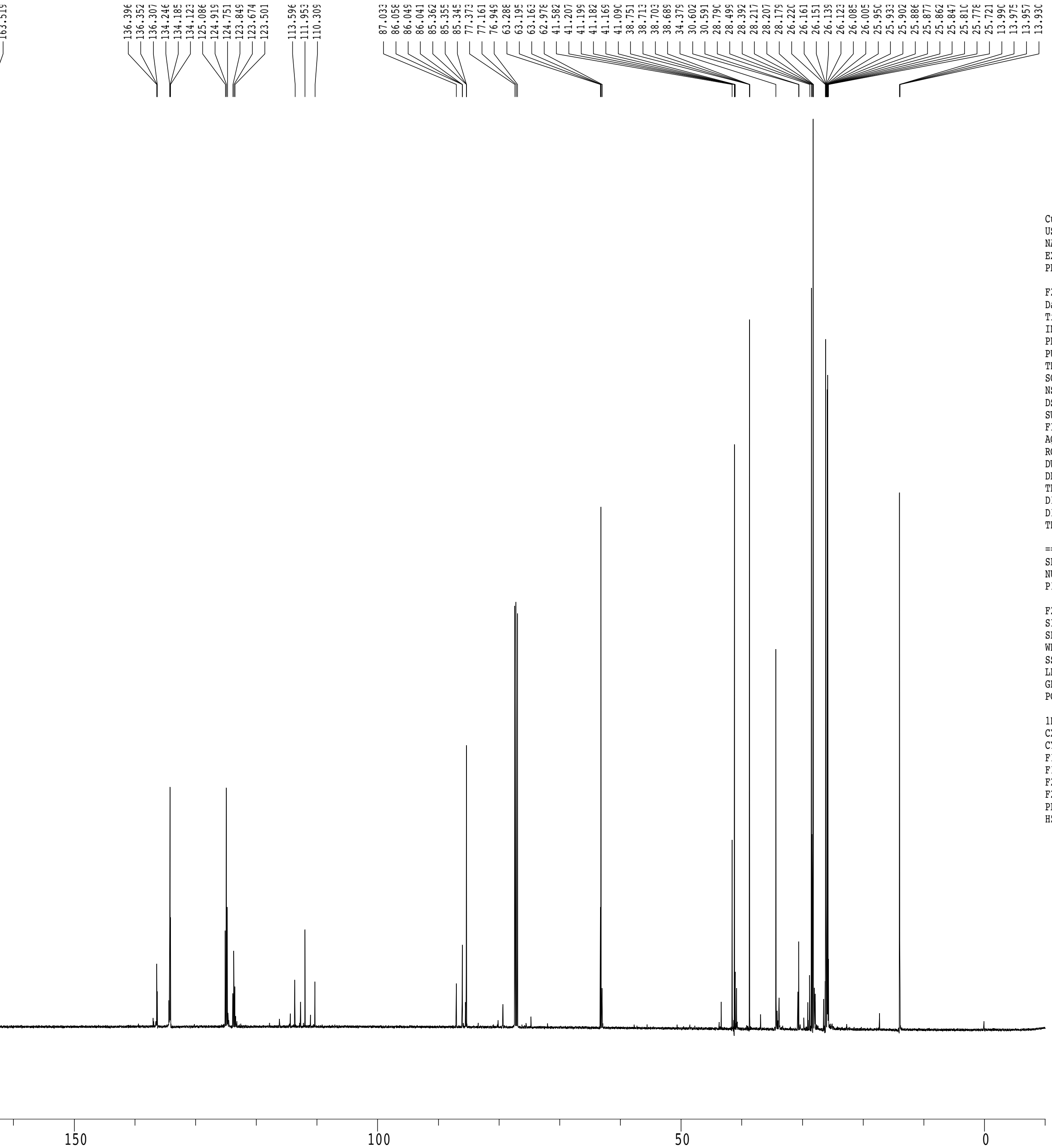


Current Data Parameters
USER linpc2
NAME pcl-2-109
EXPNO 3
PROCNO 1

F2 - Acquisition Parameters
Date_ 20210816
Time 14.52
INSTRUM av600
PROBHD 5 mm CPBBO BB-
PULPROG zgdc30
TD 65536
SOLVENT CDCl3T
NS 730
DS 4
SWH 36231.883 Hz
FIDRES 0.552855 Hz
AQ 0.9044468 sec
RG 2050
DW 13.800 usec
DE 19.63 usec
TE 298.0 K
D1 0.40000001 sec
D11 0.03000000 sec
TD0 1

======== CHANNEL f1 ========
SFO1 150.9194080 MHz
NUC1 13C
P1 10.10 usec

F2 - Processing parameters
SI 65536
SF 150.9028025 MHz
WDW no
SSB 0
LB 0.00 Hz
GB 0
PC 1.00

1D NMR plot parameters
CX 22.80 cm
CY 15.00 cm
F1P 220.000 ppm
F1 33198.62 Hz
F2P -10.000 ppm
F2 -1509.03 Hz
PPMCM 10.08772 ppm/cm
HZCM 1522.26514 Hz/cm

19F spectrum

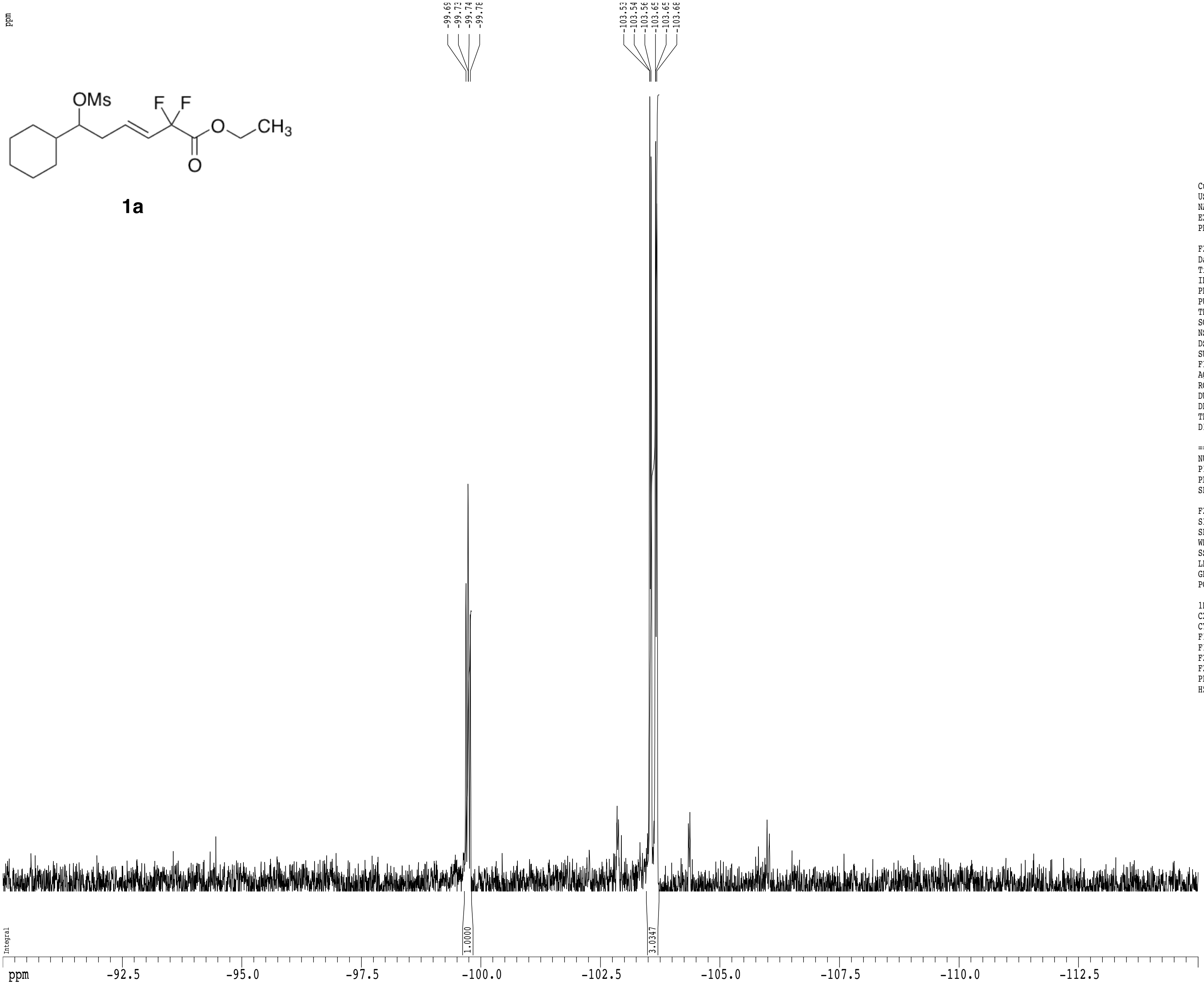


Current Data Parameters
USER linpc2
NAME pcl-2-014
EXPNO 5
PROCNO 1

F2 - Acquisition Parameters
Date_ 20210426
Time 17.17
INSTRUM drx400
PROBHD 5 mm QNP H/F/P
PULPROG zgflqn30
TD 65536
SOLVENT CDCl3
NS 32
DS 2
SWH 75187.969 Hz
FIDRES 1.147277 Hz
AQ 0.4358644 sec
RG 4597.6
DW 6.650 usec
DE 9.46 usec
TE 298.0 K
D1 2.00000000 sec

======== CHANNEL f1 ========
NUC1 19F
P1 11.75 usec
PL1 -6.00 dB
SFO1 376.4646491 MHz

F2 - Processing parameters
SI 65536
SF 376.4984640 MHz
WDW EM
SSB 0
LB 1.00 Hz
GB 0
PC 1.00

1D NMR plot parameters
CX 22.80 cm
CY 15.00 cm
F1P -90.000 ppm
F1 -33884.87 Hz
F2P -115.000 ppm
F2 -43297.33 Hz
PPMCM 1.09649 ppm/cm
HZCM 412.82730 Hz/cm

1H spectrum

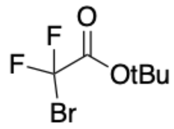

**SI-27**

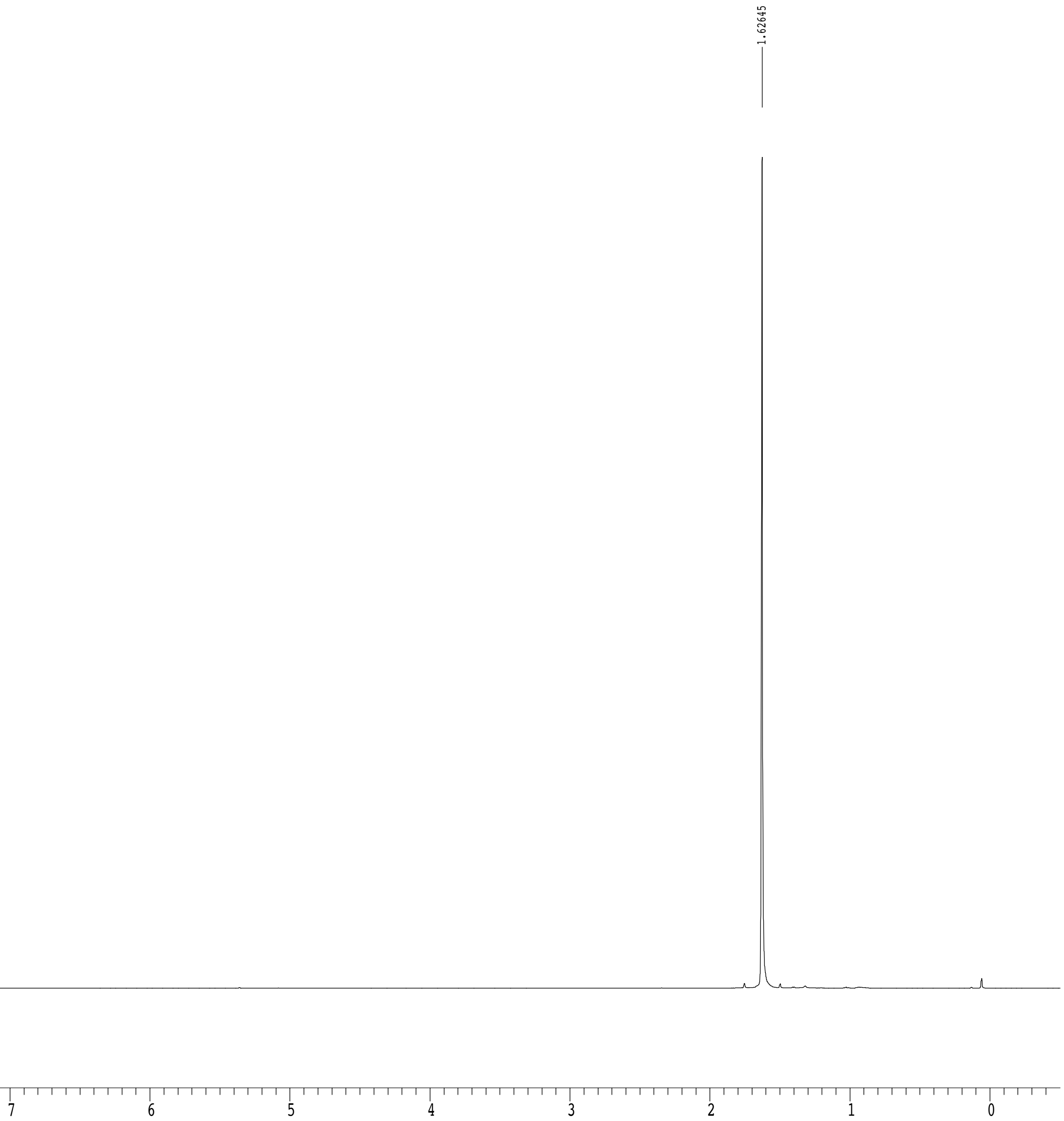


Current Data Parameters
USER mcginnit
NAME tmm-3-163
EXPNO 1
PROCNO 1

F2 - Acquisition Parameters
Date_ 20210615
Time 12.09
INSTRUM gn500
PROBHD 5 mm broadband
PULPROG zg30
TD 81728
SOLVENT CDCl3
NS 8
DS 2
SWH 8012.820 Hz
FIDRES 0.098043 Hz
AQ 5.0998774 sec
RG 181
DW 62.400 usec
DE 6.00 usec
TE 298.0 K
D1 0.10000000 sec
MCREST 0.00000000 sec
MCWRK 0.01500000 sec

======== CHANNEL f1 ========
NUC1 1H
P1 12.00 usec
PL1 -6.00 dB
SFO1 498.7534913 MHz

F2 - Processing parameters
SI 65536
SF 498.7500000 MHz
WDW EM
SSB 0
LB 0.30 Hz
GB 0
PC 1.00

1D NMR plot parameters
CX 20.00 cm
CY 12.50 cm
F1P 9.000 ppm
F1 4488.75 Hz
F2P -0.500 ppm
F2 -249.38 Hz
PPMCM 0.47500 ppm/cm
HZCM 236.90625 Hz/cm

13C spectrum with 1H decoupling

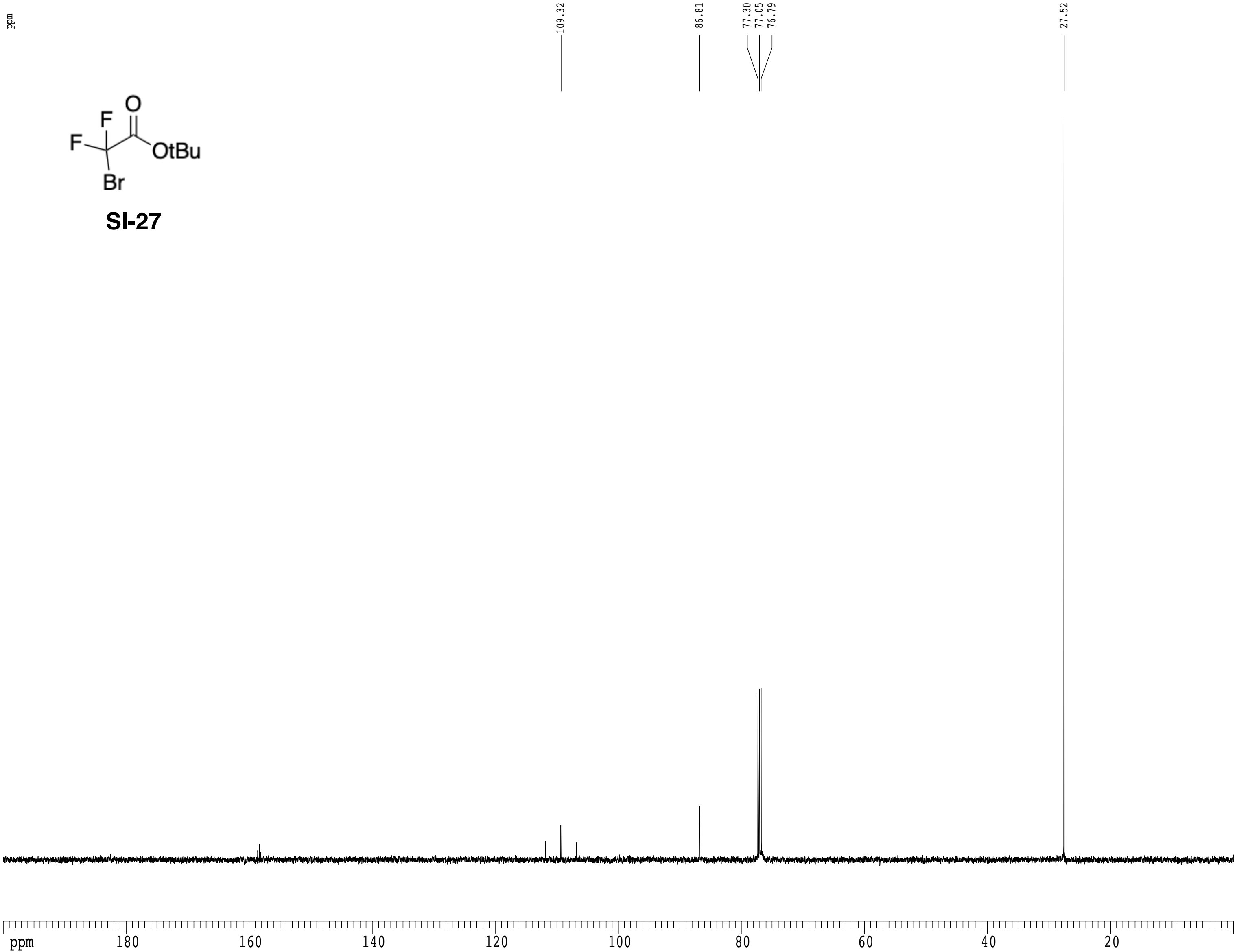


Current Data Parameters
USER mcginnit
NAME tmm-3-163
EXPNO 2
PROCNO 1

F2 - Acquisition Parameters
Date_ 20210615
Time 12.11
INSTRUM gn500
PROBHD 5 mm broadband
PULPROG zgdc30
TD 65536
SOLVENT CDCl3
NS 296
DS 4
SWH 30303.031 Hz
FIDRES 0.462388 Hz
AQ 1.0813940 sec
RG 5792.6
DW 16.500 usec
DE 6.00 usec
TE 298.0 K
D1 0.25000000 sec
d11 0.03000000 sec
MCREST 0.00000000 sec
MCWRK 0.01500000 sec

======== CHANNEL f1 ========
NUC1 13C
P1 14.20 usec
PL1 -6.00 dB
SFO1 125.4245824 MHz

======== CHANNEL f2 ========
CPDPRG2 waltz16
NUC2 1H
PCPD2 80.00 usec
PL2 -6.00 dB
PL12 12.30 dB
SFO2 498.7524937 MHz

F2 - Processing parameters
SI 65536
SF 125.4107870 MHz
WDW EM
SSB 0
LB 1.00 Hz
GB 0
PC 2.00

1D NMR plot parameters
CX 20.00 cm
CY 12.50 cm
F1P 200.000 ppm
F1 25082.16 Hz
F2P 0.000 ppm
F2 0.00 Hz
PPMCM 10.00000 ppm/cm
HZCM 1254.10791 Hz/cm

19F spectrum

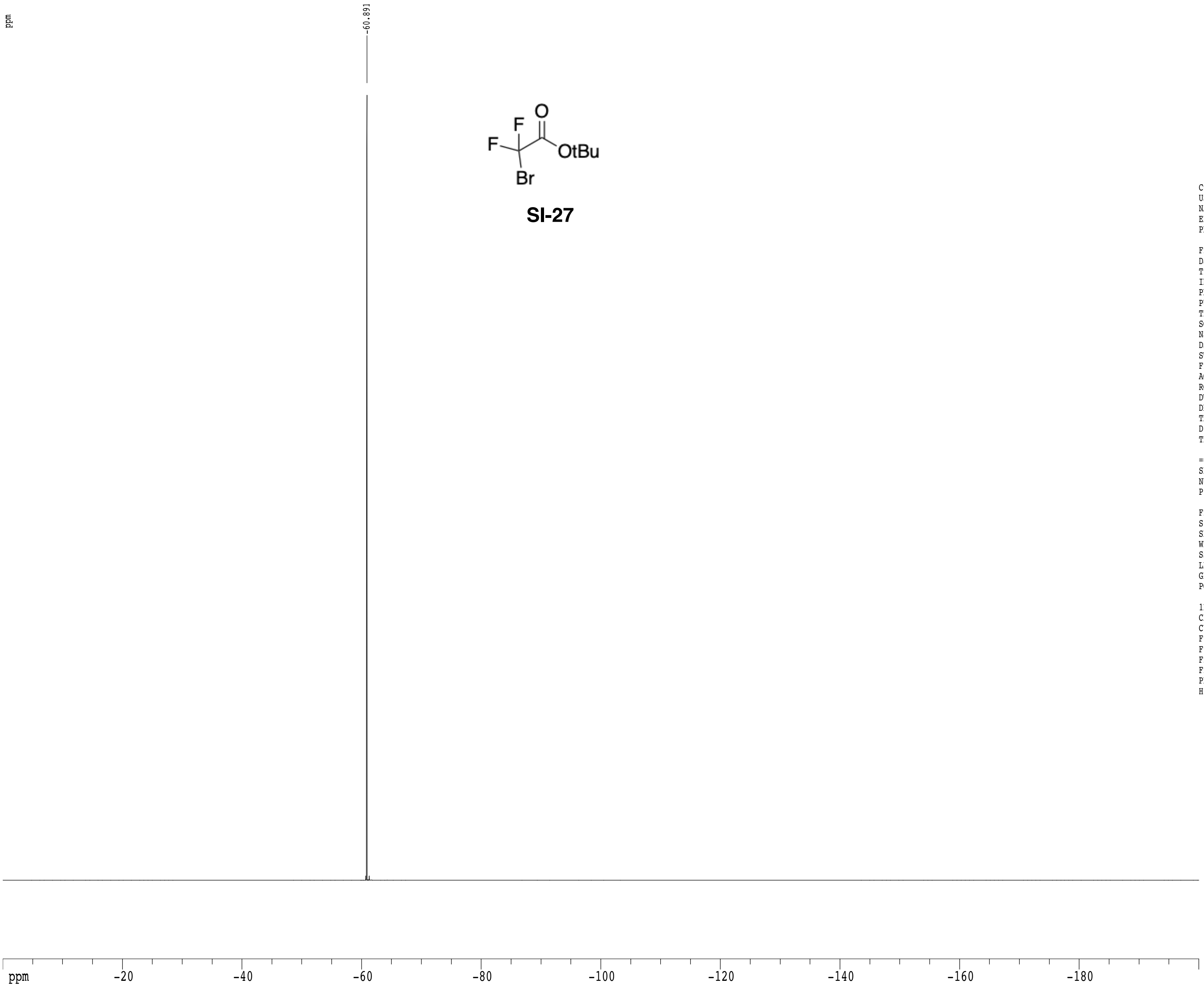


**SI-27**

```
Current Data Parameters
USER              mcginnit
NAME             tmm-3-163
EXPNO                    3
PROCNO                   1

F2 - Acquisition Parameters
Date_             20210615
Time                 12.37
INSTRUM              av600
PROBHD   5 mm CPBBO BB-
PULPROG          zgflqn30
TD                  131072
SOLVENT              CDCl3
NS                      16
DS                       2
SWH             178571.422 Hz
FIDRES            1.362392 Hz
AQ               0.3670516 sec
RG                     575
DW                   2.800 usec
DE                   18.00 usec
TE                   298.0 K
D1              3.00000000 sec
TD0                      1

======== CHANNEL f1 ========
SFO1         564.6299196 MHz
NUC1                   19F
P1                   18.25 usec

F2 - Processing parameters
SI                  131072
SF             564.6863858 MHz
WDW                     no
SSB                      0
LB                    0.00 Hz
GB                       0
PC                    1.00

1D NMR plot parameters
CX                   22.80 cm
CY                   15.00 cm
F1P                  0.000 ppm
F1                    0.00 Hz
F2P               -200.000 ppm
F2              -112937.28 Hz
PPMCM              8.77193 ppm/cm
HZCM            4953.38965 Hz/cm
```

1H spectrum

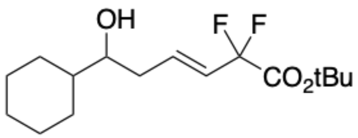

SI-28

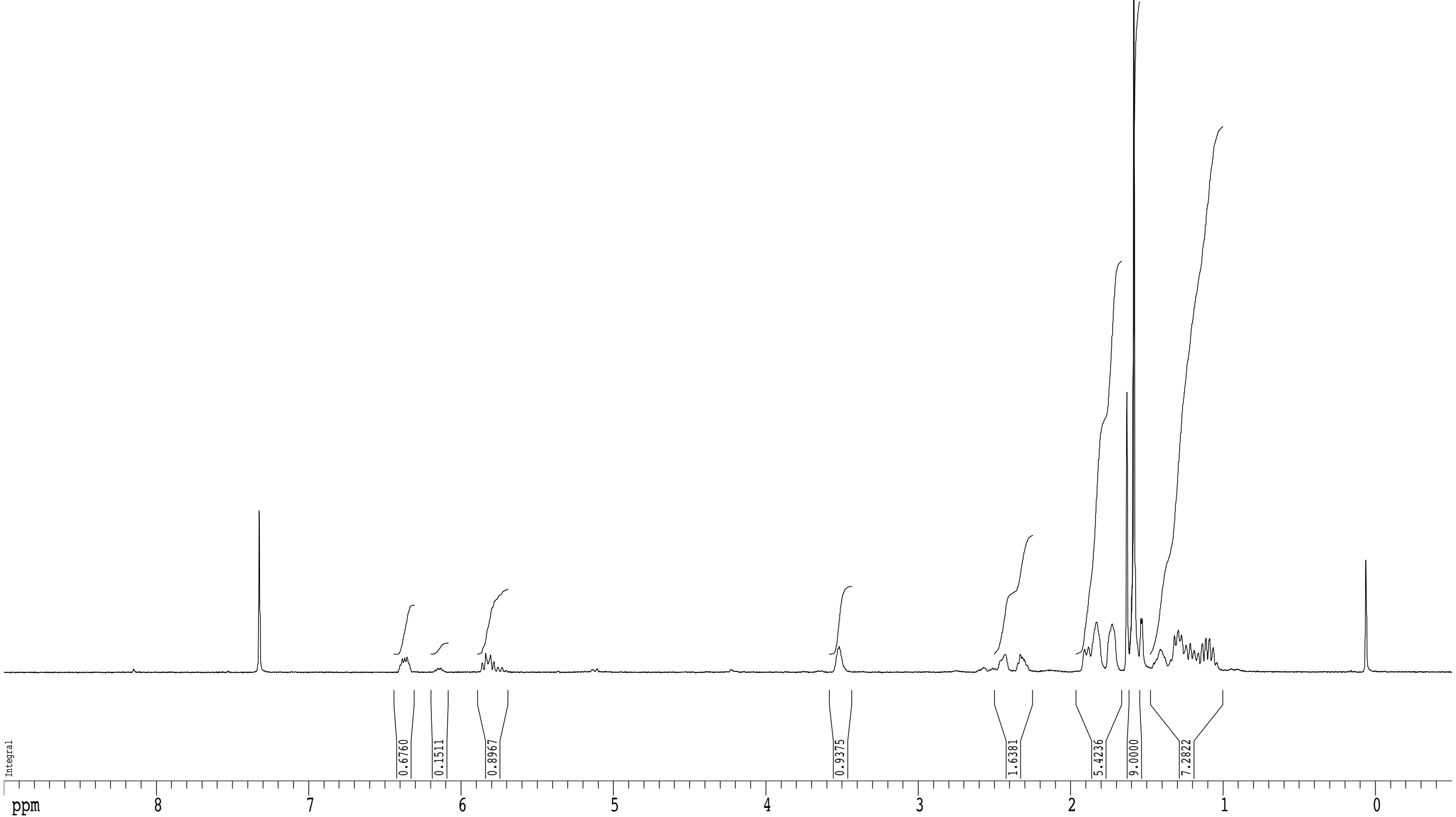


Current Data Parameters
USER mcginnit
NAME tmm-3-168
EXPNO 2
PROCNO 1

F2 - Acquisition Parameters
Date_ 20210618
Time 12.21
INSTRUM gn500
PROBHD 5 mm broadband
PULPROG zg30
TD 81728
SOLVENT CDCl3
NS 8
DS 2
SWH 8012.820 Hz
FIDRES 0.098043 Hz
AQ 5.0998774 sec
RG 2048
DW 62.400 usec
DE 6.00 usec
TE 298.0 K
D1 0.10000000 sec
MCREST 0.00000000 sec
MCWRK 0.01500000 sec

======== CHANNEL f1 ========
NUC1 1H
P1 12.00 usec
PL1 -6.00 dB
SFO1 498.7534913 MHz

F2 - Processing parameters
SI 65536
SF 498.7500000 MHz
WDW EM
SSB 0
LB 0.30 Hz
GB 0
PC 1.00

1D NMR plot parameters
CX 20.00 cm
CY 12.50 cm
F1P 9.000 ppm
F1 4488.75 Hz
F2P -0.500 ppm
F2 -249.38 Hz
PPMCM 0.47500 ppm/cm
HZCM 236.90625 Hz/cm

1H spectrum

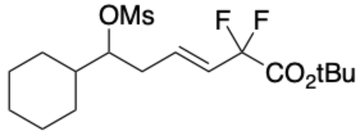


**1b**

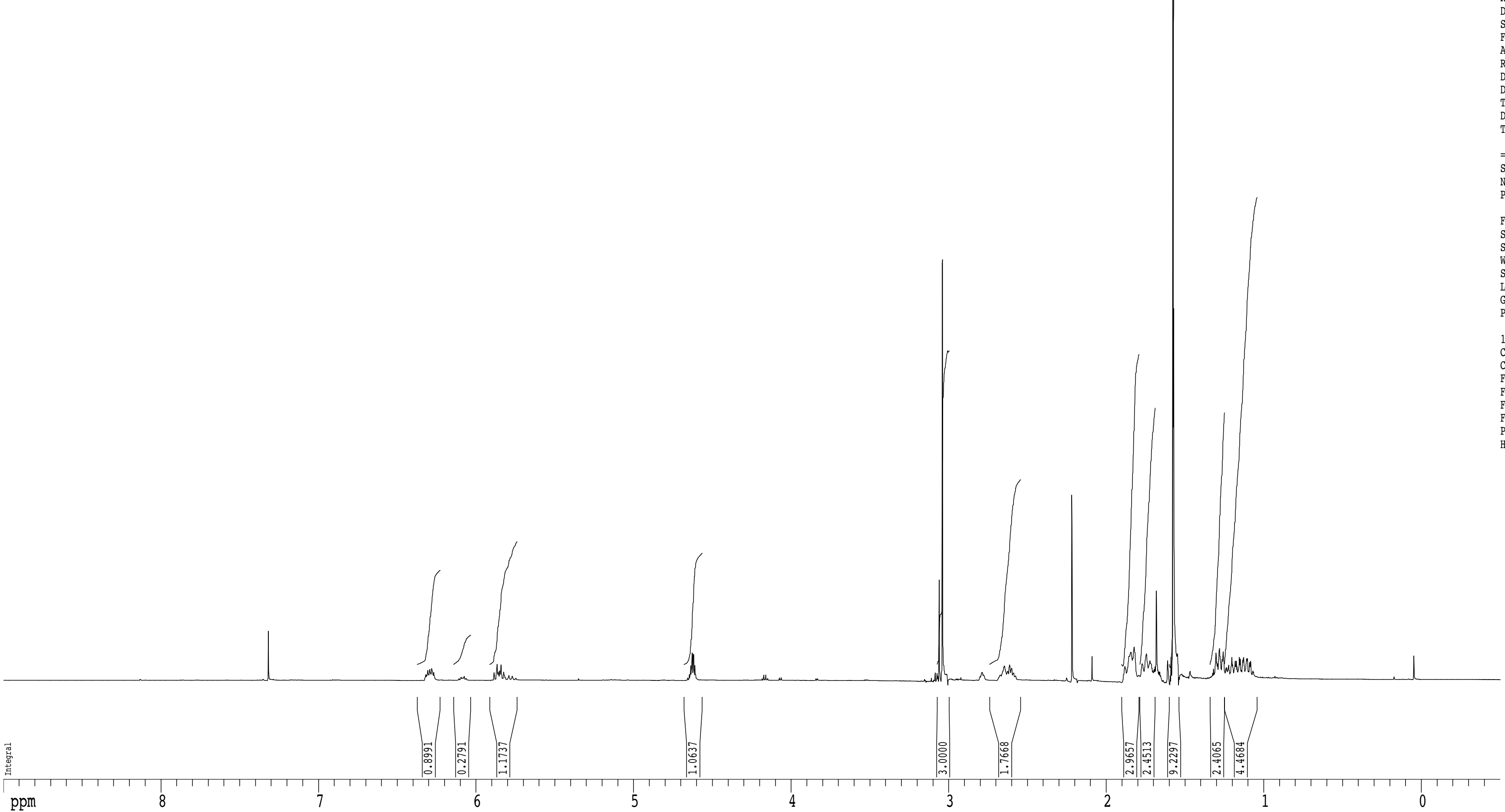


Current Data Parameters
USER mcginnit
NAME tmm-3-175
EXPNO 4
PROCNO 1

F2 - Acquisition Parameters
Date_ 20210712
Time 16.10
INSTRUM av600
PROBHD 5 mm CPBBO BB-
PULPROG zg30
TD 98074
SOLVENT CDCl3
NS 8
DS 2
SWH 9615.385 Hz
FIDRES 0.098042 Hz
AQ 5.0998979 sec
RG 10
DW 52.000 usec
DE 14.23 usec
TE 298.0 K
D1 0.10000000 sec
TD0 1

======== CHANNEL f1 ========
SFO1 600.1342009 MHz
NUC1 1H
P1 9.50 usec

F2 - Processing parameters
SI 65536
SF 600.1300000 MHz
WDW EM
SSB 0
LB 0.30 Hz
GB 0
PC 1.00

1D NMR plot parameters
CX 22.80 cm
CY 15.00 cm
F1P 9.000 ppm
F1 5401.17 Hz
F2P -0.500 ppm
F2 -300.06 Hz
PPMCM 0.41667 ppm/cm
HZCM 250.05418 Hz/cm

13C spectrum

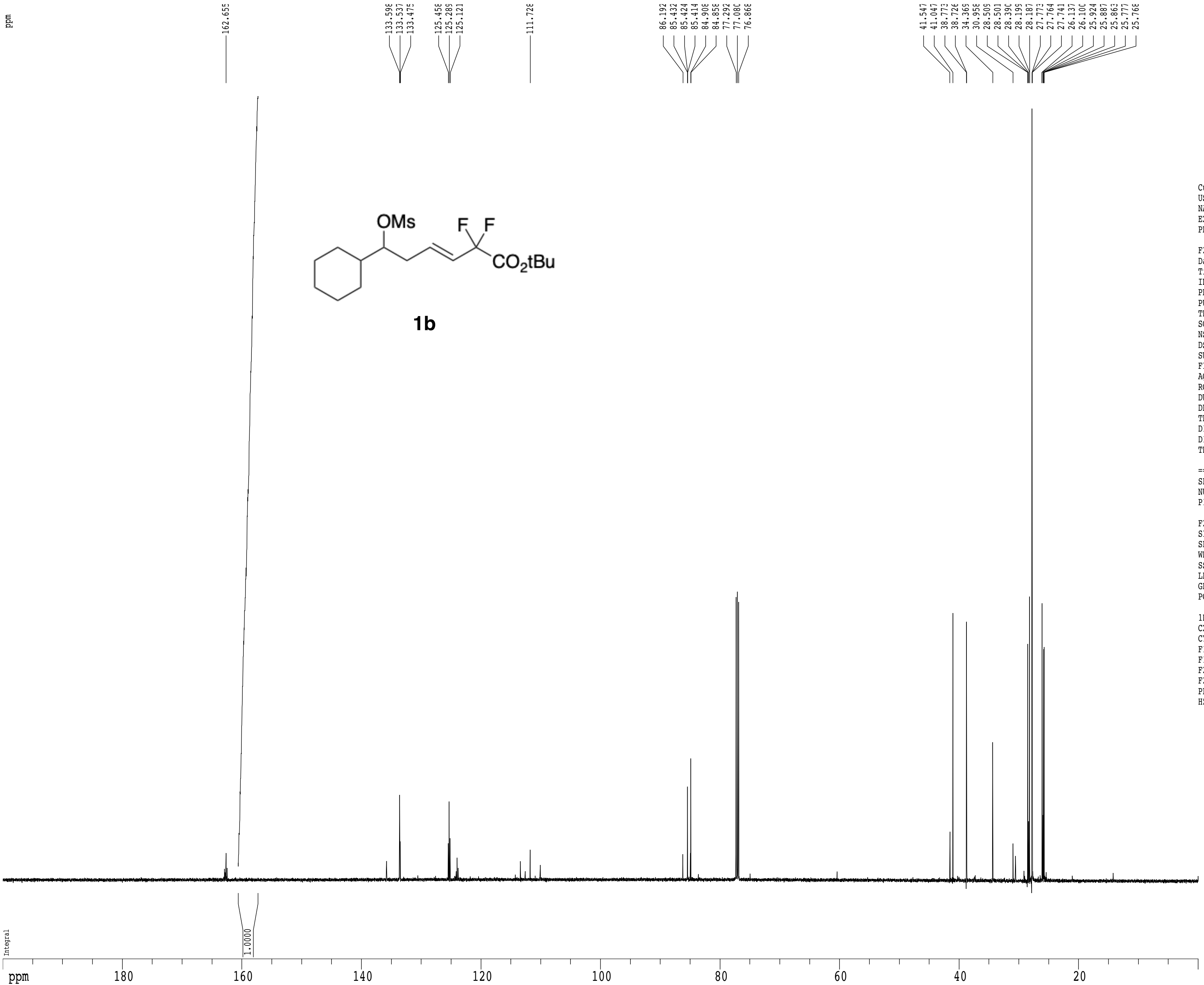


Current Data Parameters
USER mcginnit
NAME tmm-3-175
EXPNO 5
PROCNO 1

F2 - Acquisition Parameters
Date_ 20210712
Time 16.13
INSTRUM av600
PROBHD 5 mm CPBBO BB-
PULPROG zgdc30
TD 65536
SOLVENT CDCl3
NS 208
DS 4
SWH 36231.883 Hz
FIDRES 0.552855 Hz
AQ 0.9044468 sec
RG 2050
DW 13.800 usec
DE 19.63 usec
TE 298.0 K
D1 0.40000001 sec
D11 0.03000000 sec
TD0 1

======== CHANNEL f1 ========
SFO1 150.9194080 MHz
NUC1 13C
P1 10.10 usec

F2 - Processing parameters
SI 65536
SF 150.9028085 MHz
WDW no
SSB 0
LB 0.00 Hz
GB 0
PC 1.00

1D NMR plot parameters
CX 22.80 cm
CY 15.00 cm
F1P 200.000 ppm
F1 30180.56 Hz
F2P 0.000 ppm
F2 0.00 Hz
PPMCM 8.77193 ppm/cm
HZCM 1323.70886 Hz/cm

19F spectrum

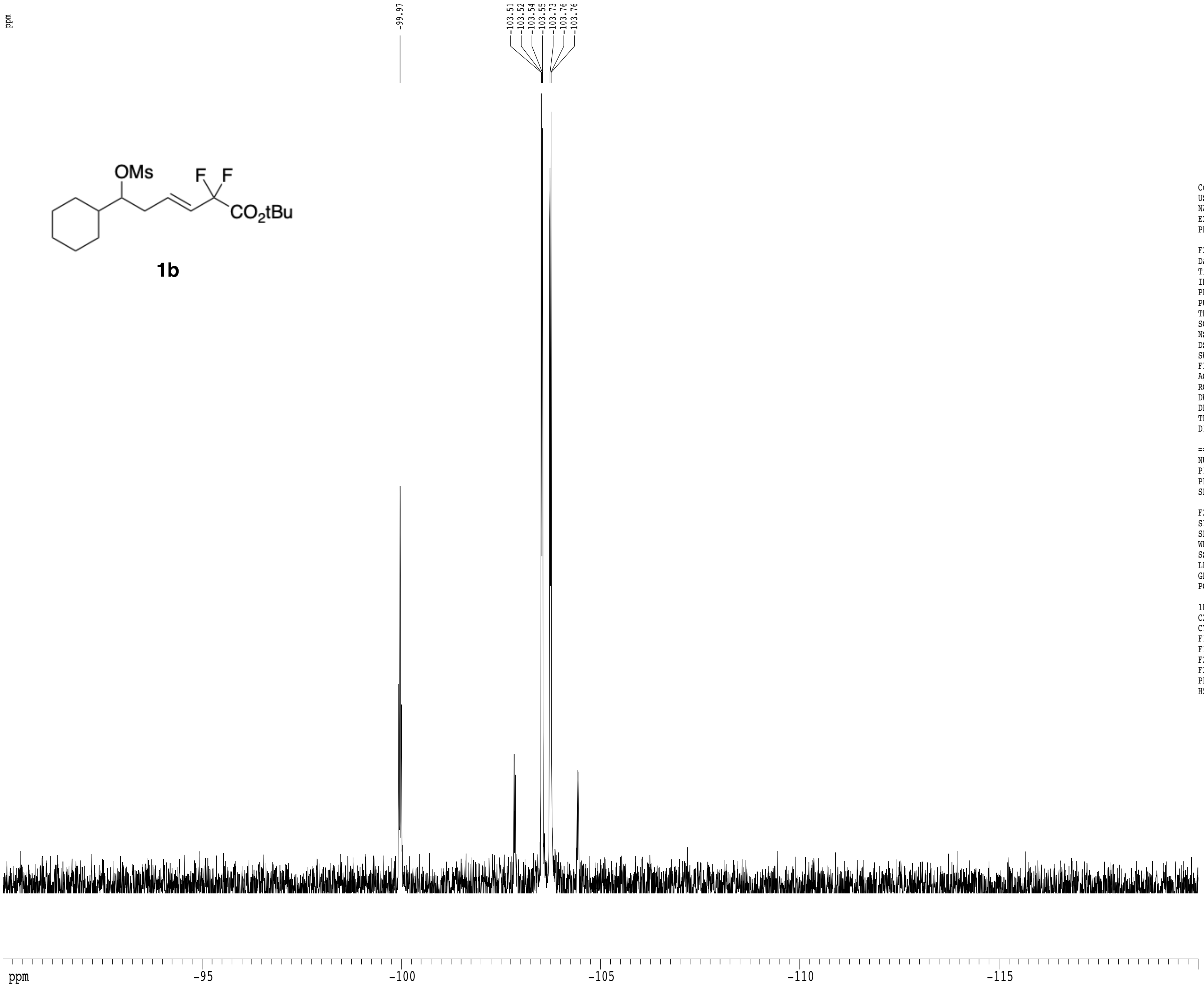


```
Current Data Parameters
USER            mcginnit
NAME           tmm-3-175
EXPNO                  8
PROCNO                 1

F2 - Acquisition Parameters
Date_           20210713
Time               13.57
INSTRUM           drx400
PROBHD   5 mm QNP H/F/P
PULPROG         zgflqn30
TD                 65536
SOLVENT            CDCl3
NS                    52
DS                     2
SWH            75187.969 Hz
FIDRES          1.147277 Hz
AQ             0.4358644 sec
RG                9195.2
DW                 6.650 usec
DE                  9.46 usec
TE                 298.0 K
D1            2.00000000 sec

======== CHANNEL f1 ========
NUC1                 19F
P1                 11.75 usec
PL1                -6.00 dB
SFO1         376.4646491 MHz

F2 - Processing parameters
SI                 65536
SF           376.4984640 MHz
WDW                   EM
SSB                    0
LB                  1.00 Hz
GB                     0
PC                  1.00

1D NMR plot parameters
CX                 22.80 cm
CY                 15.00 cm
F1P              -90.000 ppm
F1             -33884.86 Hz
F2P             -120.000 ppm
F2             -45179.82 Hz
PPMCM            1.31579 ppm/cm
HZCM           495.39273 Hz/cm
```

1H spectrum

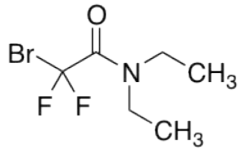

**SI-29**

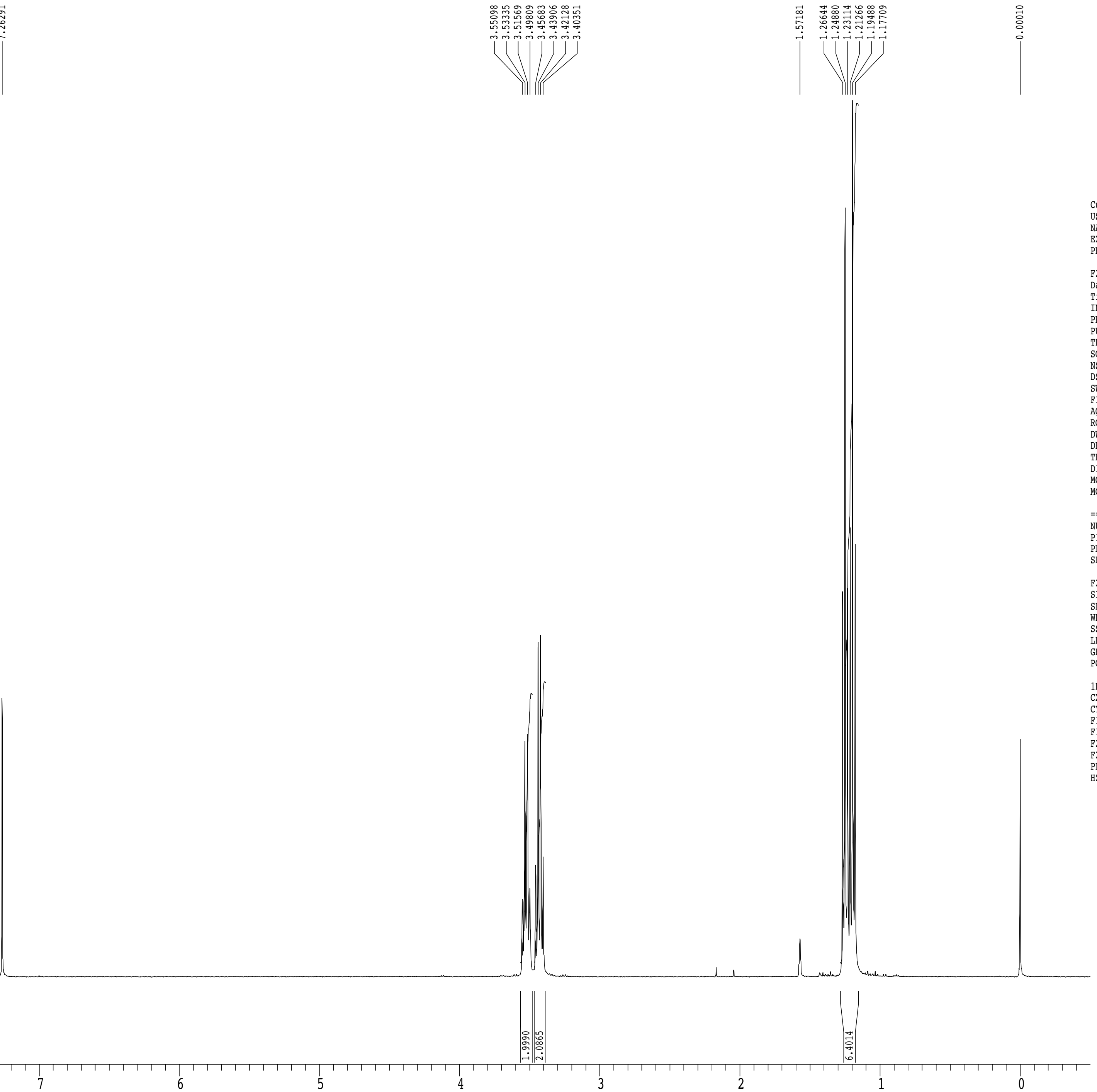


```
Current Data Parameters
USER              linpc2
NAME           pcl-2-058
EXPNO                  1
PROCNO                 1

F2 - Acquisition Parameters
Date_           20210518
Time               11.41
INSTRUM           drx400
PROBHD   5 mm QNP H/F/P
PULPROG             zg30
TD                 65536
SOLVENT            CDCl3
NS                     8
DS                     2
SWH             6410.256 Hz
FIDRES          0.097813 Hz
AQ             5.1118579 sec
RG                 456.1
DW                78.000 usec
DE                  4.50 usec
TE                 298.0 K
D1            0.10000000 sec
MCREST        0.00000000 sec
MCWRK         0.01500000 sec

======== CHANNEL f1 ========
NUC1                  1H
P1                 12.00 usec
PL1                -1.60 dB
SFO1         400.1328009 MHz

F2 - Processing parameters
SI                 65536
SF           400.1300204 MHz
WDW                   EM
SSB                    0
LB                  0.30 Hz
GB                     0
PC                  2.00

1D NMR plot parameters
CX                 22.80 cm
CY                 15.00 cm
F1P                9.000 ppm
F1               3601.17 Hz
F2P               -0.500 ppm
F2               -200.06 Hz
PPMCM            0.41667 ppm/cm
HZCM           166.72086 Hz/cm
```

1H spectrum

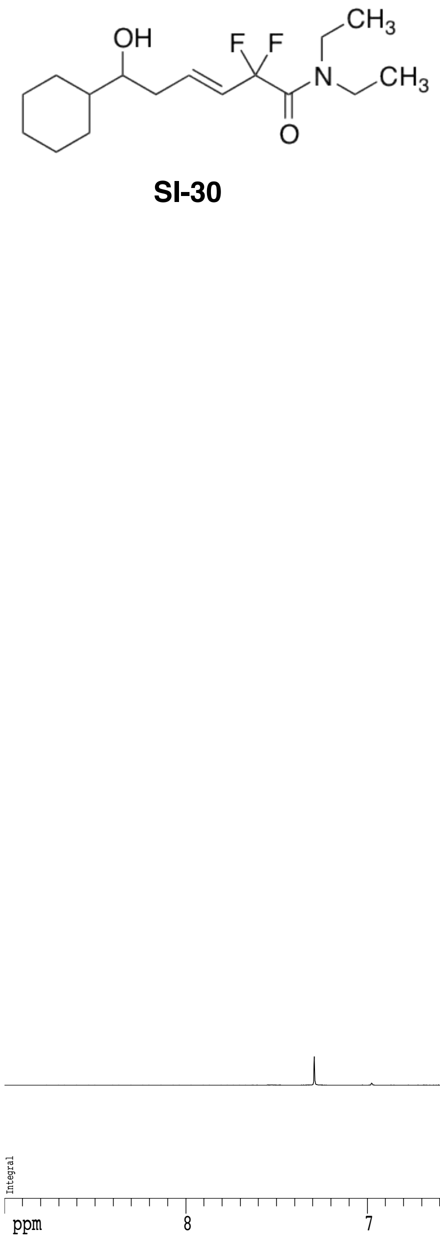


**SI-30**

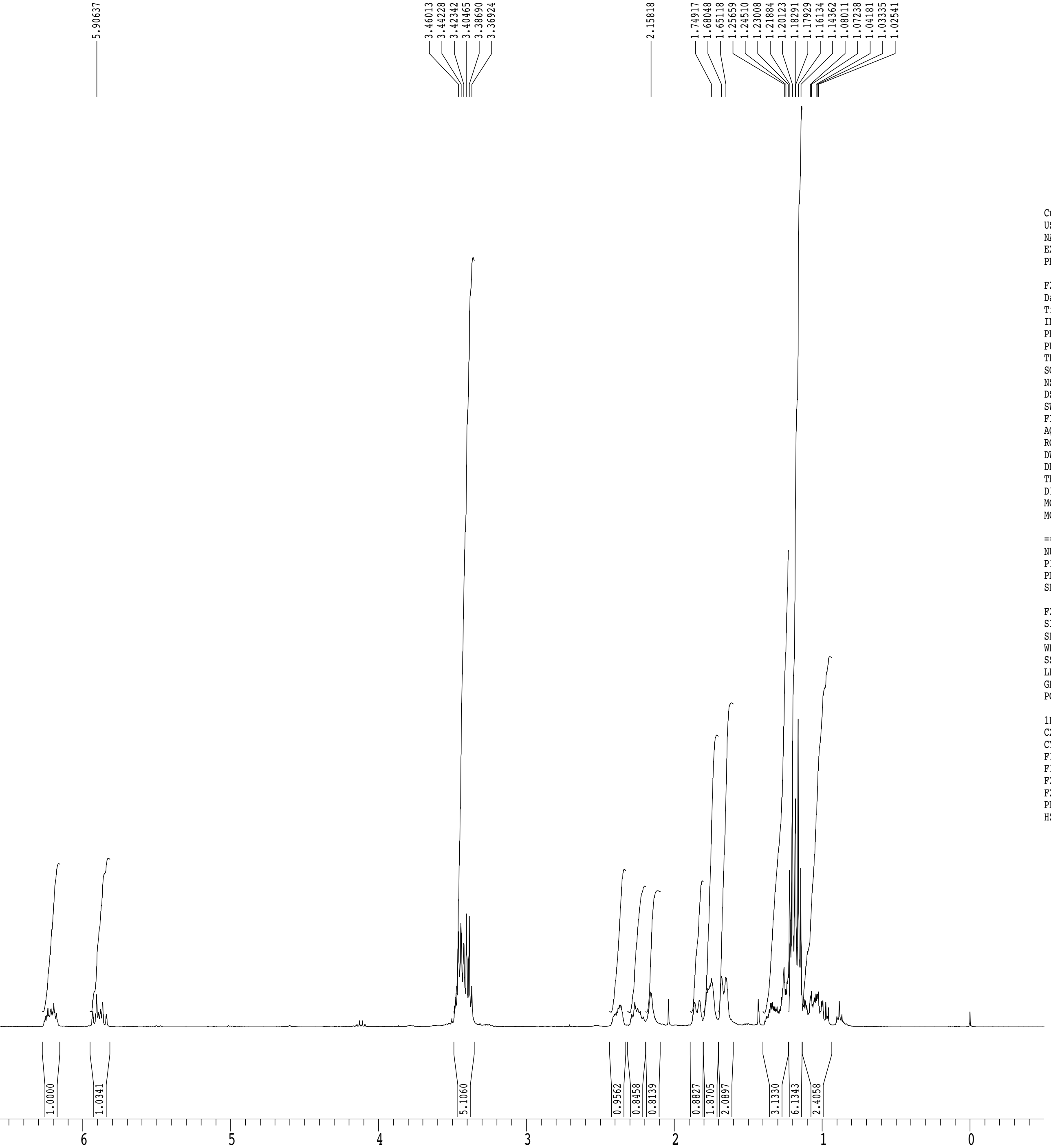


Current Data Parameters
USER linpc2
NAME pcl-2-117
EXPNO 3
PROCNO 1

F2 - Acquisition Parameters
Date_ 20210722
Time 13.33
INSTRUM drx400
PROBHD 5 mm QNP H/F/P
PULPROG zg30
TD 65536
SOLVENT CDCl3
NS 8
DS 2
SWH 6410.256 Hz
FIDRES 0.097813 Hz
AQ 5.1118579 sec
RG 57
DW 78.000 usec
DE 4.50 usec
TE 298.0 K
D1 0.10000000 sec
MCREST 0.00000000 sec
MCWRK 0.01500000 sec

======== CHANNEL f1 ========
NUC1 1H
P1 12.00 usec
PL1 -1.60 dB
SFO1 400.1328009 MHz

F2 - Processing parameters
SI 65536
SF 400.1300088 MHz
WDW EM
SSB 0
LB 0.30 Hz
GB 0
PC 2.00

1D NMR plot parameters
CX 22.80 cm
CY 5.00 cm
F1P 9.000 ppm
F1 3601.17 Hz
F2P -0.500 ppm
F2 -200.06 Hz
PPMCM 0.41667 ppm/cm
HZCM 166.72084 Hz/cm

1H spectrum

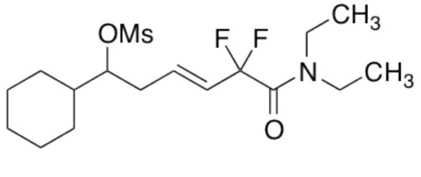


**1c**

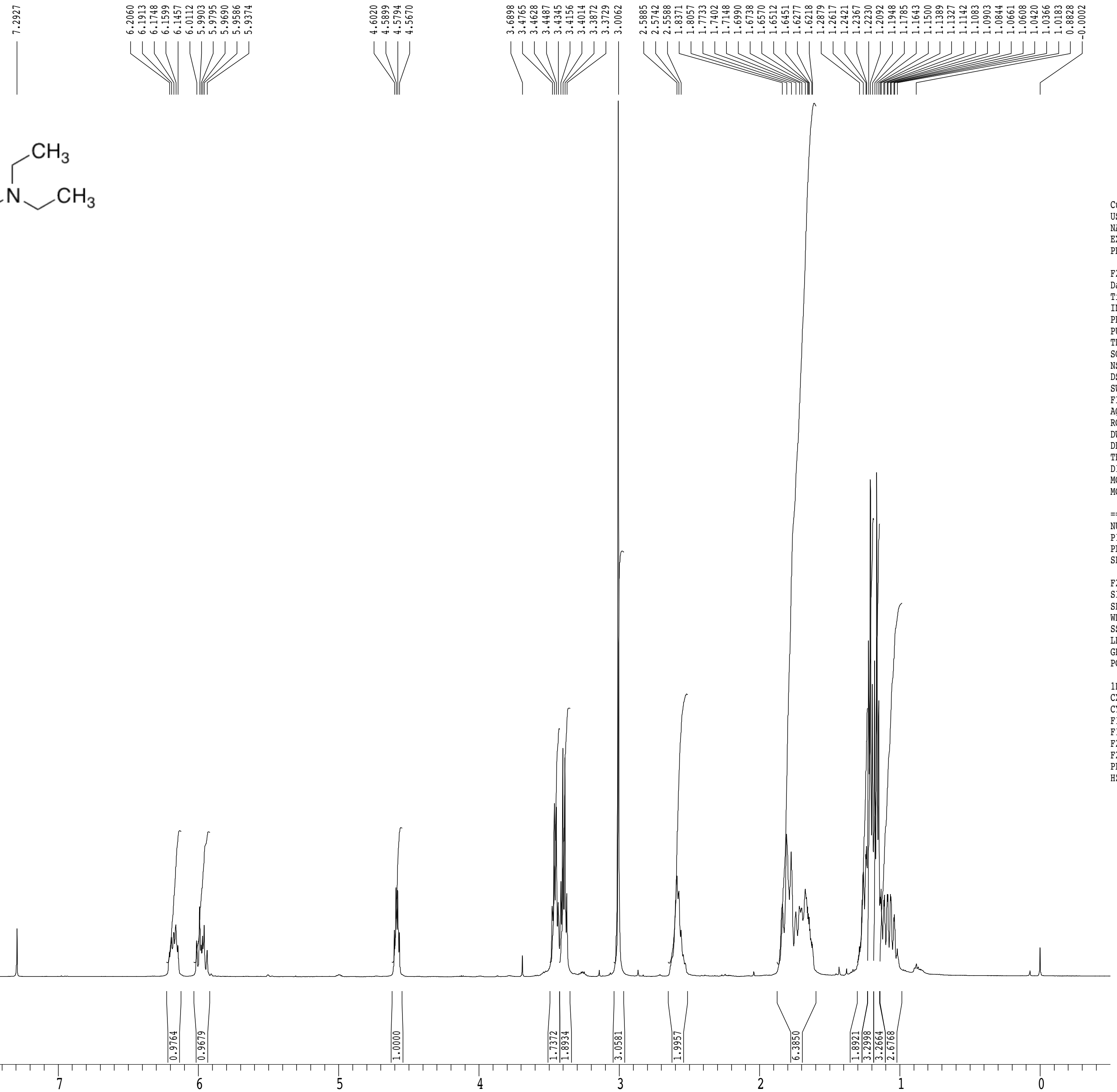


Current Data Parameters
USER linpc2
NAME pcl-2-120
EXPNO 3
PROCNO 1

F2 - Acquisition Parameters
Date_ 20210724
Time 12.43
INSTRUM cryo500
PROBHD 5 mm CPTCI 1H-
PULPROG zg30
TD 81728
SOLVENT CDCl3
NS 8
DS 2
SWH 8012.820 Hz
FIDRES 0.098043 Hz
AQ 5.0998774 sec
RG 4
DW 62.400 usec
DE 6.00 usec
TE 298.0 K
D1 0.10000000 sec
MCREST 0.00000000 sec
MCWRK 0.01500000 sec

======== CHANNEL f1 ========
NUC1 1H
P1 9.75 usec
PL1 1.60 dB
SFO1 500.2235015 MHz

F2 - Processing parameters
SI 65536
SF 500.2200144 MHz
WDW EM
SSB 0
LB 0.30 Hz
GB 0
PC 1.00

1D NMR plot parameters
CX 22.80 cm
CY 15.00 cm
F1P 9.000 ppm
F1 4501.98 Hz
F2P -0.500 ppm
F2 -250.11 Hz
PPMCM 0.41667 ppm/cm
HZCM 208.42500 Hz/cm

Z-restored spin-echo 13C spectrum with 1H decoupling

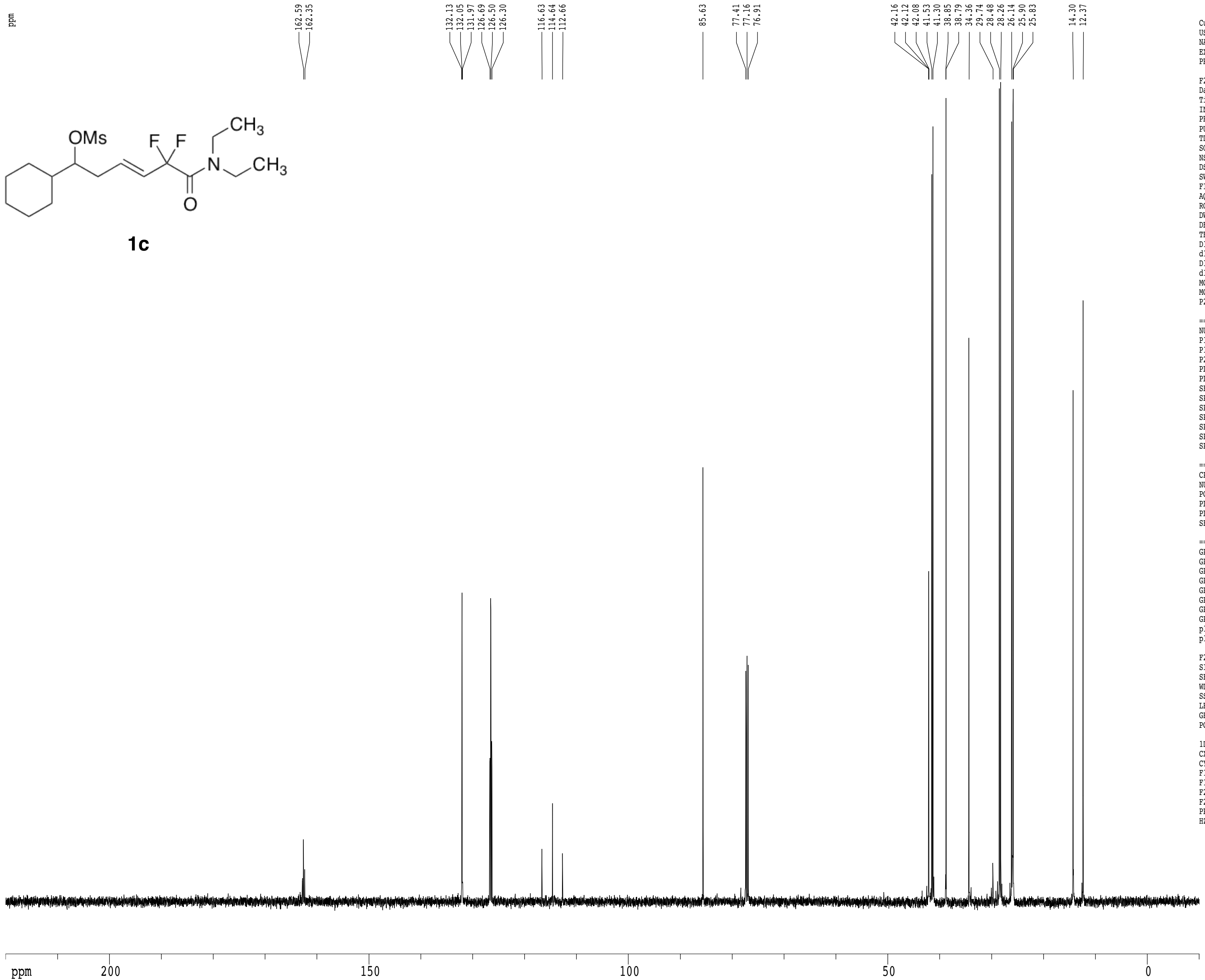


```
Current Data Parameters
USER              linpc2
NAME           pcl-2-120
EXPNO                  4
PROCNO                 1

F2 - Acquisition Parameters
Date_           20210724
Time               12.46
INSTRUM          cryo500
PROBHD   5 mm CPTCI 1H-
PULPROG  SpinEchopg30gp2.prd
TD                 65536
SOLVENT            CDCl3
NS                   120
DS                    16
SWH            30303.031 Hz
FIDRES          0.462388 Hz
AQ             1.0813940 sec
RG                7298.2
DW                16.500 usec
DE                  6.00 usec
TE                 298.0 K
D1            0.25000000 sec
d11           0.03000000 sec
D16           0.00020000 sec
d17           0.00019600 sec
MCREST        0.00000000 sec
MCWRK         0.01500000 sec
P2                 37.70 usec

======== CHANNEL f1 ========
NUC1                 13C
P1                 18.85 usec
P12              2000.00 usec
P20               500.00 usec
PL0               120.00 dB
PL1                -1.00 dB
SFO1         125.7942548 MHz
SP2                 1.55 dB
SP4                 1.55 dB
SPNAM2       Crp60comp.4
SPNAM4   Crp60,0.5,20.1
SPOFF2              0.00 Hz
SPOFF4              0.00 Hz

======== CHANNEL f2 ========
CPDPRG2          waltz16
NUC2                  1H
PCPD2             100.00 usec
PL2                 1.60 dB
PL12               22.00 dB
SFO2         500.2225011 MHz

====== GRADIENT CHANNEL =====
GPNAM1         SINE.100
GPNAM2         SINE.100
GPX1                0.00 %
GPX2                0.00 %
GPY1                0.00 %
GPY2                0.00 %
GPZ1               30.00 %
GPZ2               50.00 %
p15               500.00 usec
p16              1000.00 usec

F2 - Processing parameters
SI                 65536
SF           125.7804145 MHz
WDW                   EM
SSB                    0
LB                  1.00 Hz
GB                     0
PC                  2.00

1D NMR plot parameters
CX                 22.80 cm
CY                 15.65 cm
F1P              220.000 ppm
F1              27671.69 Hz
F2P              -10.000 ppm
F2              -1257.80 Hz
PPMCM           10.08772 ppm/cm
HZCM          1268.83752 Hz/cm
```

19F spectrum

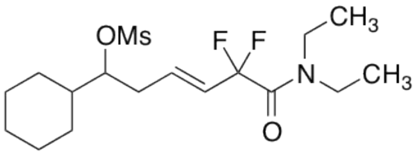


**1c**

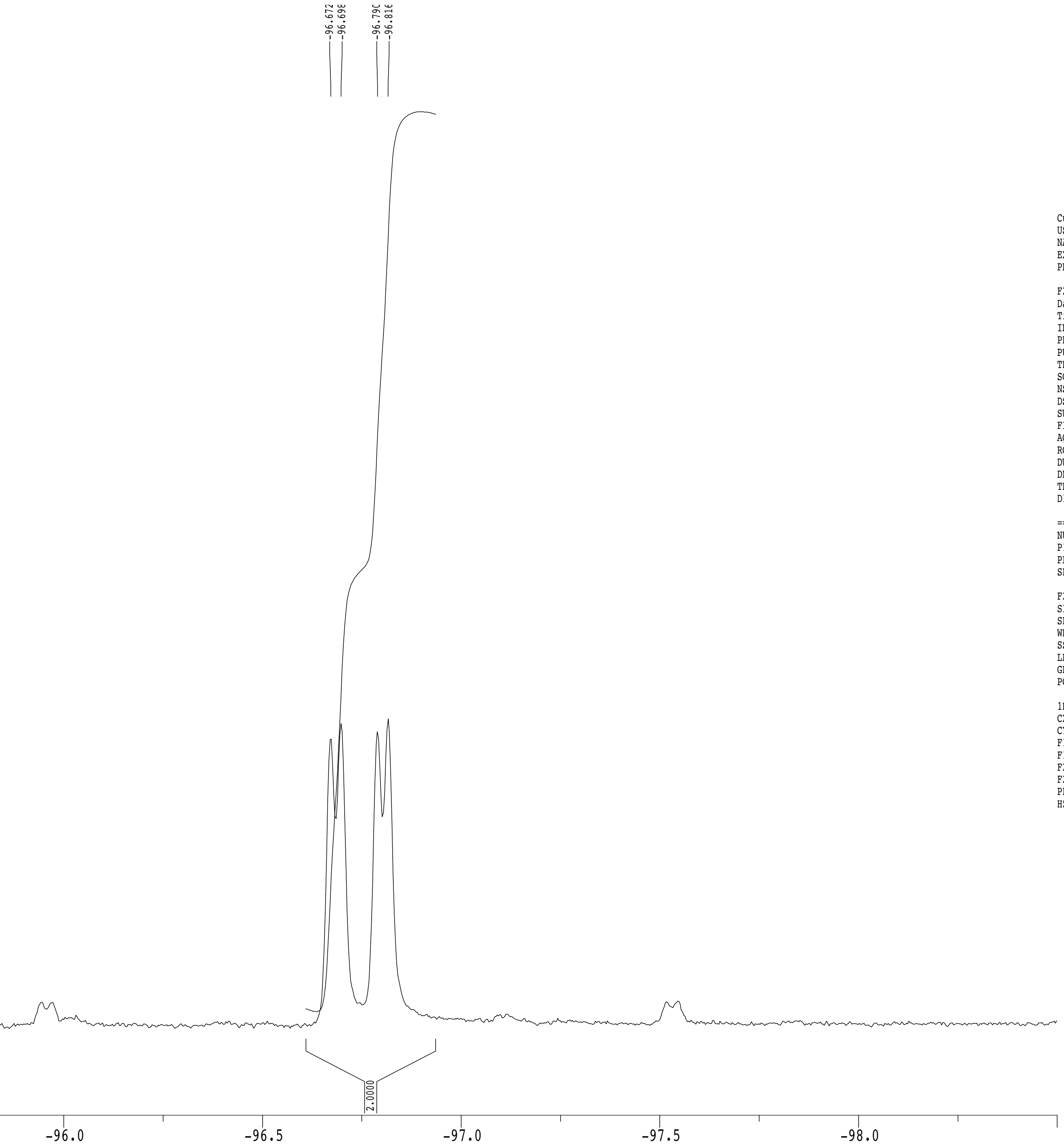


```
Current Data Parameters
USER              linpc2
NAME            pcl-2-120
EXPNO                  2
PROCNO                 1

F2 - Acquisition Parameters
Date_           20210723
Time               15.22
INSTRUM           drx400
PROBHD   5 mm QNP H/F/P
PULPROG        zgflqn30
TD                 65536
SOLVENT            CDCl3
NS                    40
DS                     2
SWH           75187.969 Hz
FIDRES         1.147277 Hz
AQ            0.4358644 sec
RG               10321.3
DW                 6.650 usec
DE                  9.46 usec
TE                 298.0 K
D1            2.00000000 sec

======== CHANNEL f1 ========
NUC1                 19F
P1                 11.75 usec
PL1                -6.00 dB
SFO1         376.4646491 MHz

F2 - Processing parameters
SI                 65536
SF           376.4984640 MHz
WDW                   EM
SSB                    0
LB                  1.00 Hz
GB                     0
PC                  1.00

1D NMR plot parameters
CX                 22.80 cm
CY                  5.00 cm
F1P              -95.000 ppm
F1             -35767.36 Hz
F2P              -98.500 ppm
F2             -37085.10 Hz
PPMCM            0.15351 ppm/cm
HZCM            57.79582 Hz/cm
```

1H spectrum

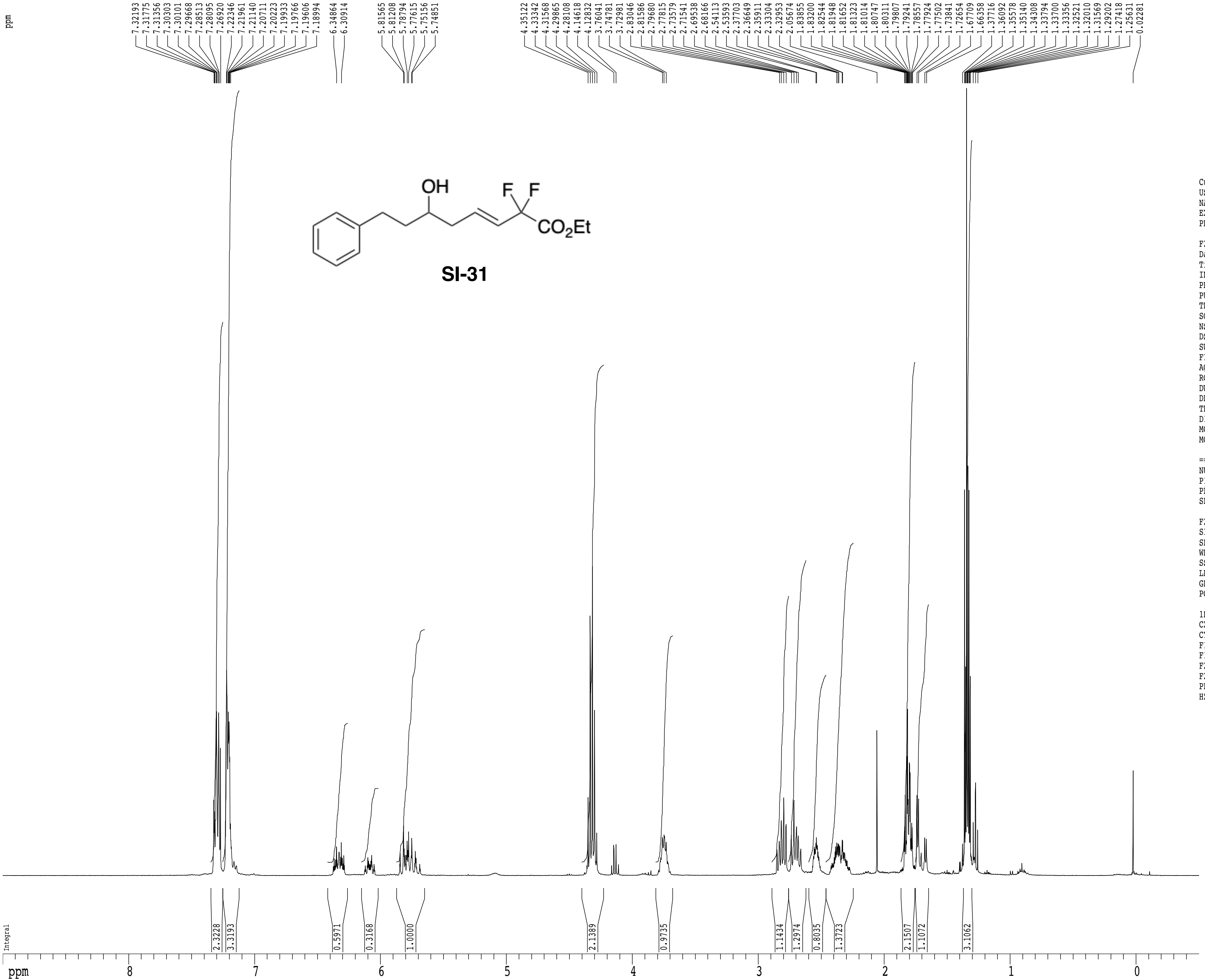


**SI-31**

```
Current Data Parameters
USER            sanforda
NAME      ABS-4-063-f37
EXPNO                  1
PROCNO                 1

F2 - Acquisition Parameters
Date_          20210219
Time              10.28
INSTRUM          drx400
PROBHD   5 mm QNP H/F/P
PULPROG            zg30
TD                38460
SOLVENT           CDCl3
NS                    8
DS                    2
SWH            6410.256 Hz
FIDRES         0.166673 Hz
AQ            2.9999299 sec
RG                 80.6
DW               78.000 usec
DE                 4.50 usec
TE                298.0 K
D1           0.10000000 sec
MCREST       0.00000000 sec
MCWRK        0.01500000 sec

======== CHANNEL f1 ========
NUC1                 1H
P1                12.00 usec
PL1               -1.60 dB
SFO1        400.1328009 MHz

F2 - Processing parameters
SI                65536
SF          400.1300175 MHz
WDW                  no
SSB                   0
LB                 0.00 Hz
GB                    0
PC                 2.00

1D NMR plot parameters
CX                22.80 cm
CY                15.00 cm
F1P               9.000 ppm
F1              3601.17 Hz
F2P              -0.500 ppm
F2              -200.06 Hz
PPMCM           0.41667 ppm/cm
HZCM          166.72084 Hz/cm
```

**SFC Chiracel OD-H, 4% IPA/$CO_2$, 2.0 mL/min**

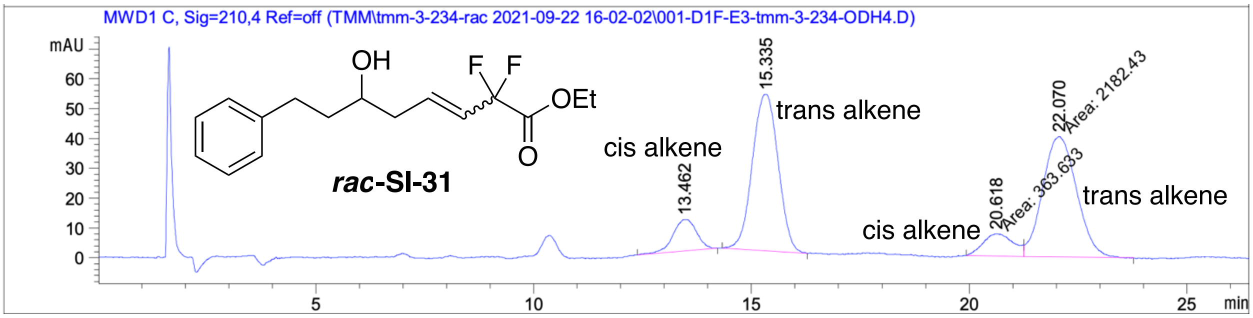


Signal 3: MWD1 C, Sig=210,4 Ref=off

| Peak # | RetTime [min] | Type | Width [min] | Area [mAU*s] | Height [mAU] | Area % |
|---|---|---|---|---|---|---|
| 1 | 13.462 | VV R | 0.4693 | 402.17569 | 10.58358 | 7.8756 |
| 2 | 15.335 | VV R | 0.5456 | 2158.38843 | 52.49989 | 42.2665 |
| 3 | 20.618 | MF | 0.8147 | 363.63287 | 7.43908 | 7.1208 |
| 4 | 22.070 | FM | 0.9031 | 2182.42529 | 40.27704 | 42.7372 |

Totals : 5106.62228 110.79958

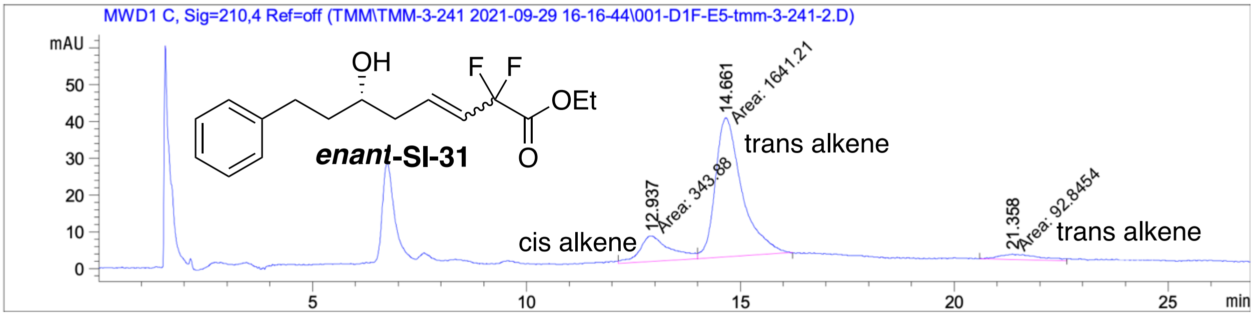


Signal 3: MWD1 C, Sig=210,4 Ref=off

| Peak # | RetTime [min] | Type | Width [min] | Area [mAU*s] | Height [mAU] | Area % |
|---|---|---|---|---|---|---|
| 1 | 12.937 | MF | 0.8330 | 343.87991 | 6.88052 | 16.5491 |
| 2 | 14.661 | FM | 0.7241 | 1641.20862 | 37.77579 | 78.9827 |
| 3 | 21.358 | MM | 1.1007 | 92.84538 | 1.40581 | 4.4682 |

Totals : 2077.93391 46.06213

1H spectrum

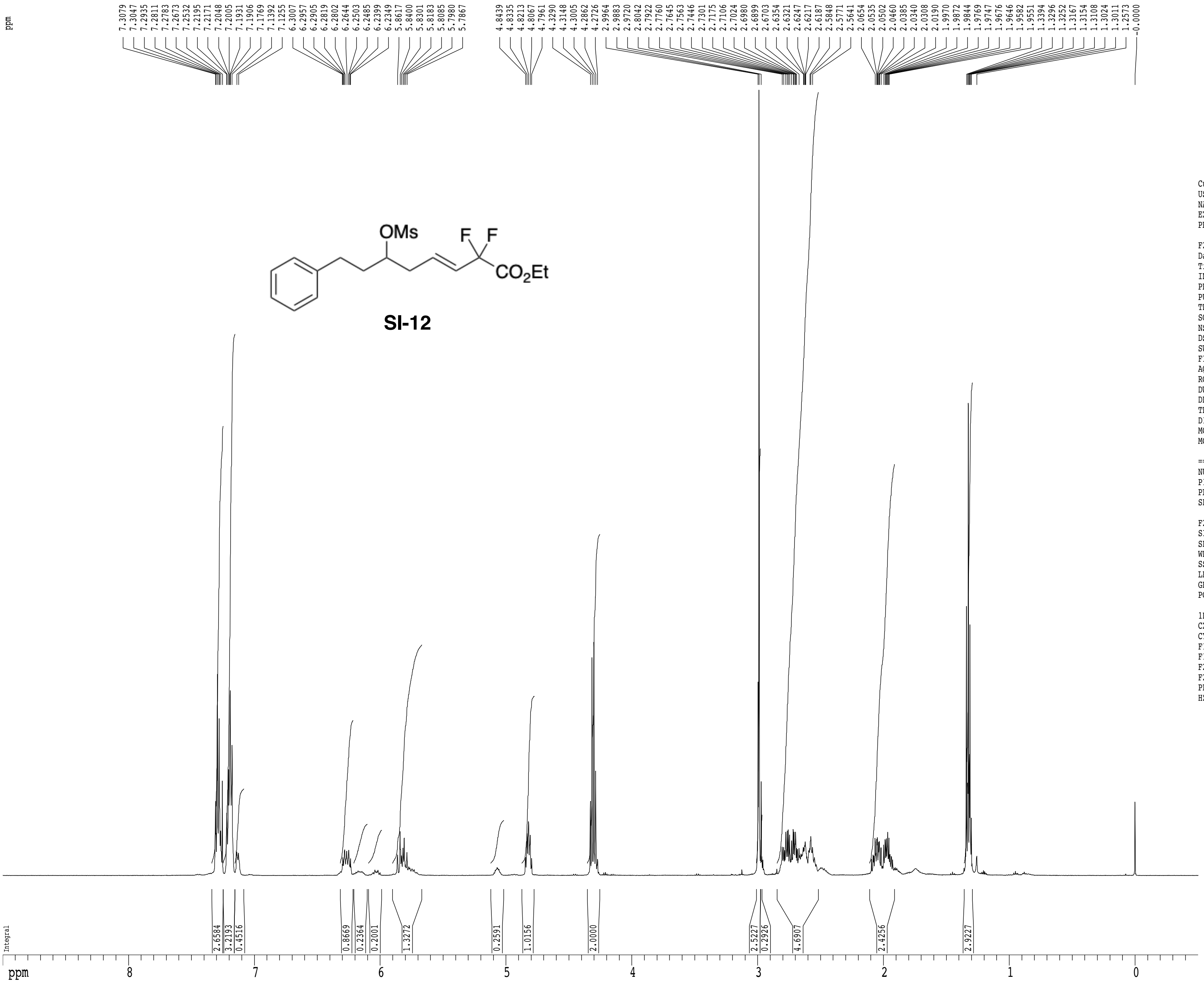


Current Data Parameters
USER sanforda
NAME ABS-3-181-proton
EXPNO 1
PROCNO 1

F2 - Acquisition Parameters
Date_ 20200714
Time 15.33
INSTRUM cryo500
PROBHD 5 mm CPTCI 1H-
PULPROG zg30
TD 81728
SOLVENT CDCl3
NS 8
DS 2
SWH 8012.820 Hz
FIDRES 0.098043 Hz
AQ 5.0998774 sec
RG 5.7
DW 62.400 usec
DE 6.00 usec
TE 298.0 K
D1 0.10000000 sec
MCREST 0.00000000 sec
MCWRK 0.01500000 sec

======== CHANNEL f1 ========
NUC1 1H
P1 7.50 usec
PL1 1.60 dB
SFO1 500.2235015 MHz

F2 - Processing parameters
SI 65536
SF 500.2200344 MHz
WDW no
SSB 0
LB 0.00 Hz
GB 0
PC 1.00

1D NMR plot parameters
CX 22.80 cm
CY 15.00 cm
F1P 9.000 ppm
F1 4501.98 Hz
F2P -0.500 ppm
F2 -250.11 Hz
PPMCM 0.41667 ppm/cm
HZCM 208.42502 Hz/cm

Z-restored spin-echo 13C spectrum with 1H decoupling

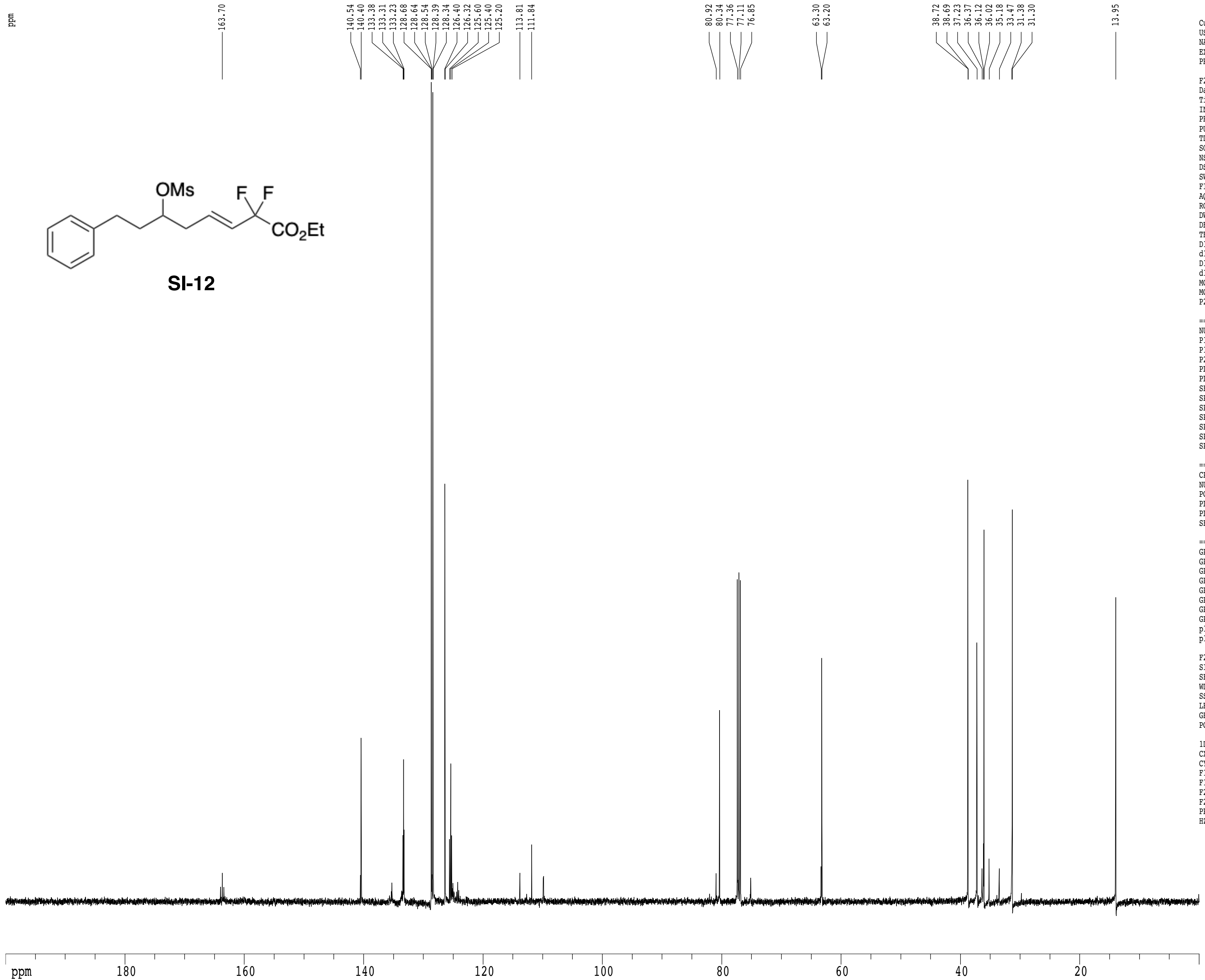


```
Current Data Parameters
USER            sanforda
NAME     ABS-3-181-carbon
EXPNO                  1
PROCNO                 1

F2 - Acquisition Parameters
Date_           20200714
Time               15.35
INSTRUM          cryo500
PROBHD   5 mm CPTCI 1H-
PULPROG  SpinEchopg30gp2.prd
TD                 65536
SOLVENT            CDCl3
NS                   352
DS                    16
SWH            30303.031 Hz
FIDRES          0.462388 Hz
AQ             1.0813940 sec
RG                7298.2
DW                16.500 usec
DE                  6.00 usec
TE                 298.0 K
D1            0.25000000 sec
d11           0.03000000 sec
D16           0.00020000 sec
d17           0.00019600 sec
MCREST        0.00000000 sec
MCWRK         0.01500000 sec
P2                 33.10 usec

======== CHANNEL f1 ========
NUC1                 13C
P1                 16.55 usec
P12              2000.00 usec
P20               500.00 usec
PL0               120.00 dB
PL1                -1.00 dB
SFO1         125.7942548 MHz
SP2                 2.70 dB
SP4                 2.70 dB
SPNAM2      Crp60comp.4
SPNAM4   Crp60,0.5,20.1
SPOFF2              0.00 Hz
SPOFF4              0.00 Hz

======== CHANNEL f2 ========
CPDPRG2          waltz16
NUC2                  1H
PCPD2             100.00 usec
PL2                 1.60 dB
PL12               23.54 dB
SFO2         500.2225011 MHz

====== GRADIENT CHANNEL =====
GPNAM1          SINE.100
GPNAM2          SINE.100
GPX1                0.00 %
GPX2                0.00 %
GPY1                0.00 %
GPY2                0.00 %
GPZ1               30.00 %
GPZ2               50.00 %
p15               500.00 usec
p16              1000.00 usec

F2 - Processing parameters
SI                 65536
SF           125.7804190 MHz
WDW                   EM
SSB                    0
LB                  1.00 Hz
GB                     0
PC                  2.00

1D NMR plot parameters
CX                 22.80 cm
CY                 15.65 cm
F1P              200.000 ppm
F1              25156.08 Hz
F2P                0.000 ppm
F2                  0.00 Hz
PPMCM            8.77193 ppm/cm
HZCM          1103.33704 Hz/cm
```

19F spectrum

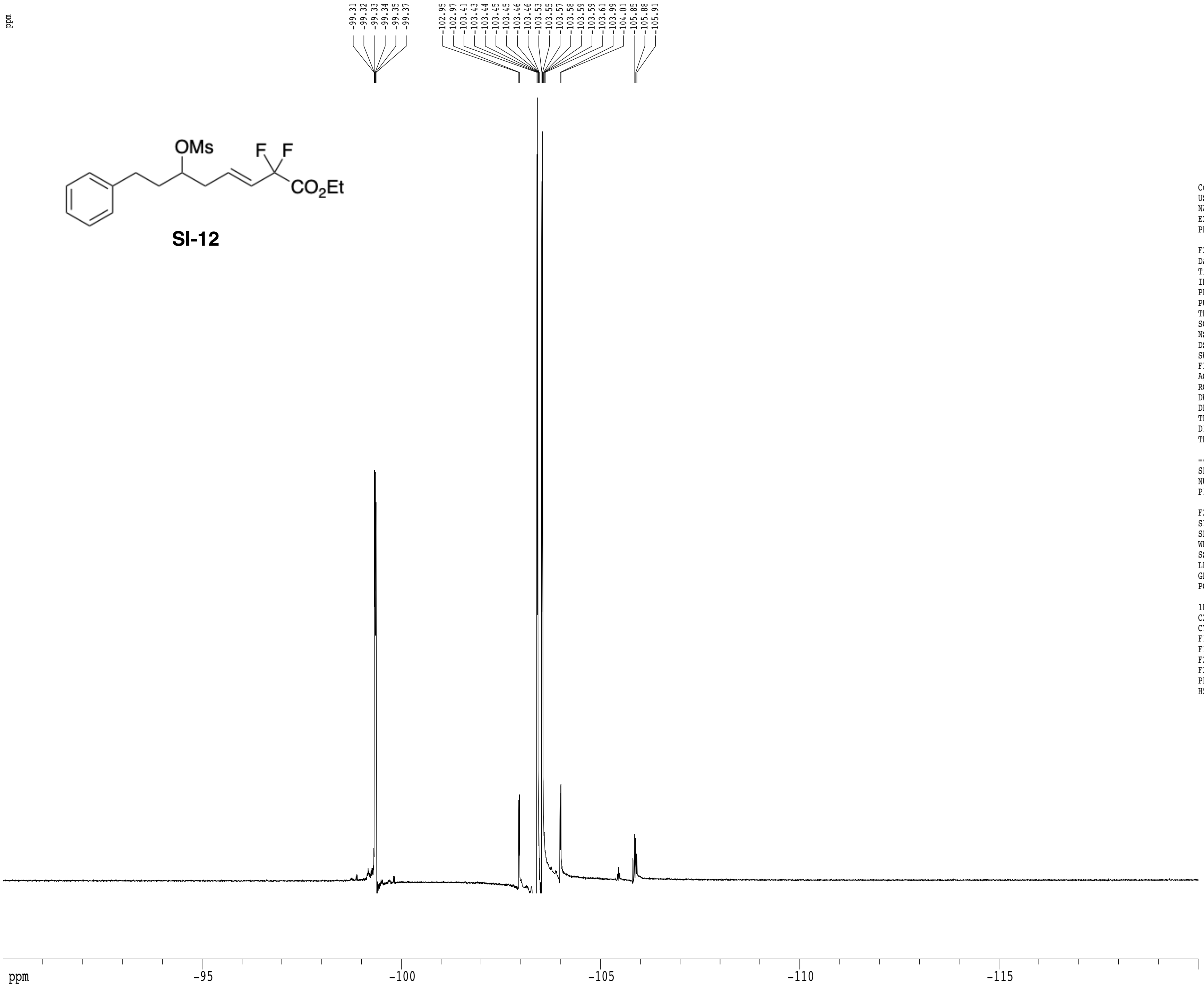


```
Current Data Parameters
USER           sanforda
NAME        ABS-4-050-f
EXPNO                 1
PROCNO                1

F2 - Acquisition Parameters
Date_          20210212
Time              12.39
INSTRUM           av600
PROBHD   5 mm CPBBO BB-
PULPROG        zgflqn30
TD               131072
SOLVENT           CDCl3
NS                   16
DS                    2
SWH          178571.422 Hz
FIDRES         1.362392 Hz
AQ            0.3670516 sec
RG                  575
DW                2.800 usec
DE                18.00 usec
TE                298.2 K
D1           3.00000000 sec
TD0                   1

======== CHANNEL f1 ========
SFO1        564.6299196 MHz
NUC1                19F
P1                18.25 usec

F2 - Processing parameters
SI               131072
SF          564.6863858 MHz
WDW                  no
SSB                   0
LB                 0.00 Hz
GB                    0
PC                 1.00

1D NMR plot parameters
CX                22.80 cm
CY                15.00 cm
F1P             -90.000 ppm
F1             -50821.78 Hz
F2P            -120.000 ppm
F2             -67762.37 Hz
PPMCM           1.31579 ppm/cm
HZCM          743.00842 Hz/cm
```

1H spectrum

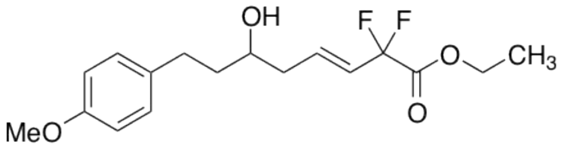


**SI-32**

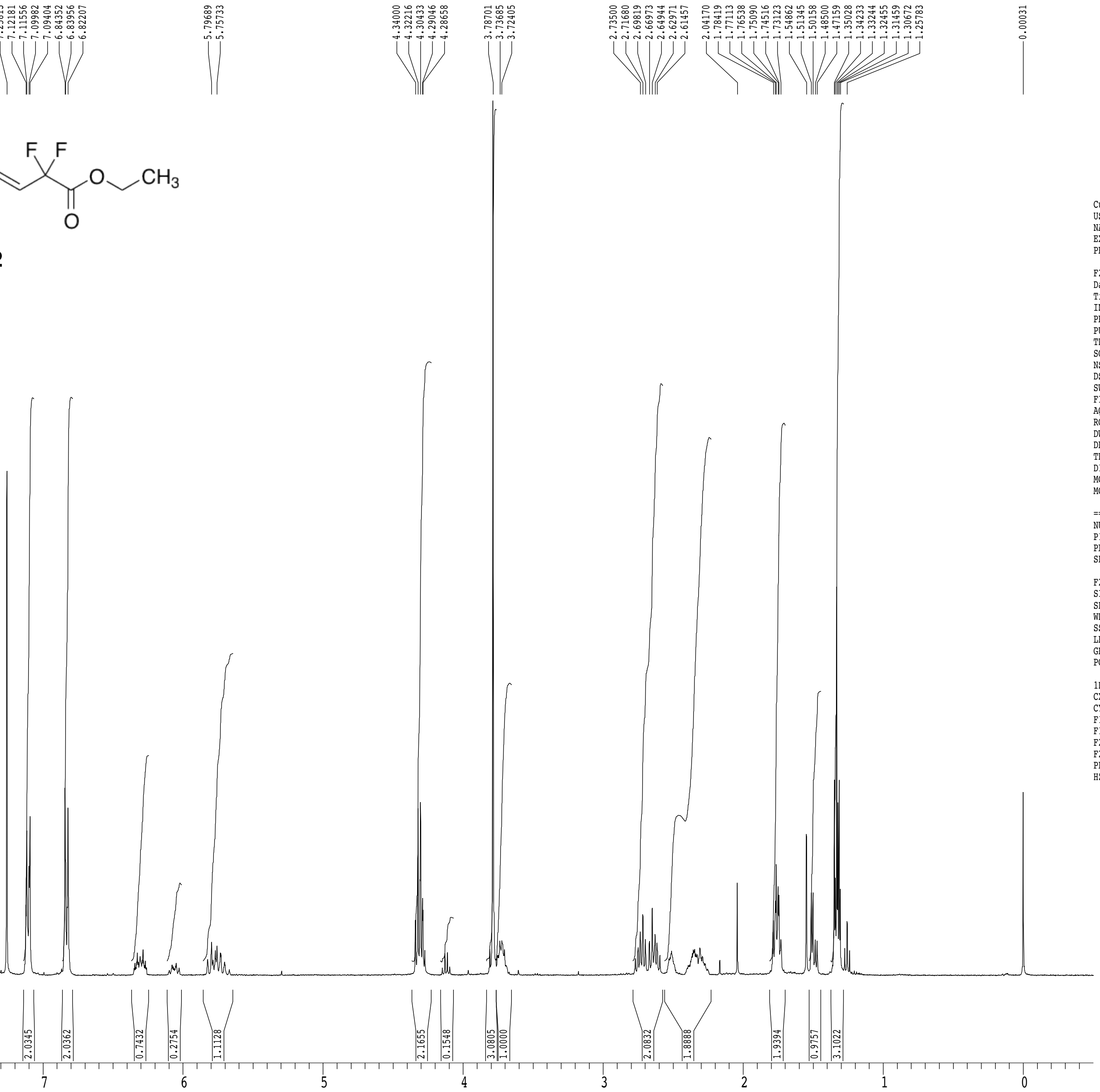


```
Current Data Parameters
USER              linpc2
NAME          pcl-2-088
EXPNO                  2
PROCNO                 1

F2 - Acquisition Parameters
Date_          20210629
Time              15.43
INSTRUM          drx400
PROBHD   5 mm QNP H/F/P
PULPROG            zg30
TD                65536
SOLVENT           CDCl3
NS                    8
DS                    2
SWH            6410.256 Hz
FIDRES         0.097813 Hz
AQ            5.1118579 sec
RG                574.7
DW               78.000 usec
DE                 4.50 usec
TE                298.0 K
D1           0.10000000 sec
MCREST       0.00000000 sec
MCWRK        0.01500000 sec

======== CHANNEL f1 ========
NUC1                 1H
P1                12.00 usec
PL1               -1.60 dB
SFO1        400.1328009 MHz

F2 - Processing parameters
SI                65536
SF          400.1300221 MHz
WDW                  EM
SSB                   0
LB                 0.30 Hz
GB                    0
PC                 2.00

1D NMR plot parameters
CX                22.80 cm
CY                15.00 cm
F1P               9.000 ppm
F1              3601.17 Hz
F2P              -0.500 ppm
F2              -200.06 Hz
PPMCM           0.41667 ppm/cm
HZCM          166.72086 Hz/cm
```

1H spectrum

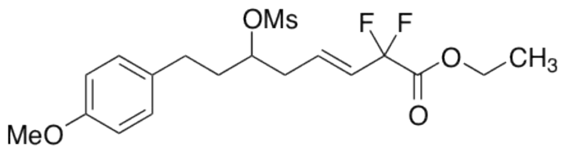

**SI-13**

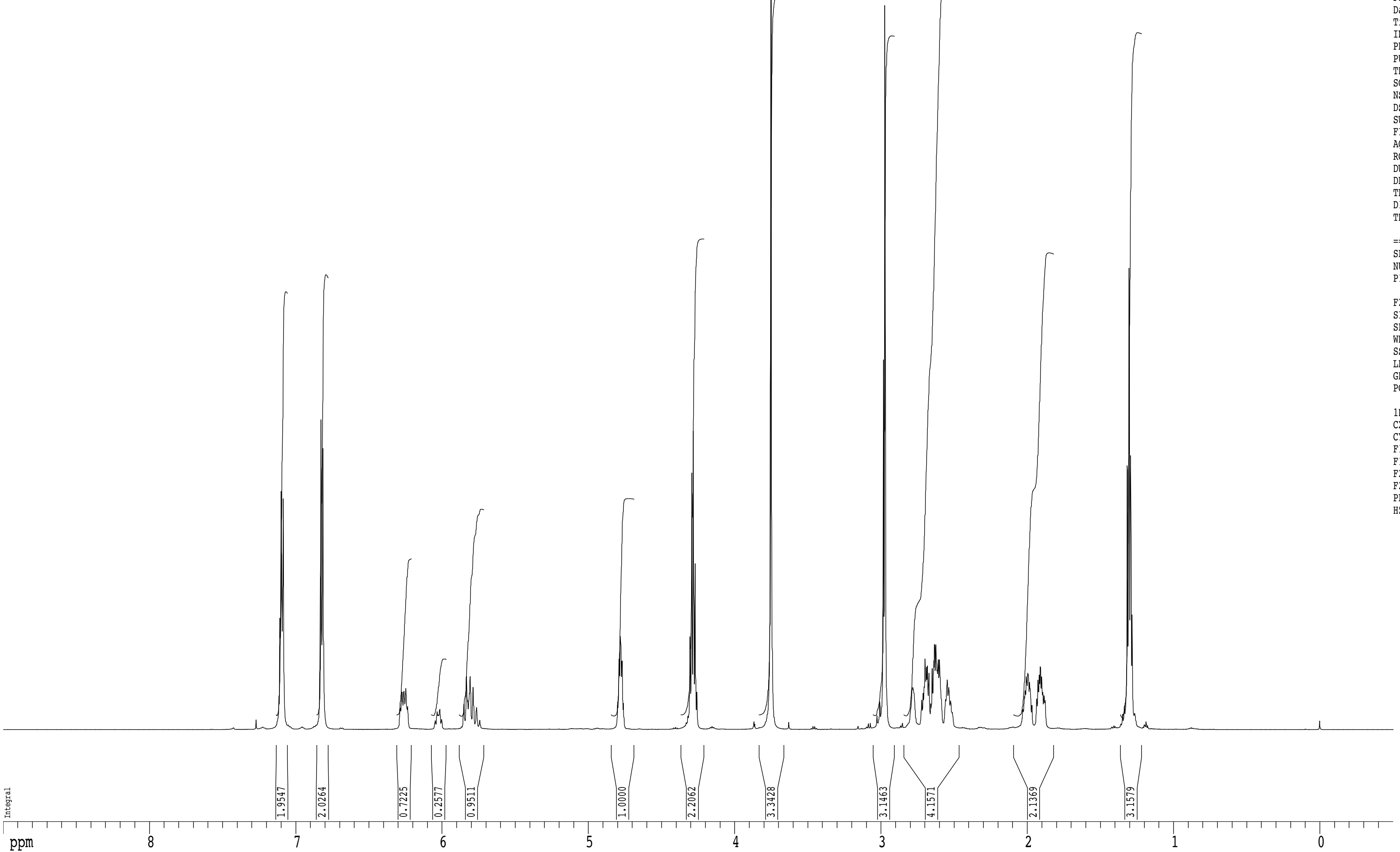

```
Current Data Parameters
USER            linpc2
NAME          pcl-2-132
EXPNO                3
PROCNO               1

F2 - Acquisition Parameters
Date_         20210816
Time              8.37
INSTRUM          av600
PROBHD   5 mm CPBBO BB-
PULPROG           zg30
TD               98074
SOLVENT          CDCl3T
NS                   8
DS                   2
SWH           9615.385 Hz
FIDRES        0.098042 Hz
AQ           5.0998979 sec
RG                7.12
DW              52.000 usec
DE               14.23 usec
TE               298.0 K
D1          0.10000000 sec
TD0                  1

======== CHANNEL f1 ========
SFO1       600.1342009 MHz
NUC1                1H
P1                9.50 usec

F2 - Processing parameters
SI               65536
SF         600.1300276 MHz
WDW                 no
SSB                  0
LB                0.00 Hz
GB                   0
PC                1.00

1D NMR plot parameters
CX               22.80 cm
CY               15.00 cm
F1P              9.000 ppm
F1             5401.17 Hz
F2P             -0.500 ppm
F2             -300.06 Hz
PPMCM          0.41667 ppm/cm
HZCM          250.05418 Hz/cm
```

13C spectrum

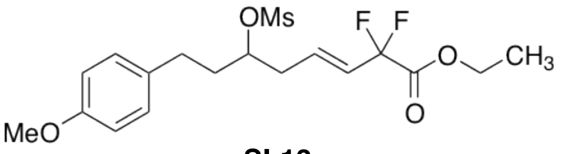


**SI-13**

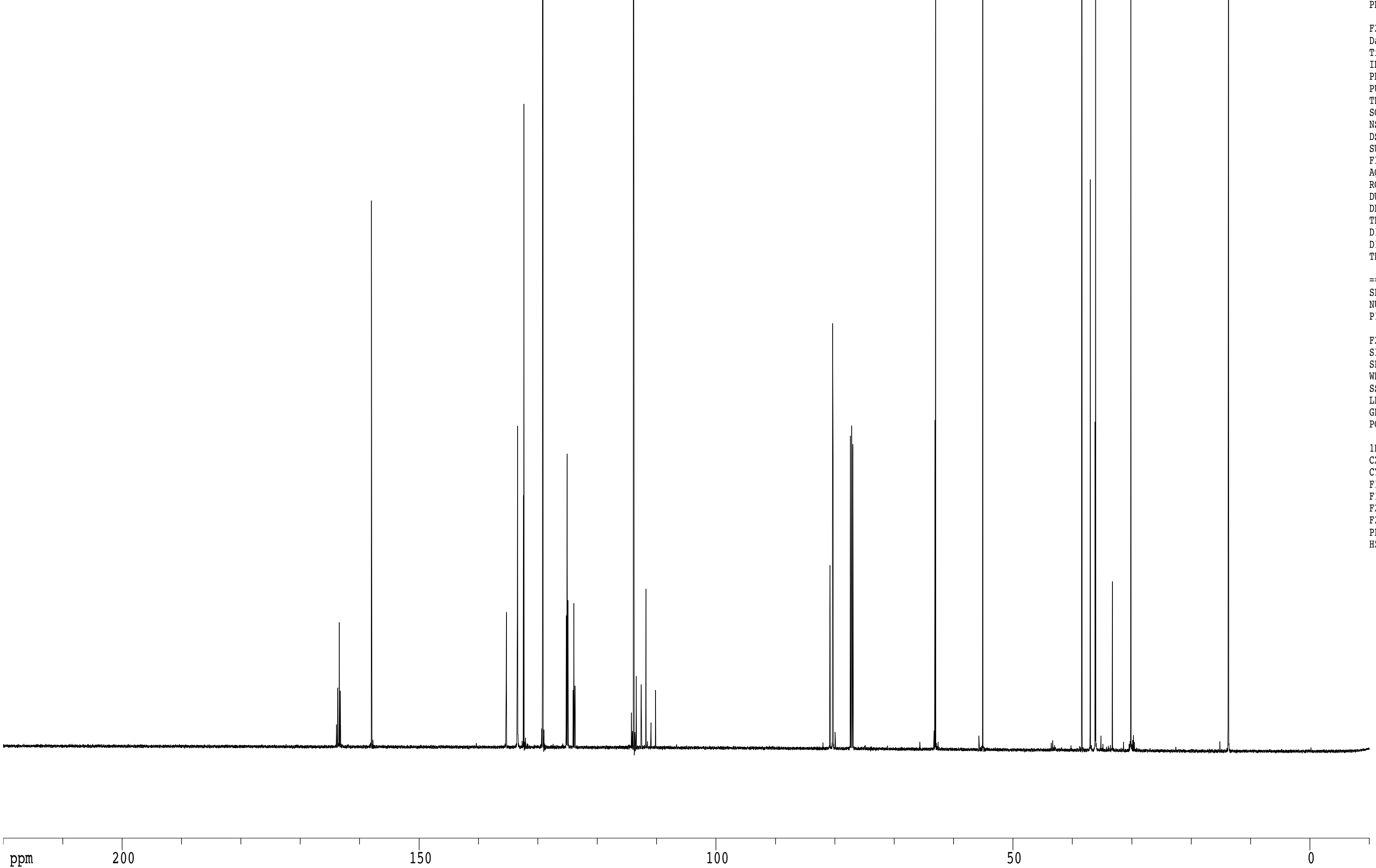


Current Data Parameters
USER linpc2
NAME pcl-2-132
EXPNO 4
PROCNO 1

F2 - Acquisition Parameters
Date_ 20210816
Time 8.41
INSTRUM av600
PROBHD 5 mm CPBBO BB-
PULPROG zgdc30
TD 65536
SOLVENT CDCl3T
NS 205
DS 4
SWH 36231.883 Hz
FIDRES 0.552855 Hz
AQ 0.9044468 sec
RG 2050
DW 13.800 usec
DE 19.63 usec
TE 298.0 K
D1 0.40000001 sec
D11 0.03000000 sec
TD0 1

======== CHANNEL f1 ========
SFO1 150.9194080 MHz
NUC1 13C
P1 10.10 usec

F2 - Processing parameters
SI 65536
SF 150.9028312 MHz
WDW no
SSB 0
LB 0.00 Hz
GB 0
PC 1.00

1D NMR plot parameters
CX 22.80 cm
CY 45.00 cm
F1P 220.000 ppm
F1 33198.62 Hz
F2P -10.000 ppm
F2 -1509.03 Hz
PPMCM 10.08772 ppm/cm
HZCM 1522.26550 Hz/cm

19F spectrum

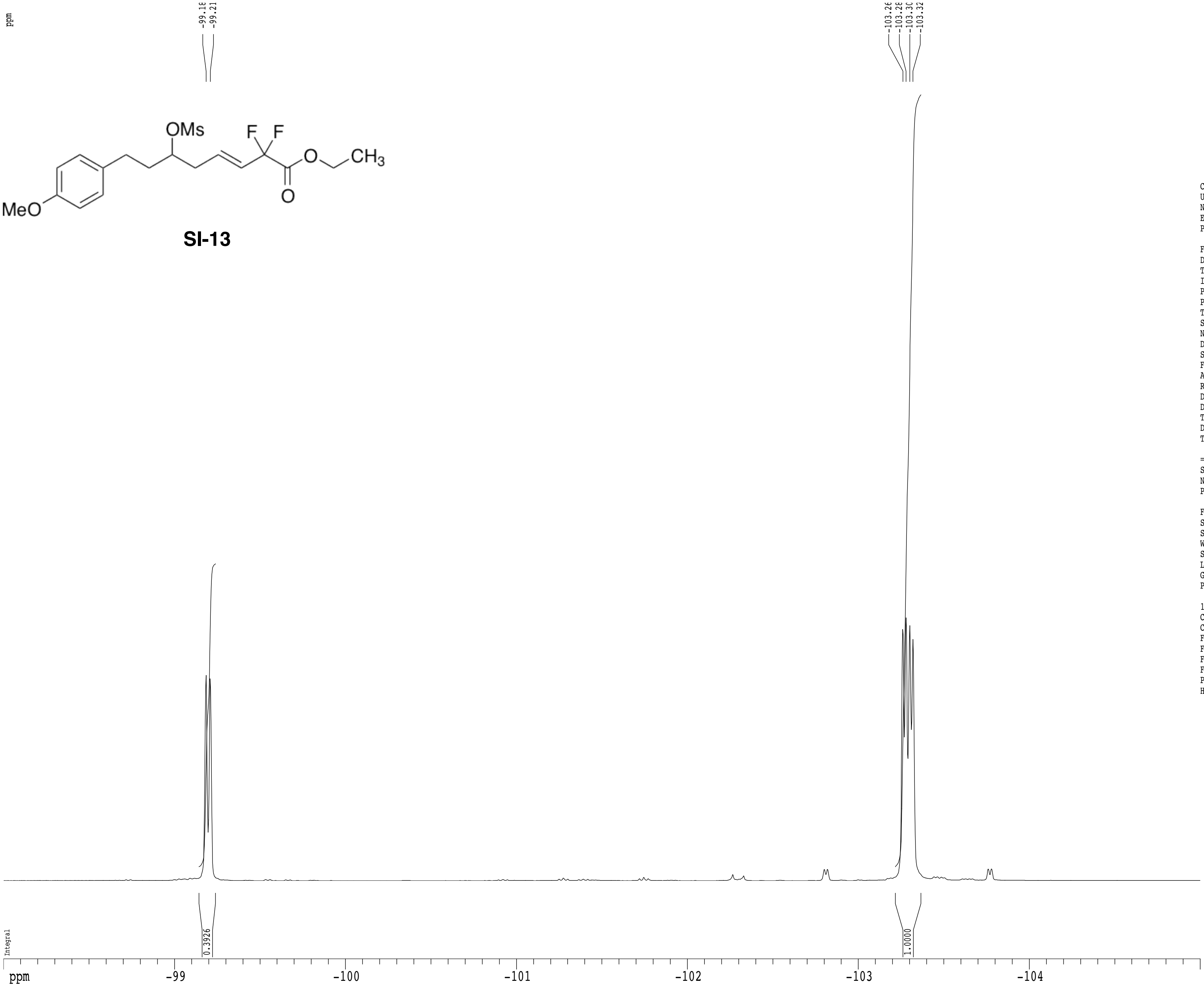


Current Data Parameters
USER linpc2
NAME pcl-2-132
EXPNO 5
PROCNO 1

F2 - Acquisition Parameters
Date_ 20210816
Time 8.49
INSTRUM av600
PROBHD 5 mm CPBBO BB-
PULPROG zgflqn30
TD 131072
SOLVENT CDCl3T
NS 16
DS 2
SWH 178571.422 Hz
FIDRES 1.362392 Hz
AQ 0.3670516 sec
RG 228
DW 2.800 usec
DE 18.00 usec
TE 298.0 K
D1 3.00000000 sec
TD0 1

======== CHANNEL f1 ========
SFO1 564.6299196 MHz
NUC1 19F
P1 18.25 usec

F2 - Processing parameters
SI 131072
SF 564.6864142 MHz
WDW no
SSB 0
LB 0.00 Hz
GB 0
PC 1.00

1D NMR plot parameters
CX 22.80 cm
CY 5.00 cm
F1P -98.000 ppm
F1 -55339.27 Hz
F2P -105.000 ppm
F2 -59292.07 Hz
PPMCM 0.30702 ppm/cm
HZCM 173.36864 Hz/cm

1H spectrum

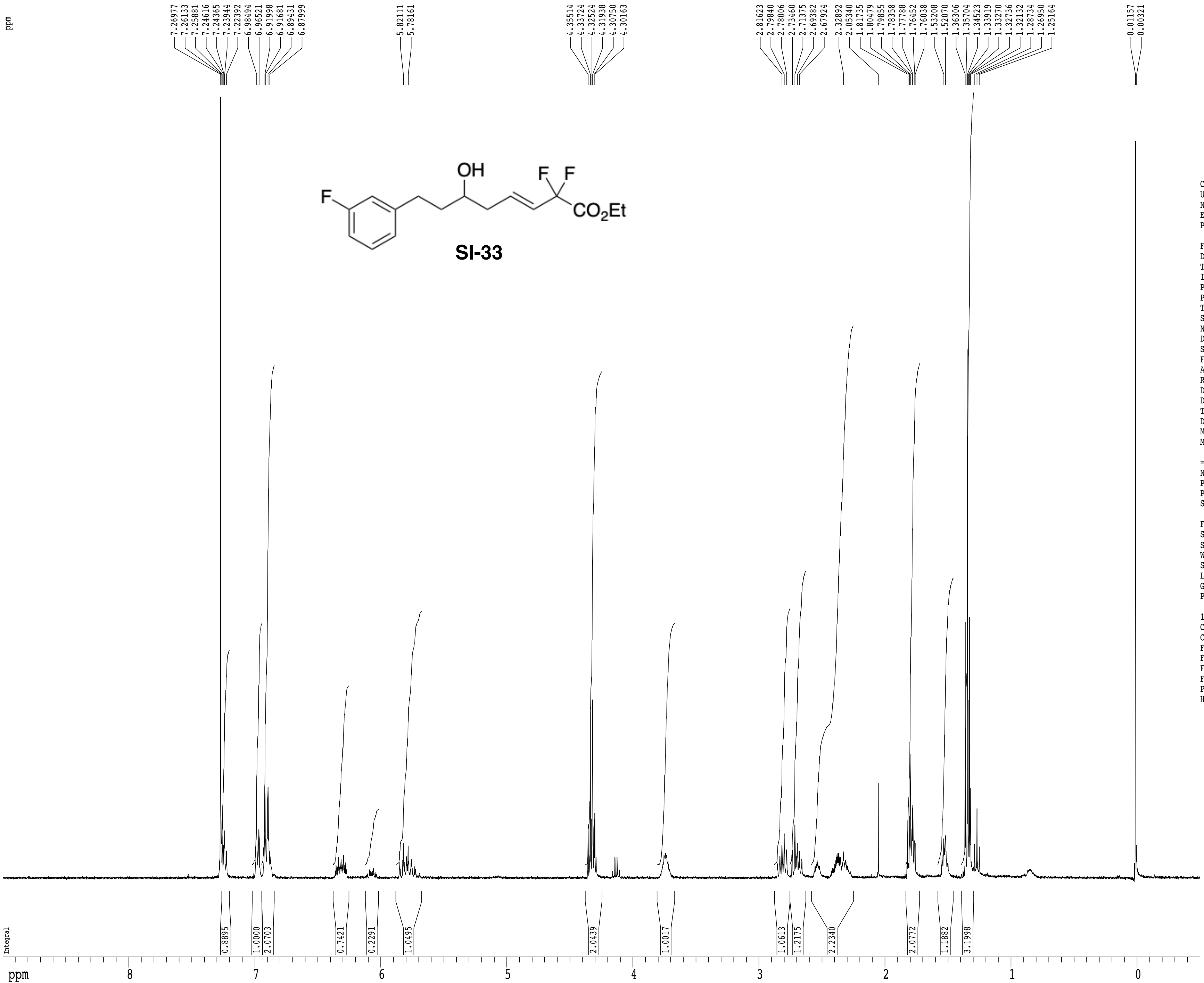


Current Data Parameters
USER sanforda
NAME ABS-4-075-pure
EXPNO 1
PROCNO 1

F2 - Acquisition Parameters
Date_ 20210302
Time 10.24
INSTRUM drx400
PROBHD 5 mm QNP H/F/P
PULPROG zg30
TD 38460
SOLVENT CDCl3
NS 8
DS 2
SWH 6410.256 Hz
FIDRES 0.166673 Hz
AQ 2.9999299 sec
RG 724.1
DW 78.000 usec
DE 4.50 usec
TE 298.0 K
D1 0.10000000 sec
MCREST 0.00000000 sec
MCWRK 0.01500000 sec

======== CHANNEL f1 ========
NUC1 1H
P1 12.00 usec
PL1 -1.60 dB
SFO1 400.1328009 MHz

F2 - Processing parameters
SI 65536
SF 400.1300175 MHz
WDW no
SSB 0
LB 0.00 Hz
GB 0
PC 2.00

1D NMR plot parameters
CX 22.80 cm
CY 15.00 cm
F1P 9.000 ppm
F1 3601.17 Hz
F2P -0.500 ppm
F2 -200.06 Hz
PPMCM 0.41667 ppm/cm
HZCM 166.72084 Hz/cm

1H spectrum

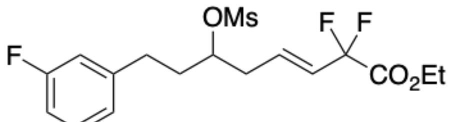


**SI-14**

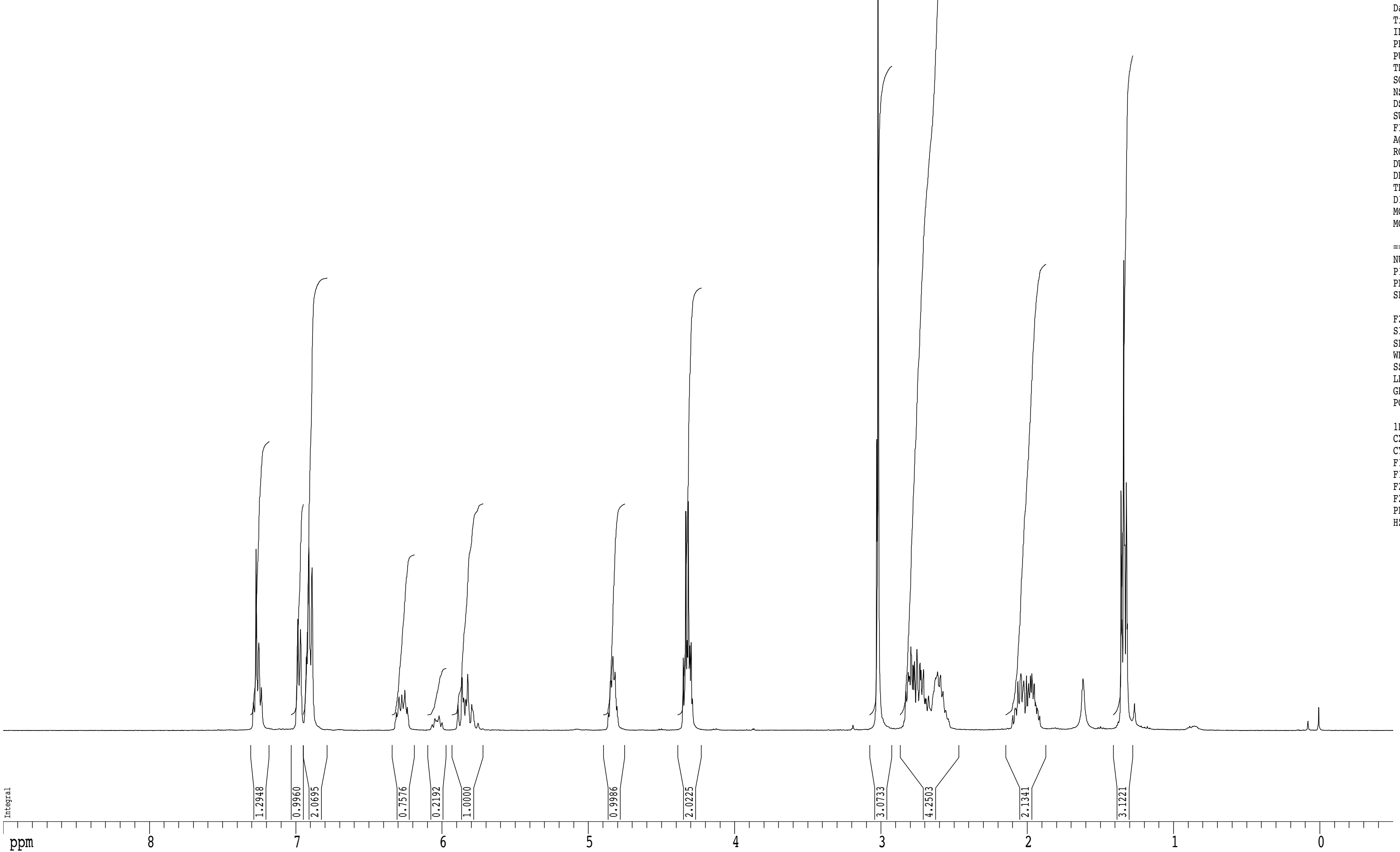


Current Data Parameters
USER mcginnit
NAME tmm-3-amboFmes
EXPNO 10
PROCNO 1

F2 - Acquisition Parameters
Date_ 20210809
Time 15.25
INSTRUM drx400
PROBHD 5 mm QNP H/F/P
PULPROG zg30
TD 65536
SOLVENT CDCl3
NS 8
DS 2
SWH 6410.256 Hz
FIDRES 0.097813 Hz
AQ 5.1118579 sec
RG 181
DW 78.000 usec
DE 4.50 usec
TE 298.0 K
D1 0.10000000 sec
MCREST 0.00000000 sec
MCWRK 0.01500000 sec

======== CHANNEL f1 ========
NUC1 1H
P1 12.00 usec
PL1 -1.60 dB
SFO1 400.1328009 MHz

F2 - Processing parameters
SI 65536
SF 400.1300175 MHz
WDW EM
SSB 0
LB 0.30 Hz
GB 0
PC 2.00

1D NMR plot parameters
CX 22.80 cm
CY 15.00 cm
F1P 9.000 ppm
F1 3601.17 Hz
F2P -0.500 ppm
F2 -200.06 Hz
PPMCM 0.41667 ppm/cm
HZCM 166.72084 Hz/cm

Z-restored spin-echo 13C spectrum with 1H decoupling

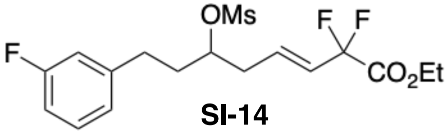


**SI-14**

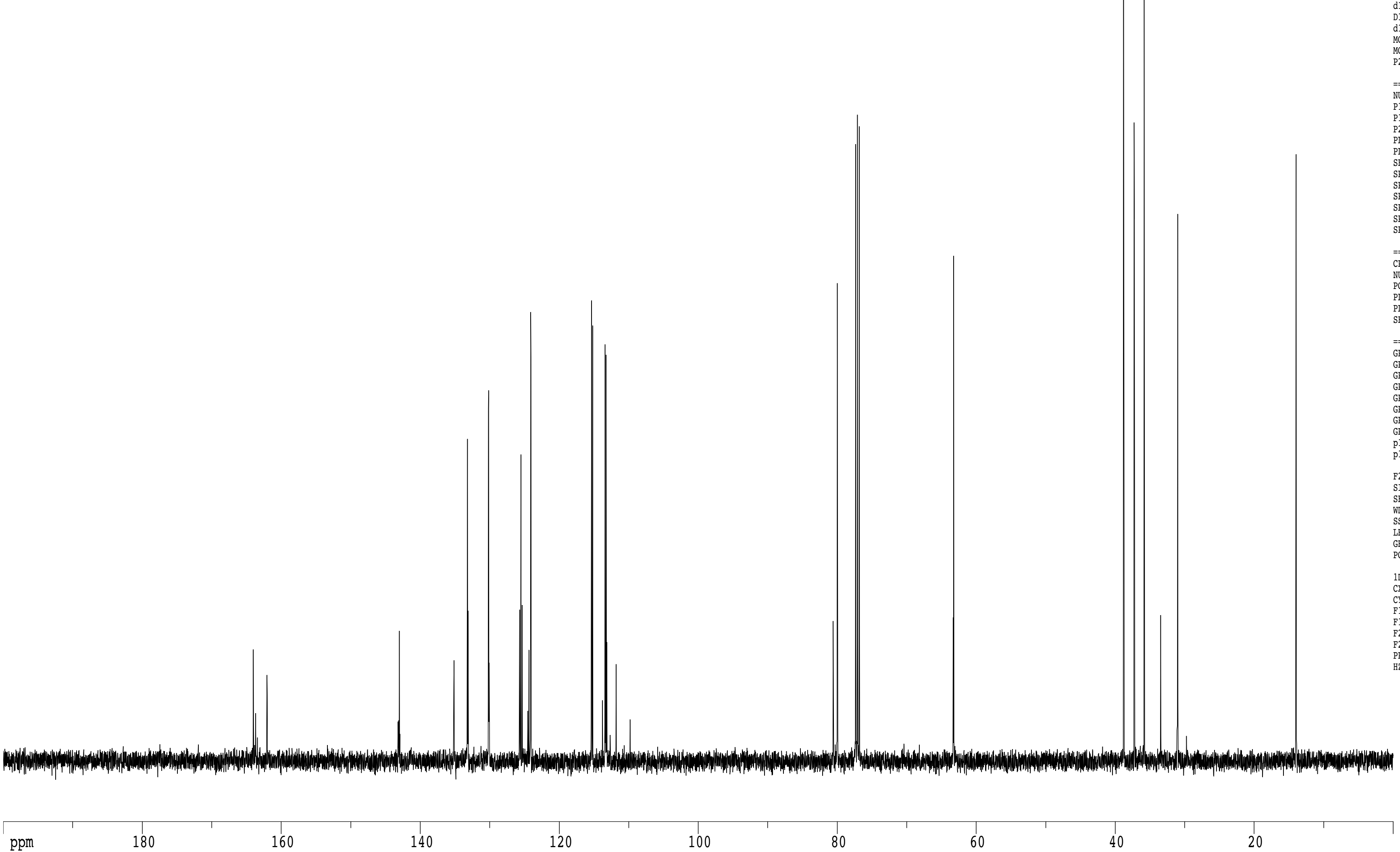


Current Data Parameters
USER mcginnit
NAME tmm-3-amboFmes
EXPNO 12
PROCNO 1

F2 - Acquisition Parameters
Date_ 20210809
Time 16.05
INSTRUM cryo500
PROBHD 5 mm CPTCI 1H-
PULPROG SpinEchopg30gp2.prd
TD 65536
SOLVENT CDCl3
NS 246
DS 16
SWH 30303.031 Hz
FIDRES 0.462388 Hz
AQ 1.0813940 sec
RG 7298.2
DW 16.500 usec
DE 6.00 usec
TE 298.0 K
D1 0.25000000 sec
d11 0.03000000 sec
D16 0.00020000 sec
d17 0.00019600 sec
MCREST 0.00000000 sec
MCWRK 0.01500000 sec
P2 37.70 usec

======== CHANNEL f1 ========
NUC1 13C
P1 18.85 usec
P12 2000.00 usec
P20 500.00 usec
PL0 120.00 dB
PL1 -1.00 dB
SFO1 125.7942548 MHz
SP2 1.55 dB
SP4 1.55 dB
SPNAM2 Crp60comp.4
SPNAM4 Crp60,0.5,20.1
SPOFF2 0.00 Hz
SPOFF4 0.00 Hz

======== CHANNEL f2 ========
CPDPRG2 waltz16
NUC2 1H
PCPD2 100.00 usec
PL2 1.60 dB
PL12 22.00 dB
SFO2 500.2225011 MHz

====== GRADIENT CHANNEL =====
GPNAM1 SINE.100
GPNAM2 SINE.100
GPX1 0.00 %
GPX2 0.00 %
GPY1 0.00 %
GPY2 0.00 %
GPZ1 30.00 %
GPZ2 50.00 %
p15 500.00 usec
p16 1000.00 usec

F2 - Processing parameters
SI 65536
SF 125.7804190 MHz
WDW EM
SSB 0
LB 1.00 Hz
GB 0
PC 2.00

1D NMR plot parameters
CX 22.80 cm
CY 15.65 cm
F1P 200.000 ppm
F1 25156.08 Hz
F2P 0.000 ppm
F2 0.00 Hz
PPMCM 8.77193 ppm/cm
HZCM 1103.33704 Hz/cm

19F spectrum

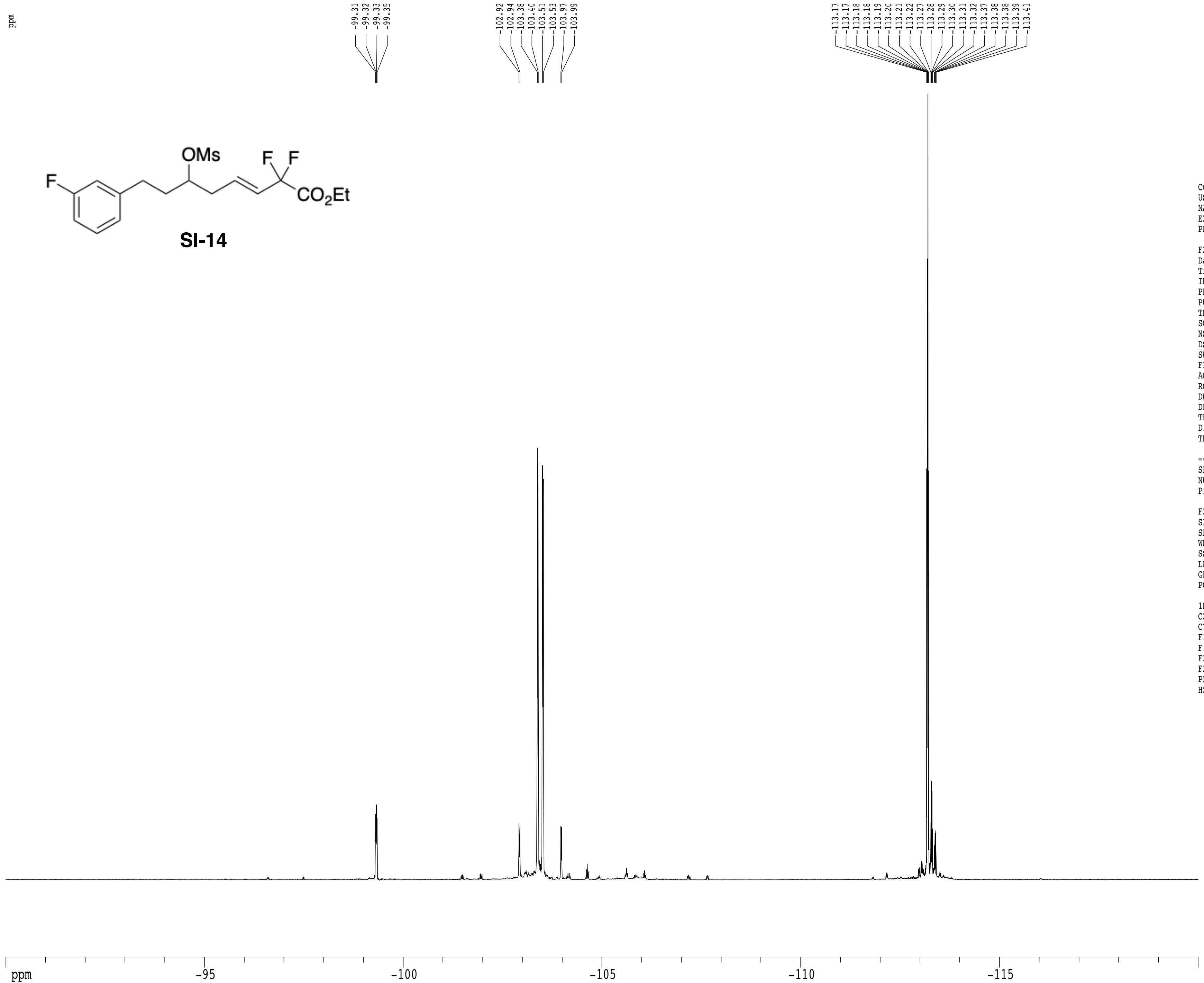


```
Current Data Parameters
USER          mcginnit
NAME          tmm-2-300
EXPNO                4
PROCNO               1

F2 - Acquisition Parameters
Date_         20210209
Time             15.01
INSTRUM          av600
PROBHD   5 mm CPBBO BB-
PULPROG       zgflqn30
TD              131072
SOLVENT          CDCl3
NS                  16
DS                   2
SWH         178571.422 Hz
FIDRES        1.362392 Hz
AQ           0.3670516 sec
RG                 456
DW               2.800 usec
DE               18.00 usec
TE               298.1 K
D1          3.00000000 sec
TD0                  1

======== CHANNEL f1 ========
SFO1       564.6299196 MHz
NUC1               19F
P1               18.25 usec

F2 - Processing parameters
SI              131072
SF         564.6863858 MHz
WDW                 no
SSB                  0
LB                0.00 Hz
GB                   0
PC                1.00

1D NMR plot parameters
CX               22.80 cm
CY               15.00 cm
F1P            -90.000 ppm
F1           -50821.78 Hz
F2P           -120.000 ppm
F2           -67762.37 Hz
PPMCM          1.31579 ppm/cm
HZCM         743.00842 Hz/cm
```

1H spectrum

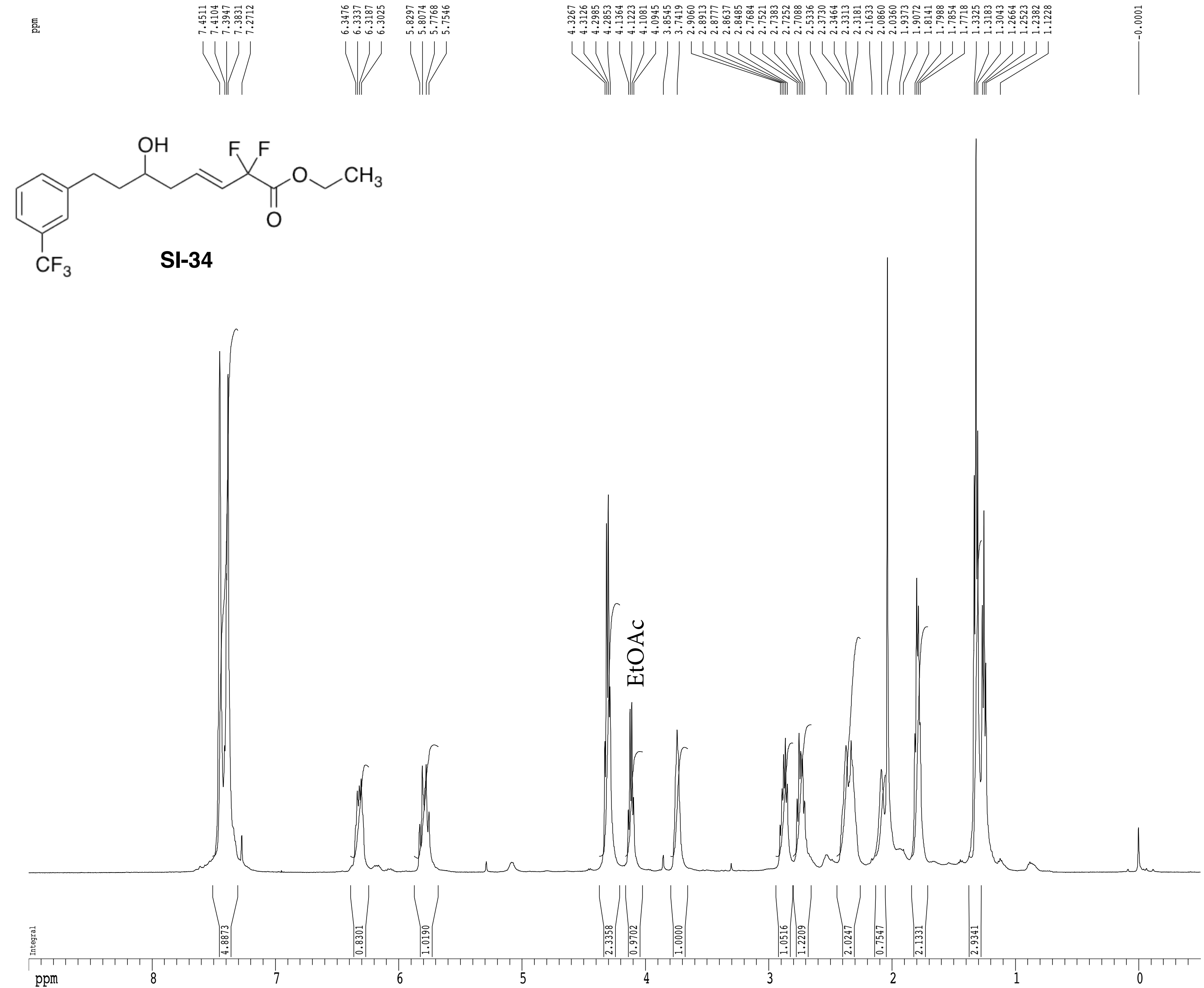


Current Data Parameters
USER linpc2
NAME pcl-1-215
EXPNO 4
PROCNO 1

F2 - Acquisition Parameters
Date_ 20210125
Time 9.50
INSTRUM gn500
PROBHD 5 mm broadband
PULPROG zg30
TD 81728
SOLVENT CDCl3
NS 8
DS 2
SWH 8012.820 Hz
FIDRES 0.098043 Hz
AQ 5.0998774 sec
RG 40.3
DW 62.400 usec
DE 6.00 usec
TE 298.0 K
D1 0.10000000 sec
MCREST 0.00000000 sec
MCWRK 0.01500000 sec

======== CHANNEL f1 ========
NUC1 1H
P1 12.00 usec
PL1 -6.00 dB
SFO1 498.7534913 MHz

F2 - Processing parameters
SI 65536
SF 498.7500251 MHz
WDW EM
SSB 0
LB 0.30 Hz
GB 0
PC 1.00

1D NMR plot parameters
CX 20.00 cm
CY 12.50 cm
F1P 9.000 ppm
F1 4488.75 Hz
F2P -0.500 ppm
F2 -249.38 Hz
PPMCM 0.47500 ppm/cm
HZCM 236.90627 Hz/cm SI-190

13C spectrum with 1H decoupling

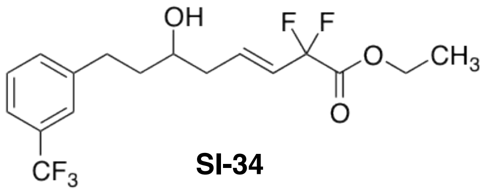

SI-34

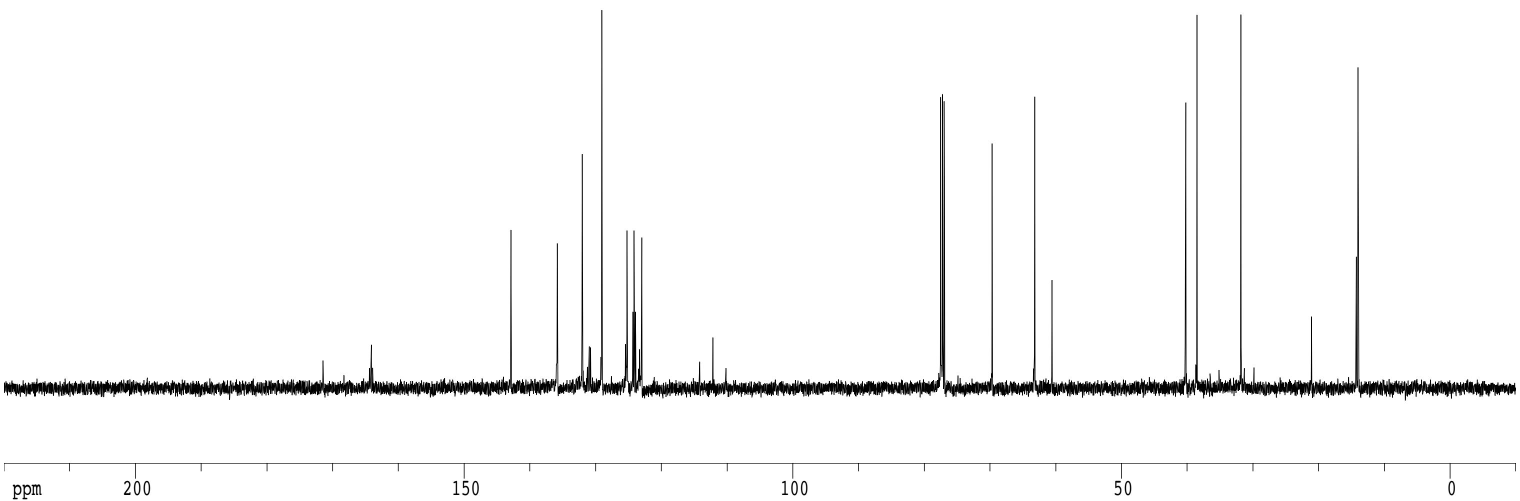


Current Data Parameters
USER linpc2
NAME pcl-1-215
EXPNO 5
PROCNO 1

F2 - Acquisition Parameters
Date_ 20210125
Time 9.52
INSTRUM gn500
PROBHD 5 mm broadband
PULPROG zgdc30
TD 65536
SOLVENT CDCl3
NS 136
DS 4
SWH 30303.031 Hz
FIDRES 0.462388 Hz
AQ 1.0813940 sec
RG 5792.6
DW 16.500 usec
DE 6.00 usec
TE 298.0 K
D1 0.25000000 sec
d11 0.03000000 sec
MCREST 0.00000000 sec
MCWRK 0.01500000 sec

======== CHANNEL f1 ========
NUC1 13C
P1 14.20 usec
PL1 -6.00 dB
SFO1 125.4245824 MHz

======== CHANNEL f2 ========
CPDPRG2 waltz16
NUC2 1H
PCPD2 80.00 usec
PL2 -6.00 dB
PL12 12.30 dB
SFO2 498.7524937 MHz

F2 - Processing parameters
SI 65536
SF 125.4107789 MHz
WDW EM
SSB 0
LB 1.00 Hz
GB 0
PC 2.00

1D NMR plot parameters
CX 20.00 cm
CY 5.00 cm
F1P 220.000 ppm
F1 27590.37 Hz
F2P -10.000 ppm
F2 -1254.11 Hz
PPMCM 11.50000 ppm/cm
HZCM 1442.22400 Hz/cm

1H spectrum

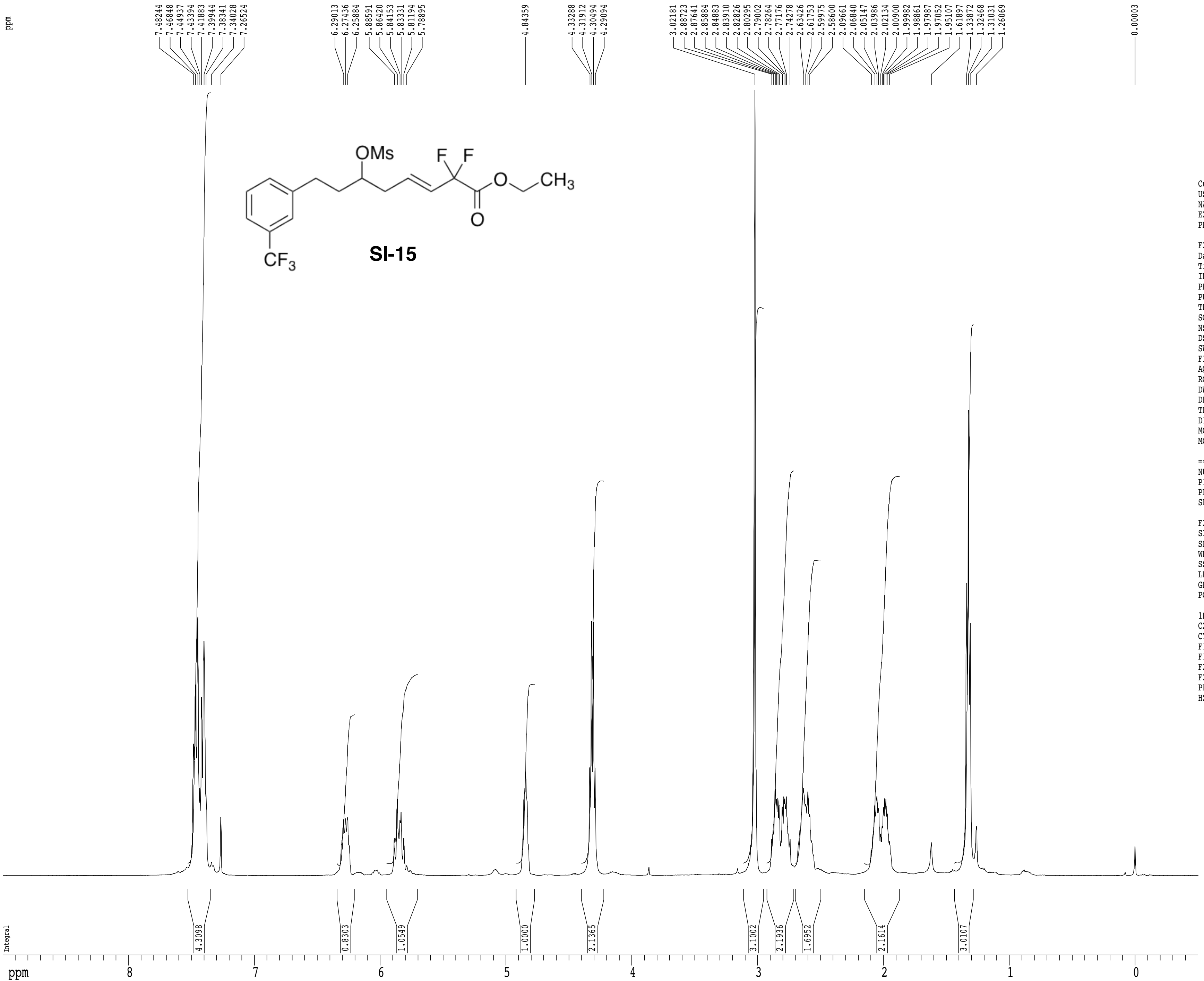


Current Data Parameters
USER linpc2
NAME pcl-1-218
EXPNO 2
PROCNO 1

F2 - Acquisition Parameters
Date_ 20210422
Time 13.37
INSTRUM cryo500
PROBHD 5 mm CPTCI 1H-
PULPROG zg30
TD 81728
SOLVENT CDCl3
NS 8
DS 2
SWH 8012.820 Hz
FIDRES 0.098043 Hz
AQ 5.0998774 sec
RG 4
DW 62.400 usec
DE 6.00 usec
TE 298.0 K
D1 0.10000000 sec
MCREST 0.00000000 sec
MCWRK 0.01500000 sec

======== CHANNEL f1 ========
NUC1 1H
P1 9.75 usec
PL1 1.60 dB
SFO1 500.2235015 MHz

F2 - Processing parameters
SI 65536
SF 500.2200282 MHz
WDW EM
SSB 0
LB 0.30 Hz
GB 0
PC 1.00

1D NMR plot parameters
CX 22.80 cm
CY 15.00 cm
F1P 9.000 ppm
F1 4501.98 Hz
F2P -0.500 ppm
F2 -250.11 Hz
PPMCM 0.41667 ppm/cm
HZCM 208.42502 Hz/cm

Z-restored spin-echo 13C spectrum with 1H decoupling

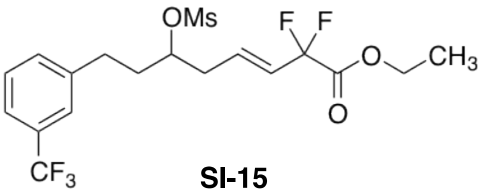


**SI-15**

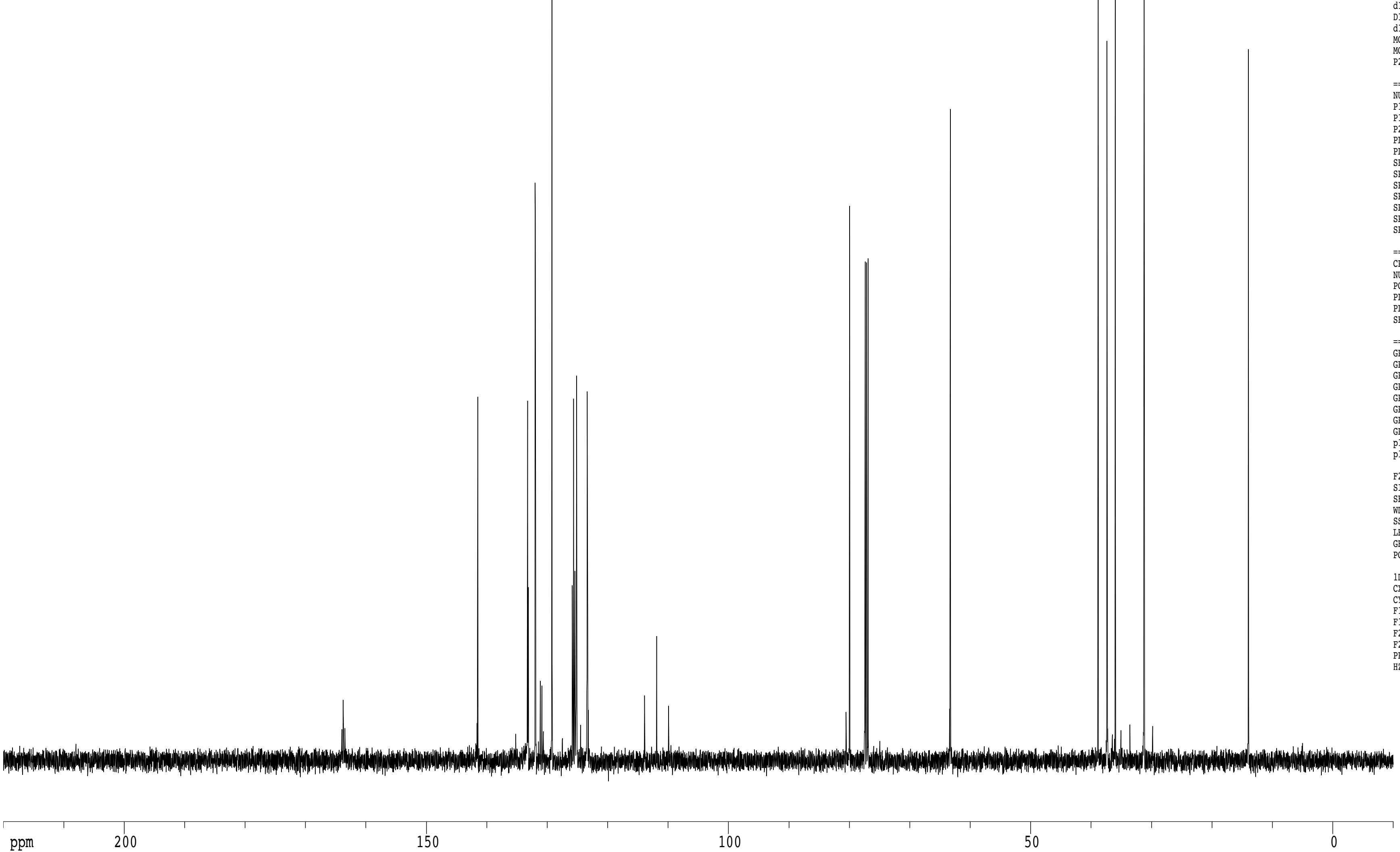


Current Data Parameters
USER linpc2
NAME pcl-1-218
EXPNO 3
PROCNO 1

F2 - Acquisition Parameters
Date_ 20210422
Time 13.40
INSTRUM cryo500
PROBHD 5 mm CPTCI 1H-
PULPROG SpinEchopg30gp2.prd
TD 65536
SOLVENT CDCl3
NS 96
DS 16
SWH 30303.031 Hz
FIDRES 0.462388 Hz
AQ 1.0813940 sec
RG 7298.2
DW 16.500 usec
DE 6.00 usec
TE 298.0 K
D1 0.25000000 sec
d11 0.03000000 sec
D16 0.00020000 sec
d17 0.00019600 sec
MCREST 0.00000000 sec
MCWRK 0.01500000 sec
P2 37.70 usec

======== CHANNEL f1 ========
NUC1 13C
P1 18.85 usec
P12 2000.00 usec
P20 500.00 usec
PL0 120.00 dB
PL1 -1.00 dB
SFO1 125.7942548 MHz
SP2 1.55 dB
SP4 1.55 dB
SPNAM2 Crp60comp.4
SPNAM4 Crp60,0.5,20.1
SPOFF2 0.00 Hz
SPOFF4 0.00 Hz

======== CHANNEL f2 ========
CPDPRG2 waltz16
NUC2 1H
PCPD2 100.00 usec
PL2 1.60 dB
PL12 22.00 dB
SFO2 500.2225011 MHz

====== GRADIENT CHANNEL =====
GPNAM1 SINE.100
GPNAM2 SINE.100
GPX1 0.00 %
GPX2 0.00 %
GPY1 0.00 %
GPY2 0.00 %
GPZ1 30.00 %
GPZ2 50.00 %
p15 500.00 usec
p16 1000.00 usec

F2 - Processing parameters
SI 65536
SF 125.7804099 MHz
WDW EM
SSB 0
LB 1.00 Hz
GB 0
PC 2.00

1D NMR plot parameters
CX 22.80 cm
CY 15.65 cm
F1P 220.000 ppm
F1 27671.69 Hz
F2P -10.000 ppm
F2 -1257.80 Hz
PPMCM 10.08772 ppm/cm
HZCM 1268.83752 Hz/cm

19F spectrum

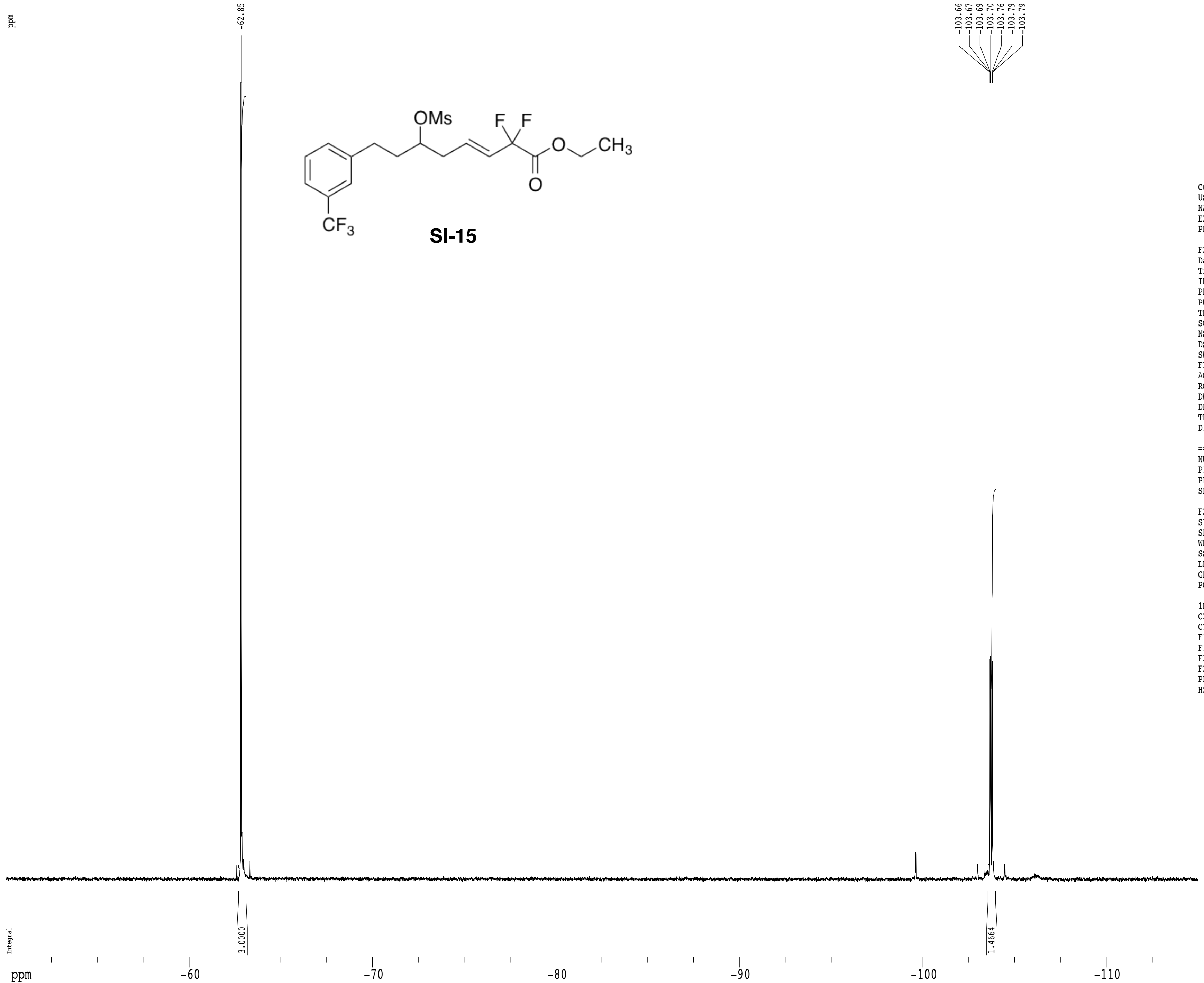


Current Data Parameters
USER linpc2
NAME pcl-1-218
EXPNO 5
PROCNO 1

F2 - Acquisition Parameters
Date_ 20210426
Time 8.03
INSTRUM drx400
PROBHD 5 mm QNP H/F/P
PULPROG zgflqn30
TD 65536
SOLVENT CDCl3
NS 24
DS 2
SWH 75187.969 Hz
FIDRES 1.147277 Hz
AQ 0.4358644 sec
RG 6502
DW 6.650 usec
DE 9.46 usec
TE 297.9 K
D1 2.00000000 sec

======== CHANNEL f1 ========
NUC1 19F
P1 11.75 usec
PL1 -6.00 dB
SFO1 376.4646491 MHz

F2 - Processing parameters
SI 65536
SF 376.4984640 MHz
WDW EM
SSB 0
LB 1.00 Hz
GB 0
PC 1.00

1D NMR plot parameters
CX 22.80 cm
CY 60.00 cm
F1P -50.000 ppm
F1 -18824.92 Hz
F2P -115.000 ppm
F2 -43297.32 Hz
PPMCM 2.85088 ppm/cm
HZCM 1073.35095 Hz/cm

1H spectrum

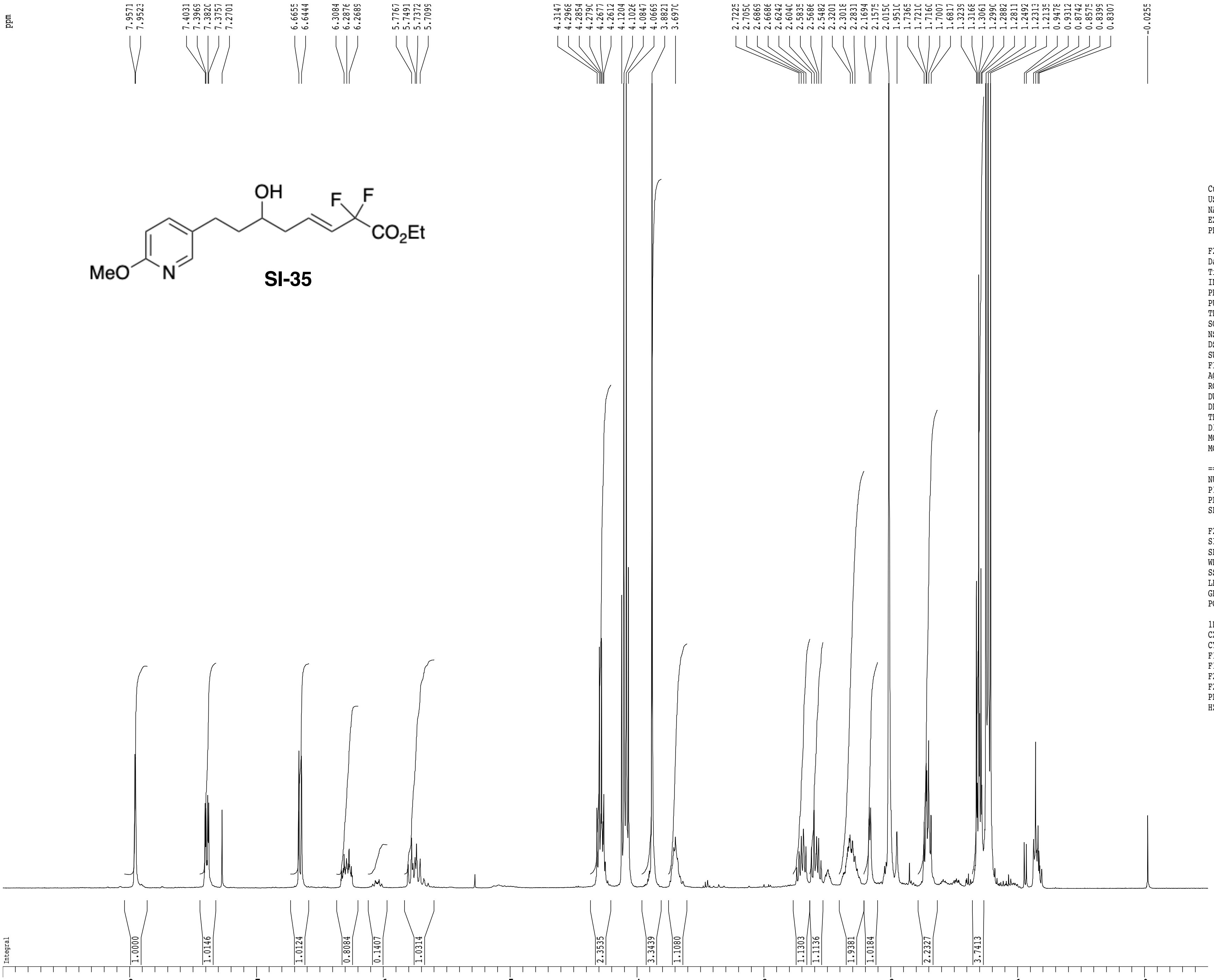


Current Data Parameters
USER mcginnit
NAME tmm-3-220
EXPNO 3
PROCNO 1

F2 - Acquisition Parameters
Date_ 20210901
Time 15.10
INSTRUM drx400
PROBHD 5 mm Multinucl
PULPROG zg30
TD 65536
SOLVENT CDCl3
NS 8
DS 2
SWH 6410.256 Hz
FIDRES 0.097813 Hz
AQ 5.1118579 sec
RG 90.5
DW 78.000 usec
DE 4.50 usec
TE 298.0 K
D1 0.10000000 sec
MCREST 0.00000000 sec
MCWRK 0.01500000 sec

======== CHANNEL f1 ========
NUC1 1H
P1 12.00 usec
PL1 -1.10 dB
SFO1 400.1328009 MHz

F2 - Processing parameters
SI 65536
SF 400.1300175 MHz
WDW EM
SSB 0
LB 0.30 Hz
GB 0
PC 2.00

1D NMR plot parameters
CX 22.80 cm
CY 75.00 cm
F1P 9.000 ppm
F1 3601.17 Hz
F2P -0.500 ppm
F2 -200.06 Hz
PPMCM 0.41667 ppm/cm
HZCM 166.72084 Hz/cm

1H spectrum

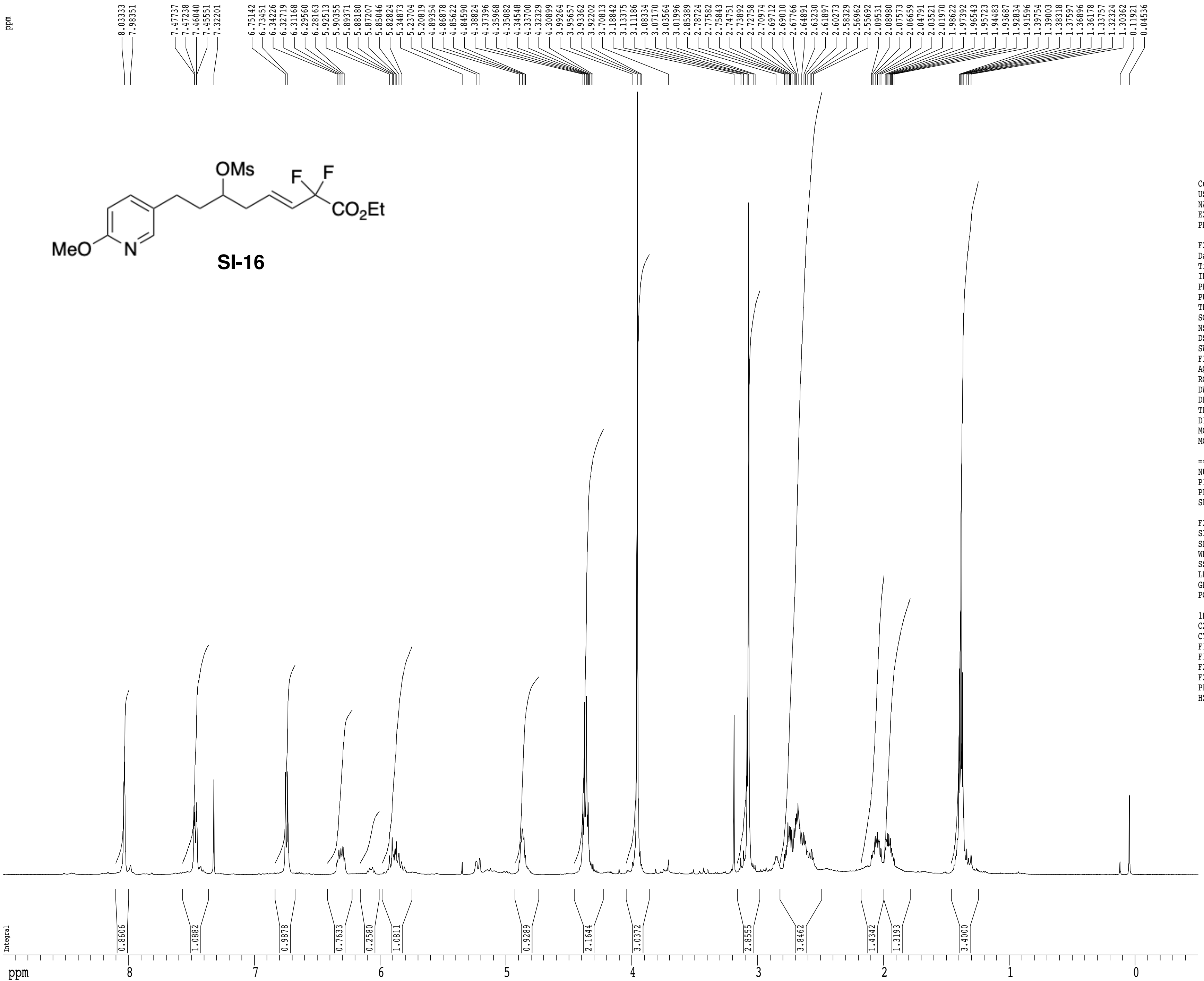


Current Data Parameters
USER mcginnit
NAME tmm-3-224-char
EXPNO 1
PROCNO 1

F2 - Acquisition Parameters
Date_ 20211223
Time 11.30
INSTRUM cryo500
PROBHD 5 mm CPTCI 1H-
PULPROG zg30
TD 81728
SOLVENT CDCl3
NS 8
DS 2
SWH 8012.820 Hz
FIDRES 0.098043 Hz
AQ 5.0998774 sec
RG 5
DW 62.400 usec
DE 6.00 usec
TE 298.0 K
D1 0.10000000 sec
MCREST 0.00000000 sec
MCWRK 0.01500000 sec

======== CHANNEL f1 ========
NUC1 1H
P1 9.75 usec
PL1 1.60 dB
SFO1 500.2235015 MHz

F2 - Processing parameters
SI 65536
SF 500.2200000 MHz
WDW EM
SSB 0
LB 0.30 Hz
GB 0
PC 1.00

1D NMR plot parameters
CX 22.80 cm
CY 15.00 cm
F1P 9.000 ppm
F1 4501.98 Hz
F2P -0.500 ppm
F2 -250.11 Hz
PPMCM 0.41667 ppm/cm
HZCM 208.42500 Hz/cm

Z-restored spin-echo 13C spectrum with 1H decoupling

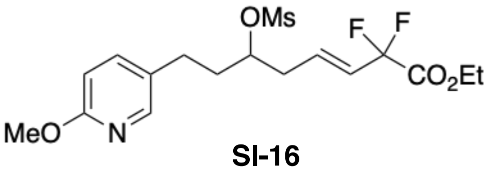


**SI-16**

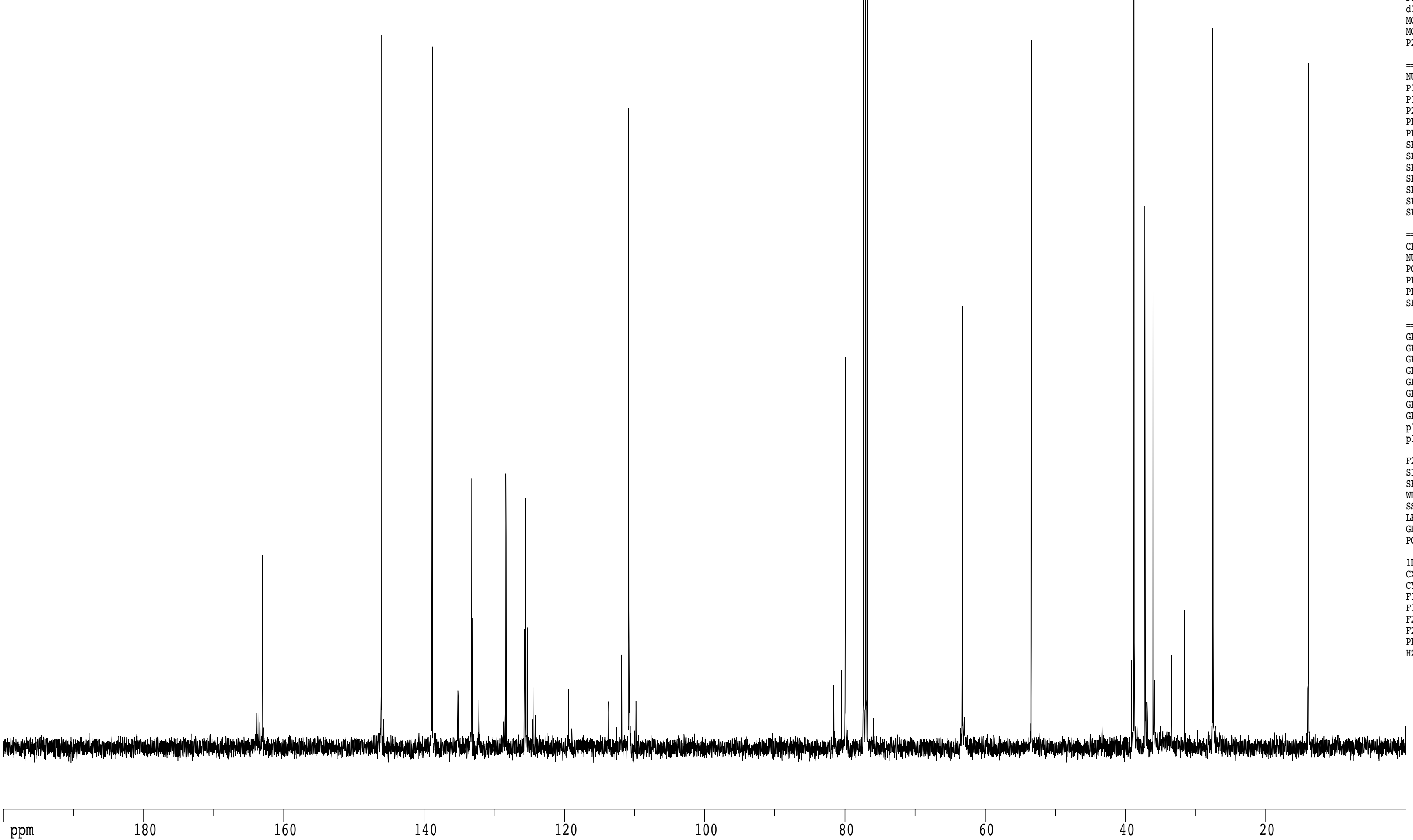


Current Data Parameters
USER mcginnit
NAME tmm-3-224-char
EXPNO 2
PROCNO 1

F2 - Acquisition Parameters
Date_ 20211223
Time 11.33
INSTRUM cryo500
PROBHD 5 mm CPTCI 1H-
PULPROG SpinEchopg30gp2.prd
TD 65536
SOLVENT CDCl3
NS 313
DS 16
SWH 30303.031 Hz
FIDRES 0.462388 Hz
AQ 1.0813940 sec
RG 6502
DW 16.500 usec
DE 6.00 usec
TE 298.0 K
D1 0.25000000 sec
d11 0.03000000 sec
D16 0.00020000 sec
d17 0.00019600 sec
MCREST 0.00000000 sec
MCWRK 0.01500000 sec
P2 37.70 usec

======== CHANNEL f1 ========
NUC1 13C
P1 18.85 usec
P12 2000.00 usec
P20 500.00 usec
PL0 120.00 dB
PL1 -1.00 dB
SFO1 125.7942548 MHz
SP2 1.55 dB
SP4 1.55 dB
SPNAM2 Crp60comp.4
SPNAM4 Crp60,0.5,20.1
SPOFF2 0.00 Hz
SPOFF4 0.00 Hz

======== CHANNEL f2 ========
CPDPRG2 waltz16
NUC2 1H
PCPD2 100.00 usec
PL2 1.60 dB
PL12 22.00 dB
SFO2 500.2225011 MHz

====== GRADIENT CHANNEL =====
GPNAM1 SINE.100
GPNAM2 SINE.100
GPX1 0.00 %
GPX2 0.00 %
GPY1 0.00 %
GPY2 0.00 %
GPZ1 30.00 %
GPZ2 50.00 %
p15 500.00 usec
p16 1000.00 usec

F2 - Processing parameters
SI 65536
SF 125.7804190 MHz
WDW EM
SSB 0
LB 1.00 Hz
GB 0
PC 2.00

1D NMR plot parameters
CX 22.80 cm
CY 15.65 cm
F1P 200.000 ppm
F1 25156.08 Hz
F2P 0.000 ppm
F2 0.00 Hz
PPMCM 8.77193 ppm/cm
HZCM 1103.33704 Hz/cm

19F spectrum

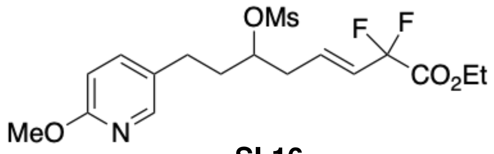

SI-16

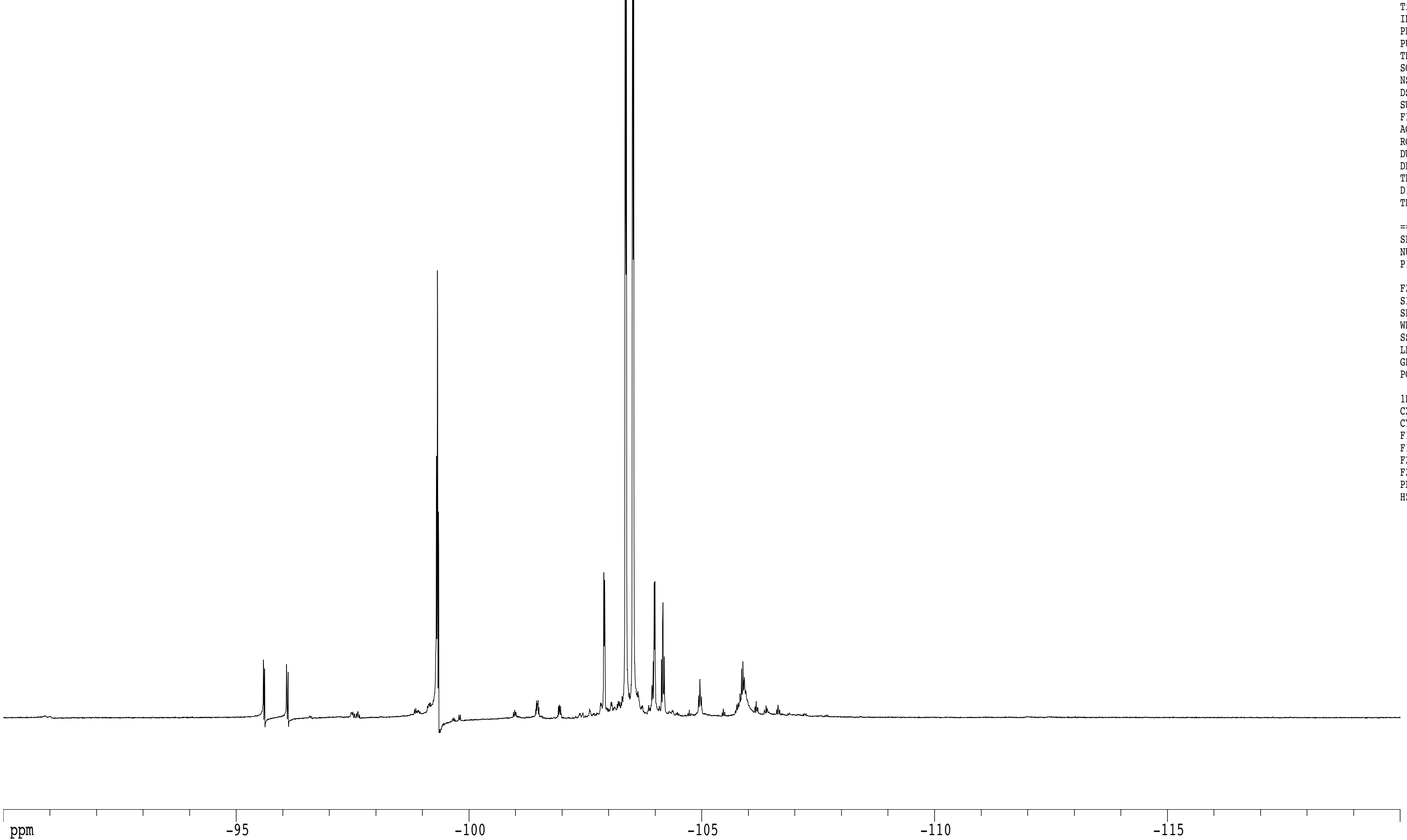


Current Data Parameters
USER mcginnit
NAME tmm-3-224-char
EXPNO 3
PROCNO 1

F2 - Acquisition Parameters
Date_ 20211223
Time 12.51
INSTRUM av600
PROBHD 5 mm CPBBO BB-
PULPROG zgflqn30
TD 131072
SOLVENT CDCl3
NS 16
DS 2
SWH 178571.422 Hz
FIDRES 1.362392 Hz
AQ 0.3670516 sec
RG 456
DW 2.800 usec
DE 18.00 usec
TE 298.0 K
D1 3.00000000 sec
TD0 1

======== CHANNEL f1 ========
SFO1 564.6299196 MHz
NUC1 19F
P1 18.25 usec

F2 - Processing parameters
SI 131072
SF 564.6863858 MHz
WDW no
SSB 0
LB 0.00 Hz
GB 0
PC 1.00

1D NMR plot parameters
CX 22.80 cm
CY 15.00 cm
F1P -90.000 ppm
F1 -50821.78 Hz
F2P -120.000 ppm
F2 -67762.37 Hz
PPMCM 1.31579 ppm/cm
HZCM 743.00842 Hz/cm

1H spectrum

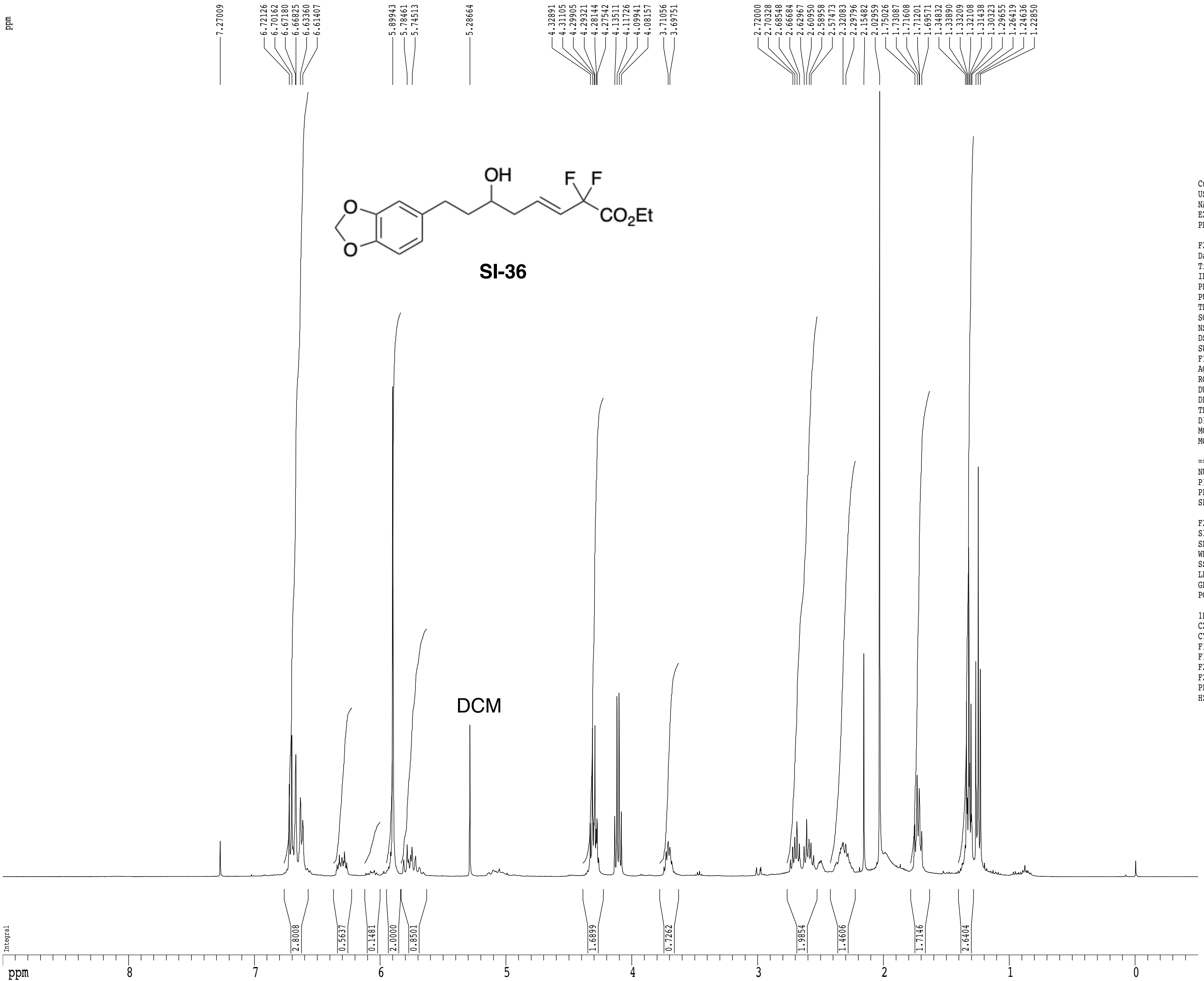


Current Data Parameters
USER mcginnit
NAME tmm-3-139
EXPNO 4
PROCNO 1

F2 - Acquisition Parameters
Date_ 20210521
Time 16.27
INSTRUM drx400
PROBHD 5 mm QNP H/F/P
PULPROG zg30
TD 65536
SOLVENT CDCl3
NS 8
DS 2
SWH 6410.256 Hz
FIDRES 0.097813 Hz
AQ 5.1118579 sec
RG 57
DW 78.000 usec
DE 4.50 usec
TE 298.0 K
D1 0.10000000 sec
MCREST 0.00000000 sec
MCWRK 0.01500000 sec

======== CHANNEL f1 ========
NUC1 1H
P1 12.00 usec
PL1 -1.60 dB
SFO1 400.1328009 MHz

F2 - Processing parameters
SI 65536
SF 400.1300175 MHz
WDW EM
SSB 0
LB 0.30 Hz
GB 0
PC 2.00

1D NMR plot parameters
CX 22.80 cm
CY 15.00 cm
F1P 9.000 ppm
F1 3601.17 Hz
F2P -0.500 ppm
F2 -200.06 Hz
PPMCM 0.41667 ppm/cm
HZCM 166.72084 Hz/cm

1H spectrum

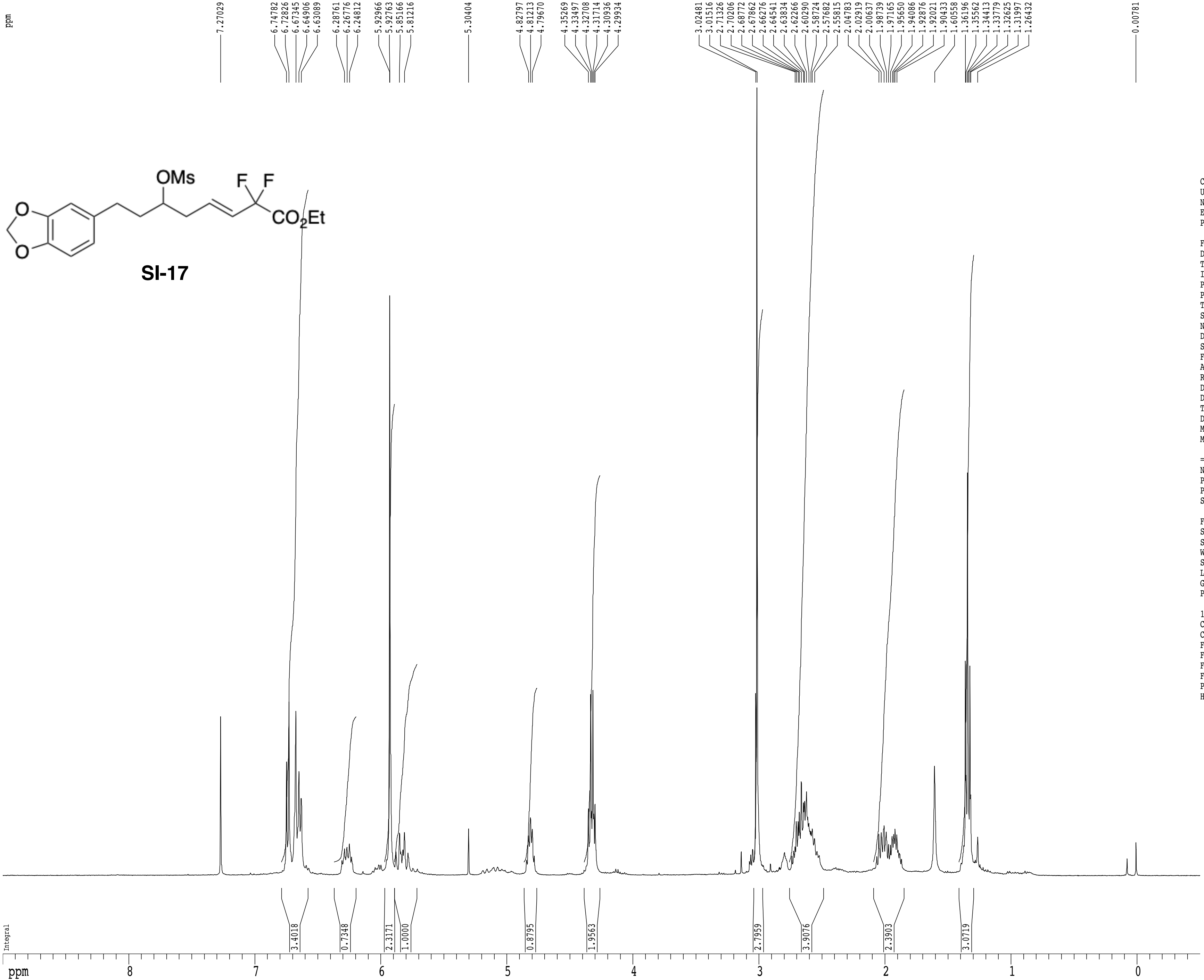


**SI-17**

```
Current Data Parameters
USER          mcginnit
NAME          tmm-3-141
EXPNO               10
PROCNO               1

F2 - Acquisition Parameters
Date_         20210809
Time             15.33
INSTRUM         drx400
PROBHD   5 mm QNP H/F/P
PULPROG           zg30
TD               65536
SOLVENT          CDCl3
NS                   8
DS                   2
SWH           6410.256 Hz
FIDRES        0.097813 Hz
AQ           5.1118579 sec
RG                 181
DW              78.000 usec
DE                4.50 usec
TE               298.0 K
D1          0.10000000 sec
MCREST      0.00000000 sec
MCWRK       0.01500000 sec

======== CHANNEL f1 ========
NUC1                1H
P1               12.00 usec
PL1              -1.60 dB
SFO1       400.1328009 MHz

F2 - Processing parameters
SI               65536
SF         400.1300175 MHz
WDW                 EM
SSB                  0
LB                0.30 Hz
GB                   0
PC                2.00

1D NMR plot parameters
CX               22.80 cm
CY               15.00 cm
F1P              9.000 ppm
F1             3601.17 Hz
F2P             -0.500 ppm
F2             -200.06 Hz
PPMCM          0.41667 ppm/cm
HZCM         166.72084 Hz/cm
```

Z-restored spin-echo 13C spectrum with 1H decoupling

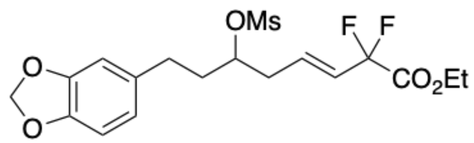

**SI-17**

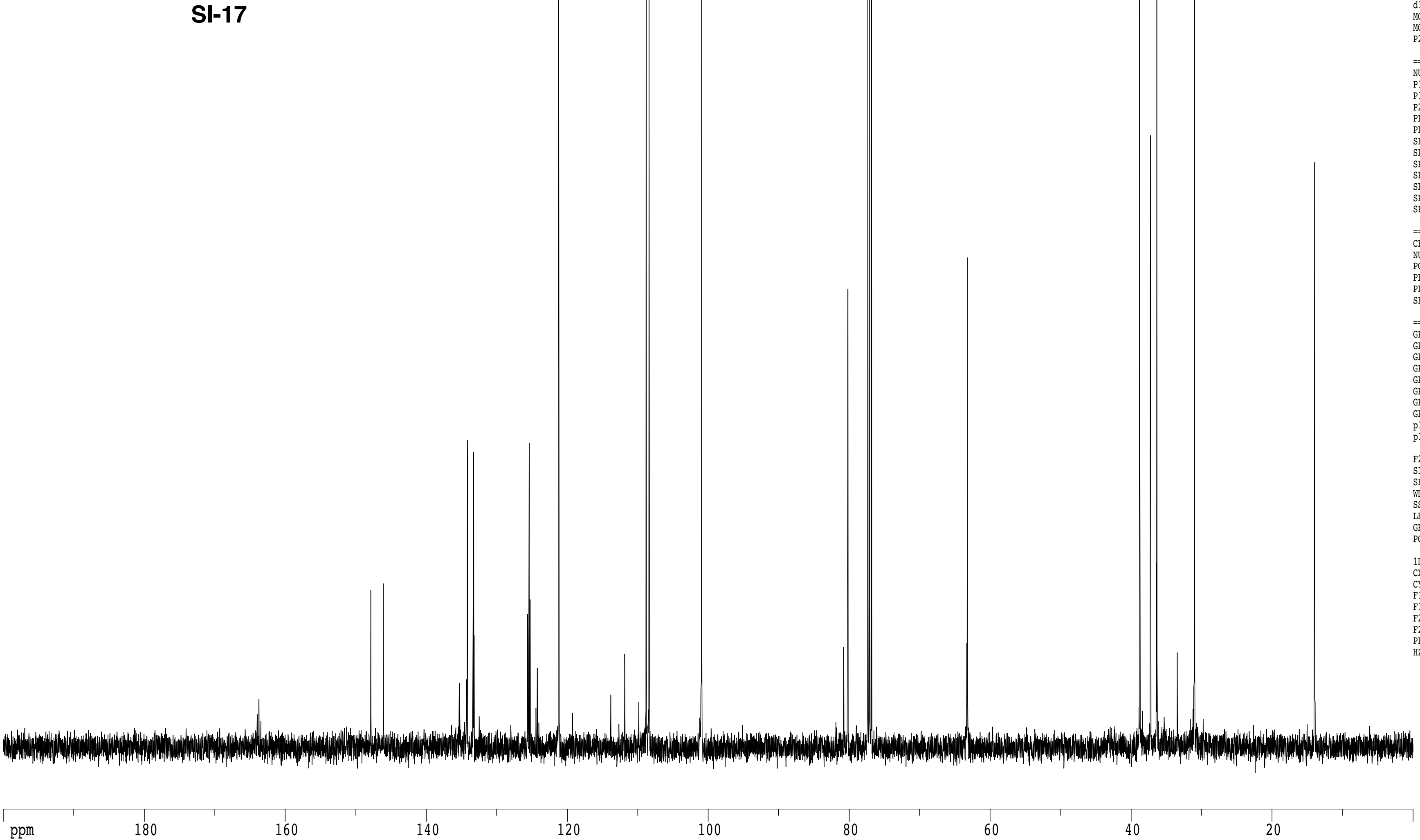


```
Current Data Parameters
USER          mcginnit
NAME          tmm-3-141
EXPNO               12
PROCNO               1

F2 - Acquisition Parameters
Date_         20210809
Time             15.58
INSTRUM        cryo500
PROBHD   5 mm CPTCI 1H-
PULPROG  SpinEchopg30gp2.prd
TD               65536
SOLVENT          CDCl3
NS                 318
DS                  16
SWH          30303.031 Hz
FIDRES        0.462388 Hz
AQ           1.0813940 sec
RG                8192
DW              16.500 usec
DE                6.00 usec
TE               298.0 K
D1          0.25000000 sec
d11         0.03000000 sec
D16         0.00020000 sec
d17         0.00019600 sec
MCREST      0.00000000 sec
MCWRK       0.01500000 sec
P2               37.70 usec

======== CHANNEL f1 ========
NUC1               13C
P1               18.85 usec
P12            2000.00 usec
P20             500.00 usec
PL0             120.00 dB
PL1              -1.00 dB
SFO1       125.7942548 MHz
SP2               1.55 dB
SP4               1.55 dB
SPNAM2     Crp60comp.4
SPNAM4   Crp60,0.5,20.1
SPOFF2            0.00 Hz
SPOFF4            0.00 Hz

======== CHANNEL f2 ========
CPDPRG2        waltz16
NUC2                1H
PCPD2           100.00 usec
PL2               1.60 dB
PL12             22.00 dB
SFO2       500.2225011 MHz

====== GRADIENT CHANNEL =====
GPNAM1        SINE.100
GPNAM2        SINE.100
GPX1              0.00 %
GPX2              0.00 %
GPY1              0.00 %
GPY2              0.00 %
GPZ1             30.00 %
GPZ2             50.00 %
p15             500.00 usec
p16            1000.00 usec

F2 - Processing parameters
SI               65536
SF         125.7804190 MHz
WDW                 EM
SSB                  0
LB                1.00 Hz
GB                   0
PC                2.00

1D NMR plot parameters
CX               22.80 cm
CY               15.65 cm
F1P            200.000 ppm
F1            25156.08 Hz
F2P              0.000 ppm
F2                0.00 Hz
PPMCM          8.77193 ppm/cm
HZCM        1103.33704 Hz/cm
```

19F spectrum

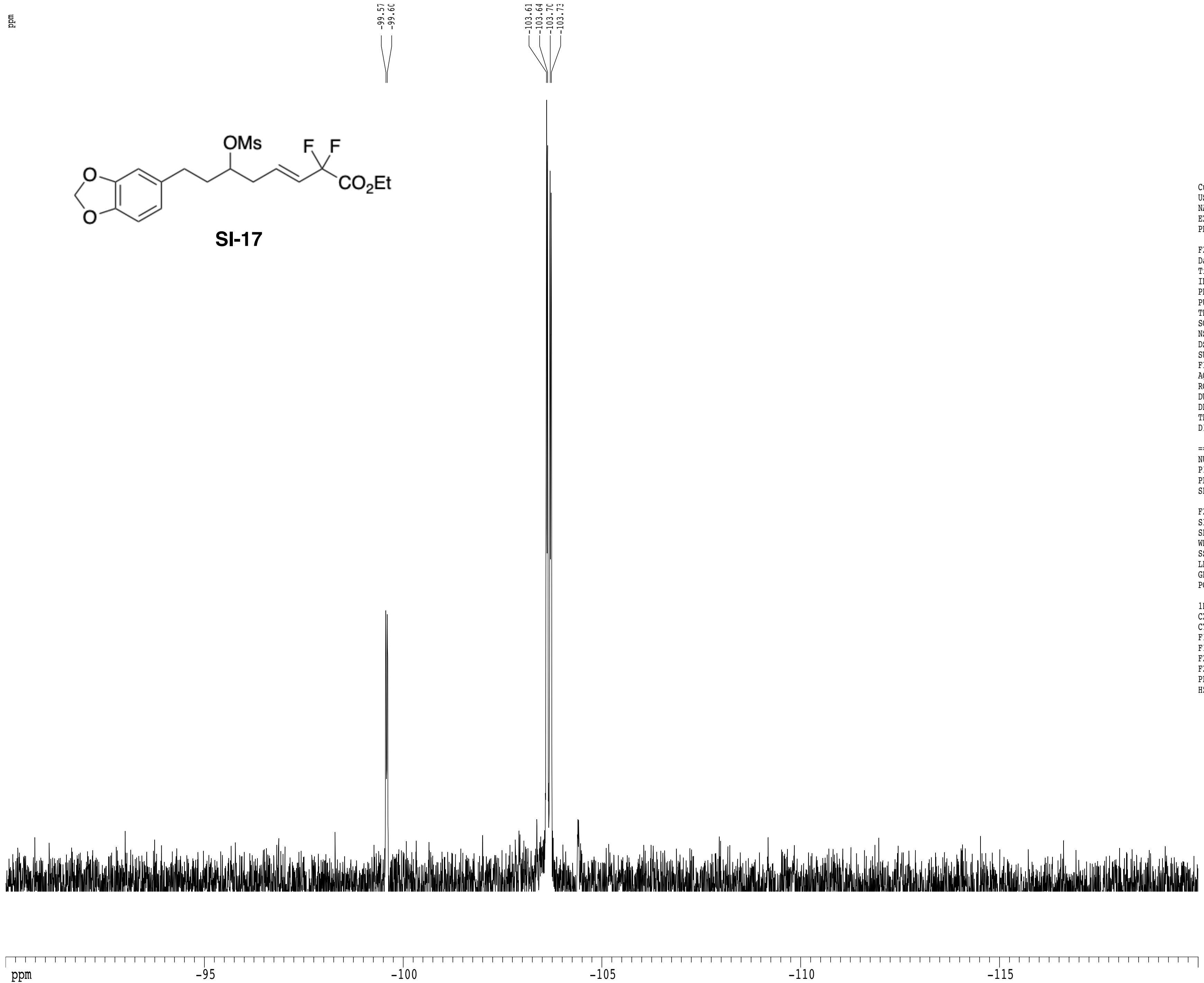


SI-17

Current Data Parameters
USER mcginnit
NAME tmm-3-141
EXPNO 11
PROCNO 1

F2 - Acquisition Parameters
Date_ 20210809
Time 15.36
INSTRUM drx400
PROBHD 5 mm QNP H/F/P
PULPROG zgflqn30
TD 65536
SOLVENT CDCl3
NS 93
DS 2
SWH 75187.969 Hz
FIDRES 1.147277 Hz
AQ 0.4358644 sec
RG 6502
DW 6.650 usec
DE 9.46 usec
TE 298.0 K
D1 2.00000000 sec

======== CHANNEL f1 ========
NUC1 19F
P1 11.75 usec
PL1 -6.00 dB
SFO1 376.4646491 MHz

F2 - Processing parameters
SI 65536
SF 376.4984640 MHz
WDW EM
SSB 0
LB 1.00 Hz
GB 0
PC 1.00

1D NMR plot parameters
CX 22.80 cm
CY 15.00 cm
F1P -90.000 ppm
F1 -33884.86 Hz
F2P -120.000 ppm
F2 -45179.82 Hz
PPMCM 1.31579 ppm/cm
HZCM 495.39273 Hz/cm

1H spectrum

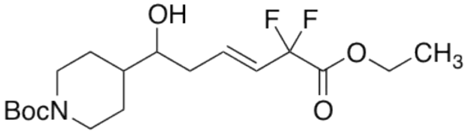

**SI-37**

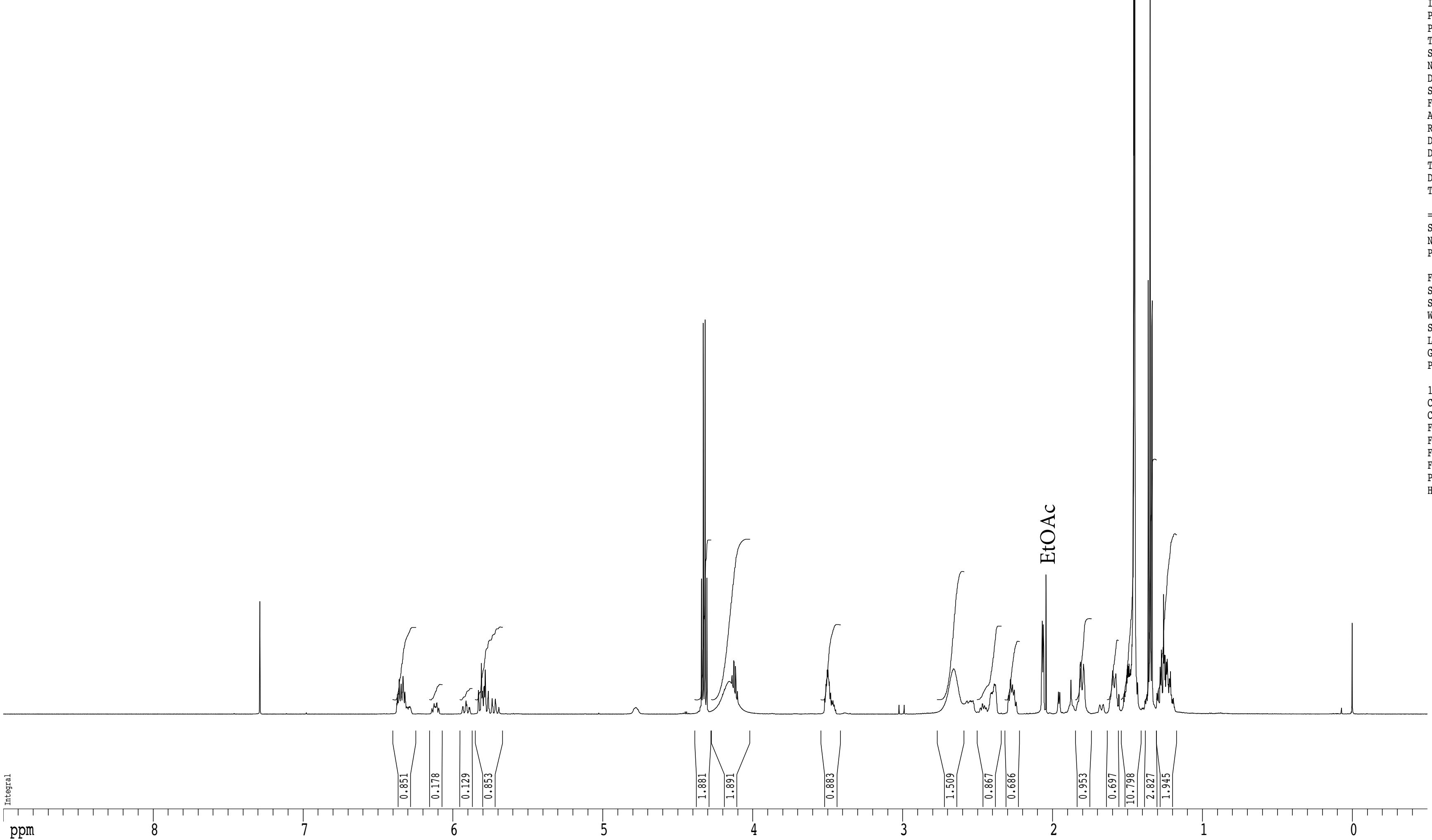


```
Current Data Parameters
USER            linpc2
NAME         pcl-2-204
EXPNO                1
PROCNO               1

F2 - Acquisition Parameters
Date_         20211012
Time             16.36
INSTRUM          av600
PROBHD   5 mm CPBBO BB-
PULPROG           zg30
TD               98074
SOLVENT         CDCl3T
NS                   8
DS                   2
SWH           9615.385 Hz
FIDRES        0.098042 Hz
AQ           5.0998979 sec
RG                  10
DW              52.000 usec
DE               14.23 usec
TE               298.0 K
D1          0.10000000 sec
TD0                  1

======== CHANNEL f1 ========
SFO1       600.1342009 MHz
NUC1                1H
P1                9.50 usec

F2 - Processing parameters
SI               65536
SF         600.1300194 MHz
WDW                 no
SSB                  0
LB                0.00 Hz
GB                   0
PC                1.00

1D NMR plot parameters
CX               22.80 cm
CY               45.00 cm
F1P              9.000 ppm
F1             5401.17 Hz
F2P             -0.500 ppm
F2             -300.06 Hz
PPMCM          0.41667 ppm/cm
HZCM          250.05418 Hz/cm
```

19F spectrum

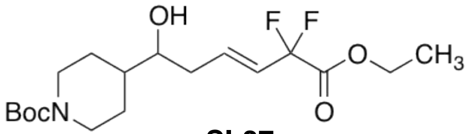

SI-37

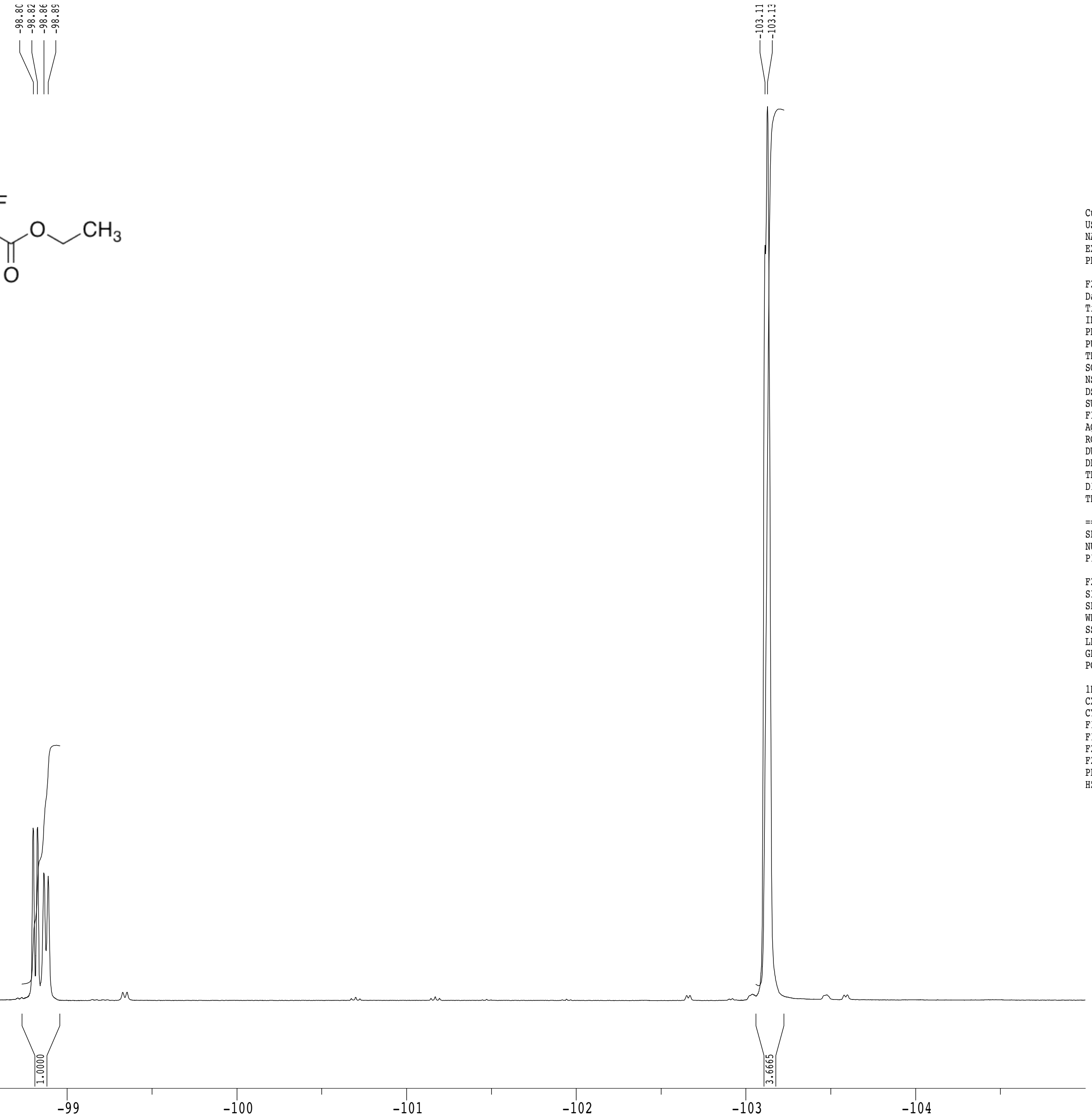


Current Data Parameters
USER linpc2
NAME pcl-2-204
EXPNO 2
PROCNO 1

F2 - Acquisition Parameters
Date_ 20211012
Time 16.41
INSTRUM av600
PROBHD 5 mm CPBBO BB-
PULPROG zgflqn30
TD 131072
SOLVENT CDCl3T
NS 16
DS 2
SWH 178571.422 Hz
FIDRES 1.362392 Hz
AQ 0.3670516 sec
RG 575
DW 2.800 usec
DE 18.00 usec
TE 298.0 K
D1 3.00000000 sec
TD0 1

======== CHANNEL f1 ========
SFO1 564.6299196 MHz
NUC1 19F
P1 18.25 usec

F2 - Processing parameters
SI 131072
SF 564.6863858 MHz
WDW no
SSB 0
LB 0.00 Hz
GB 0
PC 1.00

1D NMR plot parameters
CX 22.80 cm
CY 15.00 cm
F1P -97.000 ppm
F1 -54774.58 Hz
F2P -105.000 ppm
F2 -59292.07 Hz
PPMCM 0.35088 ppm/cm
HZCM 198.13559 Hz/cm

1H spectrum

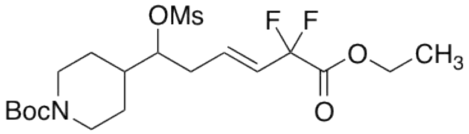


**SI-18**

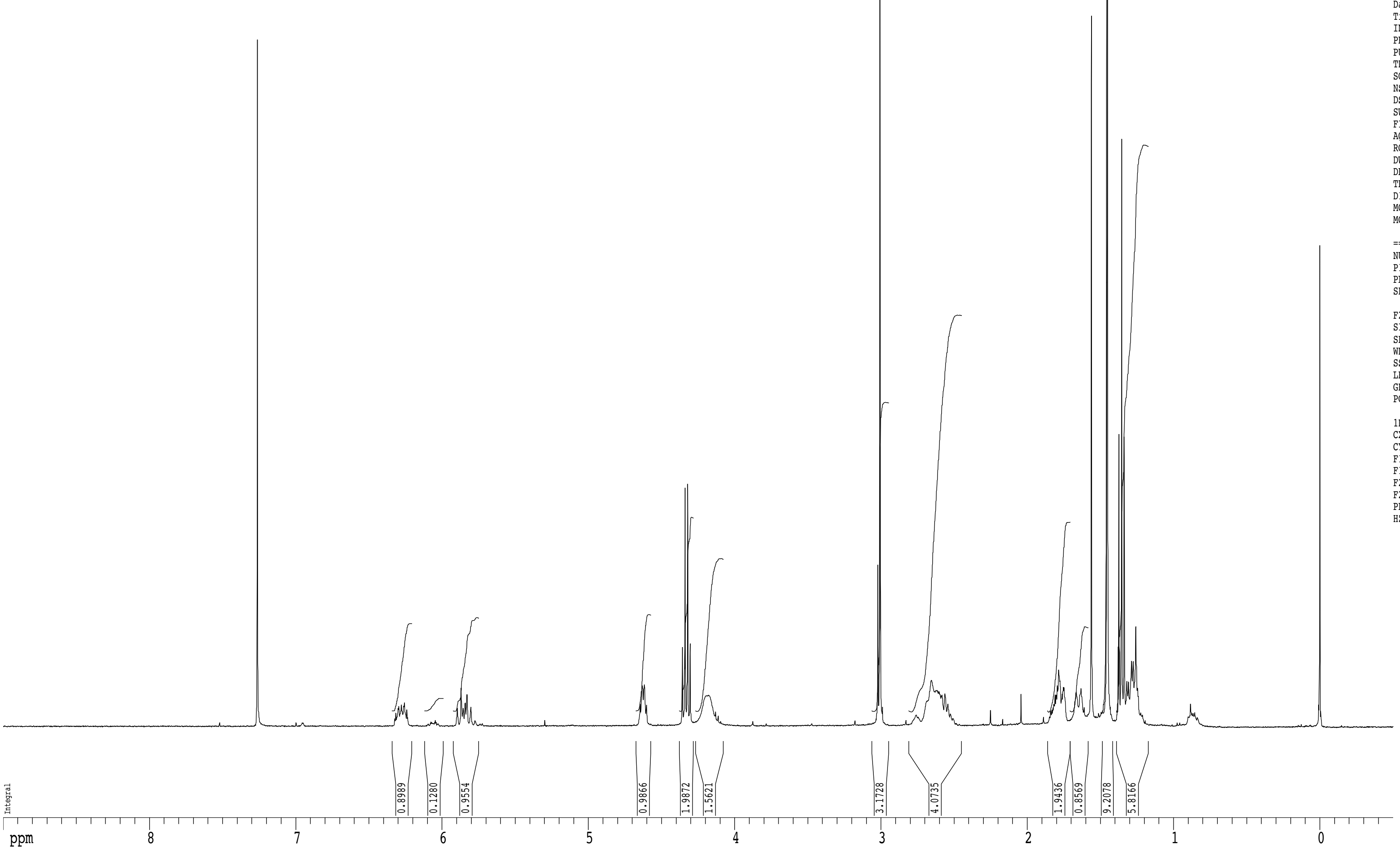


Current Data Parameters
USER linpc2
NAME pcl-2-099
EXPNO 5
PROCNO 1

F2 - Acquisition Parameters
Date_ 20210715
Time 10.02
INSTRUM drx400
PROBHD 5 mm QNP H/F/P
PULPROG zg30
TD 65536
SOLVENT CDCl3
NS 8
DS 2
SWH 6410.256 Hz
FIDRES 0.097813 Hz
AQ 5.1118579 sec
RG 456.1
DW 78.000 usec
DE 4.50 usec
TE 298.0 K
D1 0.10000000 sec
MCREST 0.00000000 sec
MCWRK 0.01500000 sec

======== CHANNEL f1 ========
NUC1 1H
P1 12.00 usec
PL1 -1.60 dB
SFO1 400.1328009 MHz

F2 - Processing parameters
SI 65536
SF 400.1300209 MHz
WDW EM
SSB 0
LB 0.30 Hz
GB 0
PC 2.00

1D NMR plot parameters
CX 22.80 cm
CY 45.00 cm
F1P 9.000 ppm
F1 3601.17 Hz
F2P -0.500 ppm
F2 -200.06 Hz
PPMCM 0.41667 ppm/cm
HZCM 166.72086 Hz/cm

Z-restored spin-echo 13C spectrum with 1H decoupling

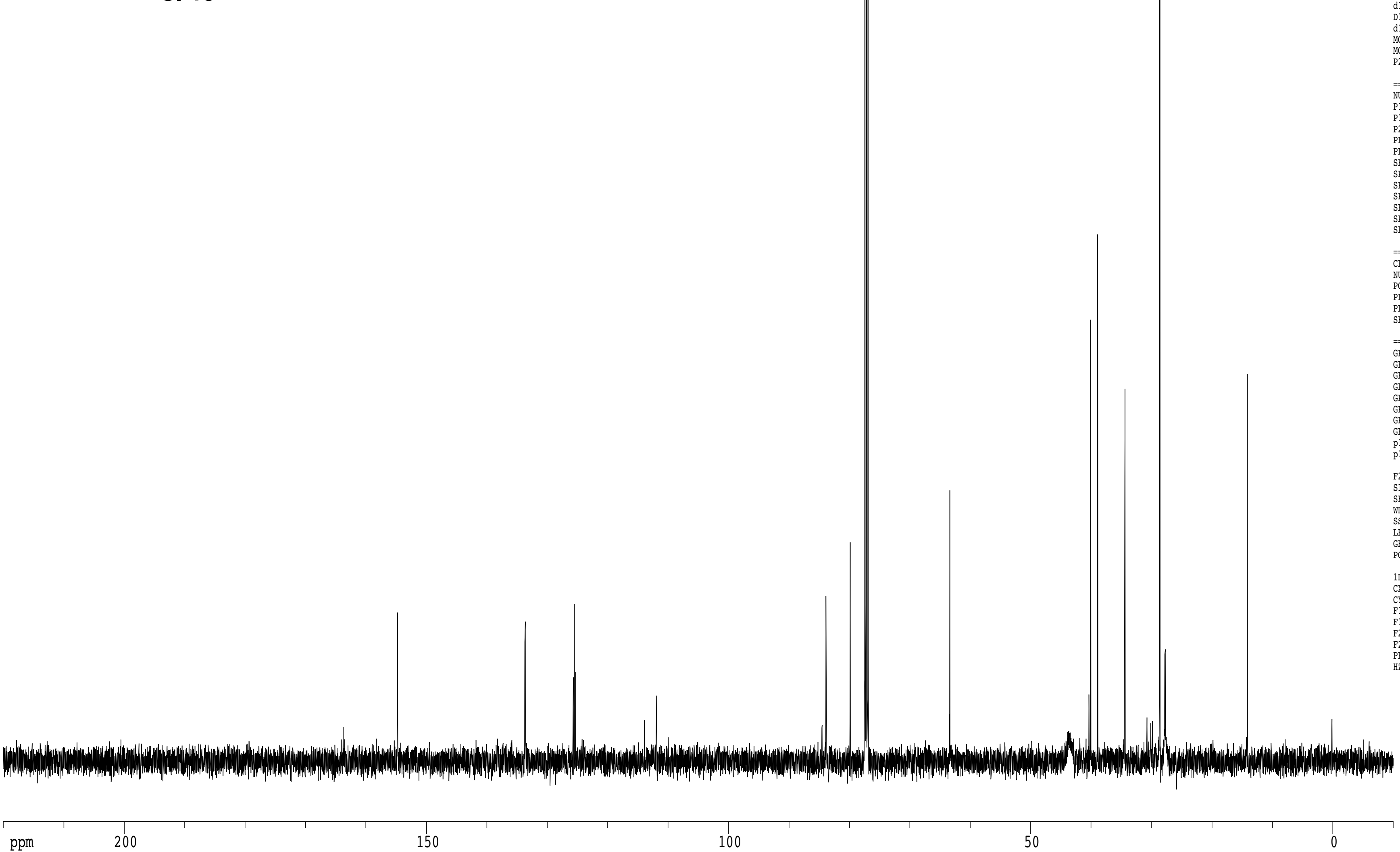


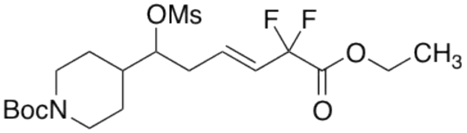


**SI-18**

Current Data Parameters
USER linpc2
NAME pcl-2-099
EXPNO 8
PROCNO 1

F2 - Acquisition Parameters
Date_ 20210716
Time 8.28
INSTRUM cryo500
PROBHD 5 mm CPTCI 1H-
PULPROG SpinEchopg30gp2.prd
TD 65536
SOLVENT CDCl3
NS 1024
DS 16
SWH 30303.031 Hz
FIDRES 0.462388 Hz
AQ 1.0813940 sec
RG 5160.6
DW 16.500 usec
DE 6.00 usec
TE 298.0 K
D1 0.25000000 sec
d11 0.03000000 sec
D16 0.00020000 sec
d17 0.00019600 sec
MCREST 0.00000000 sec
MCWRK 0.01500000 sec
P2 37.70 usec

======== CHANNEL f1 ========
NUC1 13C
P1 18.85 usec
P12 2000.00 usec
P20 500.00 usec
PL0 120.00 dB
PL1 -1.00 dB
SFO1 125.7942548 MHz
SP2 1.55 dB
SP4 1.55 dB
SPNAM2 Crp60comp.4
SPNAM4 Crp60,0.5,20.1
SPOFF2 0.00 Hz
SPOFF4 0.00 Hz

======== CHANNEL f2 ========
CPDPRG2 waltz16
NUC2 1H
PCPD2 100.00 usec
PL2 1.60 dB
PL12 22.00 dB
SFO2 500.2225011 MHz

====== GRADIENT CHANNEL =====
GPNAM1 SINE.100
GPNAM2 SINE.100
GPX1 0.00 %
GPX2 0.00 %
GPY1 0.00 %
GPY2 0.00 %
GPZ1 30.00 %
GPZ2 50.00 %
p15 500.00 usec
p16 1000.00 usec

F2 - Processing parameters
SI 65536
SF 125.7804062 MHz
WDW EM
SSB 0
LB 1.00 Hz
GB 0
PC 2.00

1D NMR plot parameters
CX 22.80 cm
CY 30.00 cm
F1P 220.000 ppm
F1 27671.69 Hz
F2P -10.000 ppm
F2 -1257.80 Hz
PPMCM 10.08772 ppm/cm
HZCM 1268.83740 Hz/cm

19F spectrum

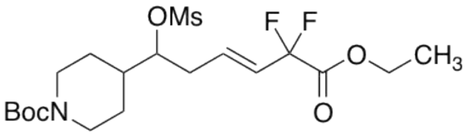


**SI-18**

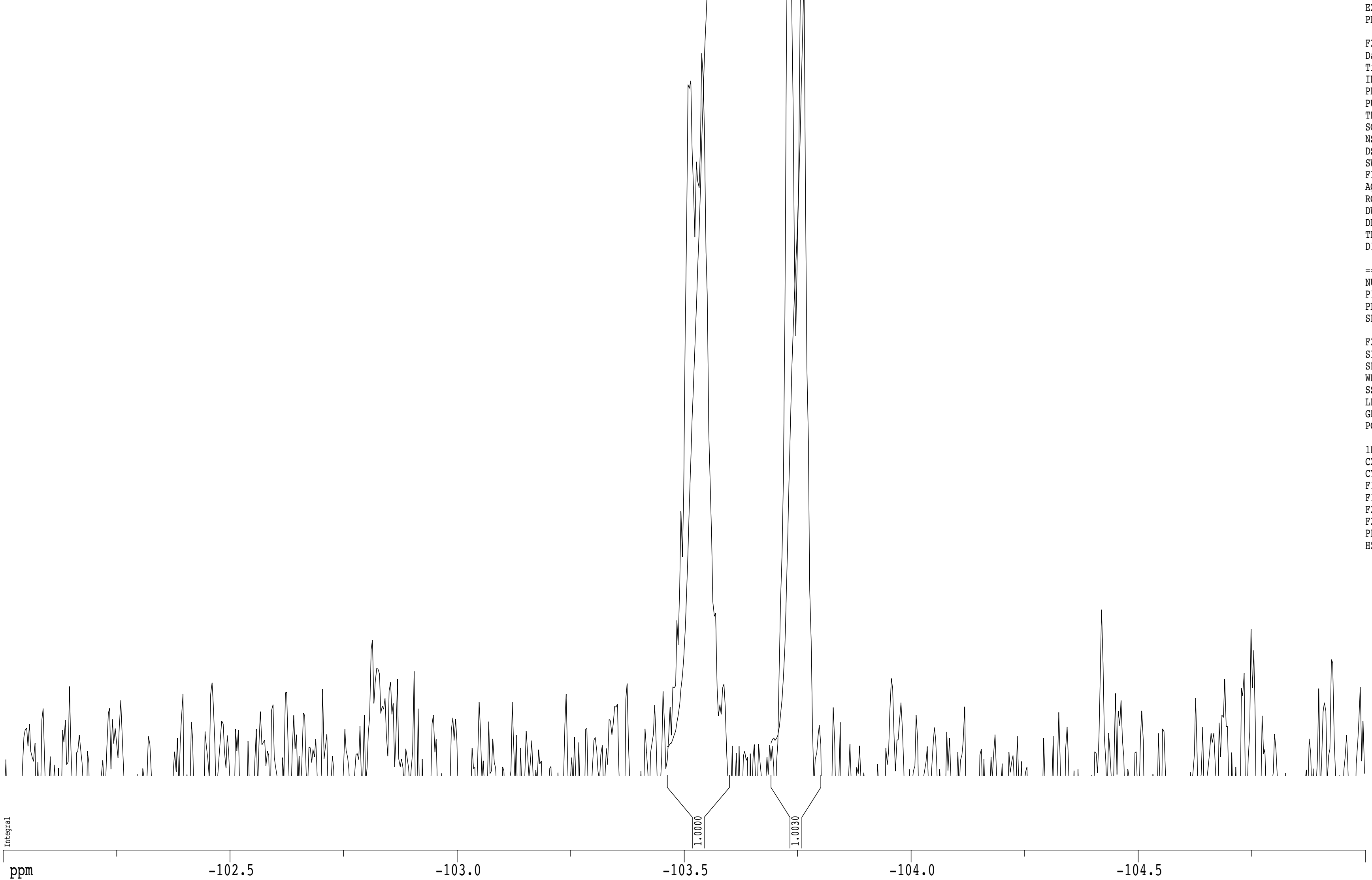


```
Current Data Parameters
USER            linpc2
NAME          pcl-2-099
EXPNO                4
PROCNO               1

F2 - Acquisition Parameters
Date_          20210715
Time              10.12
INSTRUM          drx400
PROBHD   5 mm QNP H/F/P
PULPROG        zgflqn30
TD                65536
SOLVENT           CDCl3
NS                   56
DS                    2
SWH           75187.969 Hz
FIDRES         1.147277 Hz
AQ            0.4358644 sec
RG                 6502
DW                6.650 usec
DE                 9.46 usec
TE                298.0 K
D1           2.00000000 sec

======== CHANNEL f1 ========
NUC1                19F
P1                11.75 usec
PL1               -6.00 dB
SFO1        376.4646491 MHz

F2 - Processing parameters
SI                65536
SF          376.4984640 MHz
WDW                  EM
SSB                   0
LB                 1.00 Hz
GB                    0
PC                 1.00

1D NMR plot parameters
CX                22.80 cm
CY                15.00 cm
F1P            -102.000 ppm
F1            -38402.84 Hz
F2P            -105.000 ppm
F2            -39532.34 Hz
PPMCM           0.13158 ppm/cm
HZCM           49.53928 Hz/cm
```

1H spectrum

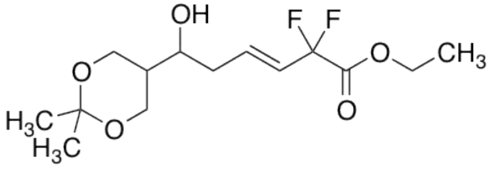

**SI-38**

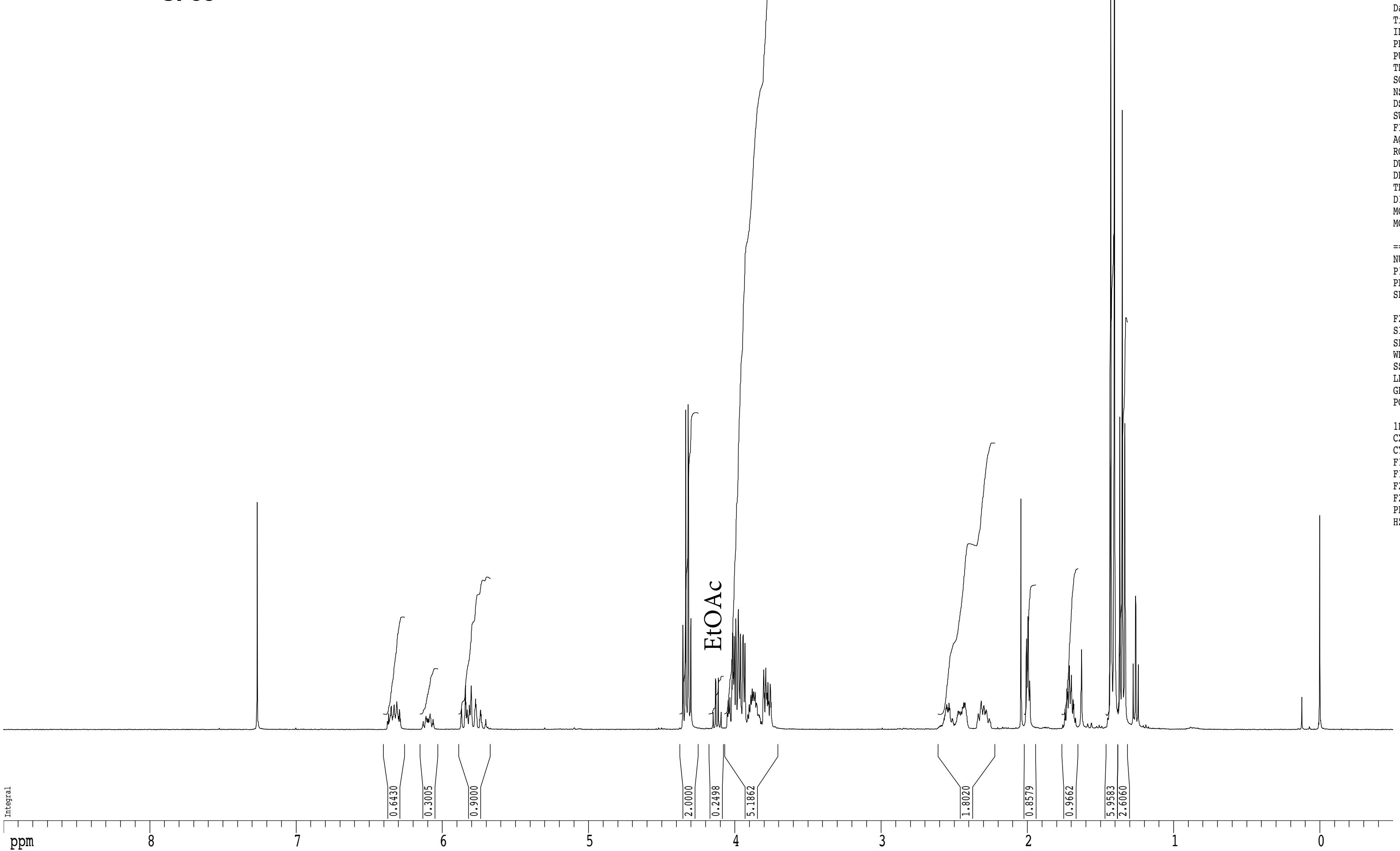


```
Current Data Parameters
USER              linpc2
NAME           pcl-1-254
EXPNO                  4
PROCNO                 1

F2 - Acquisition Parameters
Date_           20210226
Time               15.49
INSTRUM           drx400
PROBHD   5 mm QNP H/F/P
PULPROG             zg30
TD                 65536
SOLVENT            CDCl3
NS                     8
DS                     2
SWH             6410.256 Hz
FIDRES          0.097813 Hz
AQ             5.1118579 sec
RG                   256
DW                78.000 usec
DE                  4.50 usec
TE                 298.0 K
D1            0.10000000 sec
MCREST        0.00000000 sec
MCWRK         0.01500000 sec

======== CHANNEL f1 ========
NUC1                  1H
P1                 12.00 usec
PL1                -1.60 dB
SFO1         400.1328009 MHz

F2 - Processing parameters
SI                 65536
SF           400.1300191 MHz
WDW                   EM
SSB                    0
LB                  0.30 Hz
GB                     0
PC                  2.00

1D NMR plot parameters
CX                 22.80 cm
CY                 15.00 cm
F1P                9.000 ppm
F1               3601.17 Hz
F2P               -0.500 ppm
F2               -200.06 Hz
PPMCM            0.41667 ppm/cm
HZCM            166.72084 Hz/cm
```

1H spectrum

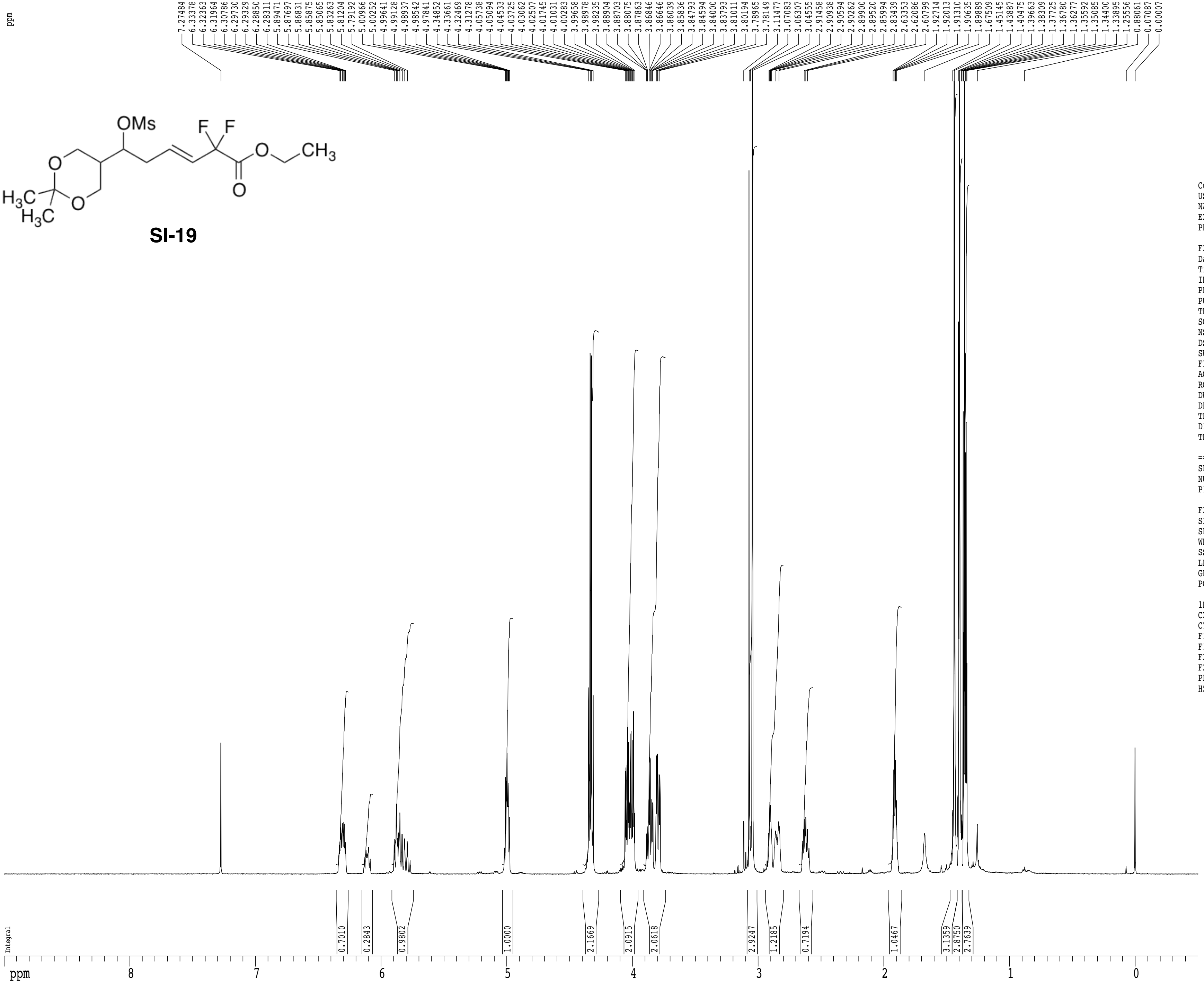

**SI-19**

Current Data Parameters
USER linpc2
NAME pcl-1-255
EXPNO 6
PROCNO 1

F2 - Acquisition Parameters
Date_ 20210816
Time 14.35
INSTRUM av600
PROBHD 5 mm CPBBO BB-
PULPROG zg30
TD 98074
SOLVENT CDCl3T
NS 8
DS 2
SWH 9615.385 Hz
FIDRES 0.098042 Hz
AQ 5.0998979 sec
RG 10
DW 52.000 usec
DE 14.23 usec
TE 298.0 K
D1 0.10000000 sec
TD0 1

======== CHANNEL f1 ========
SFO1 600.1342009 MHz
NUC1 1H
P1 9.50 usec

F2 - Processing parameters
SI 65536
SF 600.1300265 MHz
WDW no
SSB 0
LB 0.00 Hz
GB 0
PC 1.00

1D NMR plot parameters
CX 22.80 cm
CY 30.00 cm
F1P 9.000 ppm
F1 5401.17 Hz
F2P -0.500 ppm
F2 -300.06 Hz
PPMCM 0.41667 ppm/cm
HZCM 250.05418 Hz/cm

13C spectrum

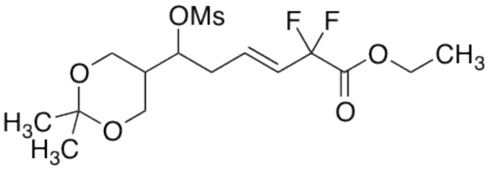


**SI-19**

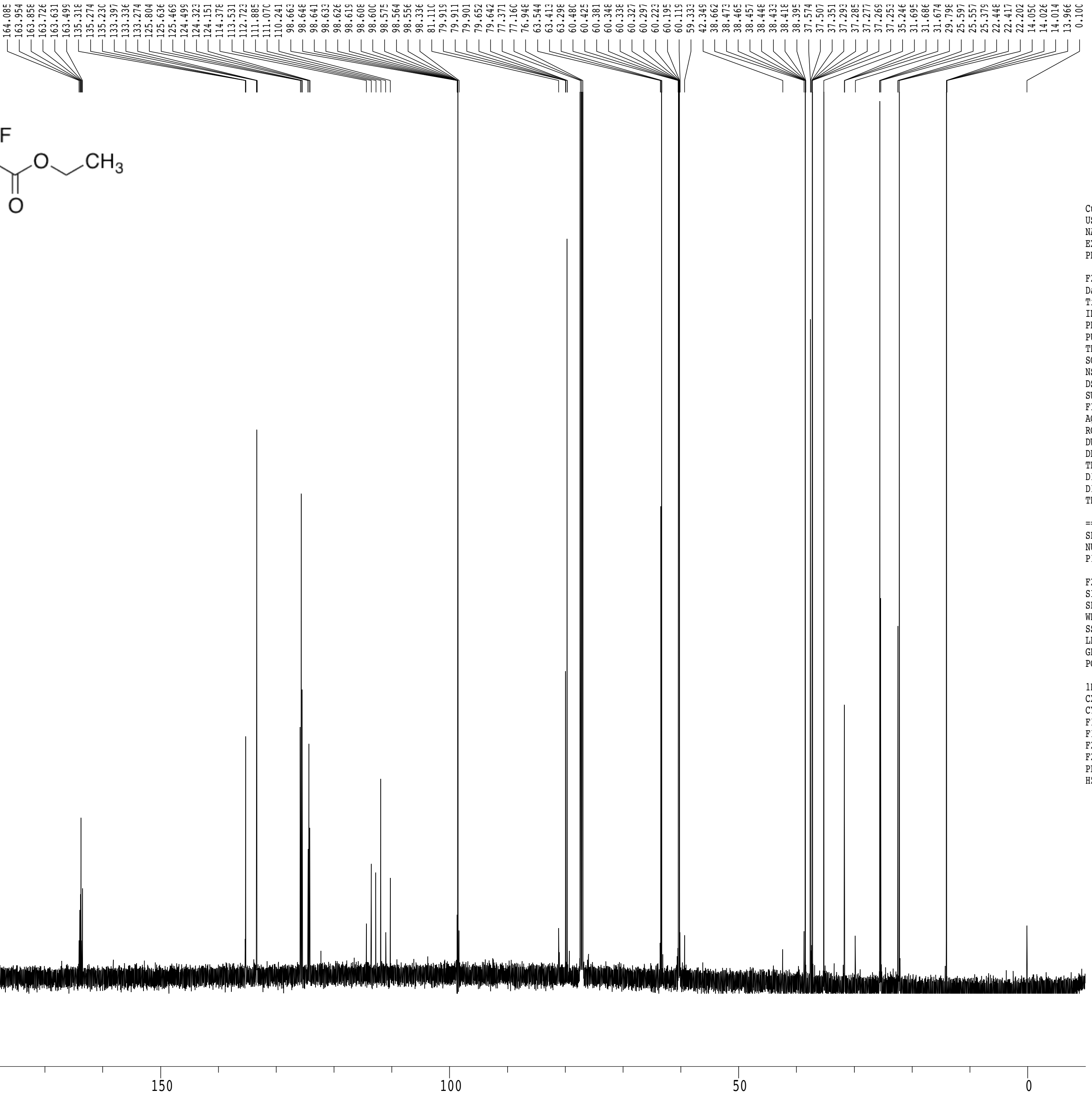


Current Data Parameters
USER linpc2
NAME pcl-1-255
EXPNO 7
PROCNO 1

F2 - Acquisition Parameters
Date_ 20210816
Time 14.38
INSTRUM av600
PROBHD 5 mm CPBBO BB-
PULPROG zgdc30
TD 65536
SOLVENT CDCl3T
NS 212
DS 4
SWH 36231.883 Hz
FIDRES 0.552855 Hz
AQ 0.9044468 sec
RG 2050
DW 13.800 usec
DE 19.63 usec
TE 298.0 K
D1 0.40000001 sec
D11 0.03000000 sec
TD0 1

======== CHANNEL f1 ========
SFO1 150.9194080 MHz
NUC1 13C
P1 10.10 usec

F2 - Processing parameters
SI 65536
SF 150.9027974 MHz
WDW no
SSB 0
LB 0.00 Hz
GB 0
PC 1.00

1D NMR plot parameters
CX 22.80 cm
CY 45.00 cm
F1P 220.000 ppm
F1 33198.62 Hz
F2P -10.000 ppm
F2 -1509.03 Hz
PPMCM 10.08772 ppm/cm
HZCM 1522.26514 Hz/cm

19F spectrum

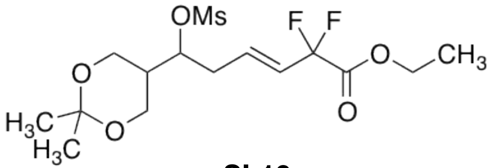


**SI-19**

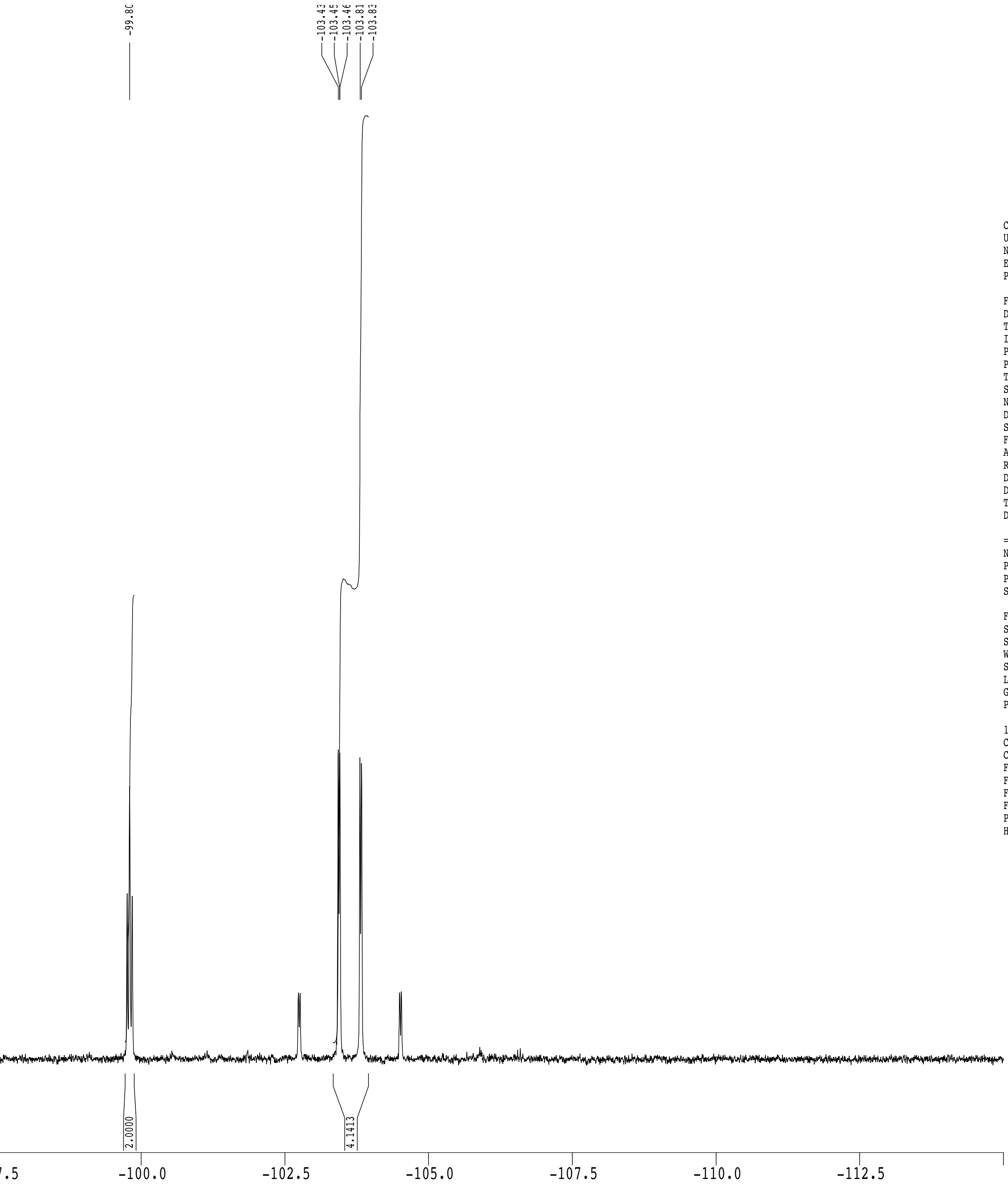


Current Data Parameters
USER linpc2
NAME pcl-1-255
EXPNO 5
PROCNO 1

F2 - Acquisition Parameters
Date_ 20210426
Time 8.09
INSTRUM drx400
PROBHD 5 mm QNP H/F/P
PULPROG zgflqn30
TD 65536
SOLVENT CDCl3
NS 24
DS 2
SWH 75187.969 Hz
FIDRES 1.147277 Hz
AQ 0.4358644 sec
RG 6502
DW 6.650 usec
DE 9.46 usec
TE 297.9 K
D1 2.00000000 sec

======== CHANNEL f1 ========
NUC1 19F
P1 11.75 usec
PL1 -6.00 dB
SFO1 376.4646491 MHz

F2 - Processing parameters
SI 65536
SF 376.4984640 MHz
WDW EM
SSB 0
LB 1.00 Hz
GB 0
PC 1.00

1D NMR plot parameters
CX 22.80 cm
CY 5.00 cm
F1P -90.000 ppm
F1 -33884.87 Hz
F2P -115.000 ppm
F2 -43297.33 Hz
PPMCM 1.09649 ppm/cm
HZCM 412.82730 Hz/cm

# 1H spectrum

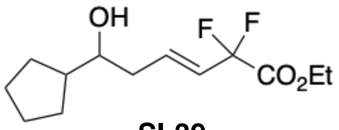


**SI-39**

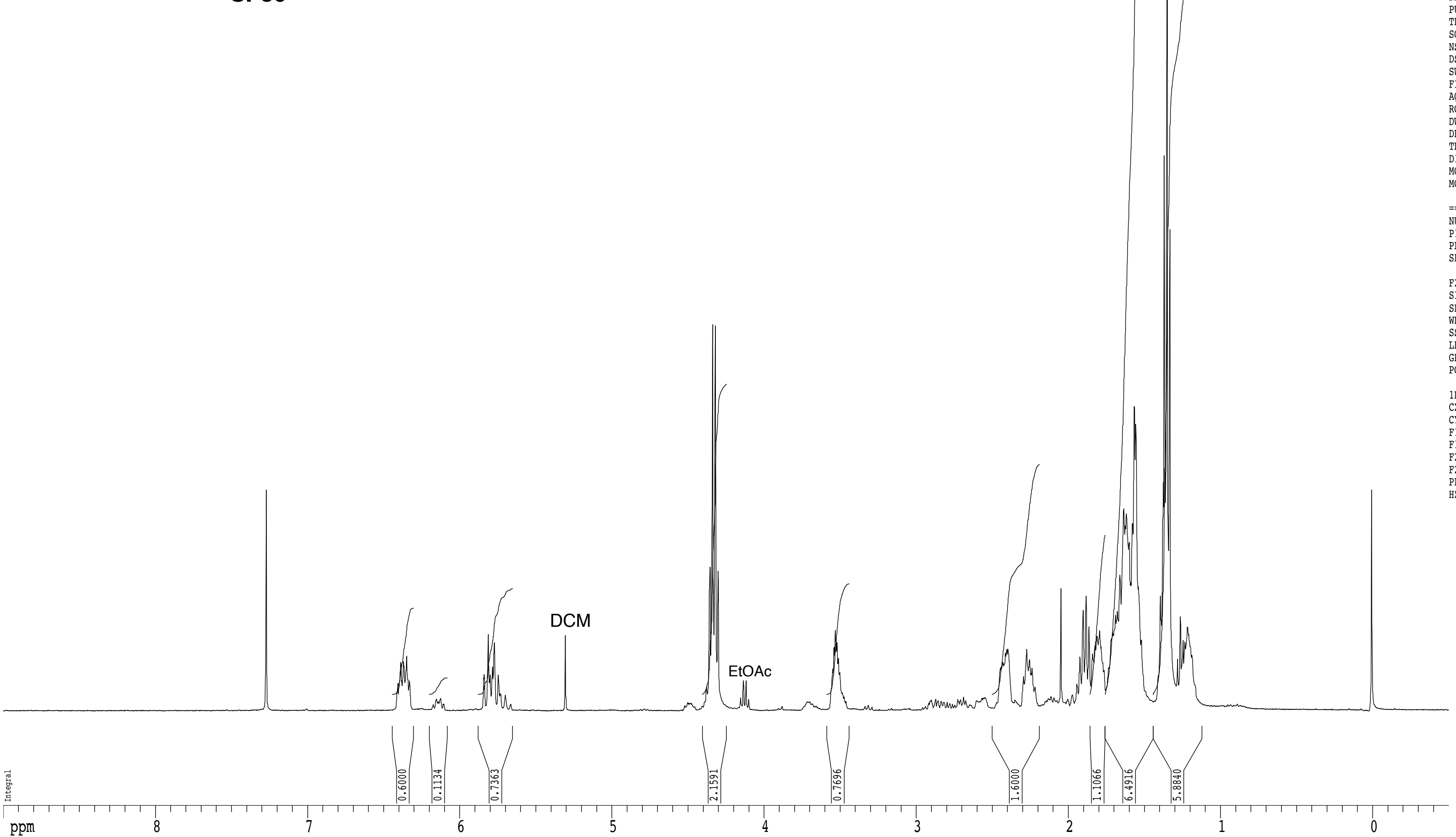


Current Data Parameters
USER mcginnit
NAME tmm-3-107
EXPNO 1
PROCNO 1

F2 - Acquisition Parameters
Date_ 20210427
Time 17.15
INSTRUM drx400
PROBHD 5 mm QNP H/F/P
PULPROG zg30
TD 65536
SOLVENT CDCl3
NS 8
DS 2
SWH 6410.256 Hz
FIDRES 0.097813 Hz
AQ 5.1118579 sec
RG 203.2
DW 78.000 usec
DE 4.50 usec
TE 298.0 K
D1 0.10000000 sec
MCREST 0.00000000 sec
MCWRK 0.01500000 sec

======== CHANNEL f1 ========
NUC1 1H
P1 12.00 usec
PL1 -1.60 dB
SFO1 400.1328009 MHz

F2 - Processing parameters
SI 65536
SF 400.1300175 MHz
WDW EM
SSB 0
LB 0.30 Hz
GB 0
PC 2.00

1D NMR plot parameters
CX 22.80 cm
CY 15.00 cm
F1P 9.000 ppm
F1 3601.17 Hz
F2P -0.500 ppm
F2 -200.06 Hz
PPMCM 0.41667 ppm/cm
HZCM 166.72084 Hz/cm

1H spectrum

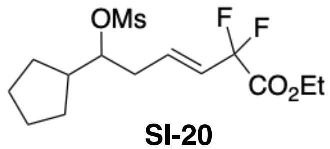


**SI-20**

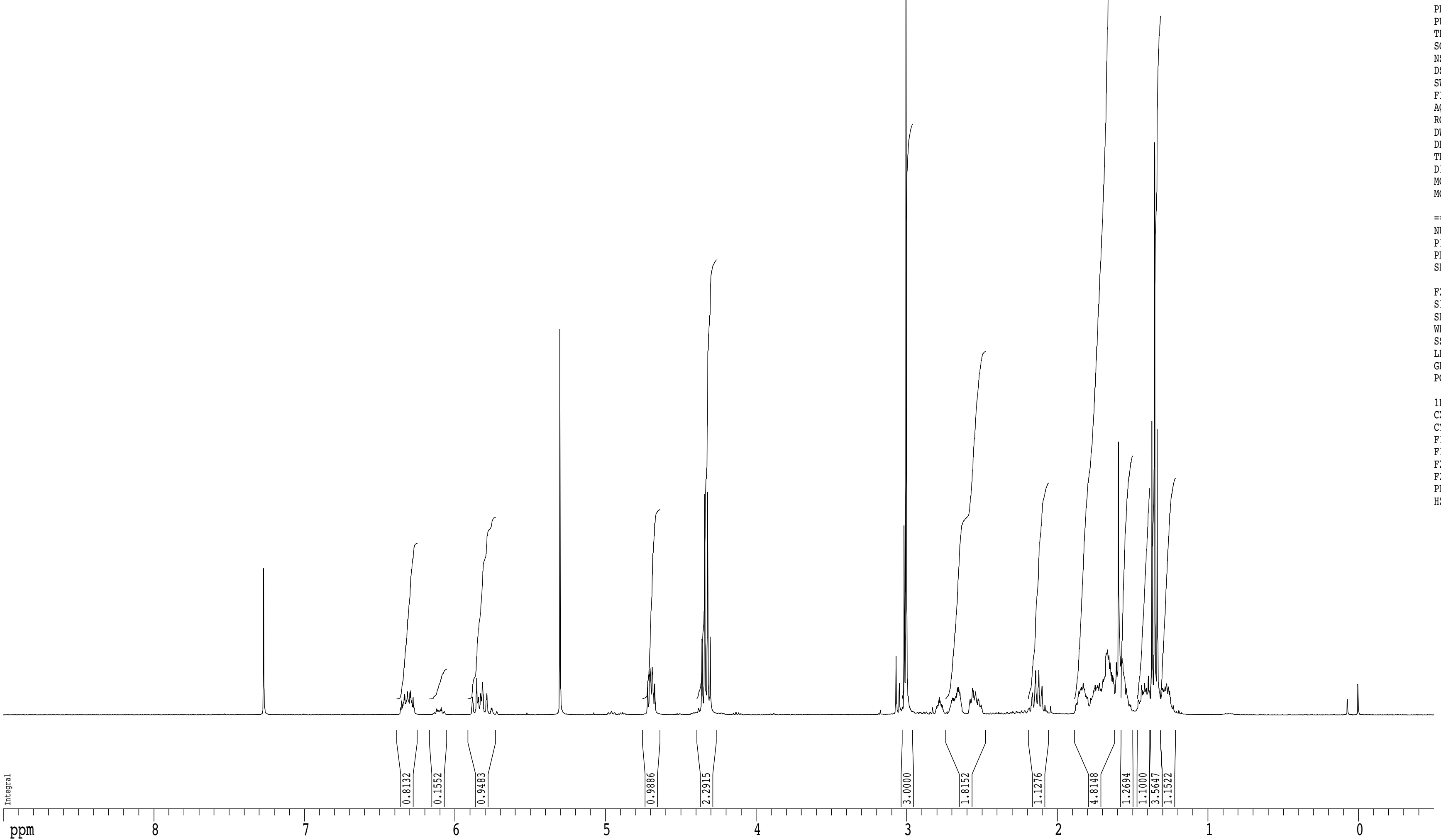


Current Data Parameters
USER mcginnit
NAME tmm-3-111
EXPNO 10
PROCNO 1

F2 - Acquisition Parameters
Date_ 20210809
Time 15.17
INSTRUM drx400
PROBHD 5 mm QNP H/F/P
PULPROG zg30
TD 65536
SOLVENT CDCl3
NS 8
DS 2
SWH 6410.256 Hz
FIDRES 0.097813 Hz
AQ 5.1118579 sec
RG 203.2
DW 78.000 usec
DE 4.50 usec
TE 298.0 K
D1 0.10000000 sec
MCREST 0.00000000 sec
MCWRK 0.01500000 sec

======== CHANNEL f1 ========
NUC1 1H
P1 12.00 usec
PL1 -1.60 dB
SFO1 400.1328009 MHz

F2 - Processing parameters
SI 65536
SF 400.1300175 MHz
WDW EM
SSB 0
LB 0.30 Hz
GB 0
PC 2.00

1D NMR plot parameters
CX 22.80 cm
CY 15.00 cm
F1P 9.000 ppm
F1 3601.17 Hz
F2P -0.500 ppm
F2 -200.06 Hz
PPMCM 0.41667 ppm/cm
HZCM 166.72084 Hz/cm

Z-restored spin-echo 13C spectrum with 1H decoupling

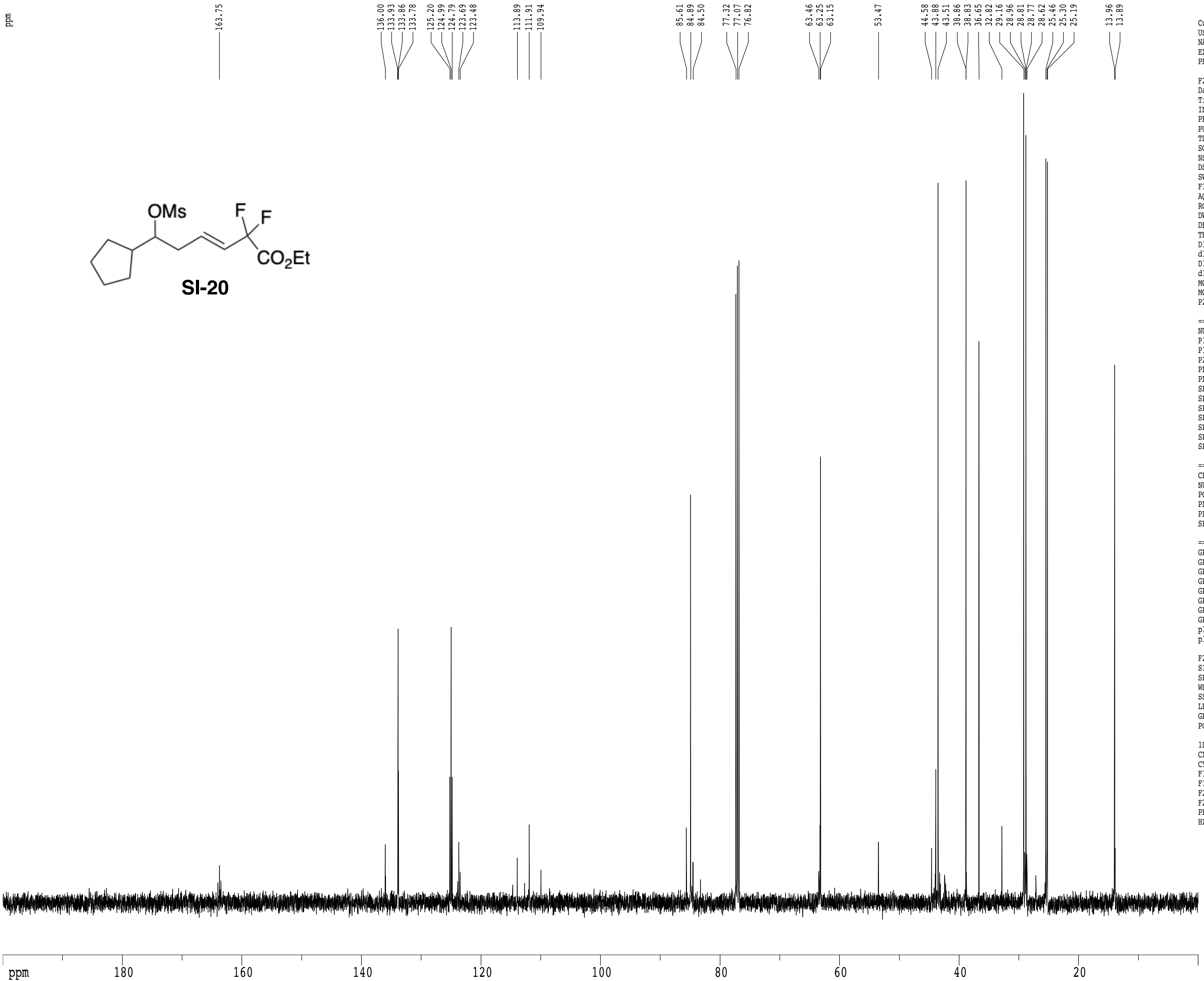


```
Current Data Parameters
USER           mcginnit
NAME          tmm-3-111
EXPNO                12
PROCNO                1

F2 - Acquisition Parameters
Date_          20210809
Time              15.49
INSTRUM         cryo500
PROBHD   5 mm CPTCI 1H-
PULPROG  SpinEchopg30gp2.prd
TD                65536
SOLVENT           CDCl3
NS                  285
DS                   16
SWH          30303.031 Hz
FIDRES        0.462388 Hz
AQ           1.0813940 sec
RG                 3251
DW              16.500 usec
DE                6.00 usec
TE               298.0 K
D1          0.25000000 sec
d11         0.03000000 sec
D16         0.00020000 sec
d17         0.00019600 sec
MCREST      0.00000000 sec
MCWRK       0.01500000 sec
P2               37.70 usec

======== CHANNEL f1 ========
NUC1                13C
P1               18.85 usec
P12            2000.00 usec
P20             500.00 usec
PL0             120.00 dB
PL1              -1.00 dB
SFO1        125.7942548 MHz
SP2               1.55 dB
SP4               1.55 dB
SPNAM2      Crp60comp.4
SPNAM4   Crp60,0.5,20.1
SPOFF2             0.00 Hz
SPOFF4             0.00 Hz

======== CHANNEL f2 ========
CPDPRG2          waltz16
NUC2                  1H
PCPD2            100.00 usec
PL2                1.60 dB
PL12              22.00 dB
SFO2        500.2225011 MHz

====== GRADIENT CHANNEL =====
GPNAM1          SINE.100
GPNAM2          SINE.100
GPX1                0.00 %
GPX2                0.00 %
GPY1                0.00 %
GPY2                0.00 %
GPZ1               30.00 %
GPZ2               50.00 %
p15               500.00 usec
p16              1000.00 usec

F2 - Processing parameters
SI                 65536
SF          125.7804190 MHz
WDW                   EM
SSB                    0
LB                  1.00 Hz
GB                     0
PC                  2.00

1D NMR plot parameters
CX                 22.80 cm
CY                 15.65 cm
F1P              200.000 ppm
F1              25156.08 Hz
F2P                0.000 ppm
F2                  0.00 Hz
PPMCM            8.77193 ppm/cm
HZCM          1103.33704 Hz/cm
```

19F spectrum

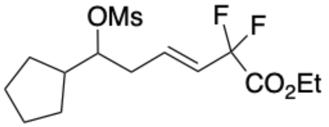


**SI-20**

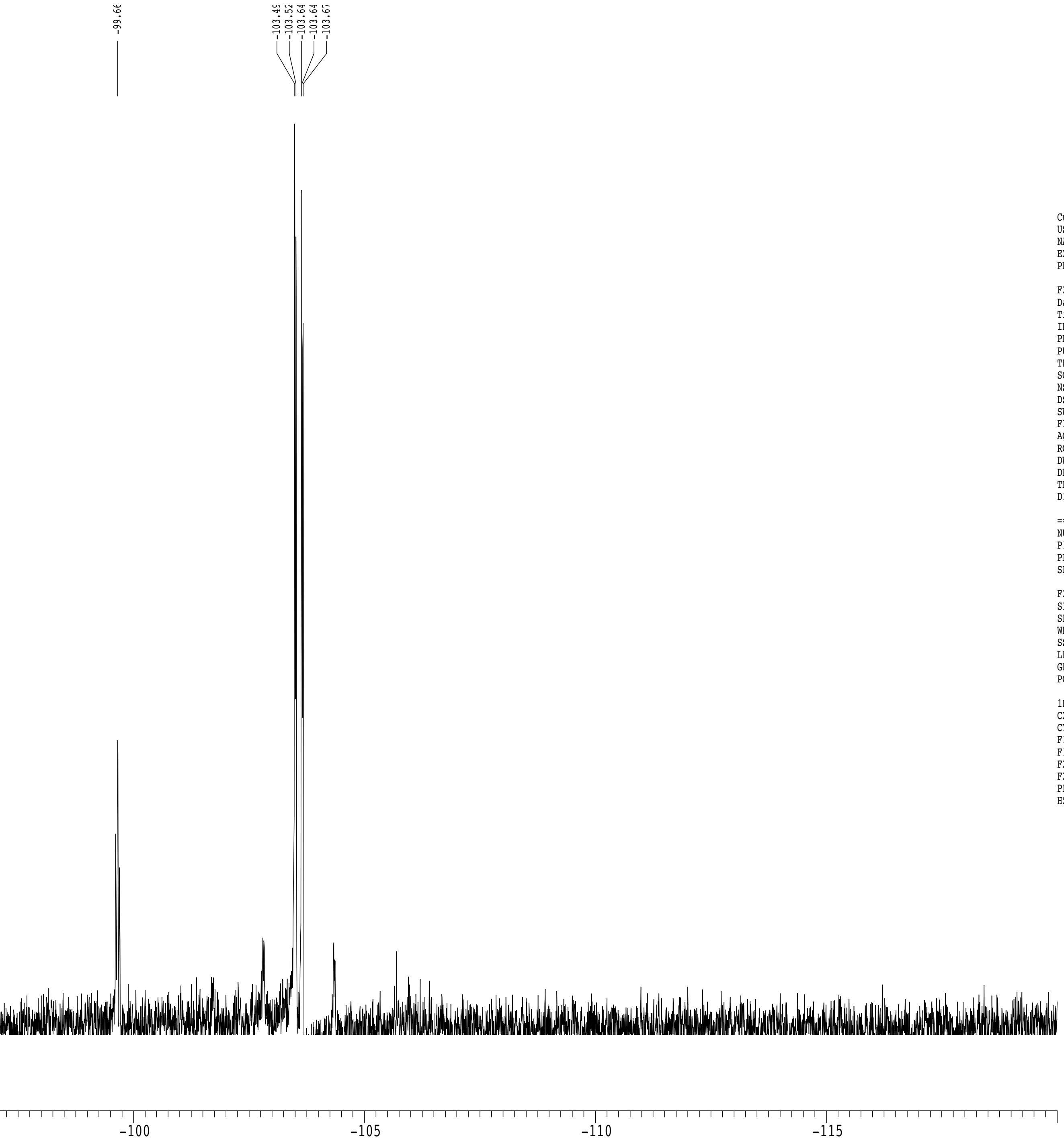


Current Data Parameters
USER mcginnit
NAME tmm-3-111
EXPNO 11
PROCNO 1

F2 - Acquisition Parameters
Date_ 20210809
Time 15.19
INSTRUM drx400
PROBHD 5 mm QNP H/F/P
PULPROG zgflqn30
TD 65536
SOLVENT CDCl3
NS 90
DS 2
SWH 75187.969 Hz
FIDRES 1.147277 Hz
AQ 0.4358644 sec
RG 6502
DW 6.650 usec
DE 9.46 usec
TE 298.0 K
D1 2.00000000 sec

======== CHANNEL f1 ========
NUC1 19F
P1 11.75 usec
PL1 -6.00 dB
SFO1 376.4646491 MHz

F2 - Processing parameters
SI 65536
SF 376.4984640 MHz
WDW EM
SSB 0
LB 1.00 Hz
GB 0
PC 1.00

1D NMR plot parameters
CX 22.80 cm
CY 15.00 cm
F1P -90.000 ppm
F1 -33884.86 Hz
F2P -120.000 ppm
F2 -45179.82 Hz
PPMCM 1.31579 ppm/cm
HZCM 495.39273 Hz/cm

1H spectrum

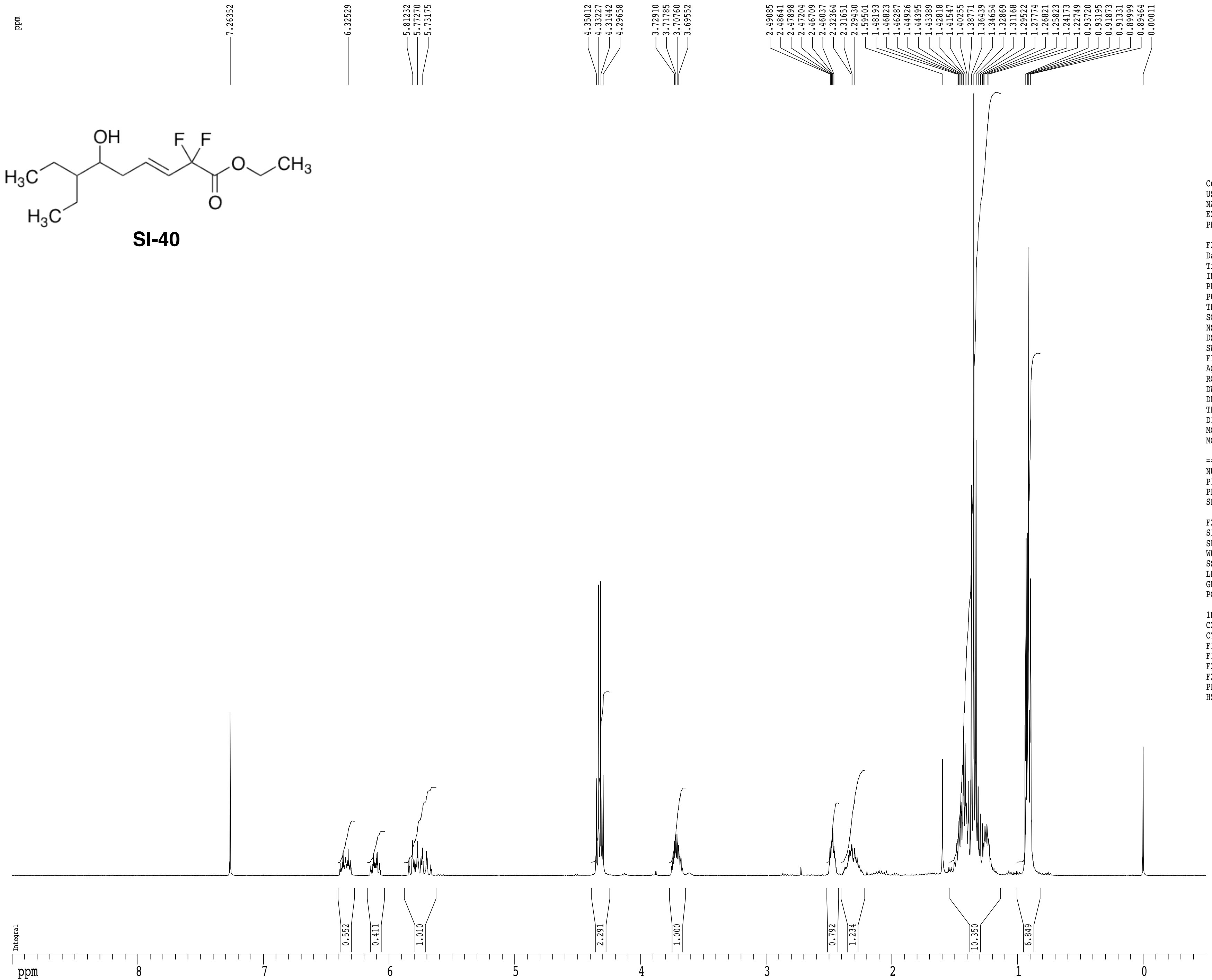


**SI-40**

Current Data Parameters
USER linpc2
NAME pcl-1-259
EXPNO 3
PROCNO 1

F2 - Acquisition Parameters
Date_ 20210305
Time 15.17
INSTRUM drx400
PROBHD 5 mm QNP H/F/P
PULPROG zg30
TD 65536
SOLVENT CDCl3
NS 8
DS 2
SWH 6410.256 Hz
FIDRES 0.097813 Hz
AQ 5.1118579 sec
RG 203.2
DW 78.000 usec
DE 4.50 usec
TE 298.0 K
D1 0.10000000 sec
MCREST 0.00000000 sec
MCWRK 0.01500000 sec

======== CHANNEL f1 ========
NUC1 1H
P1 12.00 usec
PL1 -1.60 dB
SFO1 400.1328009 MHz

F2 - Processing parameters
SI 65536
SF 400.1300197 MHz
WDW EM
SSB 0
LB 0.30 Hz
GB 0
PC 2.00

1D NMR plot parameters
CX 22.80 cm
CY 15.00 cm
F1P 9.000 ppm
F1 3601.17 Hz
F2P -0.500 ppm
F2 -200.06 Hz
PPMCM 0.41667 ppm/cm
HZCM 166.72084 Hz/cm

1H spectrum

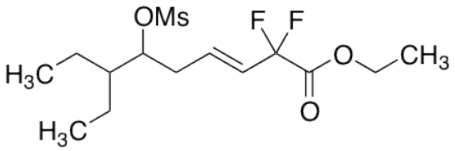


**SI-21**

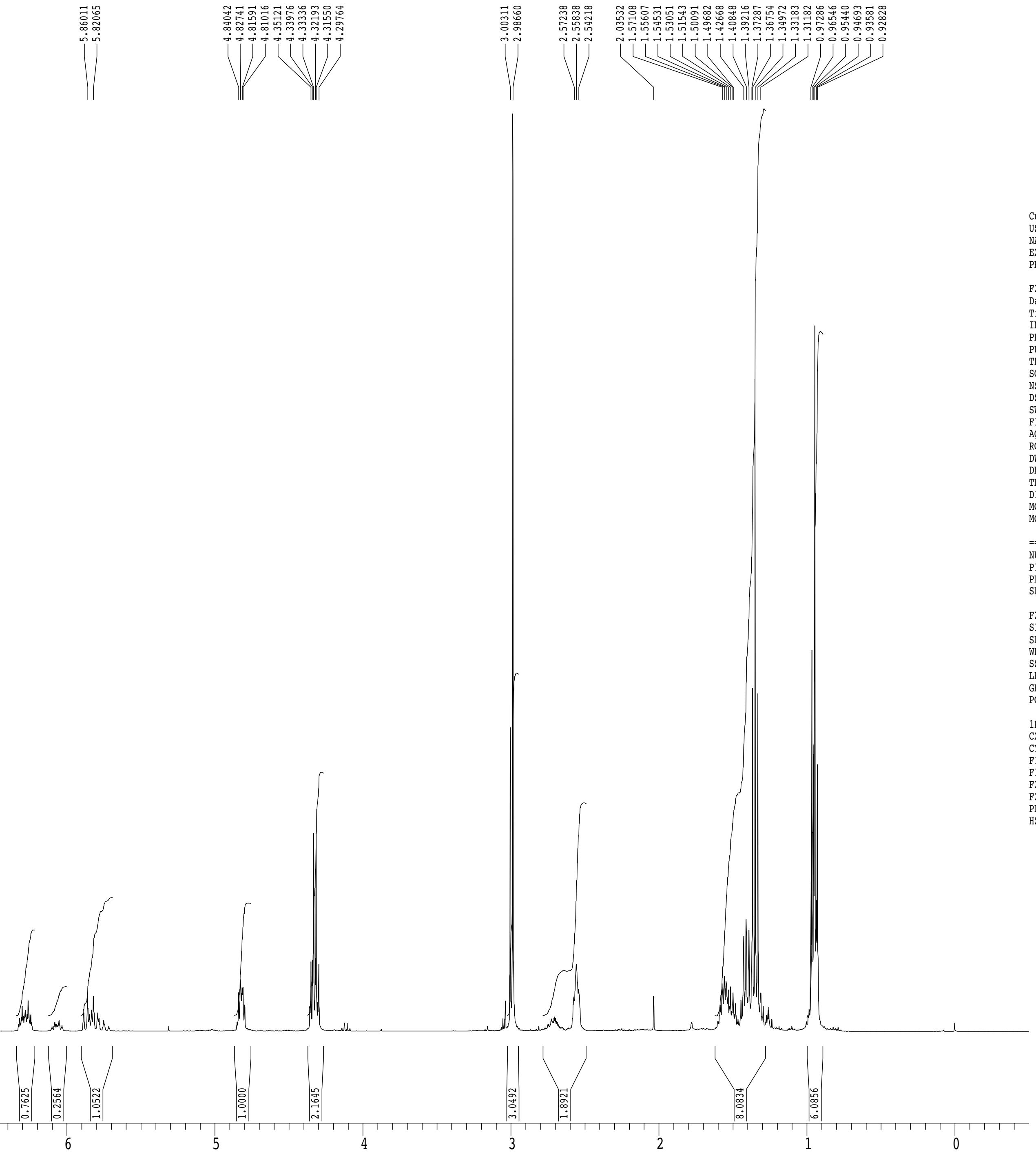


Current Data Parameters
USER linpc2
NAME pcl-1-260
EXPNO 6
PROCNO 1

F2 - Acquisition Parameters
Date_ 20210423
Time 15.12
INSTRUM drx400
PROBHD 5 mm QNP H/F/P
PULPROG zg30
TD 65536
SOLVENT CDCl3
NS 8
DS 2
SWH 6410.256 Hz
FIDRES 0.097813 Hz
AQ 5.1118579 sec
RG 32
DW 78.000 usec
DE 4.50 usec
TE 298.0 K
D1 0.10000000 sec
MCREST 0.00000000 sec
MCWRK 0.01500000 sec

======== CHANNEL f1 ========
NUC1 1H
P1 12.00 usec
PL1 -1.60 dB
SFO1 400.1328009 MHz

F2 - Processing parameters
SI 65536
SF 400.1300019 MHz
WDW EM
SSB 0
LB 0.30 Hz
GB 0
PC 2.00

1D NMR plot parameters
CX 22.80 cm
CY 15.00 cm
F1P 9.000 ppm
F1 3601.17 Hz
F2P -0.500 ppm
F2 -200.06 Hz
PPMCM 0.41667 ppm/cm
HZCM 166.72084 Hz/cm

13C spectrum with 1H decoupling

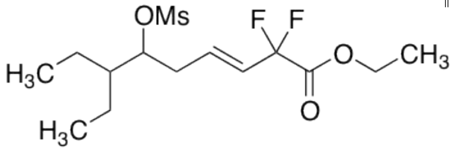


**SI-21**

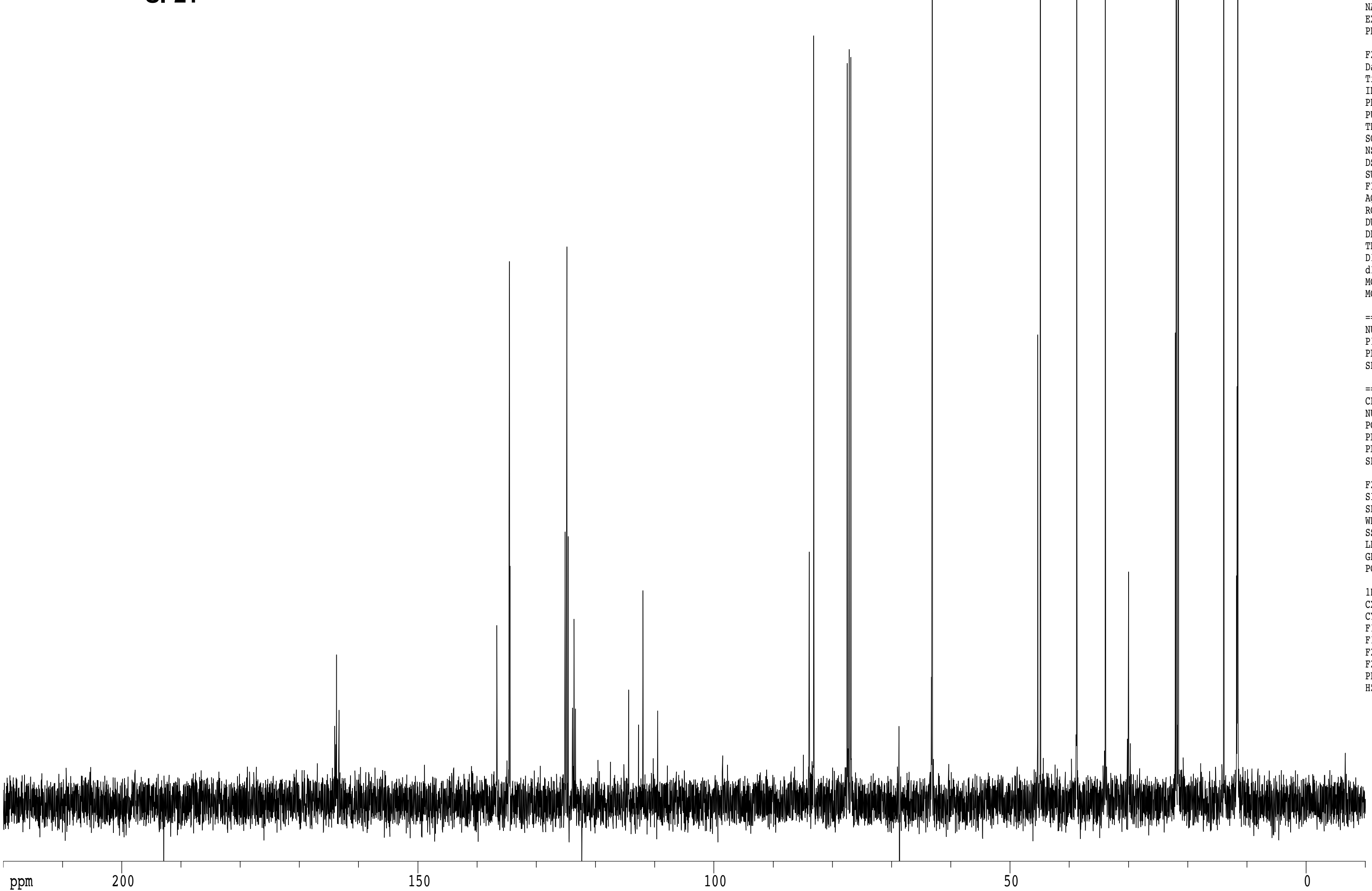


```
Current Data Parameters
USER              linpc2
NAME          pcl-1-260
EXPNO                  7
PROCNO                 1

F2 - Acquisition Parameters
Date_           20210423
Time               15.14
INSTRUM           drx400
PROBHD   5 mm QNP H/F/P
PULPROG          zgdc30
TD                 65536
SOLVENT            CDCl3
NS                    56
DS                     4
SWH          24154.590 Hz
FIDRES        0.368570 Hz
AQ           1.3566452 sec
RG                9195.2
DW              20.700 usec
DE               20.39 usec
TE               297.9 K
D1          0.10000000 sec
d11         0.03000000 sec
MCREST      0.00000000 sec
MCWRK       0.01500000 sec

======== CHANNEL f1 ========
NUC1                 13C
P1                  8.30 usec
PL1                -3.00 dB
SFO1         100.6237964 MHz

======== CHANNEL f2 ========
CPDPRG2          waltz16
NUC2                  1H
PCPD2              90.00 usec
PL2                -1.60 dB
PL12               16.50 dB
SFO2         400.1328009 MHz

F2 - Processing parameters
SI                 65536
SF           100.6127665 MHz
WDW                   EM
SSB                    0
LB                  1.00 Hz
GB                     0
PC                  1.00

1D NMR plot parameters
CX                 22.80 cm
CY                 30.00 cm
F1P              220.000 ppm
F1              22134.81 Hz
F2P              -10.000 ppm
F2              -1006.13 Hz
PPMCM           10.08772 ppm/cm
HZCM          1014.95343 Hz/cm
```

19F spectrum

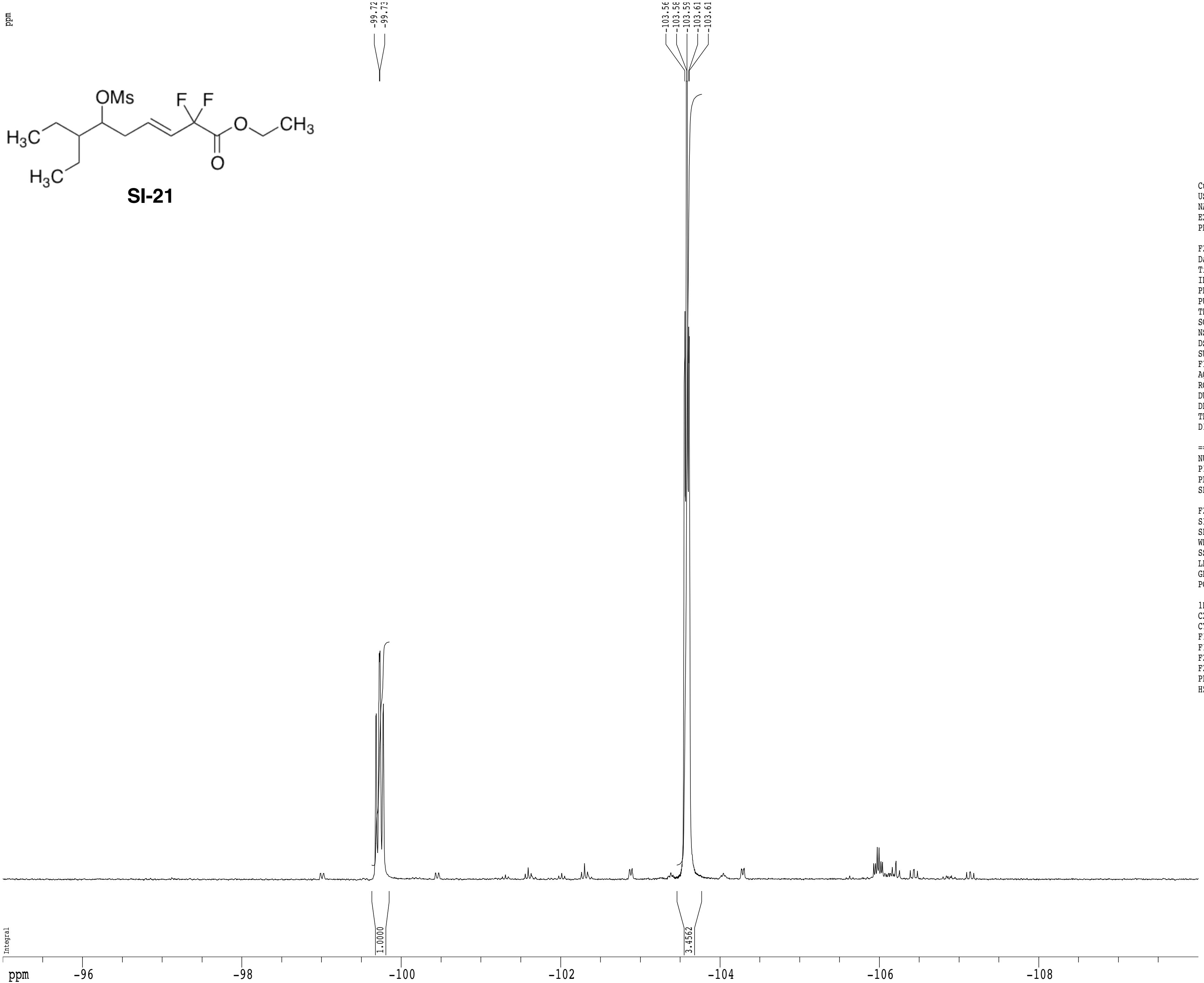


```
Current Data Parameters
USER              linpc2
NAME           pcl-1-260
EXPNO                  9
PROCNO                 1

F2 - Acquisition Parameters
Date_           20210423
Time               15.24
INSTRUM           drx400
PROBHD   5 mm QNP H/F/P
PULPROG         zgflqn30
TD                 65536
SOLVENT            CDCl3
NS                    96
DS                     2
SWH           75187.969 Hz
FIDRES         1.147277 Hz
AQ            0.4358644 sec
RG                  3251
DW                 6.650 usec
DE                  9.46 usec
TE                 298.0 K
D1            2.00000000 sec

======== CHANNEL f1 ========
NUC1                 19F
P1                 11.75 usec
PL1                -6.00 dB
SFO1         376.4646491 MHz

F2 - Processing parameters
SI                 65536
SF           376.4984640 MHz
WDW                   EM
SSB                    0
LB                  1.00 Hz
GB                     0
PC                  1.00

1D NMR plot parameters
CX                 22.80 cm
CY                 20.00 cm
F1P              -95.000 ppm
F1             -35767.36 Hz
F2P             -110.000 ppm
F2             -41414.83 Hz
PPMCM            0.65789 ppm/cm
HZCM           247.69637 Hz/cm
```

1H spectrum

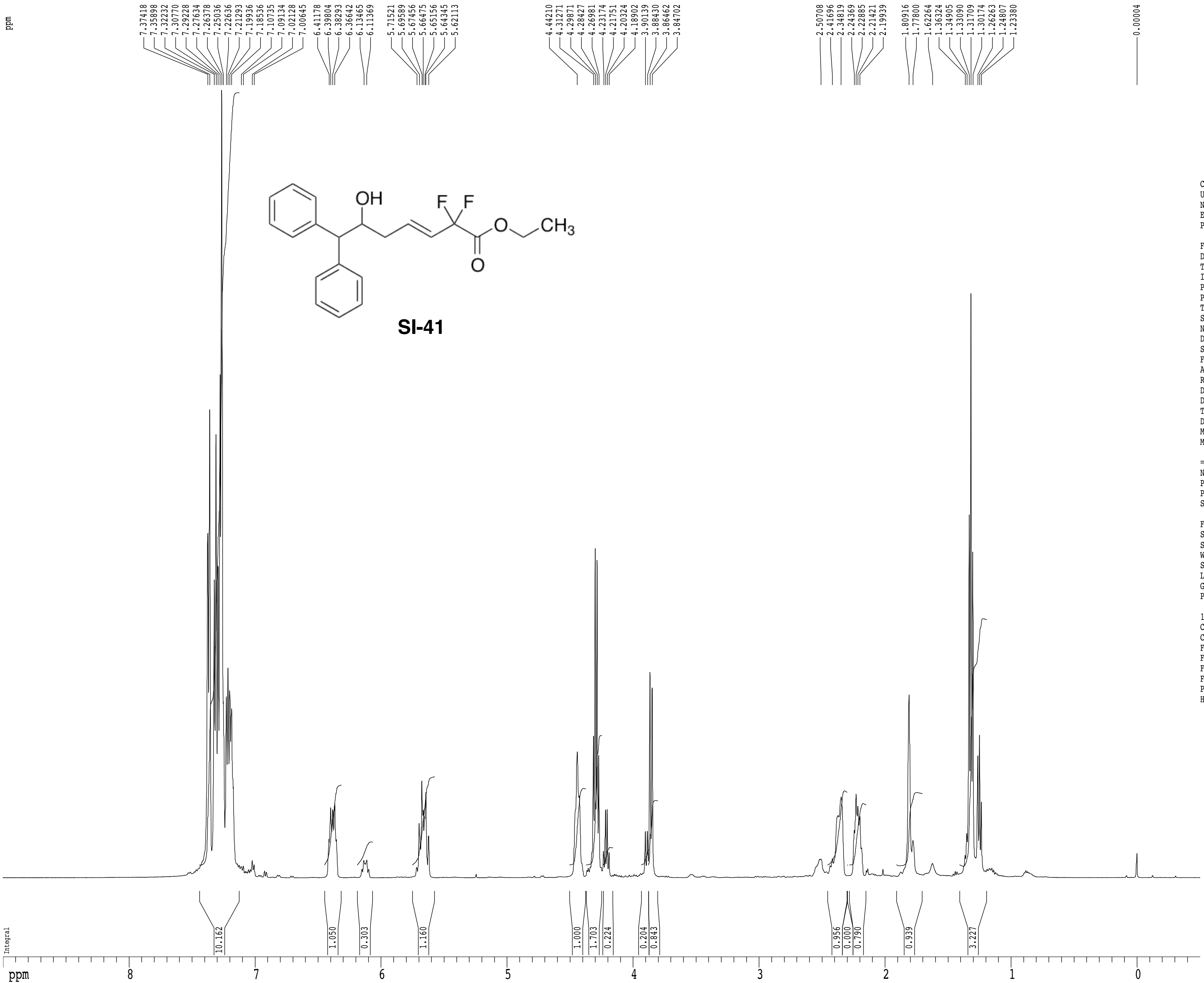


Current Data Parameters
USER linpc2
NAME pcl-1-268
EXPNO 2
PROCNO 1

F2 - Acquisition Parameters
Date_ 20210422
Time 13.30
INSTRUM cryo500
PROBHD 5 mm CPTCI 1H-
PULPROG zg30
TD 81728
SOLVENT CDCl3
NS 8
DS 2
SWH 8012.820 Hz
FIDRES 0.098043 Hz
AQ 5.0998774 sec
RG 2.8
DW 62.400 usec
DE 6.00 usec
TE 298.0 K
D1 0.10000000 sec
MCREST 0.00000000 sec
MCWRK 0.01500000 sec

======== CHANNEL f1 ========
NUC1 1H
P1 9.75 usec
PL1 1.60 dB
SFO1 500.2235015 MHz

F2 - Processing parameters
SI 65536
SF 500.2200490 MHz
WDW EM
SSB 0
LB 0.30 Hz
GB 0
PC 1.00

1D NMR plot parameters
CX 22.80 cm
CY 15.00 cm
F1P 9.000 ppm
F1 4501.98 Hz
F2P -0.500 ppm
F2 -250.11 Hz
PPMCM 0.41667 ppm/cm
HZCM 208.42503 Hz/cm

Z-restored spin-echo 13C spectrum with 1H decoupling

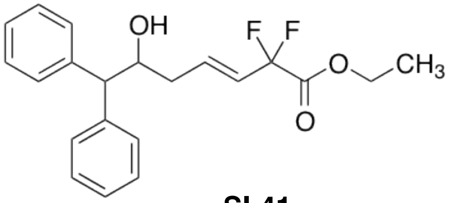

**SI-41**

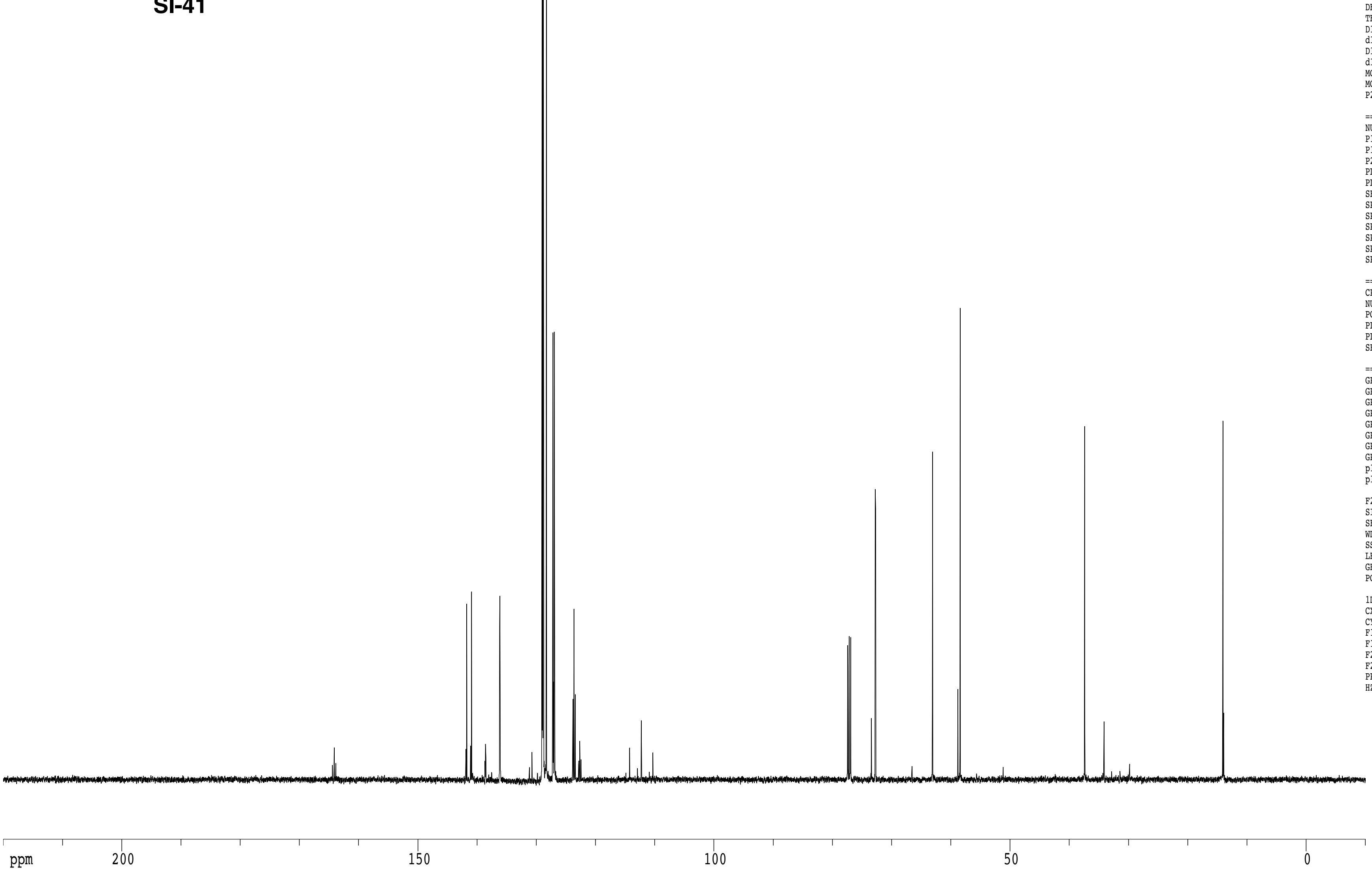


```
Current Data Parameters
USER              linpc2
NAME          pcl-1-268
EXPNO                  3
PROCNO                 1

F2 - Acquisition Parameters
Date_           20210422
Time               13.33
INSTRUM          cryo500
PROBHD   5 mm CPTCI 1H-
PULPROG  SpinEchopg30gp2.prd
TD                 65536
SOLVENT            CDCl3
NS                    96
DS                    16
SWH            30303.031 Hz
FIDRES          0.462388 Hz
AQ             1.0813940 sec
RG                  3251
DW                16.500 usec
DE                  6.00 usec
TE                 298.0 K
D1            0.25000000 sec
d11           0.03000000 sec
D16           0.00020000 sec
d17           0.00019600 sec
MCREST        0.00000000 sec
MCWRK         0.01500000 sec
P2                 37.70 usec

======== CHANNEL f1 ========
NUC1                 13C
P1                 18.85 usec
P12              2000.00 usec
P20               500.00 usec
PL0               120.00 dB
PL1                -1.00 dB
SFO1         125.7942548 MHz
SP2                 1.55 dB
SP4                 1.55 dB
SPNAM2       Crp60comp.4
SPNAM4    Crp60,0.5,20.1
SPOFF2              0.00 Hz
SPOFF4              0.00 Hz

======== CHANNEL f2 ========
CPDPRG2           waltz16
NUC2                   1H
PCPD2             100.00 usec
PL2                 1.60 dB
PL12               22.00 dB
SFO2         500.2225011 MHz

====== GRADIENT CHANNEL =====
GPNAM1          SINE.100
GPNAM2          SINE.100
GPX1                0.00 %
GPX2                0.00 %
GPY1                0.00 %
GPY2                0.00 %
GPZ1               30.00 %
GPZ2               50.00 %
p15               500.00 usec
p16              1000.00 usec

F2 - Processing parameters
SI                 65536
SF           125.7804173 MHz
WDW                   EM
SSB                    0
LB                  1.00 Hz
GB                     0
PC                  2.00

1D NMR plot parameters
CX                 22.80 cm
CY                 15.65 cm
F1P              220.000 ppm
F1              27671.69 Hz
F2P              -10.000 ppm
F2              -1257.80 Hz
PPMCM           10.08772 ppm/cm
HZCM          1268.83765 Hz/cm
```

# 1H spectrum

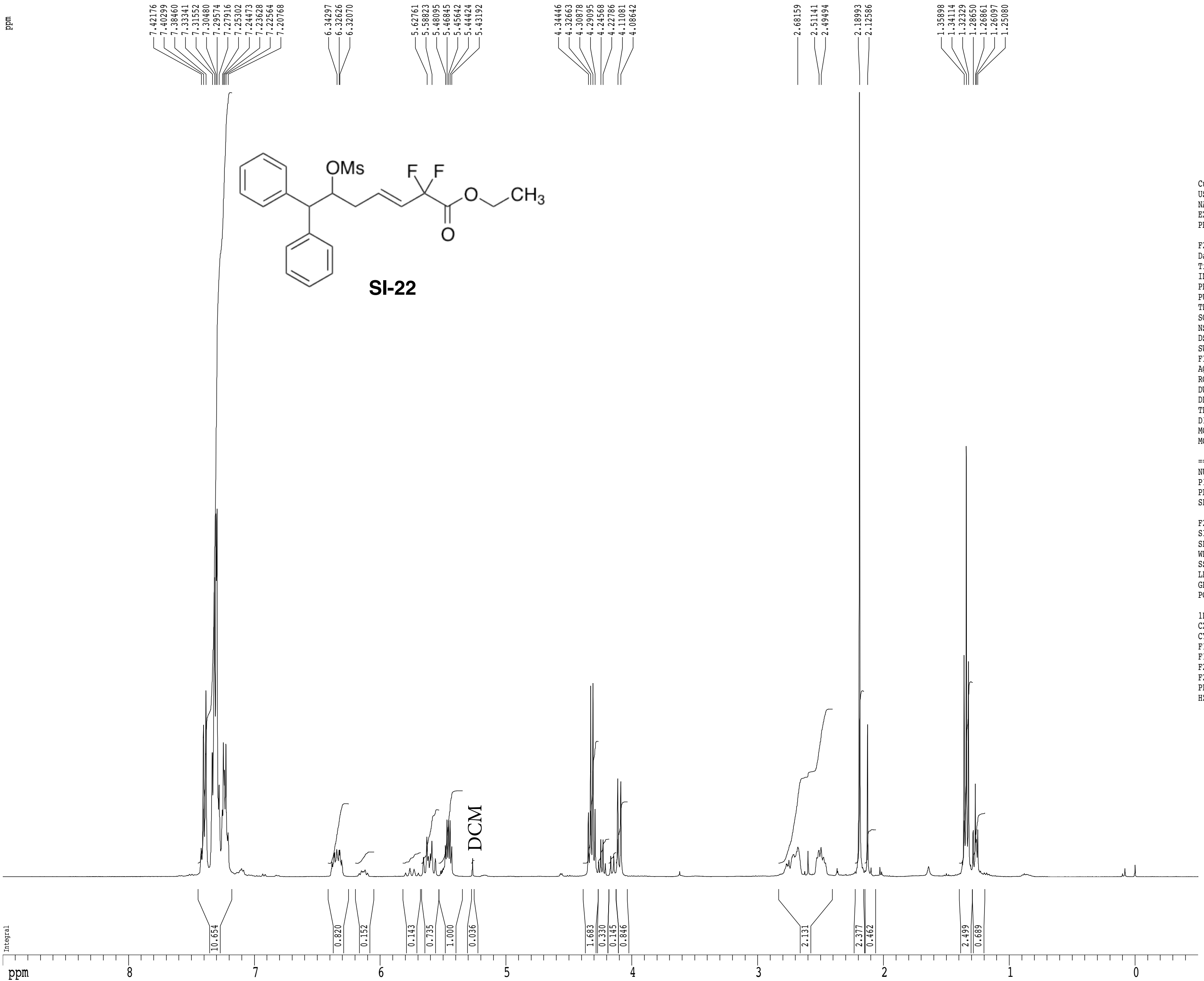


```
Current Data Parameters
USER            linpc2
NAME         pcl-2-027
EXPNO                2
PROCNO               1

F2 - Acquisition Parameters
Date_         20210426
Time              9.01
INSTRUM         drx400
PROBHD   5 mm QNP H/F/P
PULPROG           zg30
TD               65536
SOLVENT          CDCl3
NS                   8
DS                   2
SWH           6410.256 Hz
FIDRES        0.097813 Hz
AQ           5.1118579 sec
RG                  64
DW              78.000 usec
DE                4.50 usec
TE               298.0 K
D1          0.10000000 sec
MCREST      0.00000000 sec
MCWRK       0.01500000 sec

======== CHANNEL f1 ========
NUC1                1H
P1               12.00 usec
PL1              -1.60 dB
SFO1       400.1328009 MHz

F2 - Processing parameters
SI               65536
SF         400.1300272 MHz
WDW                 EM
SSB                  0
LB                0.30 Hz
GB                   0
PC                2.00

1D NMR plot parameters
CX               22.80 cm
CY               15.00 cm
F1P              9.000 ppm
F1             3601.17 Hz
F2P             -0.500 ppm
F2             -200.06 Hz
PPMCM          0.41667 ppm/cm
HZCM          166.72086 Hz/cm
```

13C spectrum with 1H decoupling

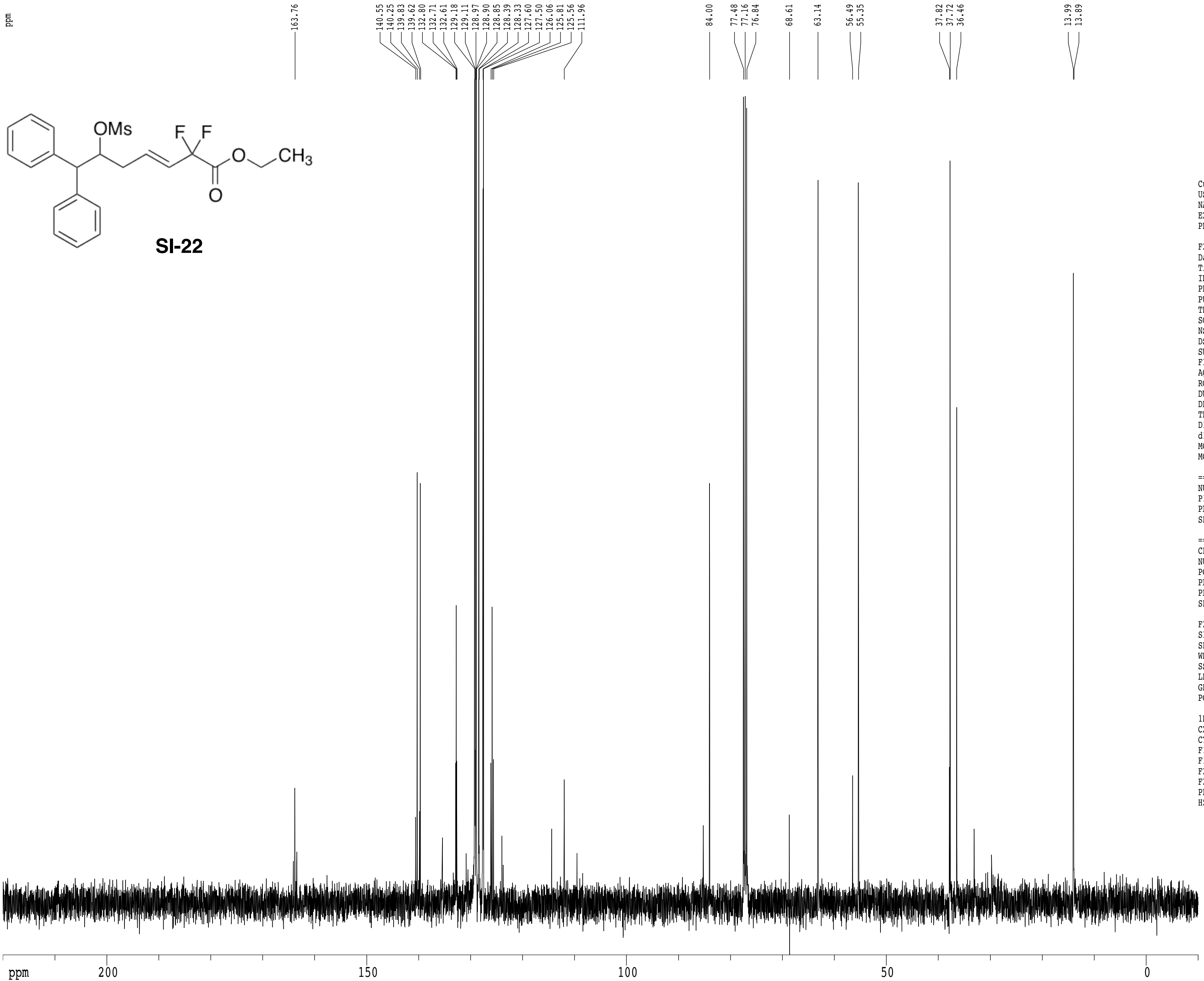

SI-22



```
Current Data Parameters
USER              linpc2
NAME           pcl-2-027
EXPNO                  3
PROCNO                 1

F2 - Acquisition Parameters
Date_           20210426
Time                9.04
INSTRUM           drx400
PROBHD   5 mm QNP H/F/P
PULPROG           zgdc30
TD                 65536
SOLVENT            CDCl3
NS                   112
DS                     4
SWH           24154.590 Hz
FIDRES         0.368570 Hz
AQ            1.3566452 sec
RG                9195.2
DW               20.700 usec
DE                20.39 usec
TE                298.1 K
D1           0.10000000 sec
d11          0.03000000 sec
MCREST       0.00000000 sec
MCWRK        0.01500000 sec

======== CHANNEL f1 ========
NUC1                 13C
P1                  8.30 usec
PL1                -3.00 dB
SFO1         100.6237964 MHz

======== CHANNEL f2 ========
CPDPRG2          waltz16
NUC2                  1H
PCPD2              90.00 usec
PL2                -1.60 dB
PL12               16.50 dB
SFO2         400.1328009 MHz

F2 - Processing parameters
SI                 65536
SF           100.6127669 MHz
WDW                   EM
SSB                    0
LB                  1.00 Hz
GB                     0
PC                  1.00

1D NMR plot parameters
CX                 22.80 cm
CY                 35.00 cm
F1P              220.000 ppm
F1              22134.81 Hz
F2P              -10.000 ppm
F2              -1006.13 Hz
PPMCM           10.08772 ppm/cm
HZCM          1014.95343 Hz/cm
```

19F spectrum

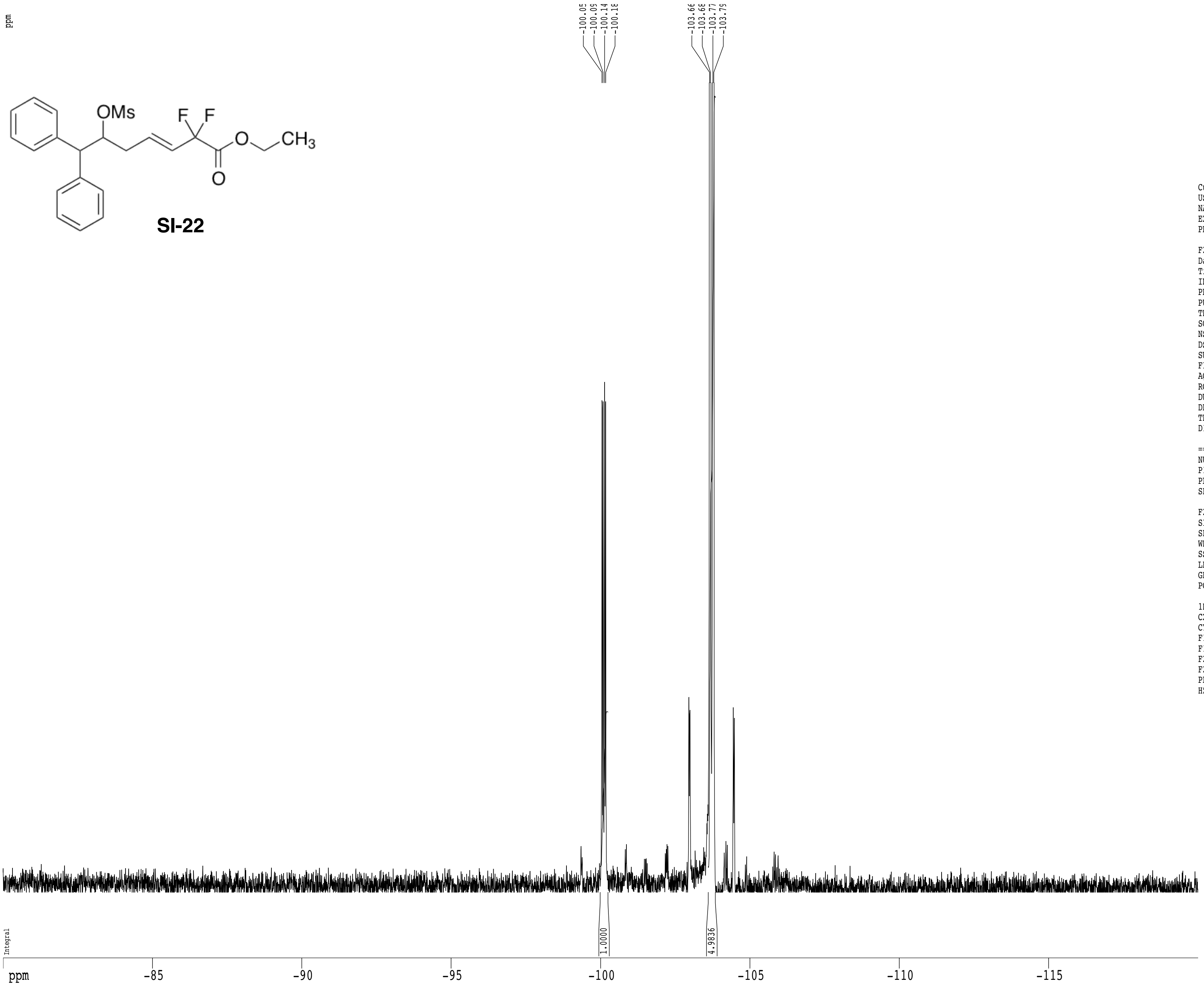


**SI-22**

```
Current Data Parameters
USER              linpc2
NAME           pcl-2-027
EXPNO                  4
PROCNO                 1

F2 - Acquisition Parameters
Date_           20210426
Time                9.07
INSTRUM           drx400
PROBHD   5 mm QNP H/F/P
PULPROG        zgflqn30
TD                 65536
SOLVENT            CDCl3
NS                    32
DS                     2
SWH            75187.969 Hz
FIDRES          1.147277 Hz
AQ             0.4358644 sec
RG                5792.6
DW                 6.650 usec
DE                  9.46 usec
TE                 298.0 K
D1            2.00000000 sec

======== CHANNEL f1 ========
NUC1                 19F
P1                 11.75 usec
PL1                -6.00 dB
SFO1         376.4646491 MHz

F2 - Processing parameters
SI                 65536
SF           376.4984640 MHz
WDW                   EM
SSB                    0
LB                  1.00 Hz
GB                     0
PC                  1.00

1D NMR plot parameters
CX                 22.80 cm
CY                 45.00 cm
F1P              -80.000 ppm
F1             -30119.88 Hz
F2P             -120.000 ppm
F2             -45179.82 Hz
PPMCM            1.75439 ppm/cm
HZCM           660.52368 Hz/cm
```

1H spectrum

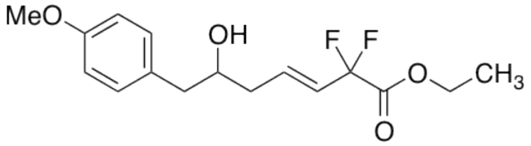


**SI-42**

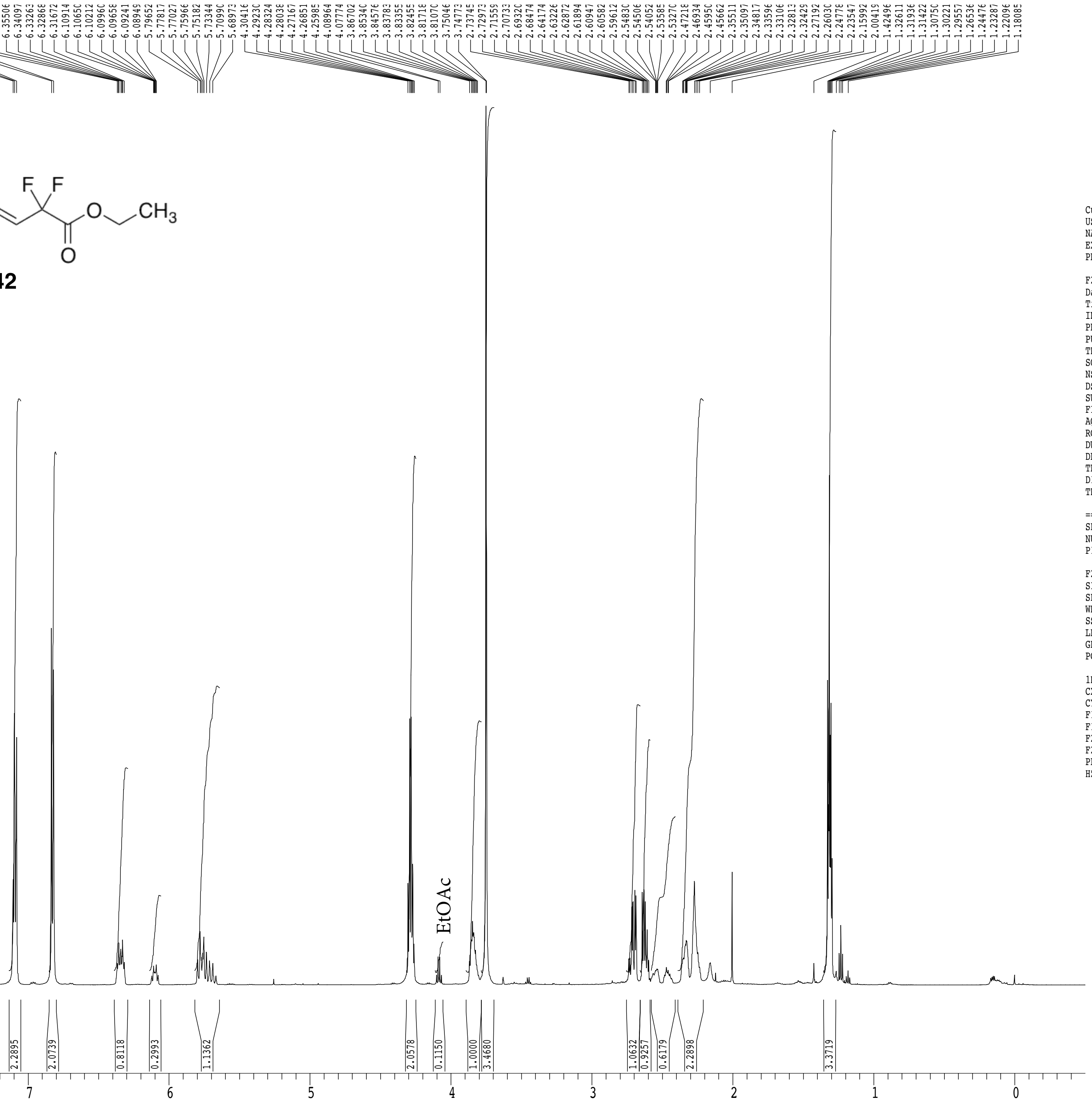


```
Current Data Parameters
USER              linpc2
NAME           pcl-2-148
EXPNO                  3
PROCNO                 1

F2 - Acquisition Parameters
Date_           20210812
Time               15.40
INSTRUM            av600
PROBHD   5 mm CPBBO BB-
PULPROG             zg30
TD                 98074
SOLVENT           CDCl3T
NS                     8
DS                     2
SWH             9615.385 Hz
FIDRES          0.098042 Hz
AQ             5.0998979 sec
RG                    10
DW                52.000 usec
DE                 14.23 usec
TE                 298.1 K
D1            0.10000000 sec
TD0                    1

======== CHANNEL f1 ========
SFO1         600.1342009 MHz
NUC1                  1H
P1                  9.50 usec

F2 - Processing parameters
SI                 65536
SF           600.1300282 MHz
WDW                   no
SSB                    0
LB                  0.00 Hz
GB                     0
PC                  1.00

1D NMR plot parameters
CX                 22.80 cm
CY                 15.00 cm
F1P                9.000 ppm
F1               5401.17 Hz
F2P               -0.500 ppm
F2               -300.06 Hz
PPMCM            0.41667 ppm/cm
HZCM            250.05418 Hz/cm
```

13C spectrum

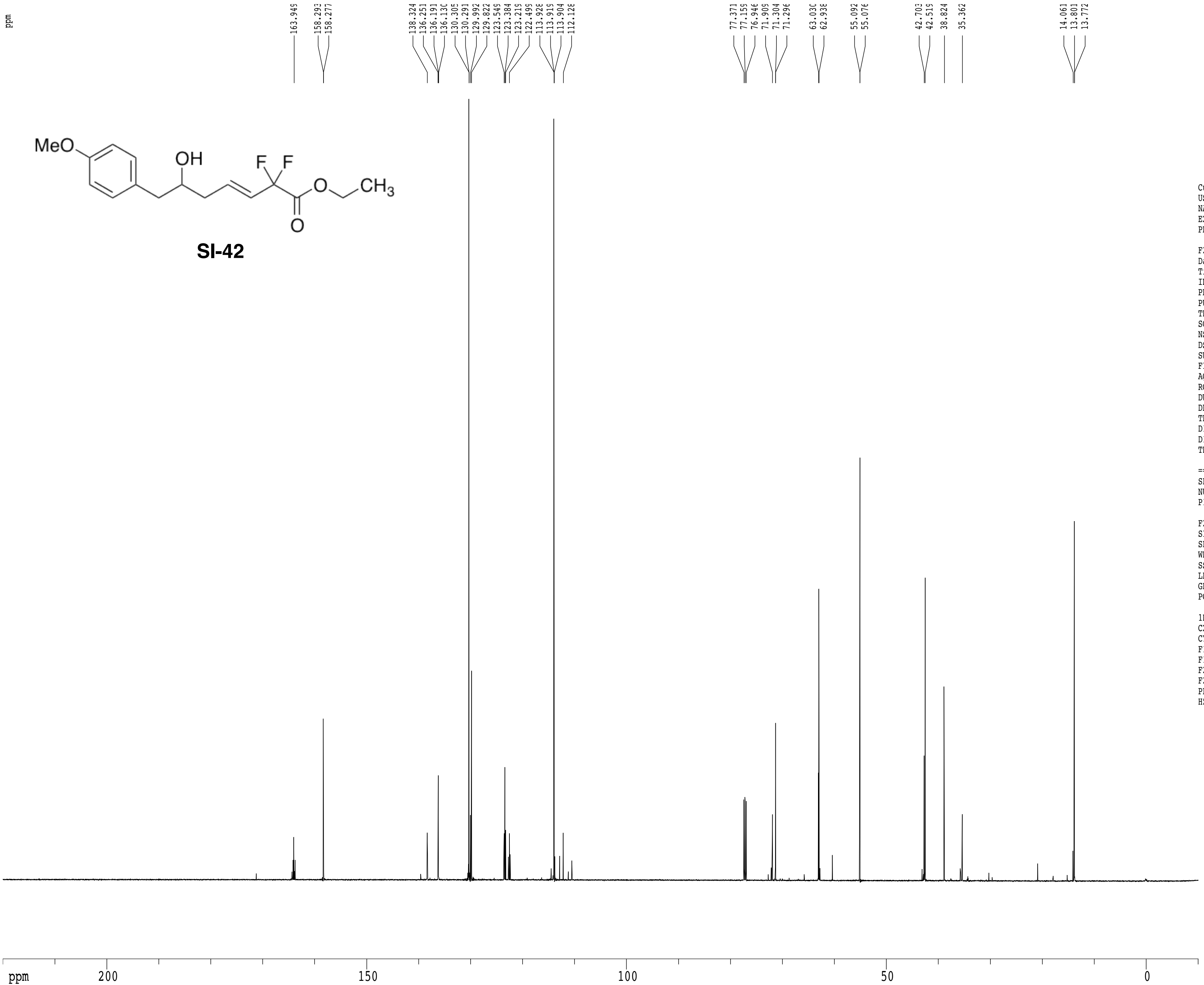


**SI-42**

Current Data Parameters
USER linpc2
NAME pcl-2-148
EXPNO 5
PROCNO 1

F2 - Acquisition Parameters
Date_ 20210812
Time 15.50
INSTRUM av600
PROBHD 5 mm CPBBO BB-
PULPROG zgdc30
TD 65536
SOLVENT CDCl3T
NS 111
DS 4
SWH 36231.883 Hz
FIDRES 0.552855 Hz
AQ 0.9044468 sec
RG 2050
DW 13.800 usec
DE 19.63 usec
TE 298.0 K
D1 0.40000001 sec
D11 0.03000000 sec
TD0 1

======== CHANNEL f1 ========
SFO1 150.9194080 MHz
NUC1 13C
P1 10.10 usec

F2 - Processing parameters
SI 65536
SF 150.9028229 MHz
WDW no
SSB 0
LB 0.00 Hz
GB 0
PC 1.00

1D NMR plot parameters
CX 22.80 cm
CY 15.00 cm
F1P 220.000 ppm
F1 33198.62 Hz
F2P -10.000 ppm
F2 -1509.03 Hz
PPMCM 10.08772 ppm/cm
HZCM 1522.26526 Hz/cm

19F spectrum

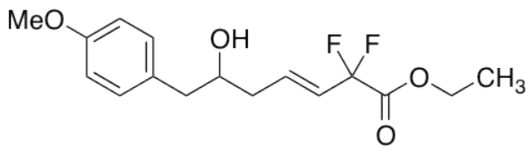

SI-42

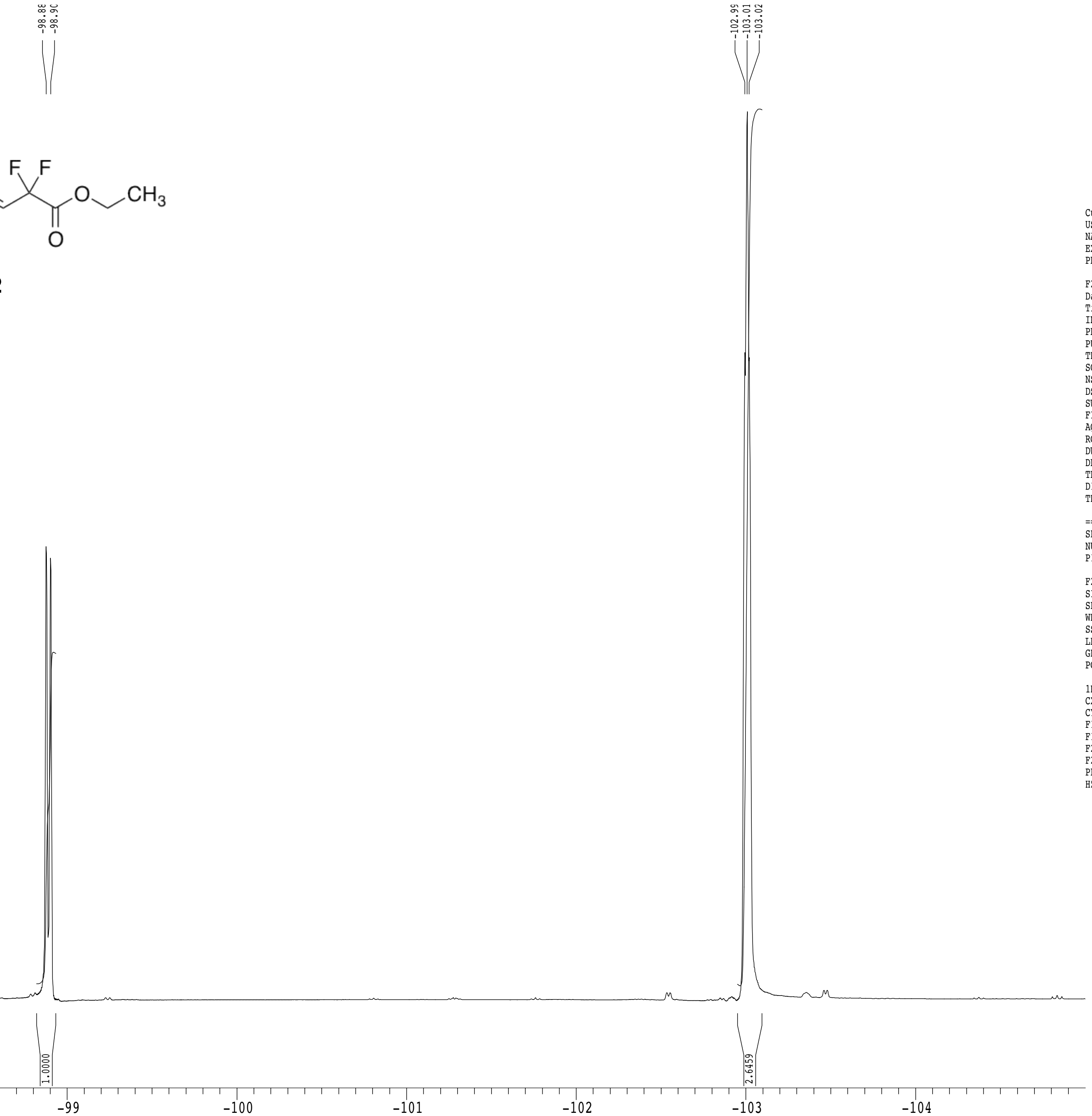


```
Current Data Parameters
USER              linpc2
NAME            pcl-2-148
EXPNO                   4
PROCNO                  1

F2 - Acquisition Parameters
Date_            20210812
Time                15.46
INSTRUM             av600
PROBHD   5 mm CPBBO BB-
PULPROG         zgflqn30
TD                 131072
SOLVENT            CDCl3T
NS                     16
DS                      2
SWH            178571.422 Hz
FIDRES           1.362392 Hz
AQ              0.3670516 sec
RG                    228
DW                  2.800 usec
DE                  18.00 usec
TE                  298.0 K
D1             3.00000000 sec
TD0                     1

======== CHANNEL f1 ========
SFO1         564.6299196 MHz
NUC1                  19F
P1                  18.25 usec

F2 - Processing parameters
SI                 131072
SF            564.6864152 MHz
WDW                    no
SSB                     0
LB                   0.00 Hz
GB                      0
PC                   1.00

1D NMR plot parameters
CX                  22.80 cm
CY                  15.00 cm
F1P               -97.000 ppm
F1              -54774.58 Hz
F2P              -105.000 ppm
F2              -59292.07 Hz
PPMCM             0.35088 ppm/cm
HZCM            198.13559 Hz/cm
```

1H spectrum

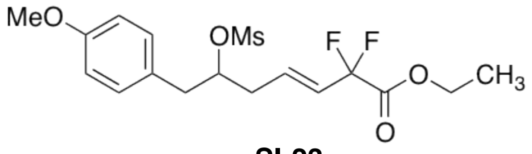

**SI-23**

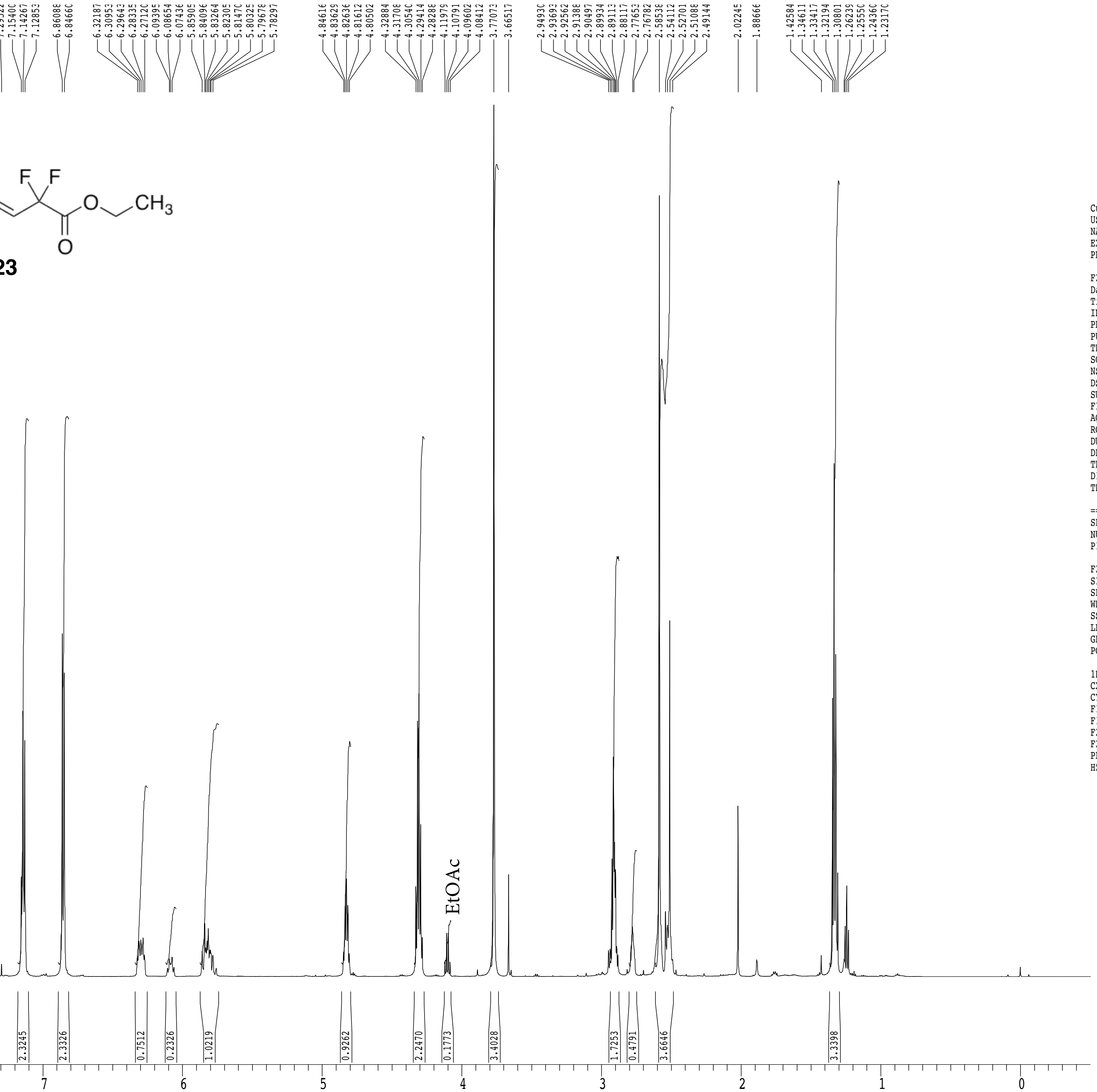


Current Data Parameters
USER linpc2
NAME pcl-2-151
EXPNO 1
PROCNO 1

F2 - Acquisition Parameters
Date_ 20210813
Time 13.47
INSTRUM av600
PROBHD 5 mm CPBBO BB-
PULPROG zg30
TD 98074
SOLVENT CDCl3T
NS 8
DS 2
SWH 9615.385 Hz
FIDRES 0.098042 Hz
AQ 5.0998979 sec
RG 8
DW 52.000 usec
DE 14.23 usec
TE 298.0 K
D1 0.10000000 sec
TD0 1

======== CHANNEL f1 ========
SFO1 600.1342009 MHz
NUC1 1H
P1 9.50 usec

F2 - Processing parameters
SI 65536
SF 600.1300128 MHz
WDW no
SSB 0
LB 0.00 Hz
GB 0
PC 1.00

1D NMR plot parameters
CX 22.80 cm
CY 15.00 cm
F1P 9.000 ppm
F1 5401.17 Hz
F2P -0.500 ppm
F2 -300.06 Hz
PPMCM 0.41667 ppm/cm
HZCM 250.05418 Hz/cm

13C spectrum

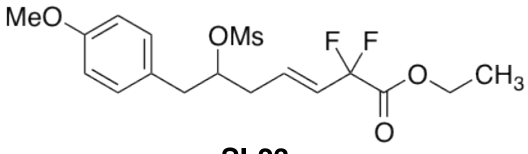


**SI-23**

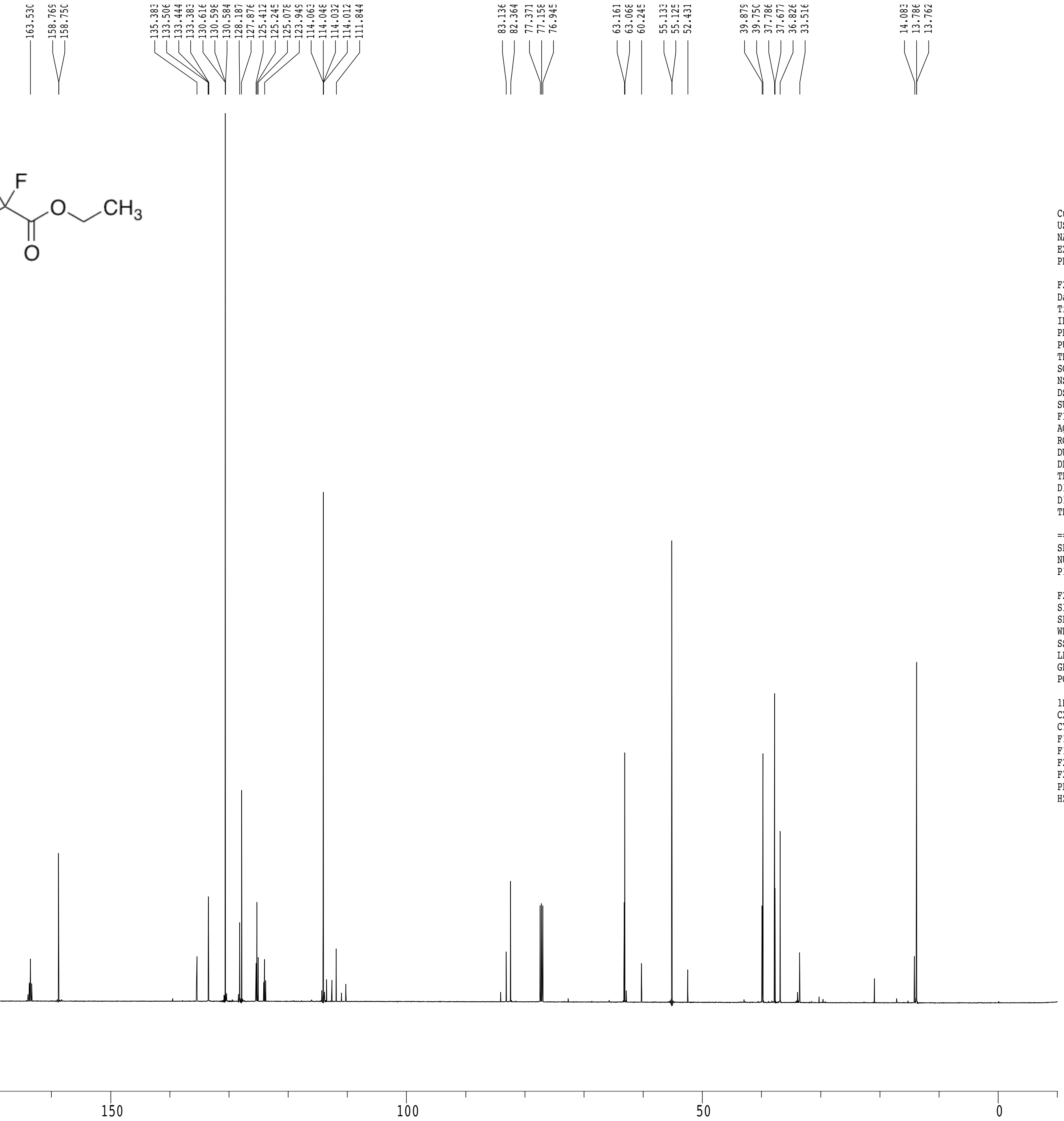


Current Data Parameters
USER linpc2
NAME pcl-2-151
EXPNO 3
PROCNO 1

F2 - Acquisition Parameters
Date_ 20210813
Time 13.59
INSTRUM av600
PROBHD 5 mm CPBBO BB-
PULPROG zgdc30
TD 65536
SOLVENT CDCl3T
NS 518
DS 4
SWH 36231.883 Hz
FIDRES 0.552855 Hz
AQ 0.9044468 sec
RG 2050
DW 13.800 usec
DE 19.63 usec
TE 298.0 K
D1 0.40000001 sec
D11 0.03000000 sec
TD0 1

======== CHANNEL f1 ========
SFO1 150.9194080 MHz
NUC1 13C
P1 10.10 usec

F2 - Processing parameters
SI 65536
SF 150.9028235 MHz
WDW no
SSB 0
LB 0.00 Hz
GB 0
PC 1.00

1D NMR plot parameters
CX 22.80 cm
CY 15.00 cm
F1P 220.000 ppm
F1 33198.62 Hz
F2P -10.000 ppm
F2 -1509.03 Hz
PPMCM 10.08772 ppm/cm
HZCM 1522.26526 Hz/cm

19F spectrum

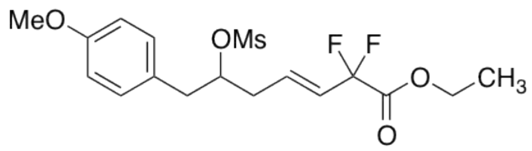


**SI-23**

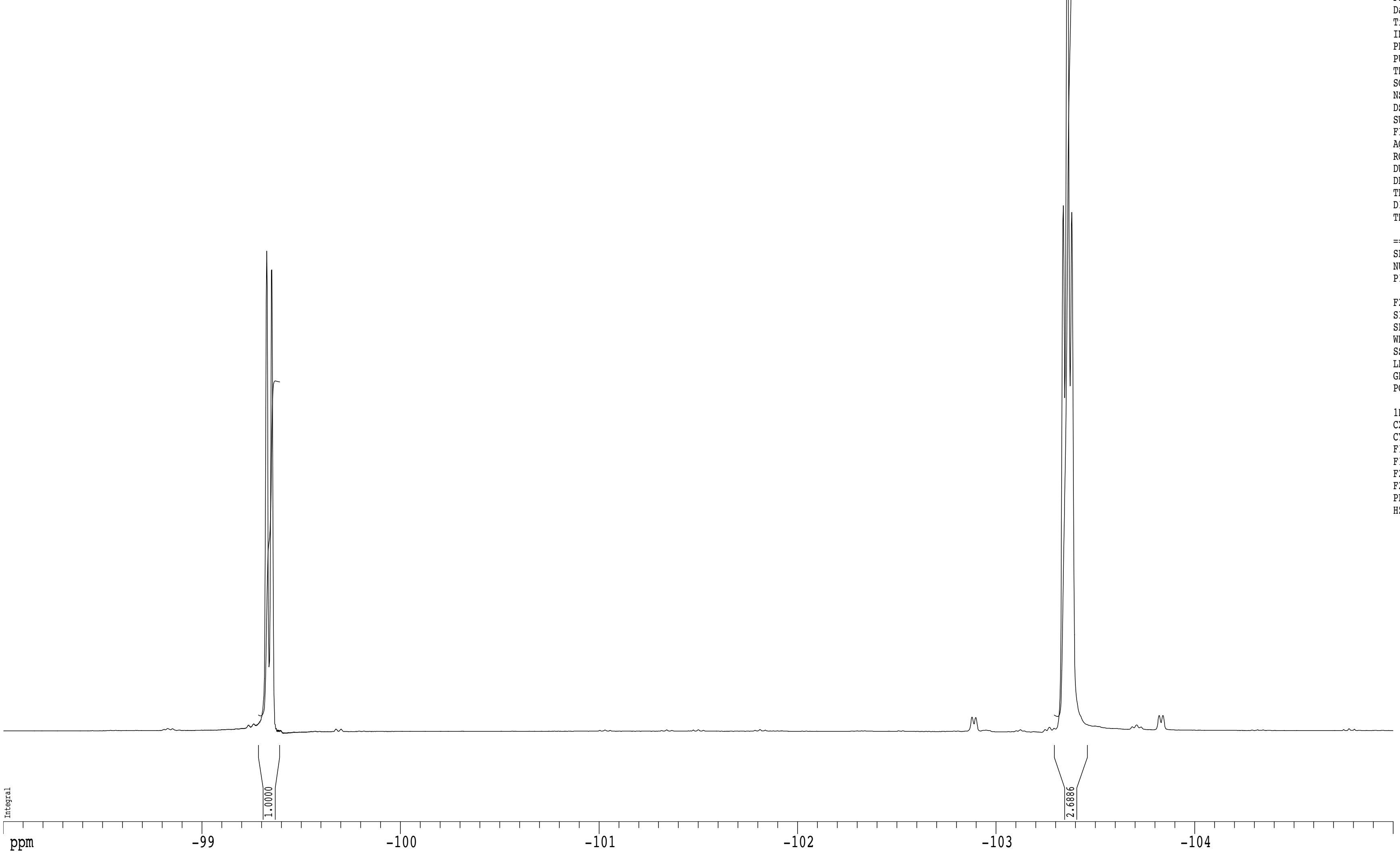


```
Current Data Parameters
USER              linpc2
NAME           pcl-2-151
EXPNO                  2
PROCNO                 1

F2 - Acquisition Parameters
Date_           20210813
Time               13.54
INSTRUM            av600
PROBHD   5 mm CPBBO BB-
PULPROG        zgflqn30
TD                131072
SOLVENT           CDCl3T
NS                    16
DS                     2
SWH          178571.422 Hz
FIDRES         1.362392 Hz
AQ            0.3670516 sec
RG                   287
DW                 2.800 usec
DE                 18.00 usec
TE                 298.0 K
D1            3.00000000 sec
TD0                    1

======== CHANNEL f1 ========
SFO1        564.6299196 MHz
NUC1                 19F
P1                 18.25 usec

F2 - Processing parameters
SI                131072
SF           564.6864005 MHz
WDW                   no
SSB                    0
LB                  0.00 Hz
GB                     0
PC                  1.00

1D NMR plot parameters
CX                 22.80 cm
CY                 15.00 cm
F1P              -98.000 ppm
F1             -55339.27 Hz
F2P             -105.000 ppm
F2             -59292.07 Hz
PPMCM            0.30702 ppm/cm
HZCM           173.36864 Hz/cm
```

1H spectrum

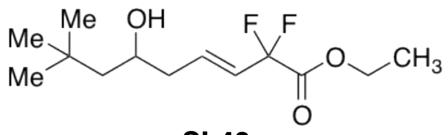

**SI-43**

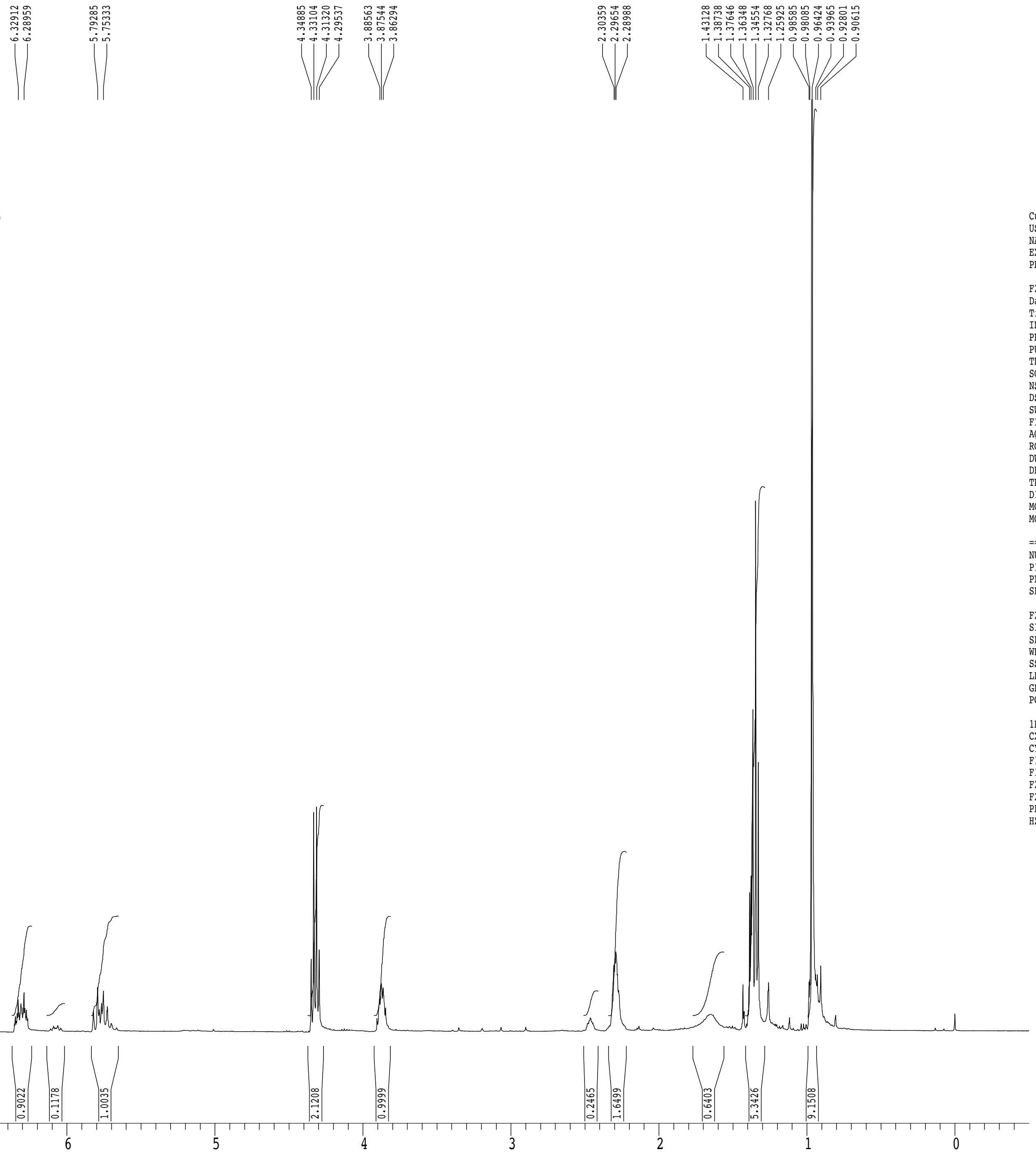


Current Data Parameters
USER linpc2
NAME pcl-2-144
EXPNO 1
PROCNO 1

F2 - Acquisition Parameters
Date_ 20210810
Time 14.07
INSTRUM drx400
PROBHD 5 mm QNP H/F/P
PULPROG zg30
TD 65536
SOLVENT CDCl3
NS 8
DS 2
SWH 6410.256 Hz
FIDRES 0.097813 Hz
AQ 5.1118579 sec
RG 57
DW 78.000 usec
DE 4.50 usec
TE 298.0 K
D1 0.10000000 sec
MCREST 0.00000000 sec
MCWRK 0.01500000 sec

======== CHANNEL f1 ========
NUC1 1H
P1 12.00 usec
PL1 -1.60 dB
SFO1 400.1328009 MHz

F2 - Processing parameters
SI 65536
SF 400.1300133 MHz
WDW EM
SSB 0
LB 0.30 Hz
GB 0
PC 2.00

1D NMR plot parameters
CX 22.80 cm
CY 45.00 cm
F1P 9.000 ppm
F1 3601.17 Hz
F2P -0.500 ppm
F2 -200.06 Hz
PPMCM 0.41667 ppm/cm
HZCM 166.72084 Hz/cm

1H spectrum

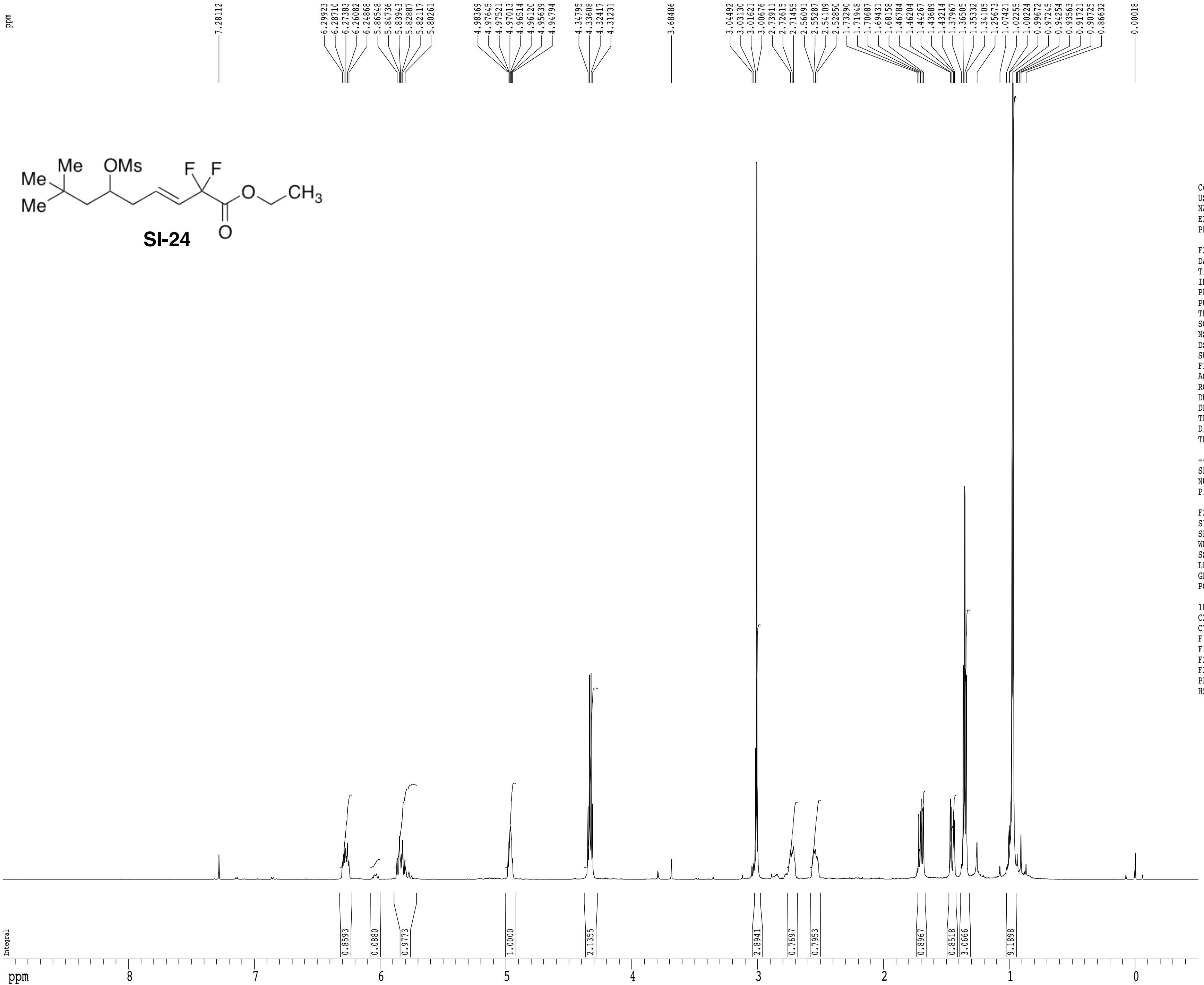


Current Data Parameters
USER linpc2
NAME pcl-2-147
EXPNO 5
PROCNO 1

F2 - Acquisition Parameters
Date_ 20210812
Time 15.58
INSTRUM av600
PROBHD 5 mm CPBBO BB-
PULPROG zg30
TD 98074
SOLVENT CDCl3T
NS 8
DS 2
SWH 9615.385 Hz
FIDRES 0.098042 Hz
AQ 5.0998979 sec
RG 10
DW 52.000 usec
DE 14.23 usec
TE 298.0 K
D1 0.10000000 sec
TD0 1

======== CHANNEL f1 ========
SFO1 600.1342009 MHz
NUC1 1H
P1 9.50 usec

F2 - Processing parameters
SI 65536
SF 600.1300221 MHz
WDW no
SSB 0
LB 0.00 Hz
GB 0
PC 1.00

1D NMR plot parameters
CX 22.80 cm
CY 30.00 cm
F1P 9.000 ppm
F1 5401.17 Hz
F2P -0.500 ppm
F2 -300.06 Hz
PPMCM 0.41667 ppm/cm
HZCM 250.05418 Hz/cm

13C spectrum

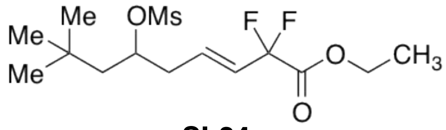


**SI-24**

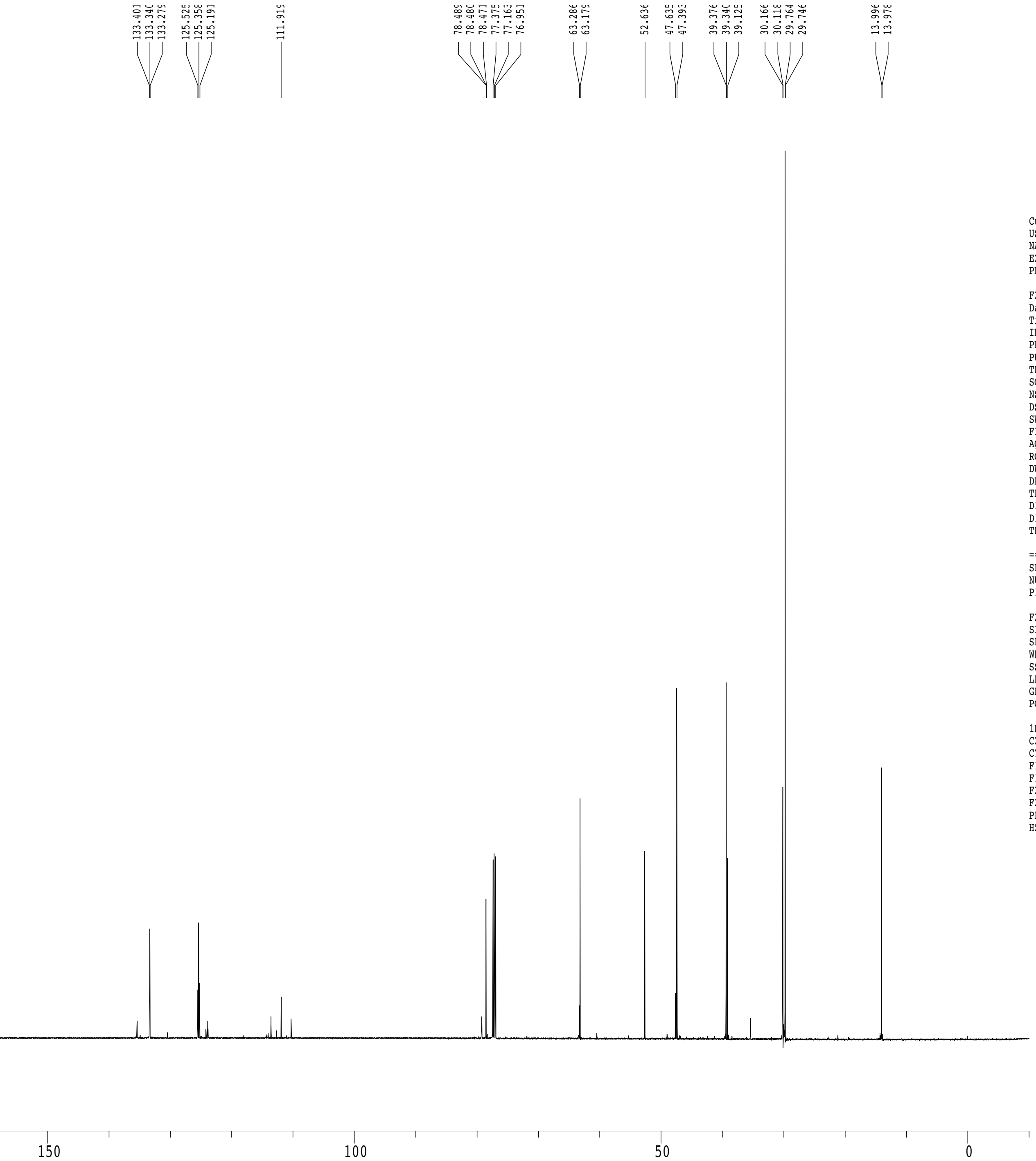


```
Current Data Parameters
USER            linpc2
NAME            pcl-2-147
EXPNO           3
PROCNO          1

F2 - Acquisition Parameters
Date_           20210811
Time            13.19
INSTRUM         av600
PROBHD   5 mm CPBBO BB-
PULPROG         zgdc30
TD              65536
SOLVENT         CDCl3T
NS              350
DS              4
SWH             36231.883 Hz
FIDRES          0.552855 Hz
AQ              0.9044468 sec
RG              2050
DW              13.800 usec
DE              19.63 usec
TE              298.0 K
D1              0.40000001 sec
D11             0.03000000 sec
TD0             1

======== CHANNEL f1 ========
SFO1            150.9194080 MHz
NUC1            13C
P1              10.10 usec

F2 - Processing parameters
SI              65536
SF              150.9028014 MHz
WDW             no
SSB             0
LB              0.00 Hz
GB              0
PC              1.00

1D NMR plot parameters
CX              22.80 cm
CY              15.00 cm
F1P             220.000 ppm
F1              33198.62 Hz
F2P             -10.000 ppm
F2              -1509.03 Hz
PPMCM           10.08772 ppm/cm
HZCM            1522.26514 Hz/cm
```

19F spectrum

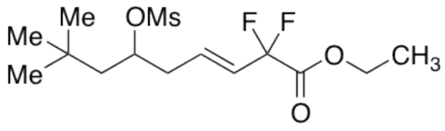

SI-24

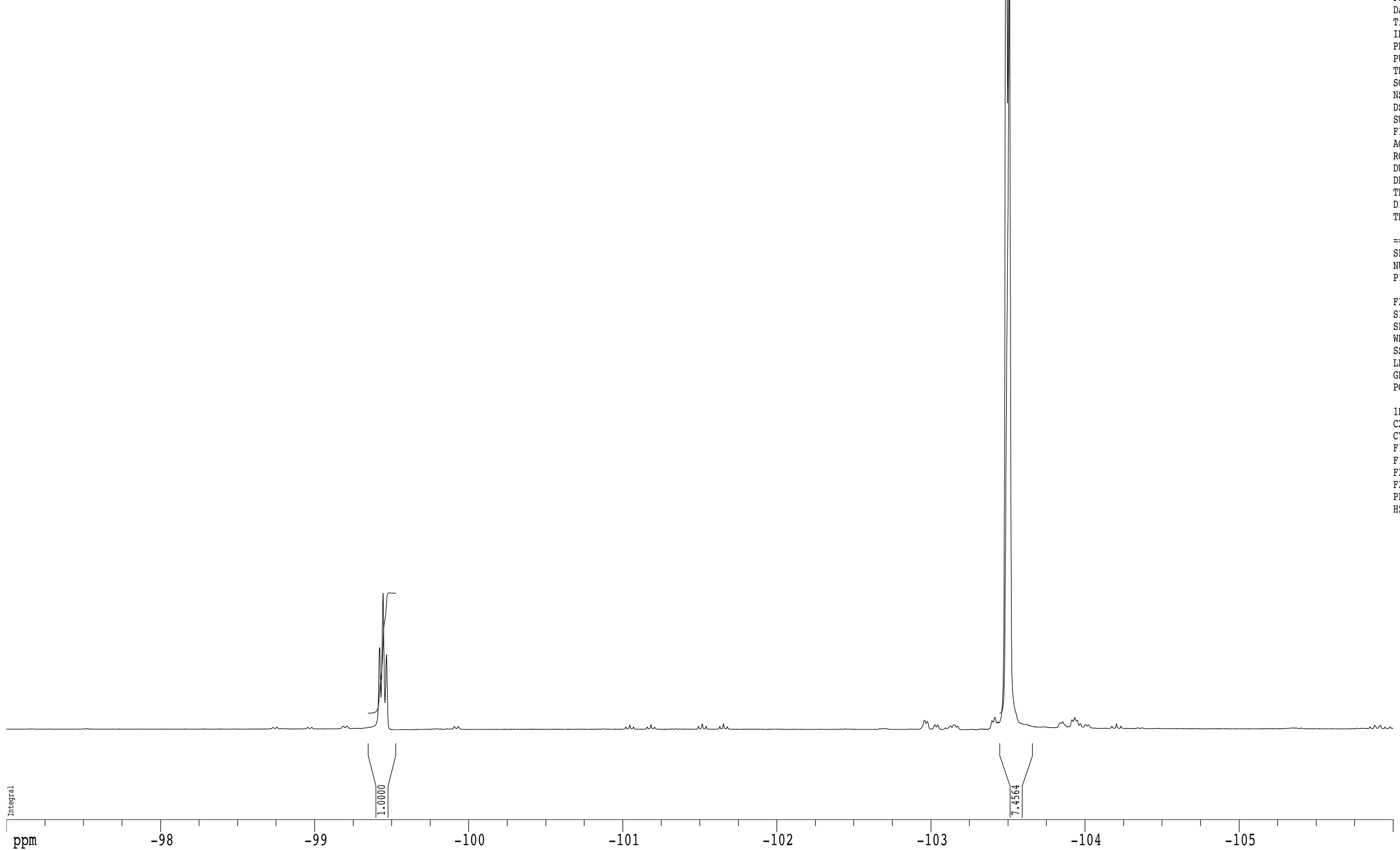


```
Current Data Parameters
USER            linpc2
NAME         pcl-2-147
EXPNO                2
PROCNO               1

F2 - Acquisition Parameters
Date_         20210811
Time             13.11
INSTRUM          av600
PROBHD   5 mm CPBBO BB-
PULPROG       zgflqn30
TD              131072
SOLVENT         CDCl3T
NS                  16
DS                   2
SWH         178571.422 Hz
FIDRES        1.362392 Hz
AQ           0.3670516 sec
RG                 362
DW               2.800 usec
DE               18.00 usec
TE               298.0 K
D1          3.00000000 sec
TD0                  1

======== CHANNEL f1 ========
SFO1       564.6299196 MHz
NUC1               19F
P1               18.25 usec

F2 - Processing parameters
SI              131072
SF         564.6863858 MHz
WDW                 no
SSB                  0
LB                0.00 Hz
GB                   0
PC                1.00

1D NMR plot parameters
CX               22.80 cm
CY               15.00 cm
F1P            -97.000 ppm
F1           -54774.58 Hz
F2P           -106.000 ppm
F2           -59856.76 Hz
PPMCM          0.39474 ppm/cm
HZCM         222.90253 Hz/cm
```

1H spectrum

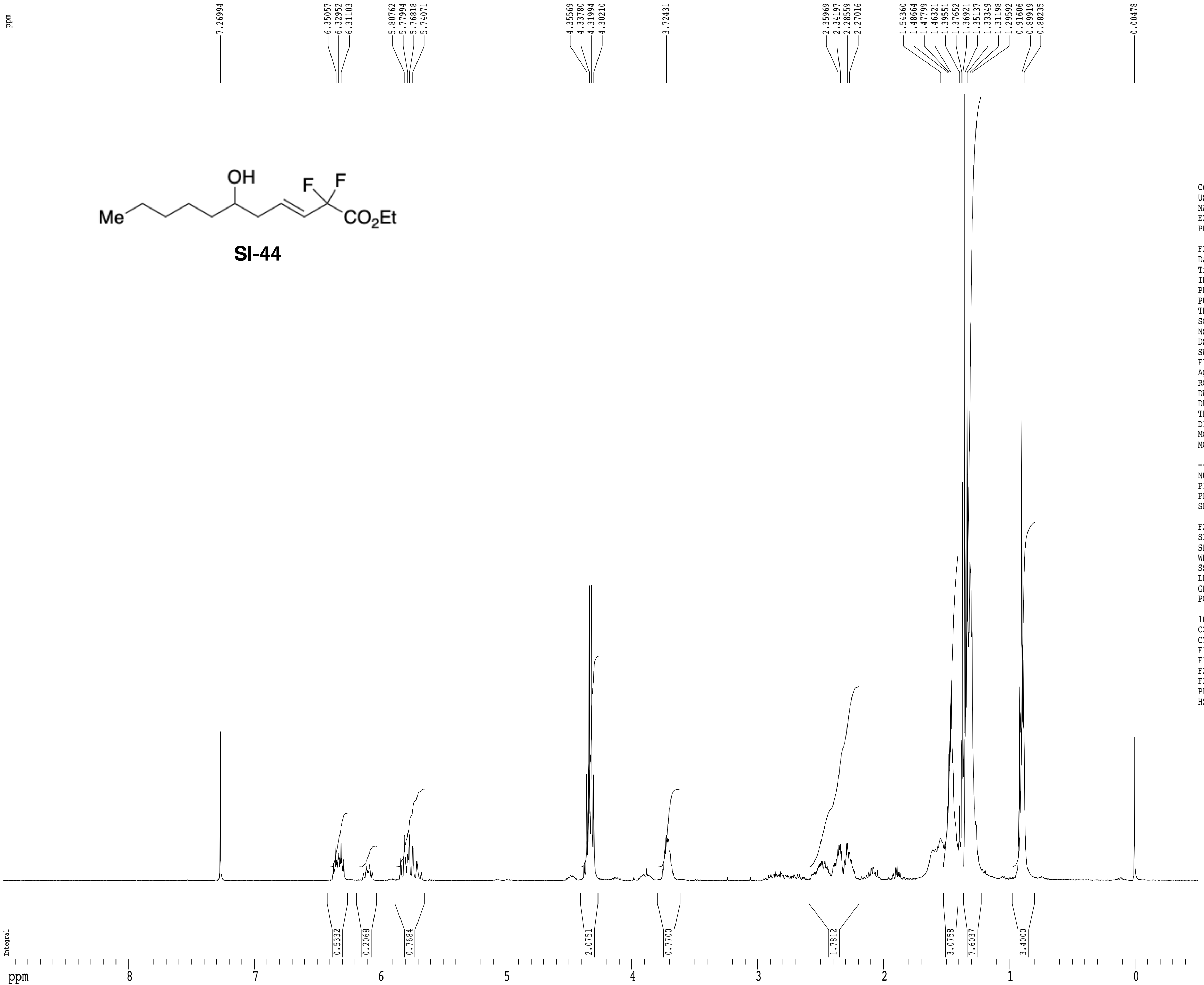


**SI-44**

```
Current Data Parameters
USER          mcginnit
NAME     tmm-3-198-char
EXPNO                 1
PROCNO                1

F2 - Acquisition Parameters
Date_          20210906
Time              13.16
INSTRUM          drx400
PROBHD   5 mm Multinucl
PULPROG            zg30
TD                65536
SOLVENT           CDCl3
NS                    8
DS                    2
SWH            6410.256 Hz
FIDRES         0.097813 Hz
AQ            5.1118579 sec
RG                  256
DW               78.000 usec
DE                 4.50 usec
TE                298.1 K
D1           0.10000000 sec
MCREST       0.00000000 sec
MCWRK        0.01500000 sec

======== CHANNEL f1 ========
NUC1                 1H
P1                12.00 usec
PL1               -1.10 dB
SFO1        400.1328009 MHz

F2 - Processing parameters
SI                65536
SF          400.1300175 MHz
WDW                  EM
SSB                   0
LB                 0.30 Hz
GB                    0
PC                 2.00

1D NMR plot parameters
CX                22.80 cm
CY                15.00 cm
F1P               9.000 ppm
F1              3601.17 Hz
F2P              -0.500 ppm
F2              -200.06 Hz
PPMCM           0.41667 ppm/cm
HZCM           166.72084 Hz/cm
```

1H spectrum

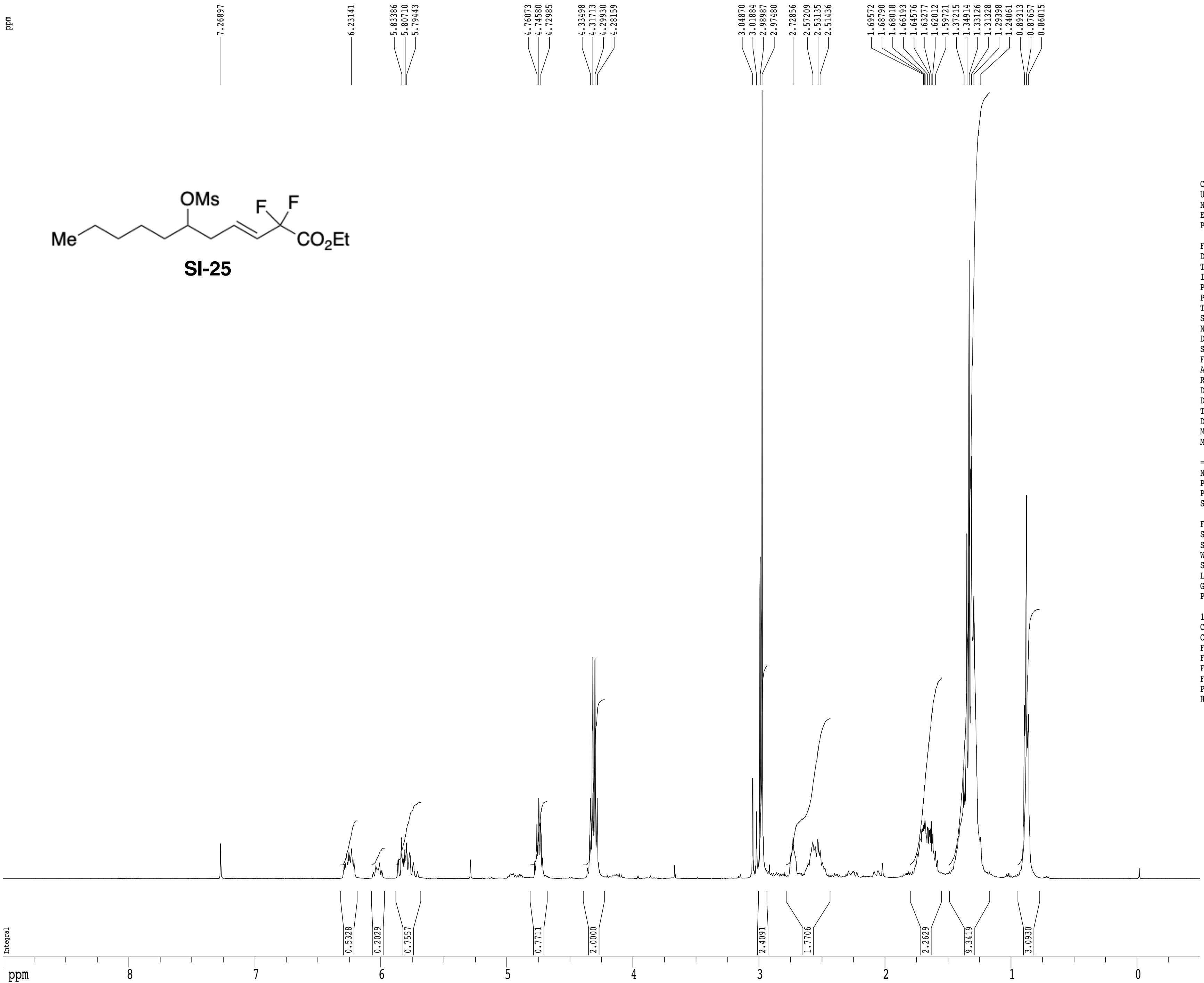


Current Data Parameters
USER mcginnit
NAME tmm-3-199
EXPNO 1
PROCNO 1

F2 - Acquisition Parameters
Date_ 20210803
Time 13.50
INSTRUM drx400
PROBHD 5 mm QNP H/F/P
PULPROG zg30
TD 65536
SOLVENT CDCl3
NS 8
DS 2
SWH 6410.256 Hz
FIDRES 0.097813 Hz
AQ 5.1118579 sec
RG 57
DW 78.000 usec
DE 4.50 usec
TE 298.0 K
D1 0.10000000 sec
MCREST 0.00000000 sec
MCWRK 0.01500000 sec

======== CHANNEL f1 ========
NUC1 1H
P1 12.00 usec
PL1 -1.60 dB
SFO1 400.1328009 MHz

F2 - Processing parameters
SI 65536
SF 400.1300175 MHz
WDW EM
SSB 0
LB 0.30 Hz
GB 0
PC 2.00

1D NMR plot parameters
CX 22.80 cm
CY 15.00 cm
F1P 9.000 ppm
F1 3601.17 Hz
F2P -0.500 ppm
F2 -200.06 Hz
PPMCM 0.41667 ppm/cm
HZCM 166.72084 Hz/cm

Z-restored spin-echo 13C spectrum with 1H decoupling

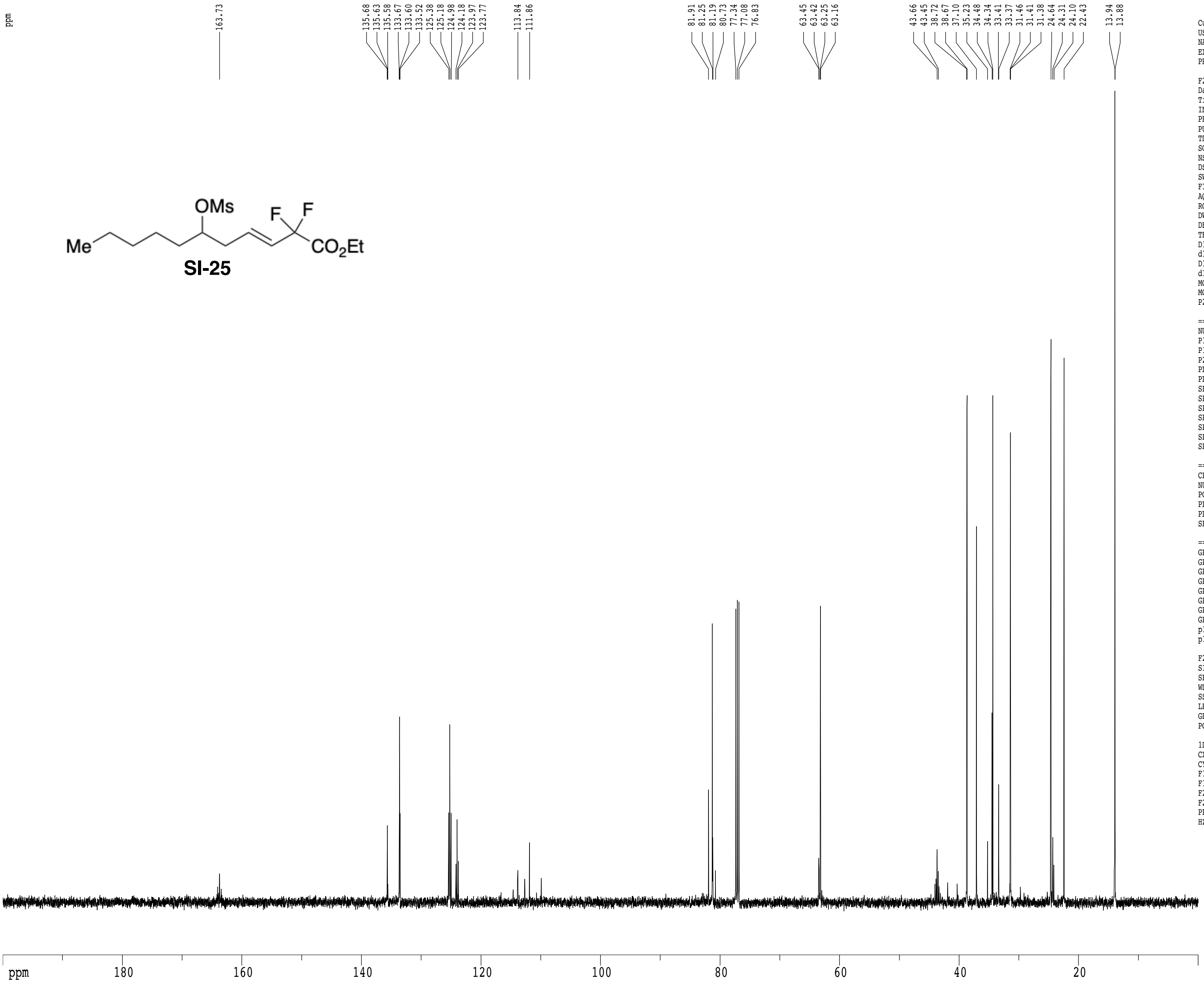


Current Data Parameters
USER mcginnit
NAME tmm-3-199
EXPNO 2
PROCNO 1

F2 - Acquisition Parameters
Date_ 20210803
Time 16.12
INSTRUM cryo500
PROBHD 5 mm CPTCI 1H-
PULPROG SpinEchopg30gp2.prd
TD 65536
SOLVENT CDCl3
NS 254
DS 16
SWH 30303.031 Hz
FIDRES 0.462388 Hz
AQ 1.0813940 sec
RG 7298.2
DW 16.500 usec
DE 6.00 usec
TE 298.0 K
D1 0.25000000 sec
d11 0.03000000 sec
D16 0.00020000 sec
d17 0.00019600 sec
MCREST 0.00000000 sec
MCWRK 0.01500000 sec
P2 37.70 usec

======== CHANNEL f1 ========
NUC1 13C
P1 18.85 usec
P12 2000.00 usec
P20 500.00 usec
PL0 120.00 dB
PL1 -1.00 dB
SFO1 125.7942548 MHz
SP2 1.55 dB
SP4 1.55 dB
SPNAM2 Crp60comp.4
SPNAM4 Crp60,0.5,20.1
SPOFF2 0.00 Hz
SPOFF4 0.00 Hz

======== CHANNEL f2 ========
CPDPRG2 waltz16
NUC2 1H
PCPD2 100.00 usec
PL2 1.60 dB
PL12 22.00 dB
SFO2 500.2225011 MHz

====== GRADIENT CHANNEL =====
GPNAM1 SINE.100
GPNAM2 SINE.100
GPX1 0.00 %
GPX2 0.00 %
GPY1 0.00 %
GPY2 0.00 %
GPZ1 30.00 %
GPZ2 50.00 %
p15 500.00 usec
p16 1000.00 usec

F2 - Processing parameters
SI 65536
SF 125.7804190 MHz
WDW EM
SSB 0
LB 1.00 Hz
GB 0
PC 2.00

1D NMR plot parameters
CX 22.80 cm
CY 15.65 cm
F1P 200.000 ppm
F1 25156.08 Hz
F2P 0.000 ppm
F2 0.00 Hz
PPMCM 8.77193 ppm/cm
HZCM 1103.33704 Hz/cm

19F spectrum

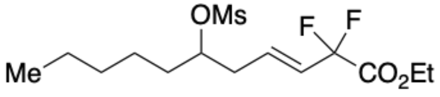


**SI-25**

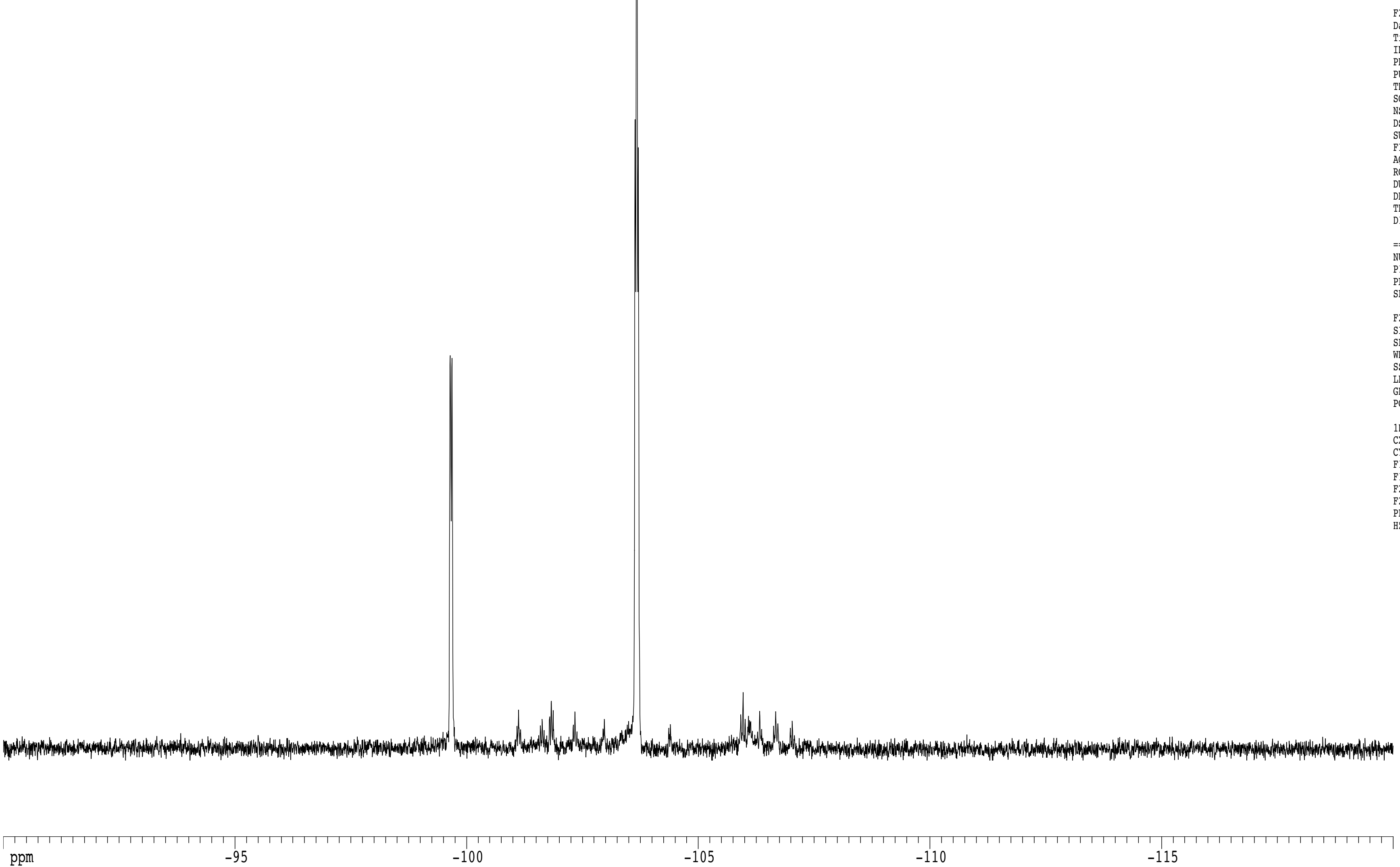


```
Current Data Parameters
USER            mcginnit
NAME           tmm-3-199
EXPNO                  3
PROCNO                 1

F2 - Acquisition Parameters
Date_           20210803
Time               15.54
INSTRUM           drx400
PROBHD   5 mm QNP H/F/P
PULPROG         zgflqn30
TD                 65536
SOLVENT            CDCl3
NS                    66
DS                     2
SWH            75187.969 Hz
FIDRES          1.147277 Hz
AQ             0.4358644 sec
RG                5160.6
DW                 6.650 usec
DE                  9.46 usec
TE                 298.0 K
D1            2.00000000 sec

======== CHANNEL f1 ========
NUC1                 19F
P1                 11.75 usec
PL1                -6.00 dB
SFO1         376.4646491 MHz

F2 - Processing parameters
SI                 65536
SF           376.4984640 MHz
WDW                   EM
SSB                    0
LB                  1.00 Hz
GB                     0
PC                  1.00

1D NMR plot parameters
CX                 22.80 cm
CY                 15.00 cm
F1P              -90.000 ppm
F1             -33884.86 Hz
F2P             -120.000 ppm
F2             -45179.82 Hz
PPMCM            1.31579 ppm/cm
HZCM           495.39273 Hz/cm
```

1H spectrum

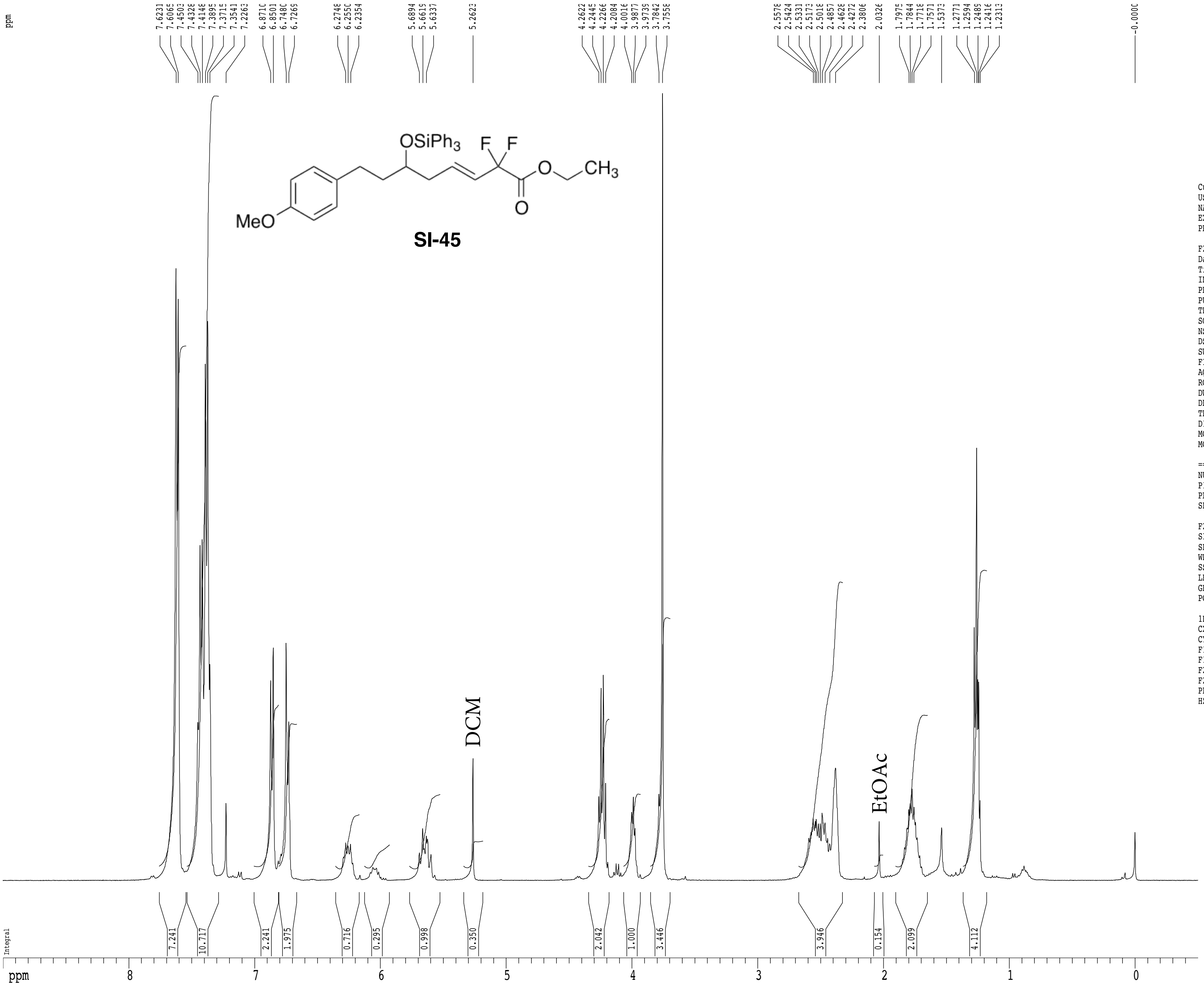


```
Current Data Parameters
USER            linpc2
NAME         pcl-2-209
EXPNO                4
PROCNO               1

F2 - Acquisition Parameters
Date_         20211018
Time             11.13
INSTRUM         drx400
PROBHD   5 mm Multinucl
PULPROG           zg30
TD               65536
SOLVENT          CDCl3
NS                   8
DS                   2
SWH          6410.256 Hz
FIDRES       0.097813 Hz
AQ          5.1118579 sec
RG                90.5
DW              78.000 usec
DE                4.50 usec
TE               297.9 K
D1          0.10000000 sec
MCREST      0.00000000 sec
MCWRK       0.01500000 sec

======== CHANNEL f1 ========
NUC1                1H
P1               12.00 usec
PL1              -1.10 dB
SFO1       400.1328009 MHz

F2 - Processing parameters
SI               65536
SF         400.1300347 MHz
WDW                 EM
SSB                  0
LB                0.30 Hz
GB                   0
PC                2.00

1D NMR plot parameters
CX               22.80 cm
CY               15.00 cm
F1P              9.000 ppm
F1             3601.17 Hz
F2P             -0.500 ppm
F2             -200.06 Hz
PPMCM          0.41667 ppm/cm
HZCM         166.72086 Hz/cm
```

**SFC Chiracel OD-H, 1.5% IPA/$CO_2$, 2.7 mL/min**

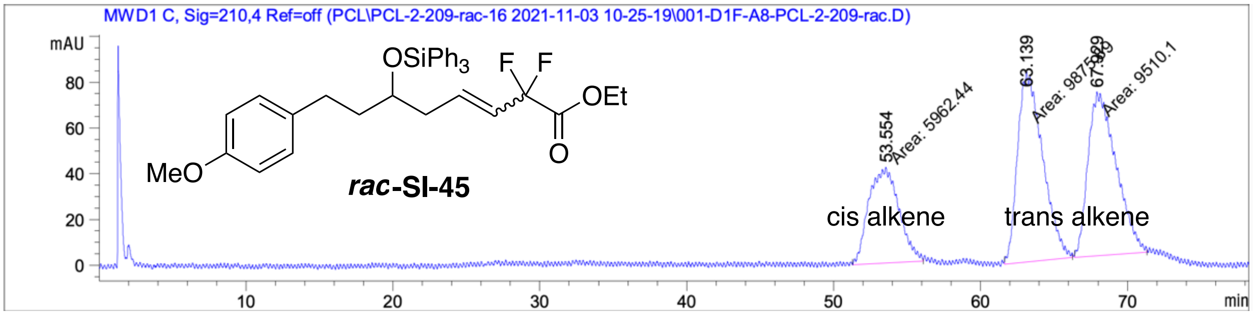


```
Signal 3: MWD1 C, Sig=210,4 Ref=off

Peak RetTime Type  Width     Area       Height     Area
  #   [min]        [min]   [mAU*s]      [mAU]       %
----|-------|----|-------|----------|----------|--------|
   1  53.554 MM     2.3714 5962.44385   41.90471  23.5219
   2  63.139 MM     1.9739 9875.88672   83.38895  38.9605
   3  67.929 MM     2.2126 9510.09961   71.63718  37.5175

Totals :                   2.53484e4   196.93084
```

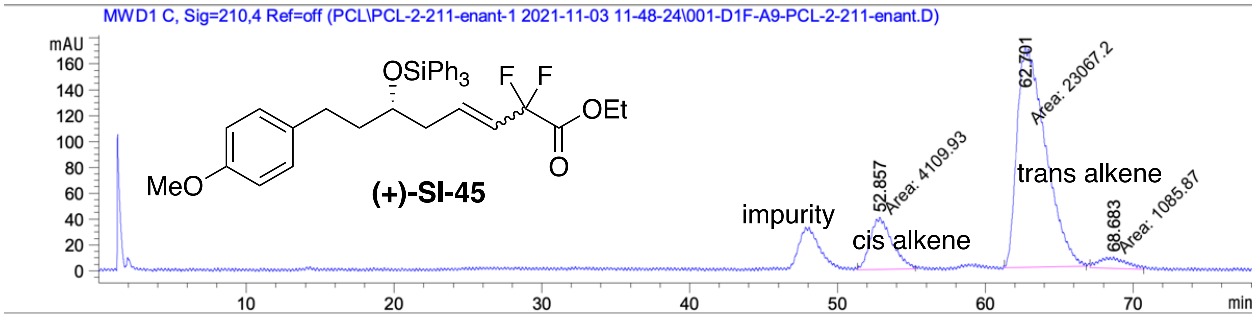


```
Signal 3: MWD1 C, Sig=210,4 Ref=off

Peak RetTime Type  Width     Area       Height     Area
  #   [min]        [min]   [mAU*s]      [mAU]       %
----|-------|----|-------|----------|----------|--------|
   1  52.857 MM     1.7057 4109.93311   40.15873  14.5417
   2  62.701 MM     2.2477 2.30672e4   171.04480  81.6162
   3  68.683 MM     2.0085 1085.87341    9.01059   3.8420

Totals :                   2.82630e4   220.21412
```

1H spectrum

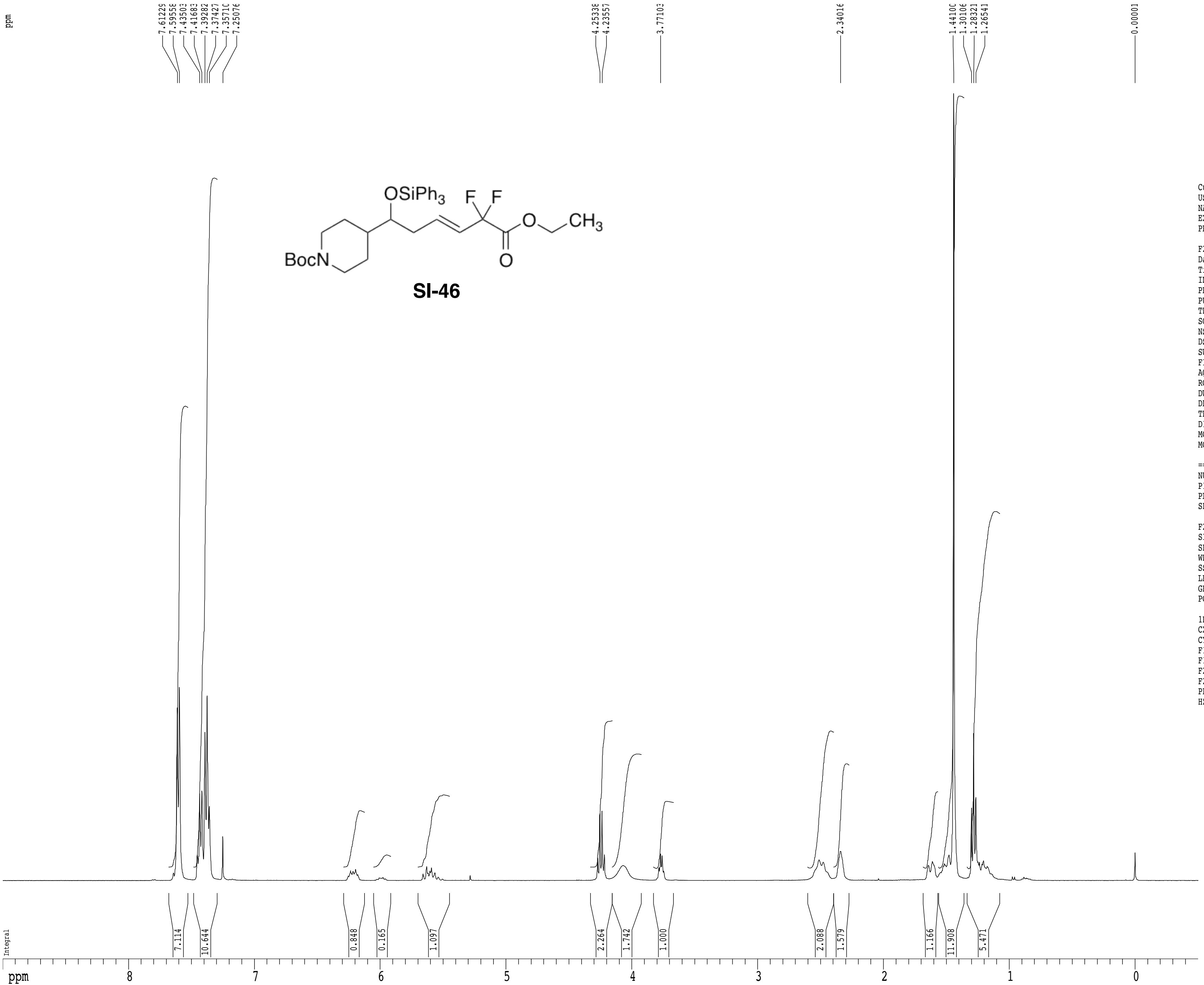


**SI-46**

Current Data Parameters
USER linpc2
NAME pcl-2-207
EXPNO 2
PROCNO 1

F2 - Acquisition Parameters
Date_ 20211013
Time 11.36
INSTRUM drx400
PROBHD 5 mm Multinucl
PULPROG zg30
TD 65536
SOLVENT CDCl3
NS 8
DS 2
SWH 6410.256 Hz
FIDRES 0.097813 Hz
AQ 5.1118579 sec
RG 143.7
DW 78.000 usec
DE 4.50 usec
TE 298.0 K
D1 0.10000000 sec
MCREST 0.00000000 sec
MCWRK 0.01500000 sec

======== CHANNEL f1 ========
NUC1 1H
P1 12.00 usec
PL1 -1.10 dB
SFO1 400.1328009 MHz

F2 - Processing parameters
SI 65536
SF 400.1300251 MHz
WDW EM
SSB 0
LB 0.30 Hz
GB 0
PC 2.00

1D NMR plot parameters
CX 22.80 cm
CY 15.00 cm
F1P 9.000 ppm
F1 3601.17 Hz
F2P -0.500 ppm
F2 -200.06 Hz
PPMCM 0.41667 ppm/cm
HZCM 166.72086 Hz/cm

**SFC Chiracel OD-H, 1.5% IPA/$CO_2$, 2.7 mL/min**

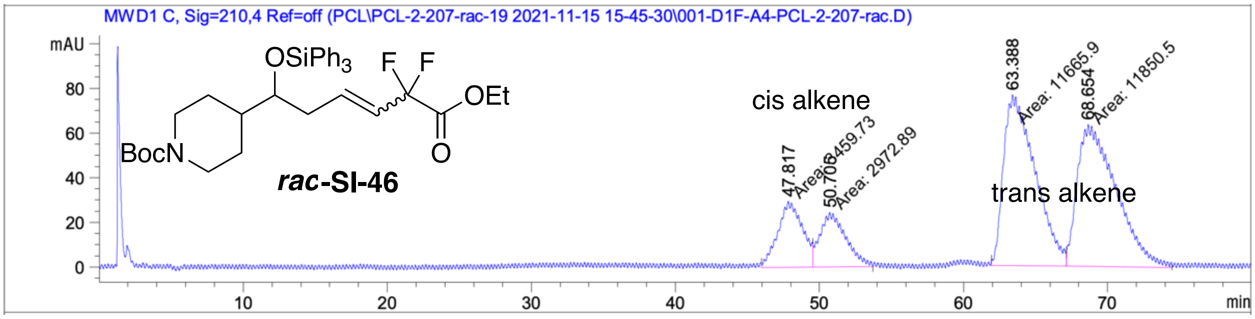


Signal 3: MWD1 C, Sig=210,4 Ref=off

| Peak # | RetTime [min] | Type | Width [min] | Area [mAU*s] | Height [mAU] | Area % |
|---|---|---|---|---|---|---|
| 1 | 47.817 | MF | 1.9667 | 3459.72656 | 29.31921 | 11.5521 |
| 2 | 50.705 | FM | 2.0363 | 2972.88721 | 24.33255 | 9.9265 |
| 3 | 63.388 | MF | 2.5407 | 1.16659e4 | 76.52760 | 38.9526 |
| 4 | 68.654 | FM | 3.1103 | 1.18505e4 | 63.50021 | 39.5688 |
| Totals : | | | | 2.99490e4 | 193.67958 | |

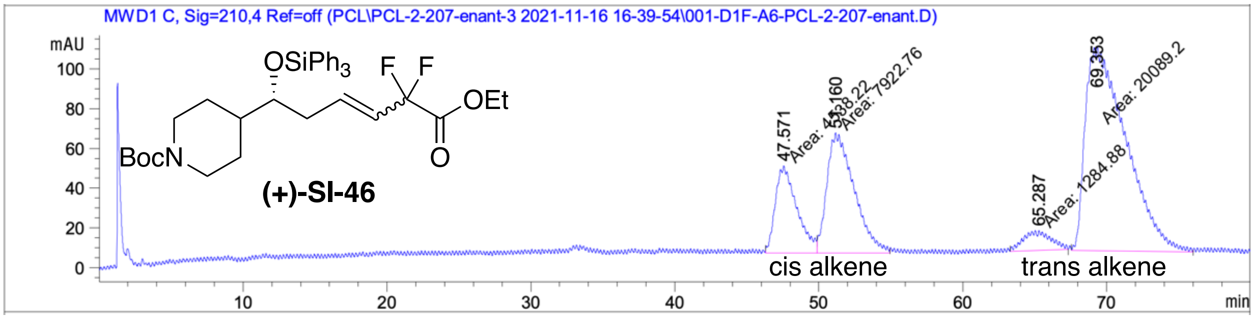


Signal 3: MWD1 C, Sig=210,4 Ref=off

| Peak # | RetTime [min] | Type | Width [min] | Area [mAU*s] | Height [mAU] | Area % |
|---|---|---|---|---|---|---|
| 1 | 47.571 | MF | 1.7281 | 4538.22021 | 43.76947 | 13.4128 |
| 2 | 51.160 | FM | 2.1847 | 7922.75977 | 60.44085 | 23.4158 |
| 3 | 65.287 | MM | 2.1543 | 1284.88037 | 9.94045 | 3.7975 |
| 4 | 69.353 | MM | 3.2621 | 2.00892e4 | 102.64028 | 59.3739 |
| Totals : | | | | 3.38351e4 | 216.79105 | |

1H spectrum

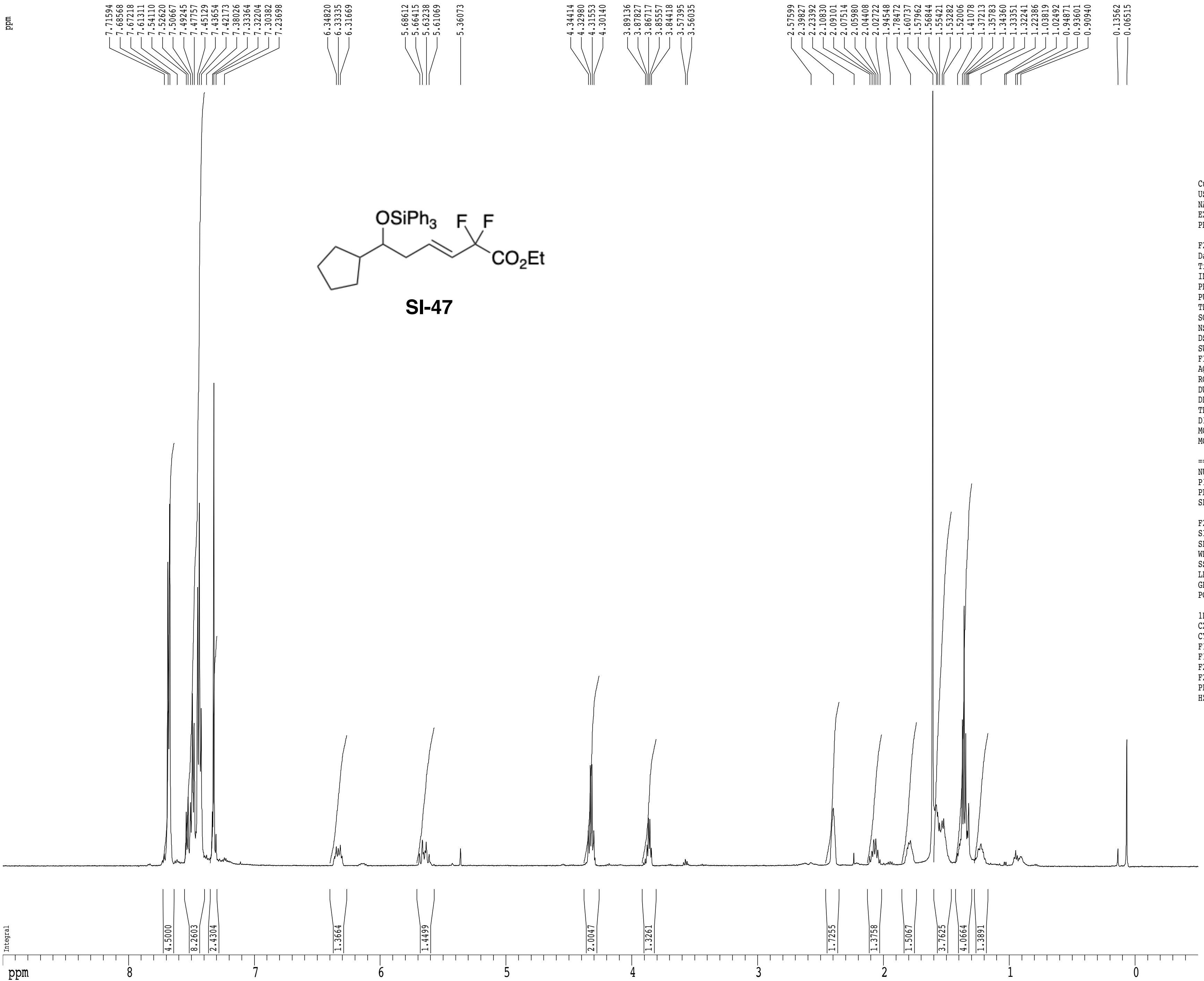


**SI-47**

| | | |
|---|---|---|
| Current Data Parameters | | |
| USER | mcginnit | |
| NAME | tmm-3-252 | |
| EXPNO | 1 | |
| PROCNO | 1 | |
| F2 - Acquisition Parameters | | |
| Date_ | 20211005 | |
| Time | 16.54 | |
| INSTRUM | cryo500 | |
| PROBHD | 5 mm CPTCI 1H- | |
| PULPROG | zg30 | |
| TD | 81728 | |
| SOLVENT | CDCl3 | |
| NS | 8 | |
| DS | 2 | |
| SWH | 8012.820 | Hz |
| FIDRES | 0.098043 | Hz |
| AQ | 5.0998774 | sec |
| RG | 7.1 | |
| DW | 62.400 | usec |
| DE | 6.00 | usec |
| TE | 298.0 | K |
| D1 | 0.10000000 | sec |
| MCREST | 0.00000000 | sec |
| MCWRK | 0.01500000 | sec |
| ======== CHANNEL f1 ======== | | |
| NUC1 | 1H | |
| P1 | 9.75 | usec |
| PL1 | 1.60 | dB |
| SFO1 | 500.2235015 | MHz |
| F2 - Processing parameters | | |
| SI | 65536 | |
| SF | 500.2200000 | MHz |
| WDW | EM | |
| SSB | 0 | |
| LB | 0.30 | Hz |
| GB | 0 | |
| PC | 1.00 | |
| 1D NMR plot parameters | | |
| CX | 22.80 | cm |
| CY | 15.00 | cm |
| F1P | 9.000 | ppm |
| F1 | 4501.98 | Hz |
| F2P | -0.500 | ppm |
| F2 | -250.11 | Hz |
| PPMCM | 0.41667 | ppm/cm |
| HZCM | 208.42500 | Hz/cm |

**HPLC Chiracel OD, 0.5% IPA/hexanes, 2.0 mL/min**

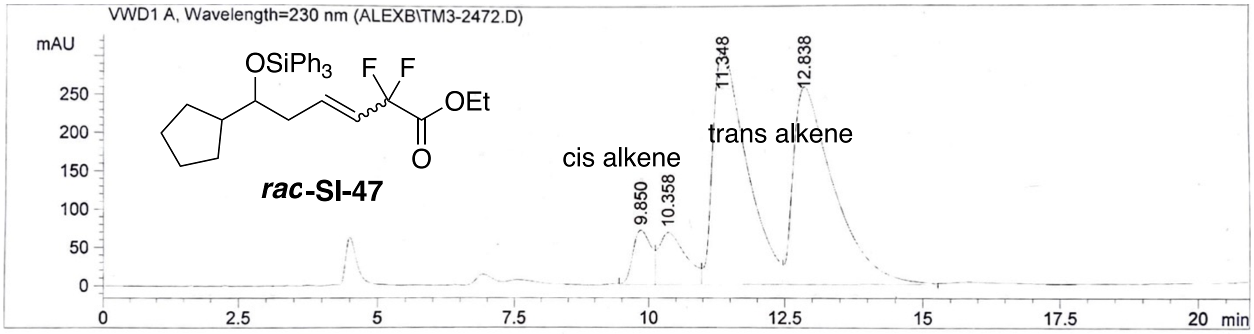


Signal 2: VWD1 A, Wavelength=230 nm

| Peak # | RetTime [min] | Type | Width [min] | Area mAU *s | Height [mAU ] | Area % |
|---|---|---|---|---|---|---|
| 1 | 9.850 | VV | 0.3619 | 1688.66687 | 72.13825 | 5.4929 |
| 2 | 10.358 | VV | 0.5061 | 2398.41235 | 68.98889 | 7.8015 |
| 3 | 11.348 | VV | 0.6286 | 1.32314e4 | 311.13757 | 43.0389 |
| 4 | 12.838 | VB | 0.7396 | 1.34244e4 | 259.23230 | 43.6667 |

Totals : 3.07429e4 711.49702

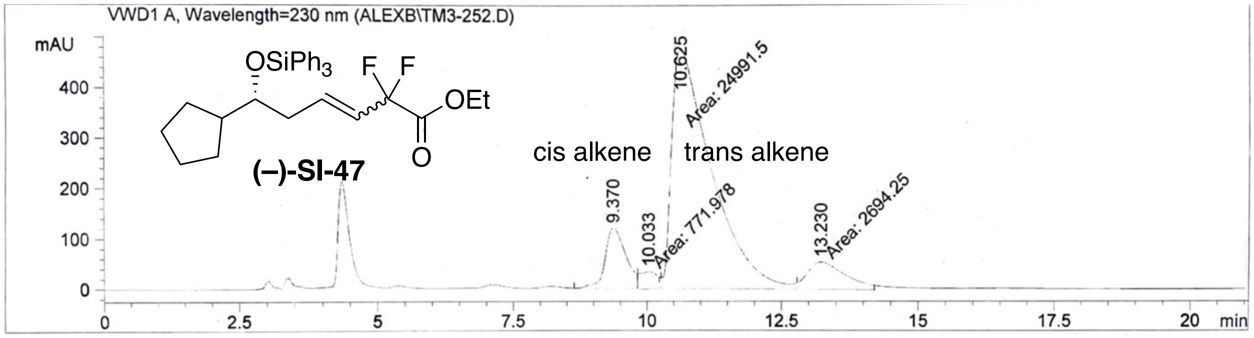


Signal 2: VWD1 A, Wavelength=230 nm

| Peak # | RetTime [min] | Type | Width [min] | Area mAU *s | Height [mAU ] | Area % |
|---|---|---|---|---|---|---|
| 1 | 9.370 | VV | 0.4036 | 3327.68066 | 122.19165 | 10.4692 |
| 2 | 10.033 | MM | 0.3603 | 771.97833 | 35.71170 | 2.4287 |
| 3 | 10.625 | MF | 0.8656 | 2.49915e4 | 481.19406 | 78.6257 |
| 4 | 13.230 | MF | 0.8141 | 2694.25488 | 55.15702 | 8.4764 |

Totals : 3.17855e4 694.25444

1H spectrum

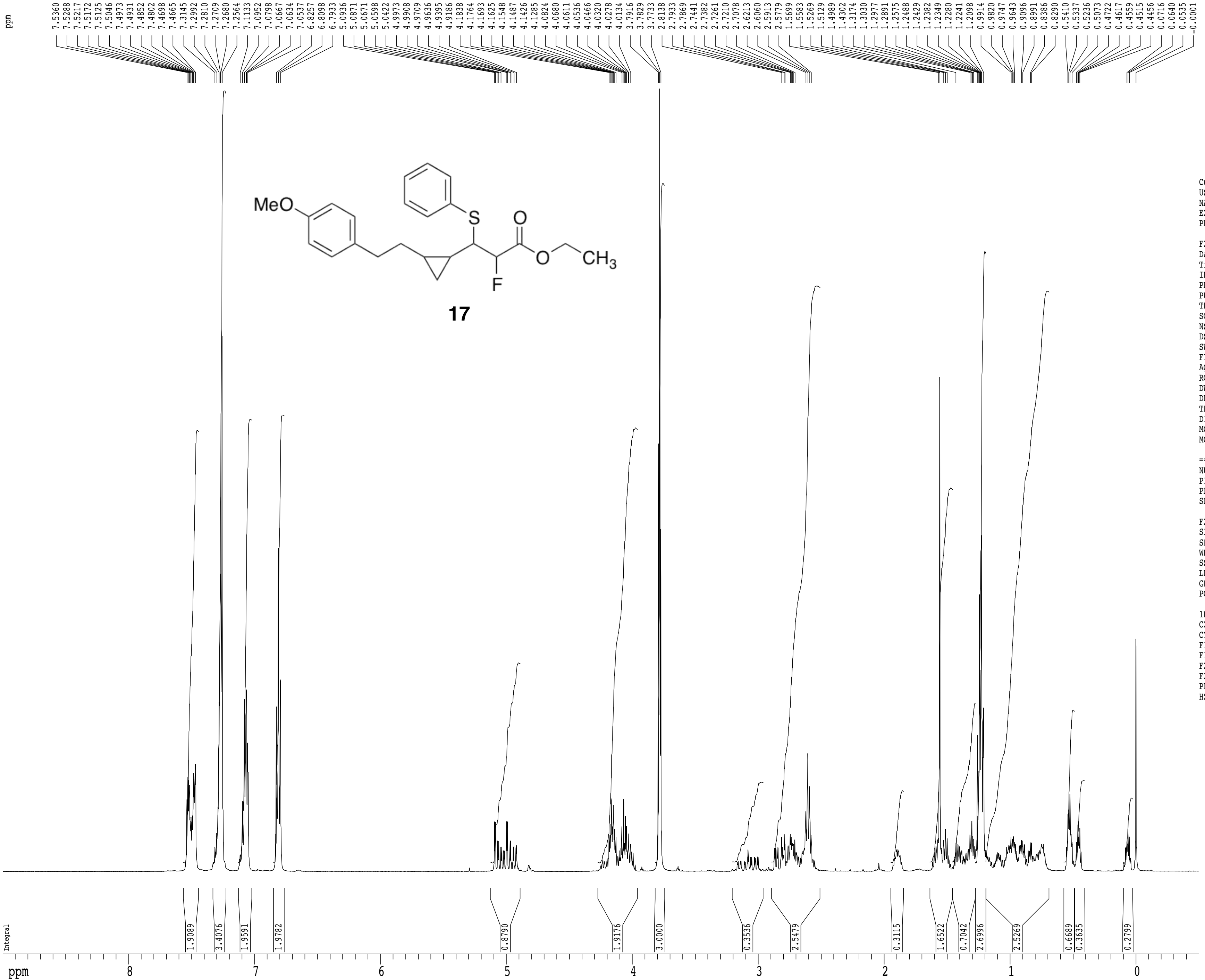


Current Data Parameters
USER linpc2
NAME pcl-2-119
EXPNO 2
PROCNO 1

F2 - Acquisition Parameters
Date_ 20210724
Time 12.51
INSTRUM cryo500
PROBHD 5 mm CPTCI 1H-
PULPROG zg30
TD 81728
SOLVENT CDCl3
NS 8
DS 2
SWH 8012.820 Hz
FIDRES 0.098043 Hz
AQ 5.0998774 sec
RG 7.1
DW 62.400 usec
DE 6.00 usec
TE 298.0 K
D1 0.10000000 sec
MCREST 0.00000000 sec
MCWRK 0.01500000 sec

======== CHANNEL f1 ========
NUC1 1H
P1 9.75 usec
PL1 1.60 dB
SFO1 500.2235015 MHz

F2 - Processing parameters
SI 65536
SF 500.2200329 MHz
WDW EM
SSB 0
LB 0.30 Hz
GB 0
PC 1.00

1D NMR plot parameters
CX 22.80 cm
CY 15.00 cm
F1P 9.000 ppm
F1 4501.98 Hz
F2P -0.500 ppm
F2 -250.11 Hz
PPMCM 0.41667 ppm/cm
HZCM 208.42502 Hz/cm

Z-restored spin-echo 13C spectrum with 1H decoupling

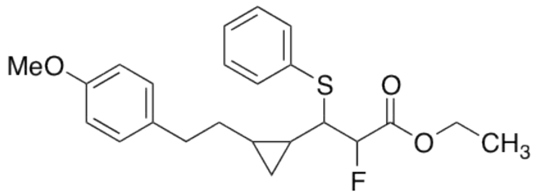


**17**

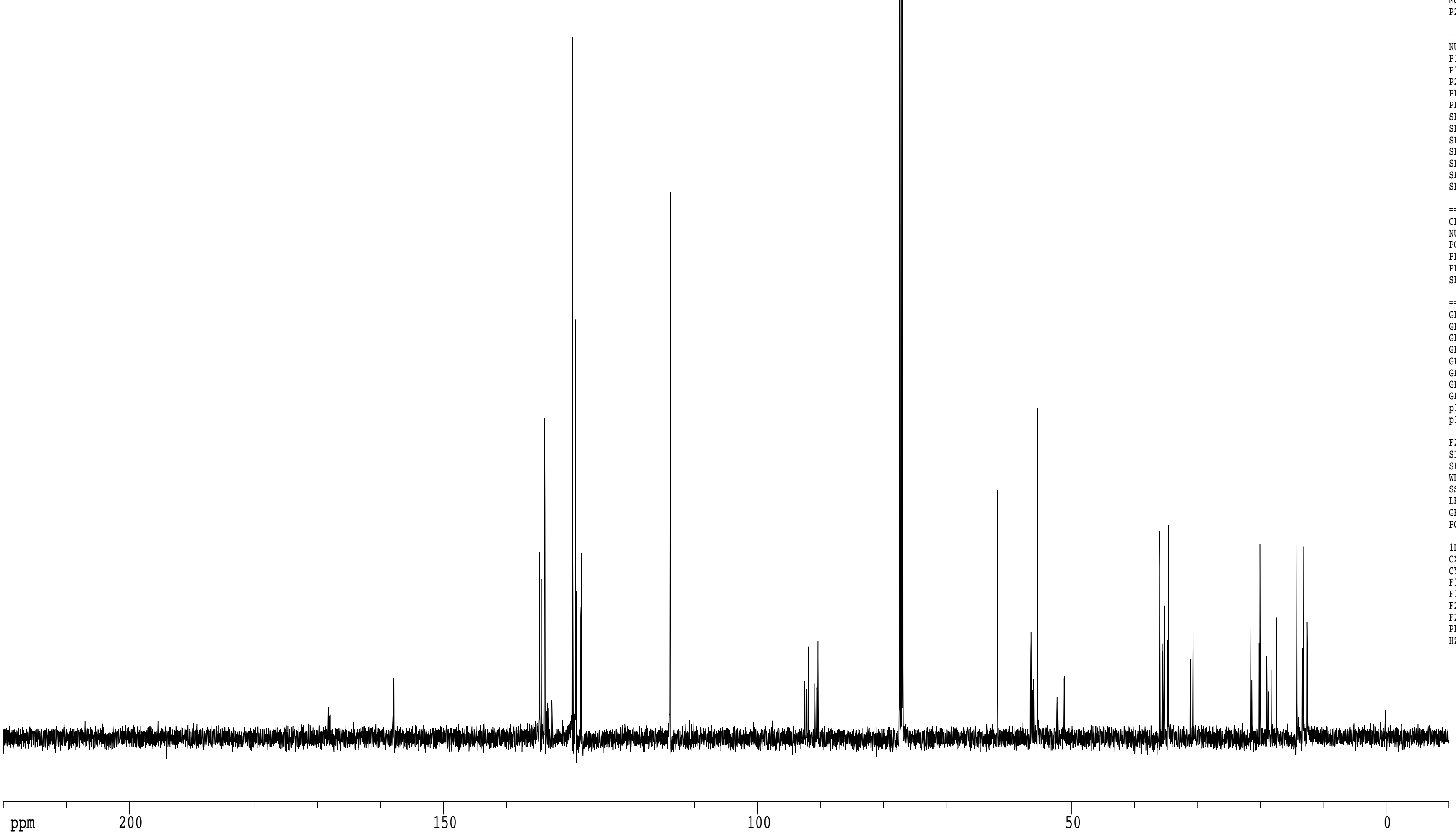


```
Current Data Parameters
USER            linpc2
NAME          pcl-2-119
EXPNO                 3
PROCNO                1

F2 - Acquisition Parameters
Date_          20210724
Time              12.54
INSTRUM         cryo500
PROBHD   5 mm CPTCI 1H-
PULPROG  SpinEchopg30gp2.prd
TD                65536
SOLVENT           CDCl3
NS                  360
DS                   16
SWH           30303.031 Hz
FIDRES         0.462388 Hz
AQ            1.0813940 sec
RG                 3251
DW               16.500 usec
DE                 6.00 usec
TE                298.0 K
D1           0.25000000 sec
d11          0.03000000 sec
D16          0.00020000 sec
d17          0.00019600 sec
MCREST       0.00000000 sec
MCWRK        0.01500000 sec
P2                37.70 usec

======== CHANNEL f1 ========
NUC1                13C
P1                18.85 usec
P12             2000.00 usec
P20              500.00 usec
PL0              120.00 dB
PL1               -1.00 dB
SFO1        125.7942548 MHz
SP2                1.55 dB
SP4                1.55 dB
SPNAM2      Crp60comp.4
SPNAM4   Crp60,0.5,20.1
SPOFF2             0.00 Hz
SPOFF4             0.00 Hz

======== CHANNEL f2 ========
CPDPRG2         waltz16
NUC2                 1H
PCPD2            100.00 usec
PL2                1.60 dB
PL12              22.00 dB
SFO2        500.2225011 MHz

====== GRADIENT CHANNEL =====
GPNAM1         SINE.100
GPNAM2         SINE.100
GPX1               0.00 %
GPX2               0.00 %
GPY1               0.00 %
GPY2               0.00 %
GPZ1              30.00 %
GPZ2              50.00 %
p15              500.00 usec
p16             1000.00 usec

F2 - Processing parameters
SI                65536
SF          125.7804071 MHz
WDW                  EM
SSB                   0
LB                 1.00 Hz
GB                    0
PC                 2.00

1D NMR plot parameters
CX                22.80 cm
CY                15.00 cm
F1P             220.000 ppm
F1             27671.69 Hz
F2P             -10.000 ppm
F2             -1257.80 Hz
PPMCM          10.08772 ppm/cm
HZCM         1268.83752 Hz/cm
```

f19 spectrum

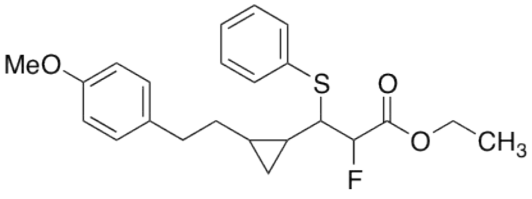

**17**

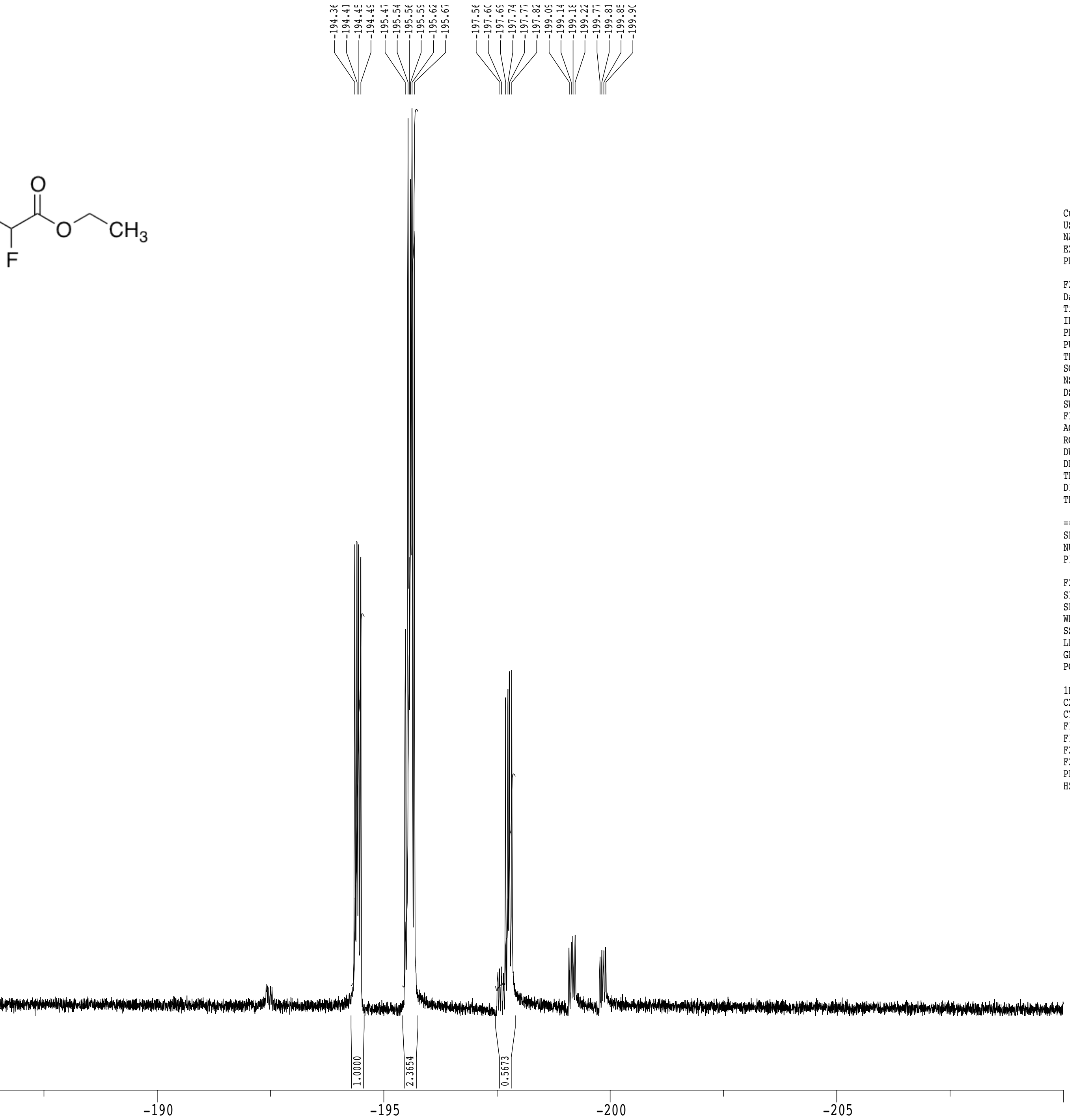


Current Data Parameters
USER linpc2
NAME pcl-2-119
EXPNO 6
PROCNO 1

F2 - Acquisition Parameters
Date_ 20210802
Time 17.11
INSTRUM av600
PROBHD 5 mm CPBBO BB-
PULPROG zgflqn30
TD 131072
SOLVENT CDCl3T
NS 16
DS 2
SWH 178571.422 Hz
FIDRES 1.362392 Hz
AQ 0.3670516 sec
RG 912
DW 2.800 usec
DE 18.00 usec
TE 298.0 K
D1 3.00000000 sec
TD0 1

======== CHANNEL f1 ========
SFO1 564.6299196 MHz
NUC1 19F
P1 18.25 usec

F2 - Processing parameters
SI 131072
SF 564.6864229 MHz
WDW no
SSB 0
LB 0.00 Hz
GB 0
PC 1.00

1D NMR plot parameters
CX 22.80 cm
CY 15.00 cm
F1P -180.000 ppm
F1 -101643.55 Hz
F2P -210.000 ppm
F2 -118584.14 Hz
PPMCM 1.31579 ppm/cm
HZCM 743.00842 Hz/cm

1H spectrum

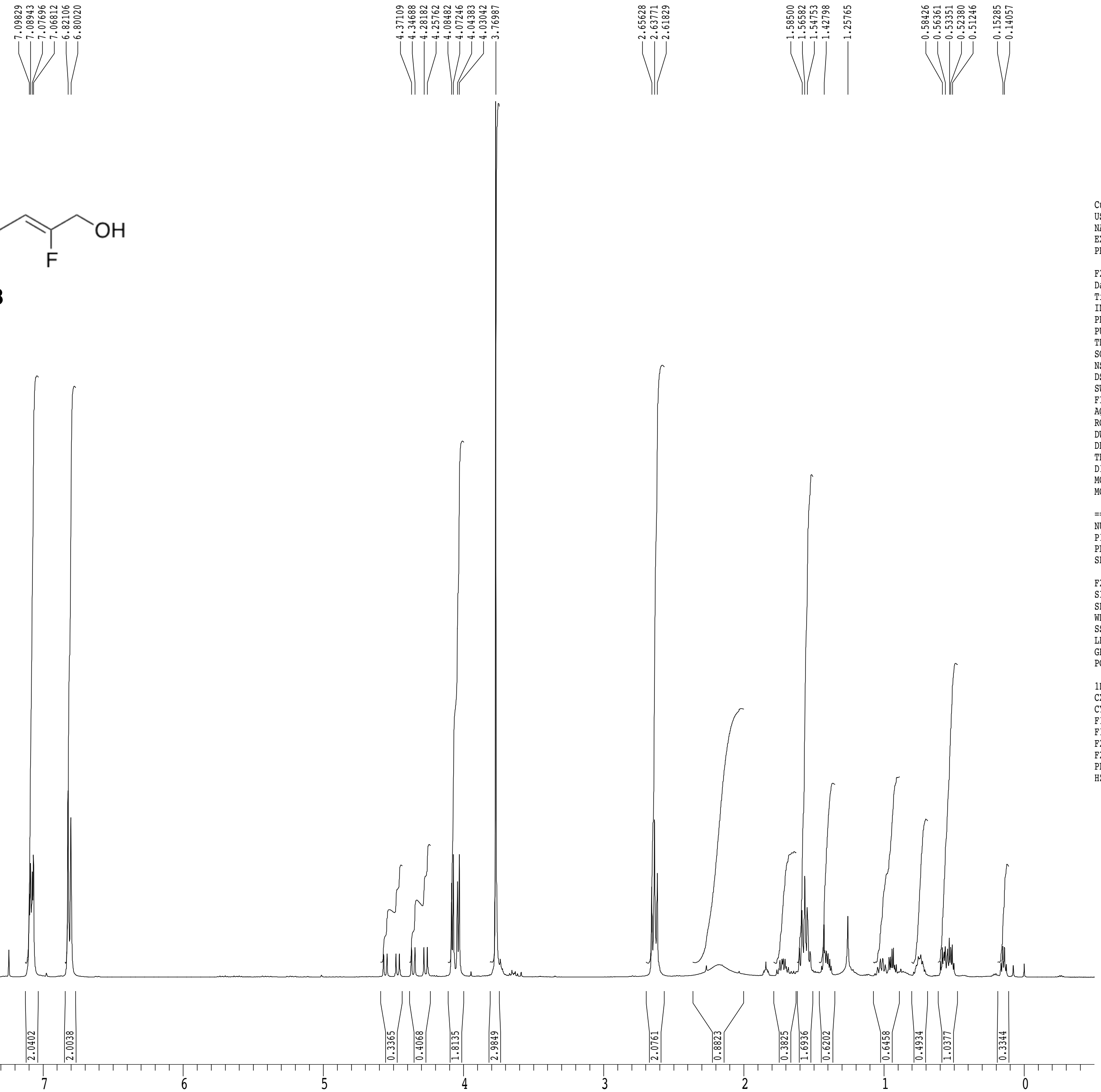


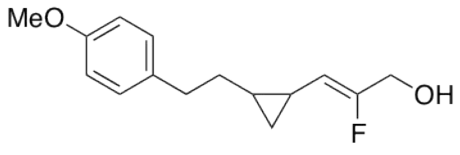


**18**

```
Current Data Parameters
USER              linpc2
NAME            pcl-2-072
EXPNO                   1
PROCNO                  1

F2 - Acquisition Parameters
Date_           20210612
Time               11.51
INSTRUM           drx400
PROBHD   5 mm QNP H/F/P
PULPROG             zg30
TD                 65536
SOLVENT            CDCl3
NS                     8
DS                     2
SWH             6410.256 Hz
FIDRES          0.097813 Hz
AQ             5.1118579 sec
RG                  50.8
DW                78.000 usec
DE                  4.50 usec
TE                 298.0 K
D1            0.10000000 sec
MCREST        0.00000000 sec
MCWRK         0.01500000 sec

======== CHANNEL f1 ========
NUC1                  1H
P1                 12.00 usec
PL1                -1.60 dB
SFO1         400.1328009 MHz

F2 - Processing parameters
SI                 65536
SF           400.1300279 MHz
WDW                   EM
SSB                    0
LB                  0.30 Hz
GB                     0
PC                  2.00

1D NMR plot parameters
CX                 22.80 cm
CY                 15.00 cm
F1P                9.000 ppm
F1               3601.17 Hz
F2P               -0.500 ppm
F2               -200.06 Hz
PPMCM            0.41667 ppm/cm
HZCM           166.72086 Hz/cm
```

1H spectrum

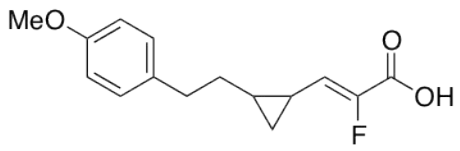

**19**

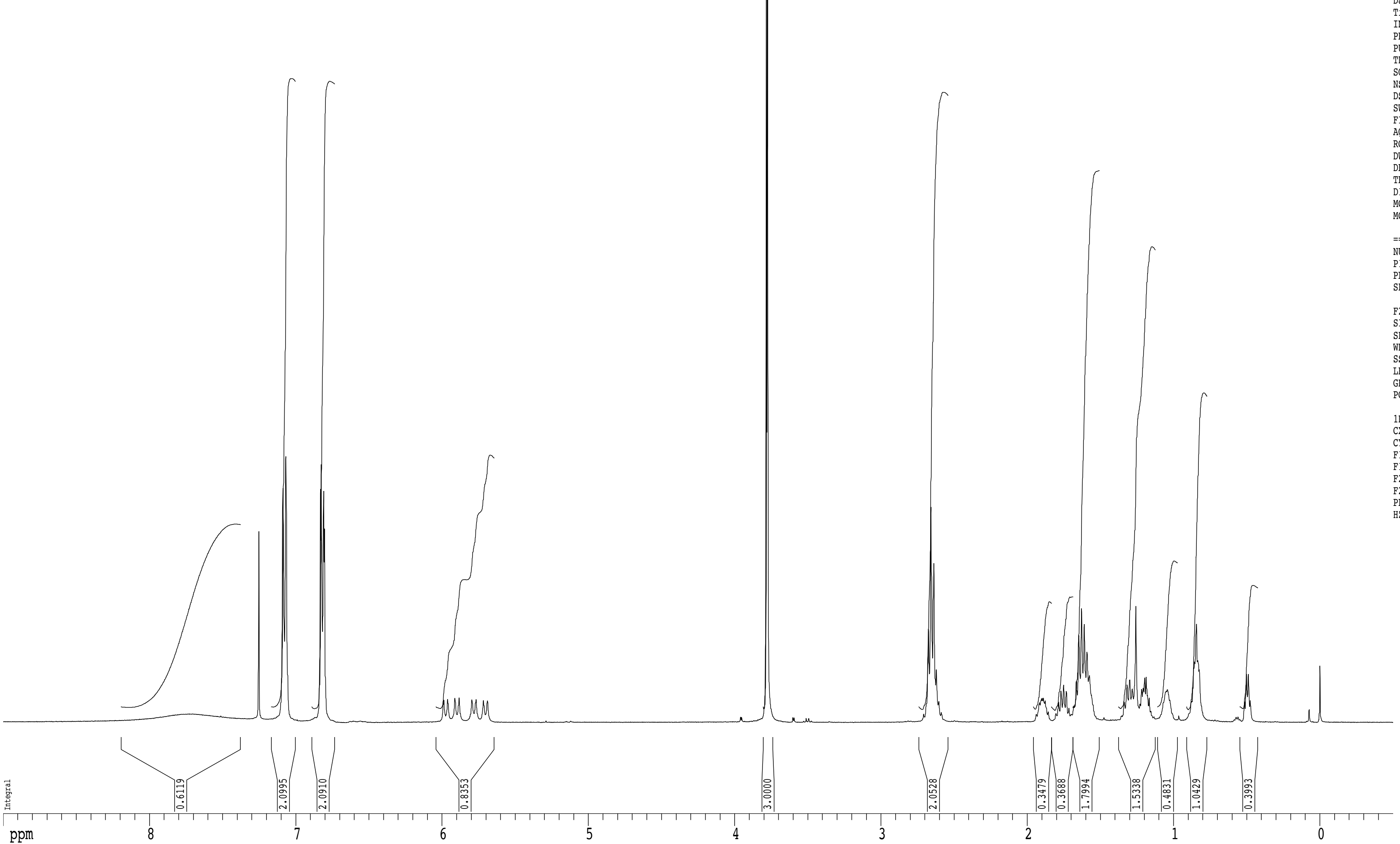


Current Data Parameters
USER linpc2
NAME pcl-2-143
EXPNO 1
PROCNO 1

F2 - Acquisition Parameters
Date_ 20210809
Time 16.40
INSTRUM drx400
PROBHD 5 mm QNP H/F/P
PULPROG zg30
TD 65536
SOLVENT CDCl3
NS 8
DS 2
SWH 6410.256 Hz
FIDRES 0.097813 Hz
AQ 5.1118579 sec
RG 203.2
DW 78.000 usec
DE 4.50 usec
TE 298.0 K
D1 0.10000000 sec
MCREST 0.00000000 sec
MCWRK 0.01500000 sec

======== CHANNEL f1 ========
NUC1 1H
P1 12.00 usec
PL1 -1.60 dB
SFO1 400.1328009 MHz

F2 - Processing parameters
SI 65536
SF 400.1300245 MHz
WDW EM
SSB 0
LB 0.30 Hz
GB 0
PC 2.00

1D NMR plot parameters
CX 22.80 cm
CY 15.00 cm
F1P 9.000 ppm
F1 3601.17 Hz
F2P -0.500 ppm
F2 -200.06 Hz
PPMCM 0.41667 ppm/cm
HZCM 166.72086 Hz/cm

13C spectrum

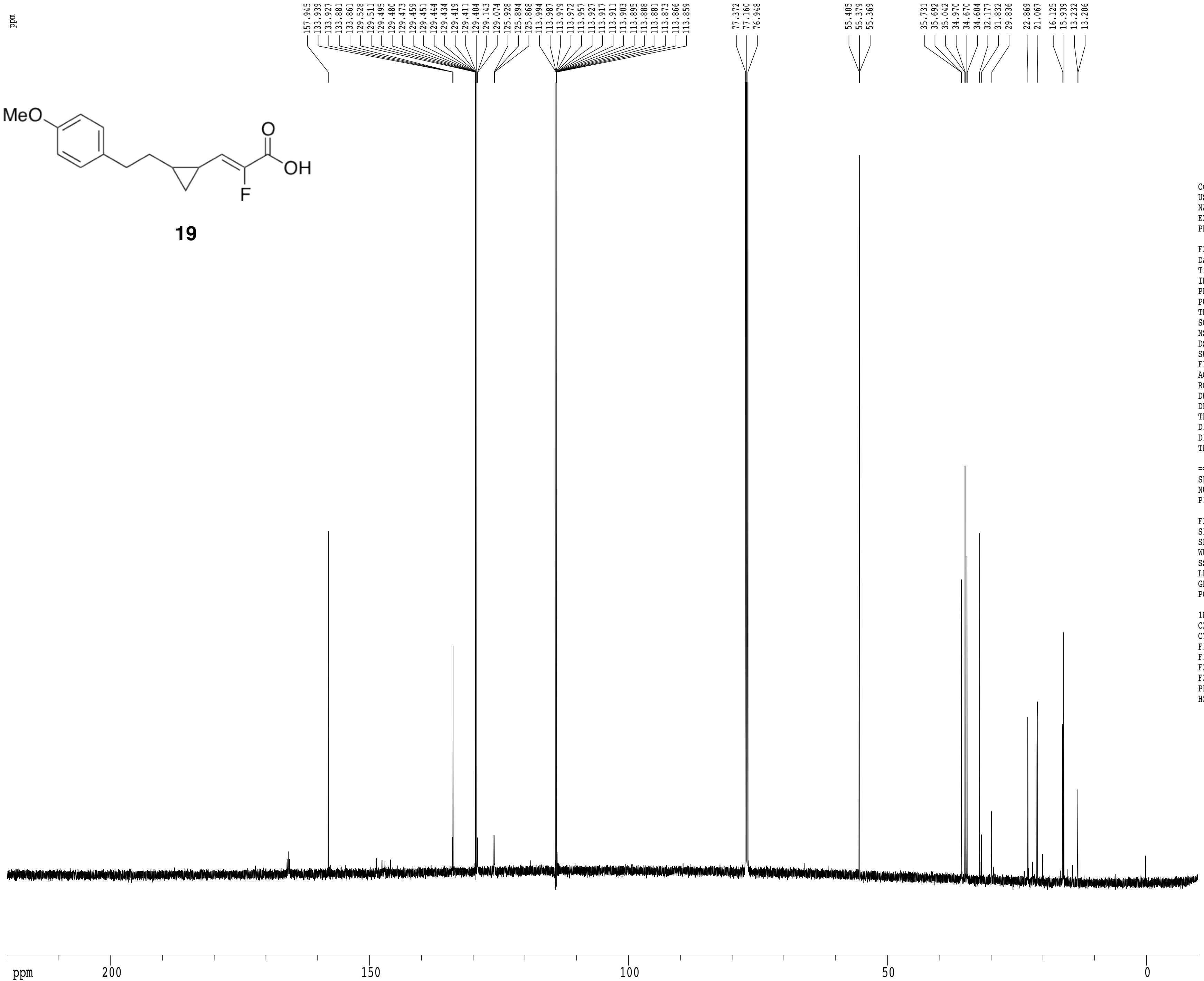


Current Data Parameters
USER linpc2
NAME pcl-2-143
EXPNO 4
PROCNO 1

F2 - Acquisition Parameters
Date_ 20210810
Time 12.14
INSTRUM av600
PROBHD 5 mm CPBBO BB-
PULPROG zgdc30
TD 65536
SOLVENT CDCl3T
NS 593
DS 4
SWH 36231.883 Hz
FIDRES 0.552855 Hz
AQ 0.9044468 sec
RG 2050
DW 13.800 usec
DE 19.63 usec
TE 298.0 K
D1 0.40000001 sec
D11 0.03000000 sec
TD0 1

======== CHANNEL f1 ========
SFO1 150.9194080 MHz
NUC1 13C
P1 10.10 usec

F2 - Processing parameters
SI 65536
SF 150.9027964 MHz
WDW no
SSB 0
LB 0.00 Hz
GB 0
PC 1.00

1D NMR plot parameters
CX 22.80 cm
CY 45.00 cm
F1P 220.000 ppm
F1 33198.62 Hz
F2P -10.000 ppm
F2 -1509.03 Hz
PPMCM 10.08772 ppm/cm
HZCM 1522.26514 Hz/cm

19F spectrum

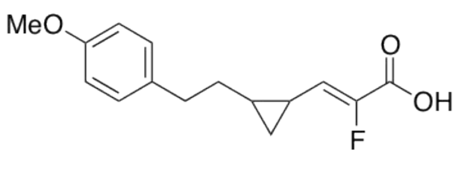


**19**

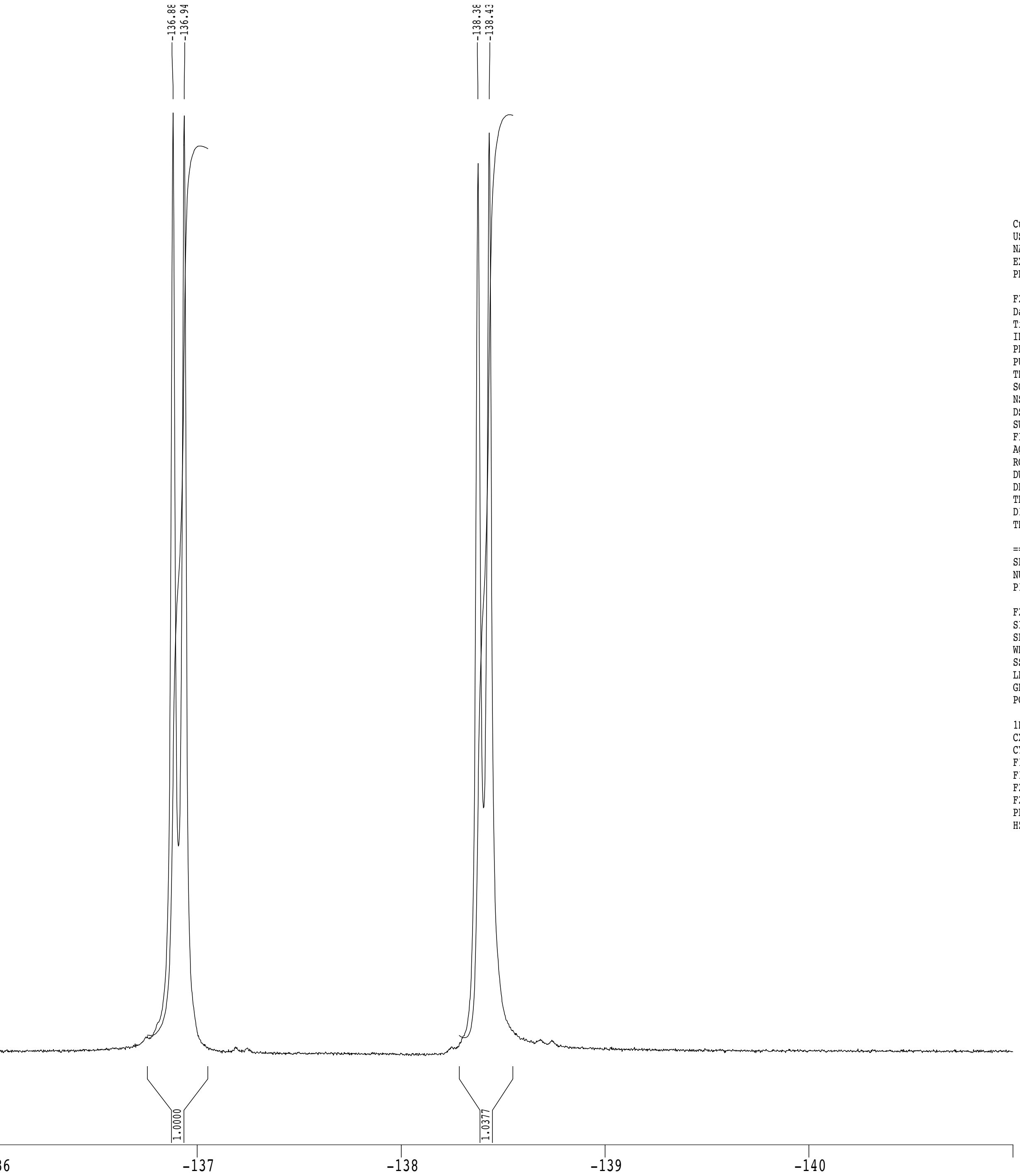


```
Current Data Parameters
USER              linpc2
NAME           pcl-2-143
EXPNO                  3
PROCNO                 1

F2 - Acquisition Parameters
Date_           20210810
Time               12.10
INSTRUM            av600
PROBHD   5 mm CPBBO BB-
PULPROG         zgflqn30
TD                131072
SOLVENT           CDCl3T
NS                    16
DS                     2
SWH           178571.422 Hz
FIDRES          1.362392 Hz
AQ             0.3670516 sec
RG                   575
DW                 2.800 usec
DE                 18.00 usec
TE                 298.0 K
D1            3.00000000 sec
TD0                    1

======== CHANNEL f1 ========
SFO1        564.6299196 MHz
NUC1                 19F
P1                 18.25 usec

F2 - Processing parameters
SI                131072
SF           564.6864244 MHz
WDW                   no
SSB                    0
LB                  0.00 Hz
GB                     0
PC                  1.00

1D NMR plot parameters
CX                 22.80 cm
CY                 15.00 cm
F1P             -134.000 ppm
F1             -75667.98 Hz
F2P             -141.000 ppm
F2             -79620.78 Hz
PPMCM            0.30702 ppm/cm
HZCM           173.36864 Hz/cm
```

1H spectrum

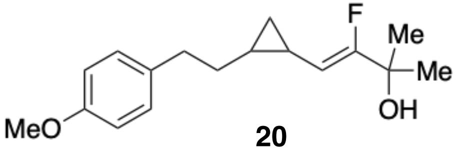

**20**

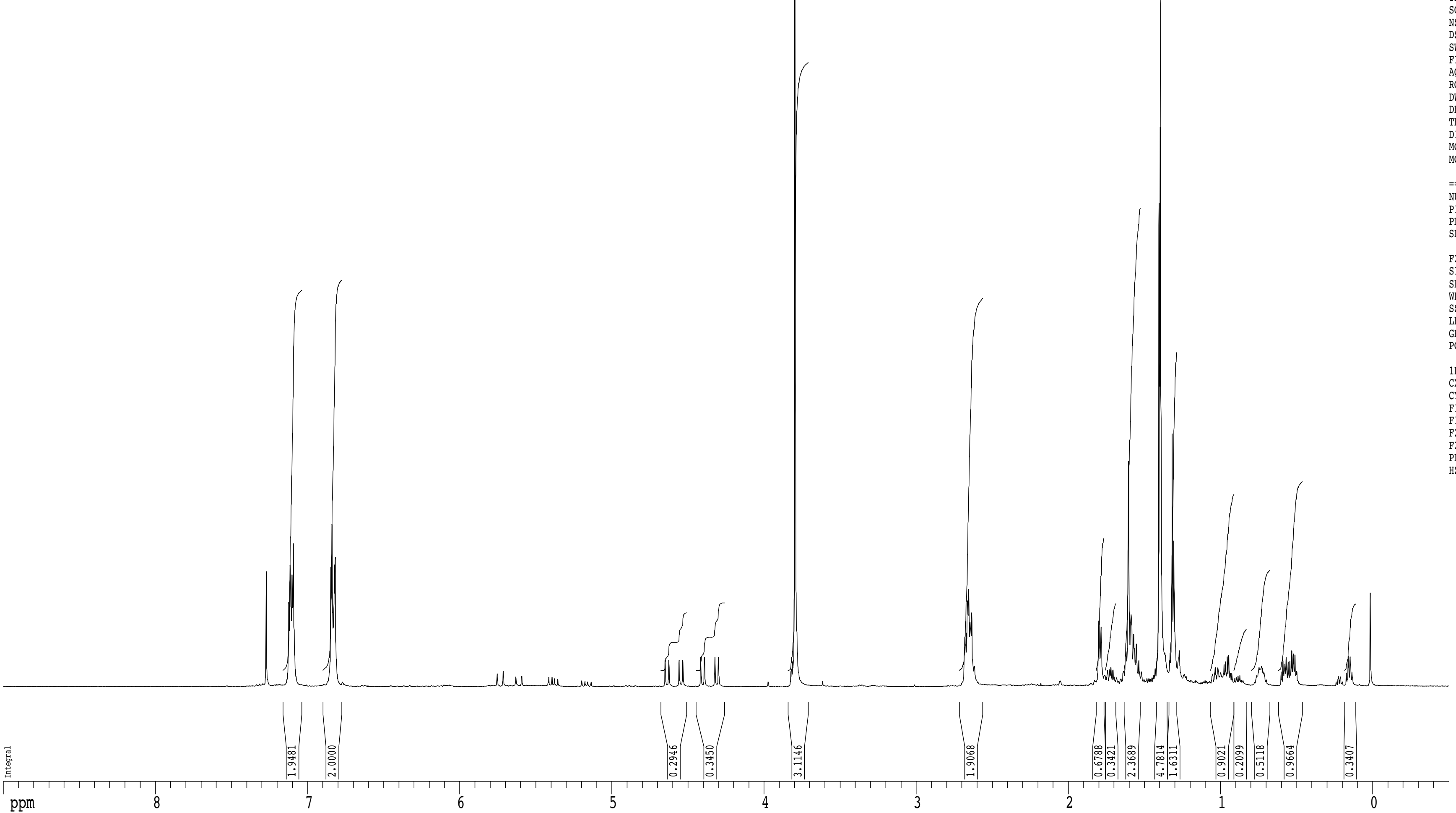


```
Current Data Parameters
USER            mcginnit
NAME      tmm-3-221-char
EXPNO                  1
PROCNO                 1

F2 - Acquisition Parameters
Date_           20210906
Time               13.20
INSTRUM           drx400
PROBHD   5 mm Multinucl
PULPROG             zg30
TD                 65536
SOLVENT            CDCl3
NS                     8
DS                     2
SWH             6410.256 Hz
FIDRES          0.097813 Hz
AQ             5.1118579 sec
RG                 322.5
DW                78.000 usec
DE                  4.50 usec
TE                 298.1 K
D1            0.10000000 sec
MCREST        0.00000000 sec
MCWRK         0.01500000 sec

======== CHANNEL f1 ========
NUC1                  1H
P1                 12.00 usec
PL1                -1.10 dB
SFO1         400.1328009 MHz

F2 - Processing parameters
SI                 65536
SF           400.1300175 MHz
WDW                   EM
SSB                    0
LB                  0.30 Hz
GB                     0
PC                  2.00

1D NMR plot parameters
CX                 22.80 cm
CY                 15.00 cm
F1P                9.000 ppm
F1               3601.17 Hz
F2P               -0.500 ppm
F2               -200.06 Hz
PPMCM            0.41667 ppm/cm
HZCM           166.72084 Hz/cm
```

13C spectrum

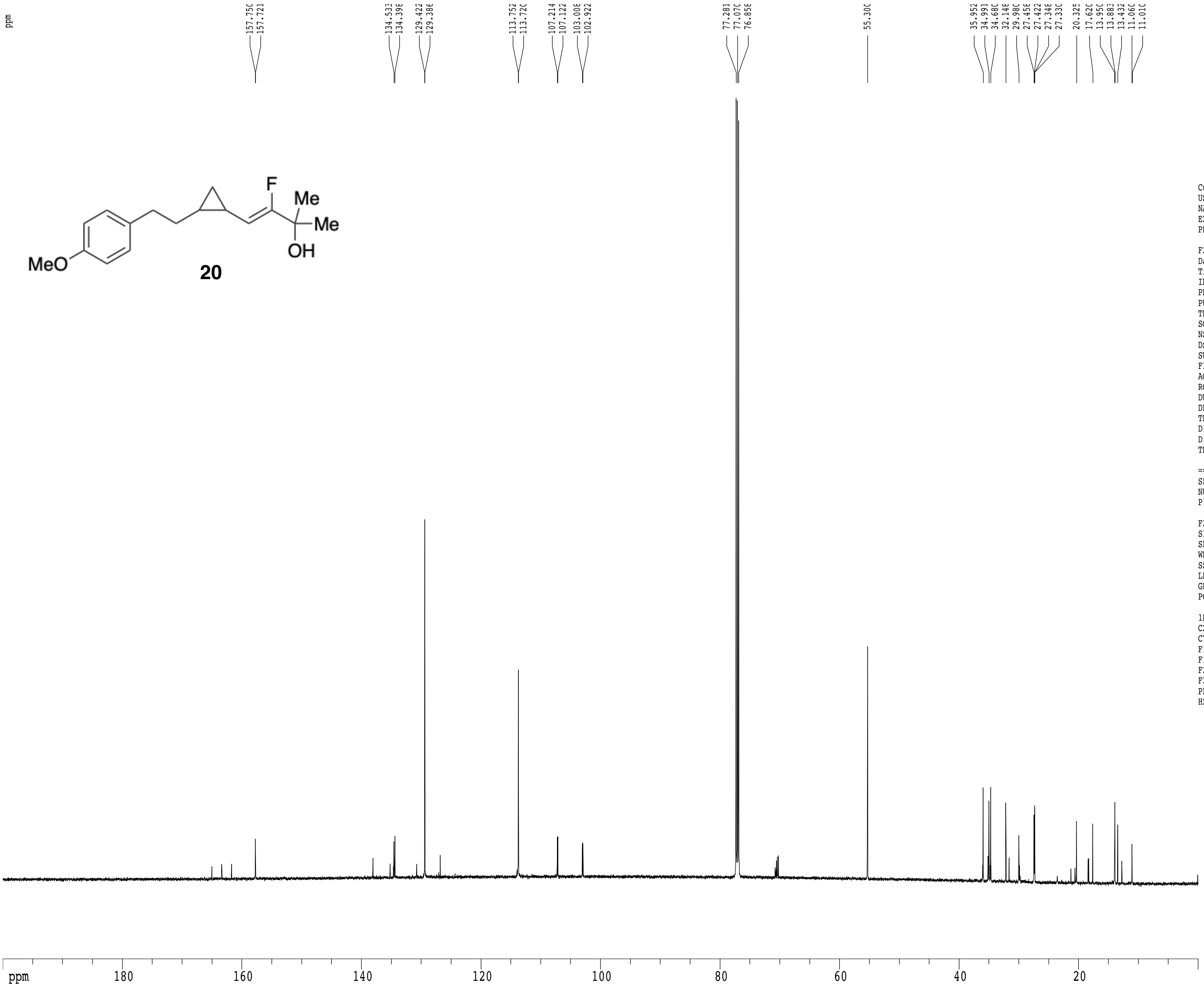


Current Data Parameters
USER mcginnit
NAME tmm-3-221-char
EXPNO 2
PROCNO 1

F2 - Acquisition Parameters
Date_ 20210906
Time 13.53
INSTRUM av600
PROBHD 5 mm CPBBO BB-
PULPROG zgdc30
TD 65536
SOLVENT CDCl3
NS 331
DS 4
SWH 36231.883 Hz
FIDRES 0.552855 Hz
AQ 0.9044468 sec
RG 2050
DW 13.800 usec
DE 19.63 usec
TE 298.0 K
D1 0.40000001 sec
D11 0.03000000 sec
TD0 1

======== CHANNEL f1 ========
SFO1 150.9194080 MHz
NUC1 13C
P1 10.10 usec

F2 - Processing parameters
SI 65536
SF 150.9028085 MHz
WDW EM
SSB 0
LB 1.00 Hz
GB 0
PC 1.00

1D NMR plot parameters
CX 22.80 cm
CY 15.00 cm
F1P 200.000 ppm
F1 30180.56 Hz
F2P 0.000 ppm
F2 0.00 Hz
PPMCM 8.77193 ppm/cm
HZCM 1323.70886 Hz/cm

19F spectrum

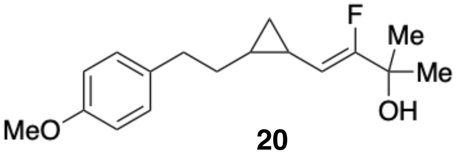


**20**

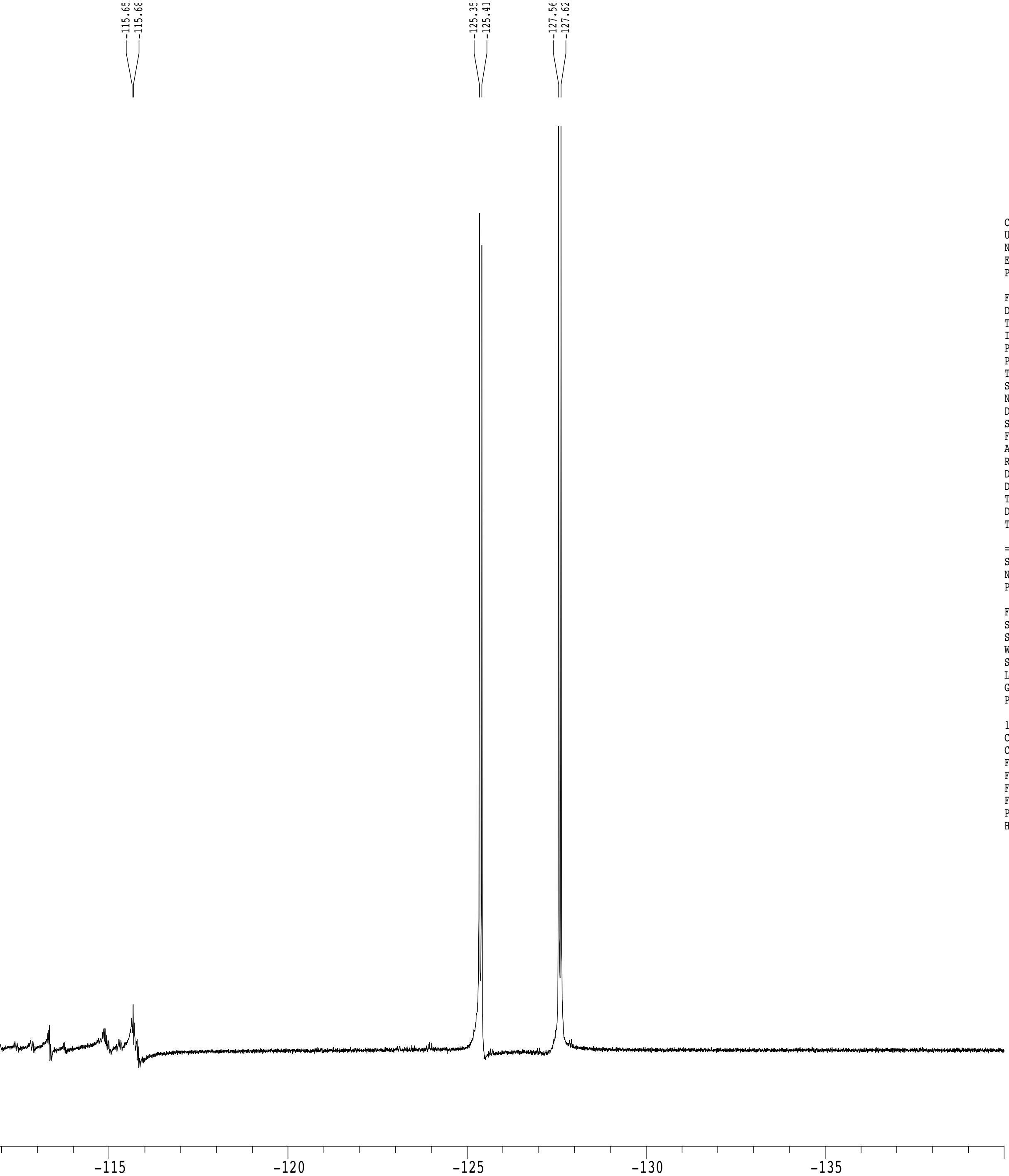


```
Current Data Parameters
USER          mcginnit
NAME     tmm-3-221-fnmr
EXPNO                2
PROCNO               1

F2 - Acquisition Parameters
Date_         20210907
Time             14.30
INSTRUM          av600
PROBHD   5 mm CPBBO BB-
PULPROG        zgflqn30
TD              131072
SOLVENT          CDCl3
NS                  16
DS                   2
SWH         178571.422 Hz
FIDRES        1.362392 Hz
AQ           0.3670516 sec
RG                 575
DW               2.800 usec
DE               18.00 usec
TE               298.0 K
D1          3.00000000 sec
TD0                  1

======== CHANNEL f1 ========
SFO1       564.6299196 MHz
NUC1               19F
P1               18.25 usec

F2 - Processing parameters
SI              131072
SF         564.6863858 MHz
WDW                 no
SSB                  0
LB                0.00 Hz
GB                   0
PC                1.00

1D NMR plot parameters
CX               22.80 cm
CY               15.00 cm
F1P           -100.000 ppm
F1           -56468.64 Hz
F2P           -140.000 ppm
F2           -79056.09 Hz
PPMCM          1.75439 ppm/cm
HZCM         990.67792 Hz/cm
```
